# Publisher Correction: Phosphoric acid-catalyzed atroposelective construction of axially chiral arylpyrroles

**DOI:** 10.1038/s41467-019-10071-w

**Published:** 2019-04-26

**Authors:** Lei Zhang, Shao-Hua Xiang, Jun (Joelle) Wang, Jian Xiao, Jun-Qi Wang, Bin Tan

**Affiliations:** 1 Department of Chemistry and Shenzhen Grubbs Institute, Southern University of Science and Technology, Shenzhen, 518055 China; 2 Academy for Advanced Interdisciplinary Studies, Southern University of Science and Technology, Shenzhen, 518055 China; 3 College of Chemistry and Pharmaceutical Sciences, Qingdao Agricultural University, Qingdao, 266109 China; 4 Department of Biology, Southern University of Science and Technology, Shenzhen, 518055 China

**Keywords:** Asymmetric catalysis, Homogeneous catalysis, Synthetic chemistry methodology

Correction to: *Nature Communications* 10.1038/s41467-019-08447-z, published online 4 February 2019.

The original PDF version of this Article contained an error in Table 3, in which all chemical structures were omitted and only the numerical data was shown. This has been corrected in the PDF version of the Article. The HTML version was correct from the time of publication.

