# Supplementary material for: Phosphoric acid-catalyzed atroposelective construction of axially chiral arylpyrroles
Source: Nat Commun. 2019 Feb 4;10:566. doi: 10.1038/s41467-019-08447-z (PMC6361918; doi:10.1038/s41467-019-08447-z)
Supplement: Supplementary file 1 — Supplementary Information [file 41467_2019_8447_MOESM1_ESM.pdf]

# Phosphoric Acid-Catalyzed Atroposelective Construction of Axially Chiral Arylpyrroles

Zhang *et al.*

## Supplementary Information

# Supplementary Figures

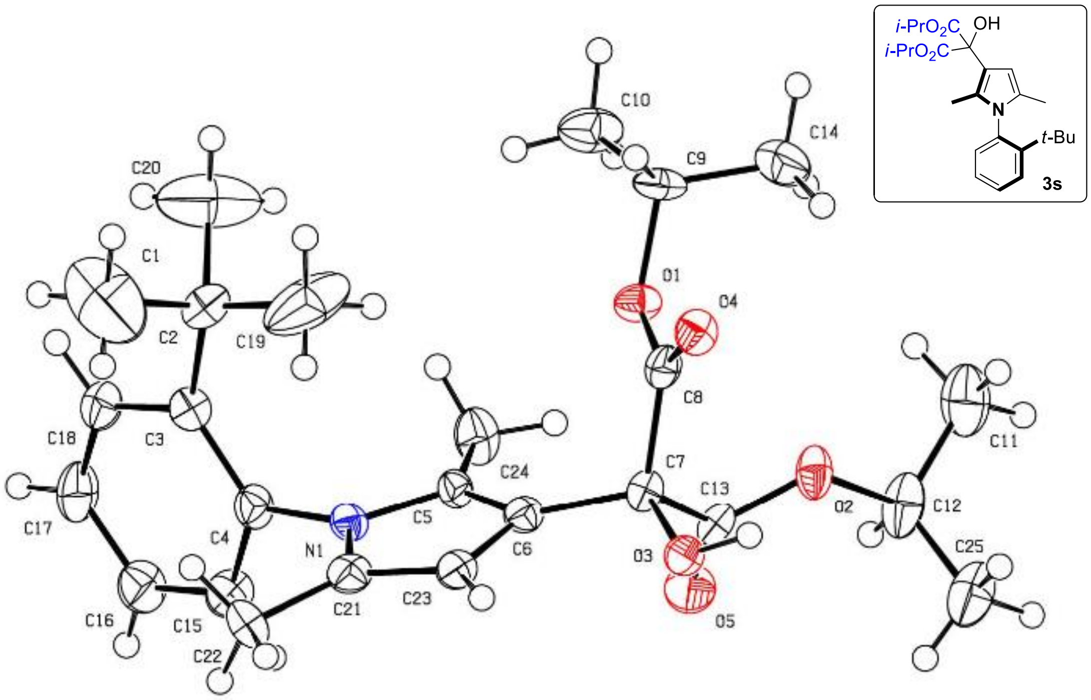


**Supplementary Figure 1.** X-ray crystal structure of **3s**.

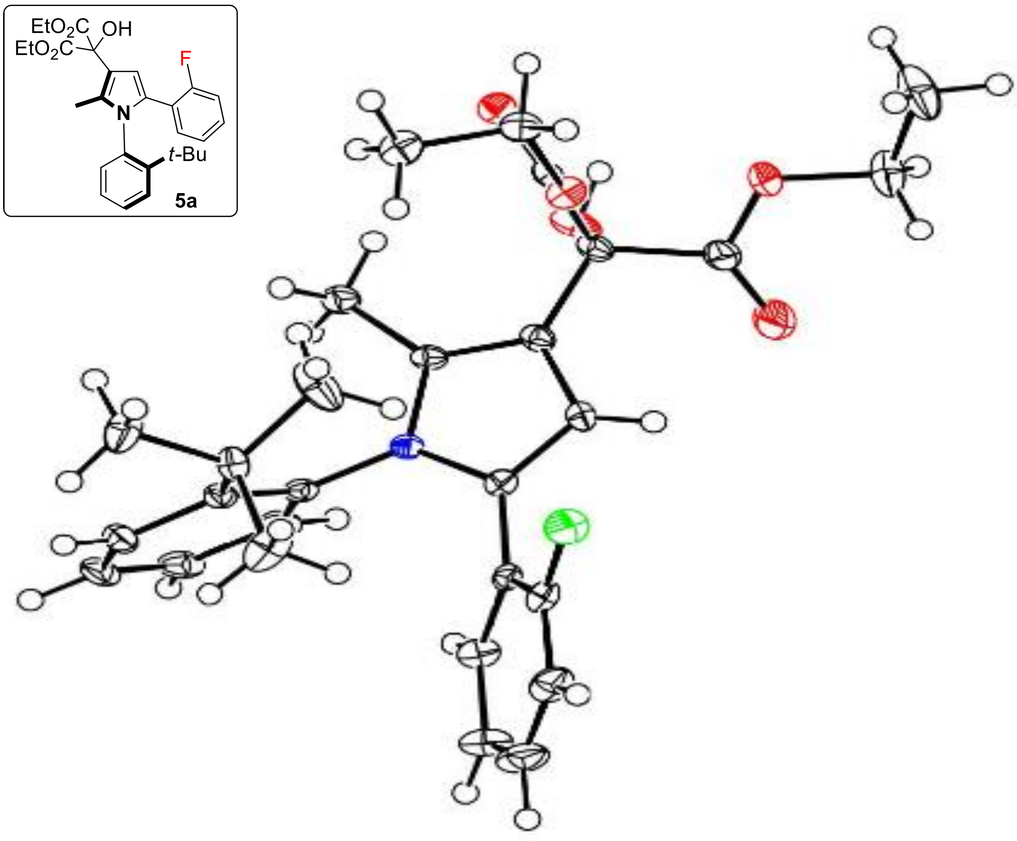


**Supplementary Figure 2.** X-ray crystal structure of **5a**.

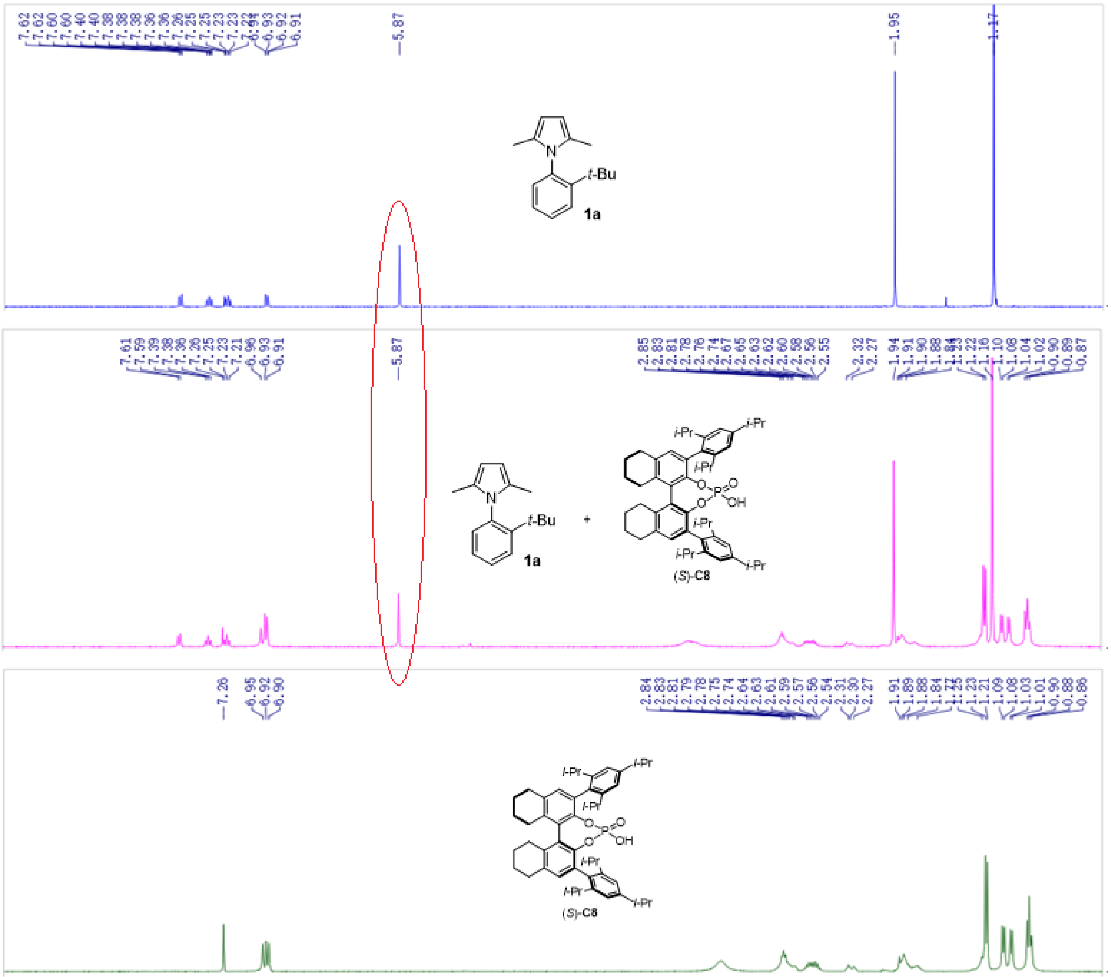


**Supplementary Figure 3.** $^{1}$H NMR monitoring experiments with **1a** and **CPA** in $CDCl_3$.

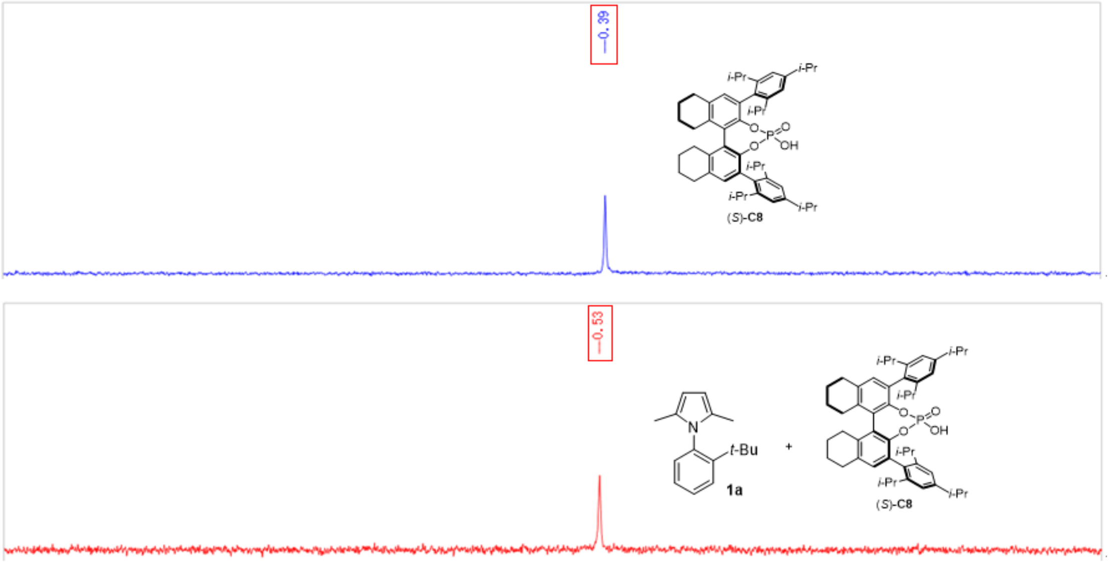


**Supplementary Figure 4.** $^{31}$P NMR monitoring experiments with **1a** and **CPA** in $CDCl_3$.

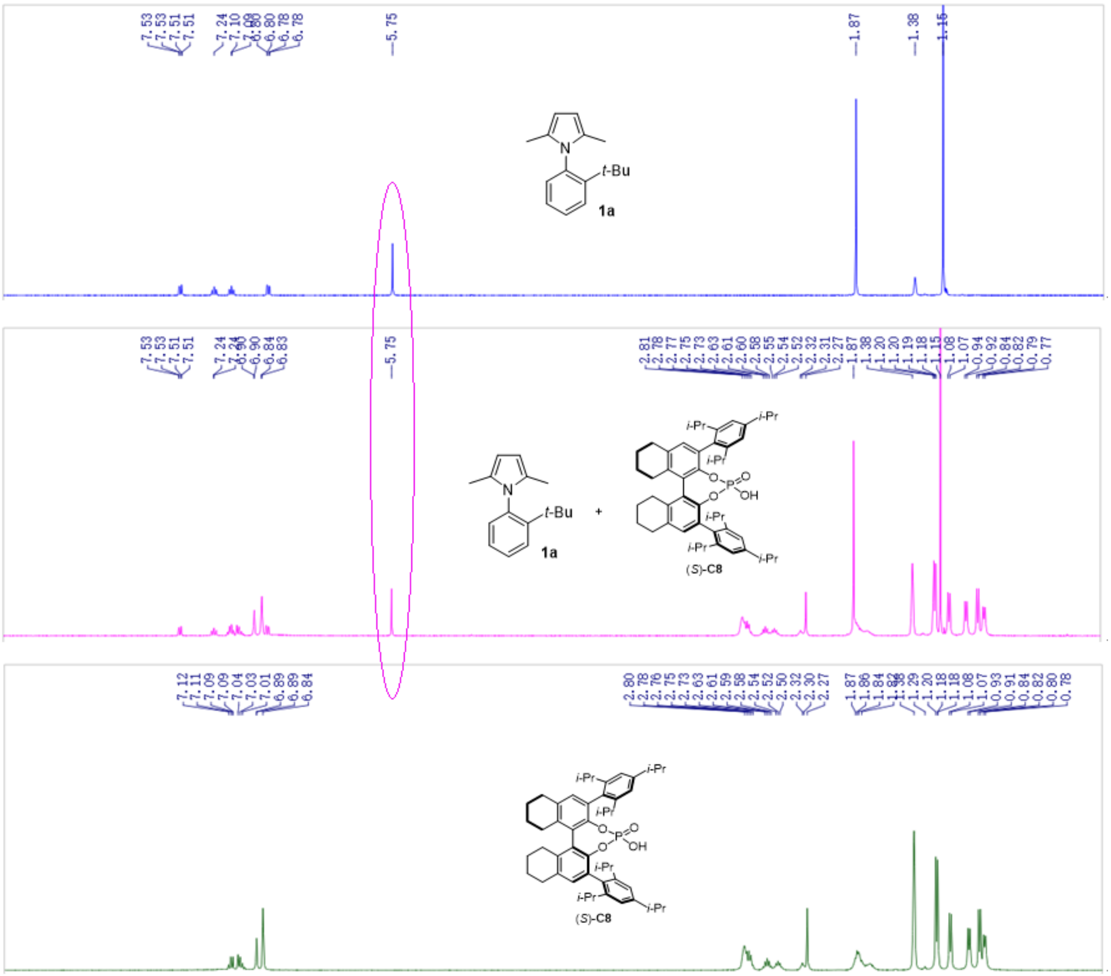


**Supplementary Figure 5.** $^{1}$H NMR monitoring experiments with **1a** and **CPA** in *c*-hexane-$d_{12}$.

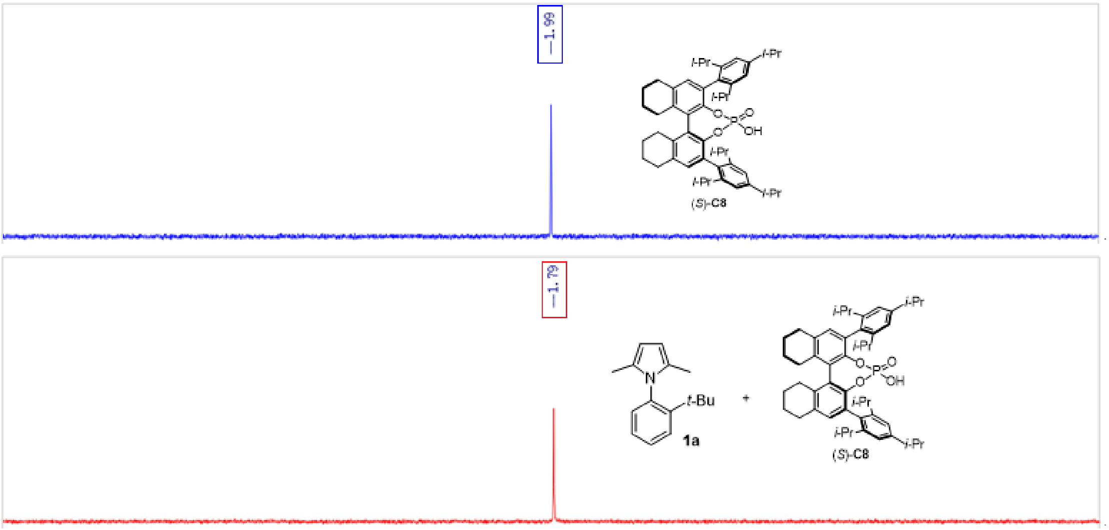


**Supplementary Figure 6.** $^{31}$P NMR monitoring experiments with **1a** and **CPA** in *c*-hexane-$d_{12}$.

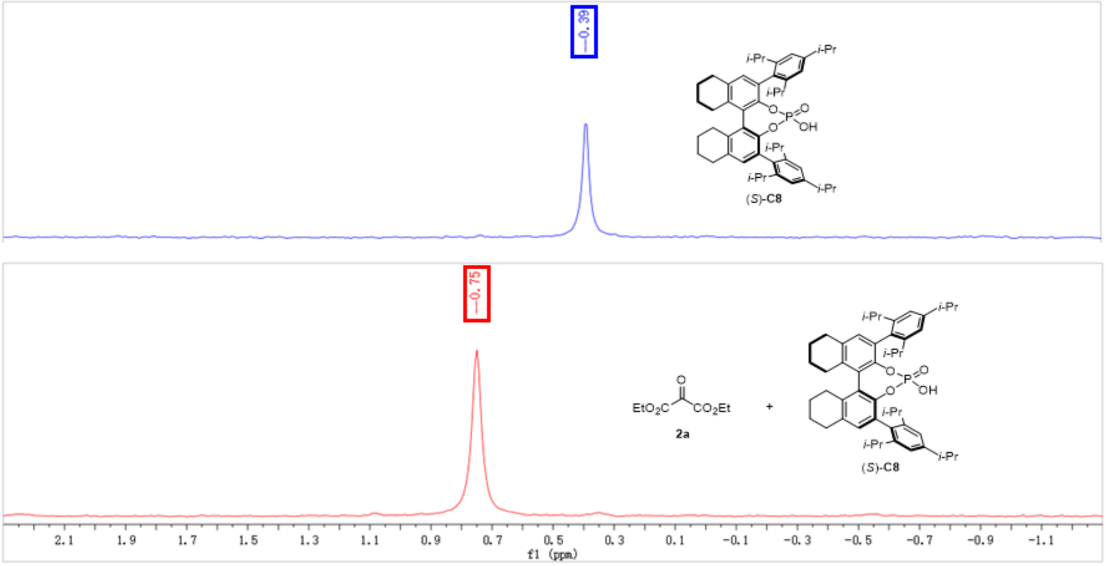


**Supplementary Figure 7.** $^{31}$P NMR monitoring experiments with **2a** and **CPA** in $CDCl_3$.

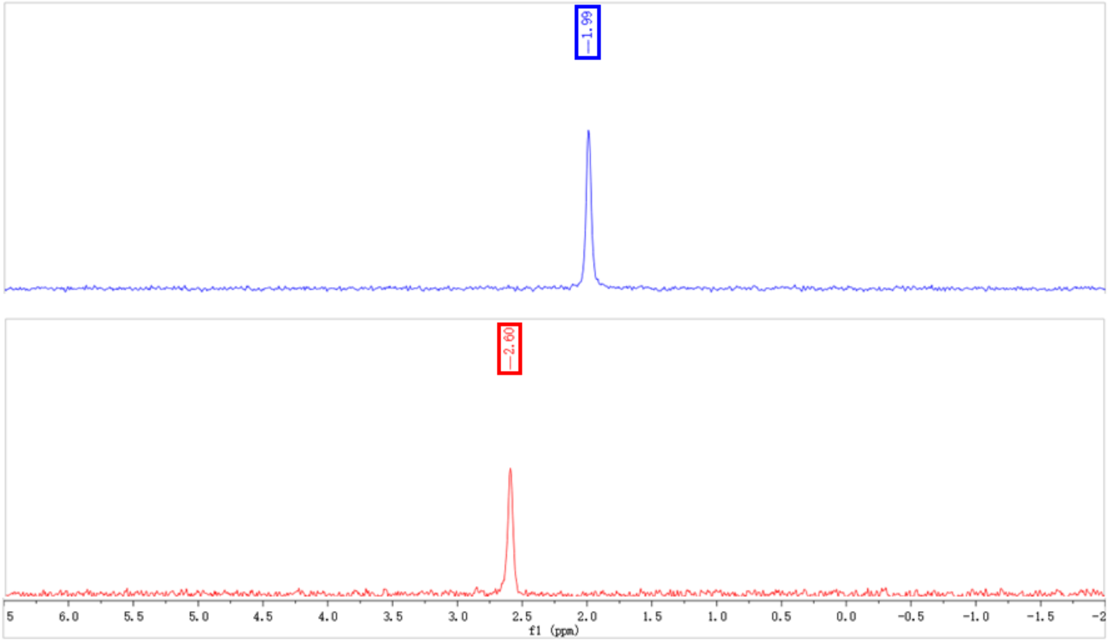


**Supplementary Figure 8.** $^{31}$P NMR monitoring experiments with **2a** and **CPA** in *c*-hexane-$d_{12}$.

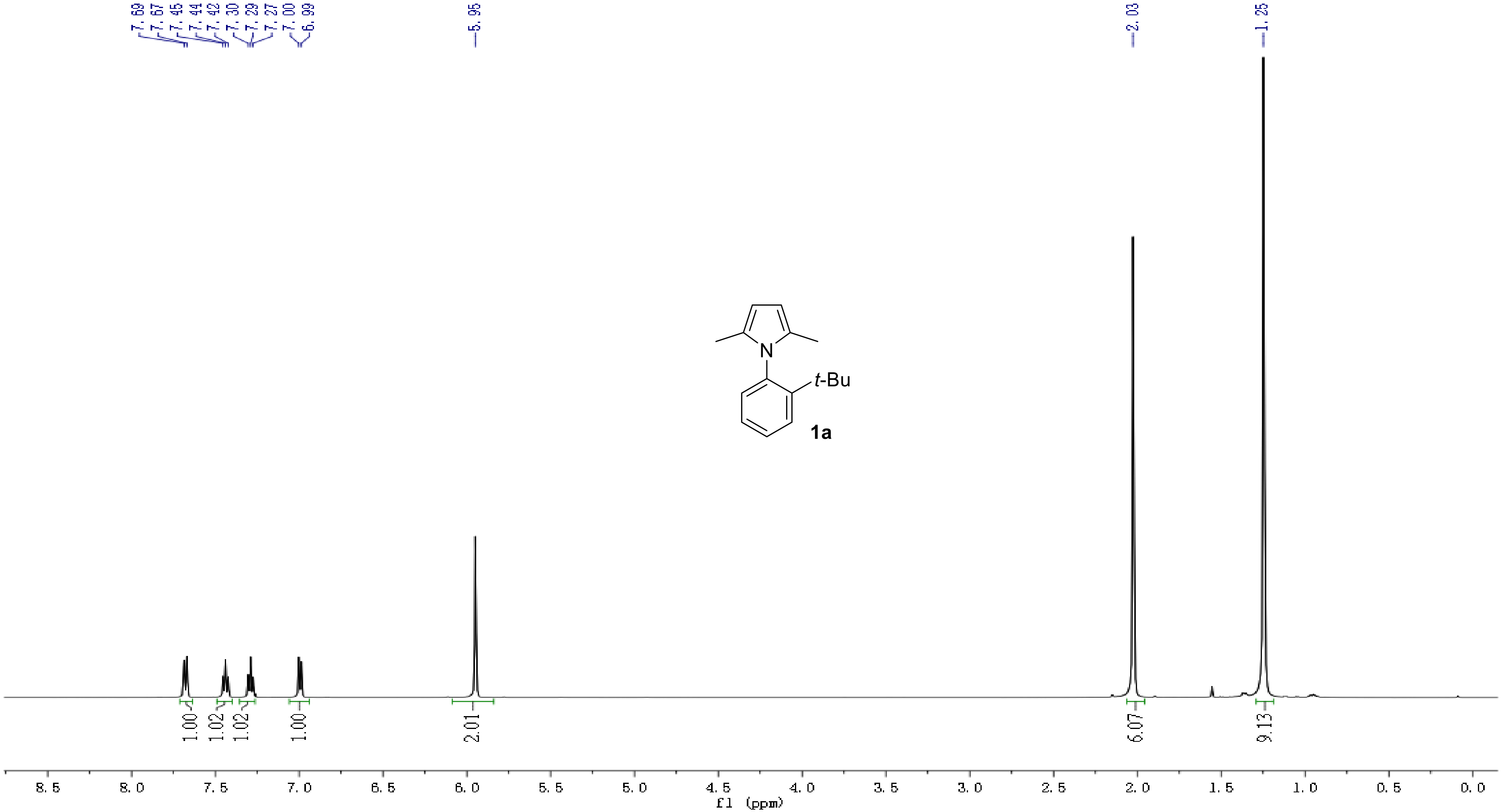


**Supplementary Figure 9.** $^{1}$H NMR of **1a**.

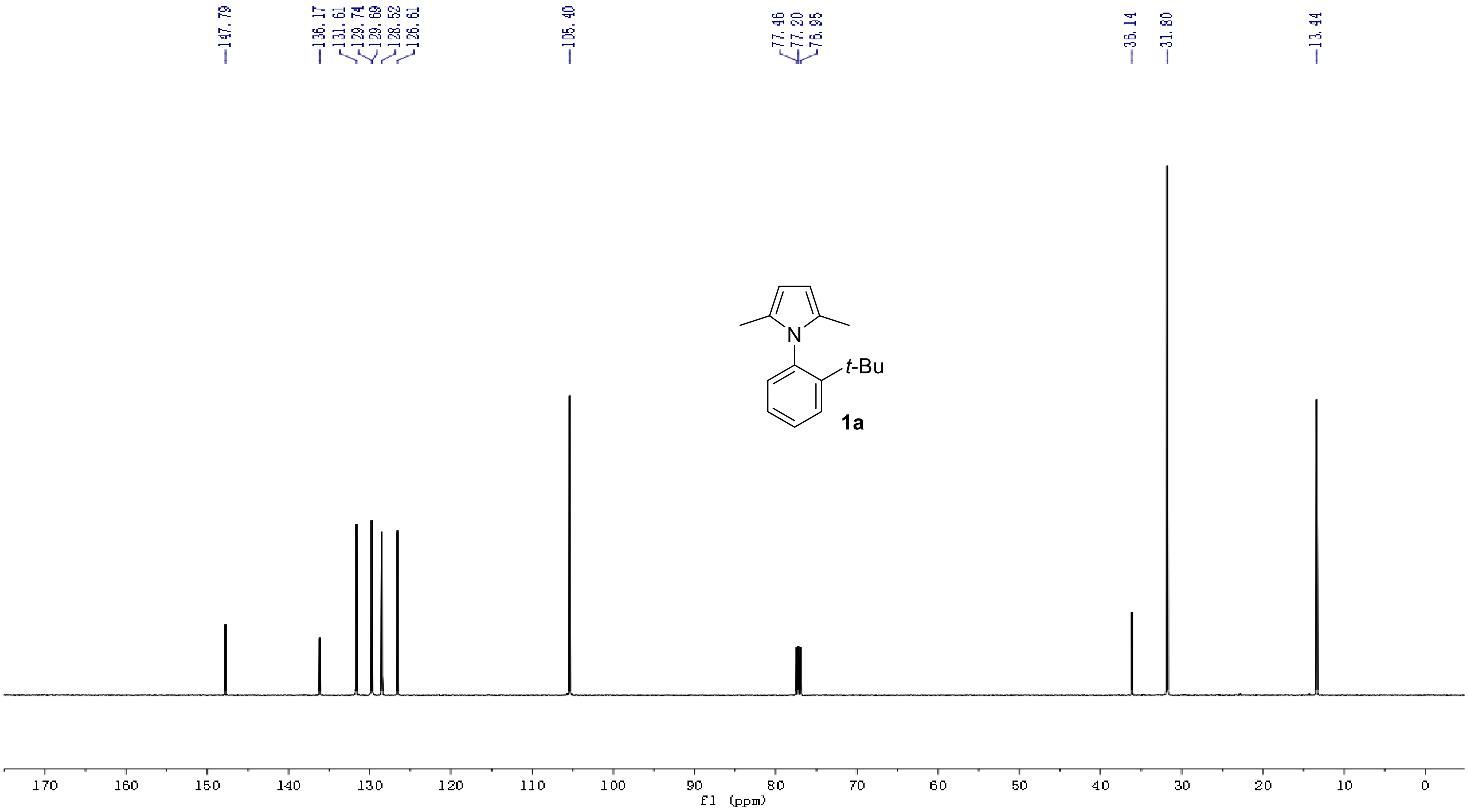


**Supplementary Figure 10.** $^{13}$C NMR of **1a**.

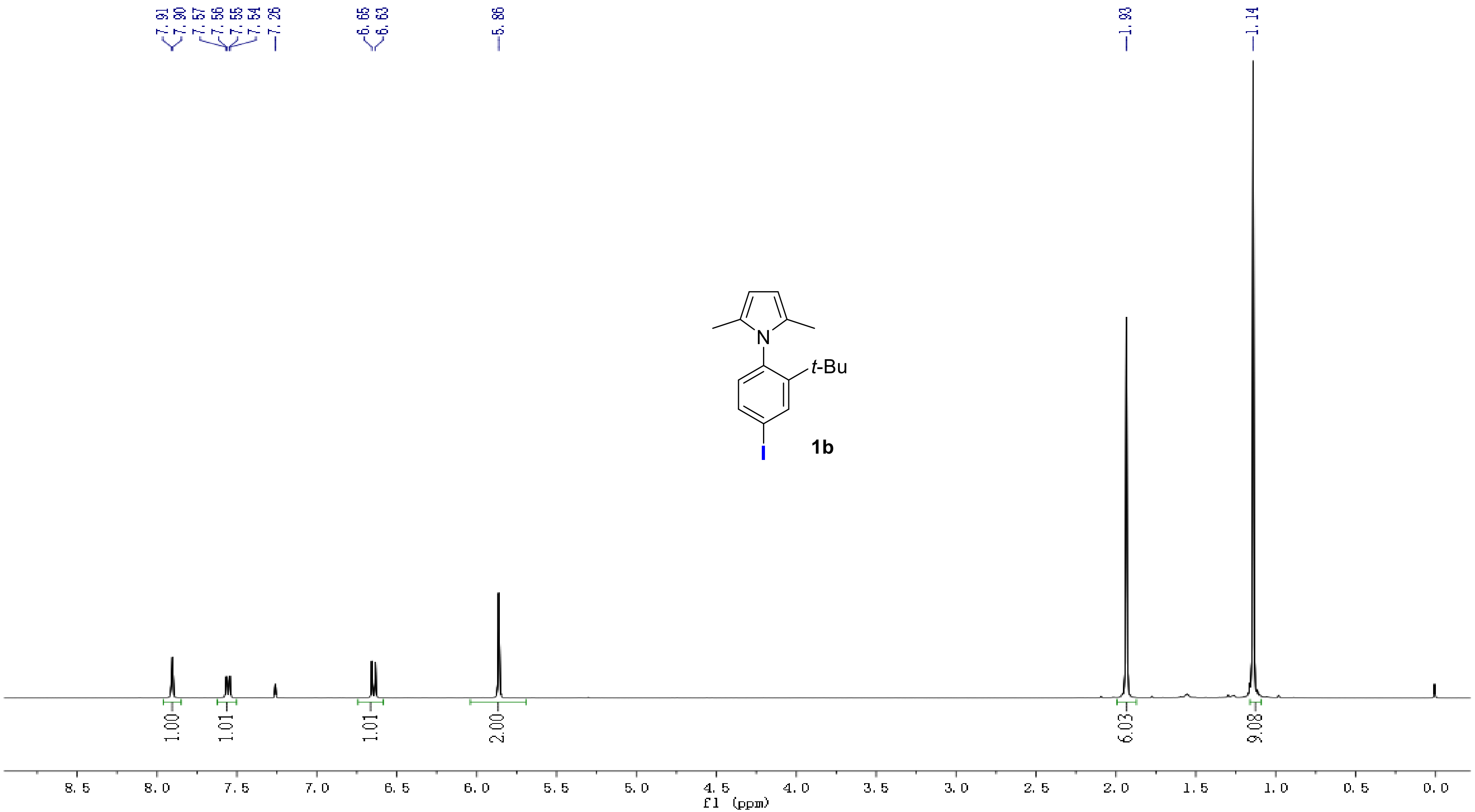


**Supplementary Figure 11.** $^{1}$H NMR of **1b**.

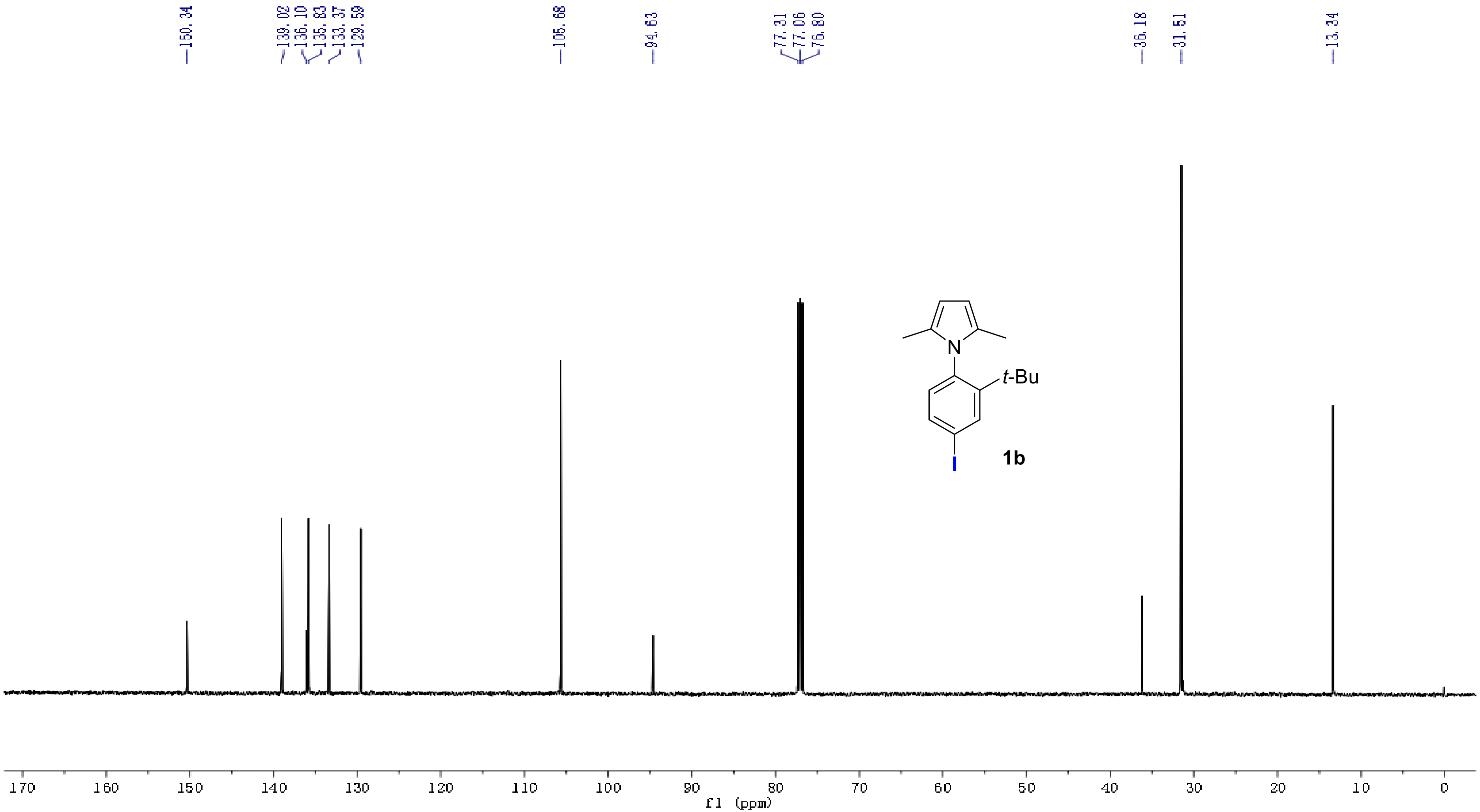


**Supplementary Figure 12.** $^{13}$C NMR of **1b**.

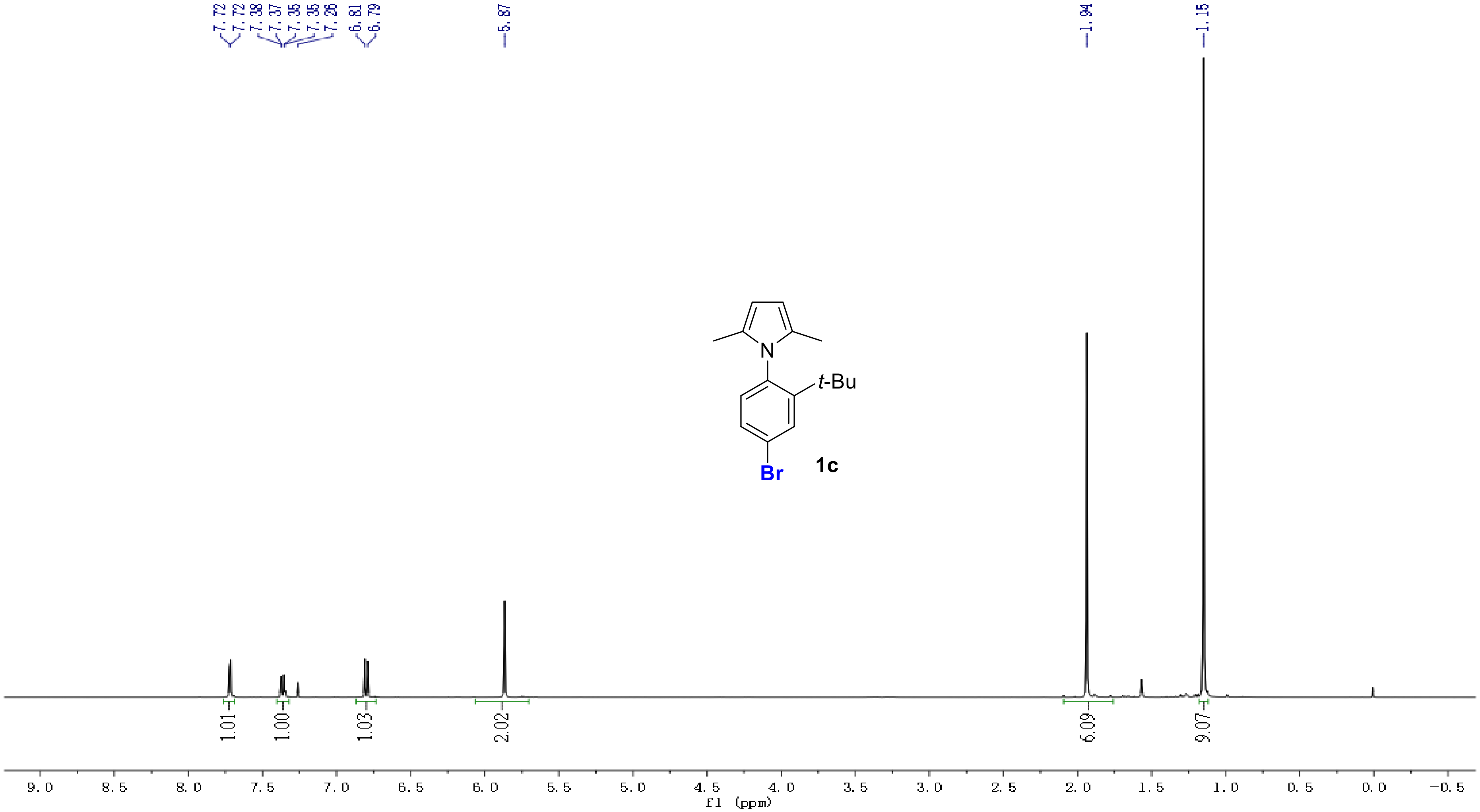


**Supplementary Figure 13.** $^{1}$H NMR of **1c**.

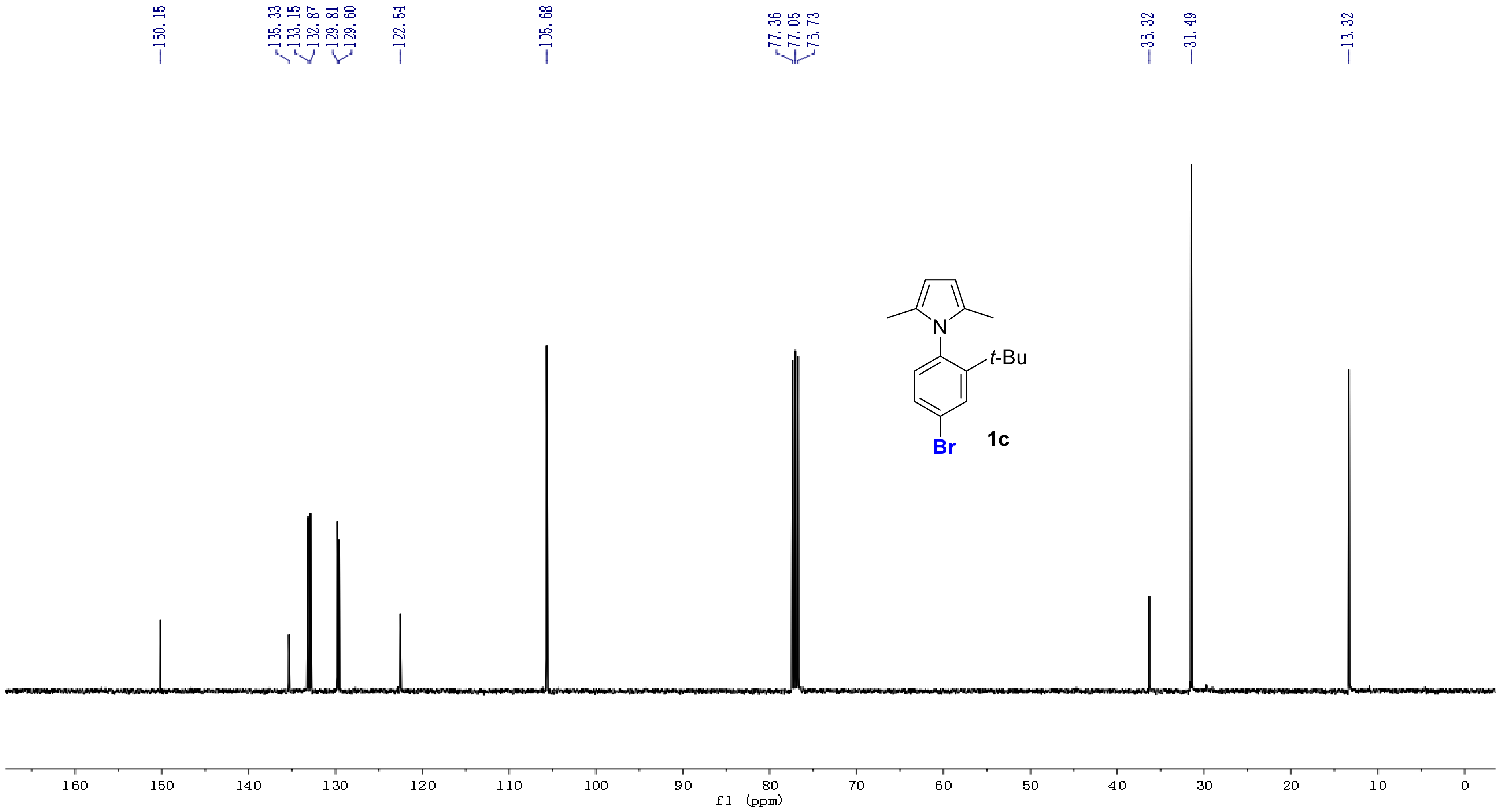


**Supplementary Figure 14.** $^{13}$C NMR of **1c**.

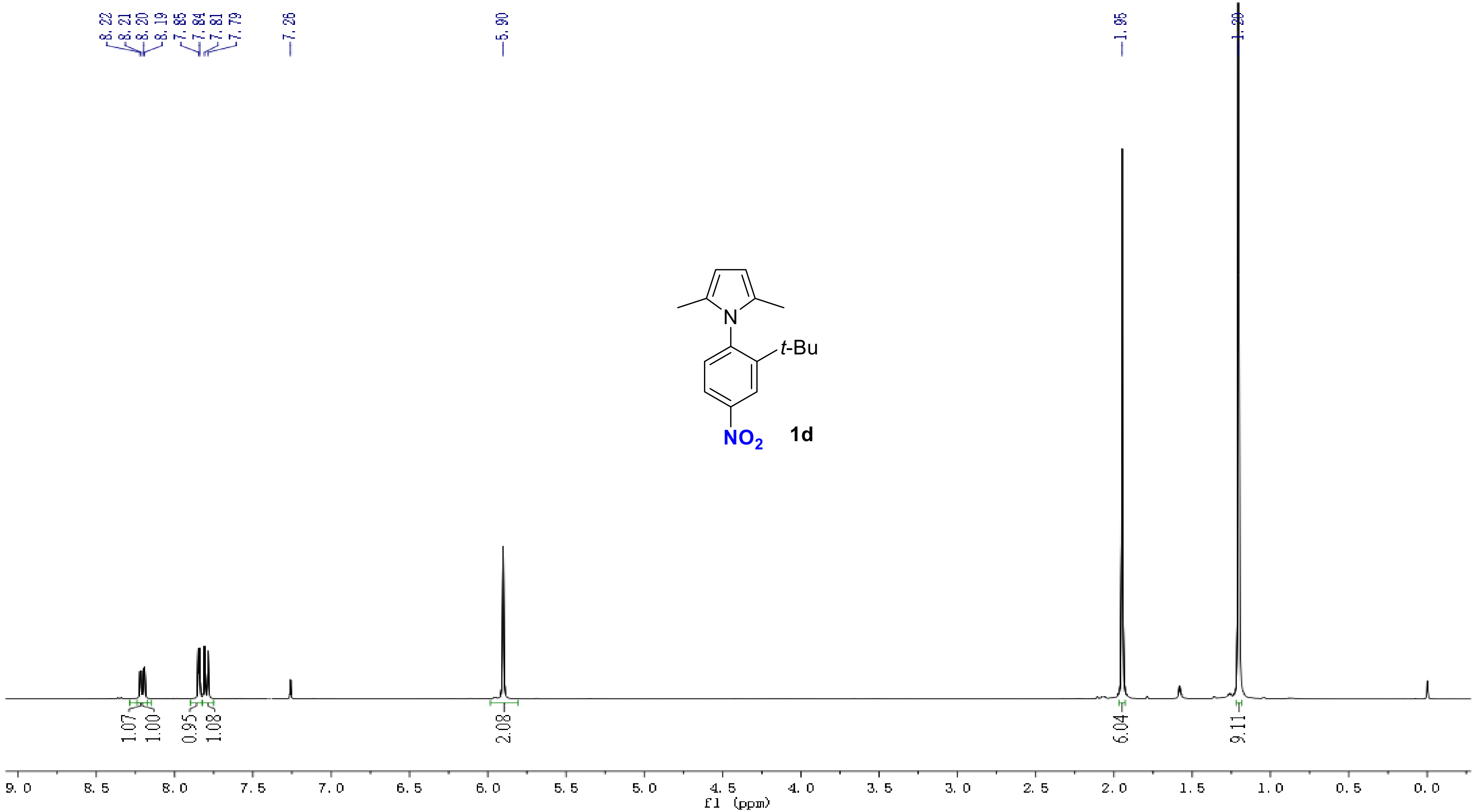


**Supplementary Figure 15.** $^1$H NMR of **1d**.

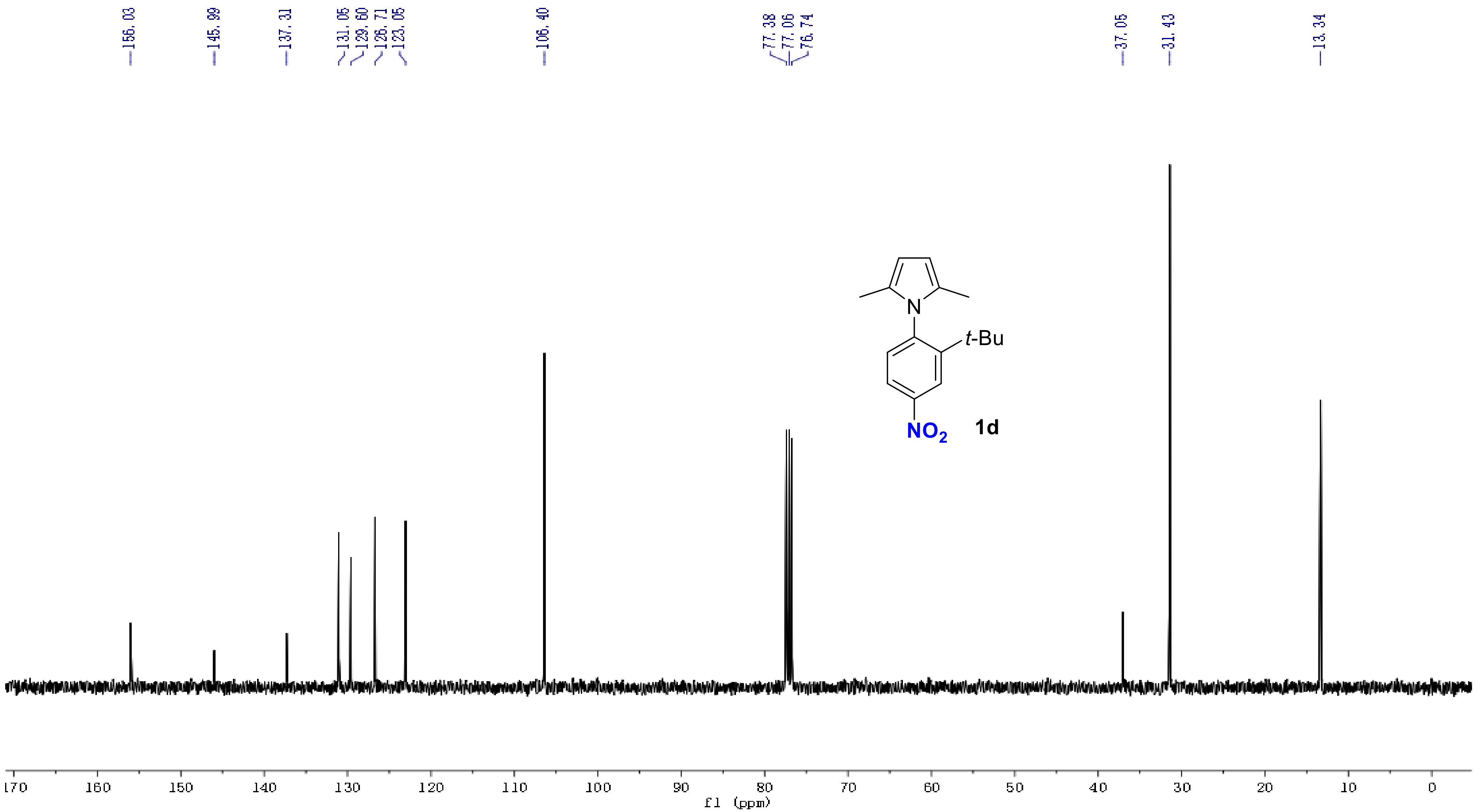


**Supplementary Figure 16.** $^{13}C$ NMR of **1d**.

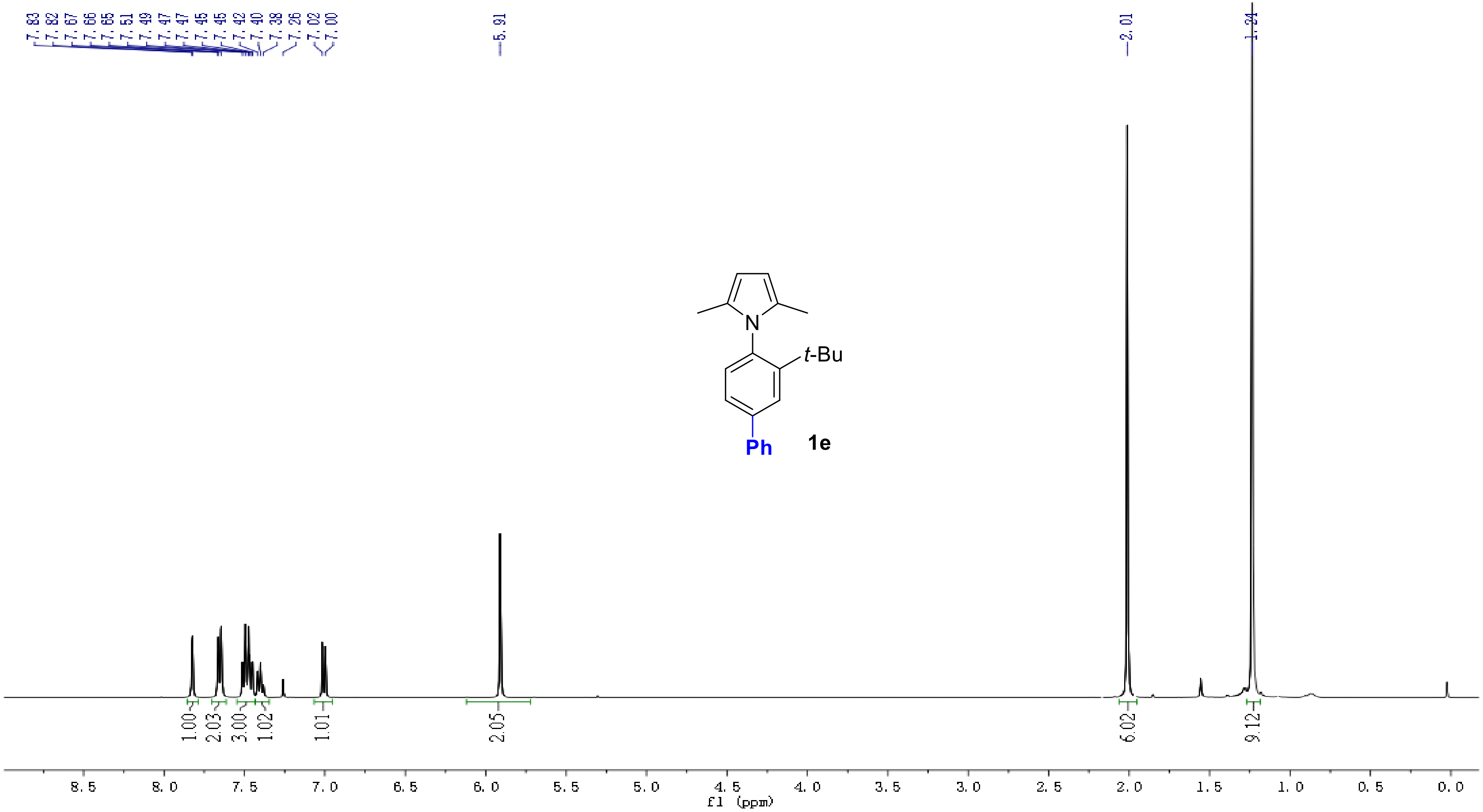


**Supplementary Figure 17.** $^{1}$H NMR of **1e**.

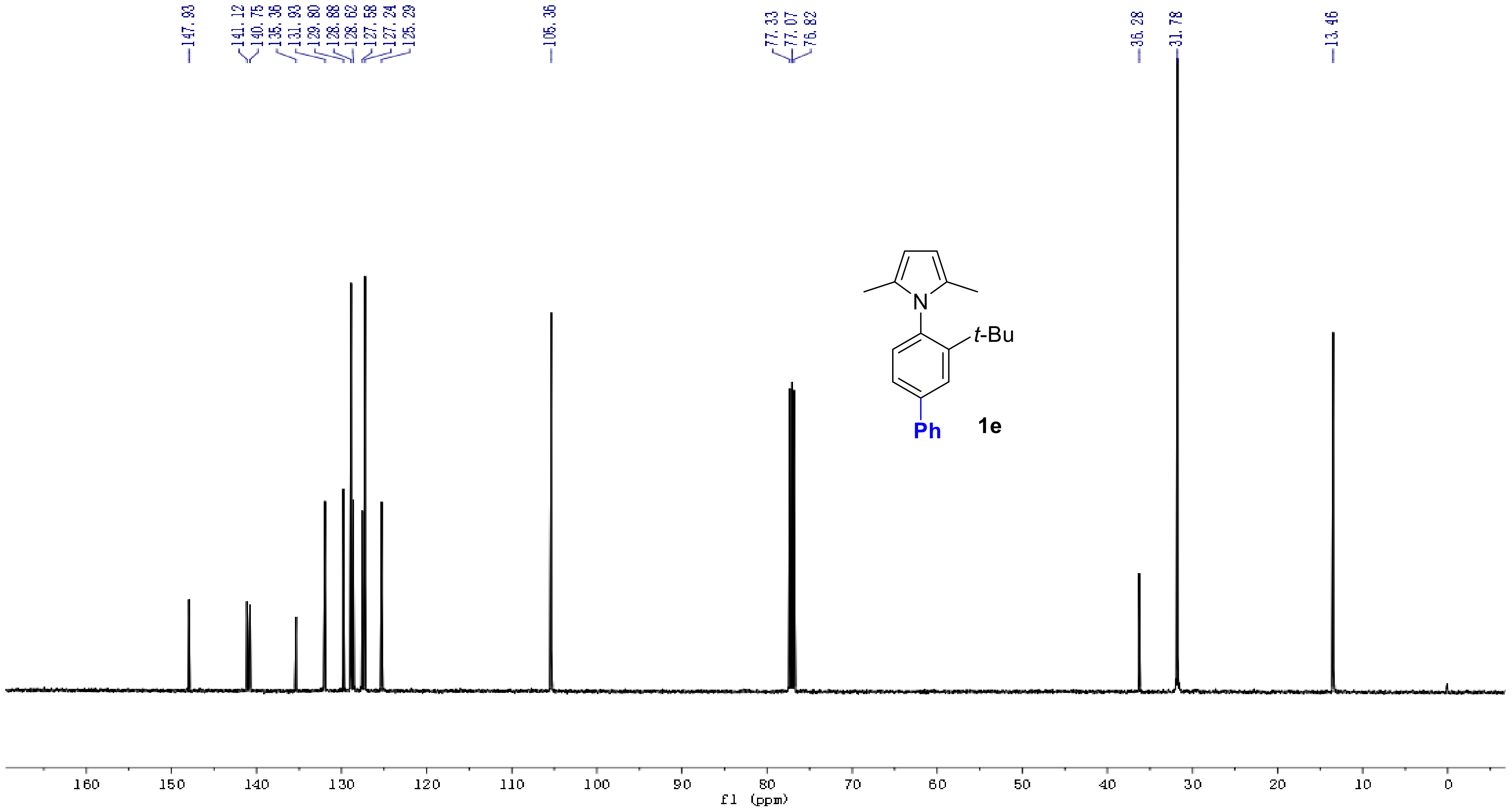


**Supplementary Figure 18.** $^{13}$C NMR of **1e**.

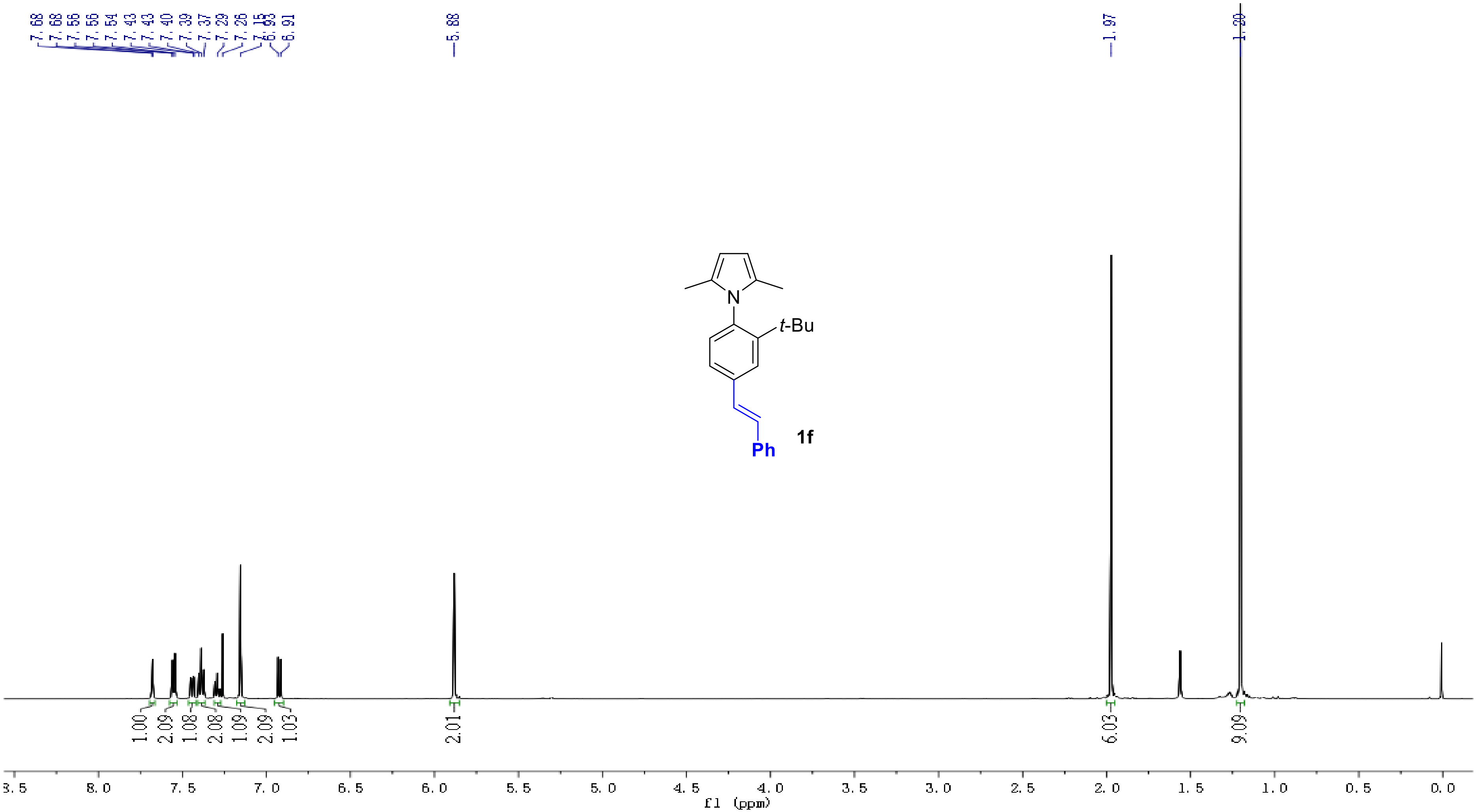

**Supplementary Figure 19.** $^1$H NMR of **1f**.

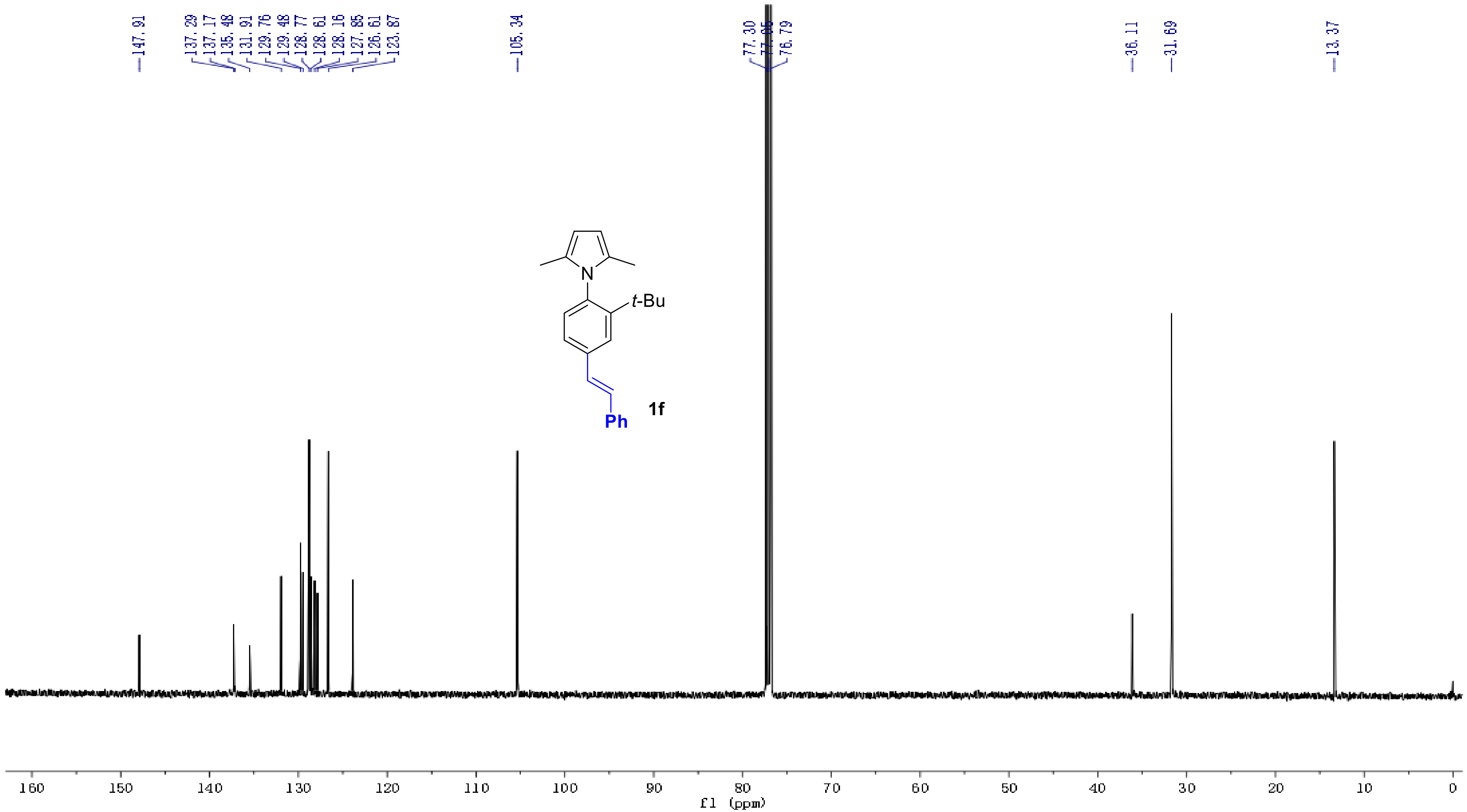


**Supplementary Figure 20.** $^{13}$C NMR of **1f**.

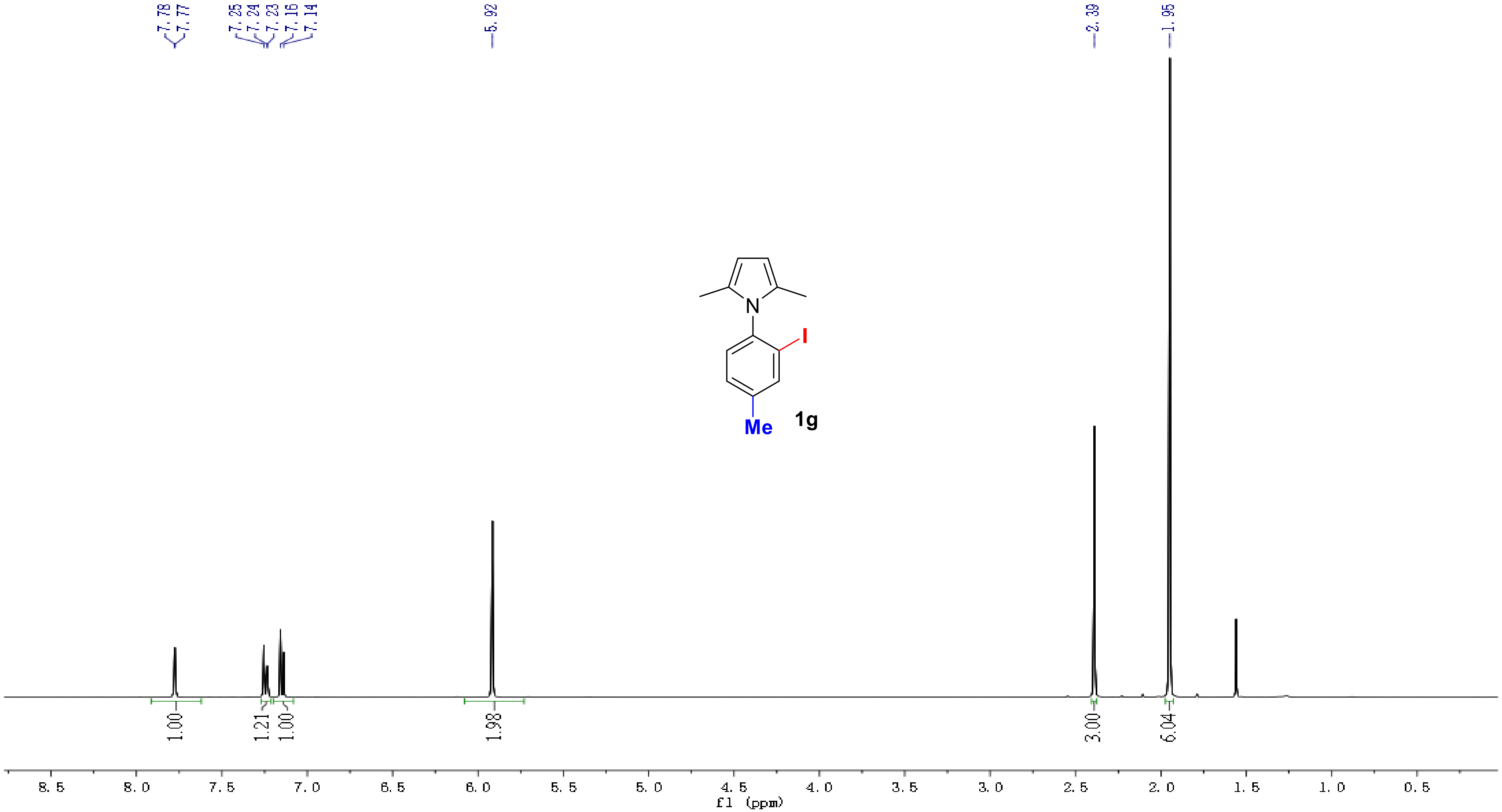


**Supplementary Figure 21.** $^{1}$H NMR of **1g**.

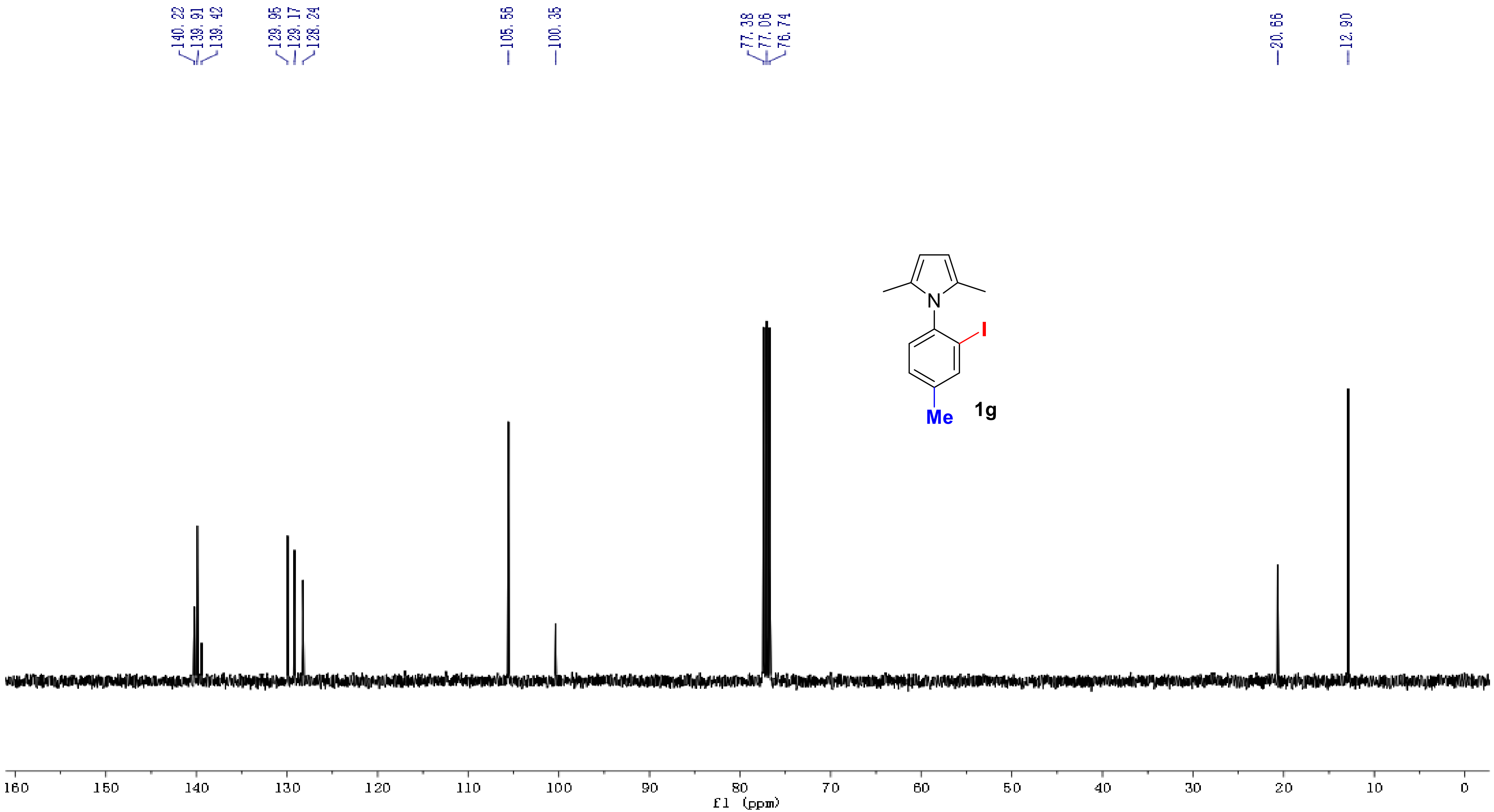


**Supplementary Figure 22.** $^{13}$C NMR of **1g**.

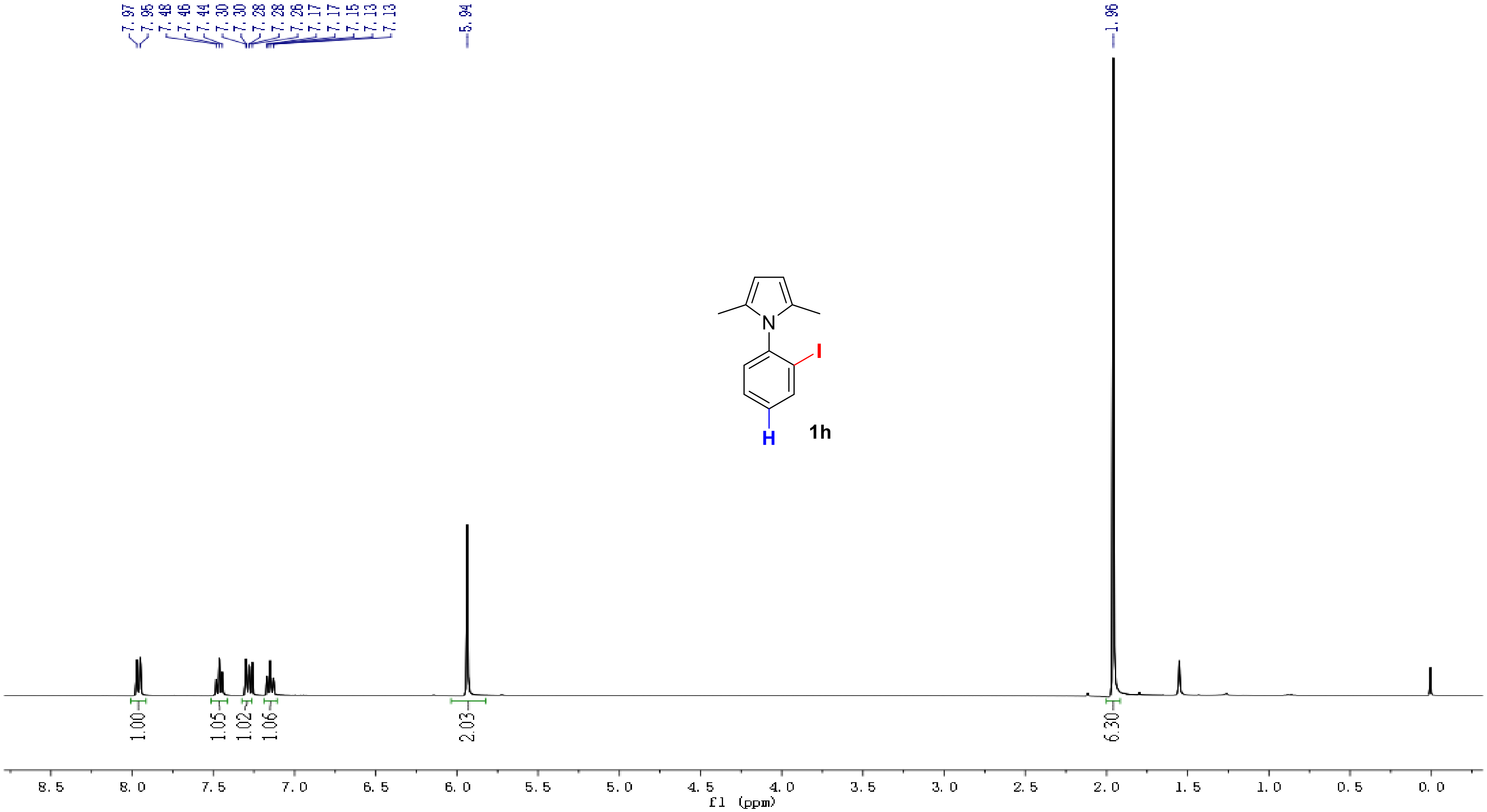


**Supplementary Figure 23.** $^{1}$H NMR of **1h**.

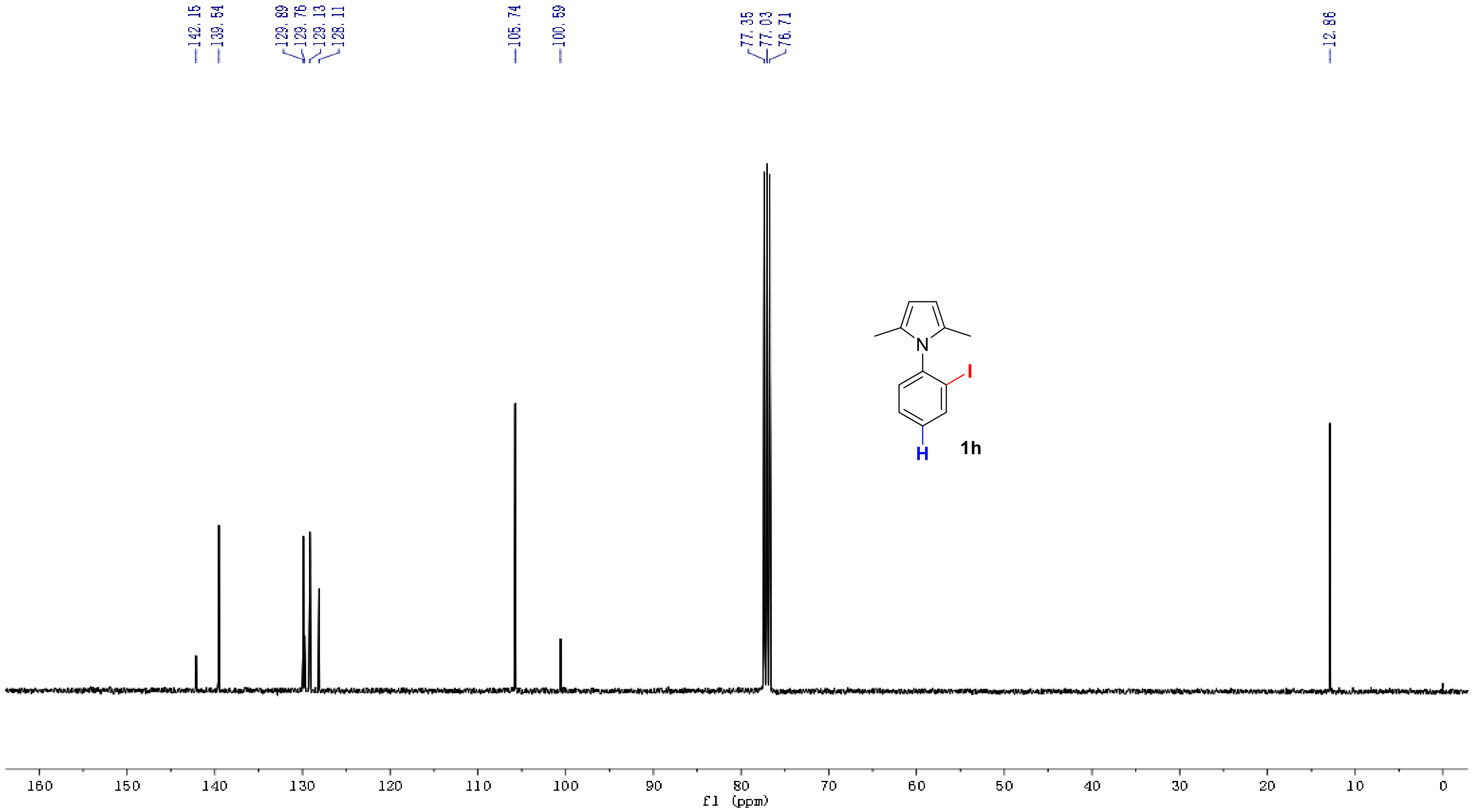


**Supplementary Figure 24.** $^{13}$C NMR of **1h**.

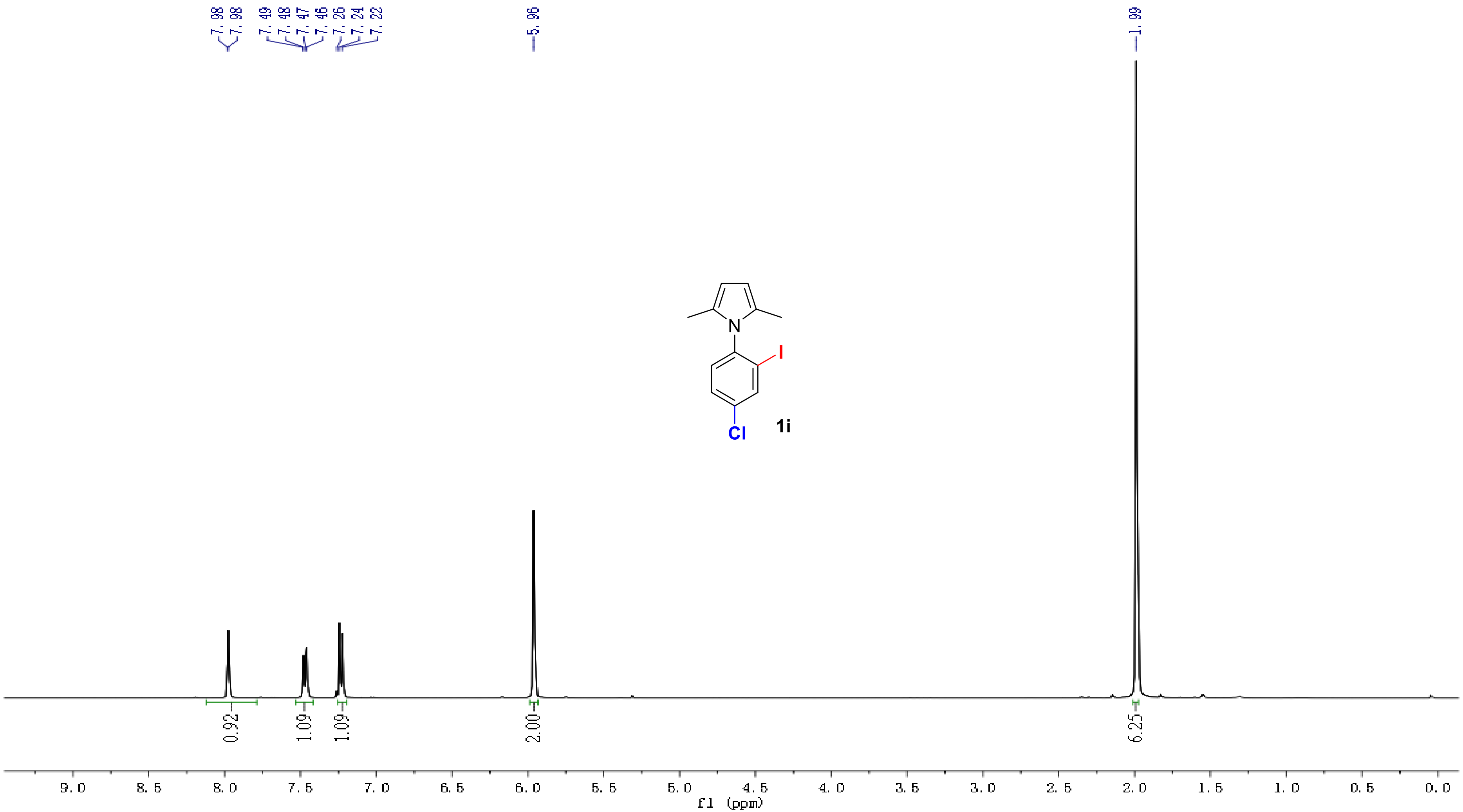


**Supplementary Figure 25.** $^{1}$H NMR of **1i**.

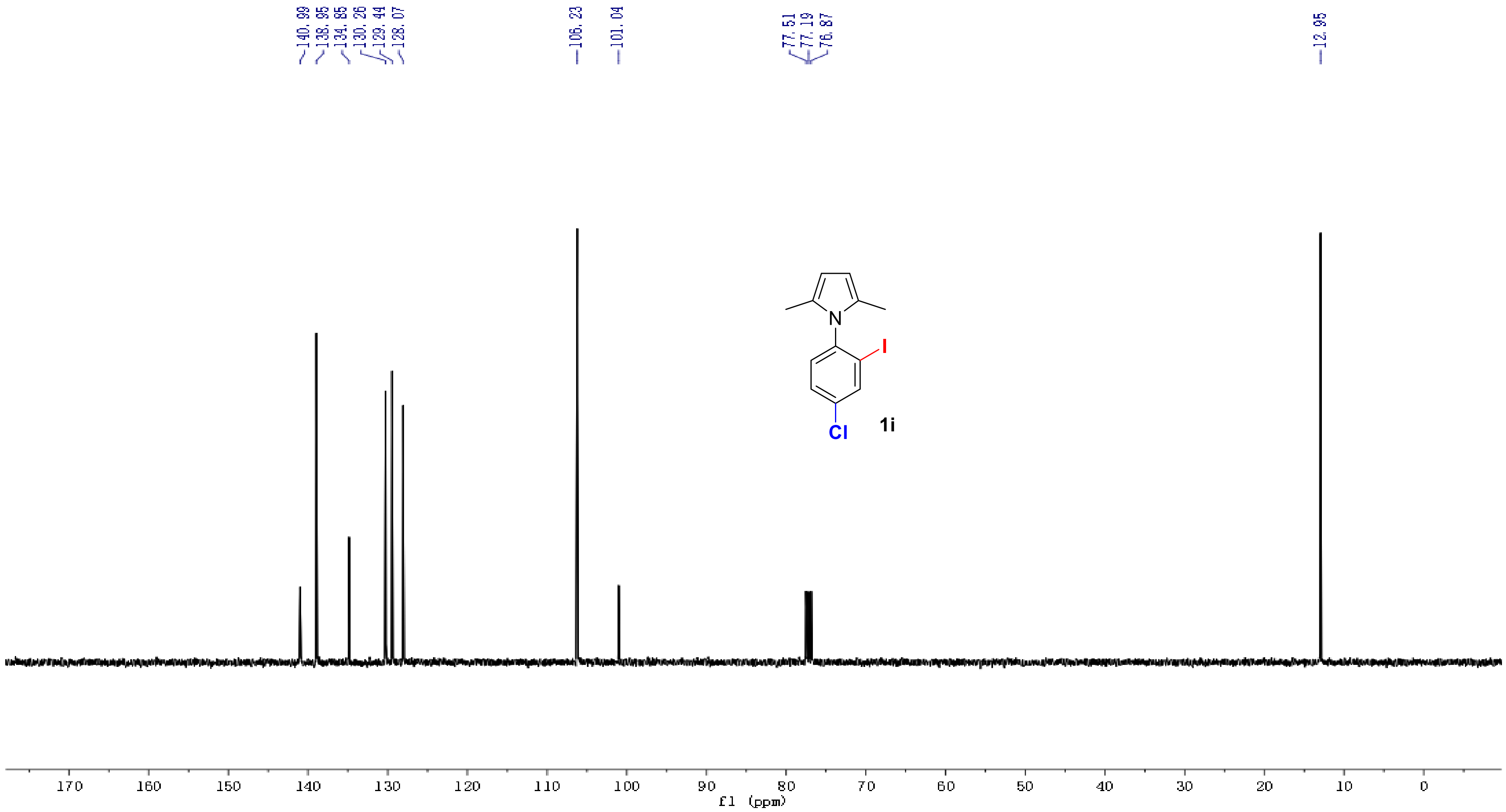


**Supplementary Figure 26.** $^{13}$C NMR of **1i**.

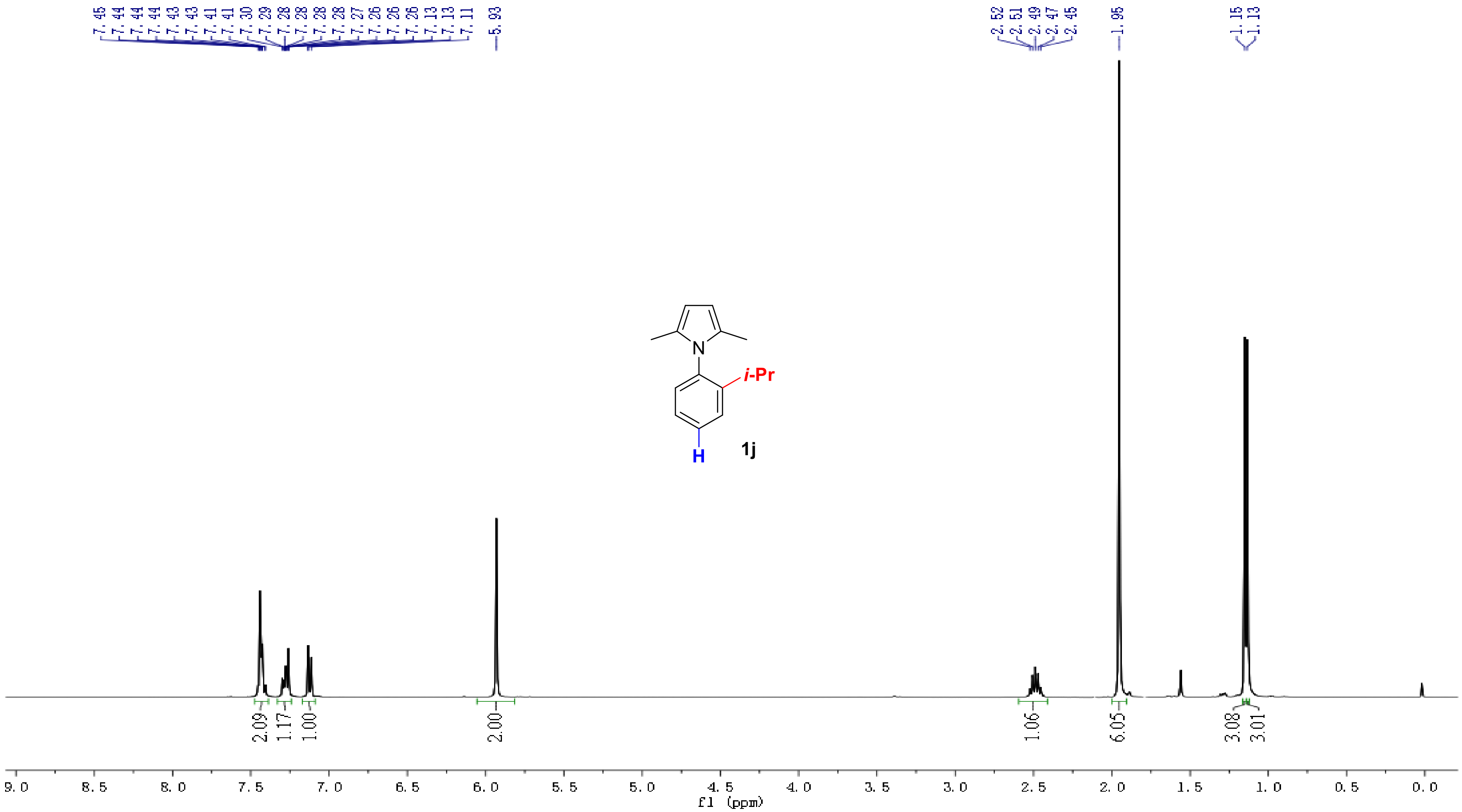


**Supplementary Figure 27.** $^{1}$H NMR of **1j**.

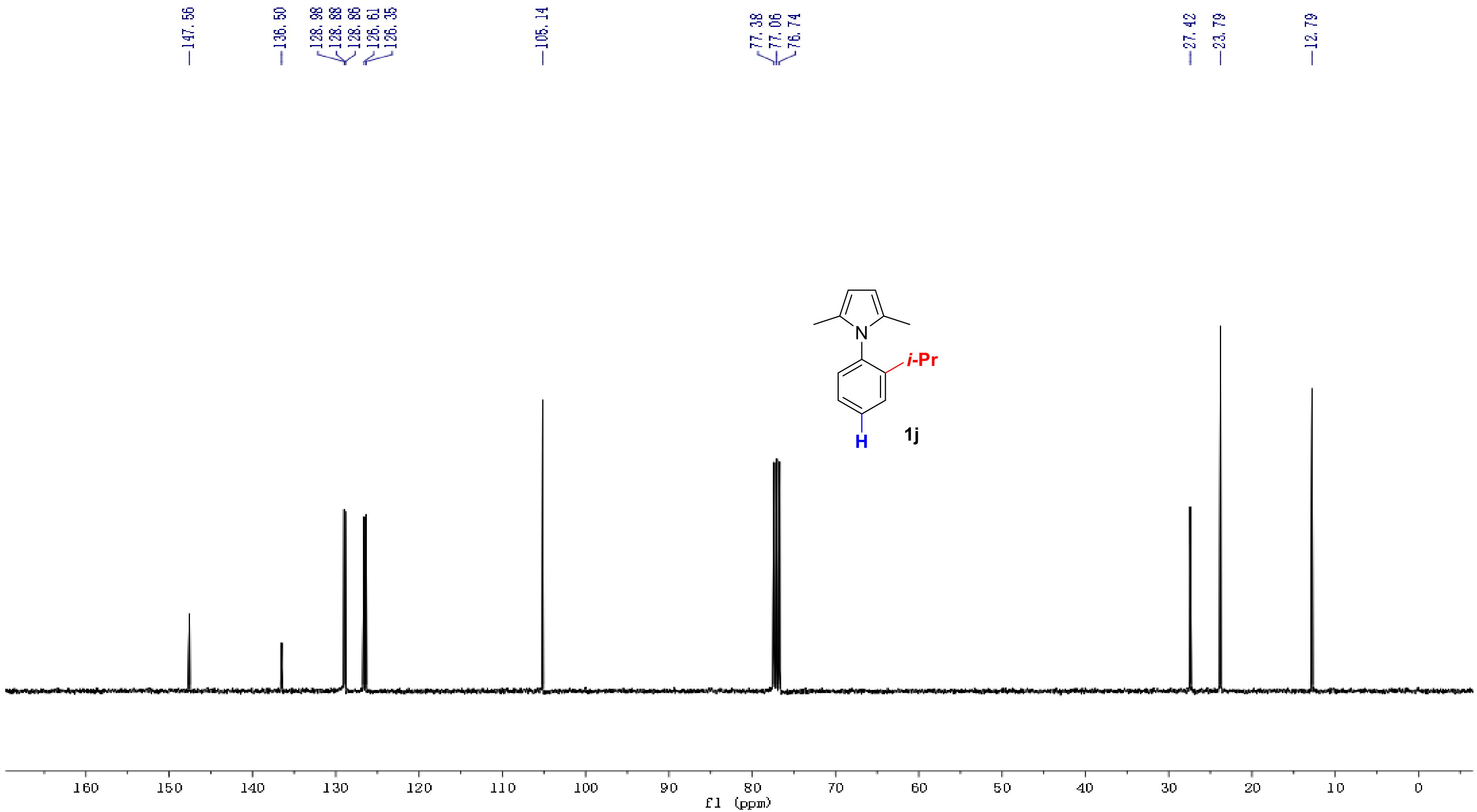


**Supplementary Figure 28.** $^{13}$C NMR of **1j**.

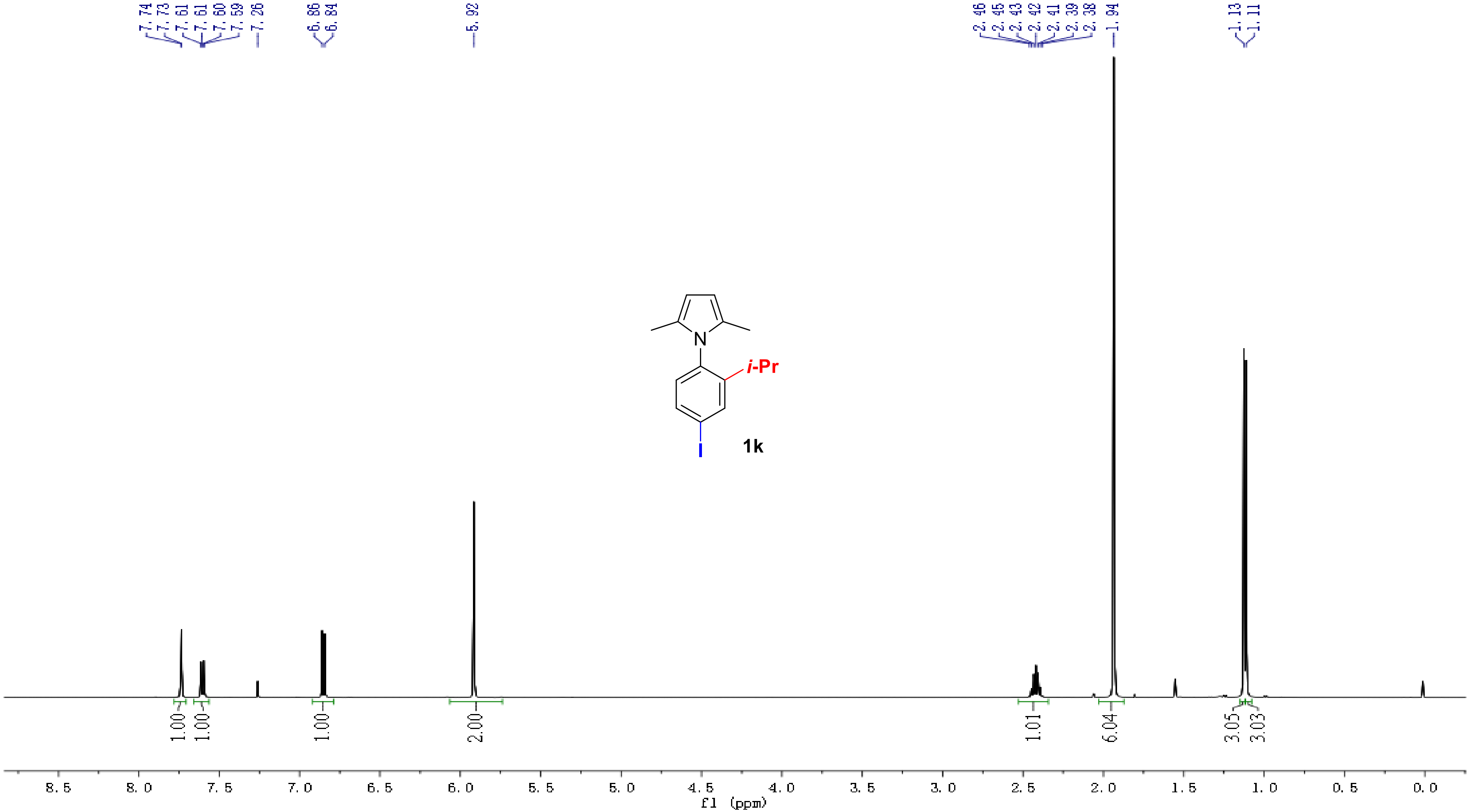


**Supplementary Figure 29.** $^{1}$H NMR of **1k**.

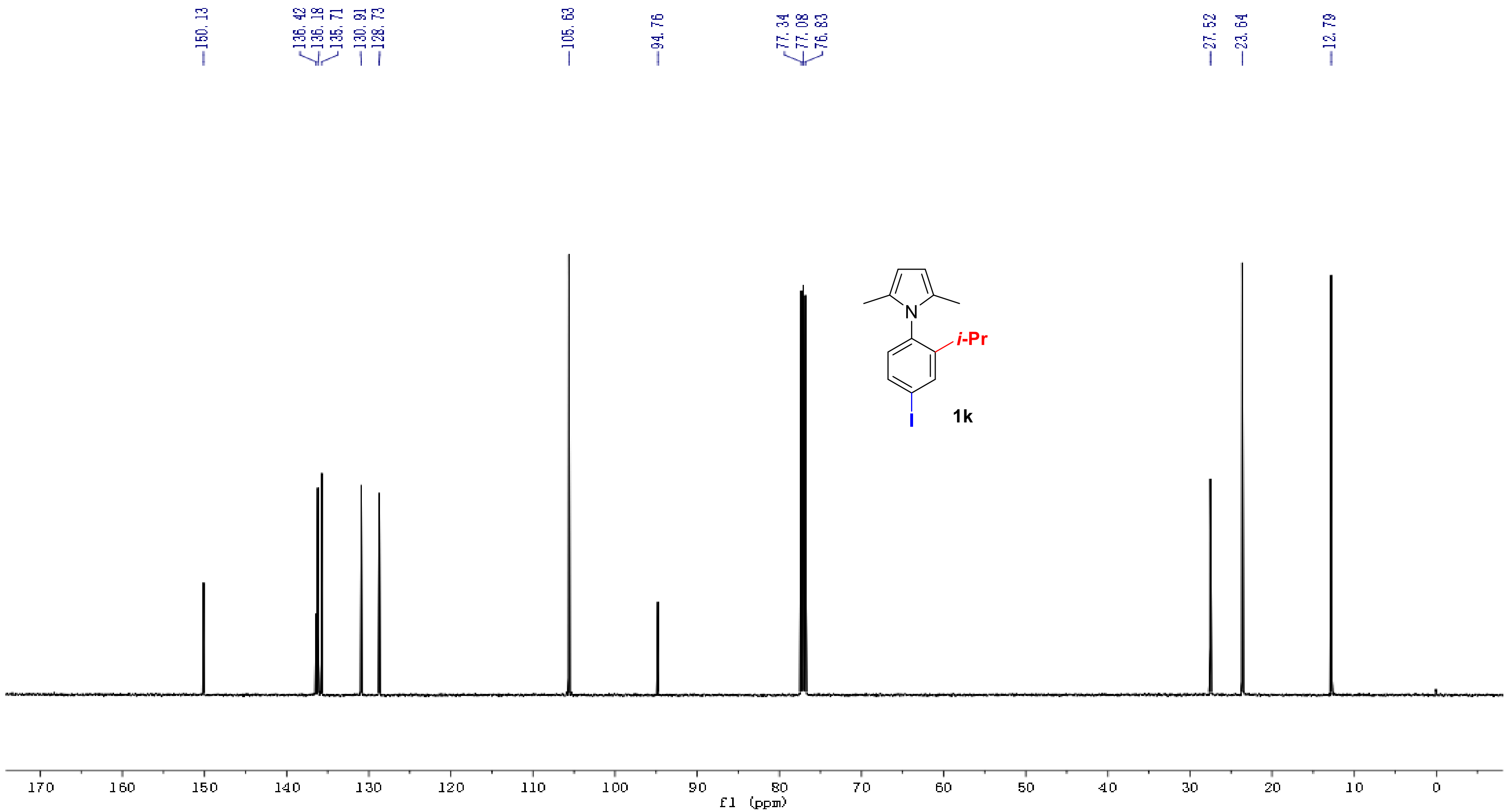


**Supplementary Figure 30.** $^{13}$C NMR of **1k**.

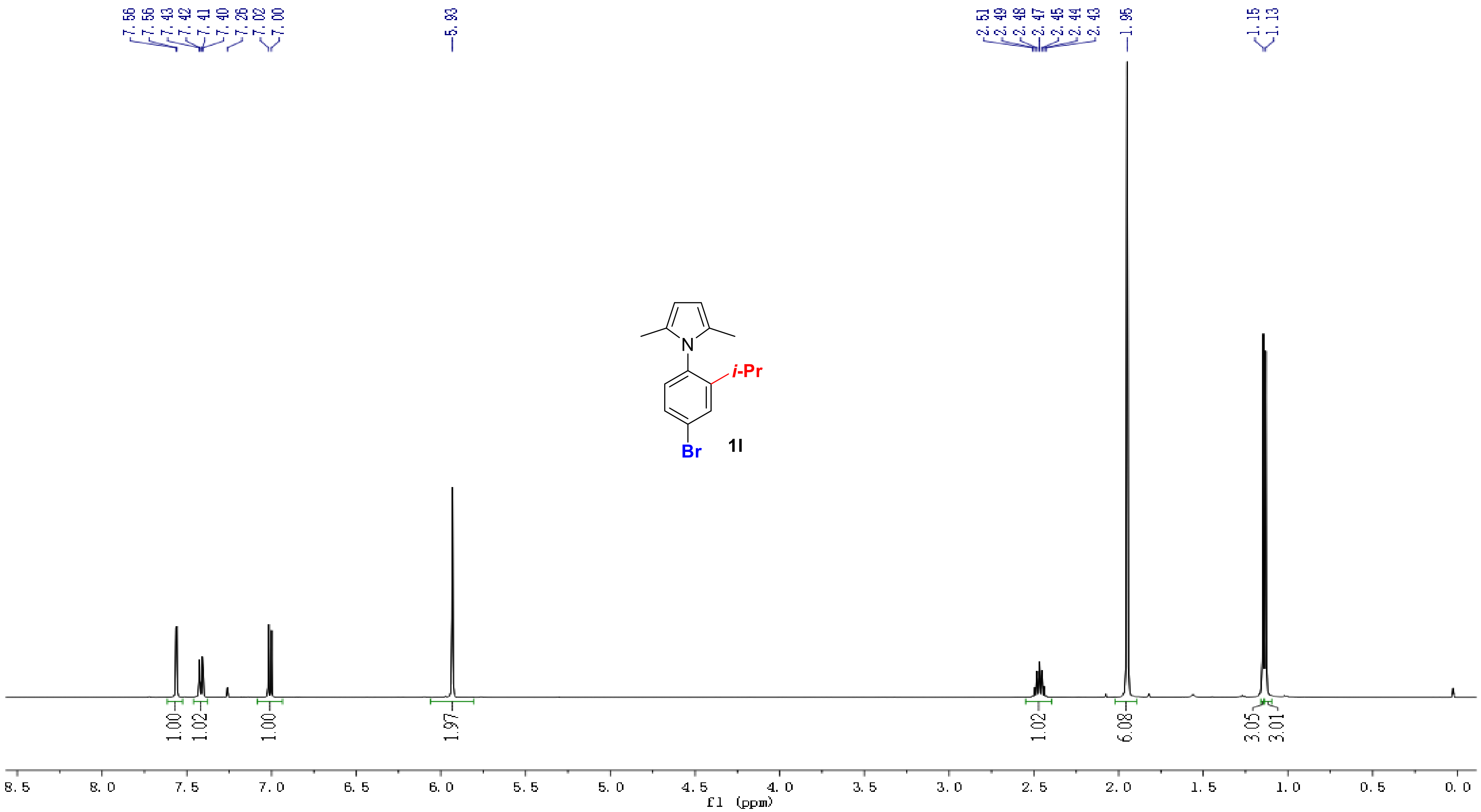


**Supplementary Figure 31.** $^{1}$H NMR of **1l**.

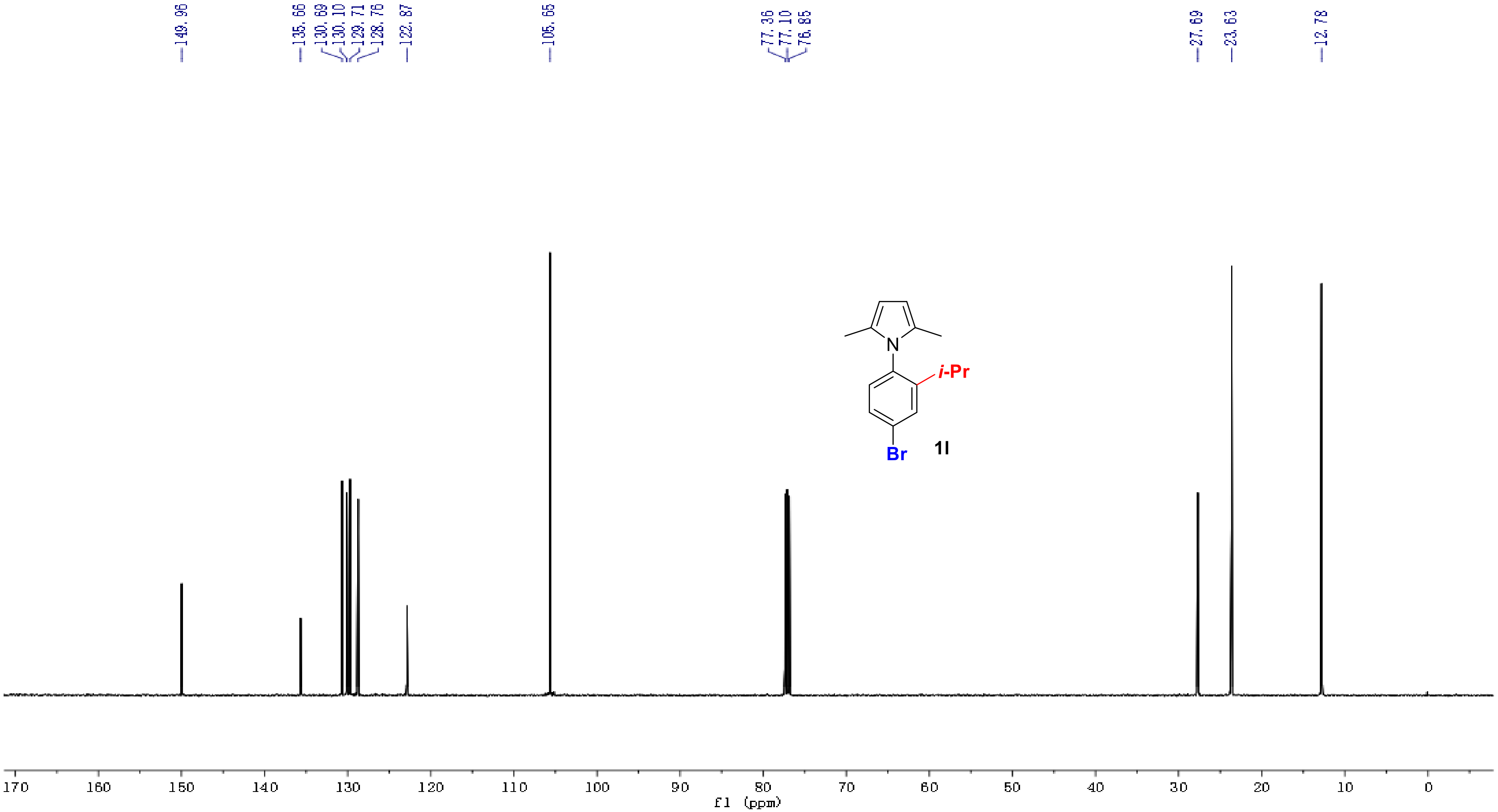


**Supplementary Figure 32.** $^{13}$C NMR of **1l**.

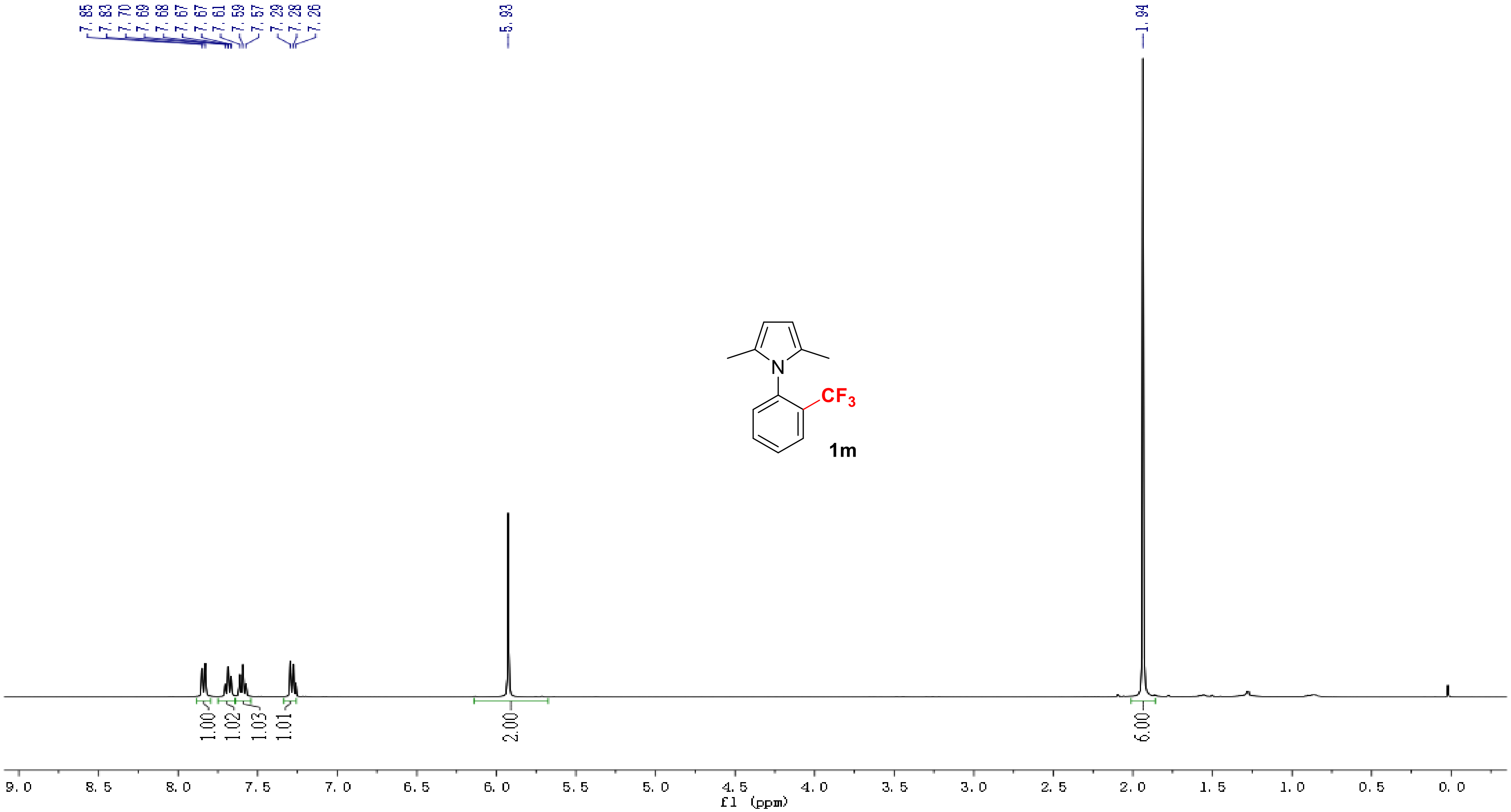


**Supplementary Figure 33.** $^1$H NMR of **1m**.

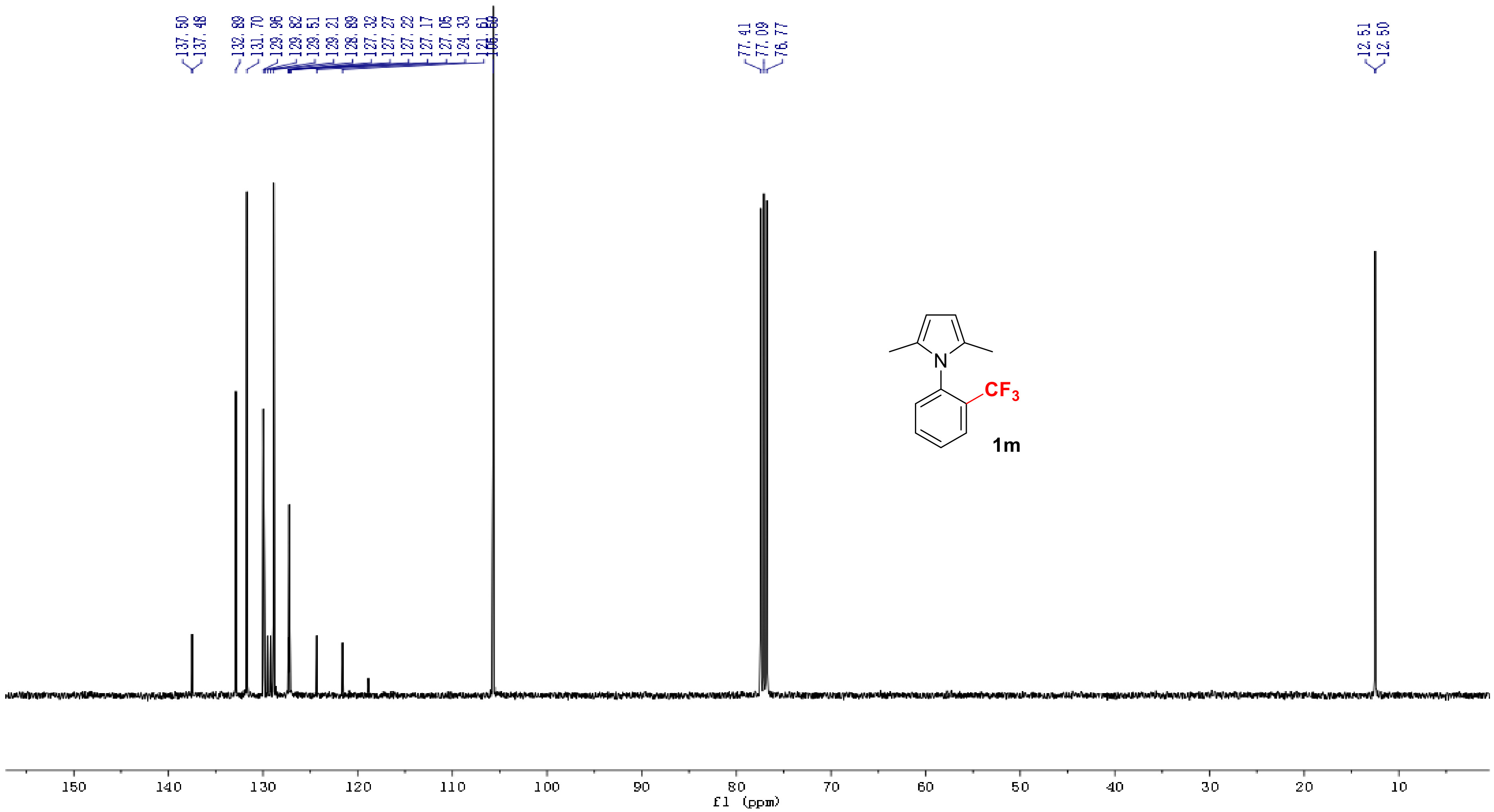


**Supplementary Figure 34.** $^{13}$C NMR of **1m**.

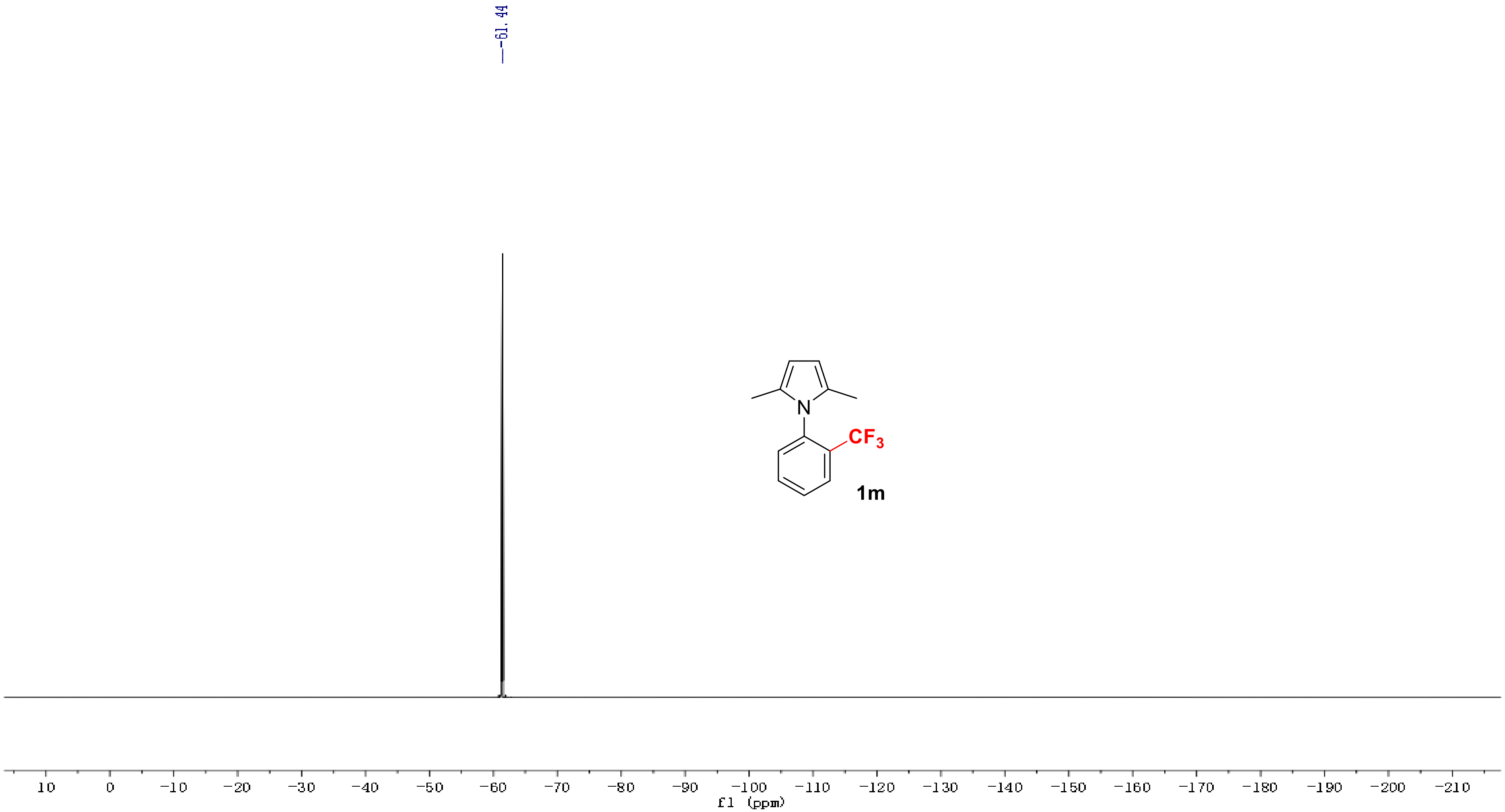


**Supplementary Figure 35.** $^{19}$F NMR of **1m**.

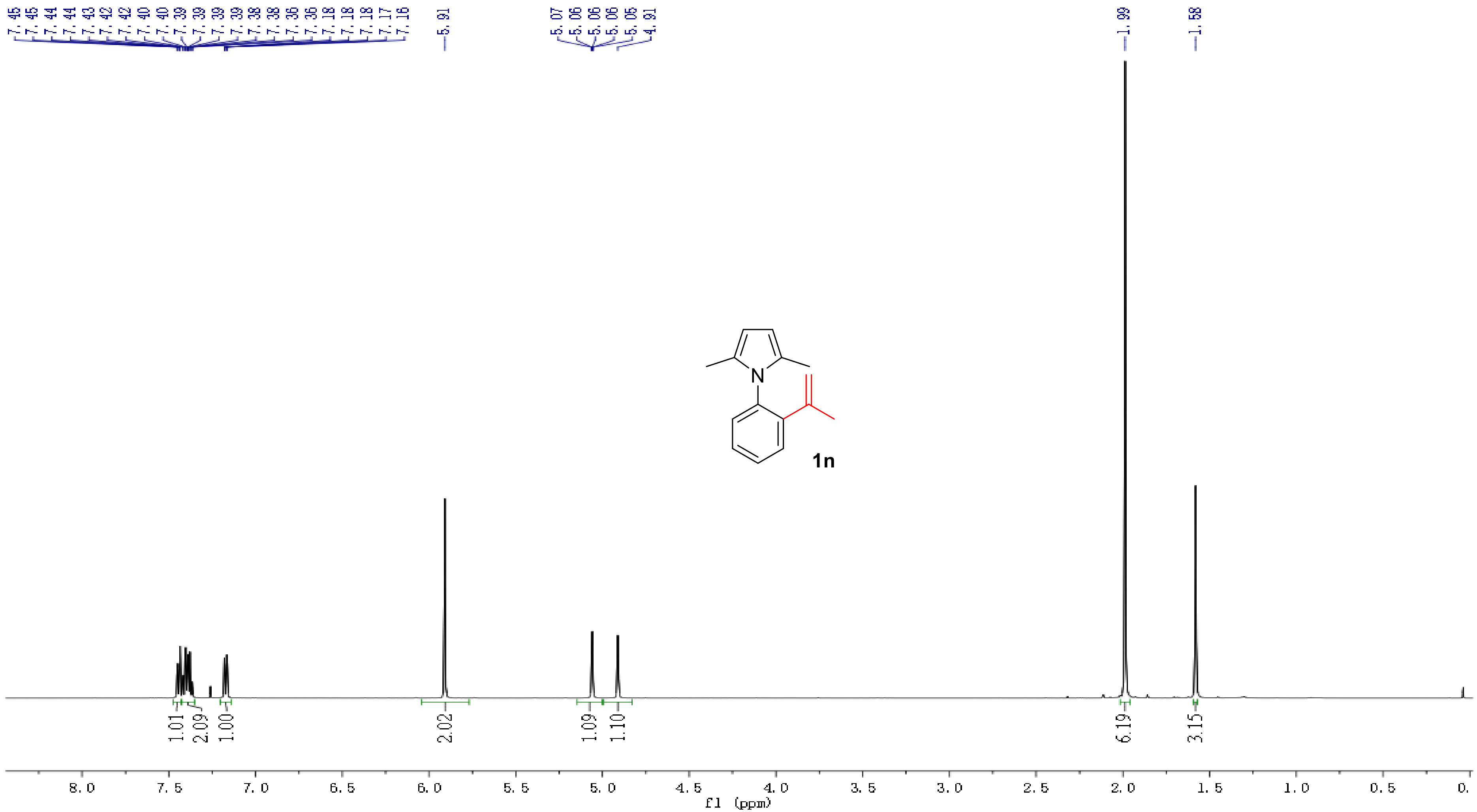


**Supplementary Figure 36.** $^{1}$H NMR of **1n**.

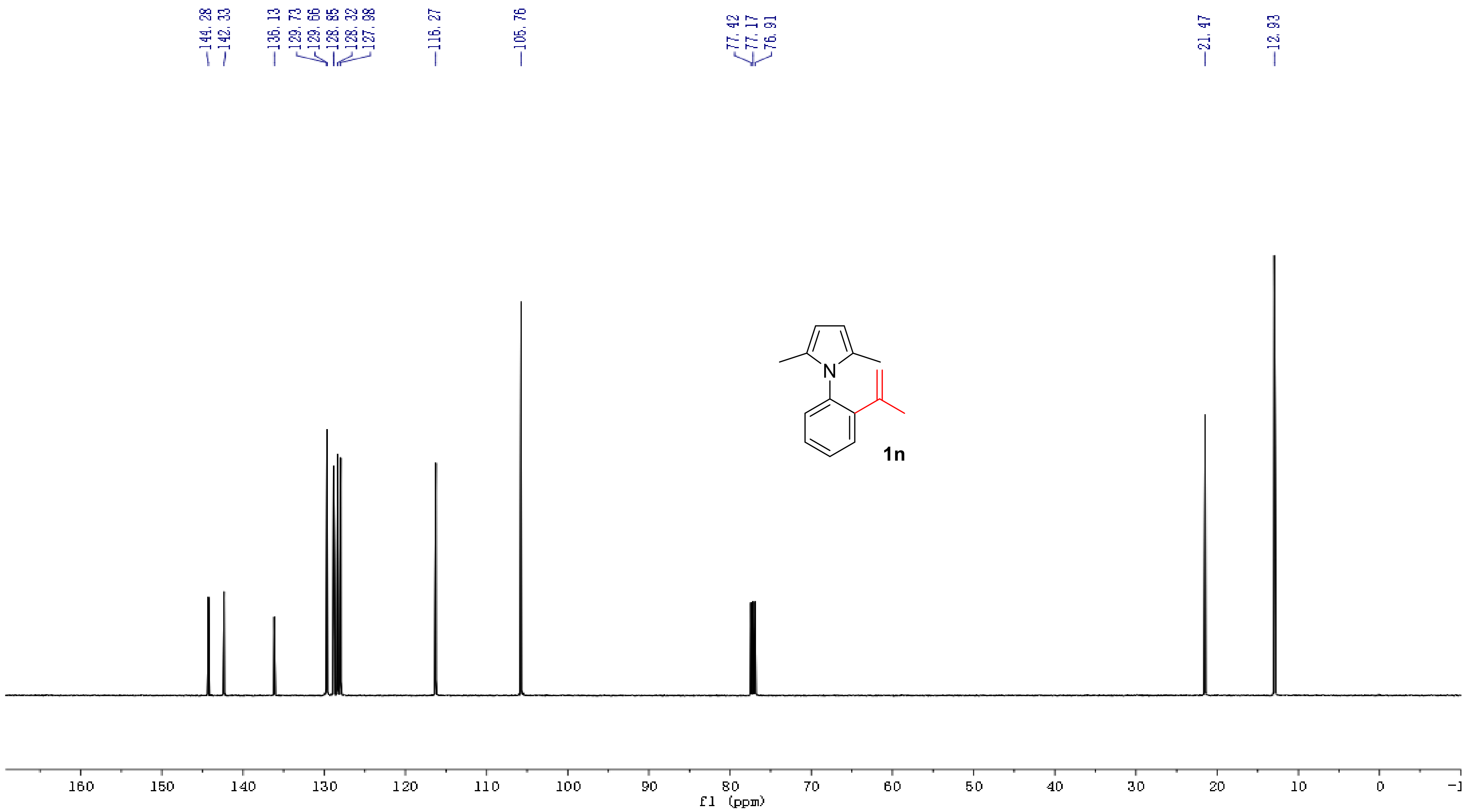


**Supplementary Figure 37.** $^{13}$C NMR of **1n**.

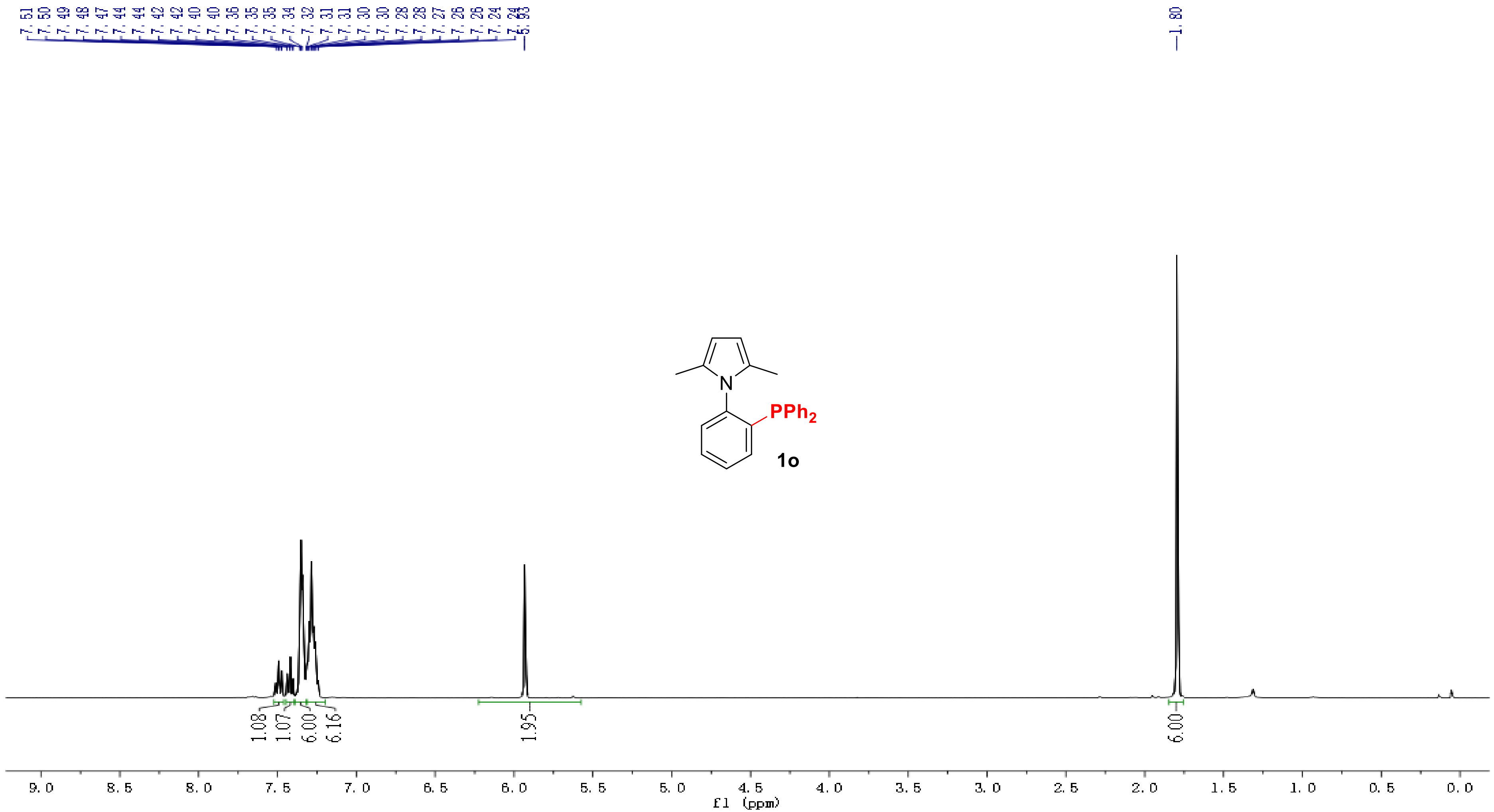


**Supplementary Figure 38.** $^{1}$H NMR of **1o**.

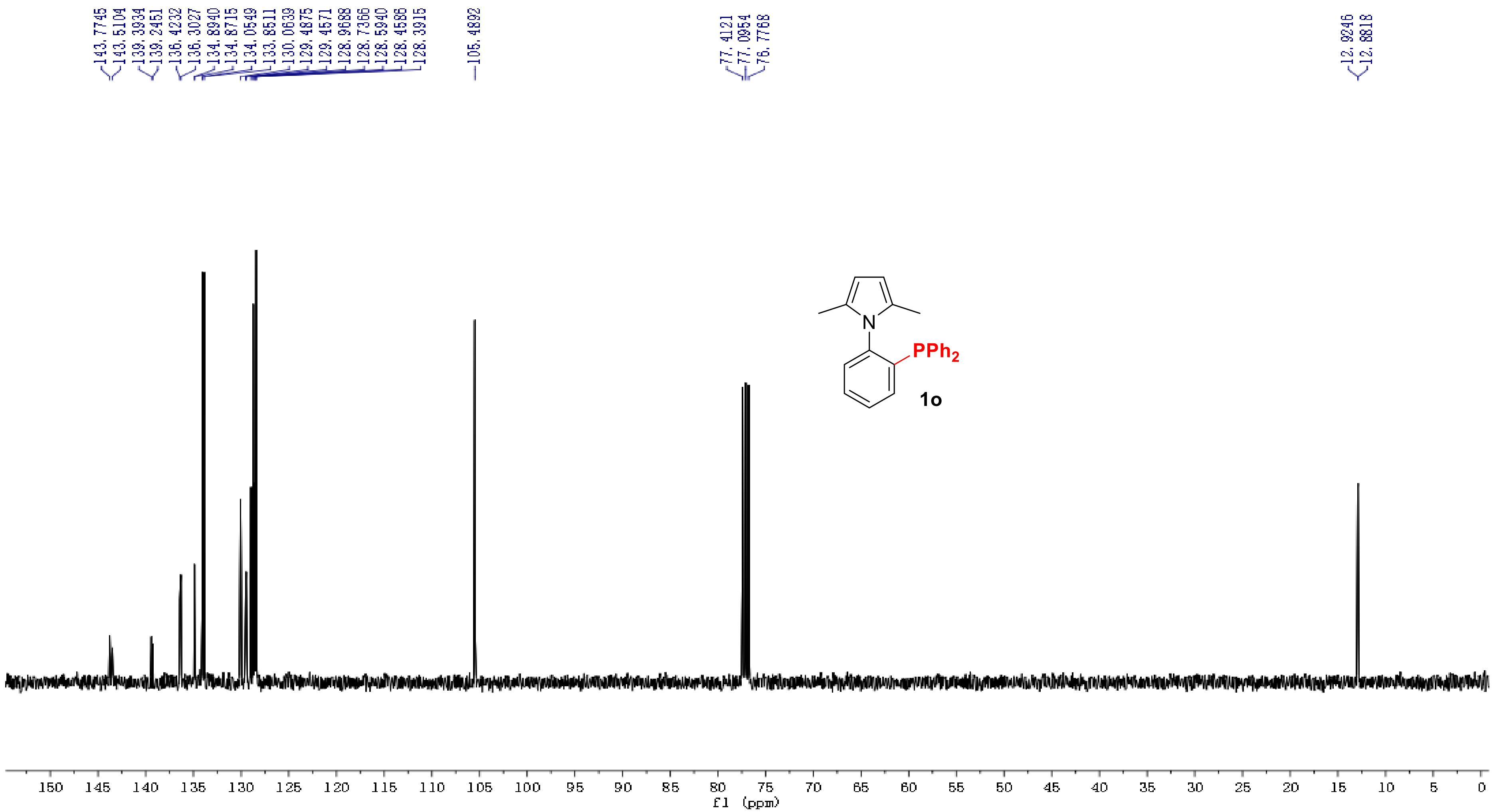


**Supplementary Figure 39.** $^{13}$C NMR of **1o**.

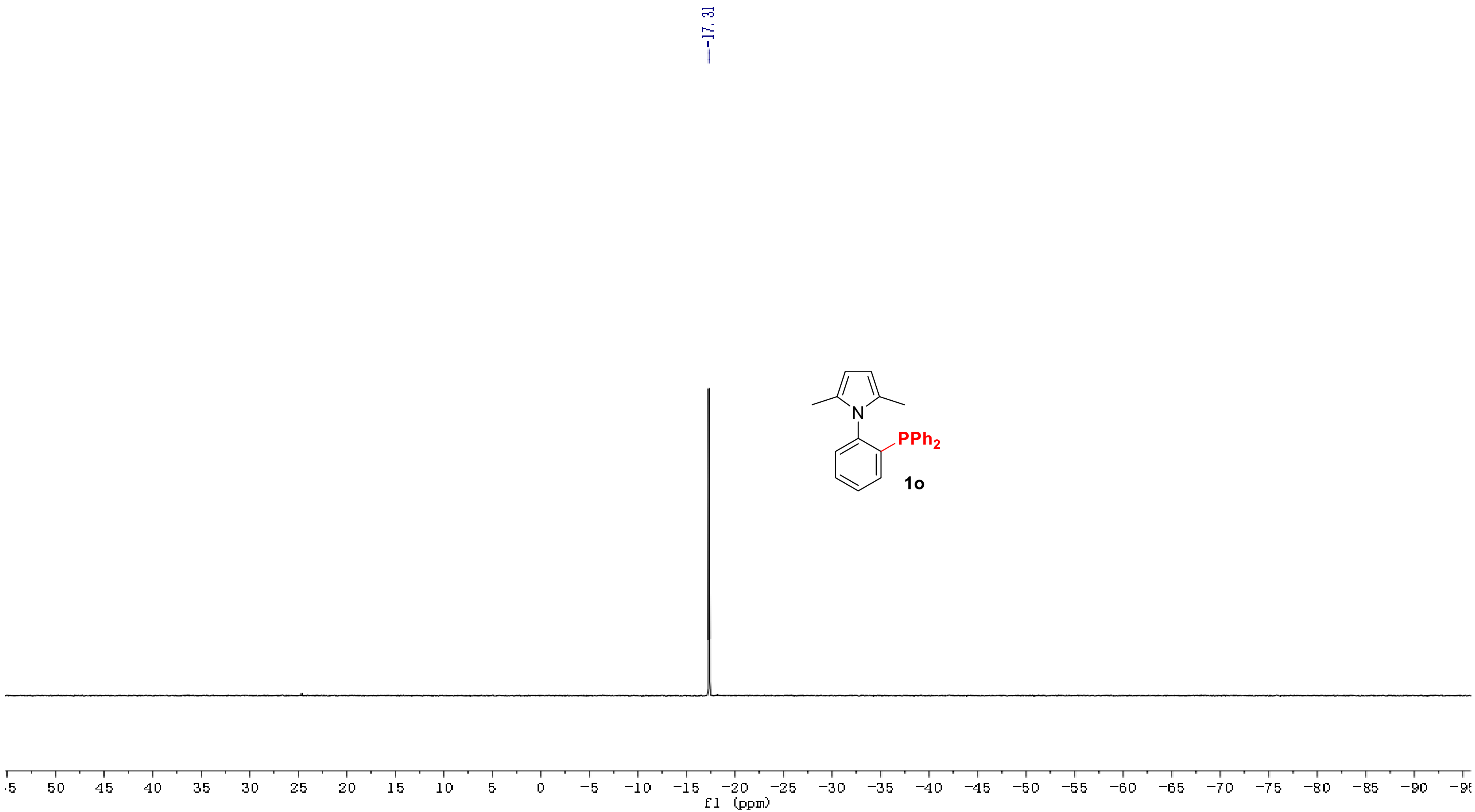


**Supplementary Figure 40.** $^{31}$P NMR of **1o**.

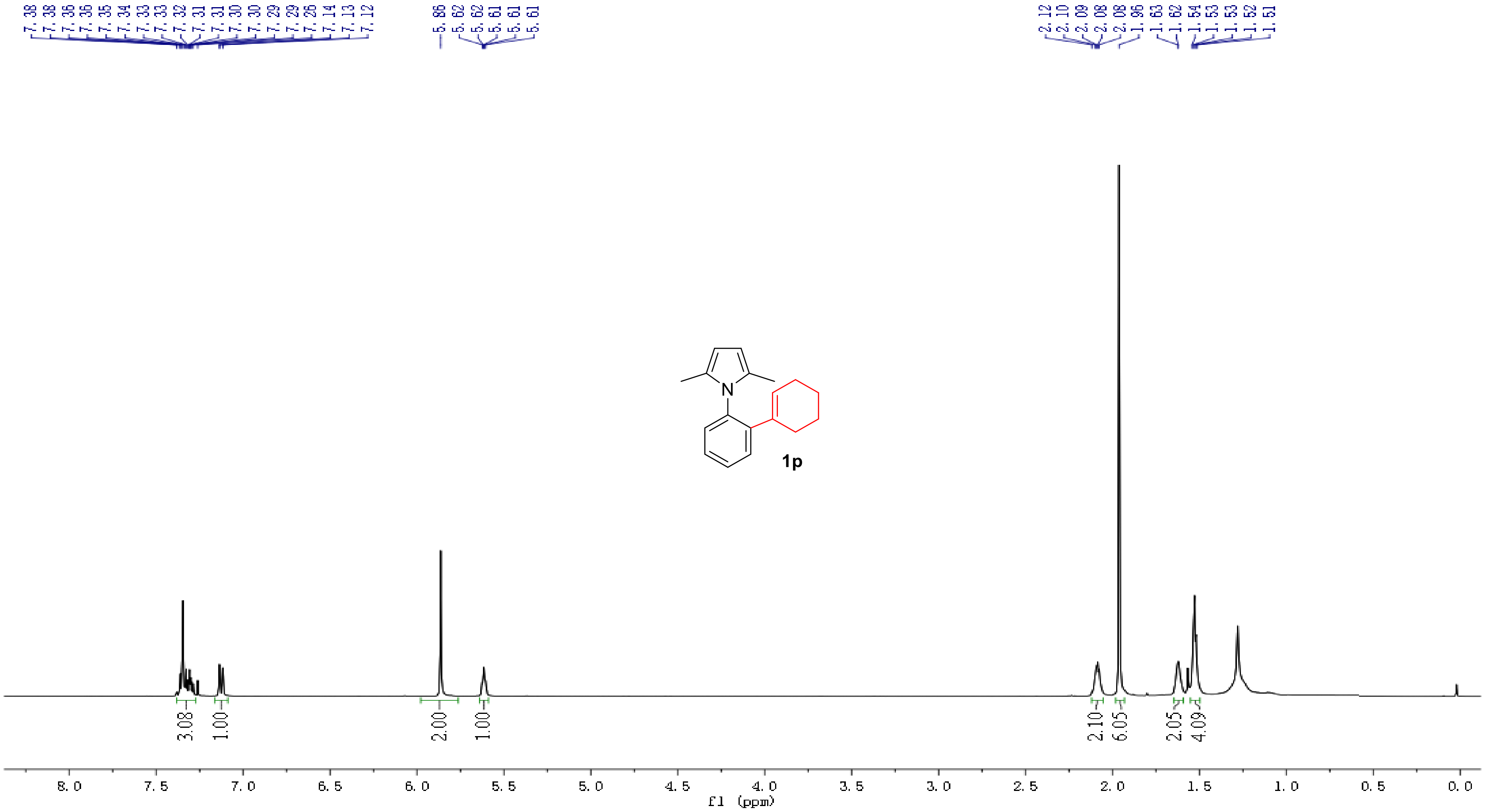


**Supplementary Figure 41.** $^{1}$H NMR of **1p**.

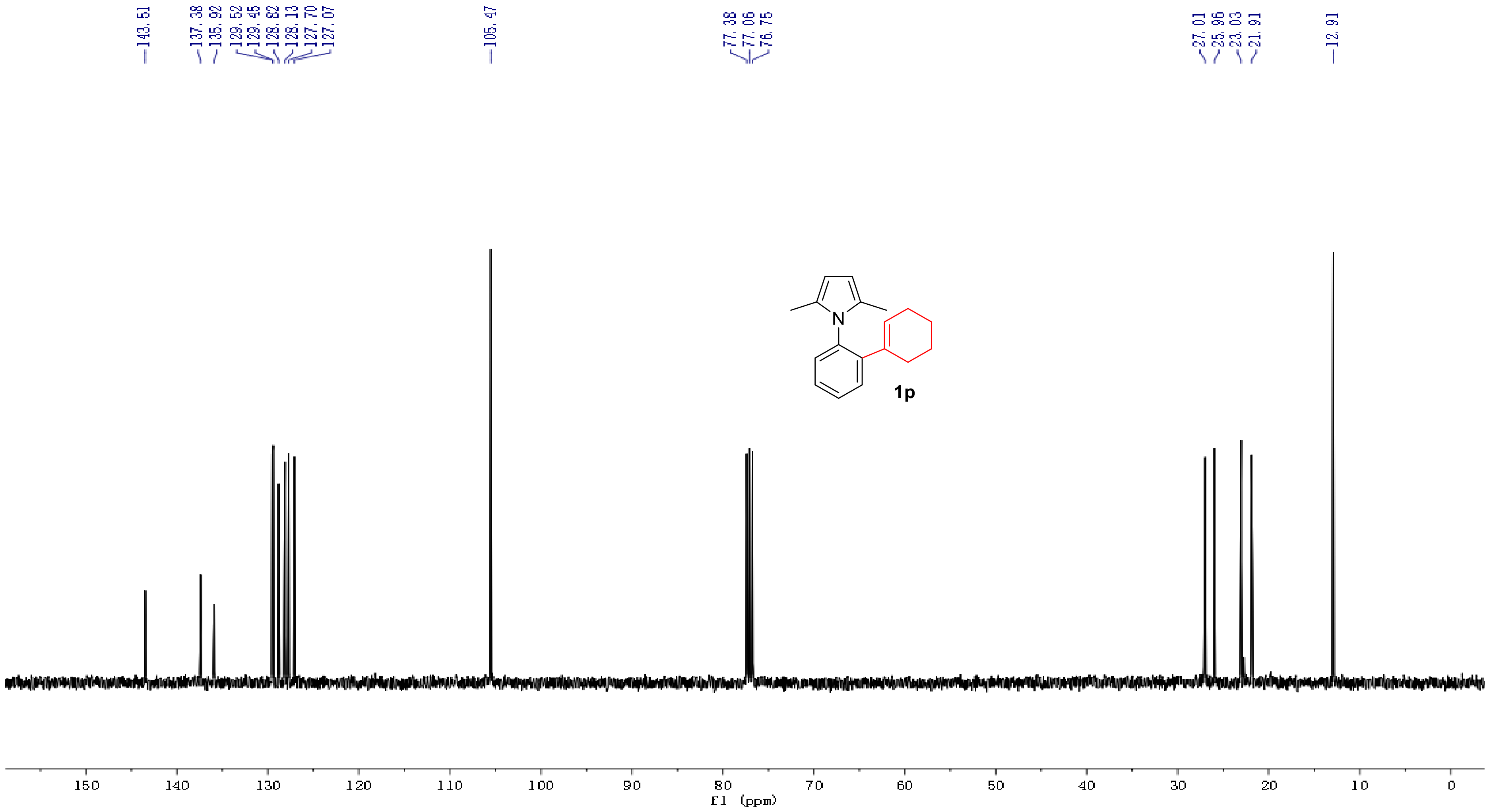


**Supplementary Figure 42.** $^{13}$C NMR of **1p**.

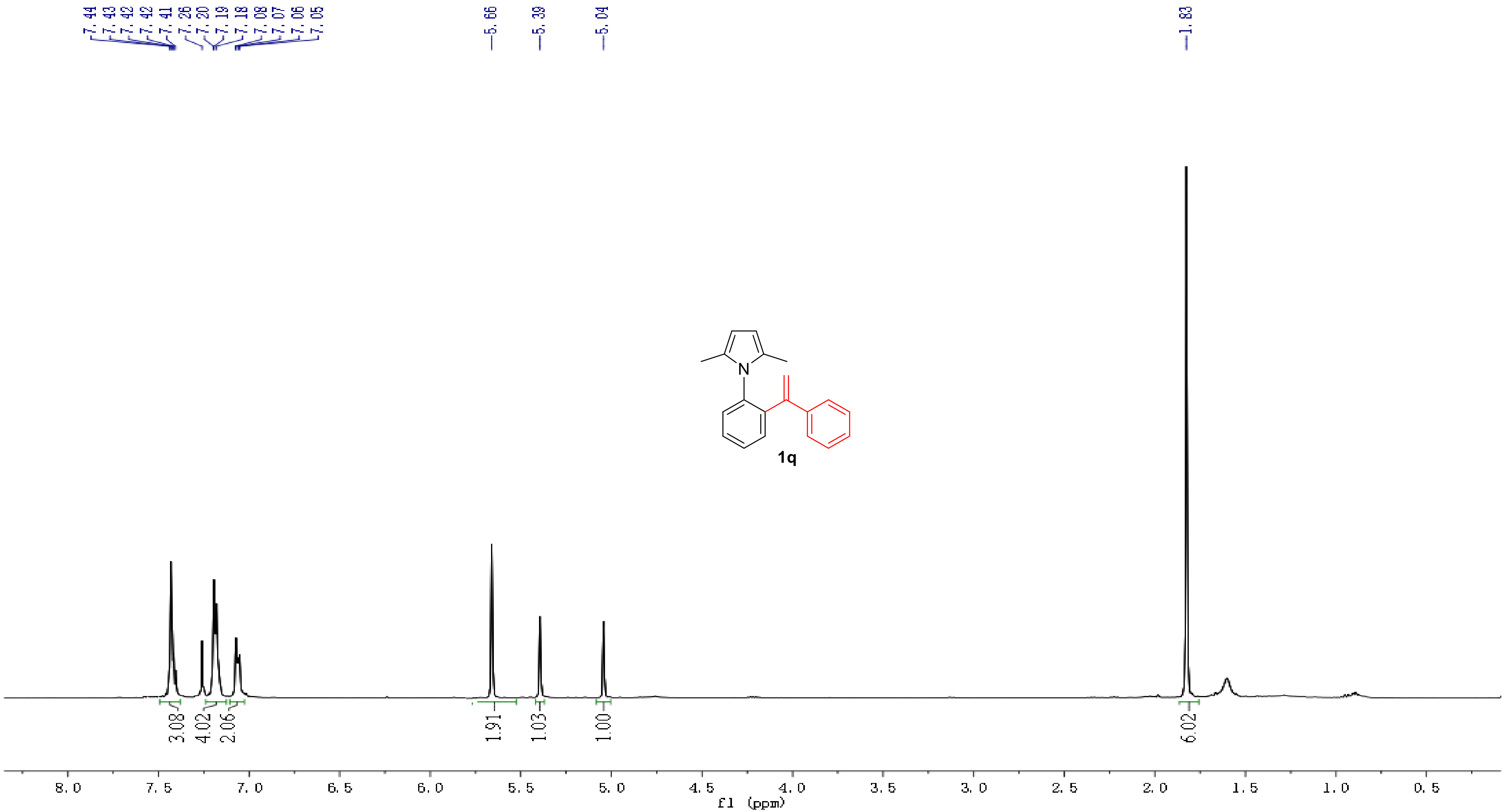


**Supplementary Figure 43.** $^1$H NMR of **1q**.

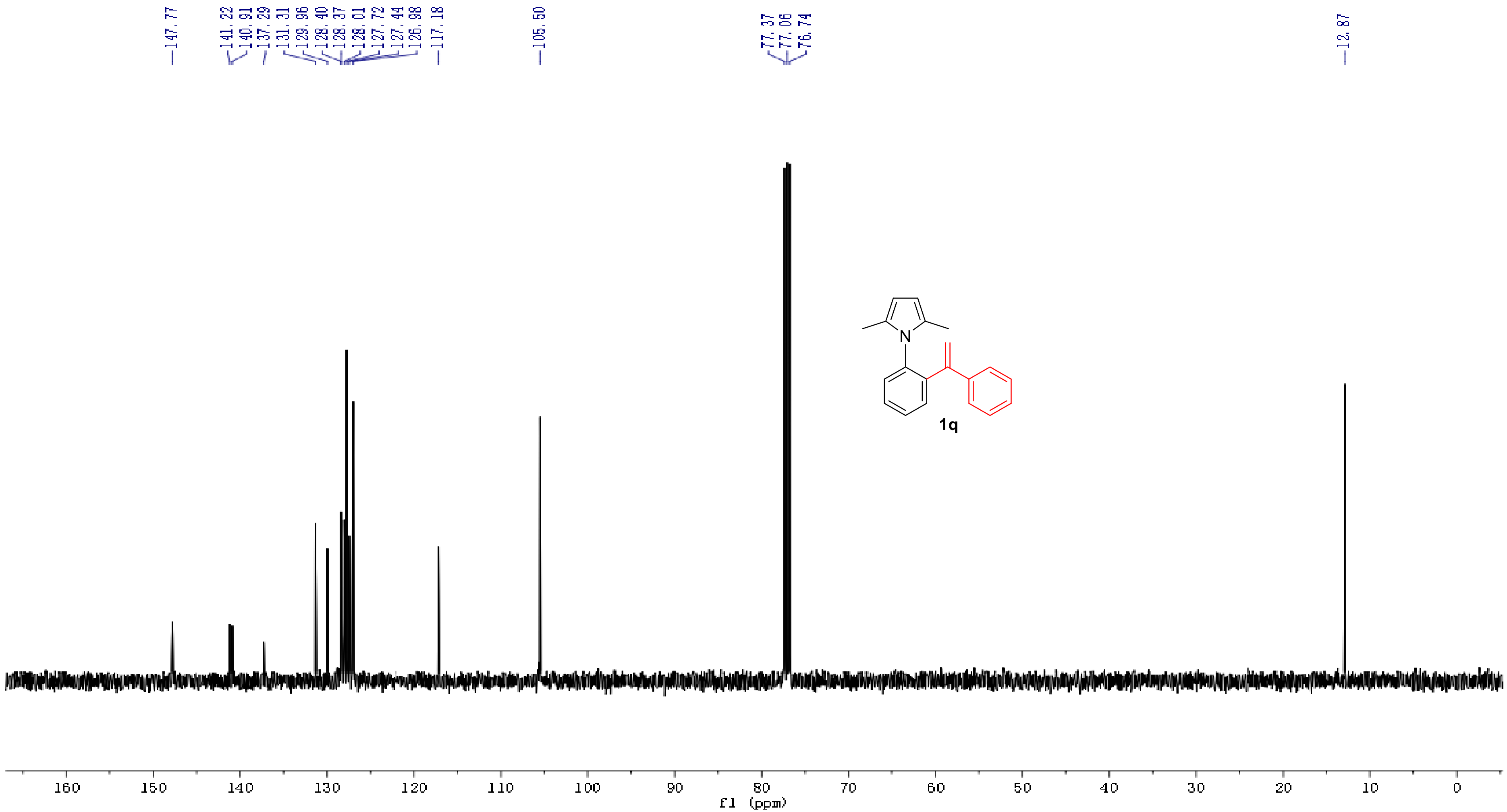


**Supplementary Figure 44.** $^{13}$C NMR of **1q**.

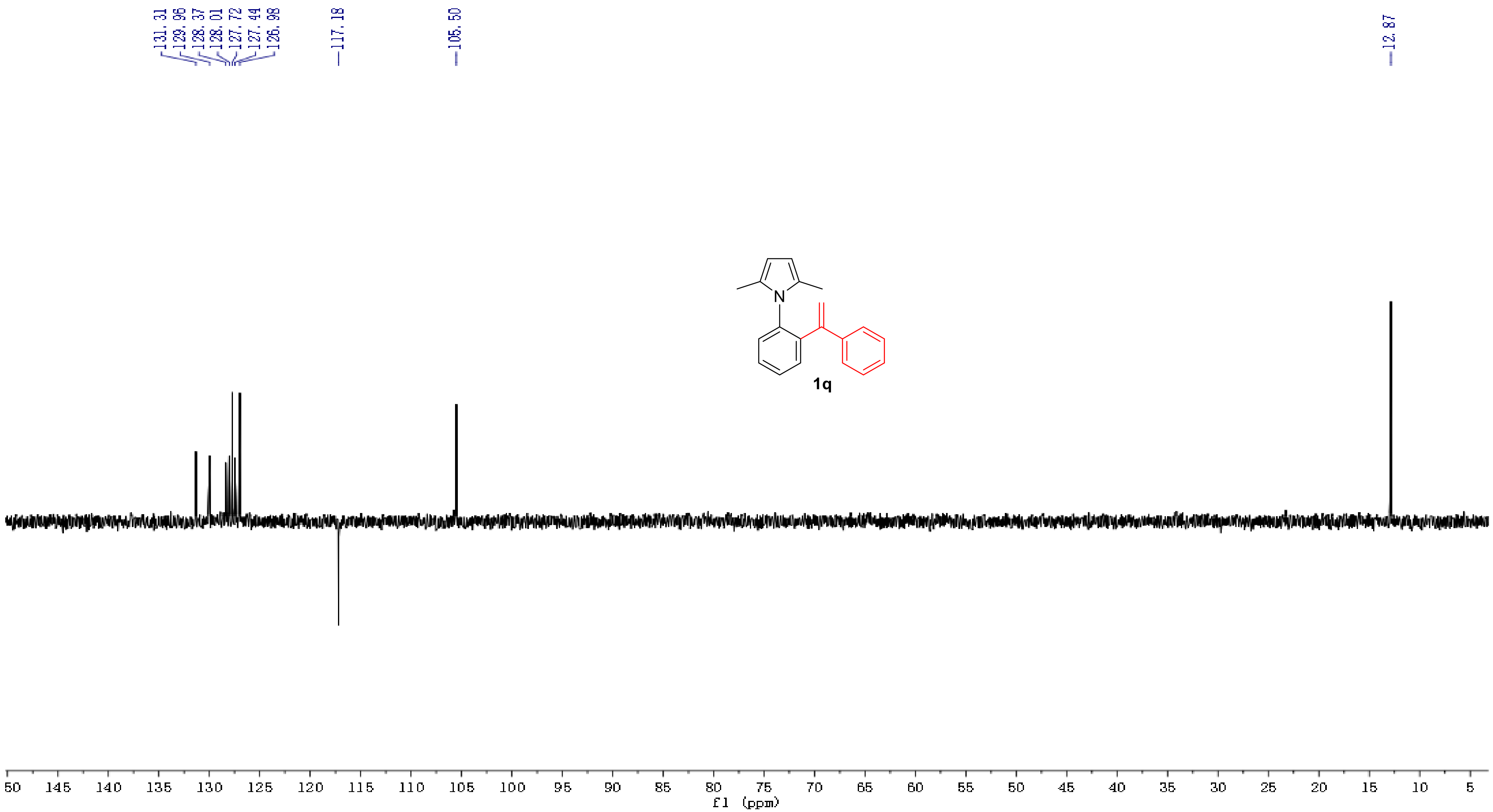


**Supplementary Figure 45.** $^{13}$C NMR-DEPT 135 of **1q**.

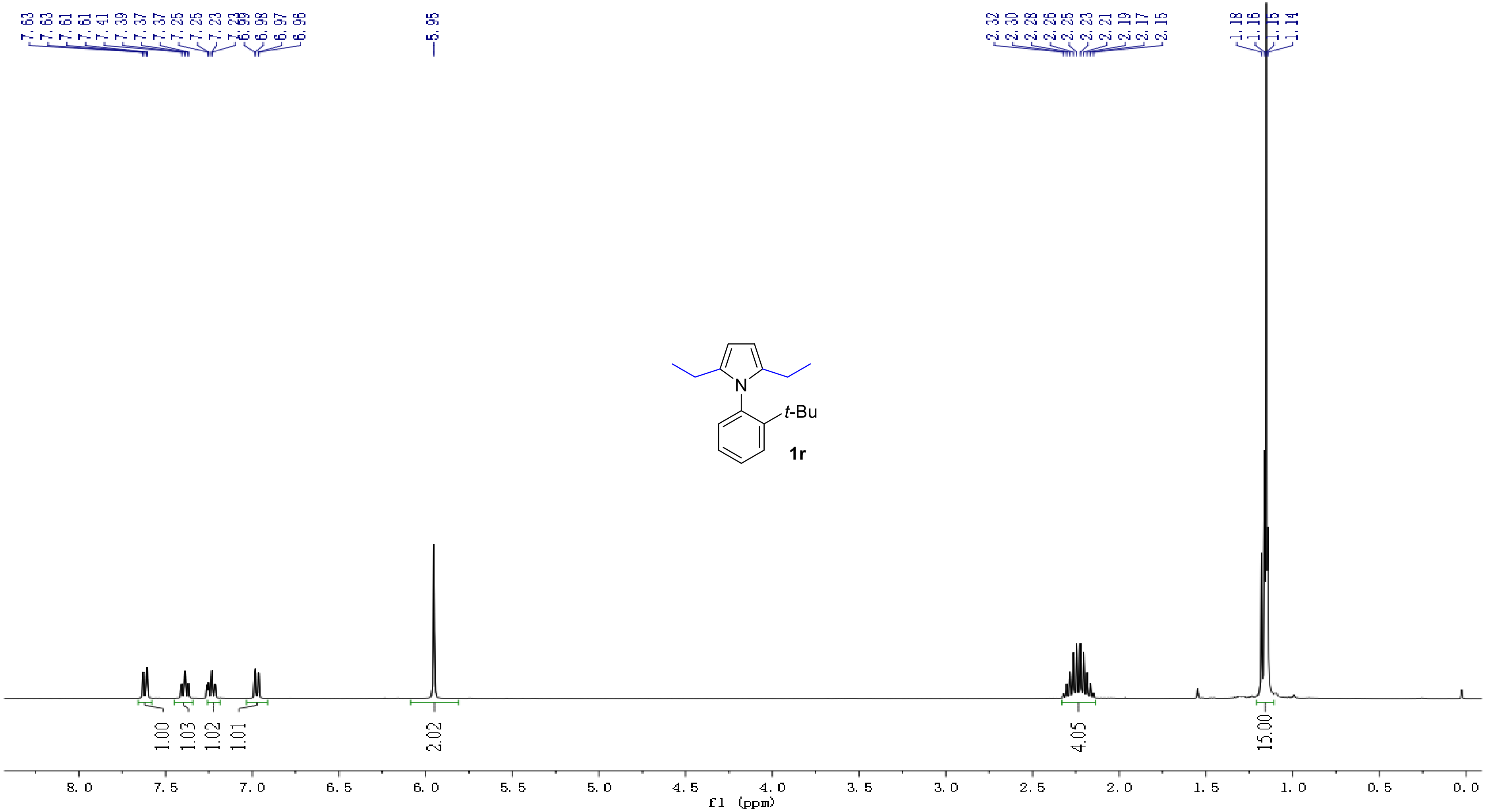


**Supplementary Figure 46.** $^1$H NMR of **1r**.

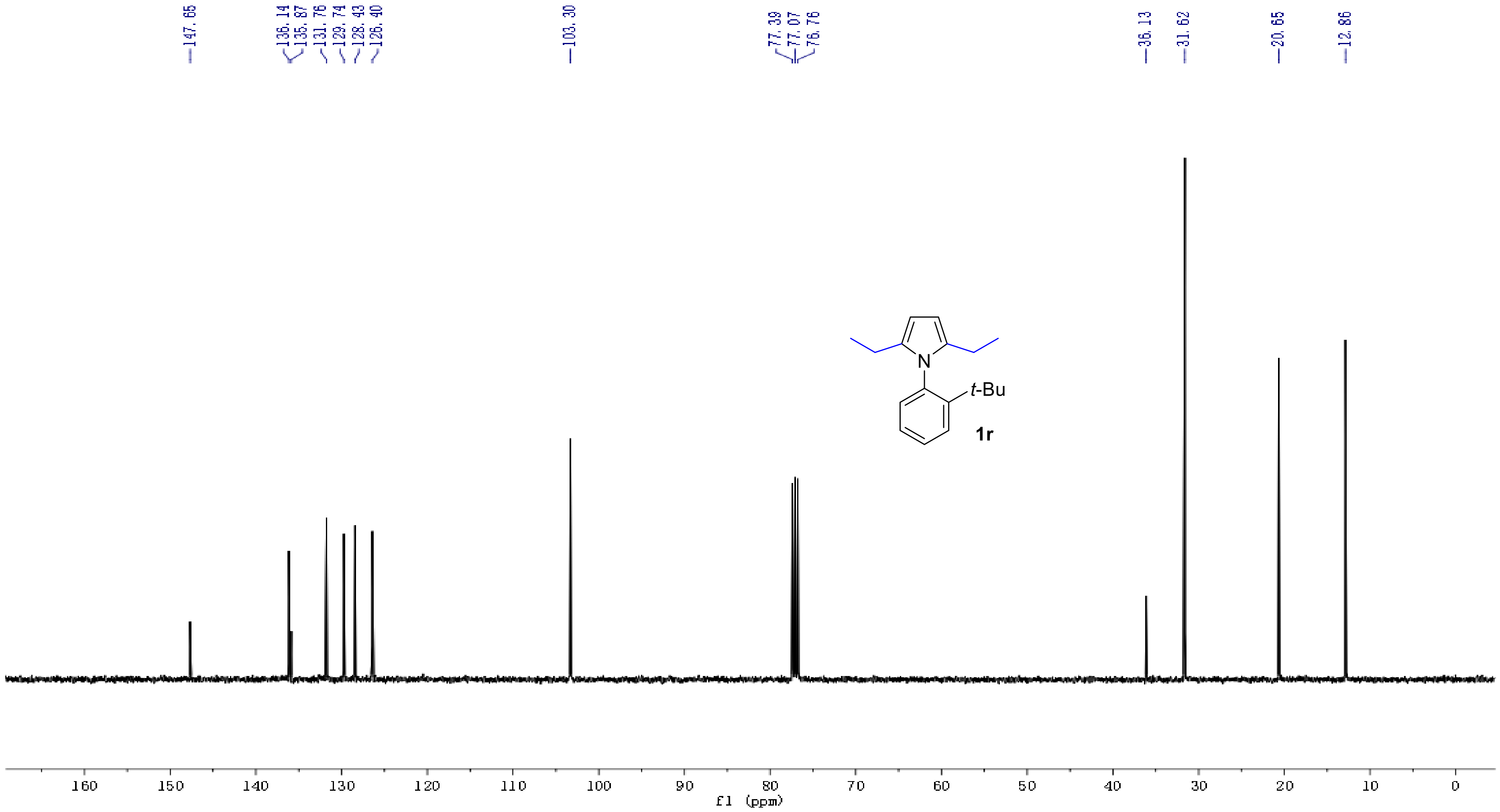


**Supplementary Figure 47.** $^{13}$C NMR of **1r**.

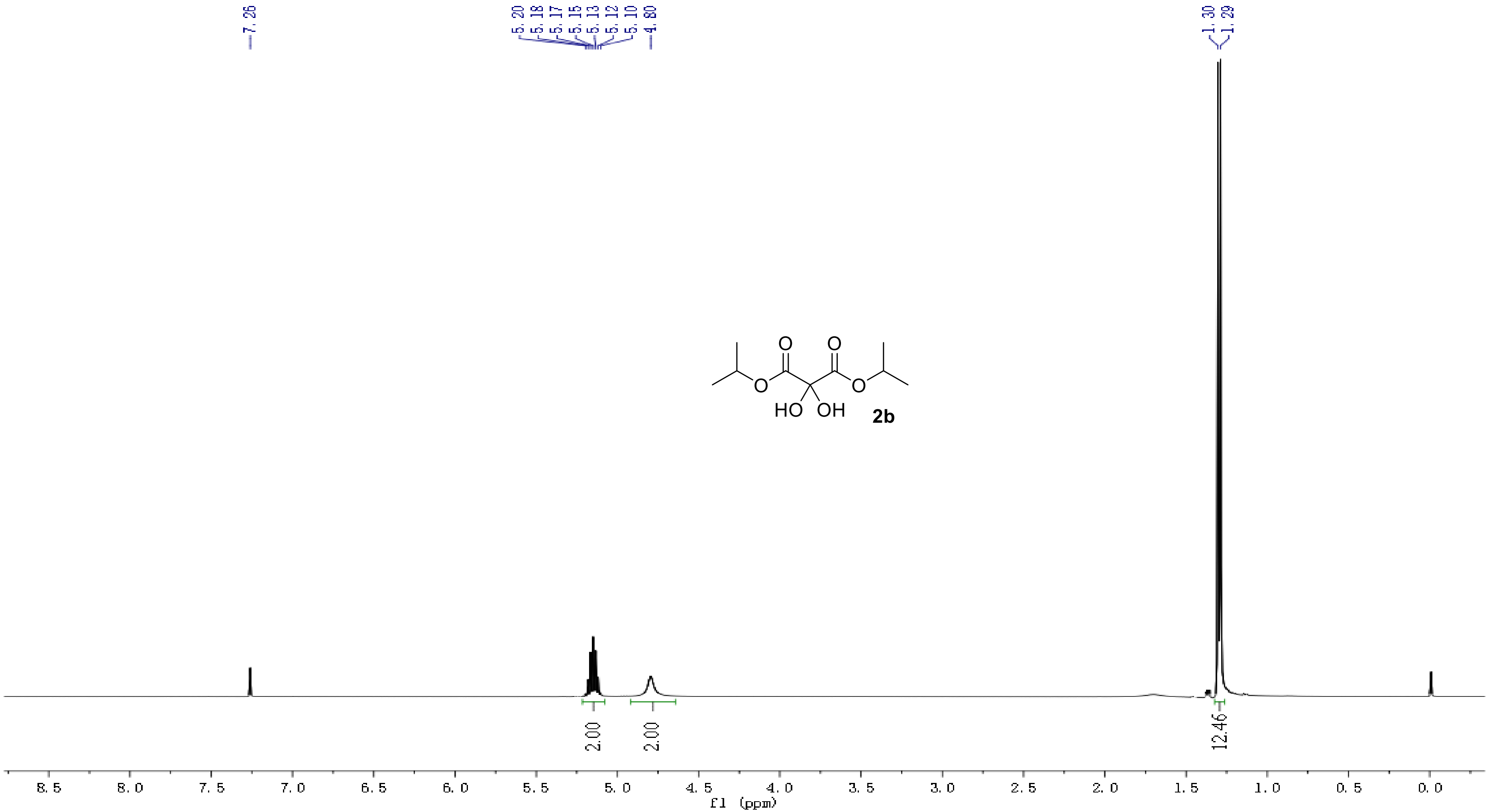


**Supplementary Figure 48.** $^{1}$H NMR of **2b**.

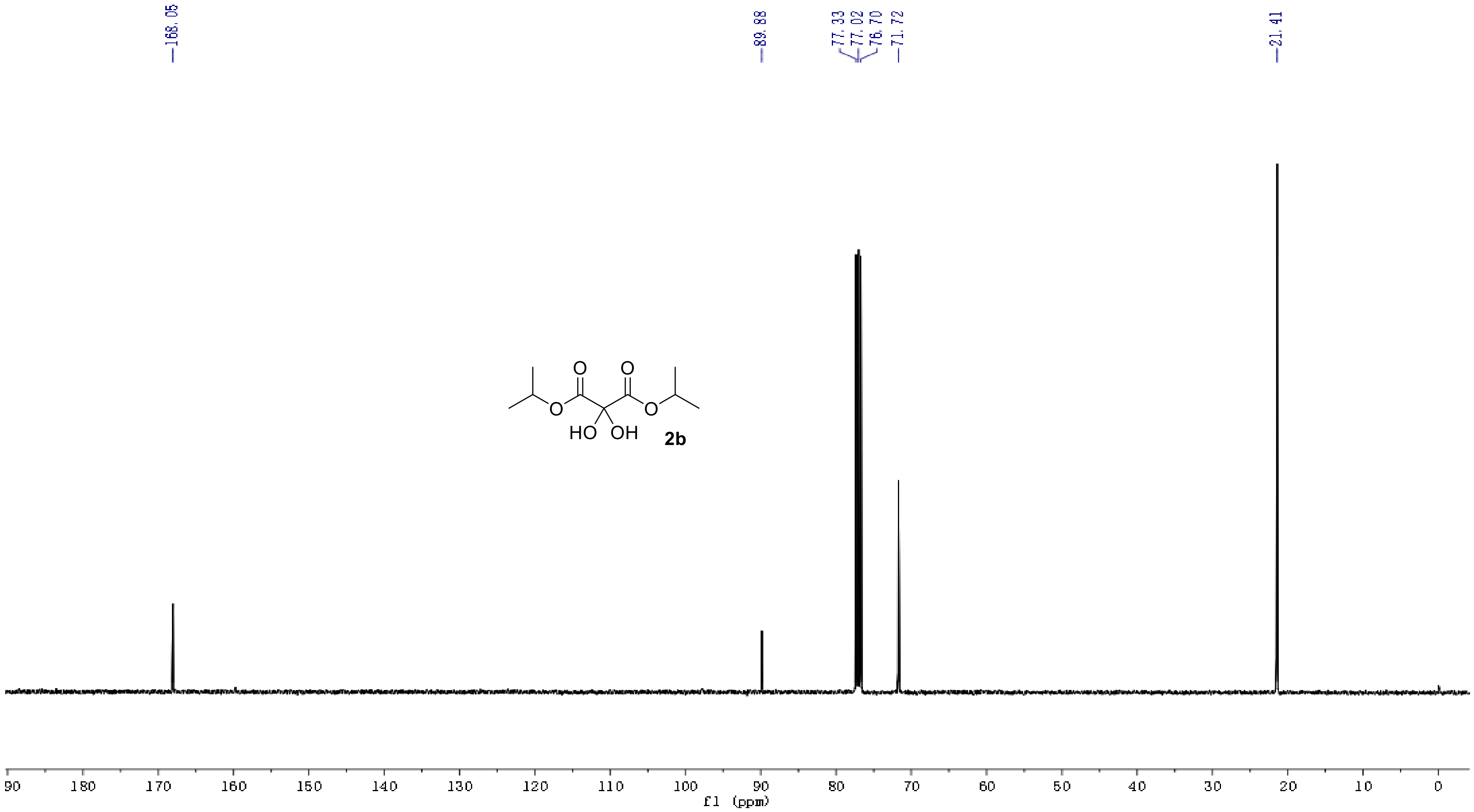


**Supplementary Figure 49.** $^{13}$C NMR of **2b**.

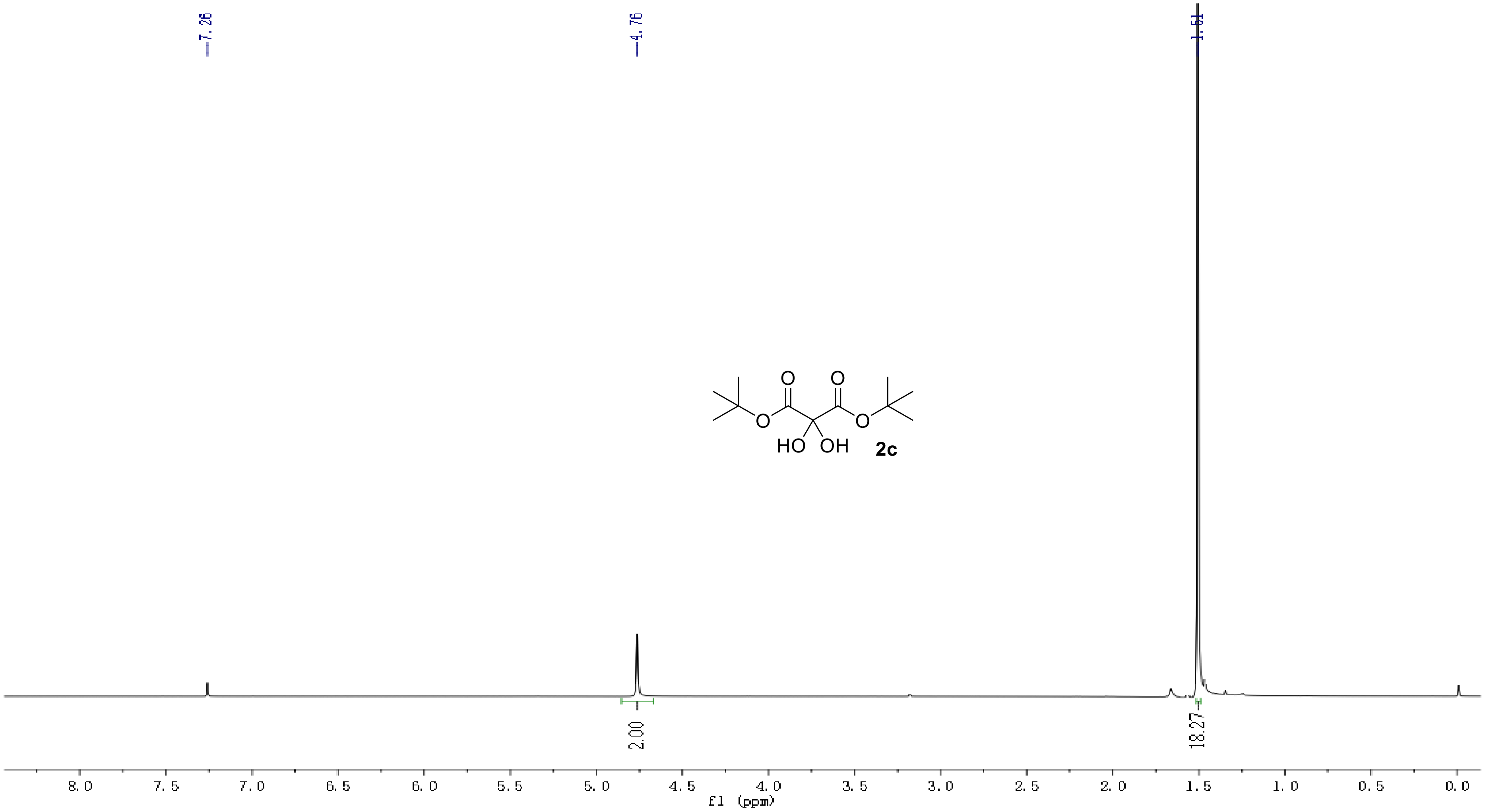


**Supplementary Figure 50.** $^{1}$H NMR of **2c**.

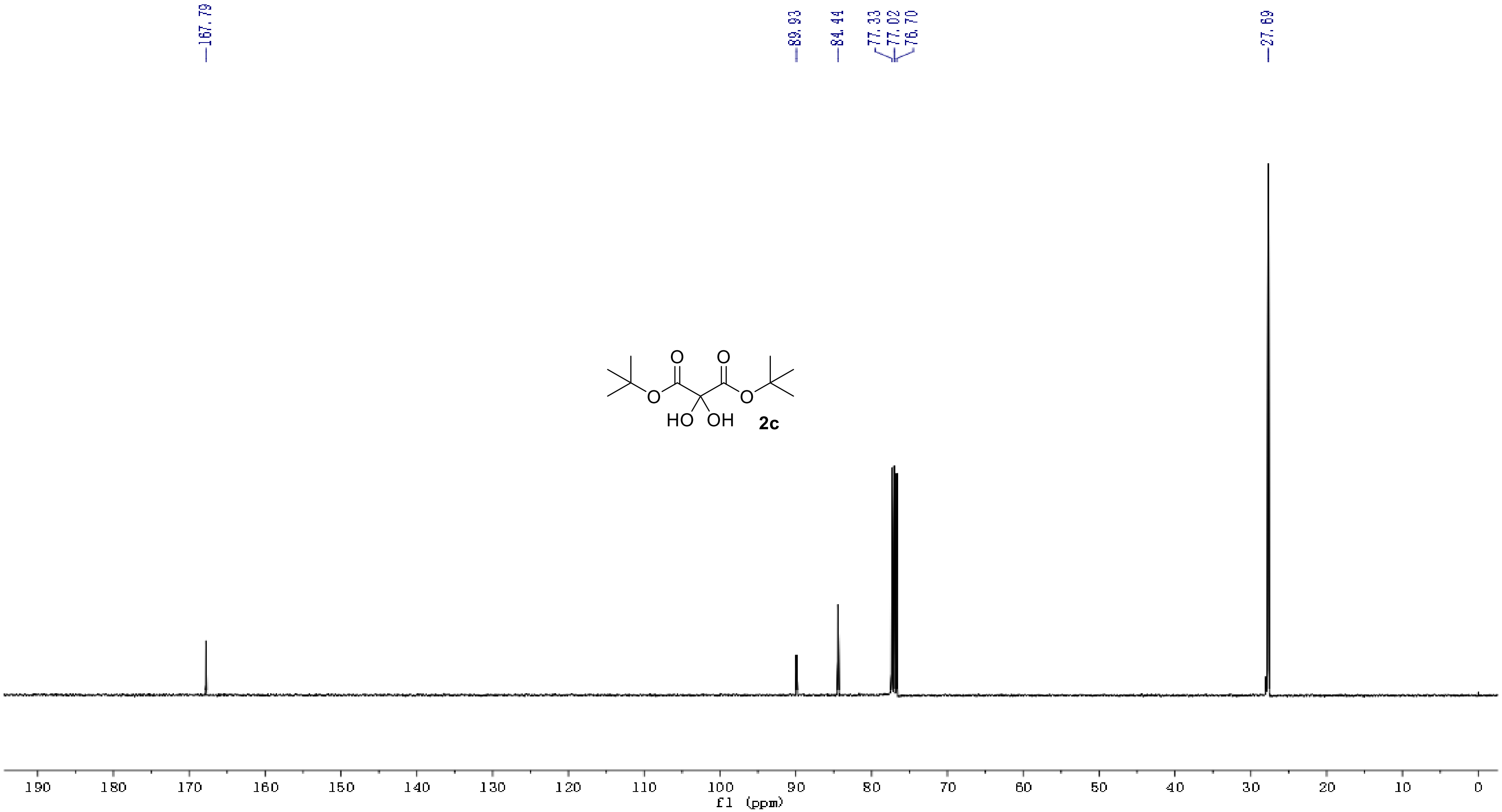


**Supplementary Figure 51.** $^{13}$C NMR of **2c**.

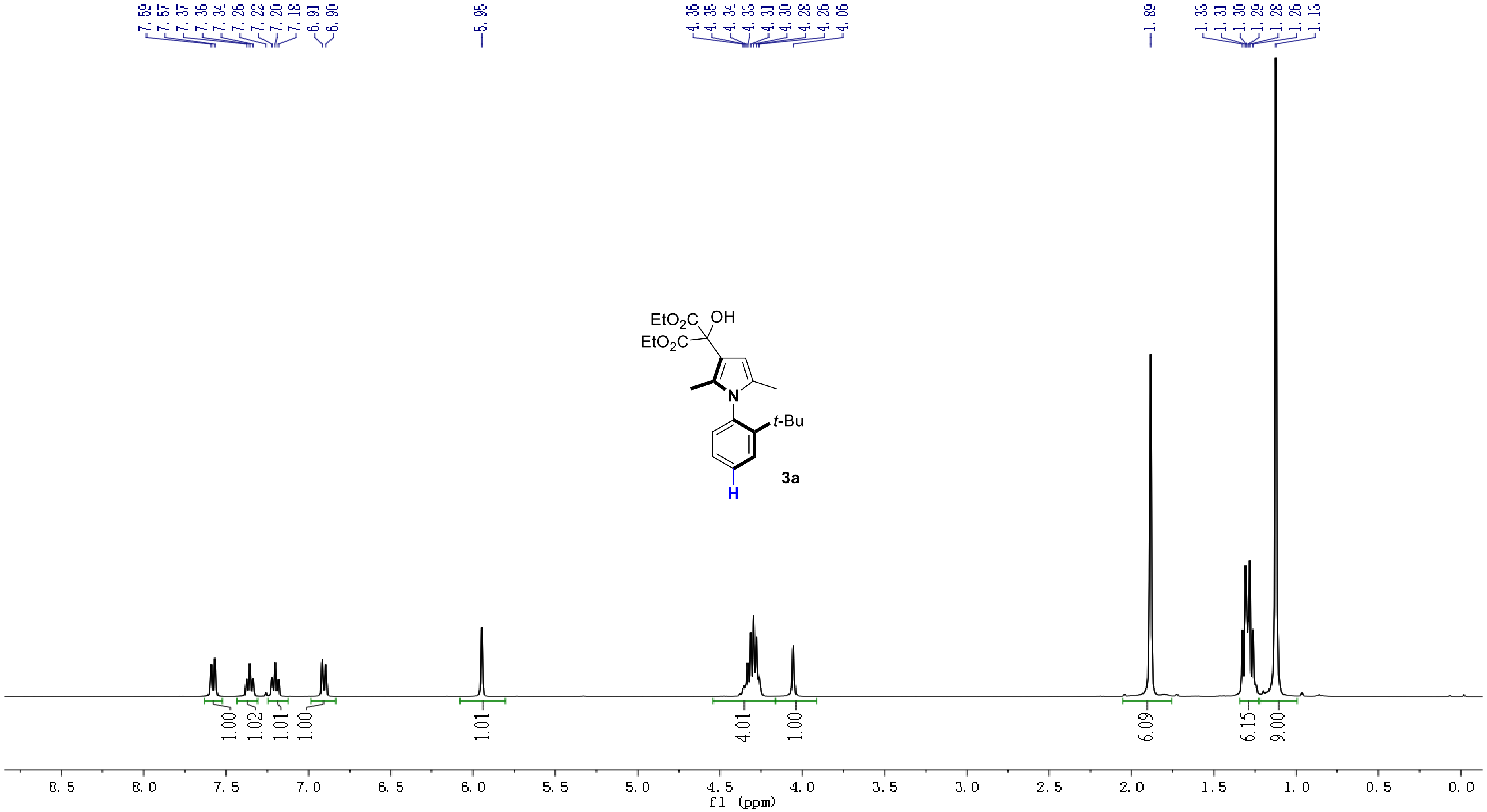


**Supplementary Figure 52.** $^{1}$H NMR of **3a**.

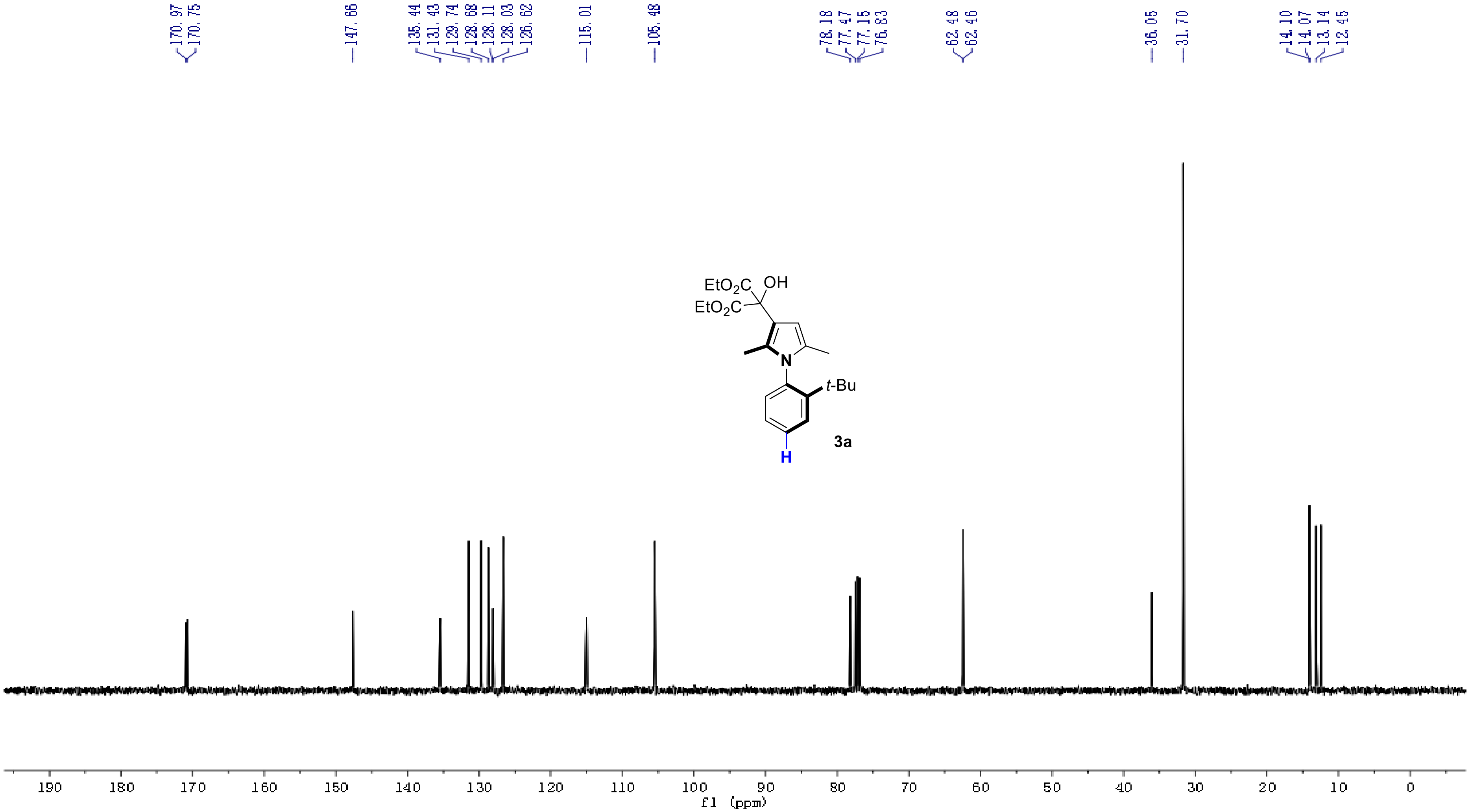


**Supplementary Figure 53.** $^{13}$C NMR of **3a**.

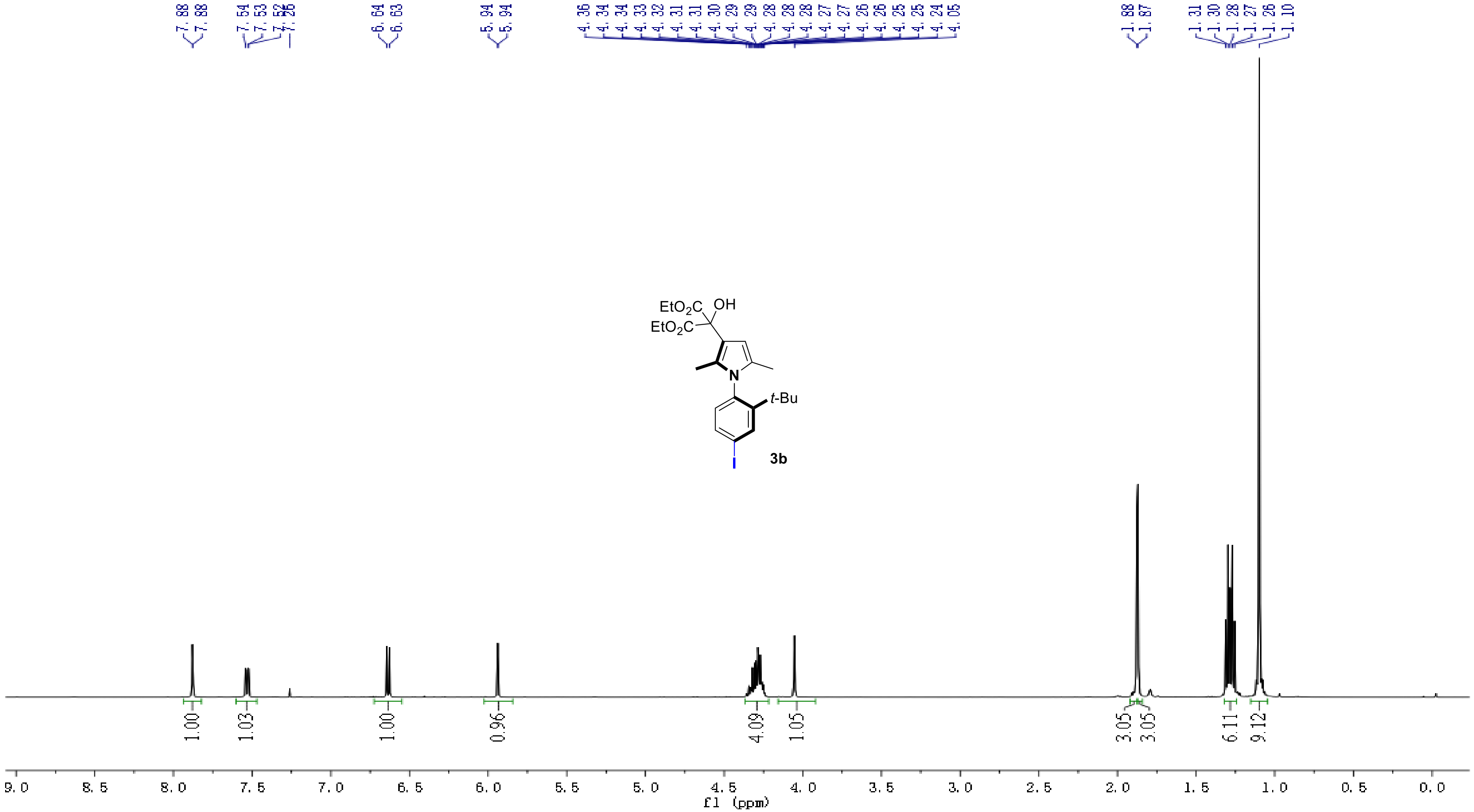


**Supplementary Figure 54.** $^{1}$H NMR of **3b**.

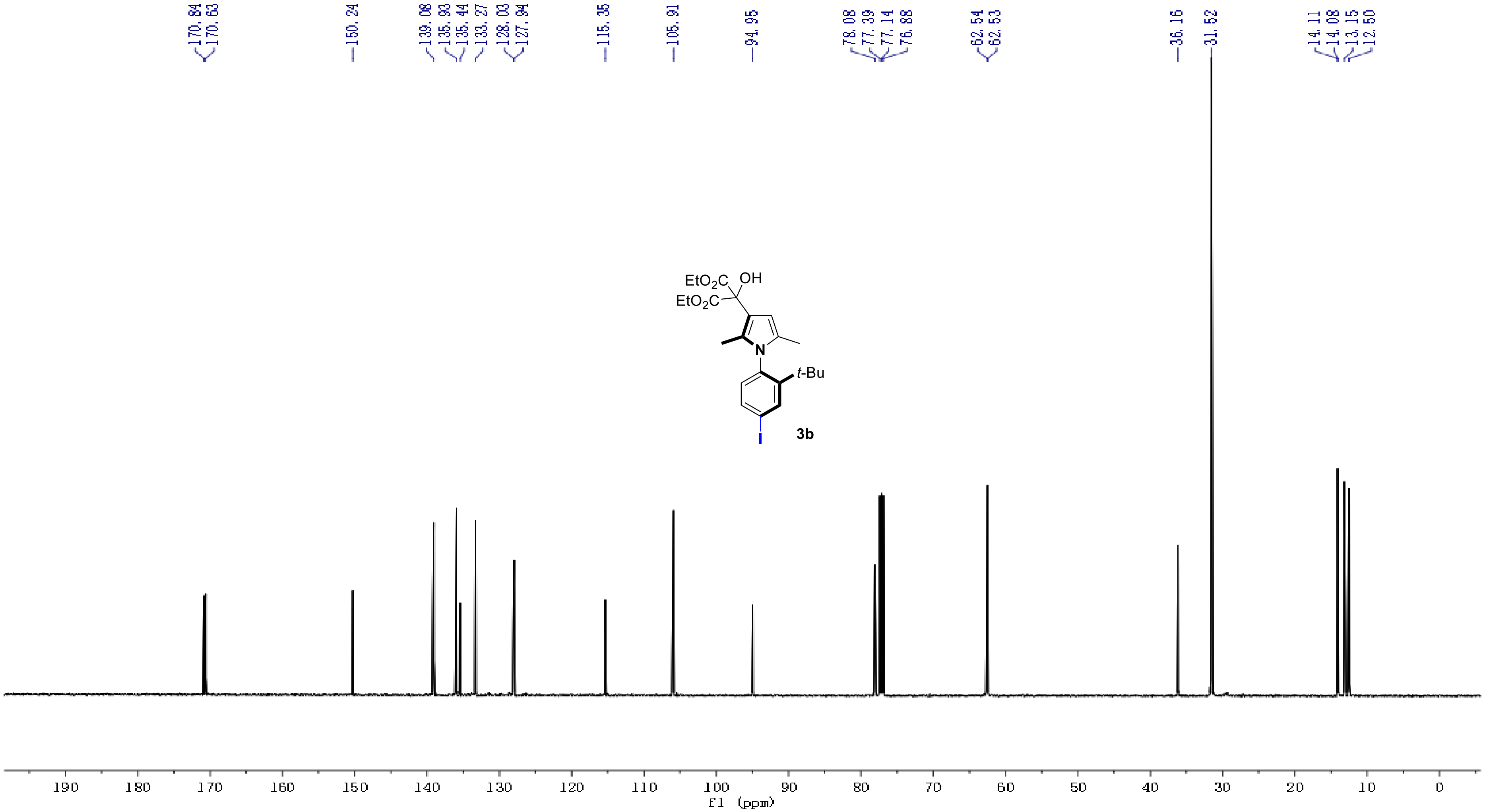


**Supplementary Figure 55.** $^{13}$C NMR of **3b**.

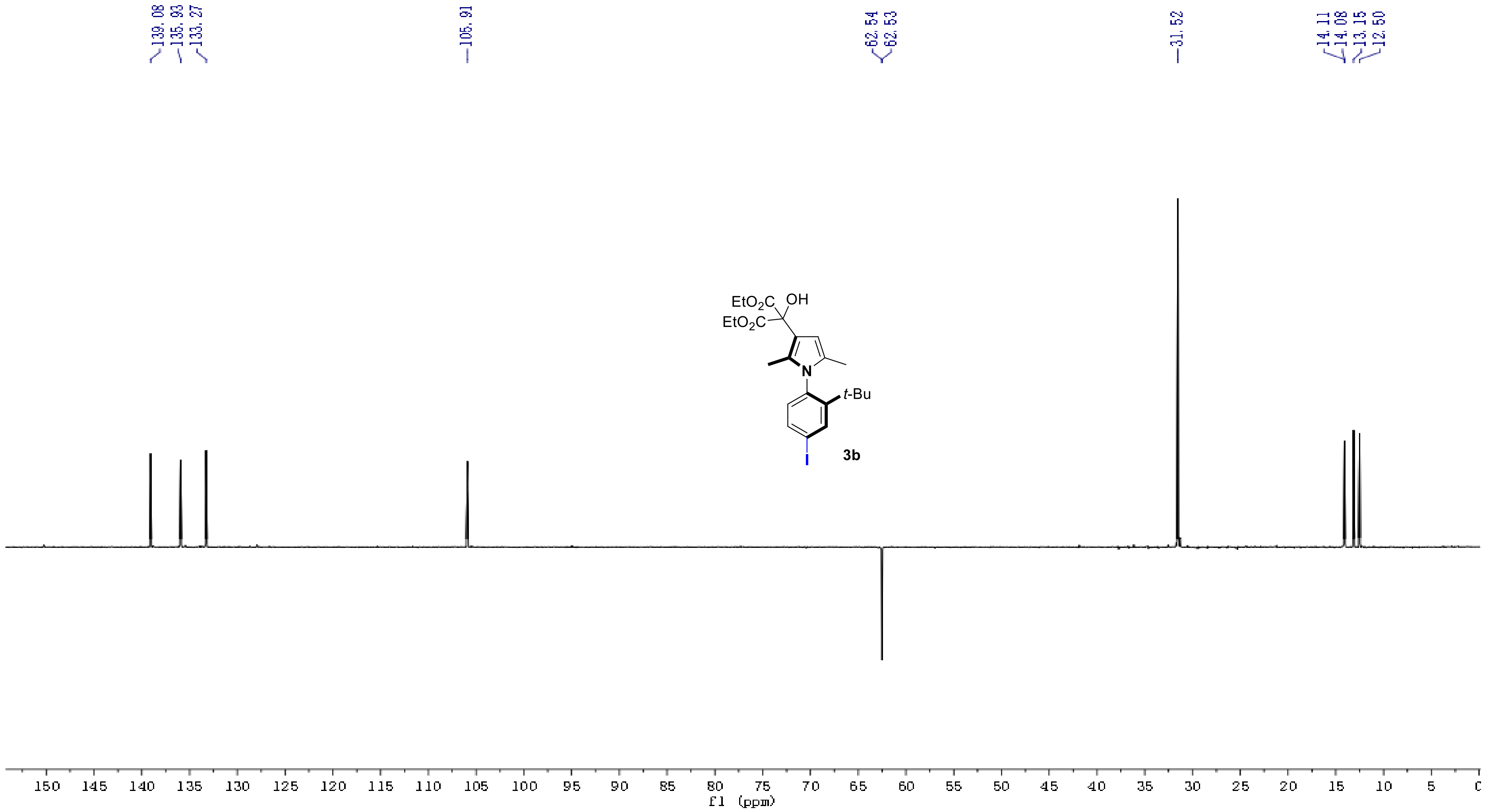


**Supplementary Figure 56.** $^{13}$C NMR-DEPT 135 of **3b**.

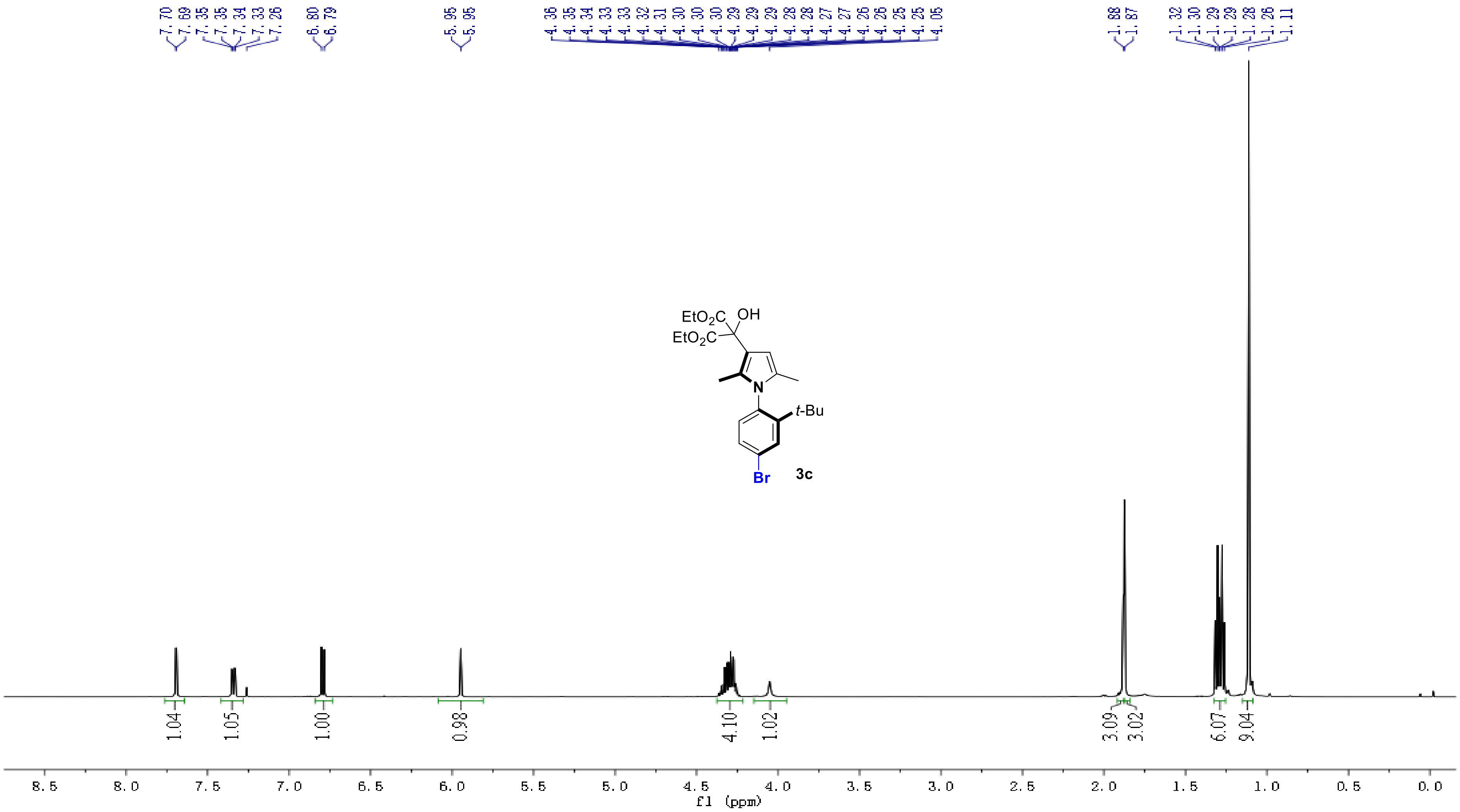

**Supplementary Figure 57.** $^{1}$H NMR of **3c**.

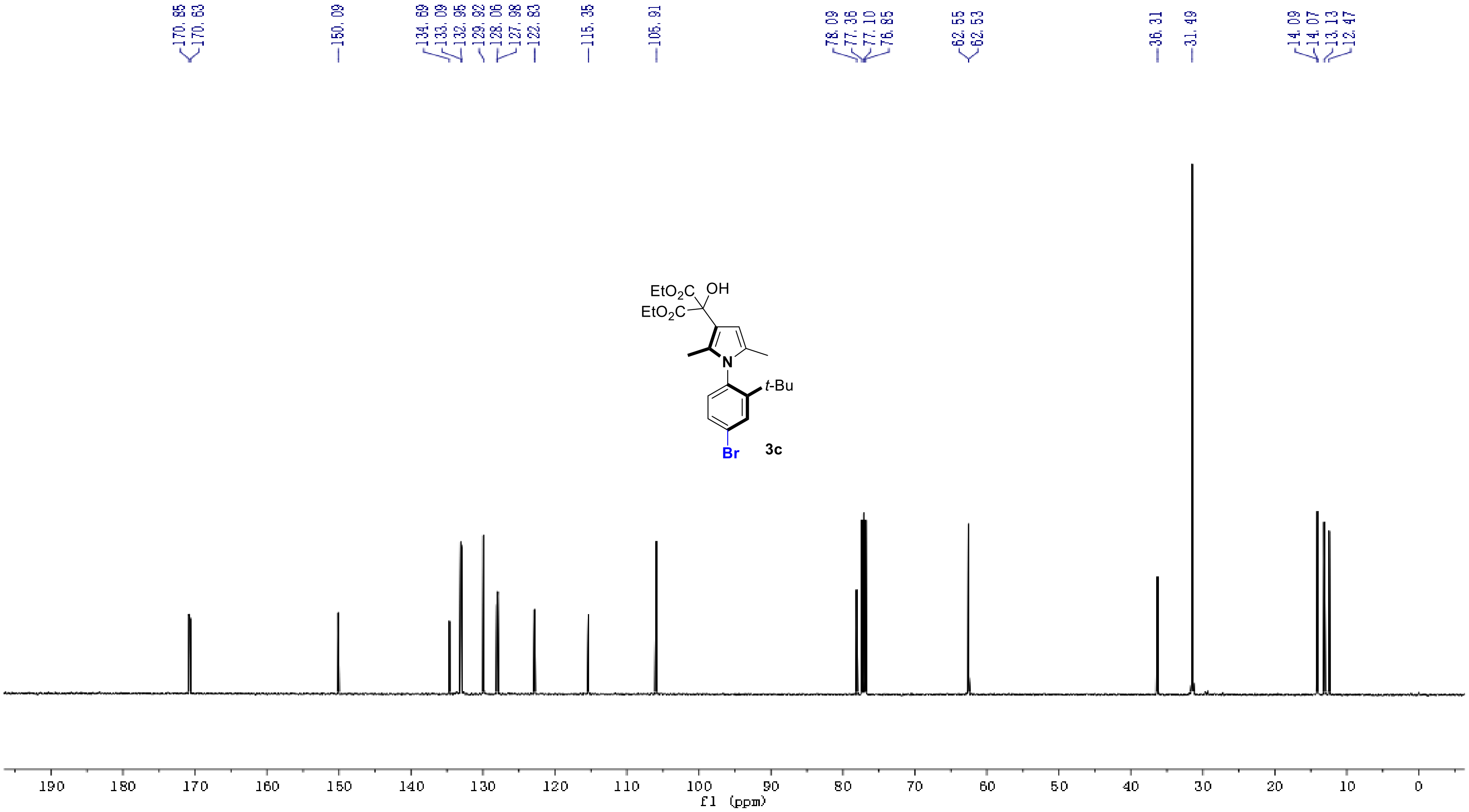


**Supplementary Figure 58.** $^{13}$C NMR of **3c**.

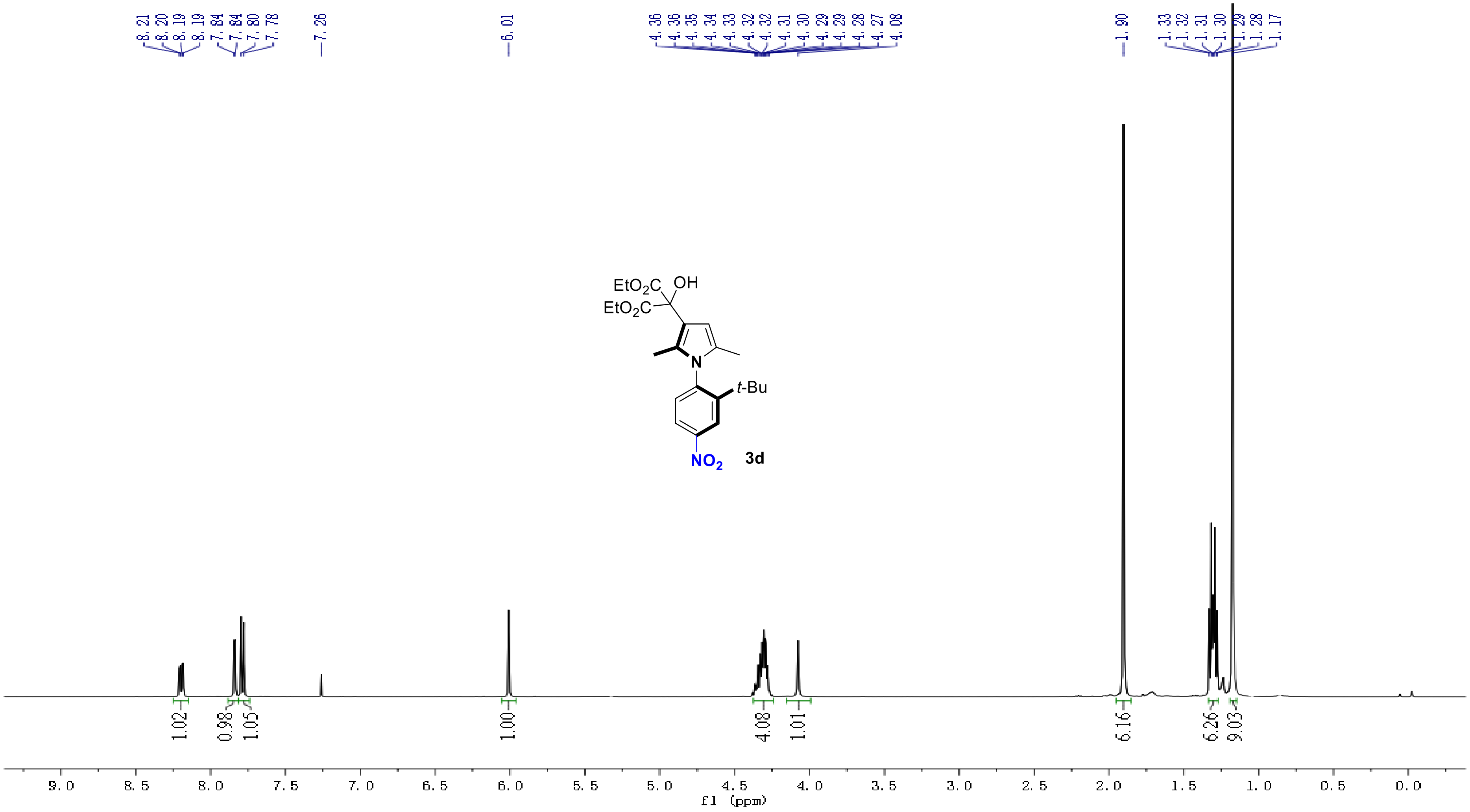


**Supplementary Figure 59.** $^{1}$H NMR of **3d**.

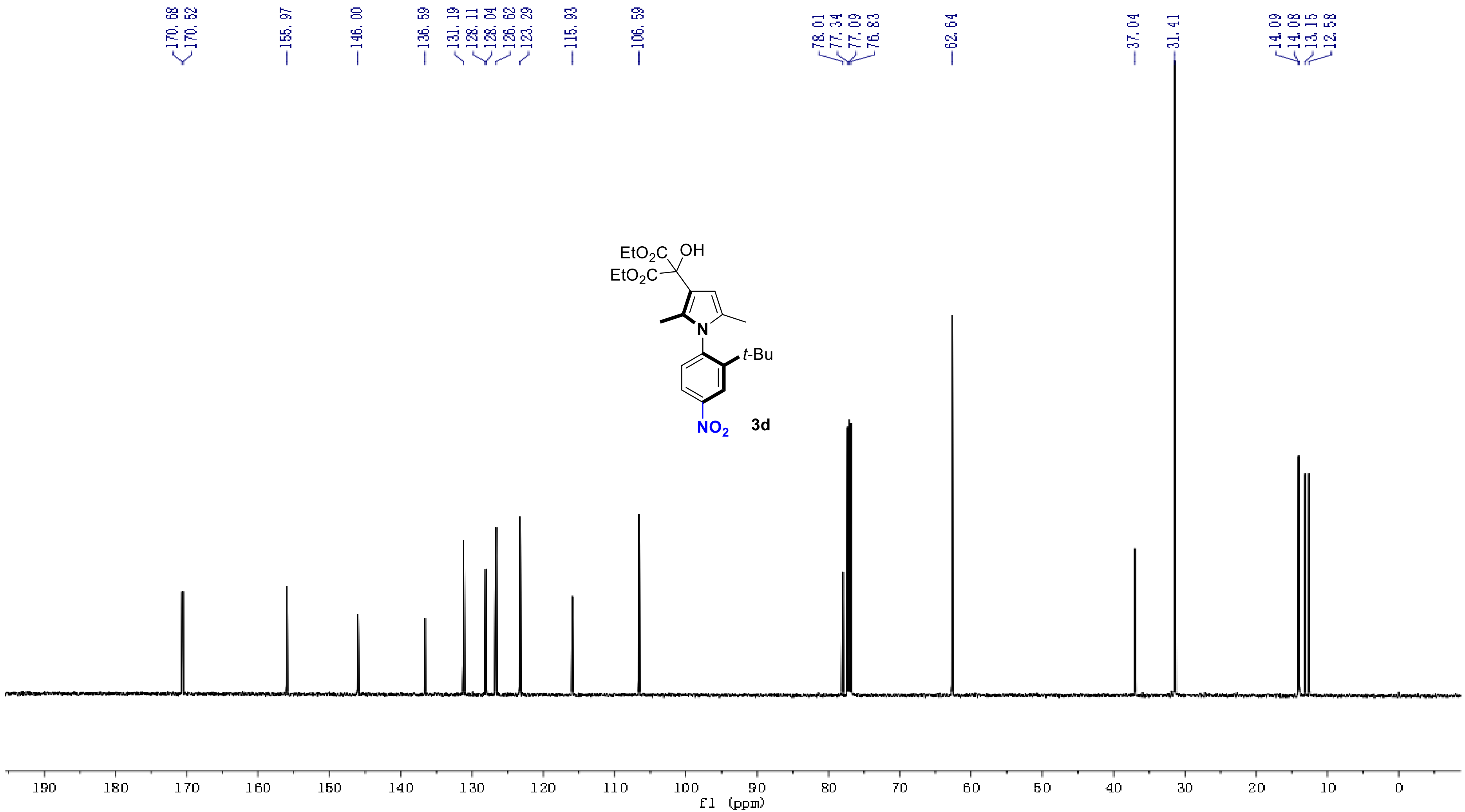


**Supplementary Figure 60.** $^{13}$C NMR of **3d**.

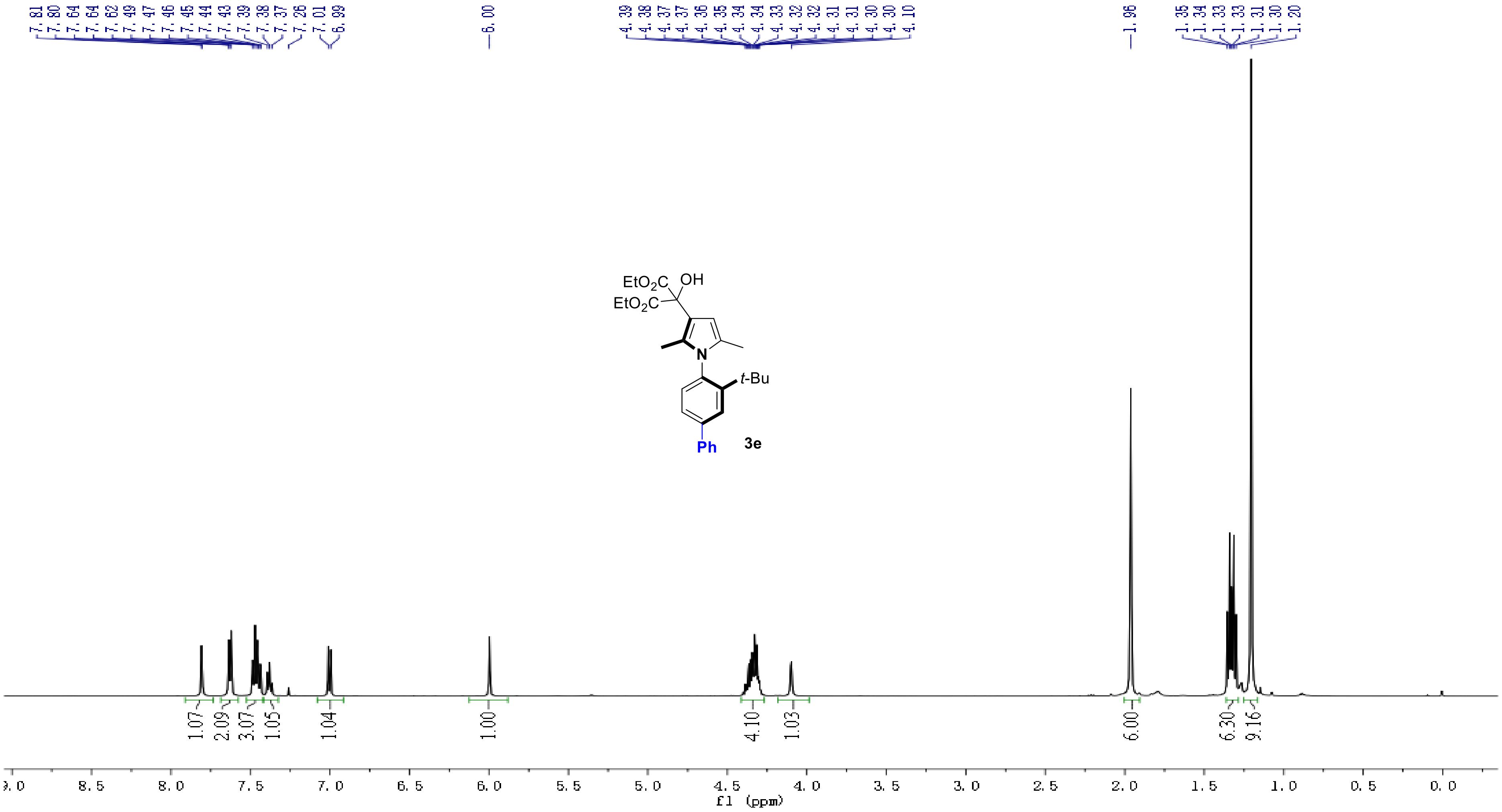

**Supplementary Figure 61.** $^{1}$H NMR of **3e**.

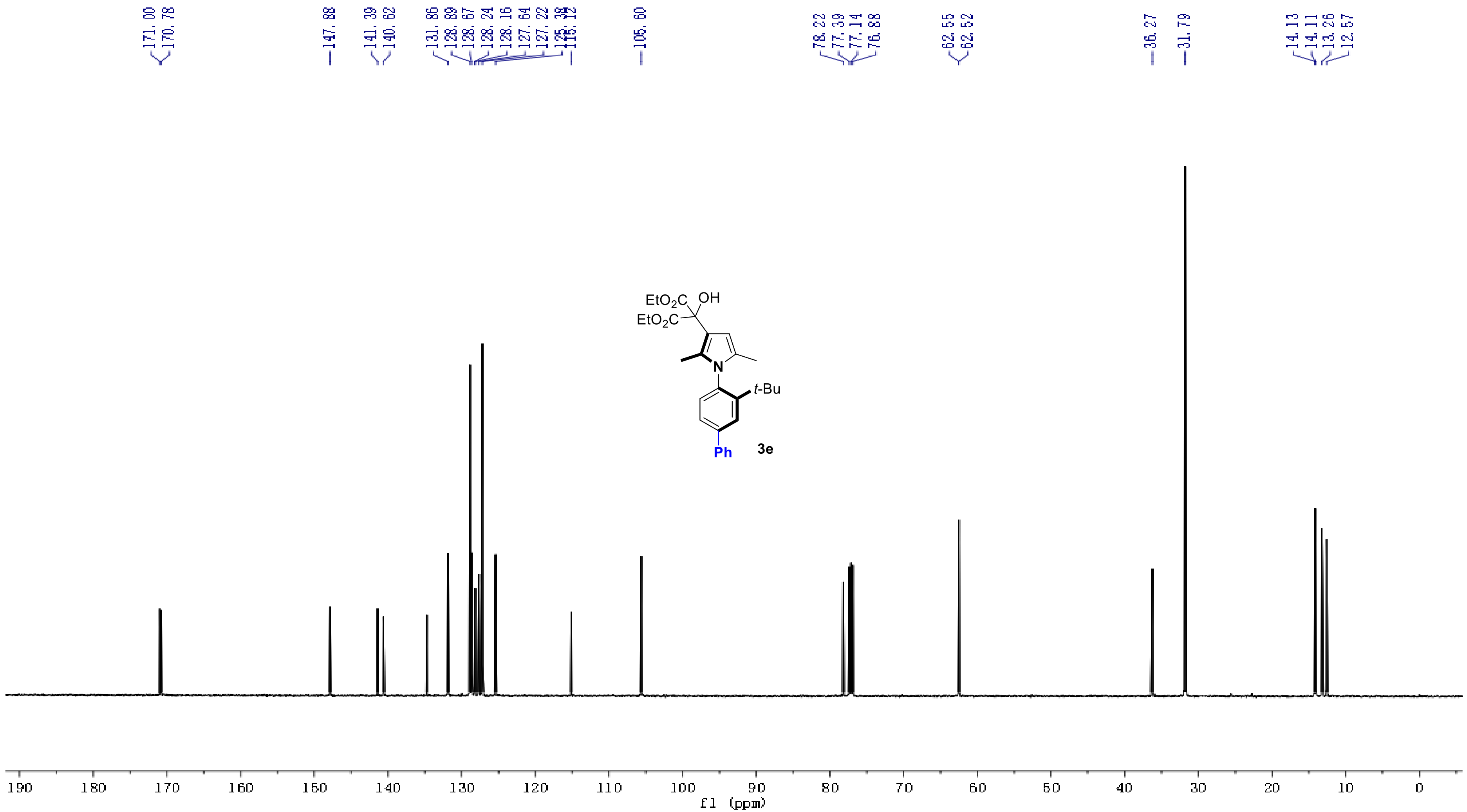


**Supplementary Figure 62.** $^{13}$C NMR of **3e**.

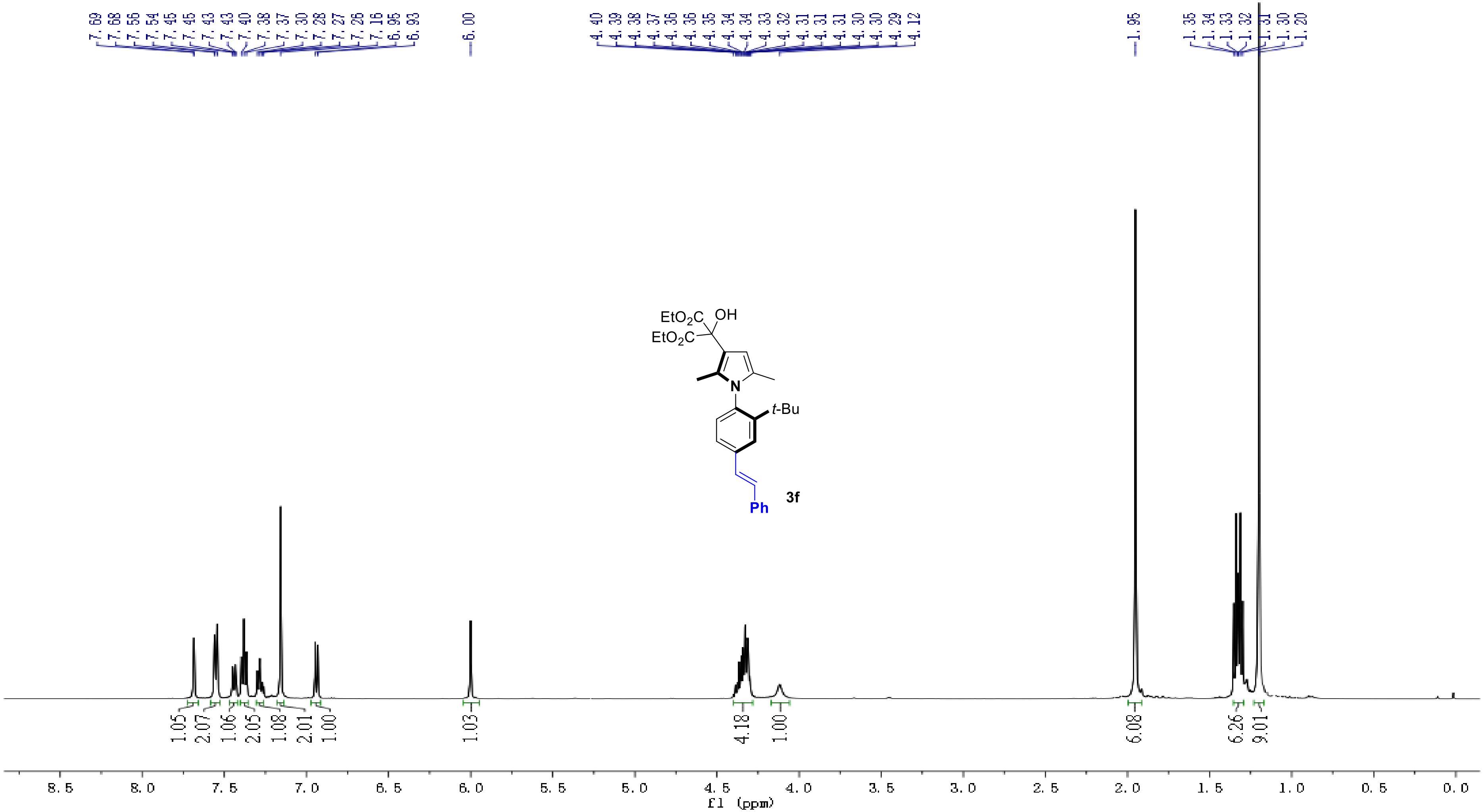


**Supplementary Figure 63.** $^{1}$H NMR of **3f**.

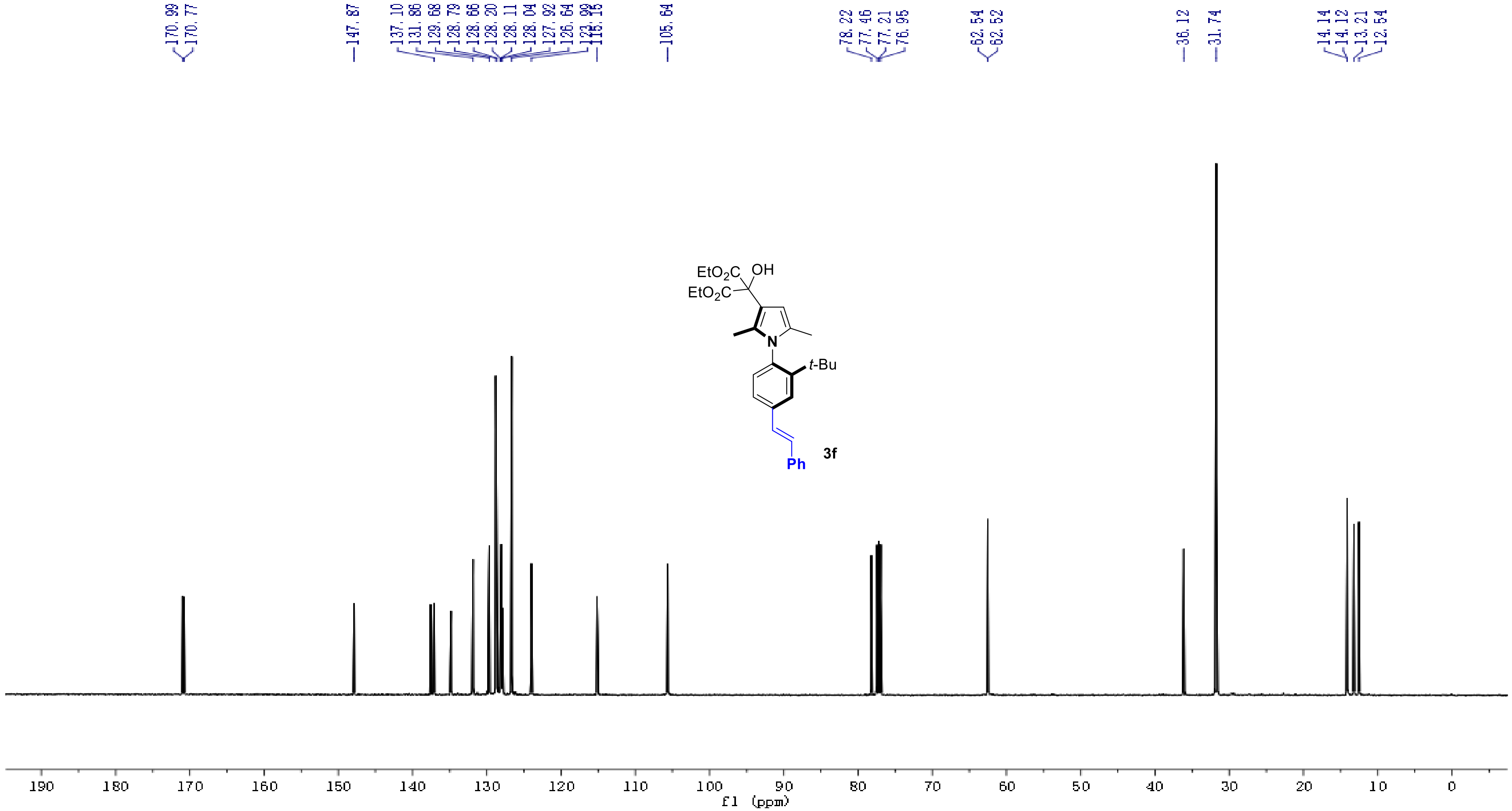


**Supplementary Figure 64.** $^{13}$C NMR of **3f**.

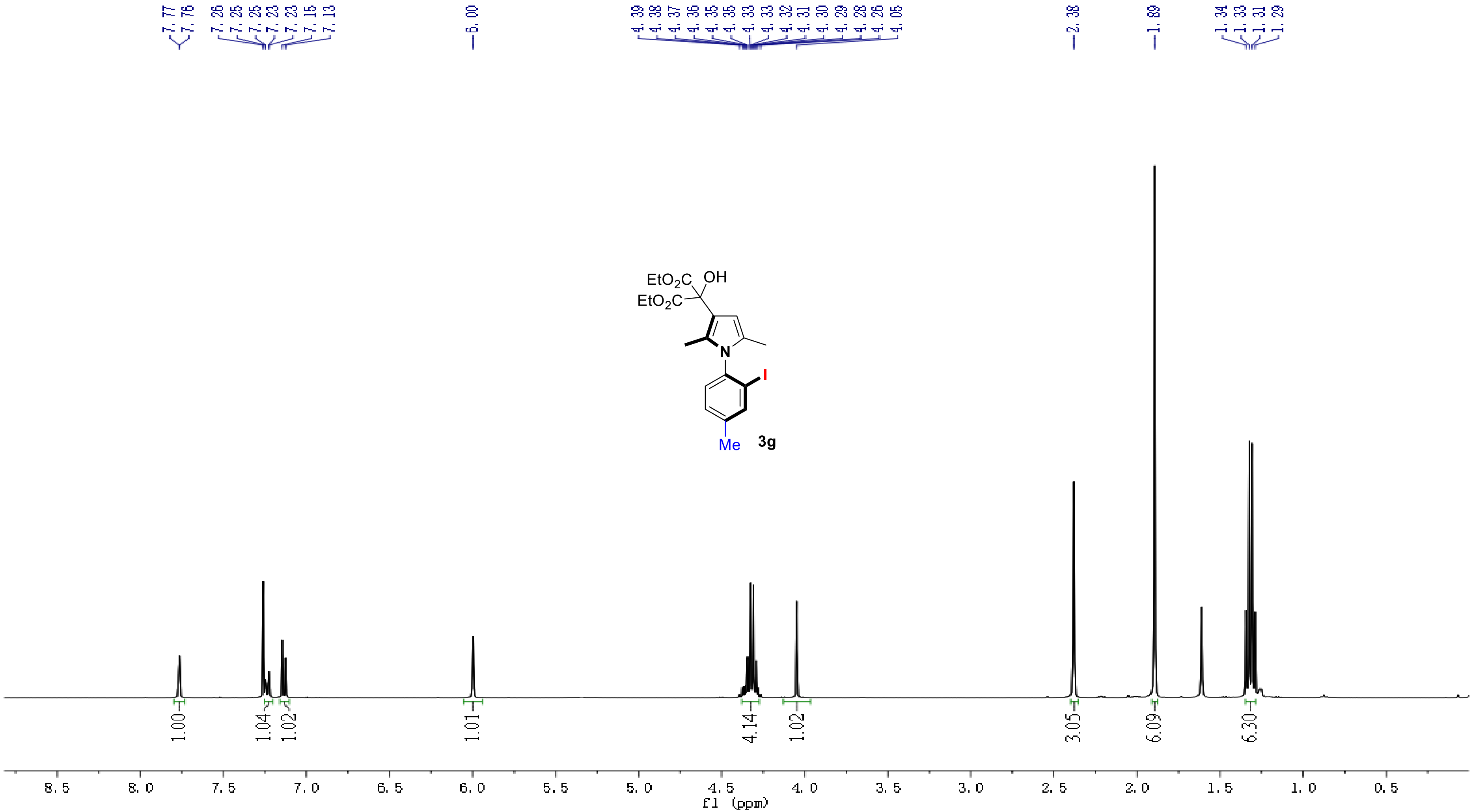


**Supplementary Figure 65.** $^1$H NMR of **3g**.

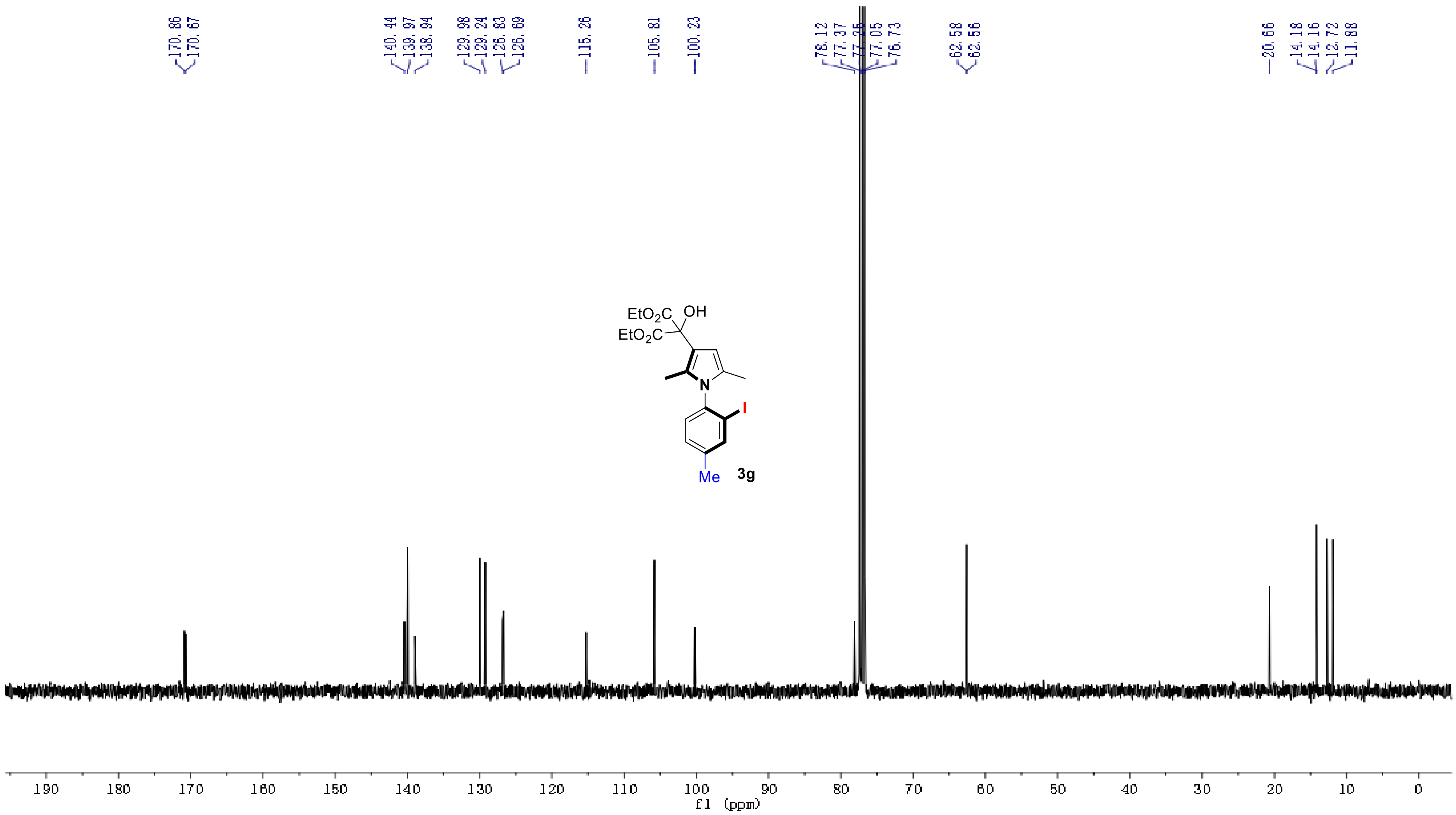


**Supplementary Figure 66.** $^{13}$C NMR of **3g**.

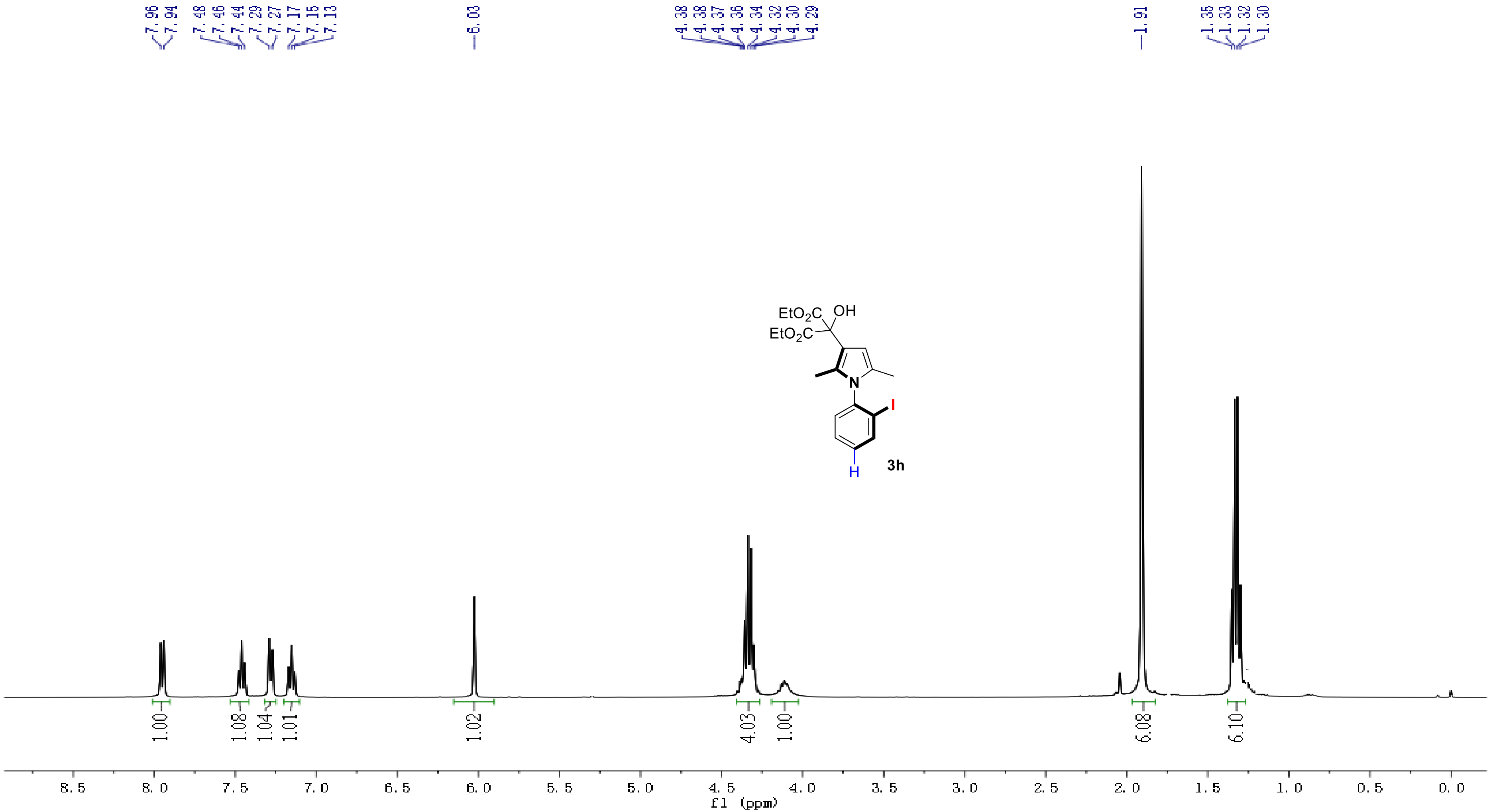


**Supplementary Figure 67.** $^{1}$H NMR of **3h**.

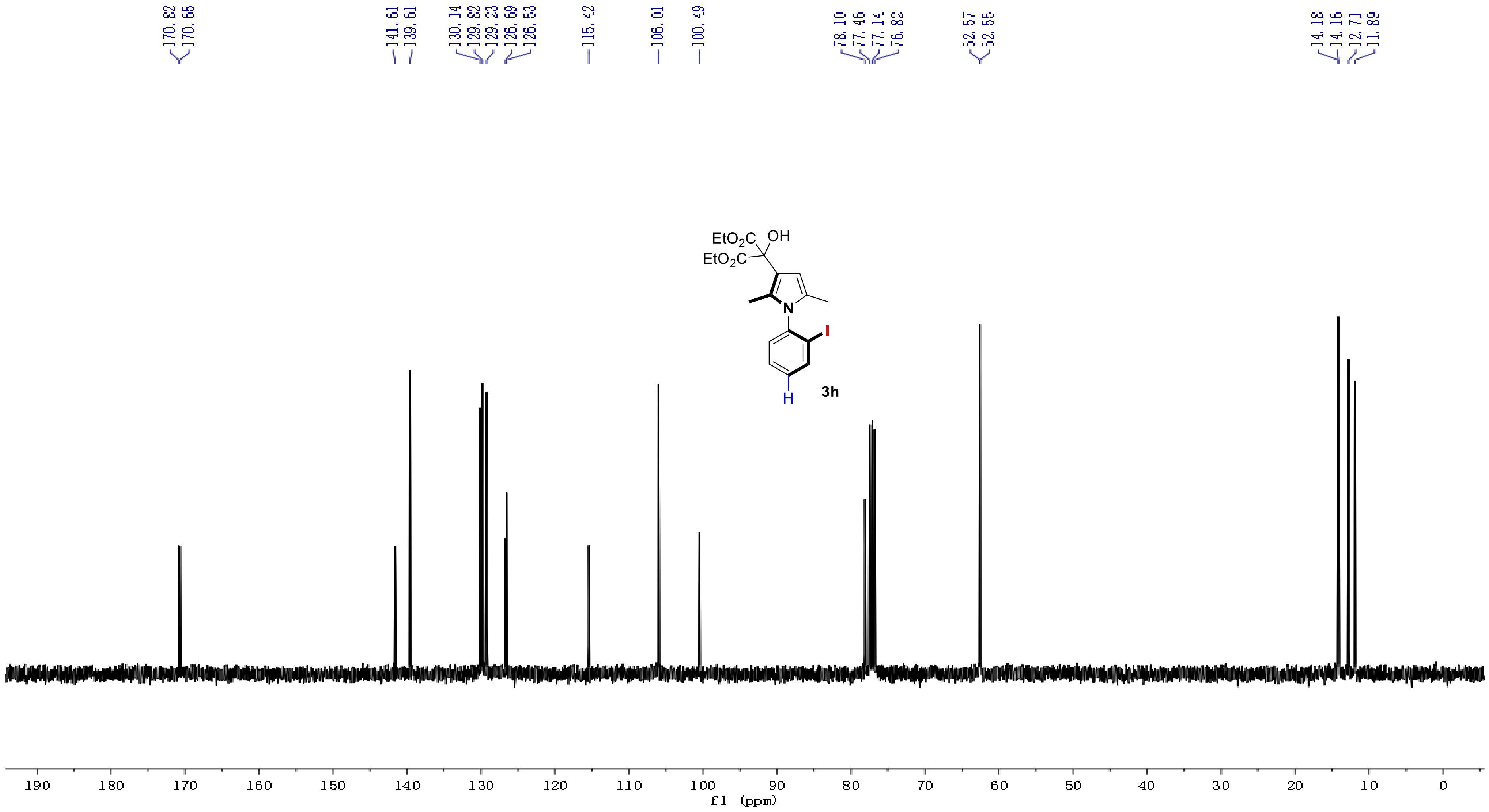


**Supplementary Figure 68.** $^{13}$C NMR of **3h**.

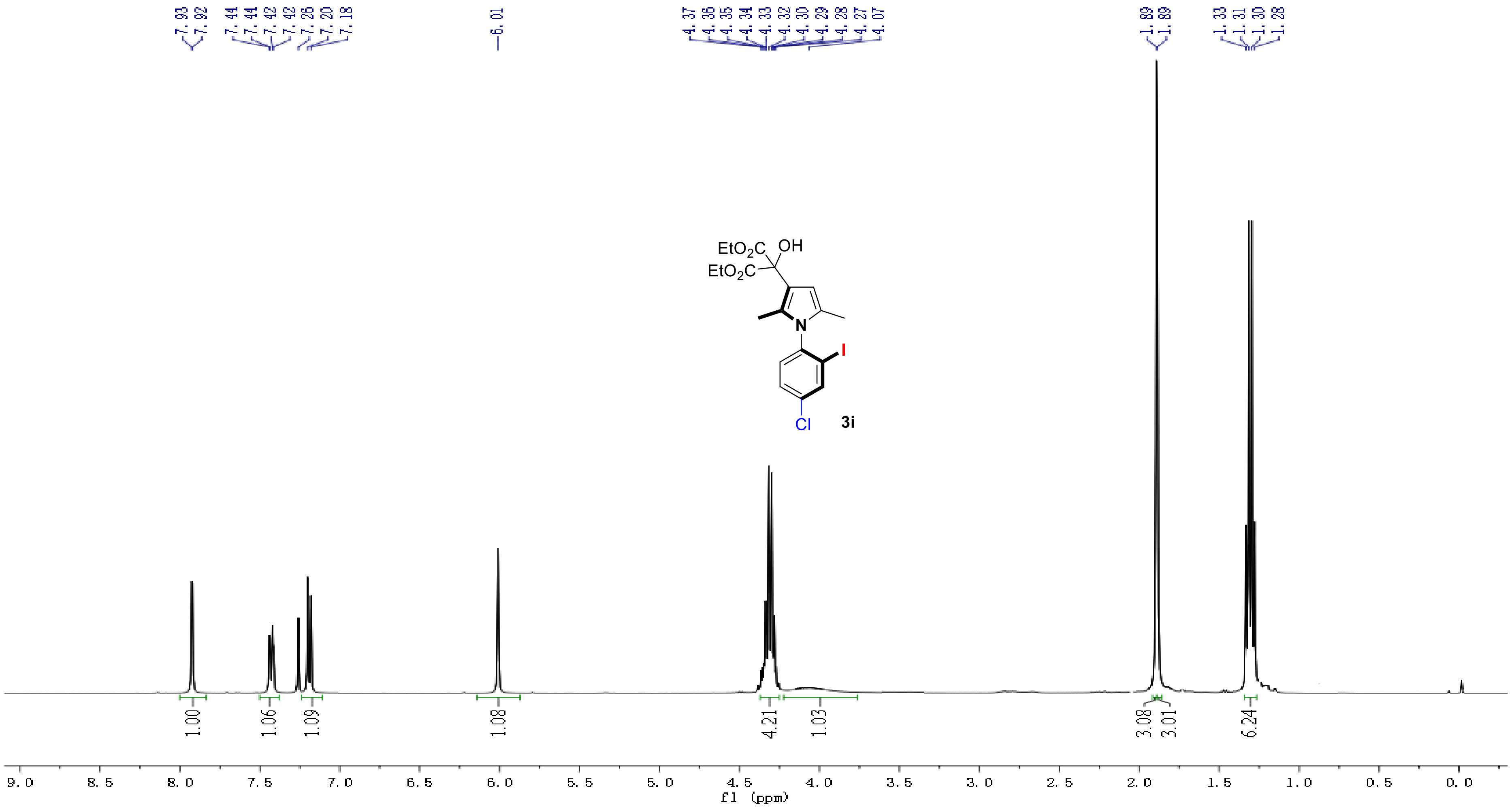


**Supplementary Figure 69.** $^{1}$H NMR of **3i**.

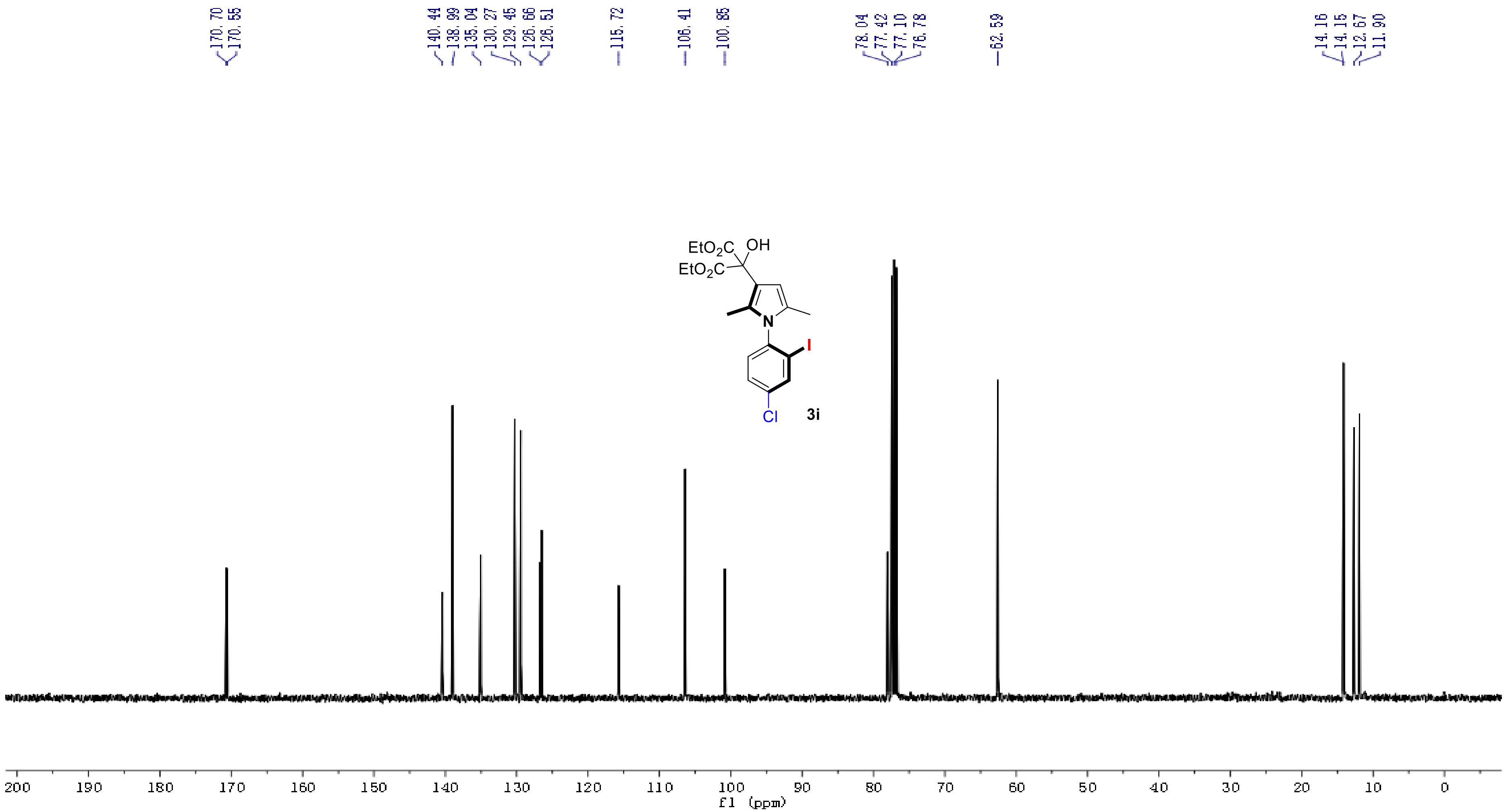


**Supplementary Figure 70.** $^{13}$C NMR of **3i**.

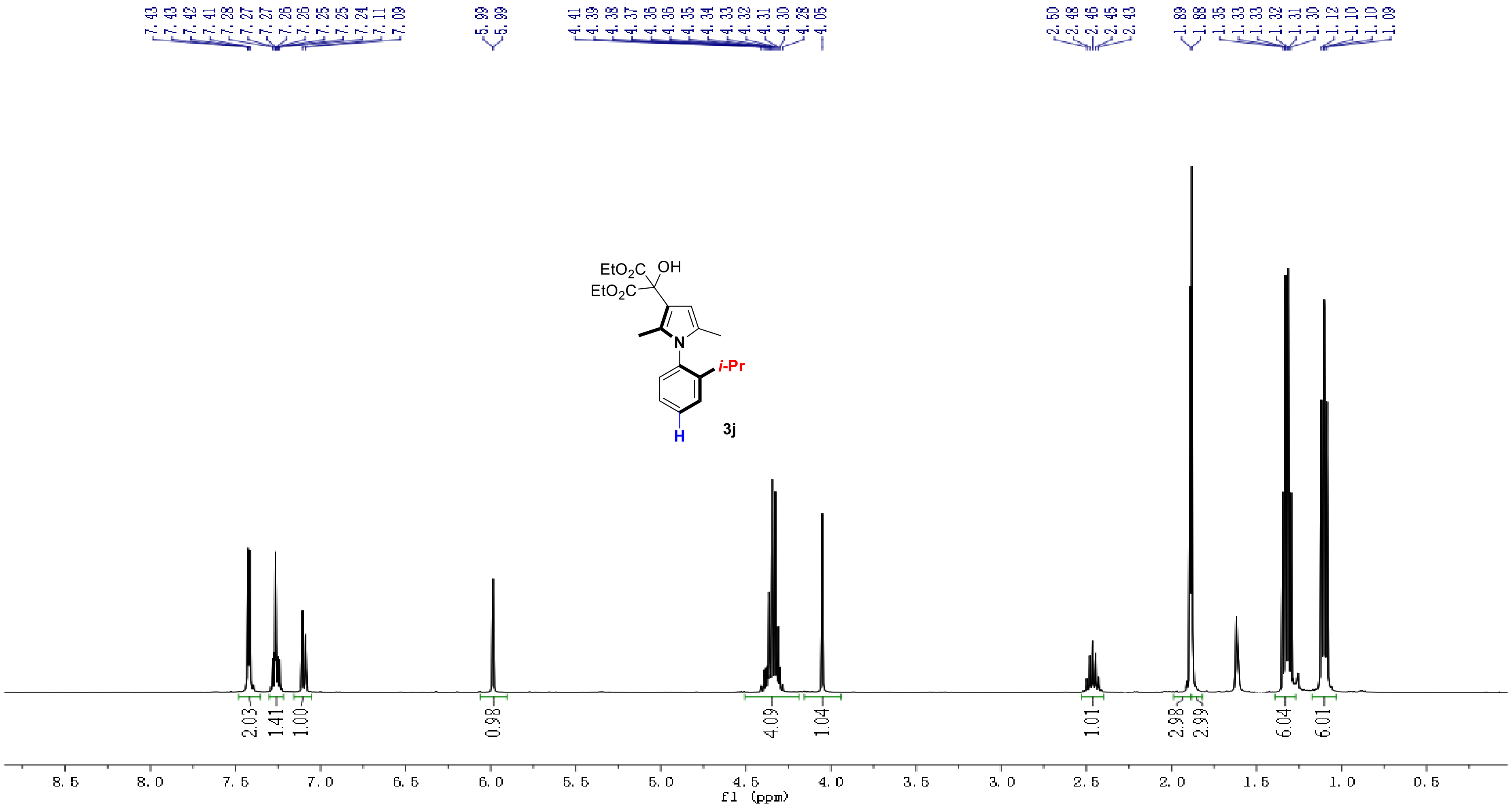


**Supplementary Figure 71.** $^{1}$H NMR of **3j**.

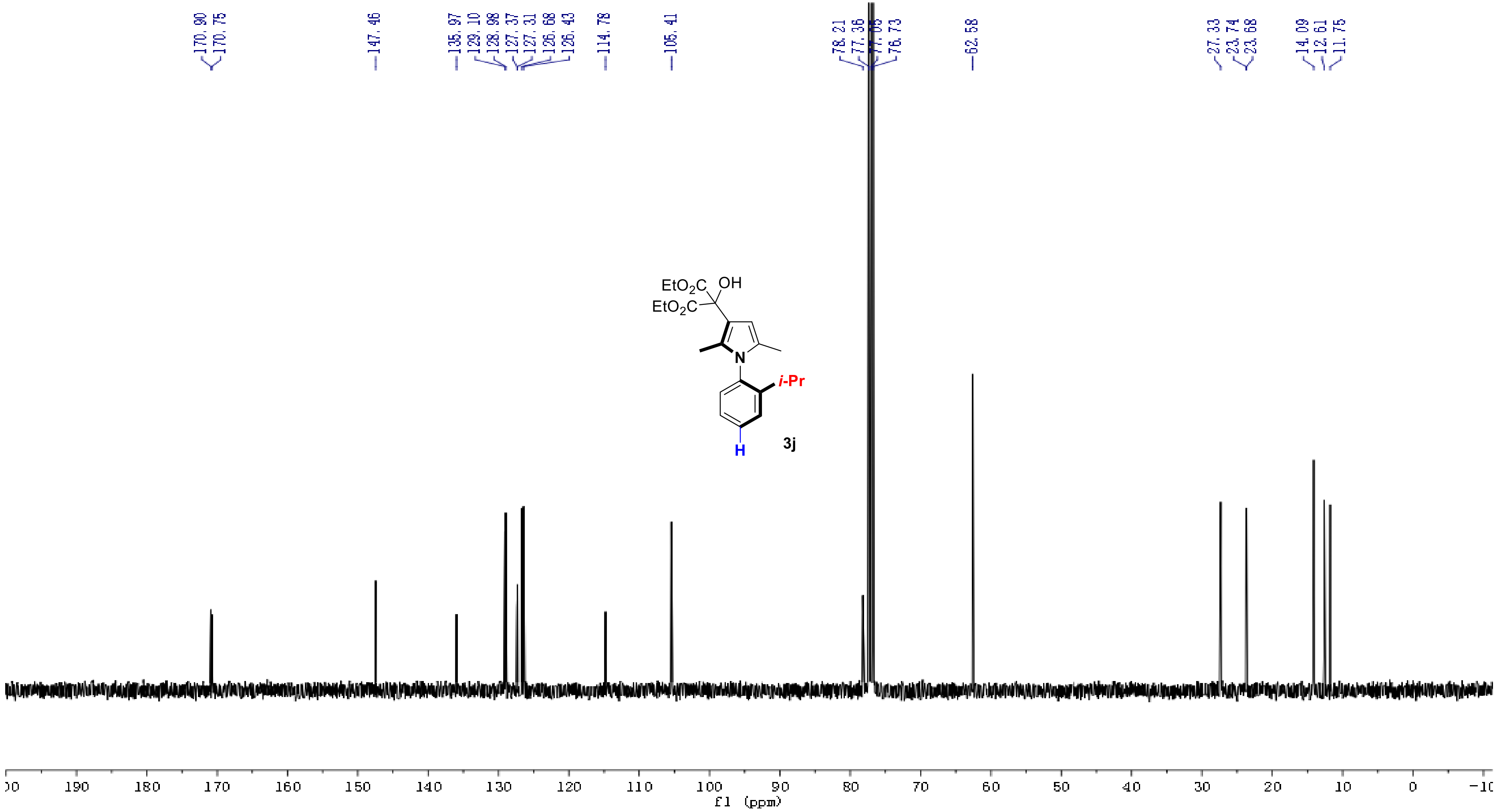


**Supplementary Figure 72.** $^{13}$C NMR of **3j**.

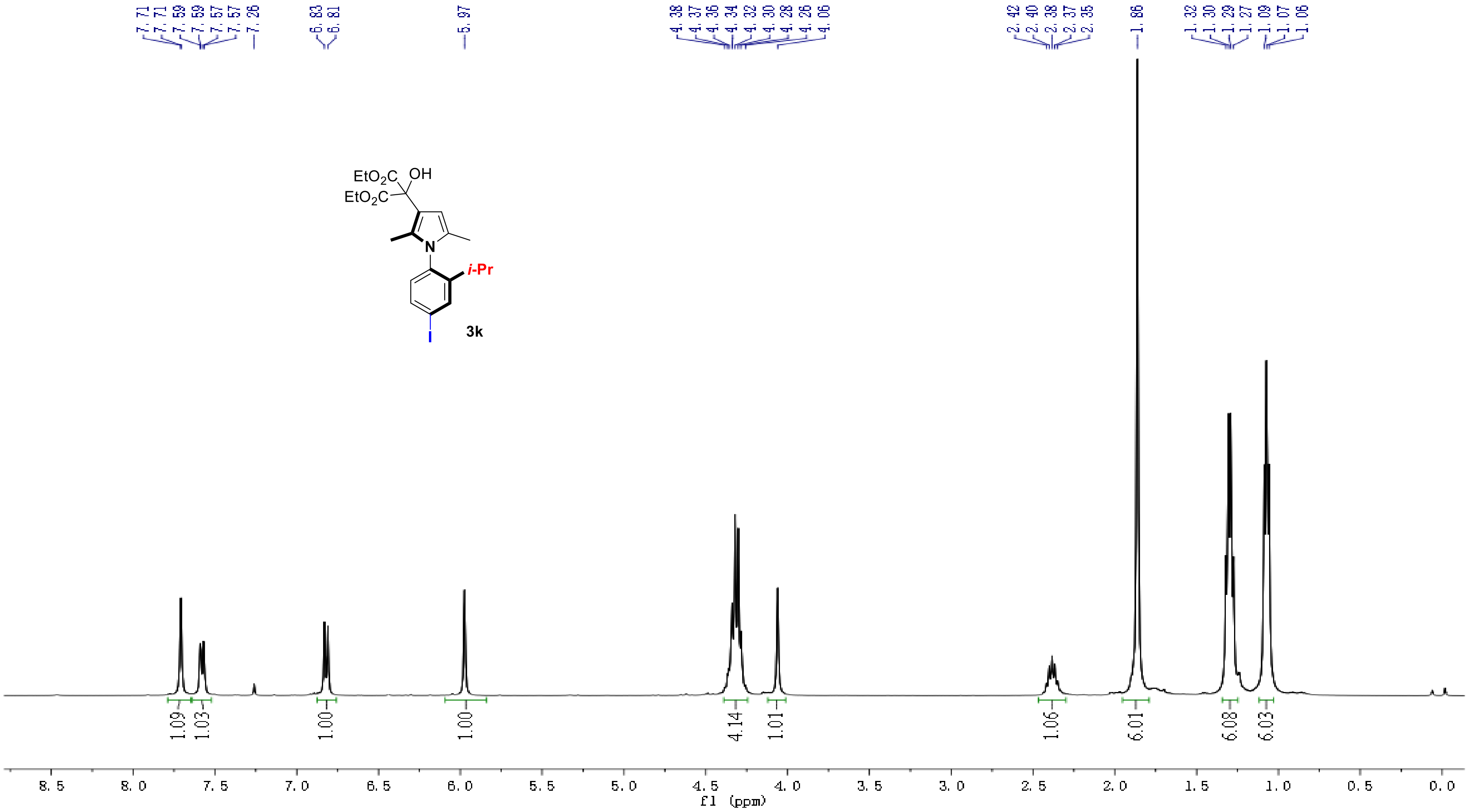


**Supplementary Figure 73.** $^{1}$H NMR of **3k**.

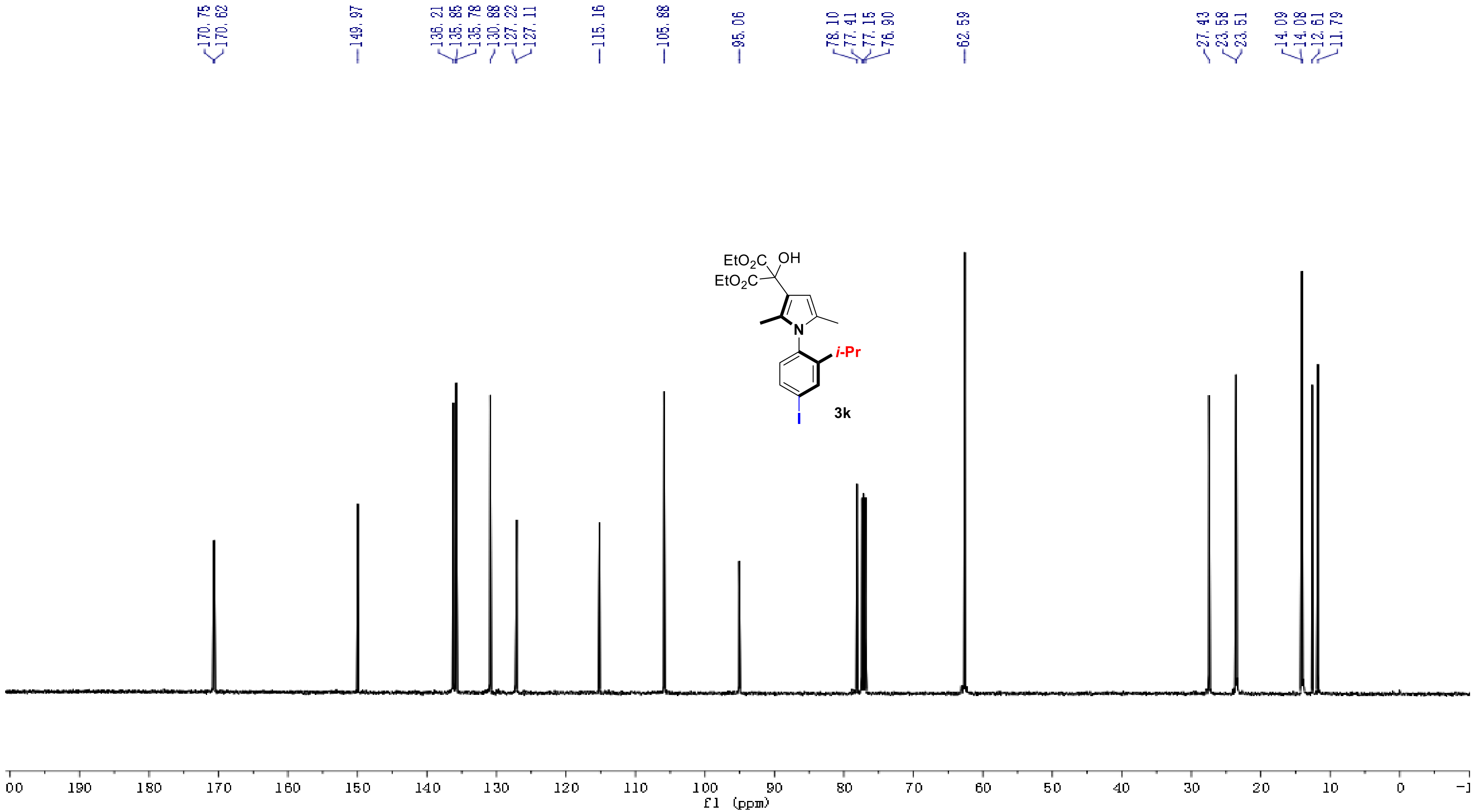


**Supplementary Figure 74.** $^{13}$C NMR of **3k**.

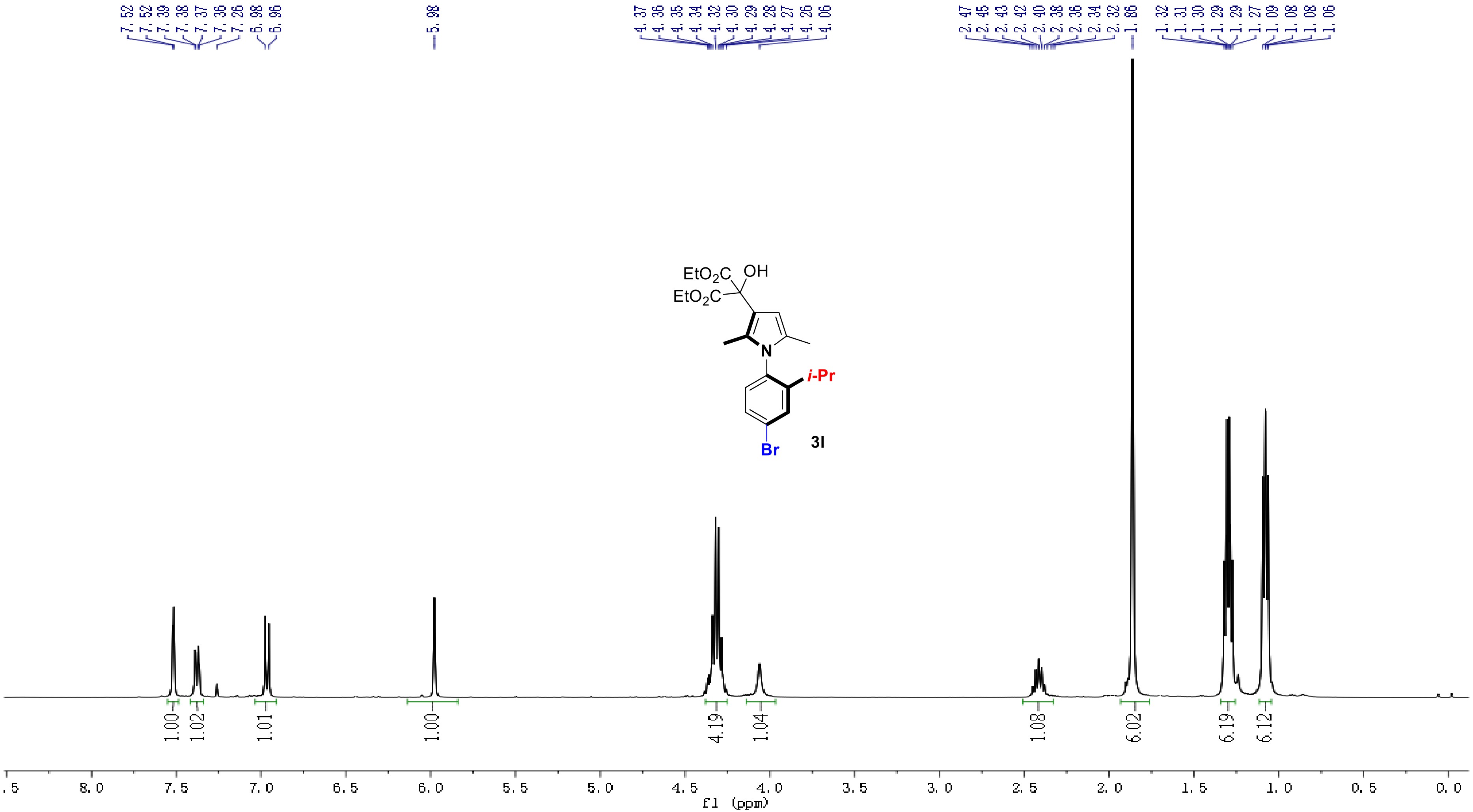


**Supplementary Figure 75.** $^{1}$H NMR of **3l**.

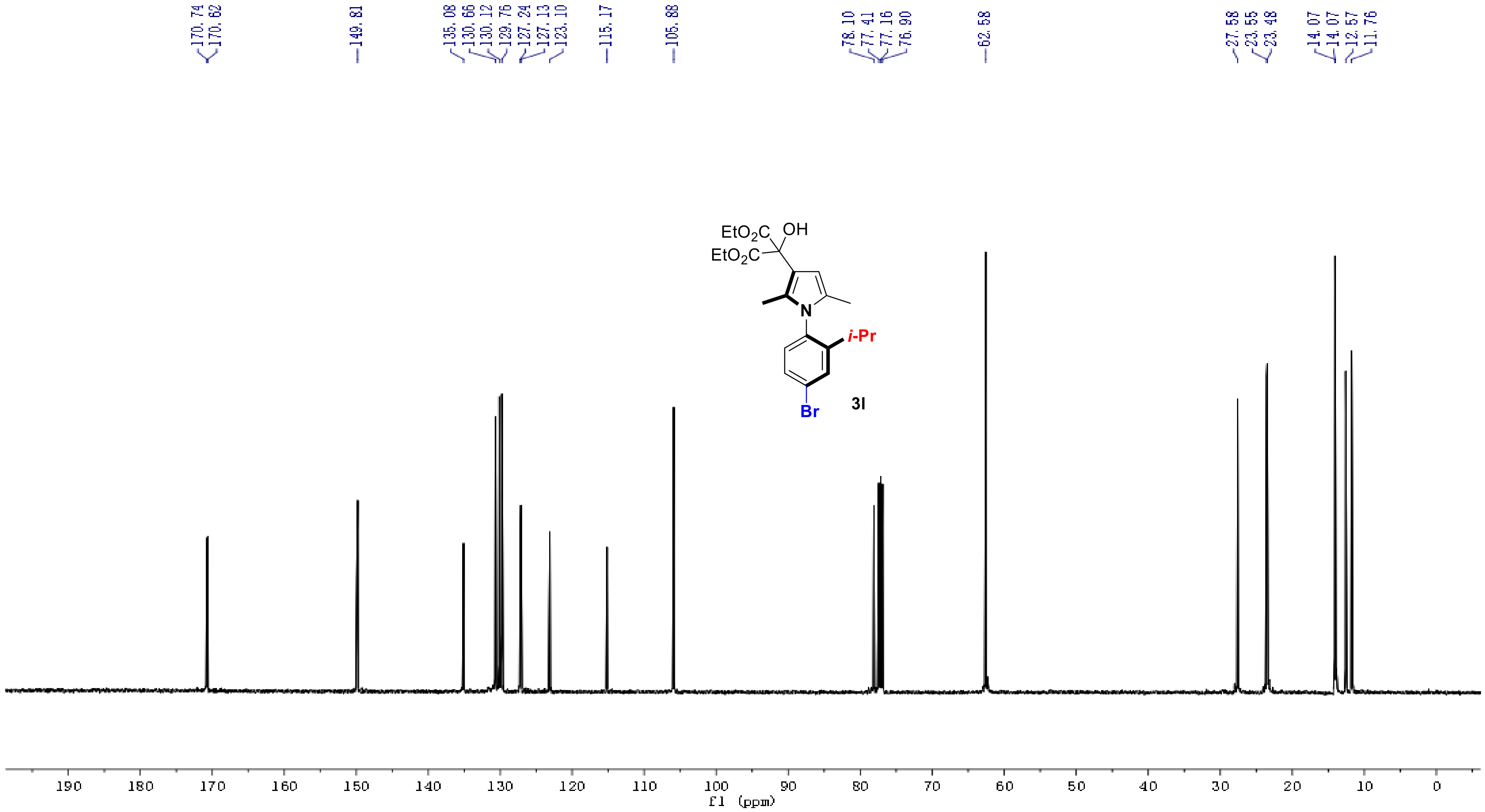


**Supplementary Figure 76.** $^{13}$C NMR of **3l**.

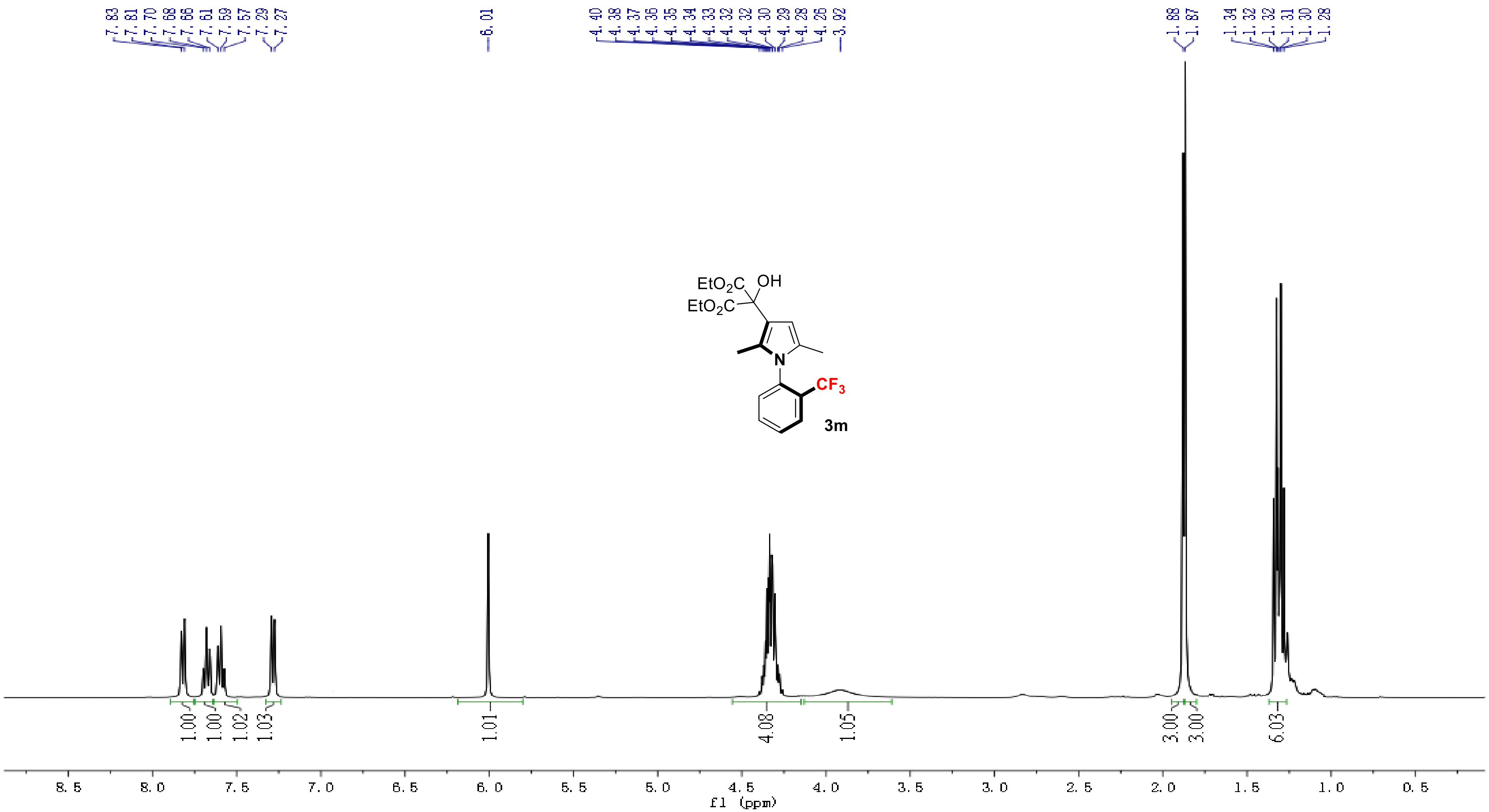


**Supplementary Figure 77.** $^{1}$H NMR of **3m**.

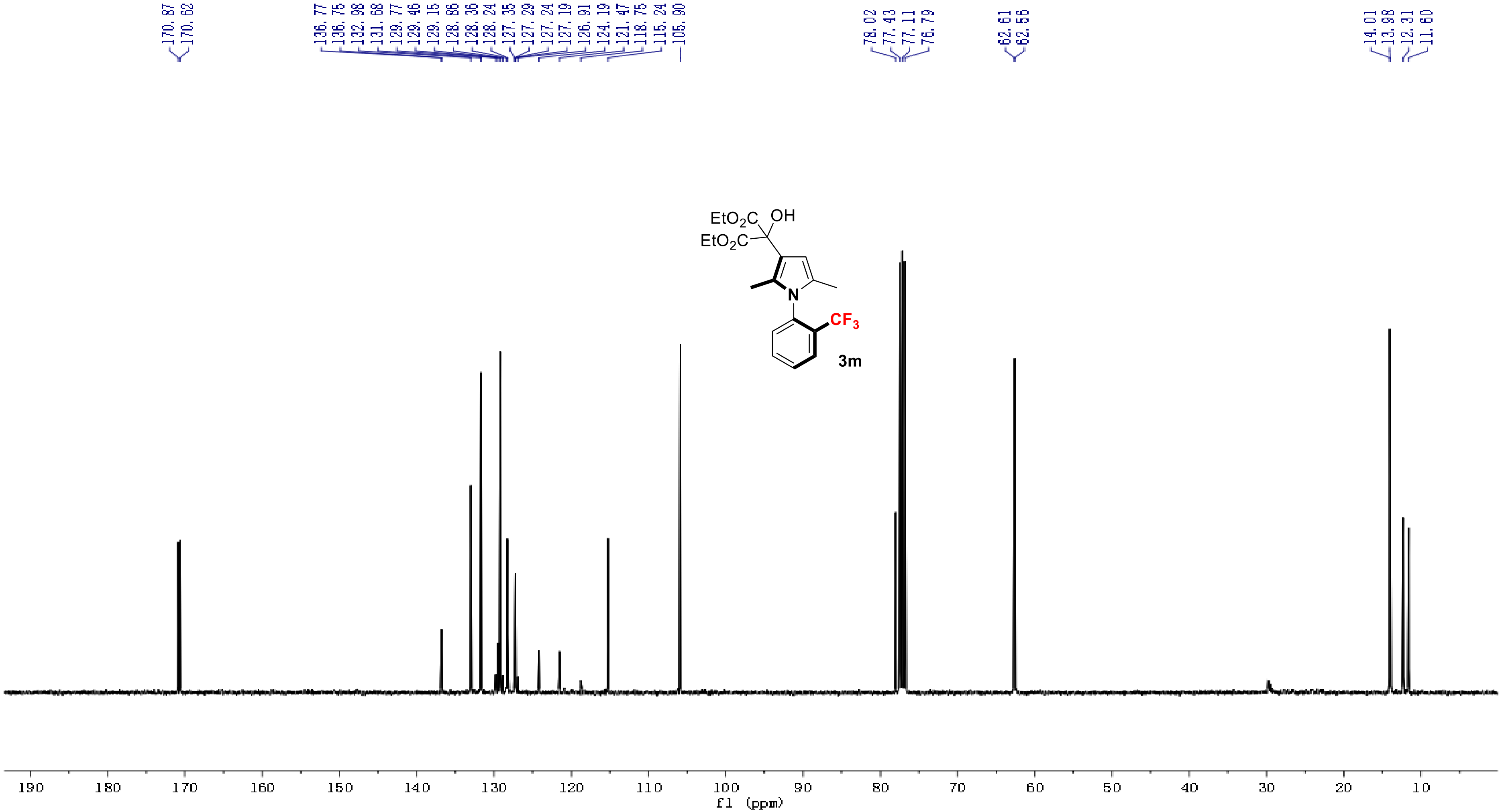


**Supplementary Figure 78.** $^{13}$C NMR of **3m**.

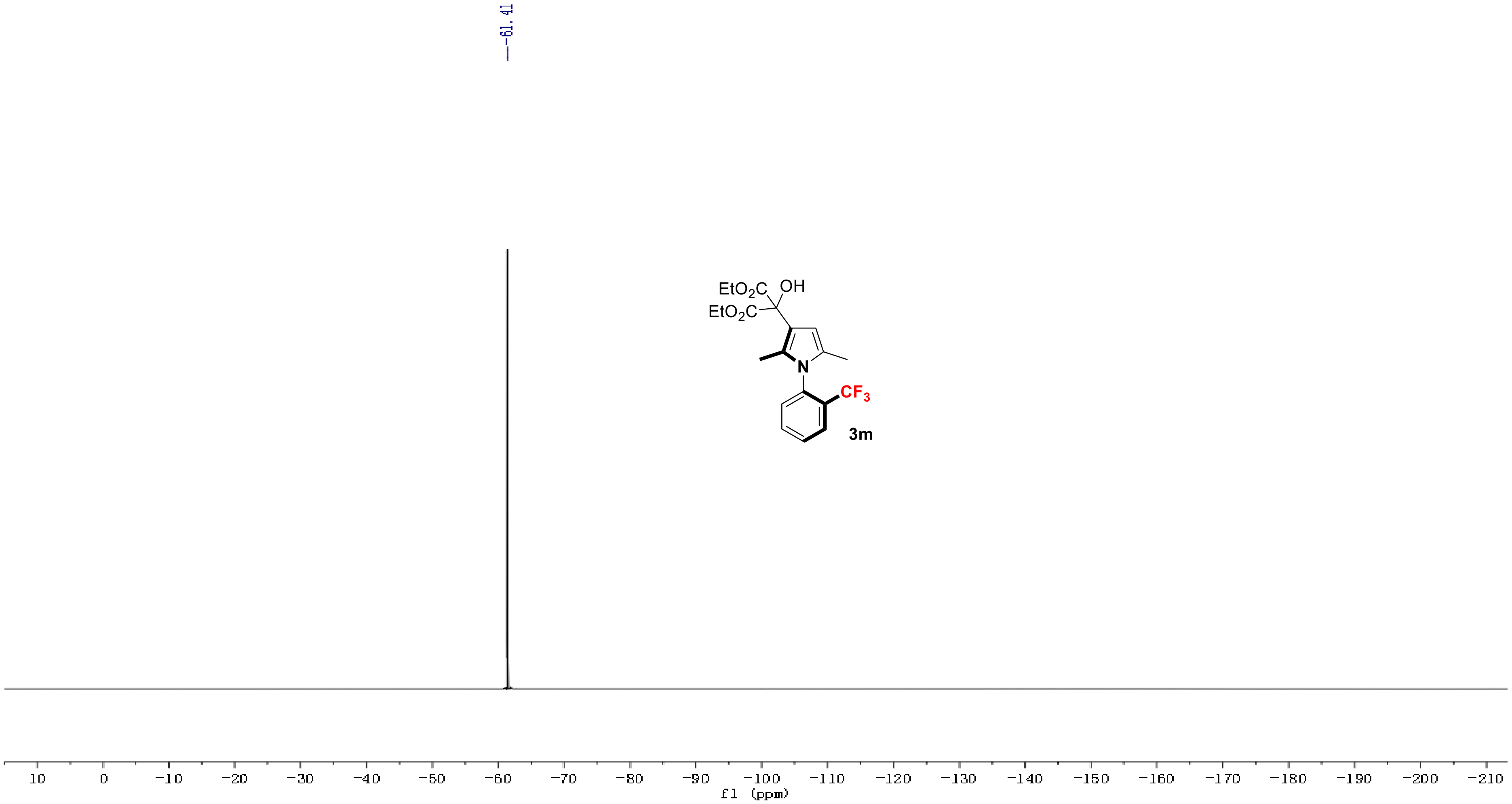


**Supplementary Figure 79.** $^{19}$F NMR of **3m**.

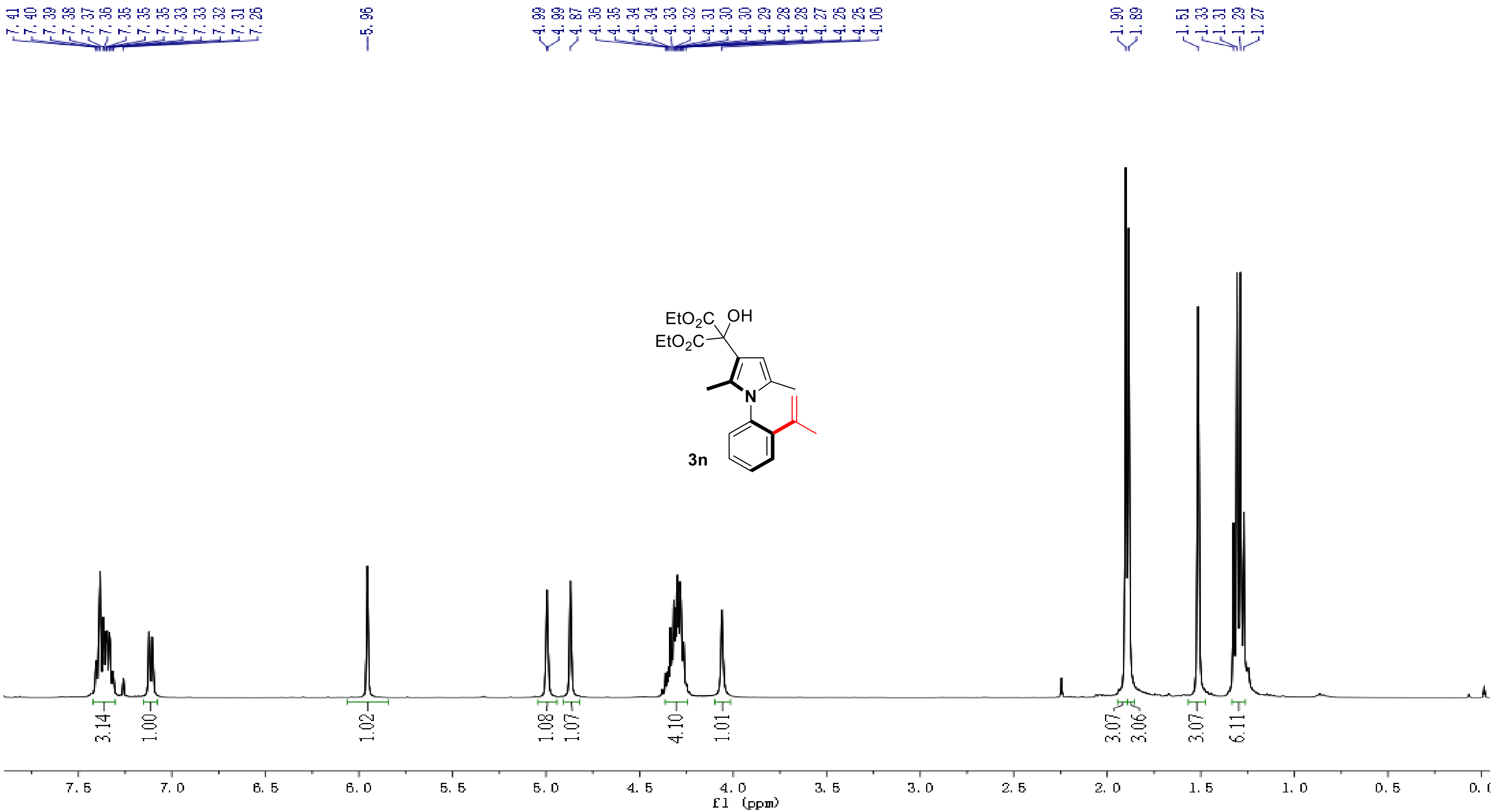


**Supplementary Figure 80.** $^1$H NMR of **3n**.

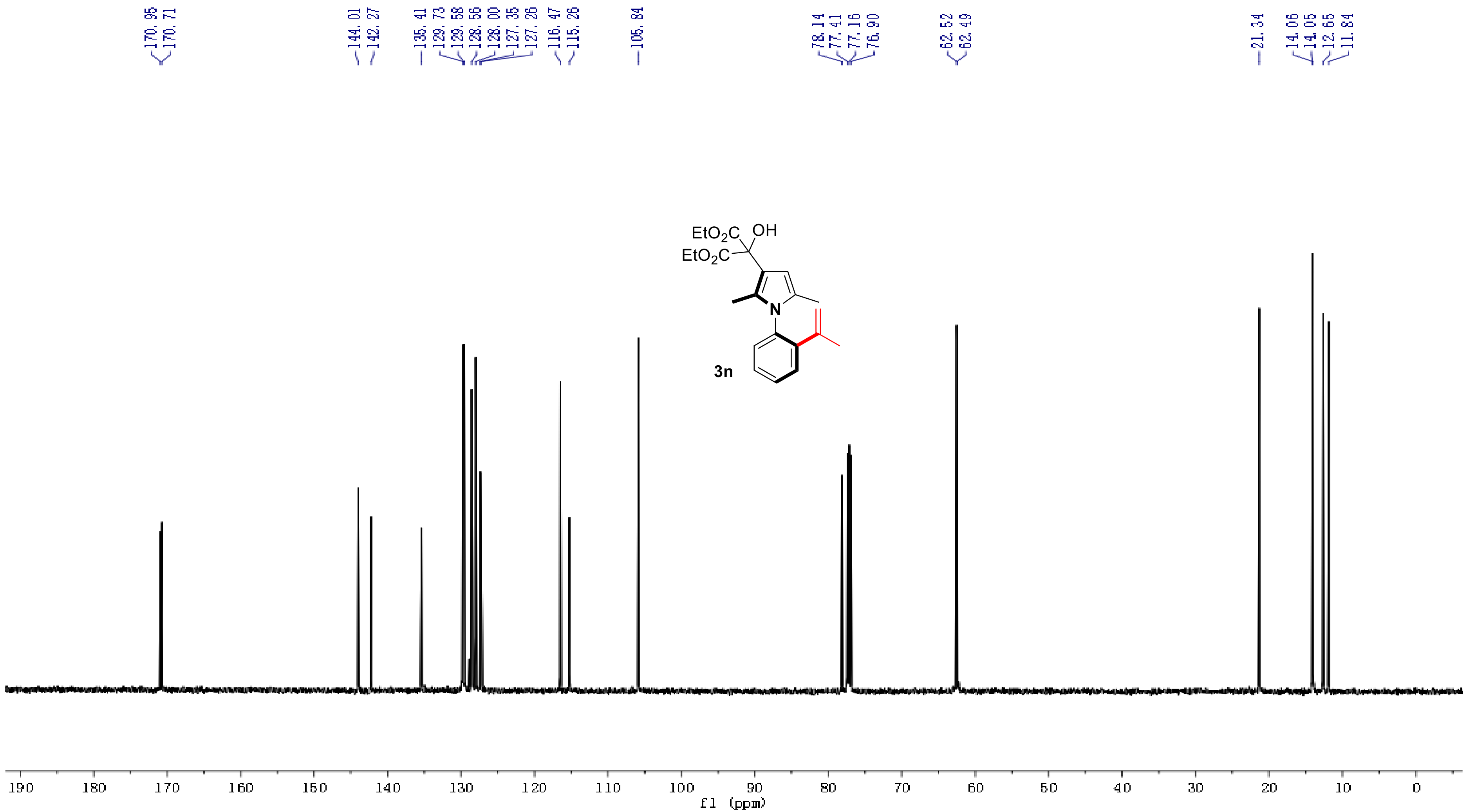


**Supplementary Figure 81.** $^{13}$C NMR of **3n**.

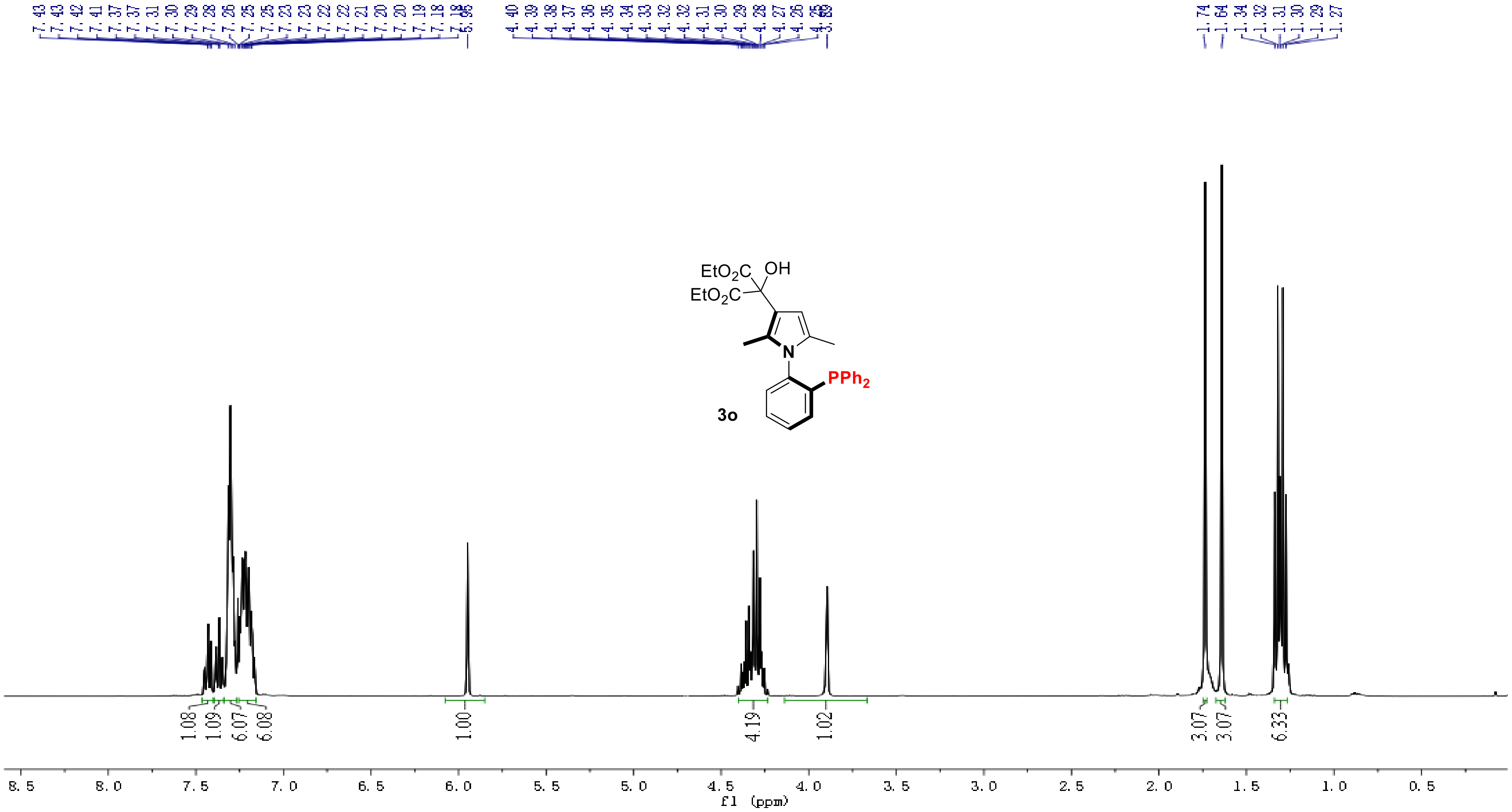

**Supplementary Figure 82.** $^{1}$H NMR of **3o**.

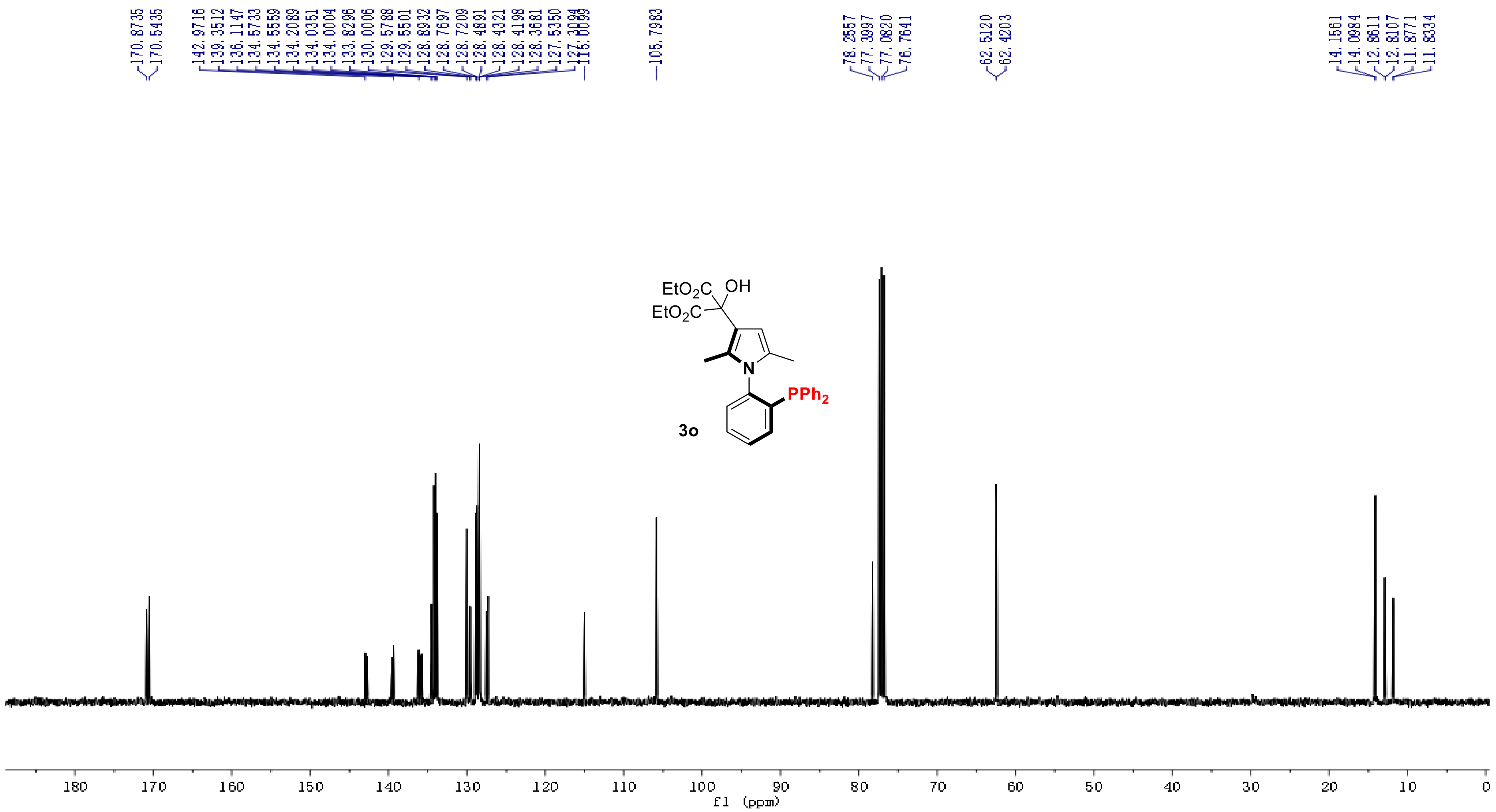

**Supplementary Figure 83.** $^{13}$C NMR of **3o**.

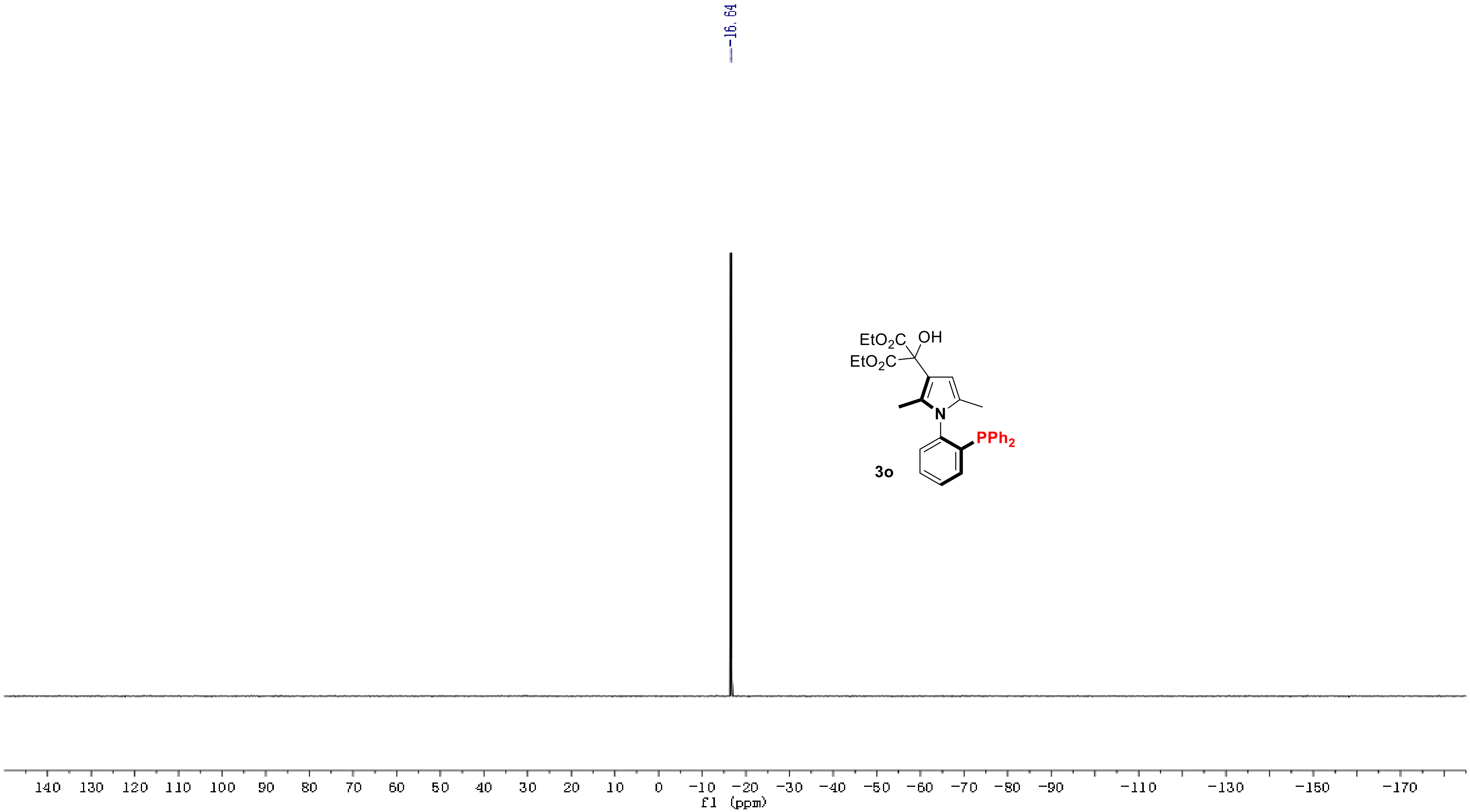


**Supplementary Figure 84.** $^{31}$P NMR of **3o**.

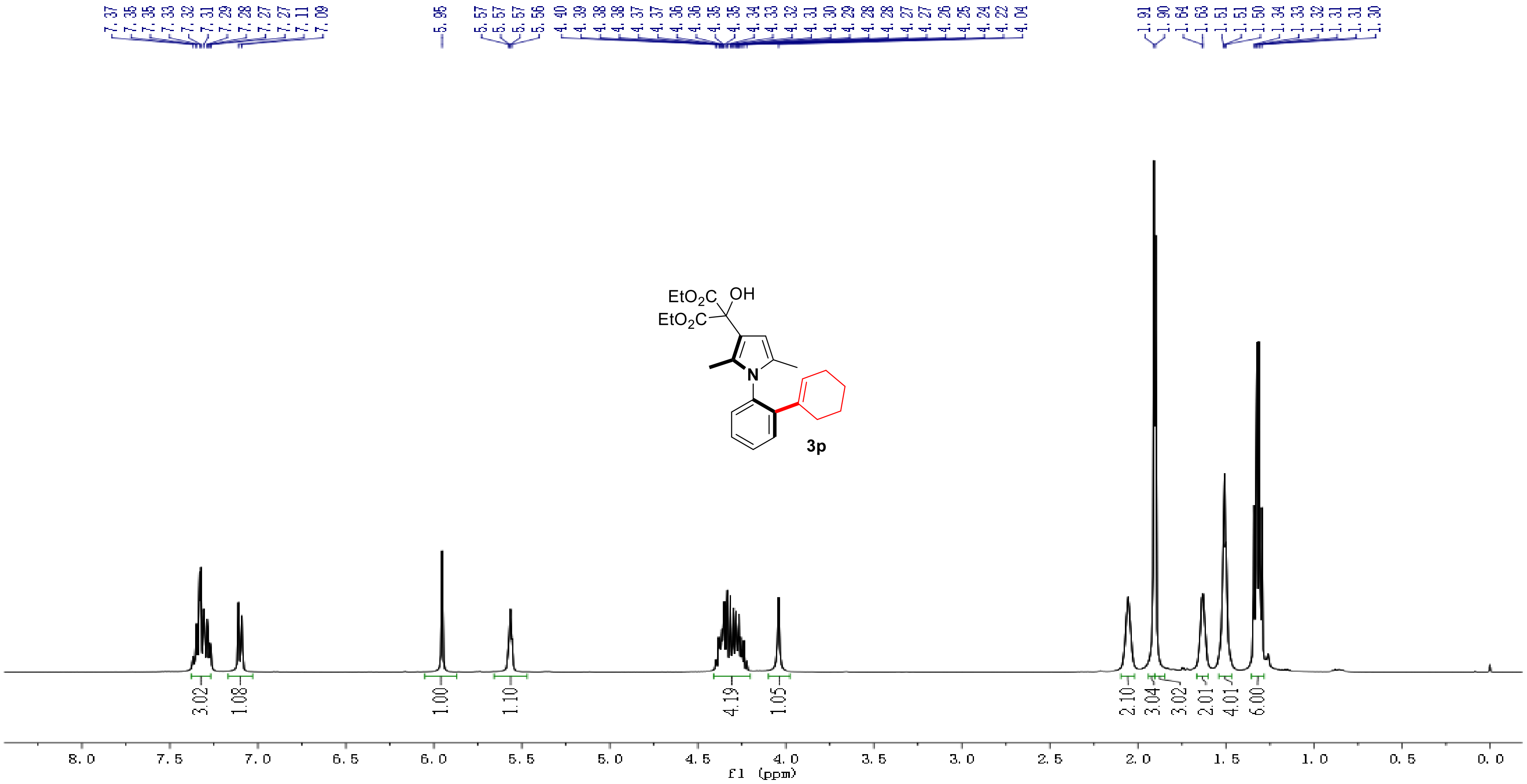

**Supplementary Figure 85.** $^1$H NMR of **3p**.

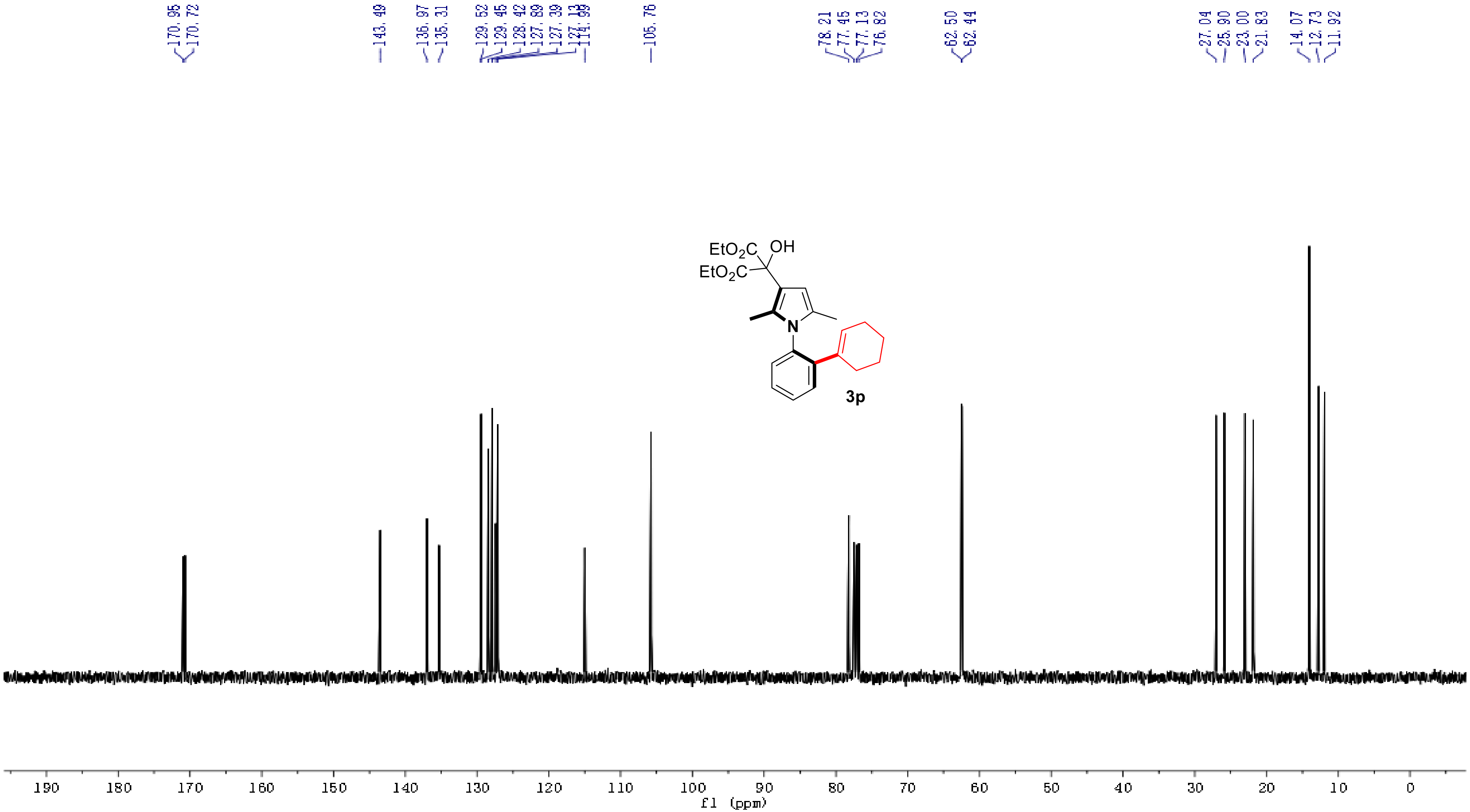


**Supplementary Figure 86.** $^{13}$C NMR of **3p**.

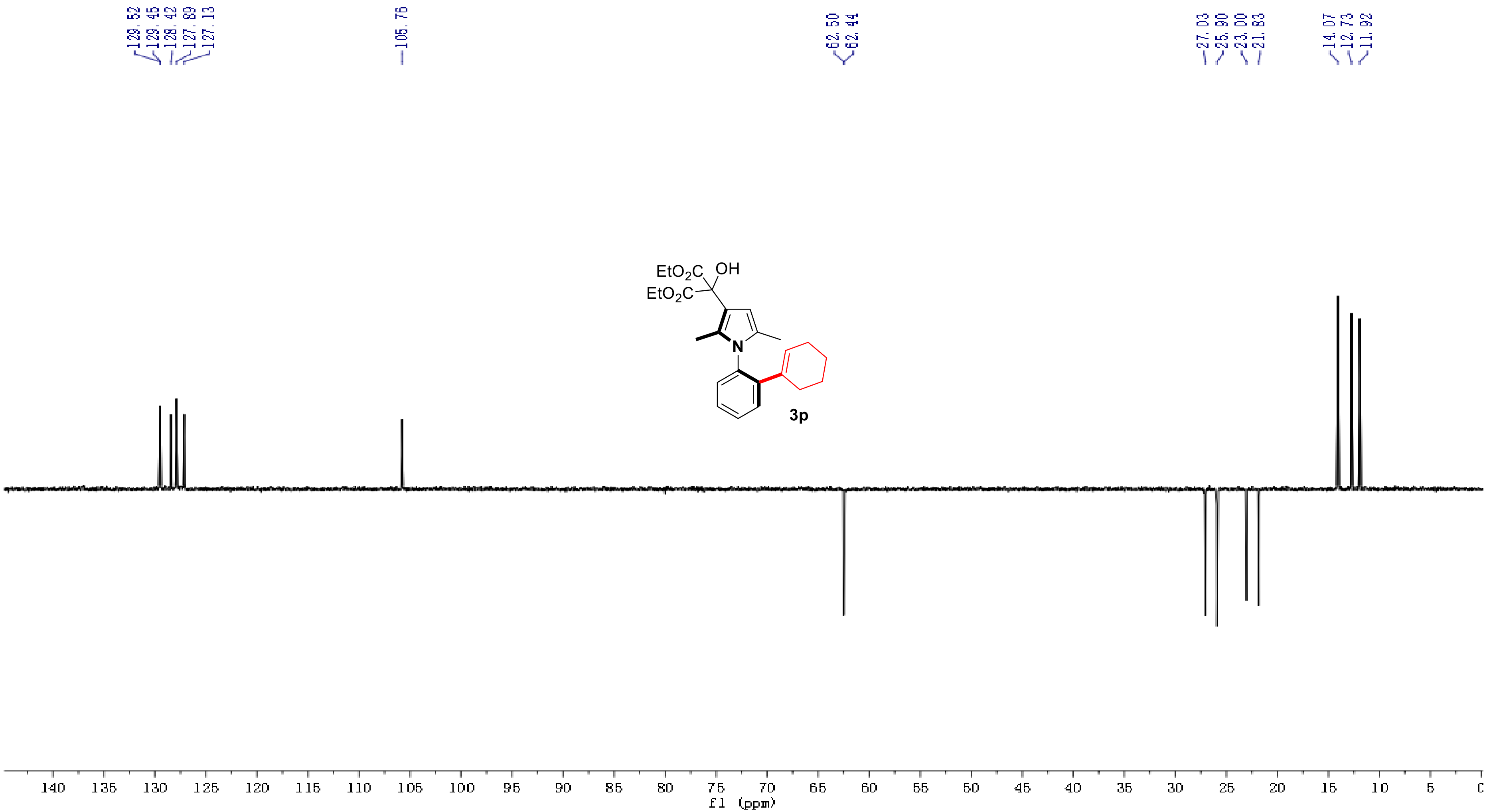


**Supplementary Figure 87.** $^{13}$C NMR-DEPT 135 of **3p**.

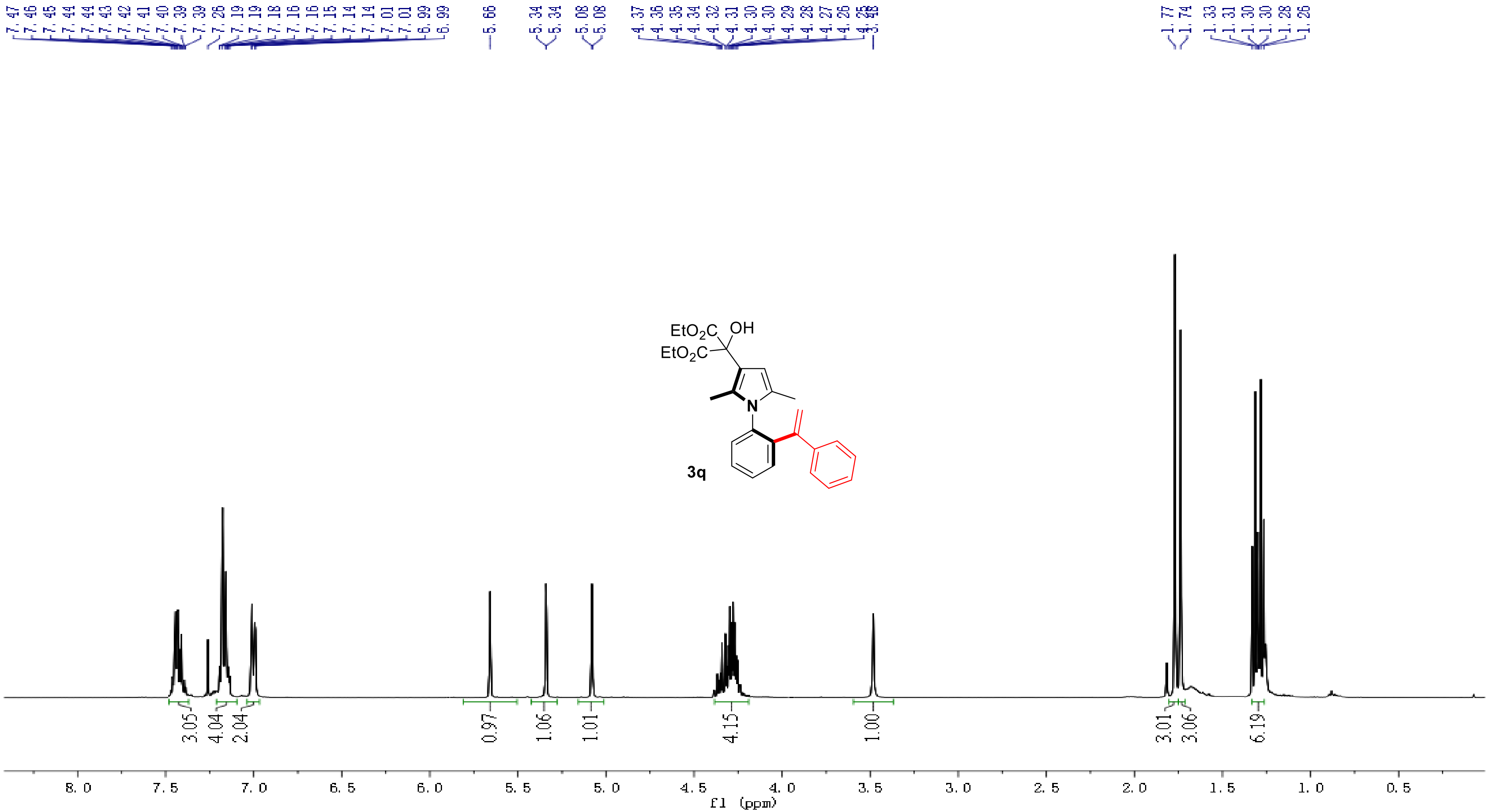


**Supplementary Figure 88.** $^{1}$H NMR of **3q**.

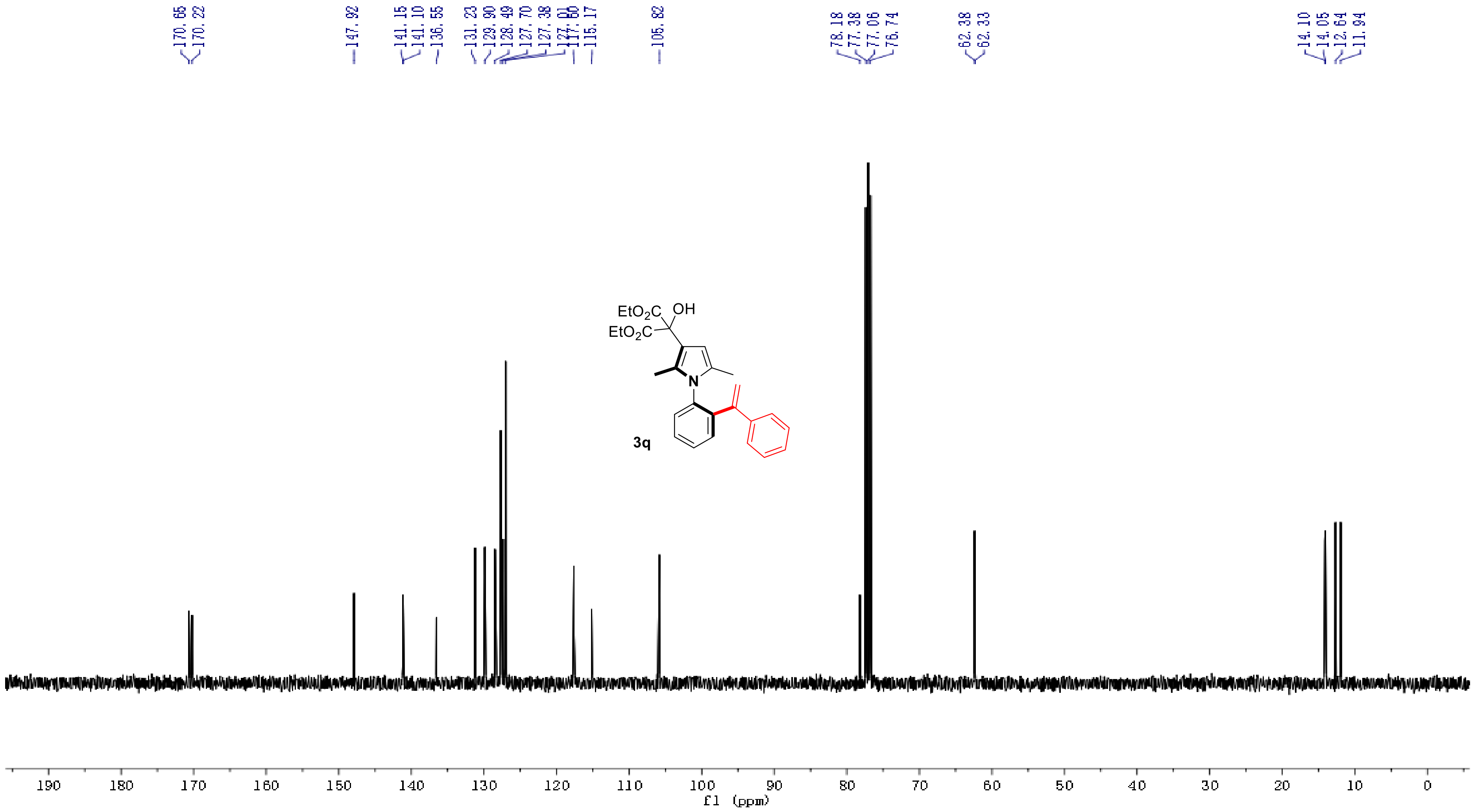


**Supplementary Figure 89.** $^{13}$C NMR of **3q**.

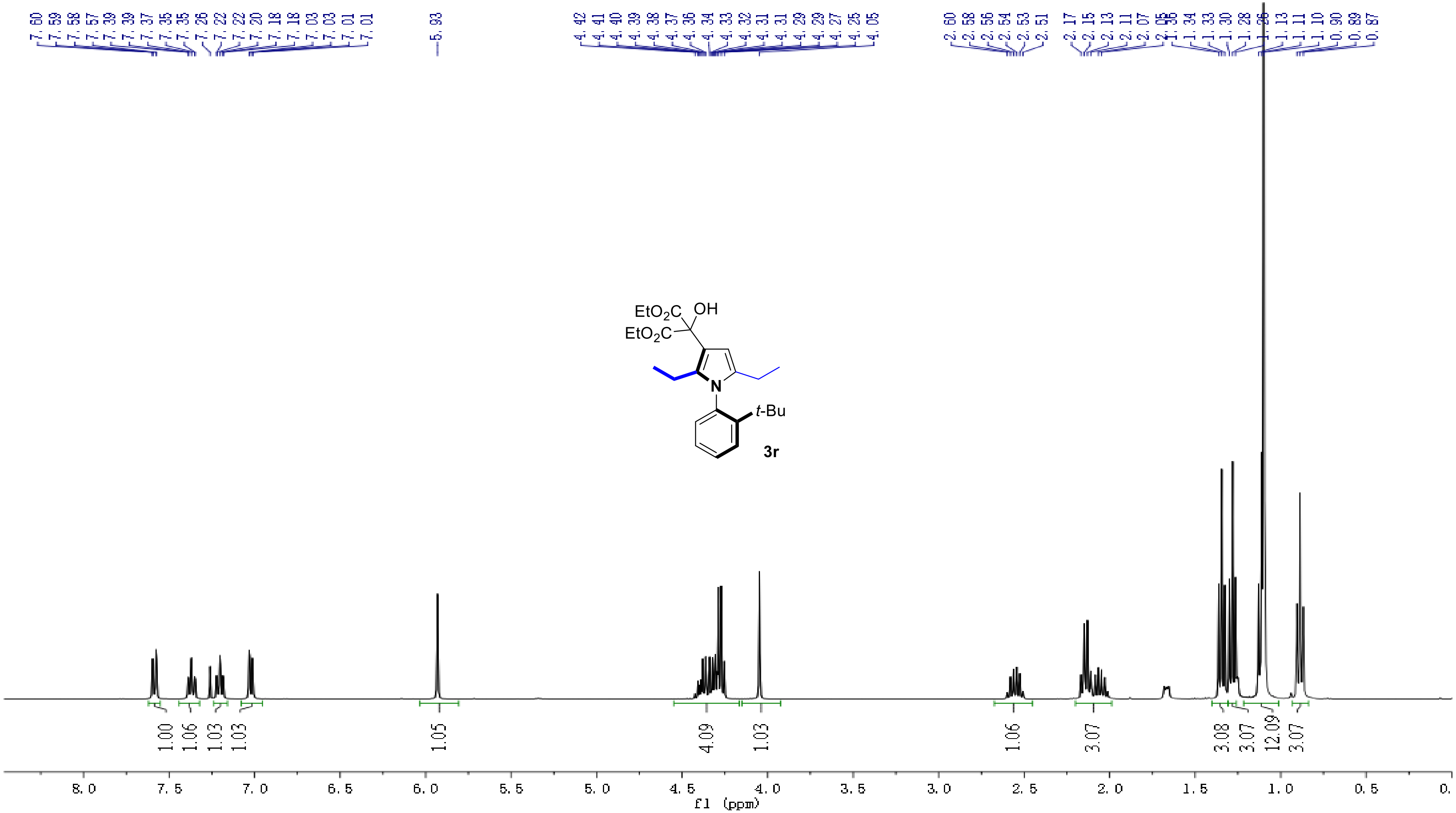


**Supplementary Figure 90.** $^{1}$H NMR of **3r**.

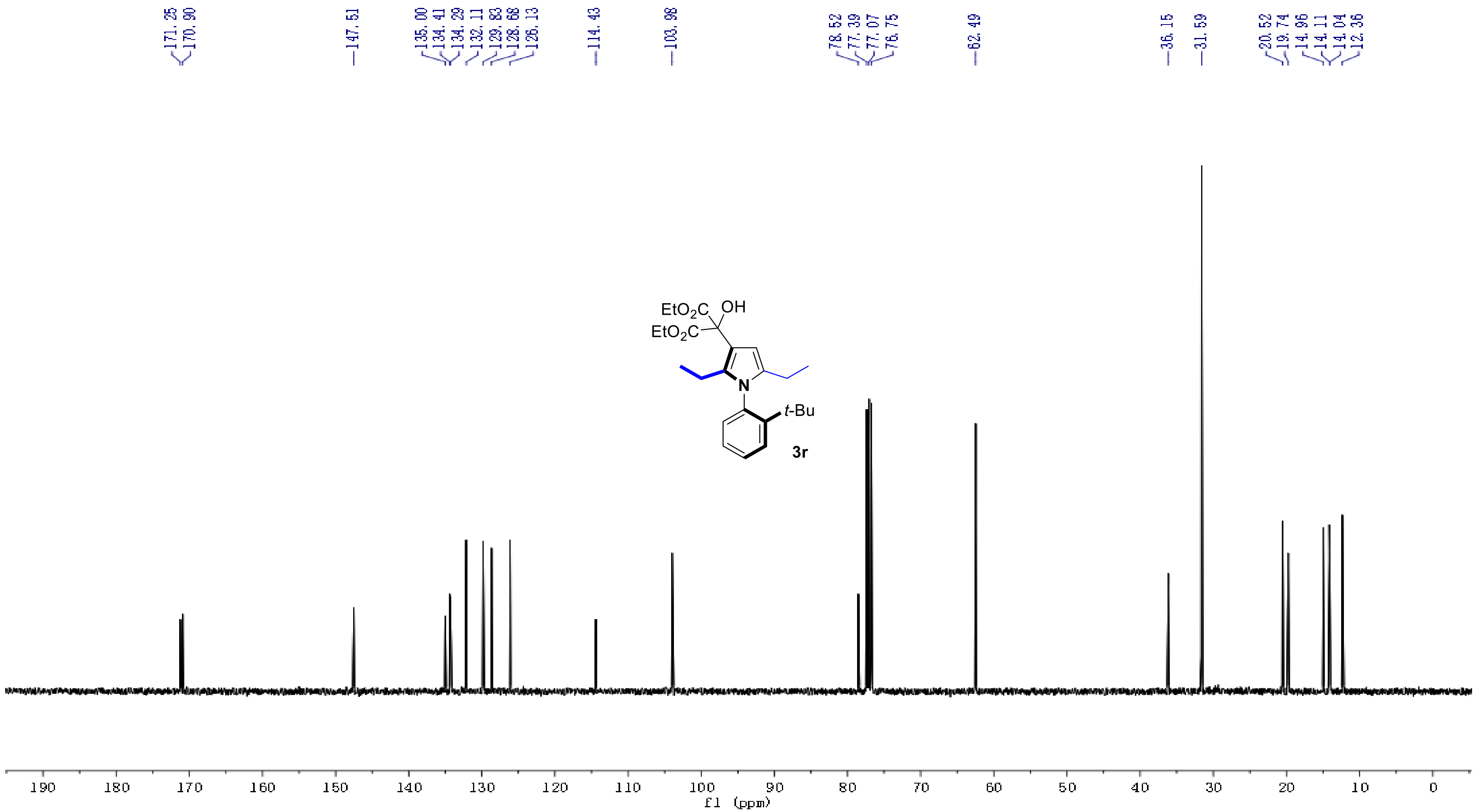


**Supplementary Figure 91.** $^{13}$C NMR of **3r**.

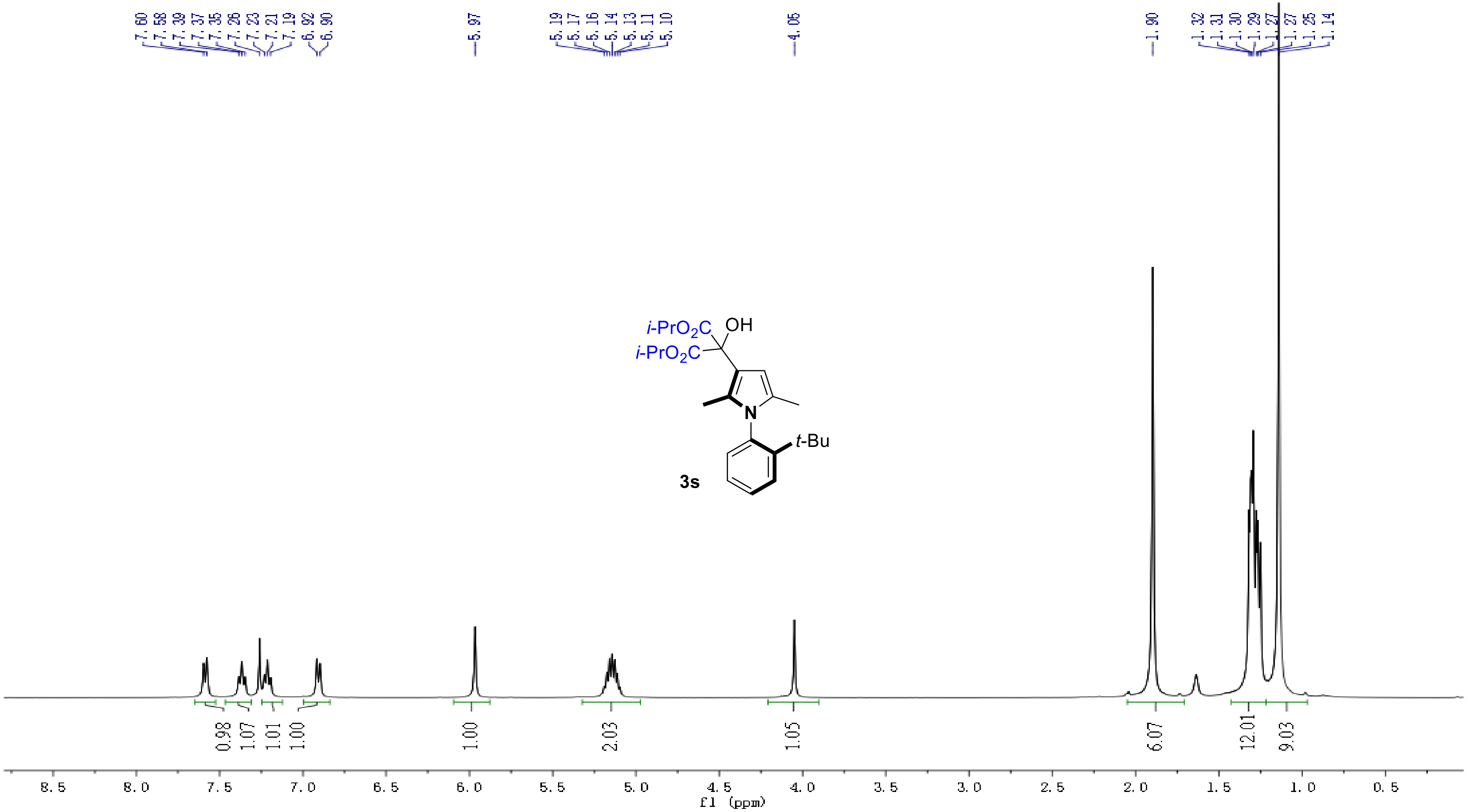


**Supplementary Figure 92.** $^1$H NMR of **3s**.

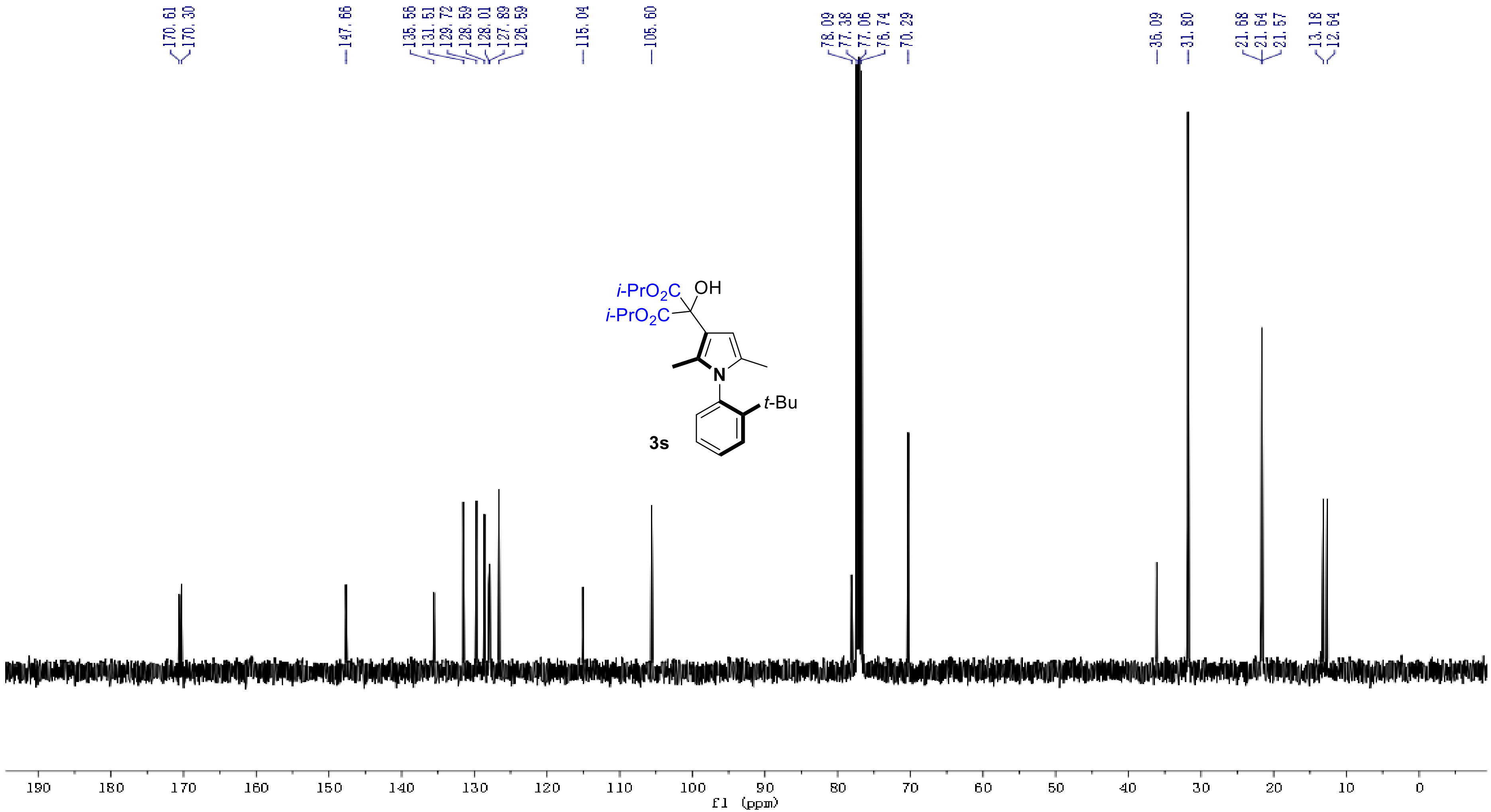


**Supplementary Figure 93.** $^{13}$C NMR of **3s**.

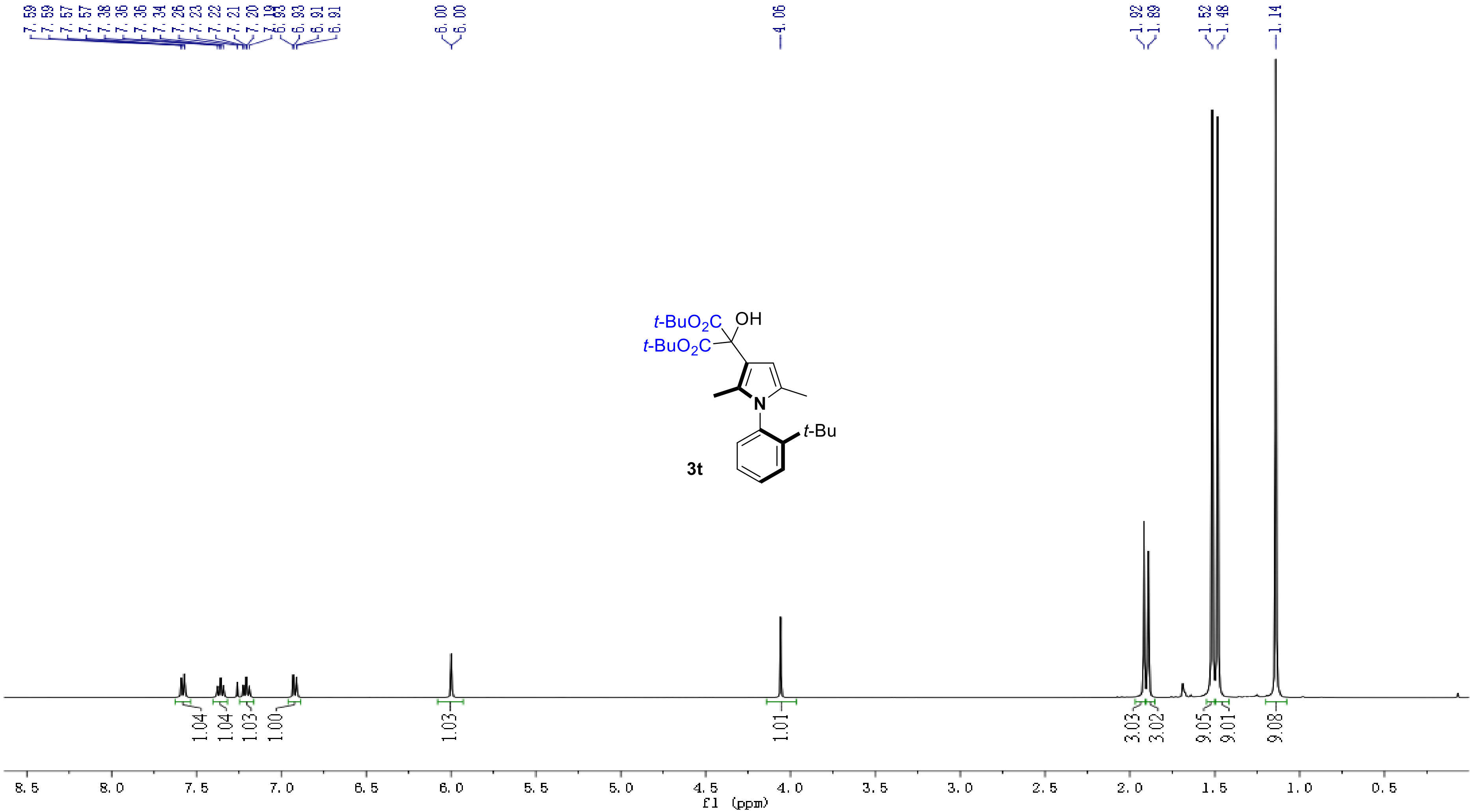


**Supplementary Figure 94.** $^{1}$H NMR of **3t**.

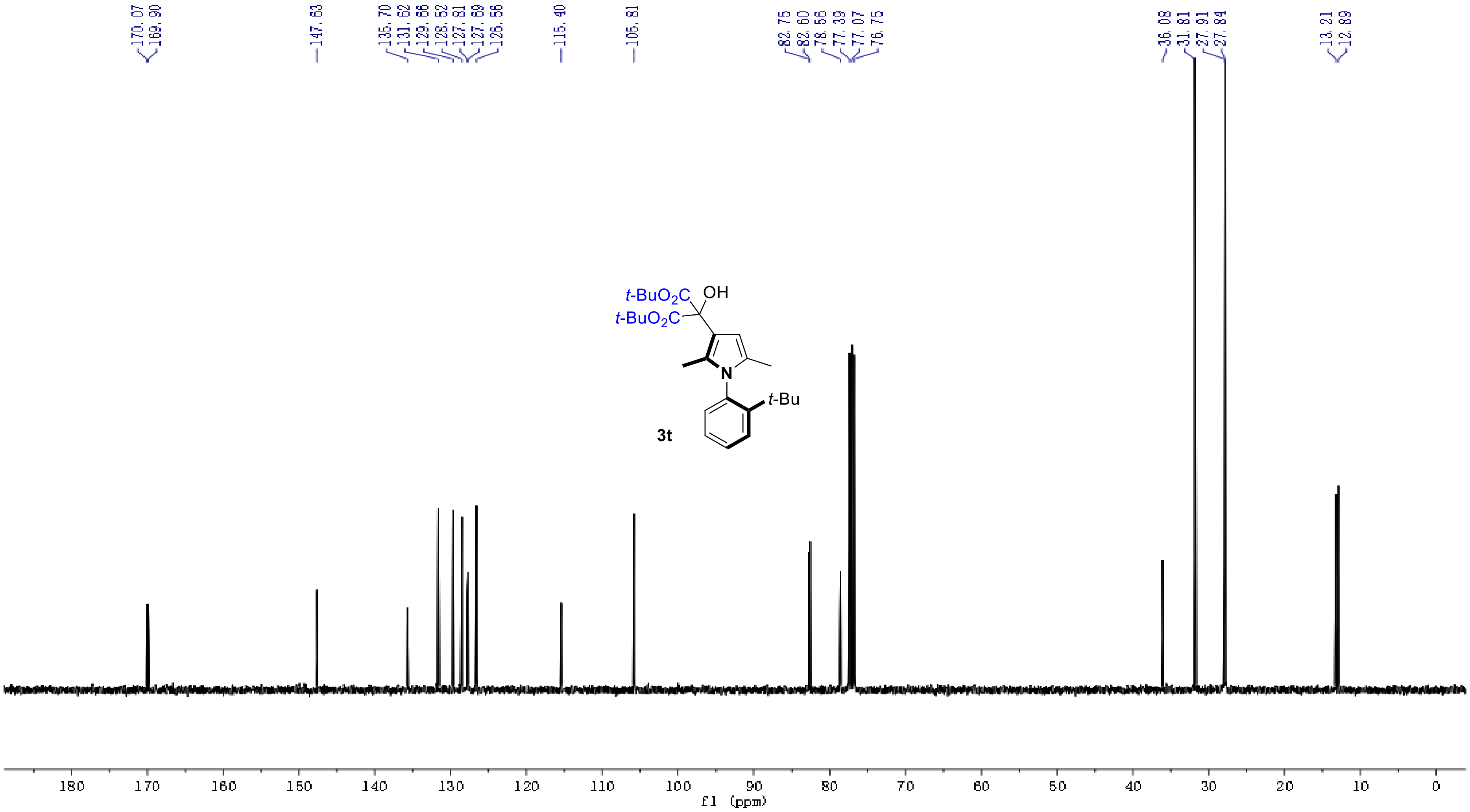


**Supplementary Figure 95.** $^{13}$C NMR of **3t**.

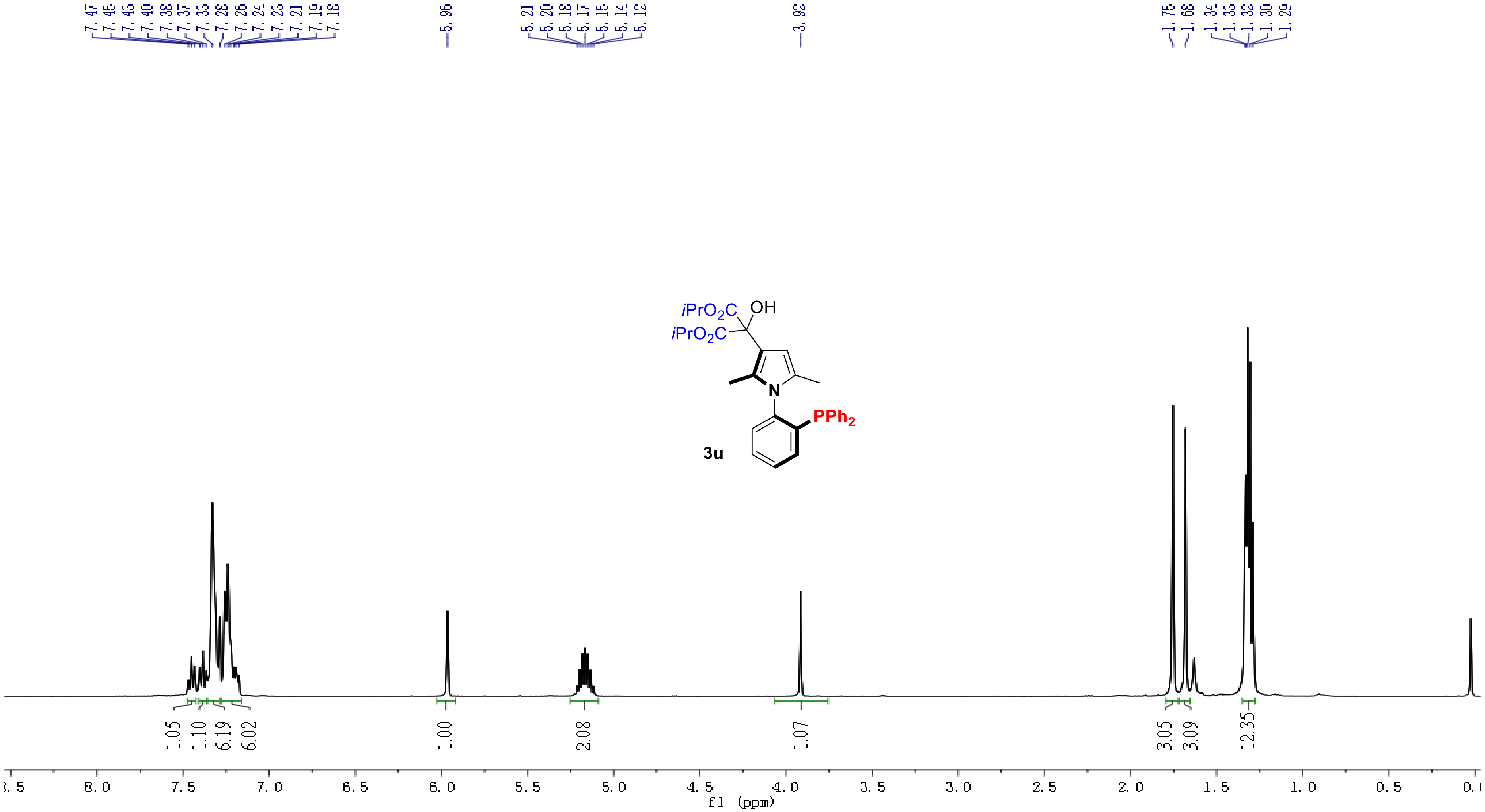


**Supplementary Figure 96.** $^1$H NMR of **3u**.

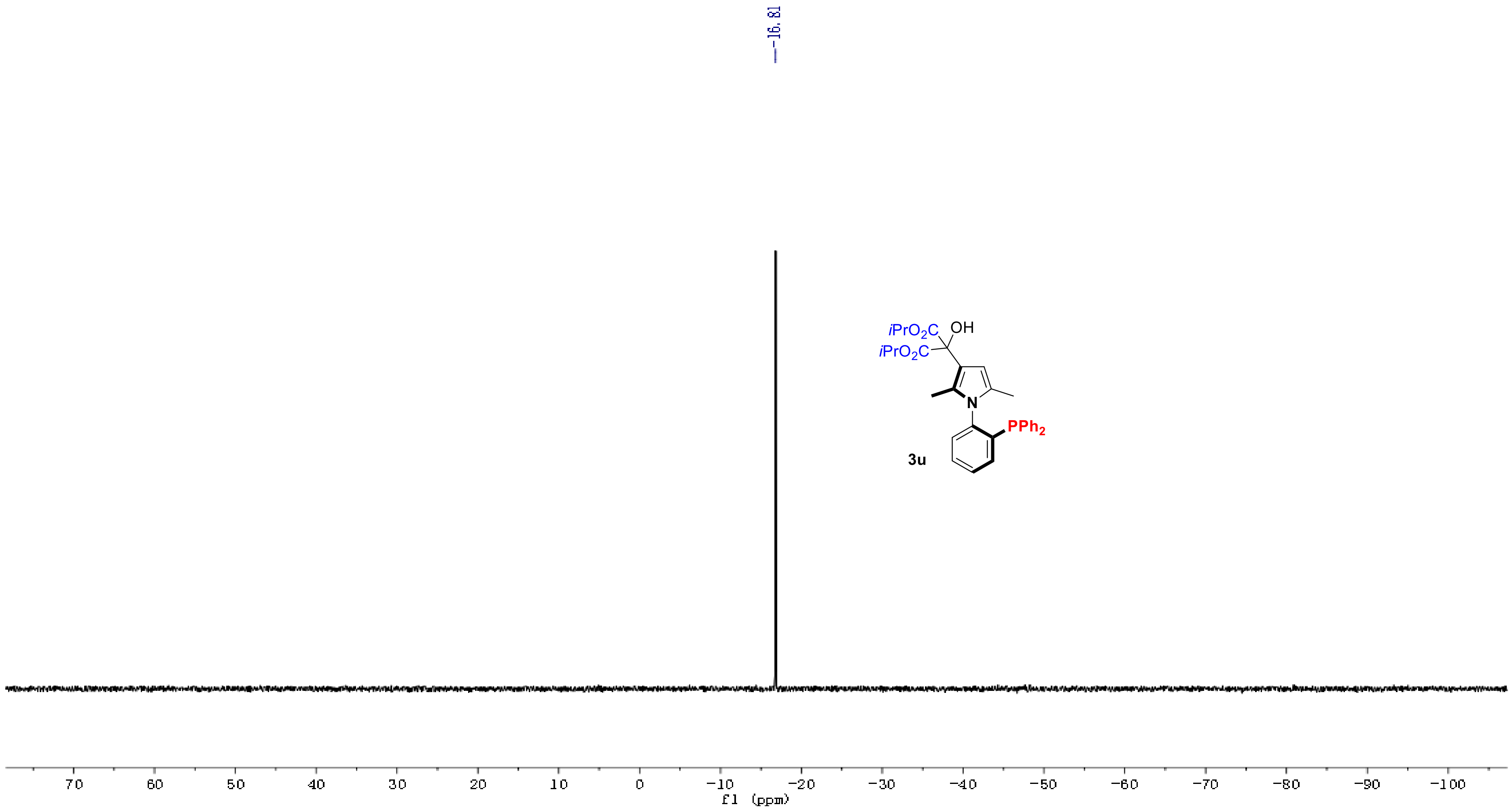


**Supplementary Figure 97.** $^{31}$P NMR of **3u**.

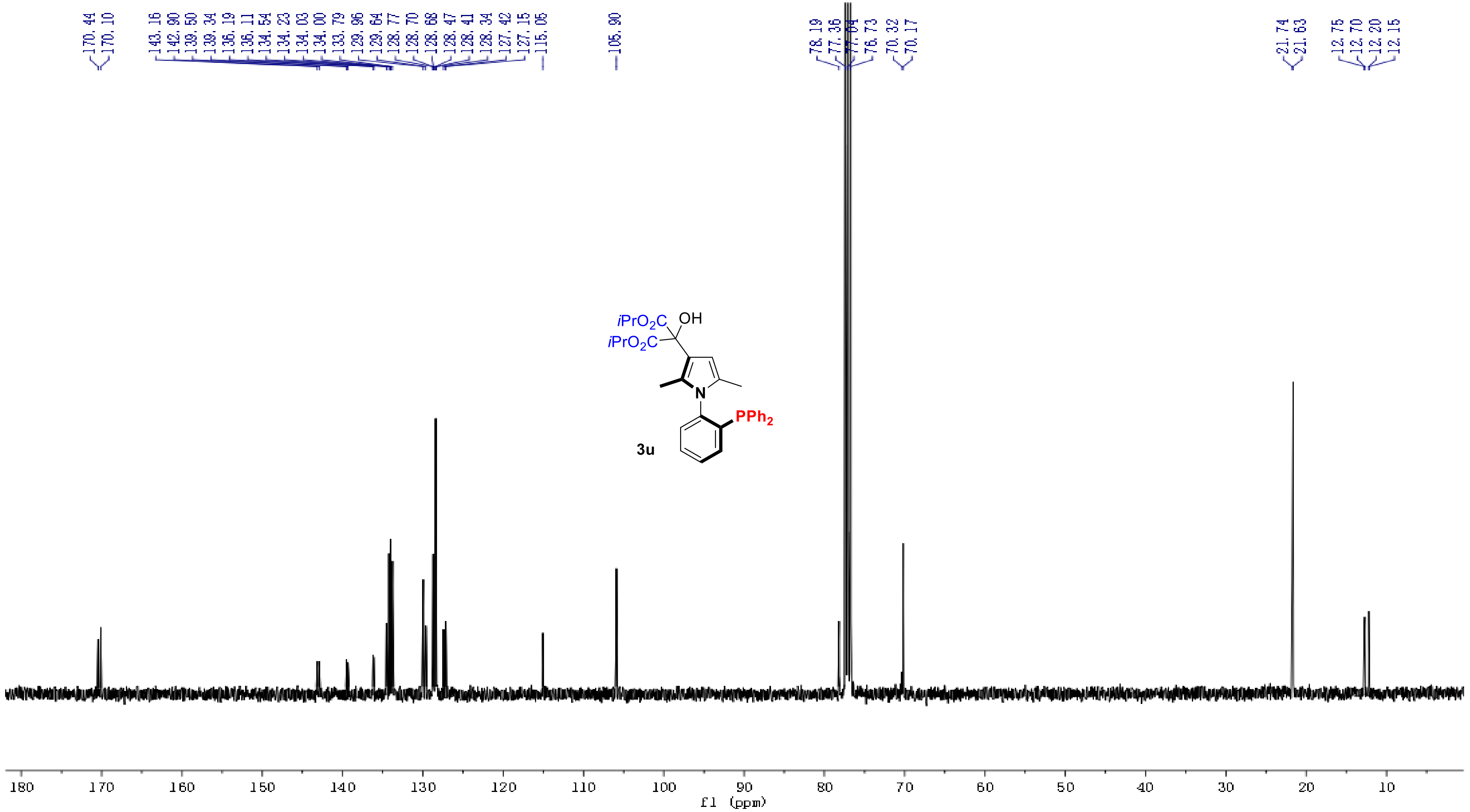


**Supplementary Figure 98.** $^{13}$C NMR of **3u**.

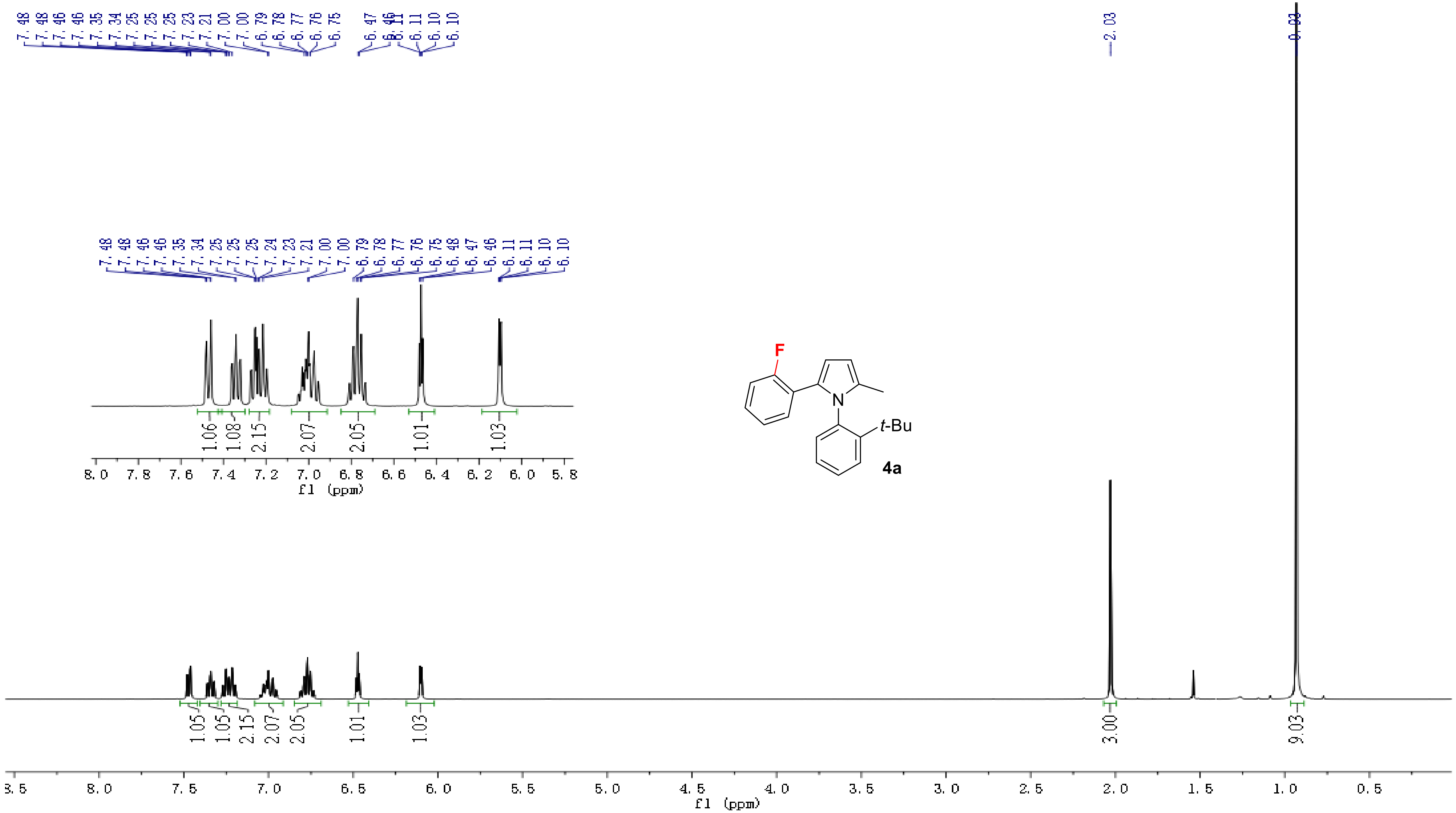


**Supplementary Figure 99.** $^{1}$H NMR of **4a**.

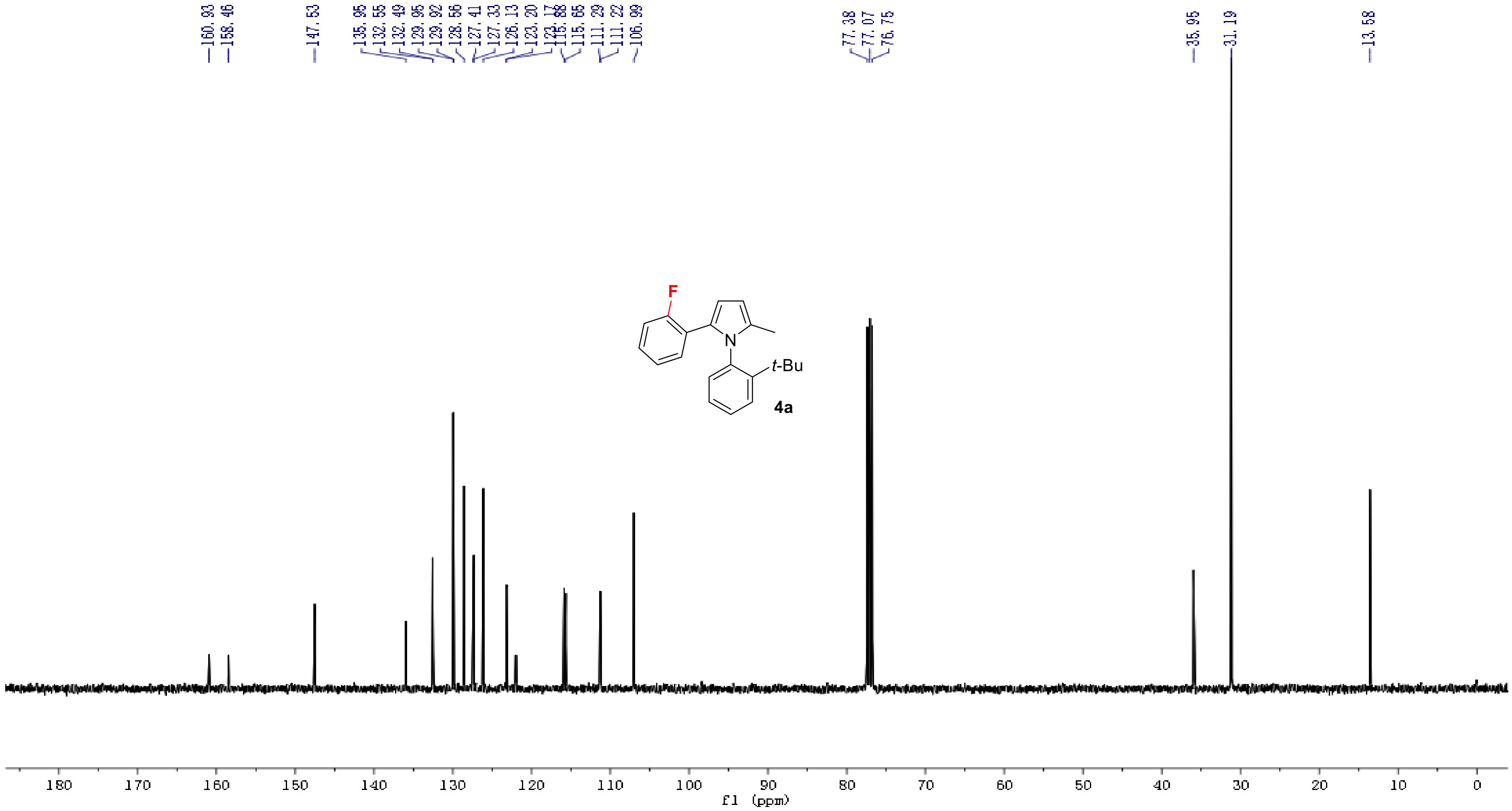


**Supplementary Figure 100.** $^{13}$C NMR of **4a**.

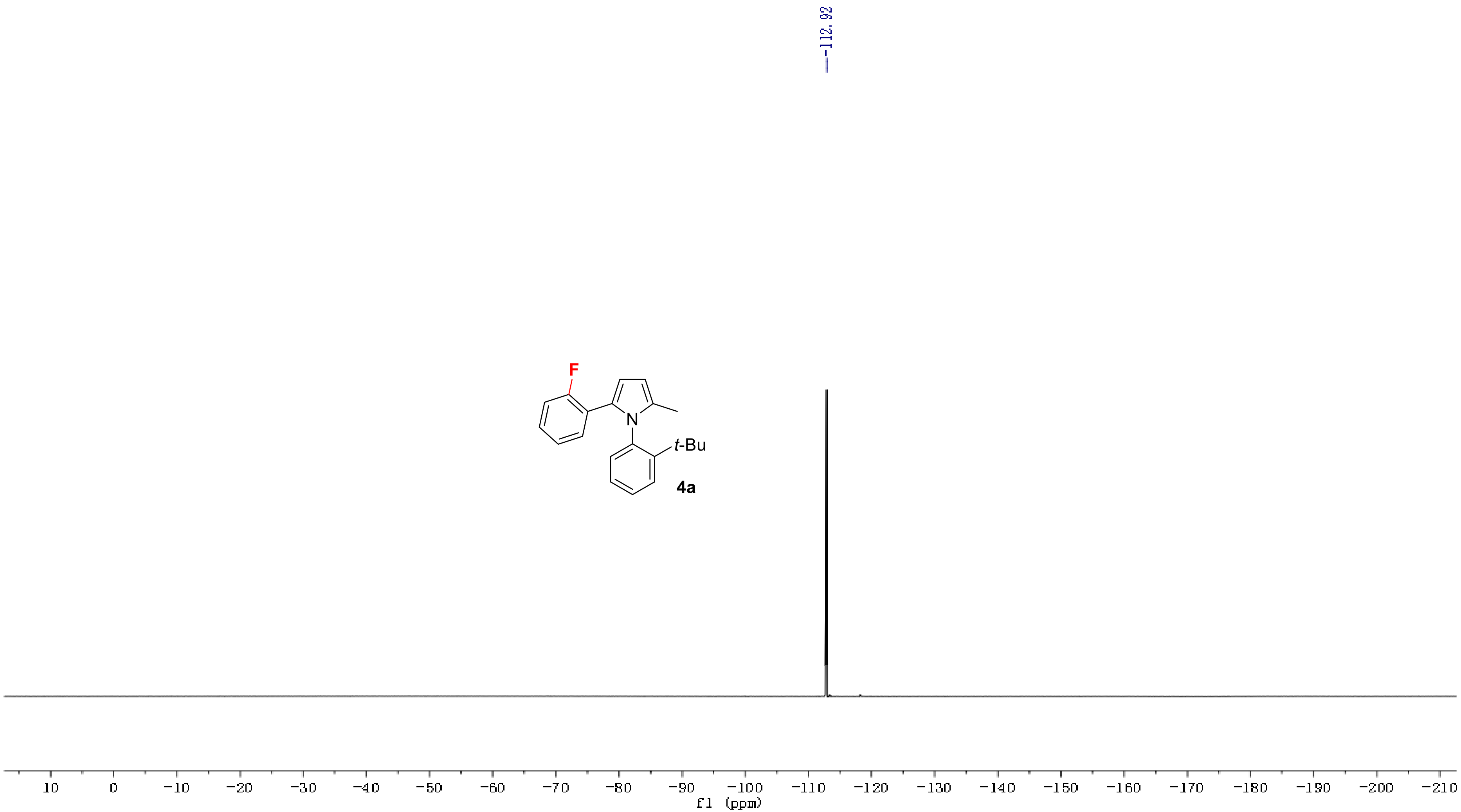


**Supplementary Figure 101.** $^{19}$F NMR of **4a**.

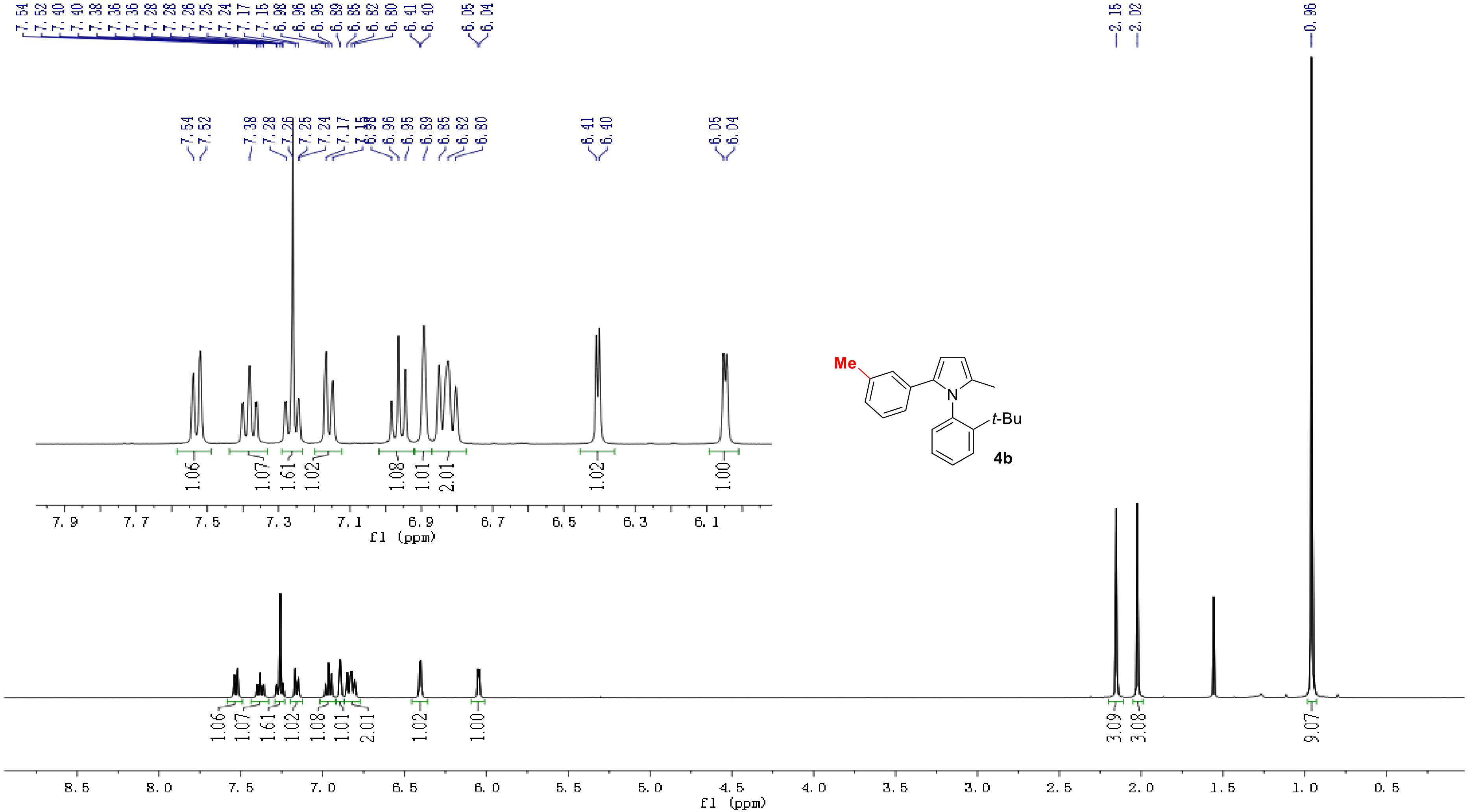


**Supplementary Figure 102.** $^{1}$H NMR of **4b**.

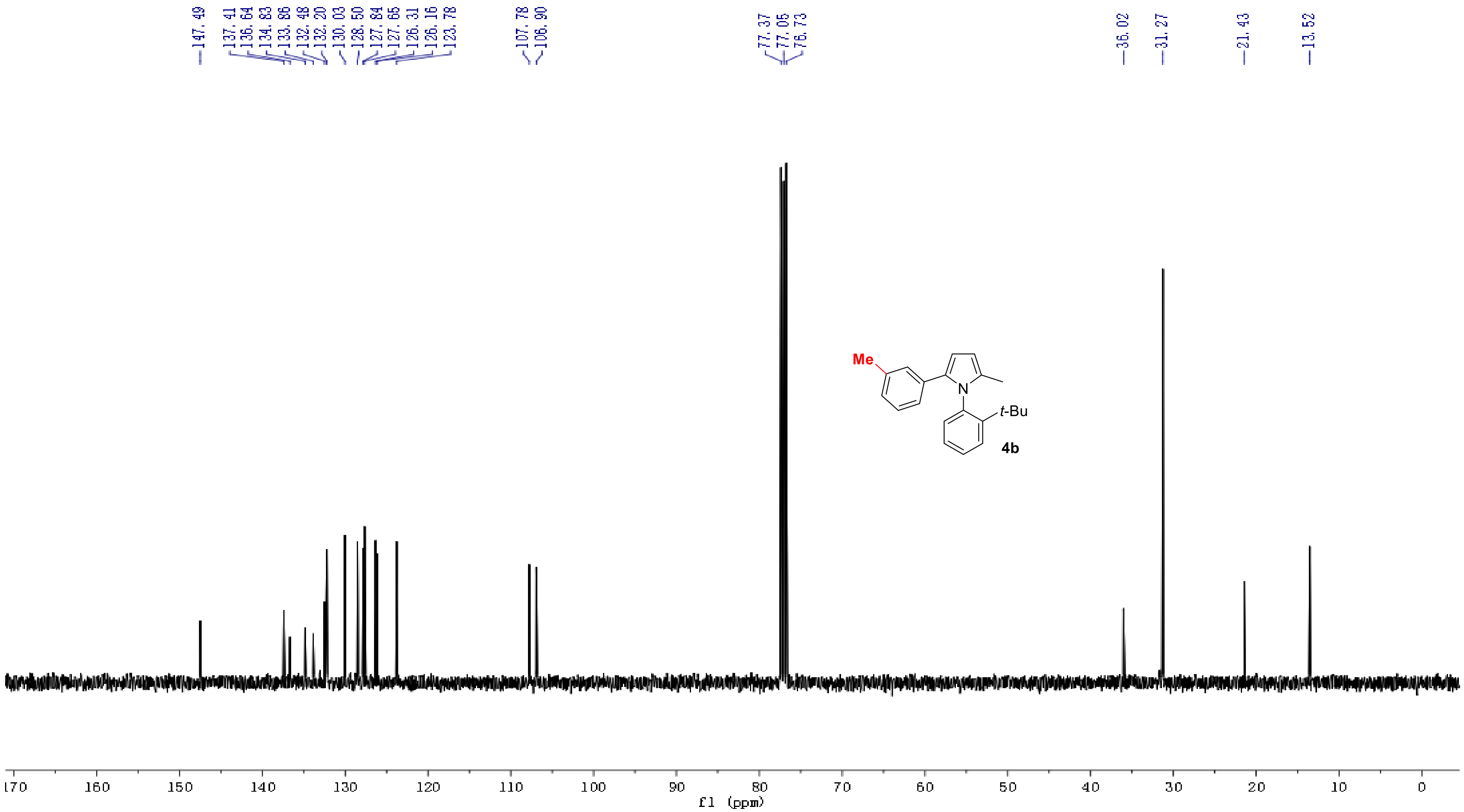


**Supplementary Figure 103.** $^{13}$C NMR of **4b**.

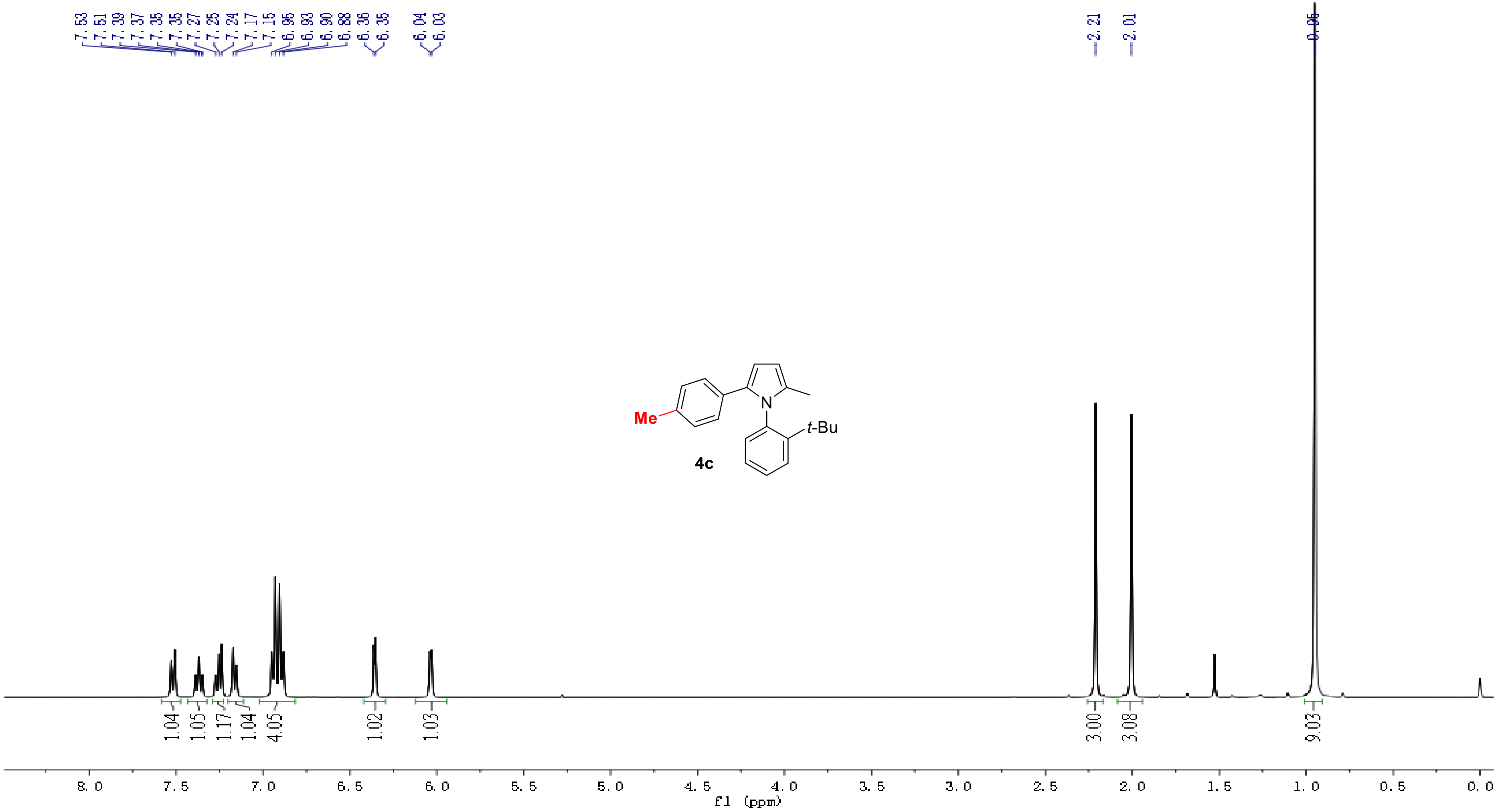


**Supplementary Figure 104.** $^{1}H$ NMR of **4c**.

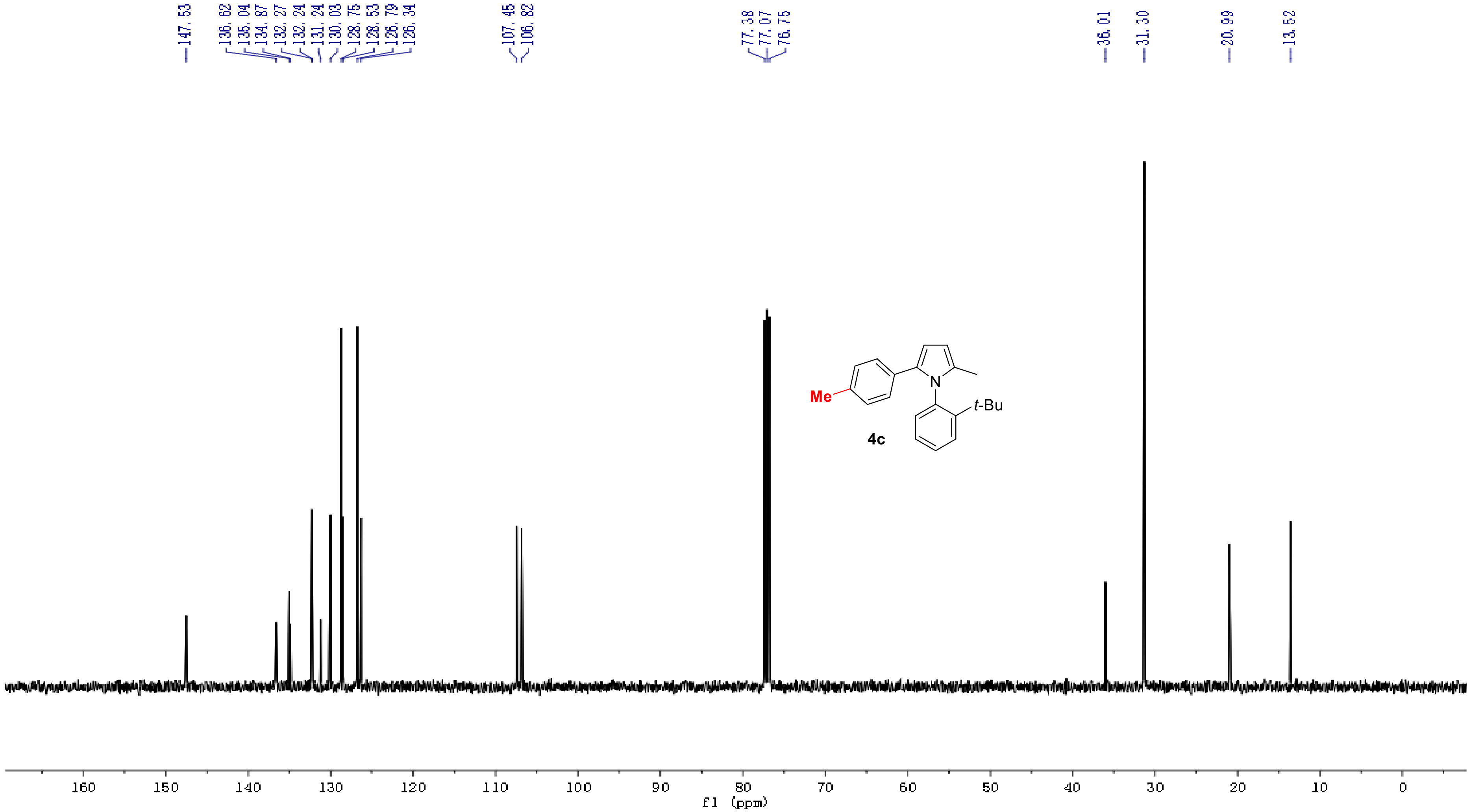


**Supplementary Figure 105.** $^{13}$C NMR of **4c**.

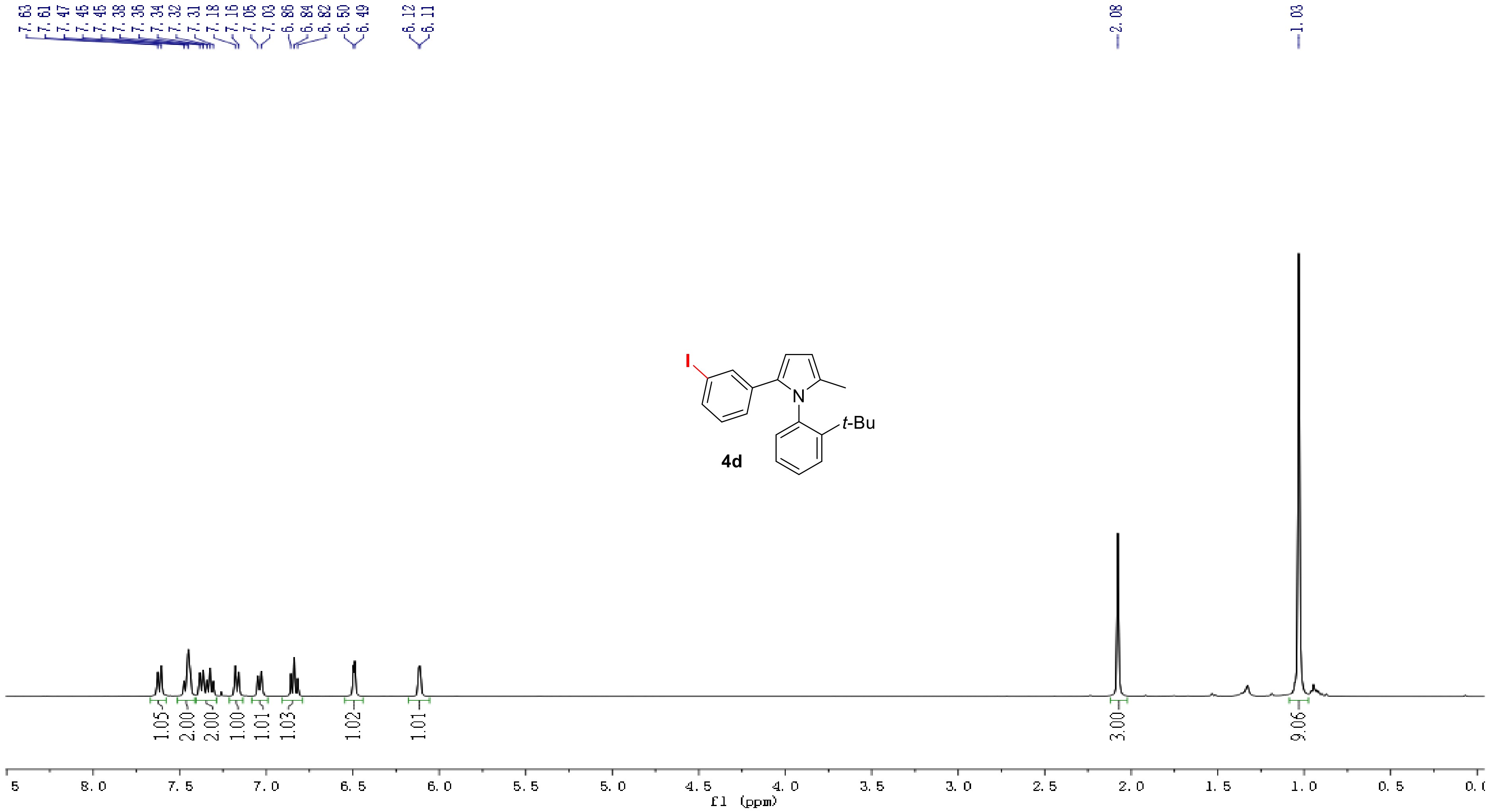

**Supplementary Figure 106.** $^{1}$H NMR of **4d**.

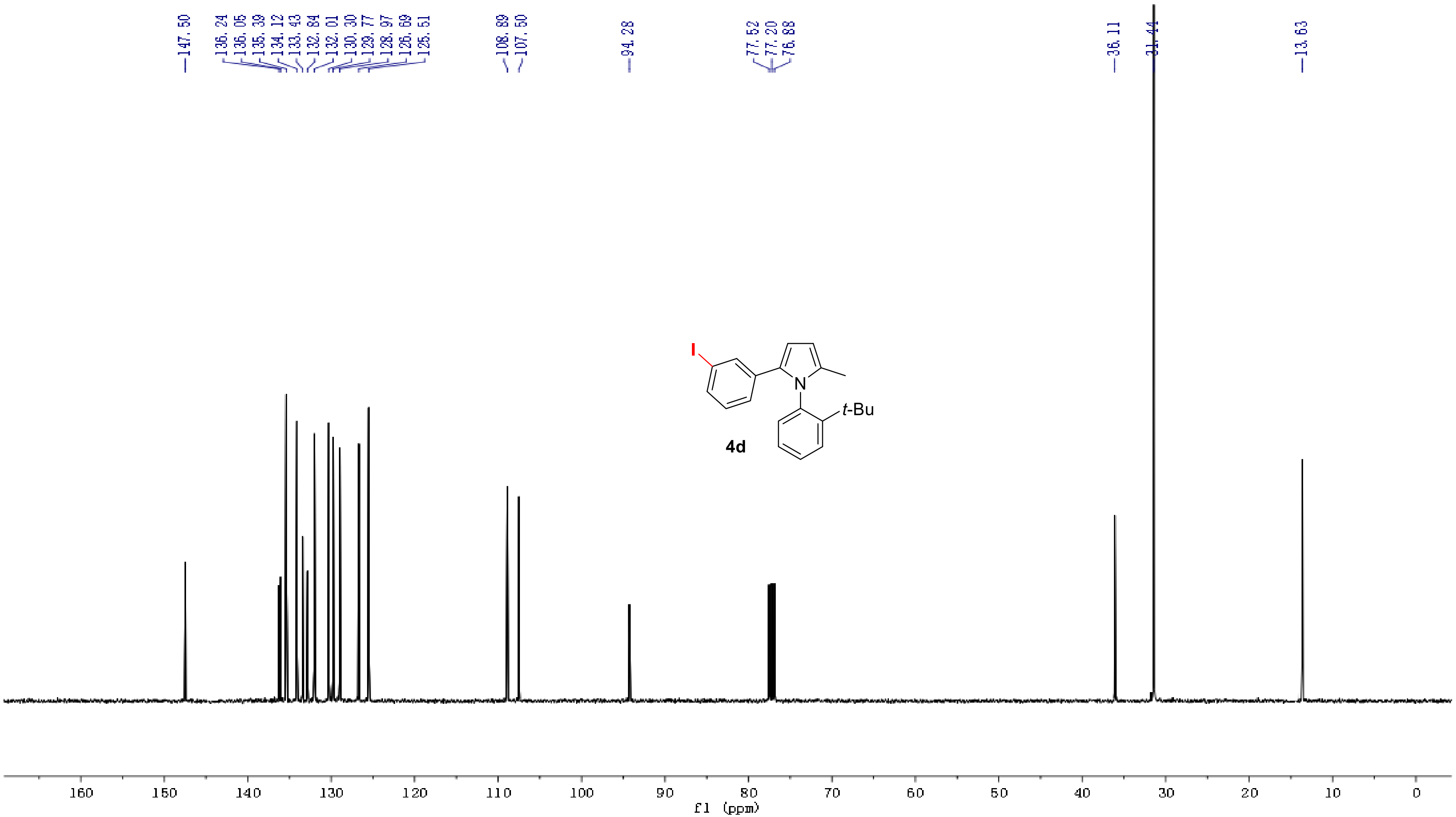


**Supplementary Figure 107.** $^{13}$C NMR of **4d**.

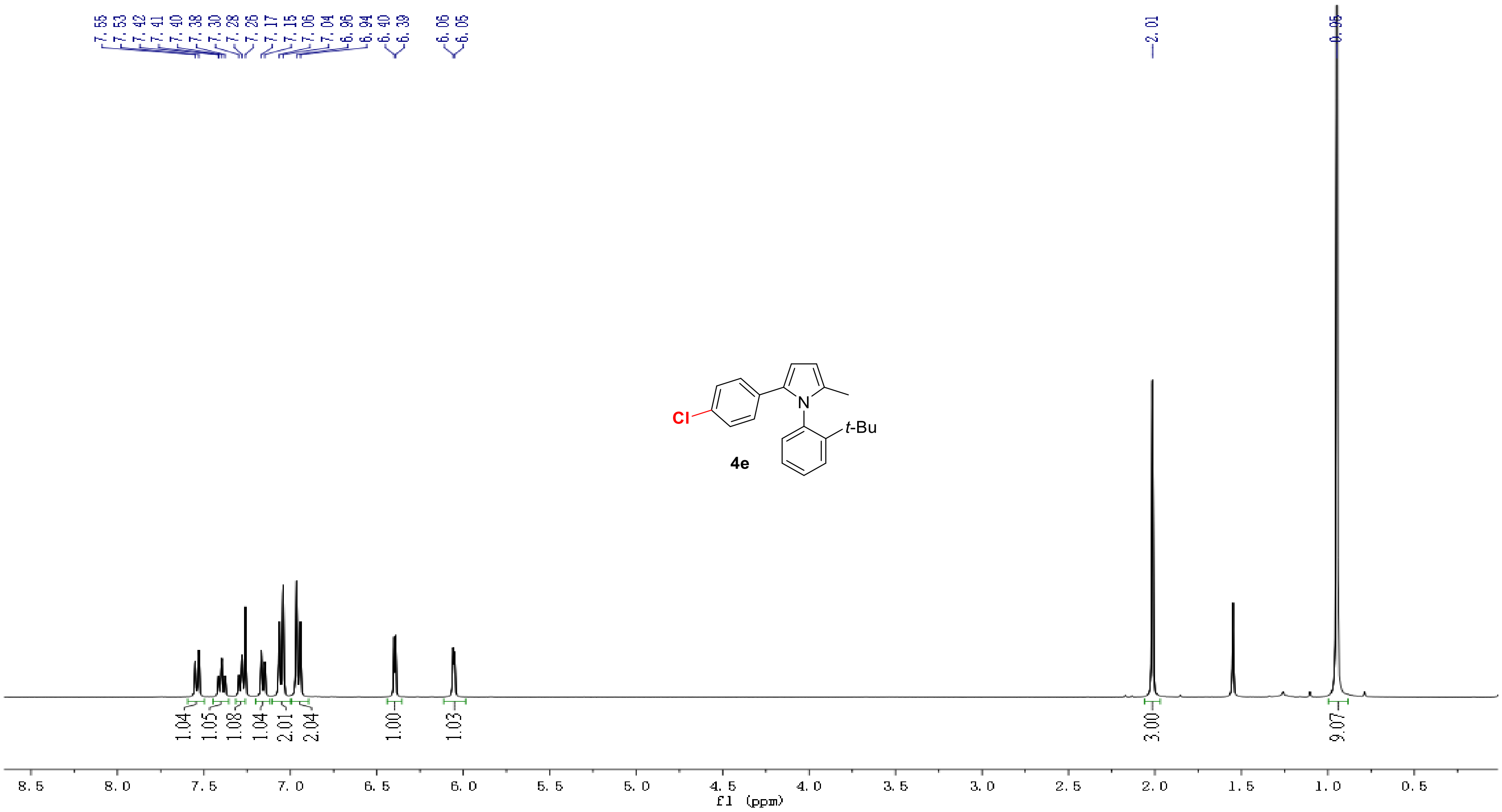


**Supplementary Figure 108.** $^{1}$H NMR of **4e**.

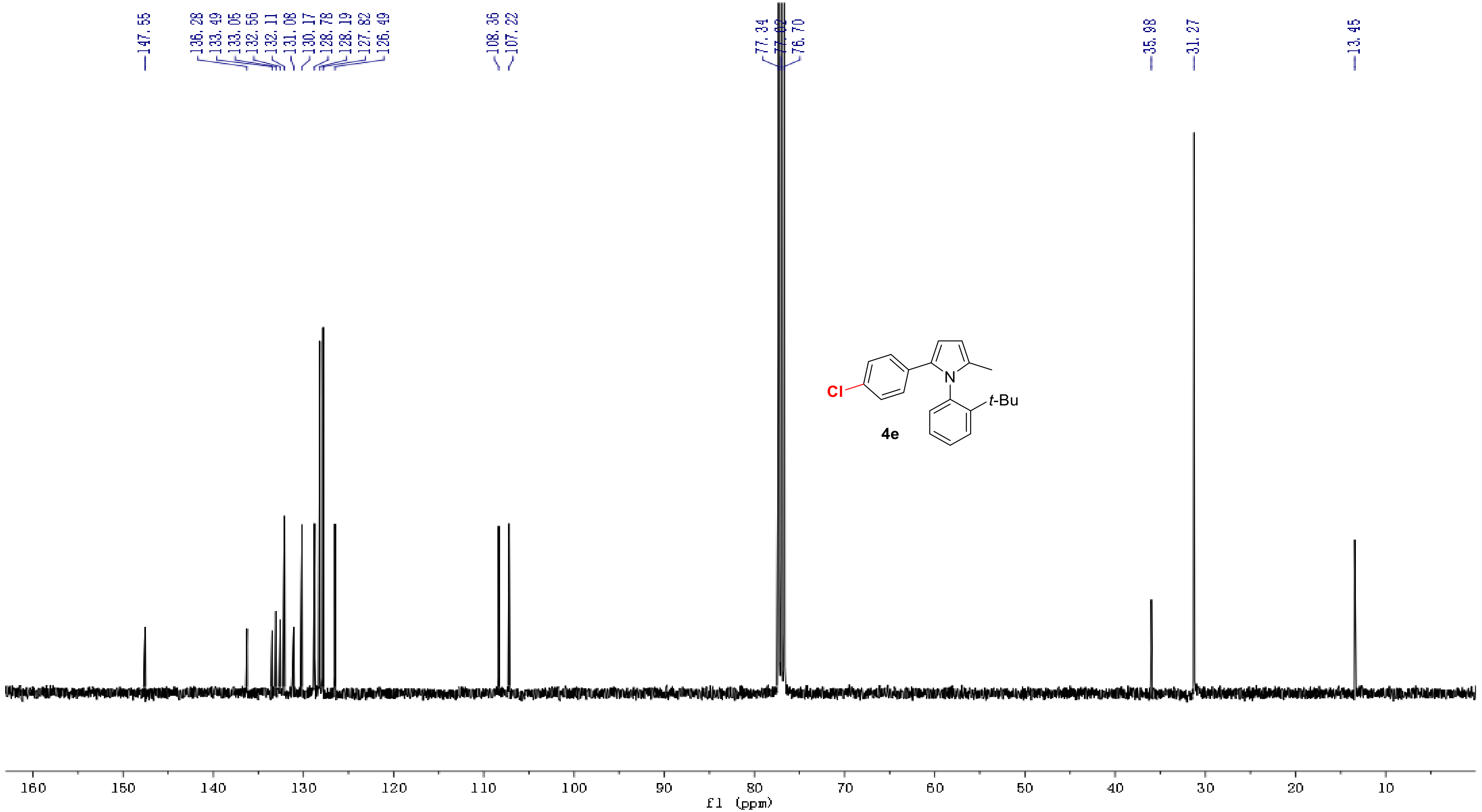


**Supplementary Figure 109.** $^{13}$C NMR of **4e**.

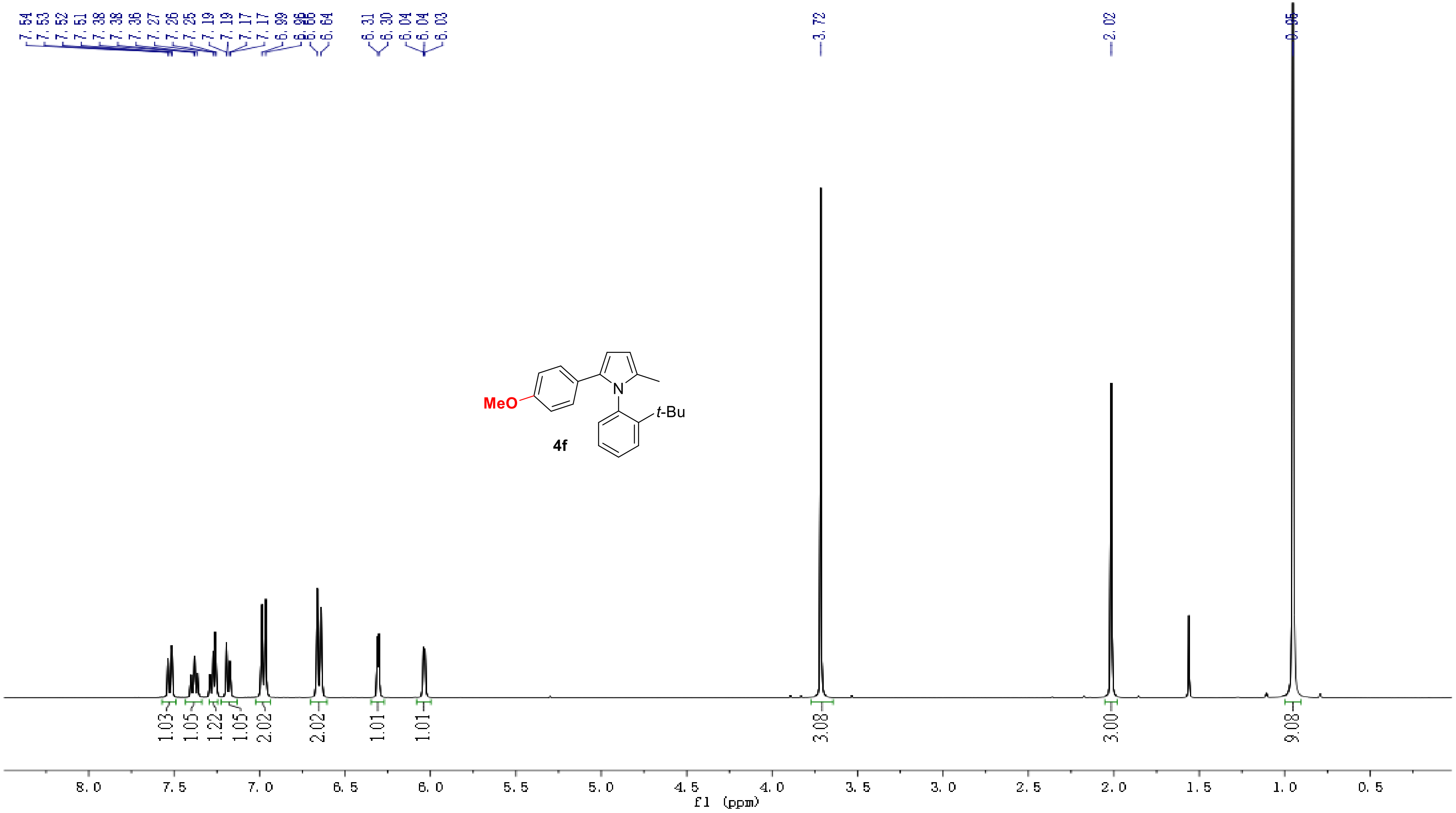


**Supplementary Figure 110.** $^1$H NMR of **4f**.

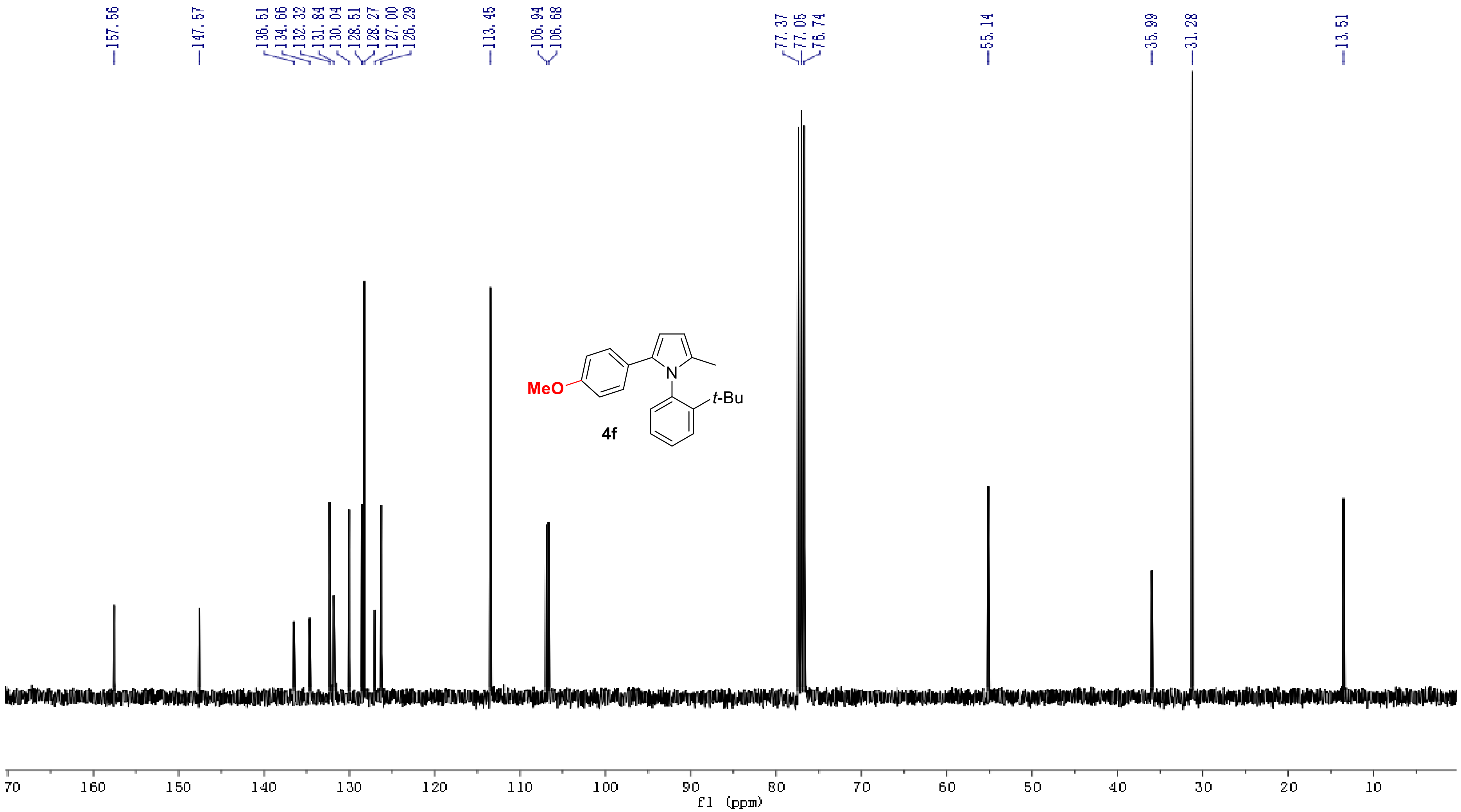


**Supplementary Figure 111.** $^{13}$C NMR of **4f**.

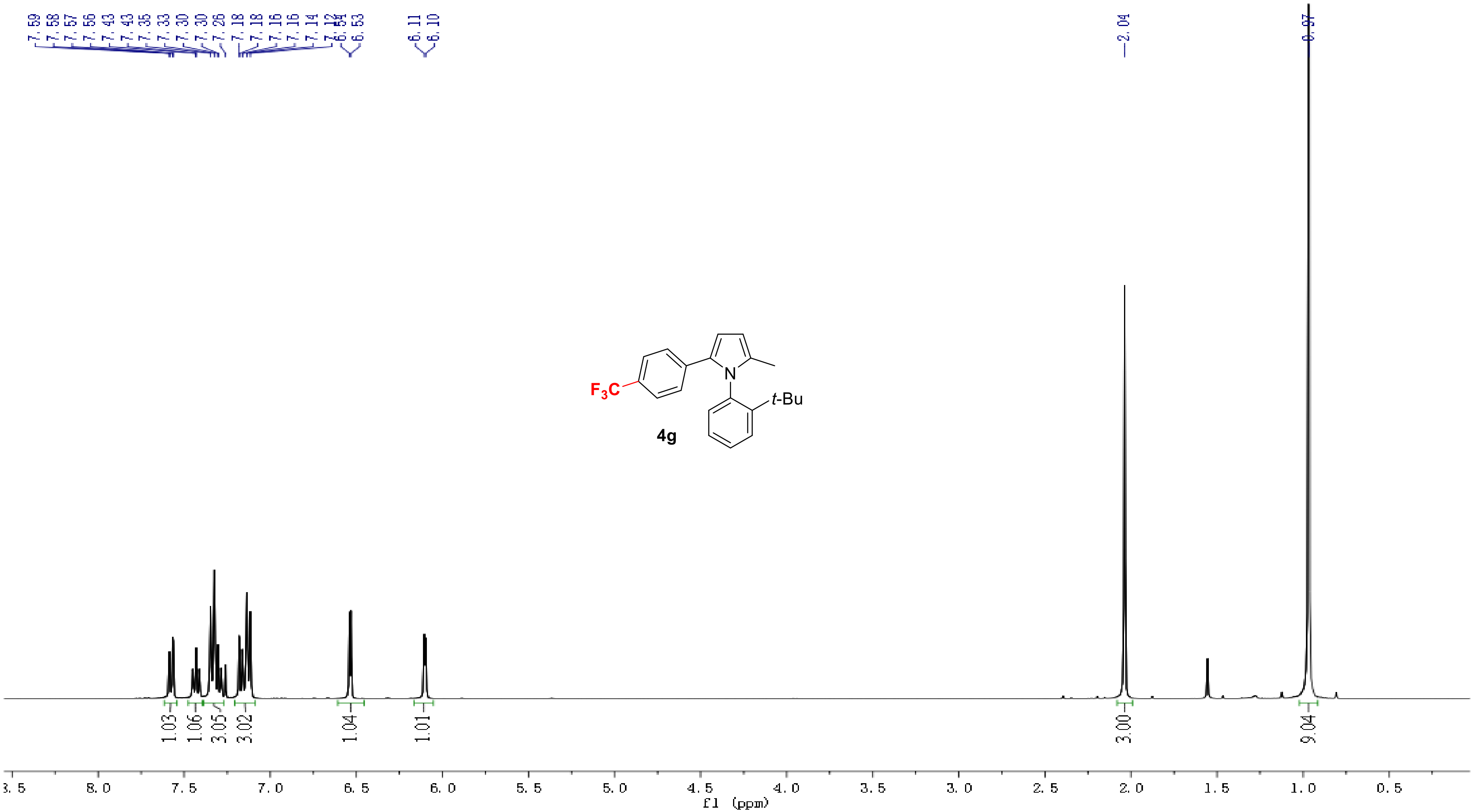


**Supplementary Figure 112.** $^{1}$H NMR of **4g**.

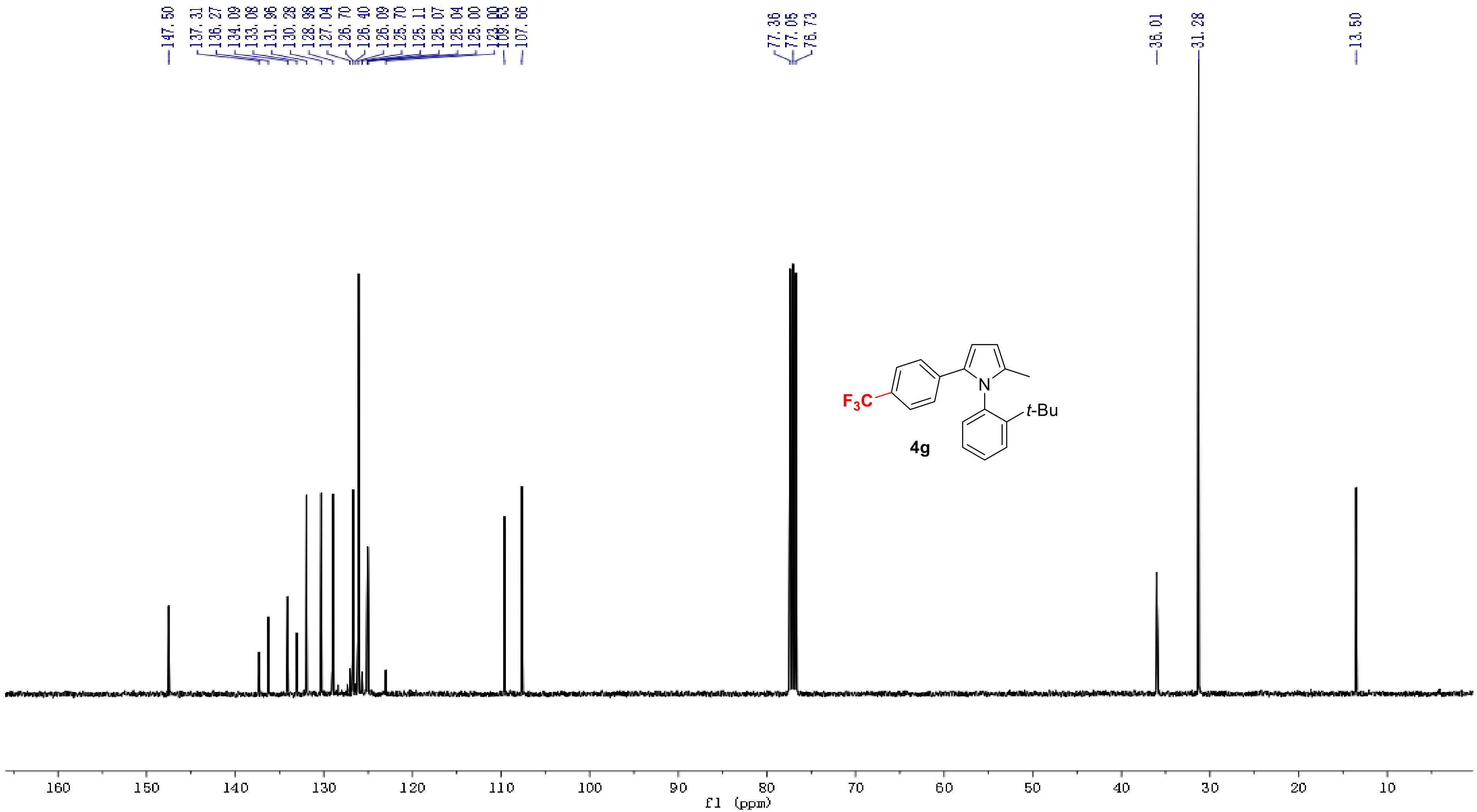


**Supplementary Figure 113.** $^{13}$C NMR of **4g**.

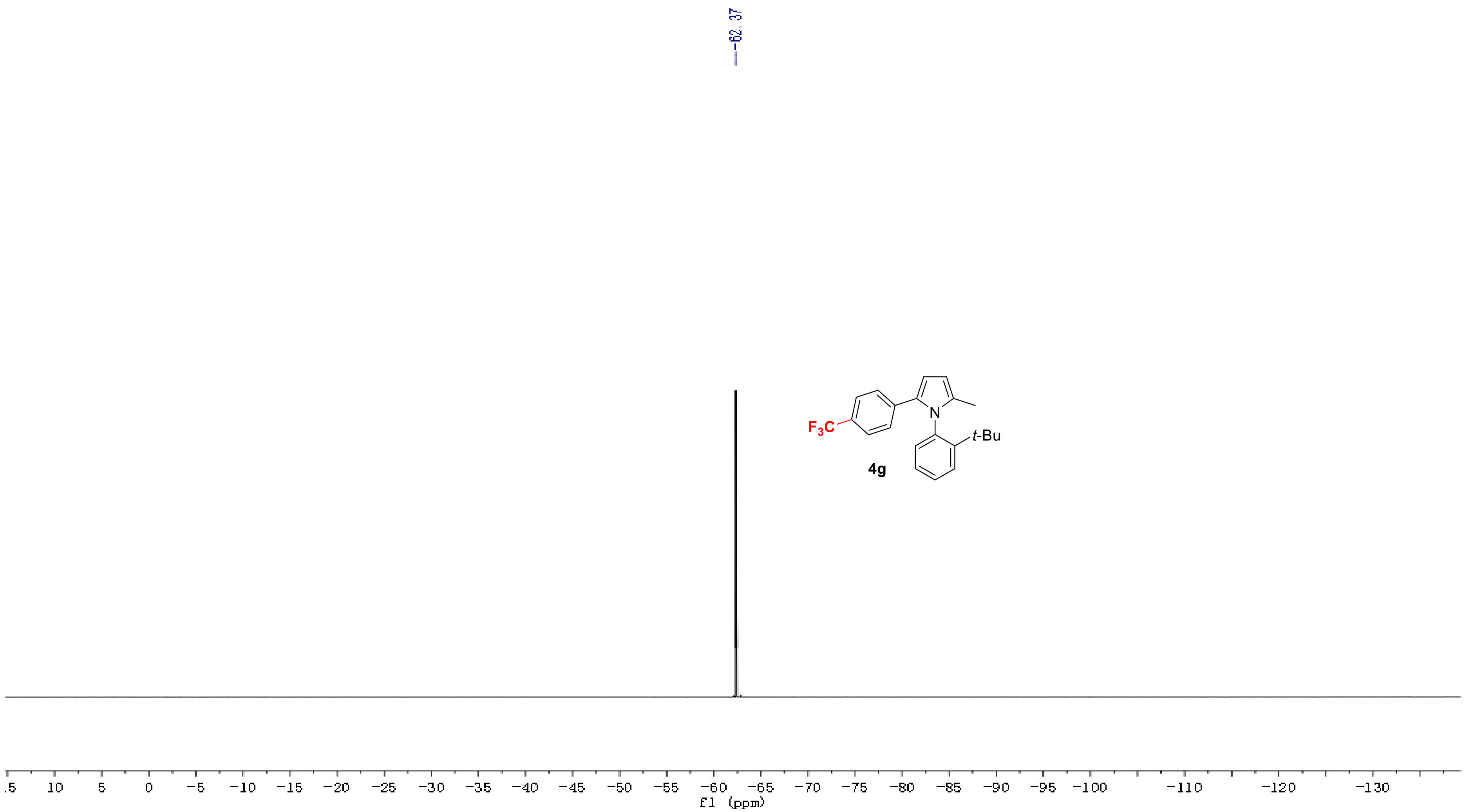


**Supplementary Figure 114.** $^{19}$F NMR of **4g**.

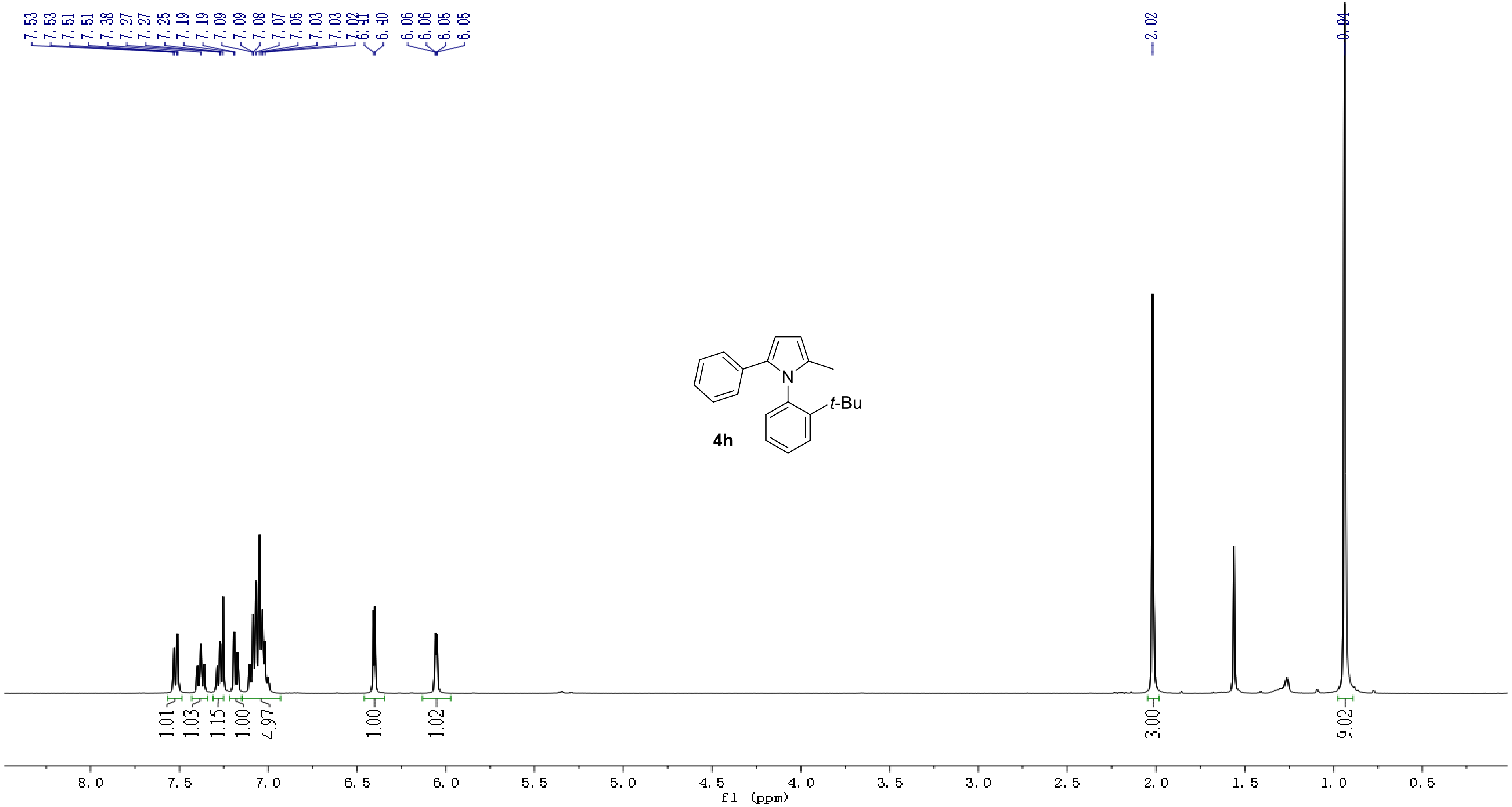


**Supplementary Figure 115.** $^1$H NMR of **4h**.

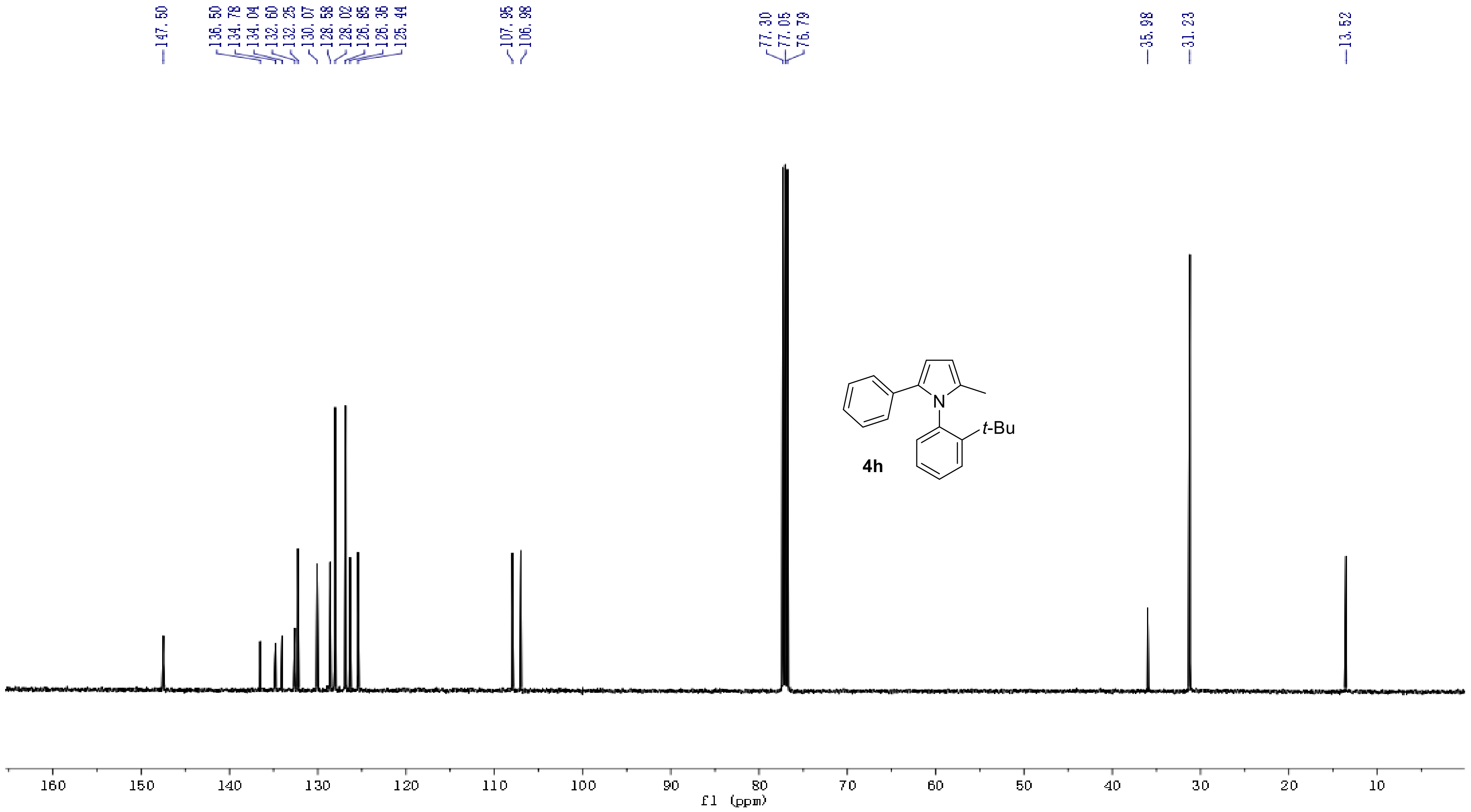


**Supplementary Figure 116.** $^{13}$C NMR of **4h**.

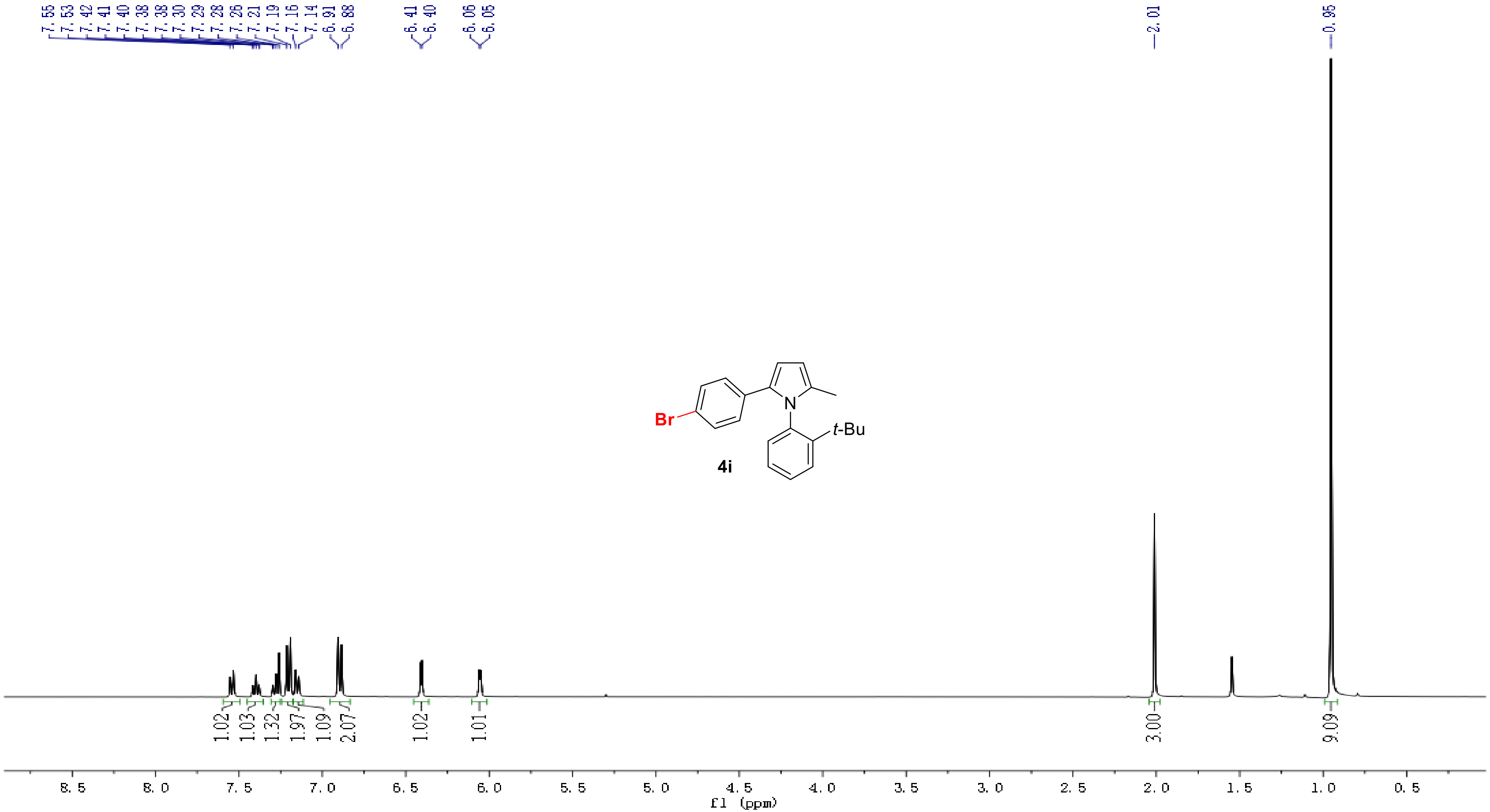


**Supplementary Figure 117.** $^{1}$H NMR of **4i**.

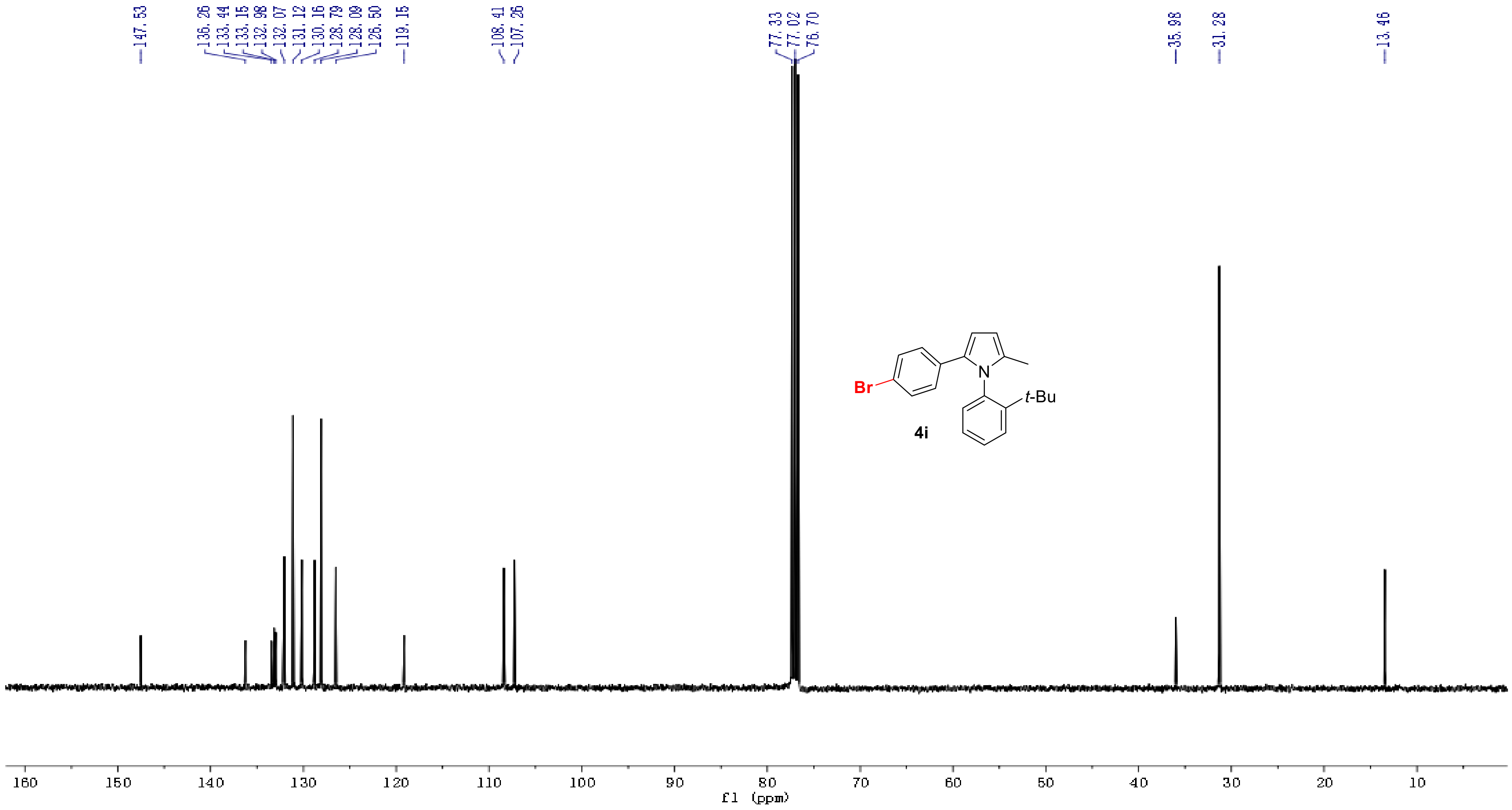


**Supplementary Figure 118.** $^{13}$C NMR of **4i**.

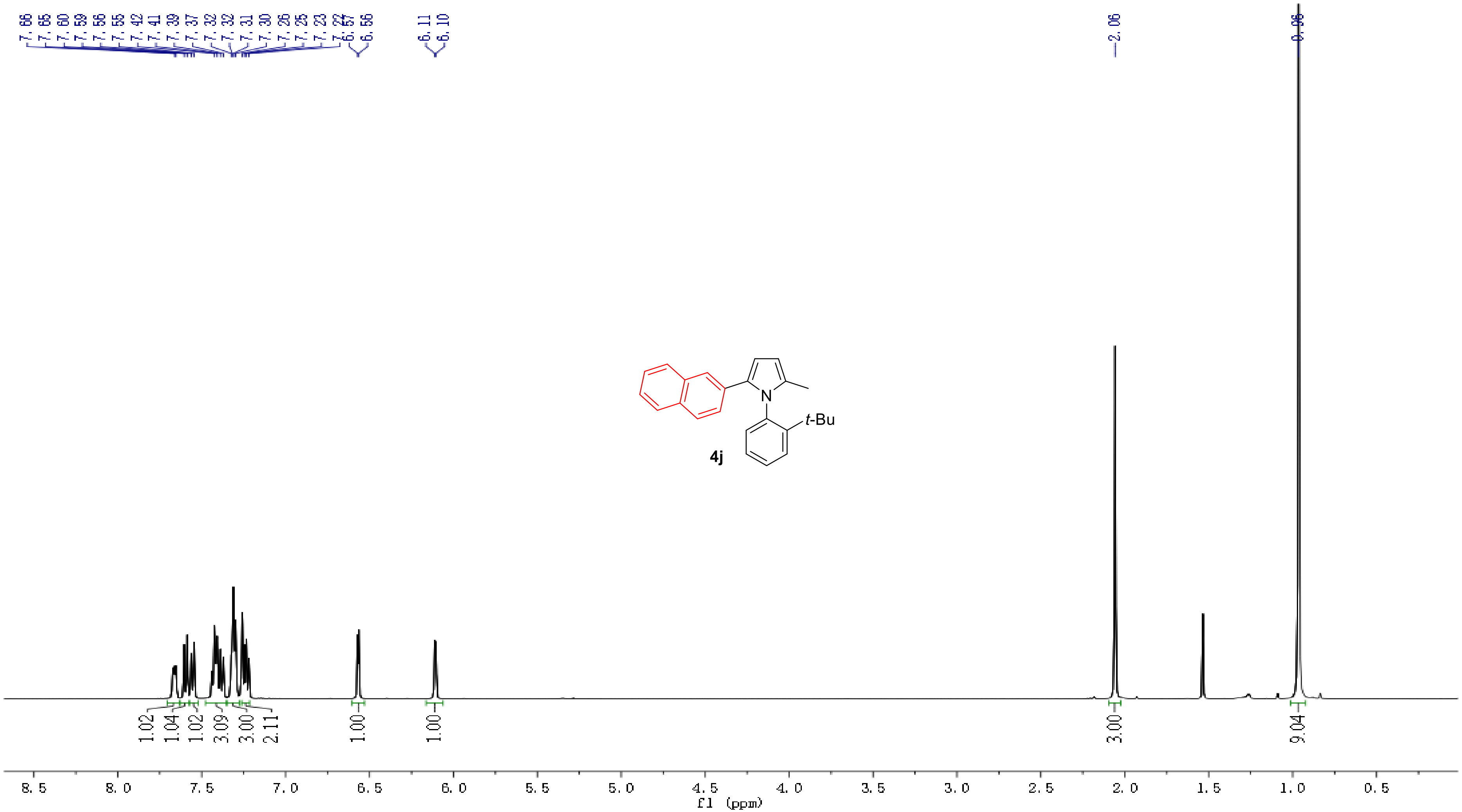


**Supplementary Figure 119.** $^{1}$H NMR of **4j**.

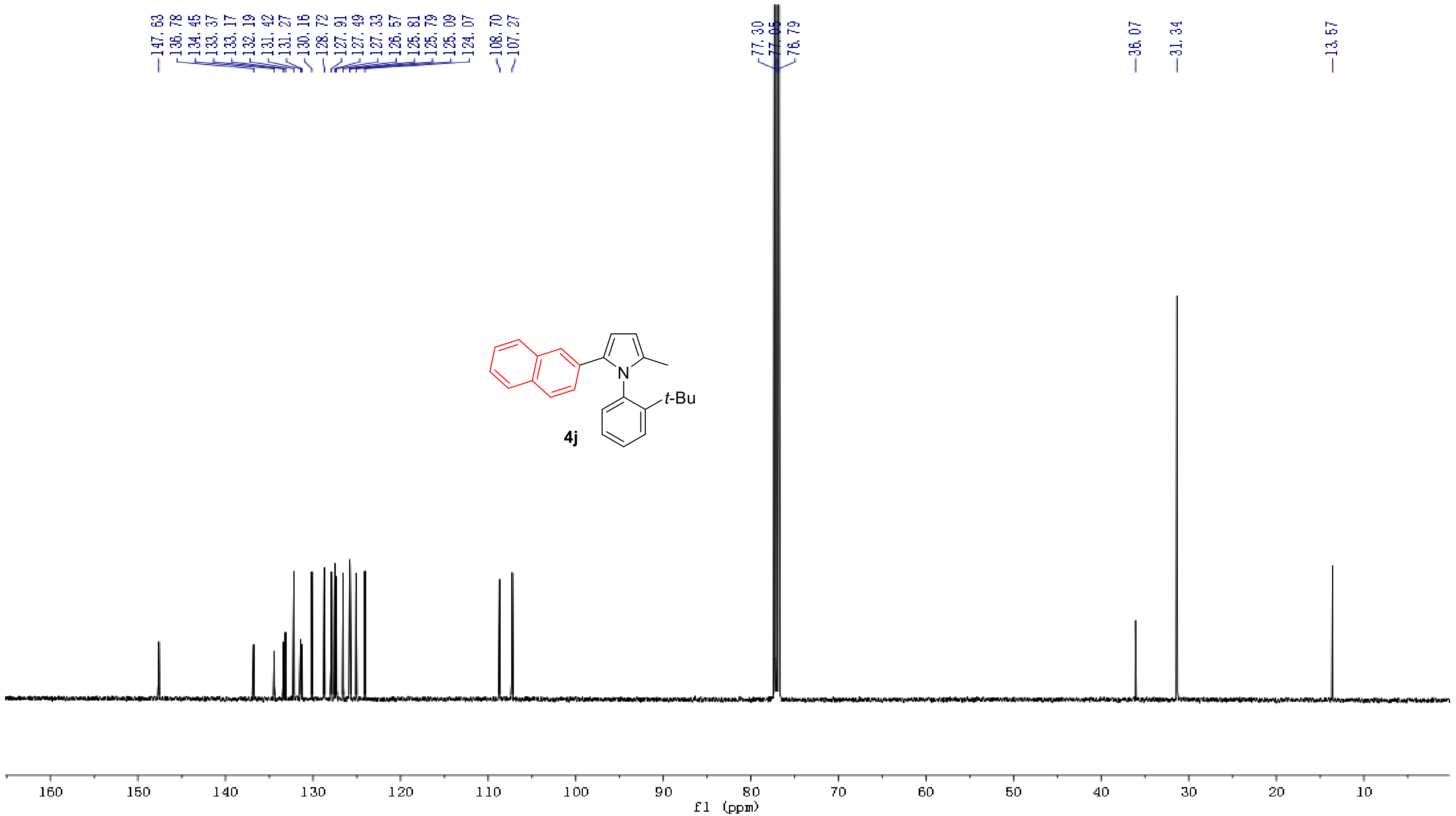


**Supplementary Figure 120.** $^{13}$C NMR of **4j**.

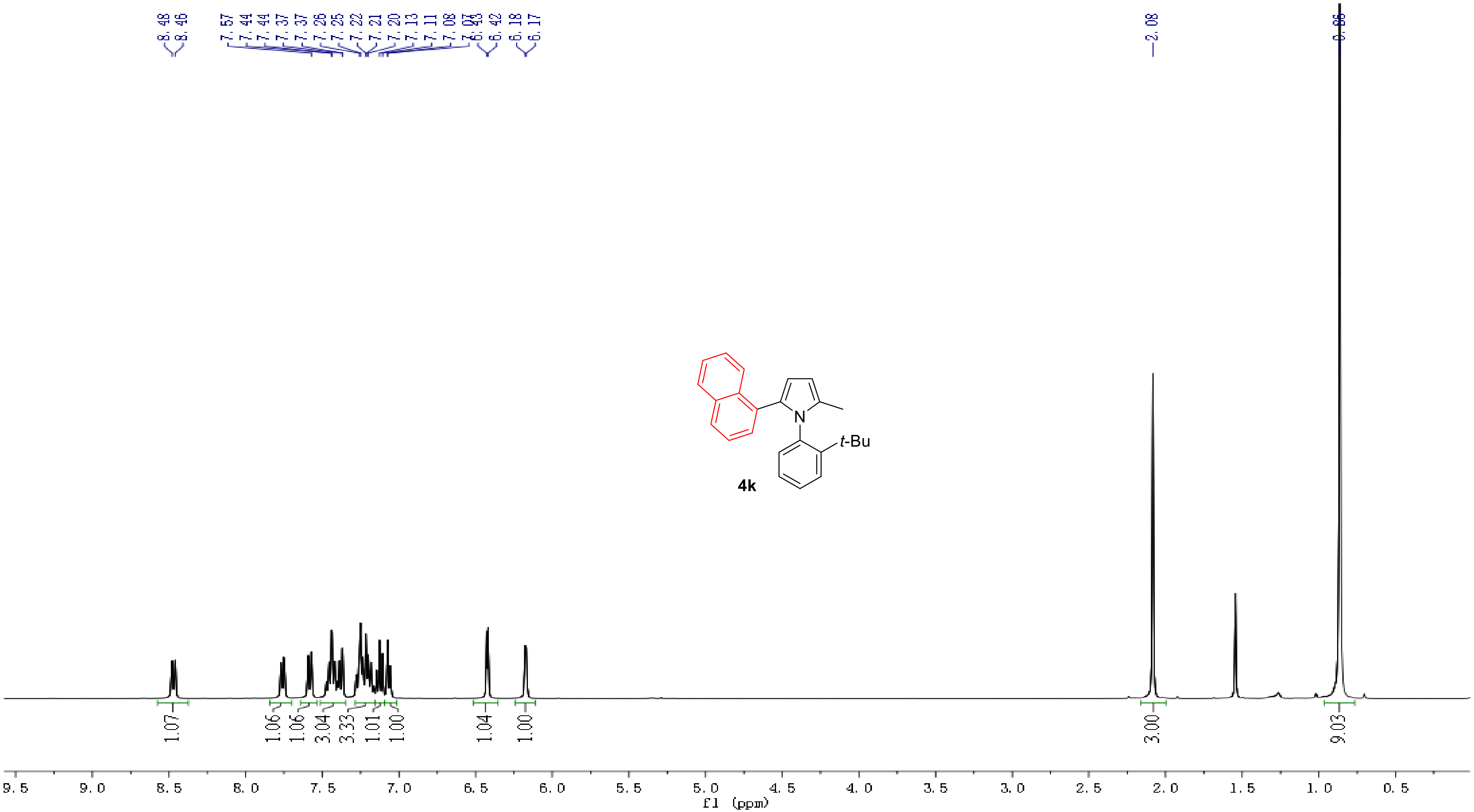


**Supplementary Figure 121.** $^{1}$H NMR of **4k**.

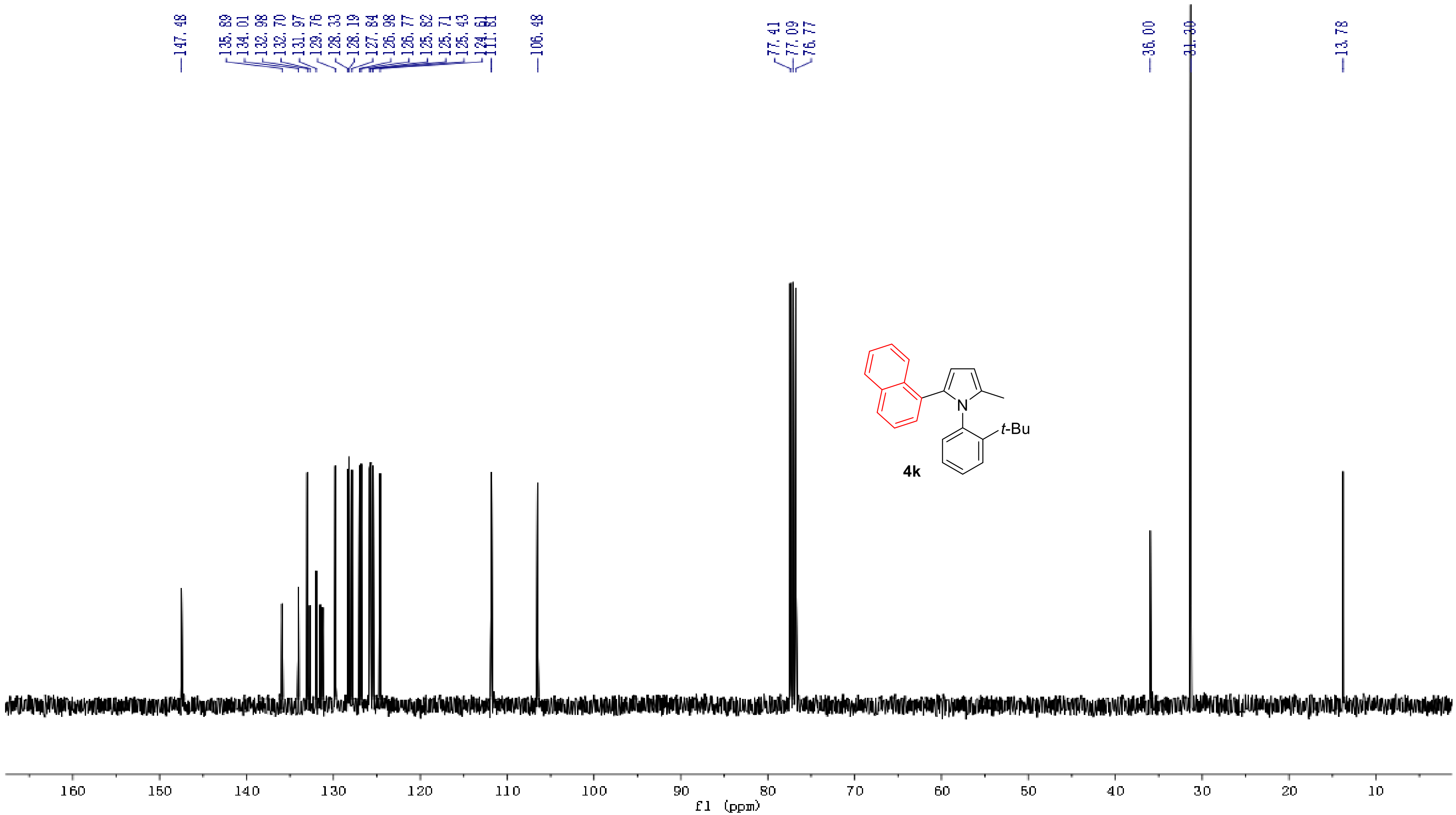


**Supplementary Figure 122.** $^{13}$C NMR of **4k**.

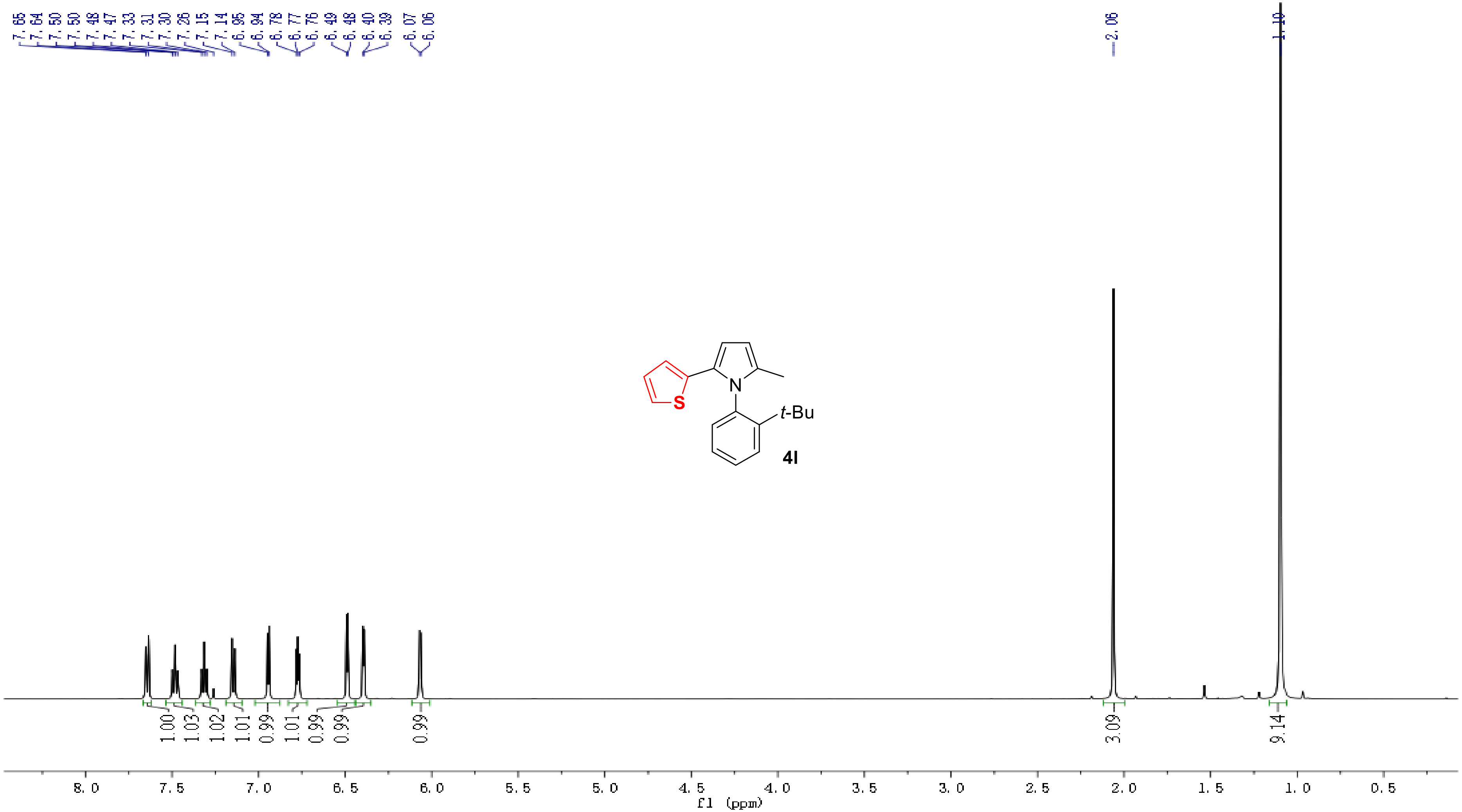


**Supplementary Figure 123.** $^{1}$H NMR of **4l**.

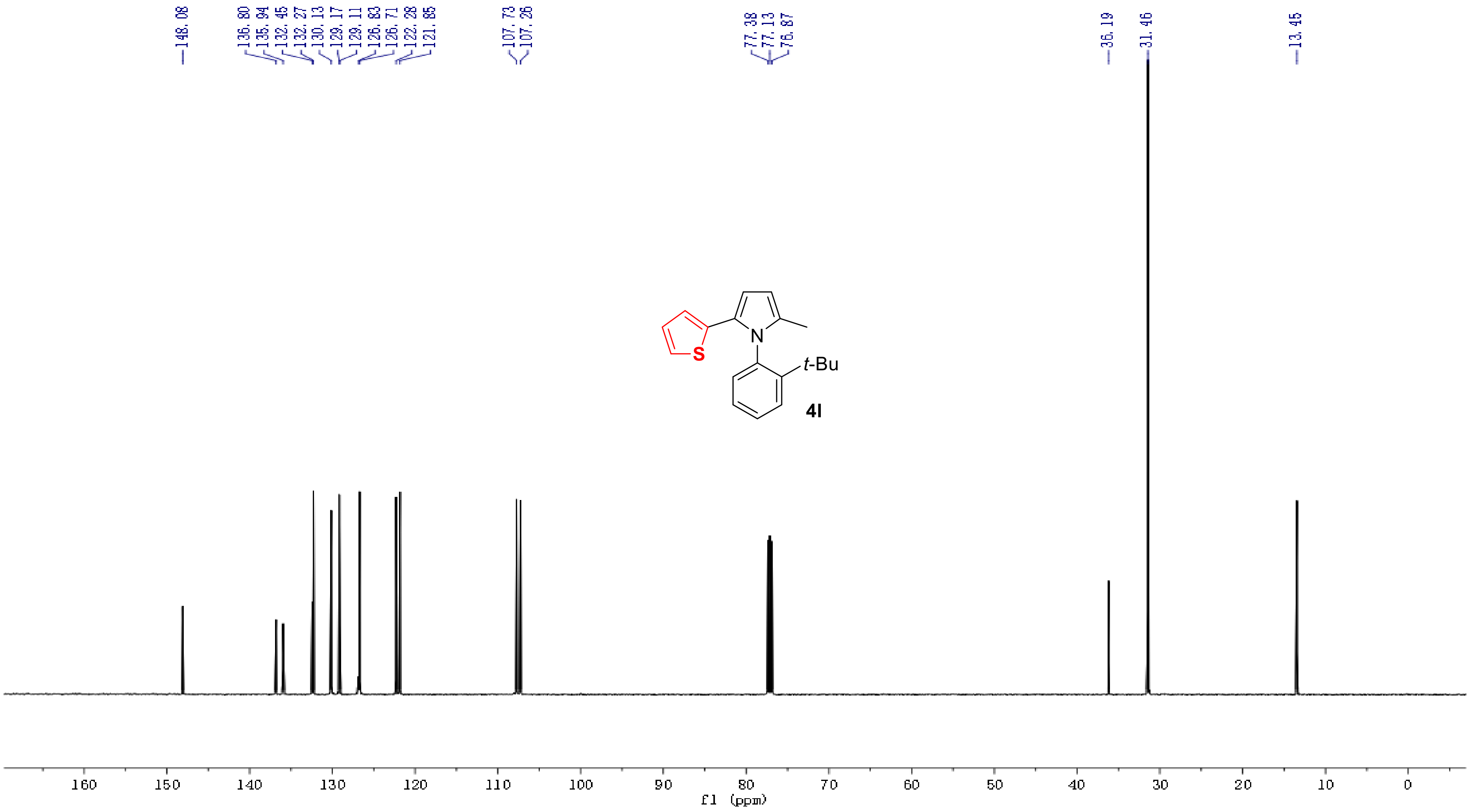


**Supplementary Figure 124.** $^{13}$C NMR of **4l**.

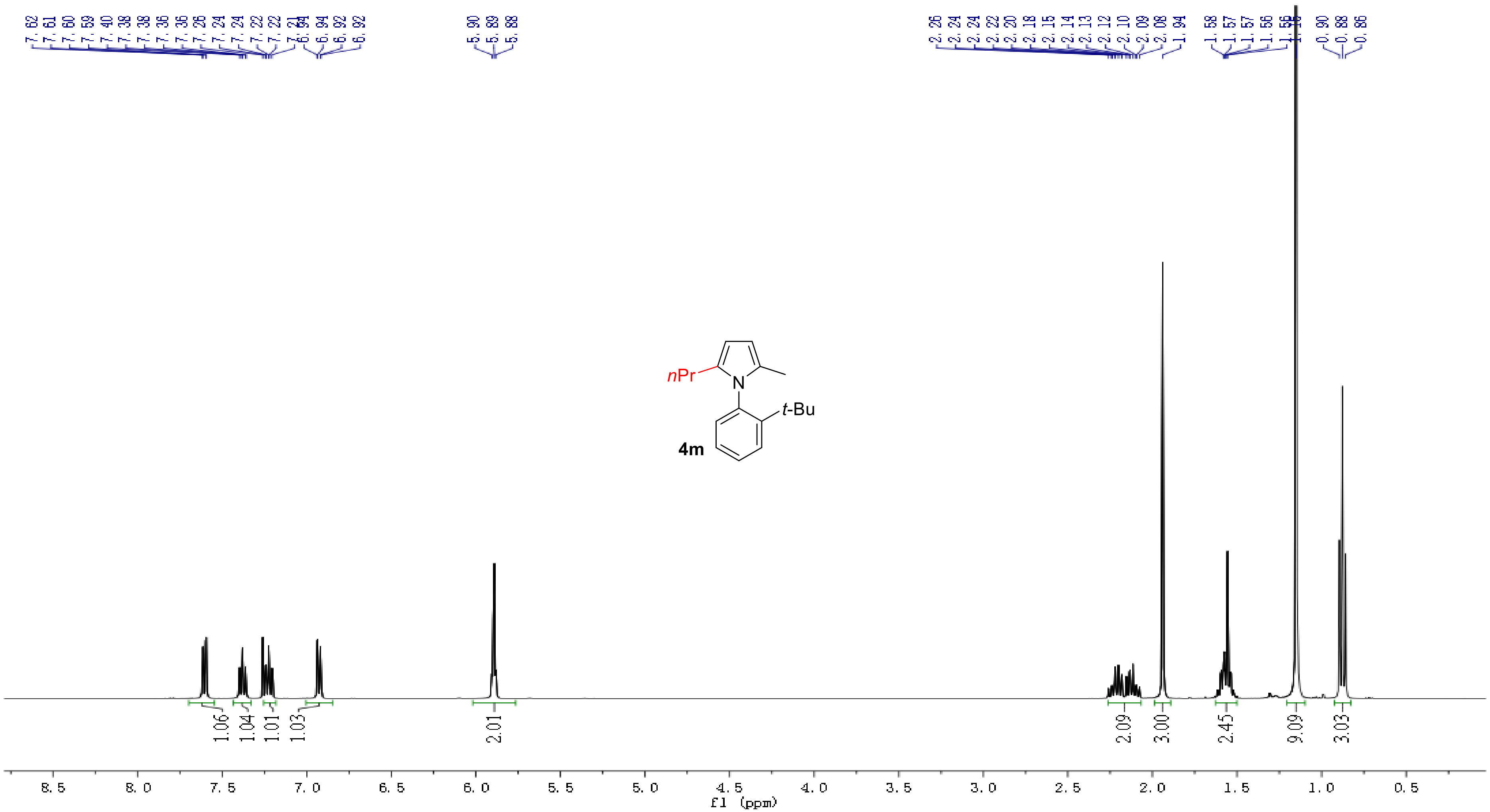

**Supplementary Figure 125.** $^1$H NMR of **4m**.

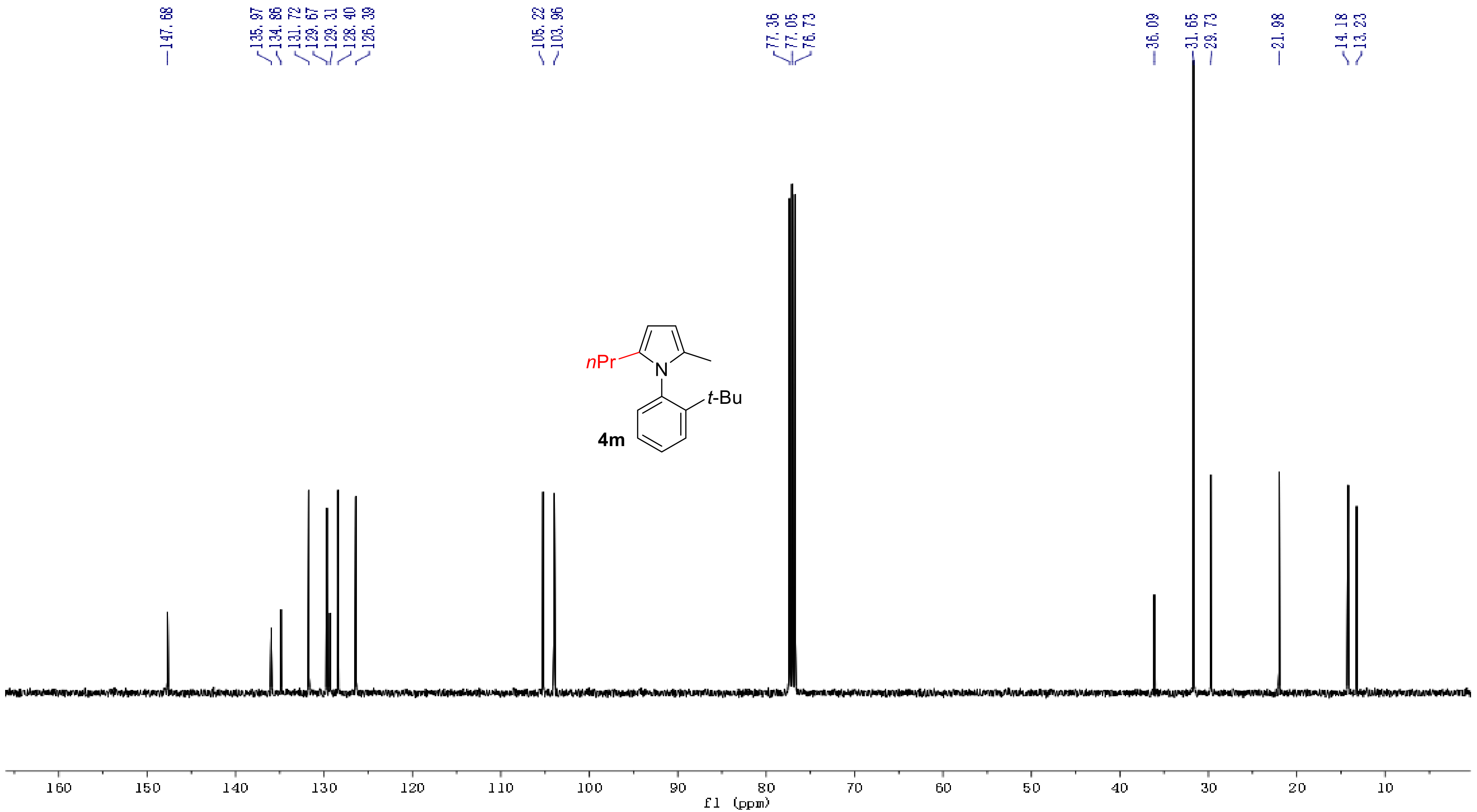


**Supplementary Figure 126.** $^{13}$C NMR of **4m**.

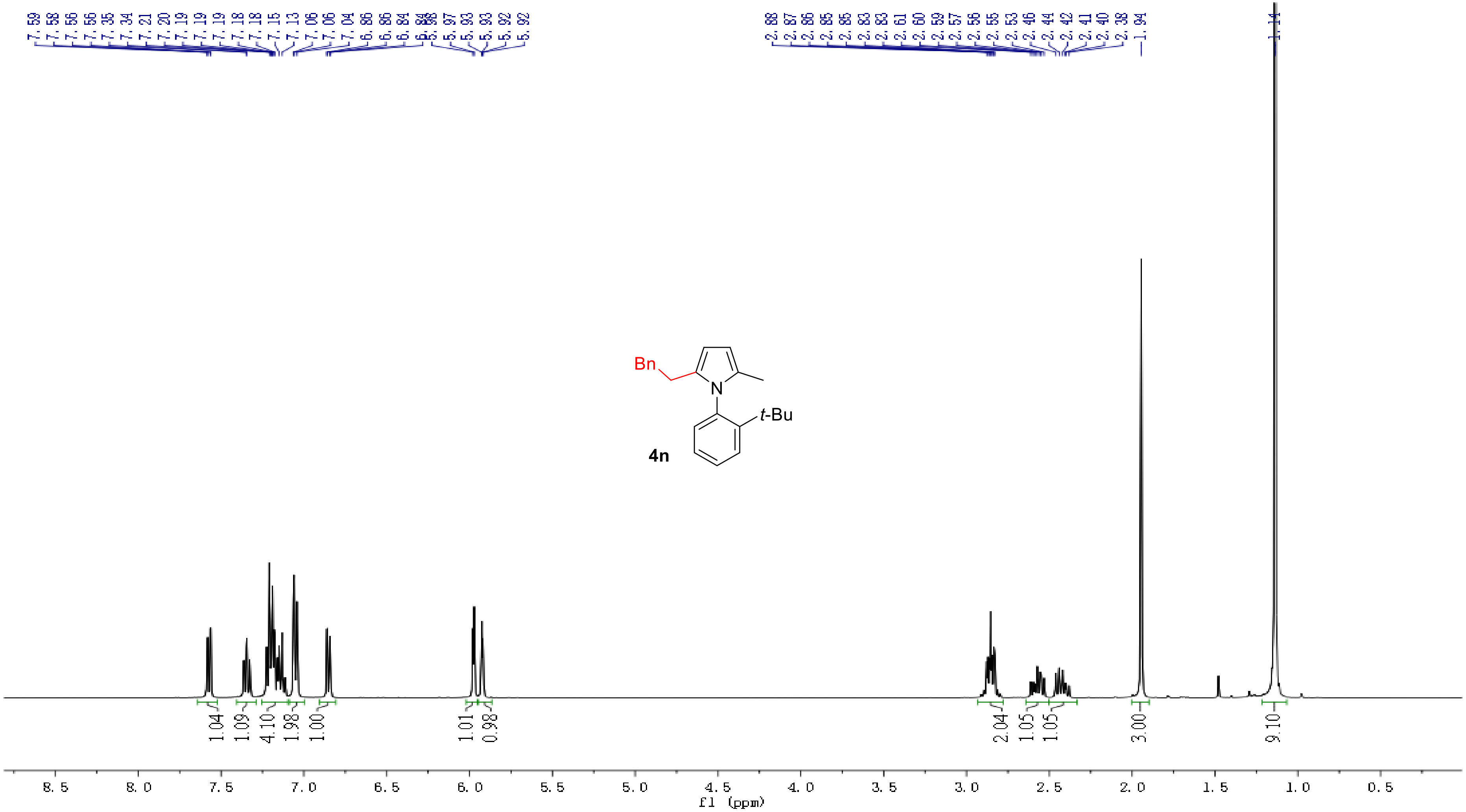


**Supplementary Figure 127.** $^{1}$H NMR of **4n**.

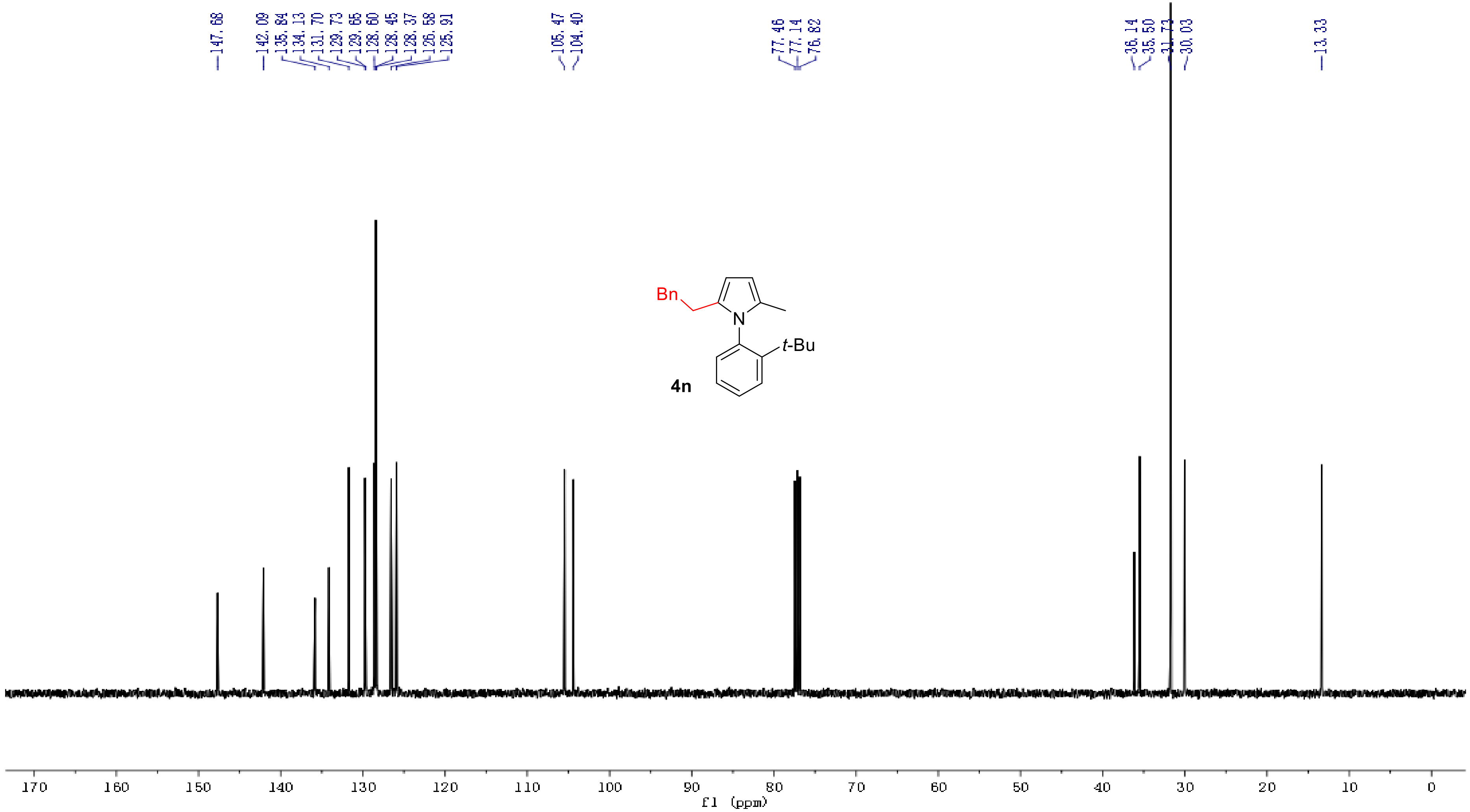


**Supplementary Figure 128.** $^{13}$C NMR of **4n**.

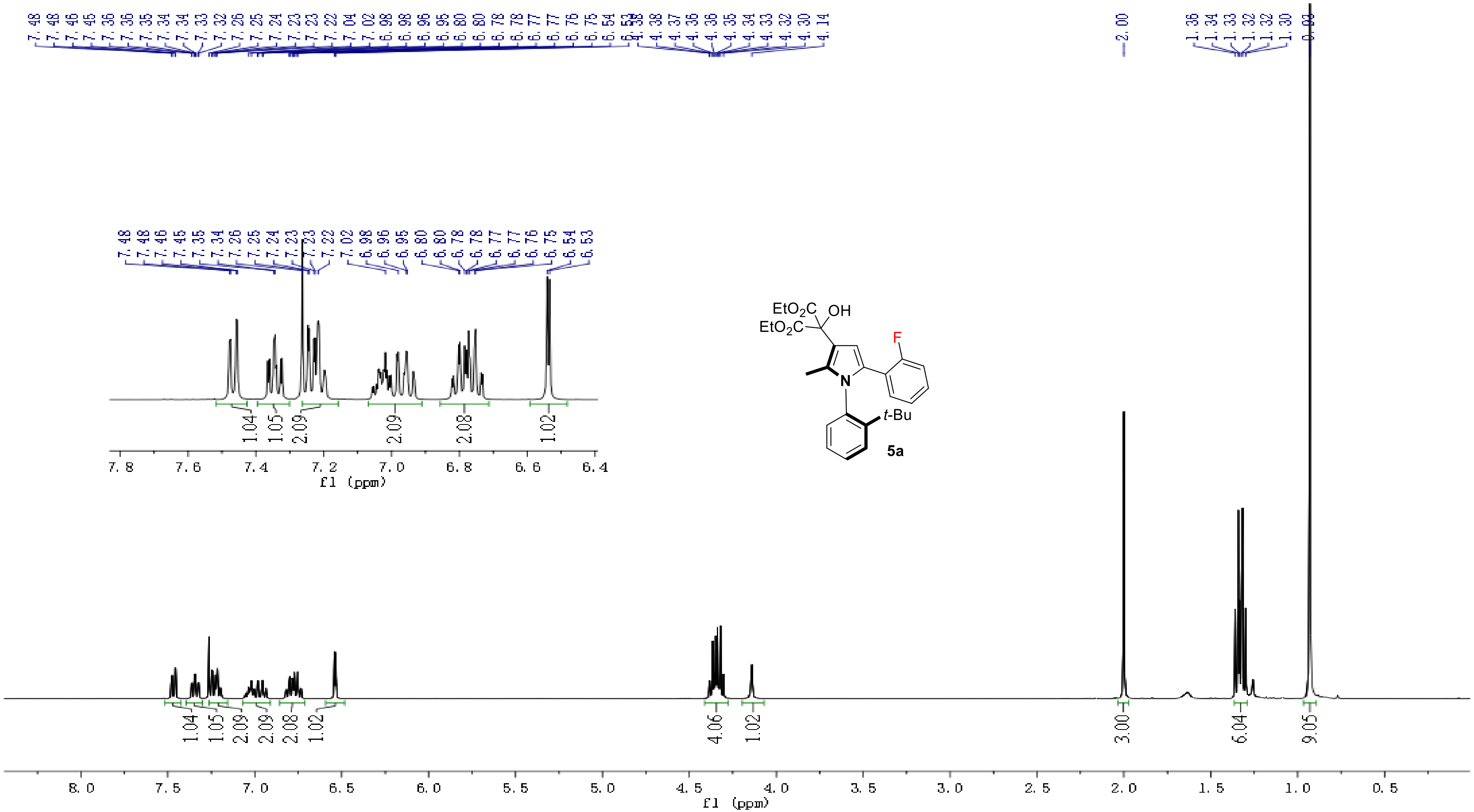


**Supplementary Figure 129.** $^{1}$H NMR of **5a**.

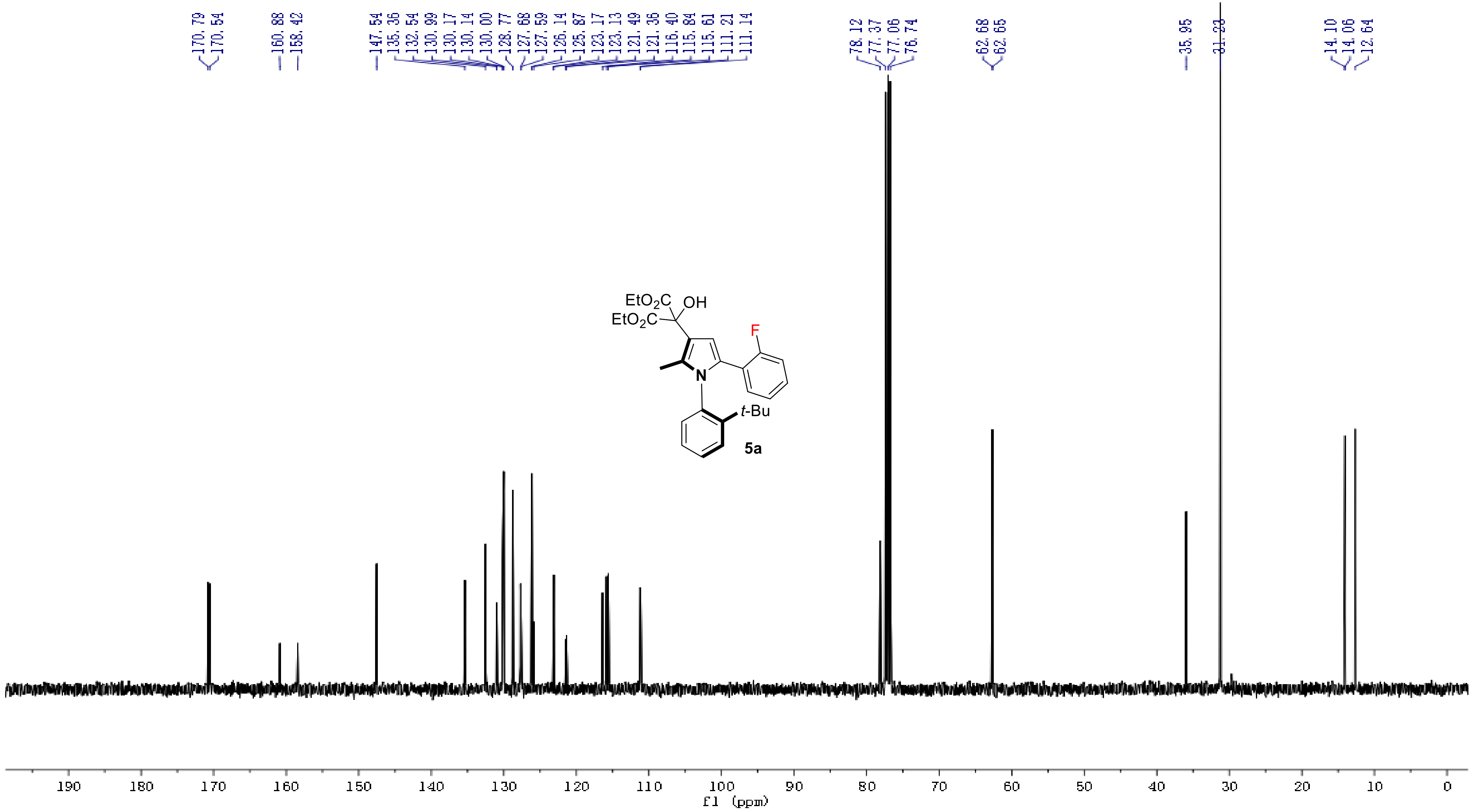


**Supplementary Figure 130.** $^{13}$C NMR of **5a**.

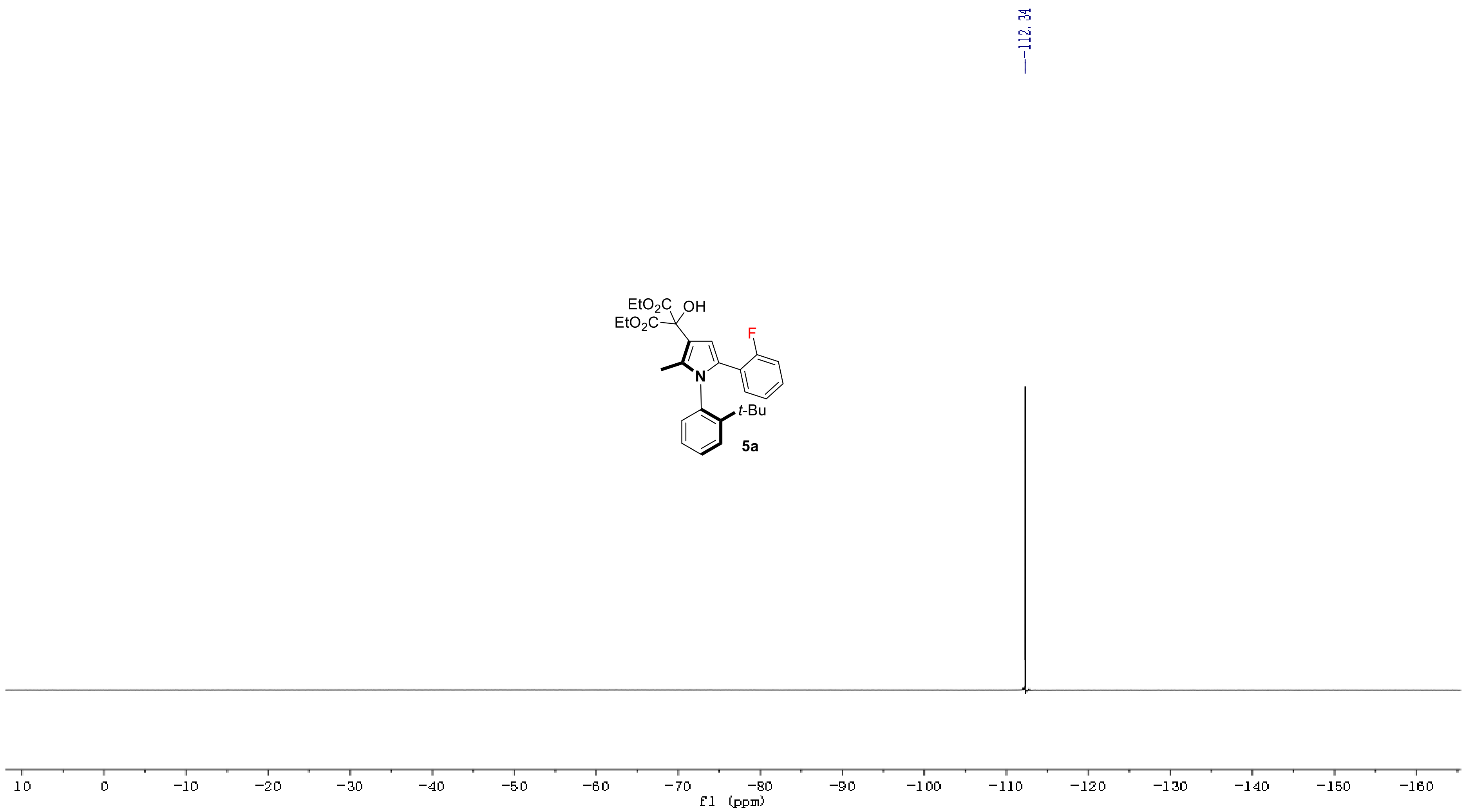


**Supplementary Figure 131.** $^{19}$F NMR of **5a**.

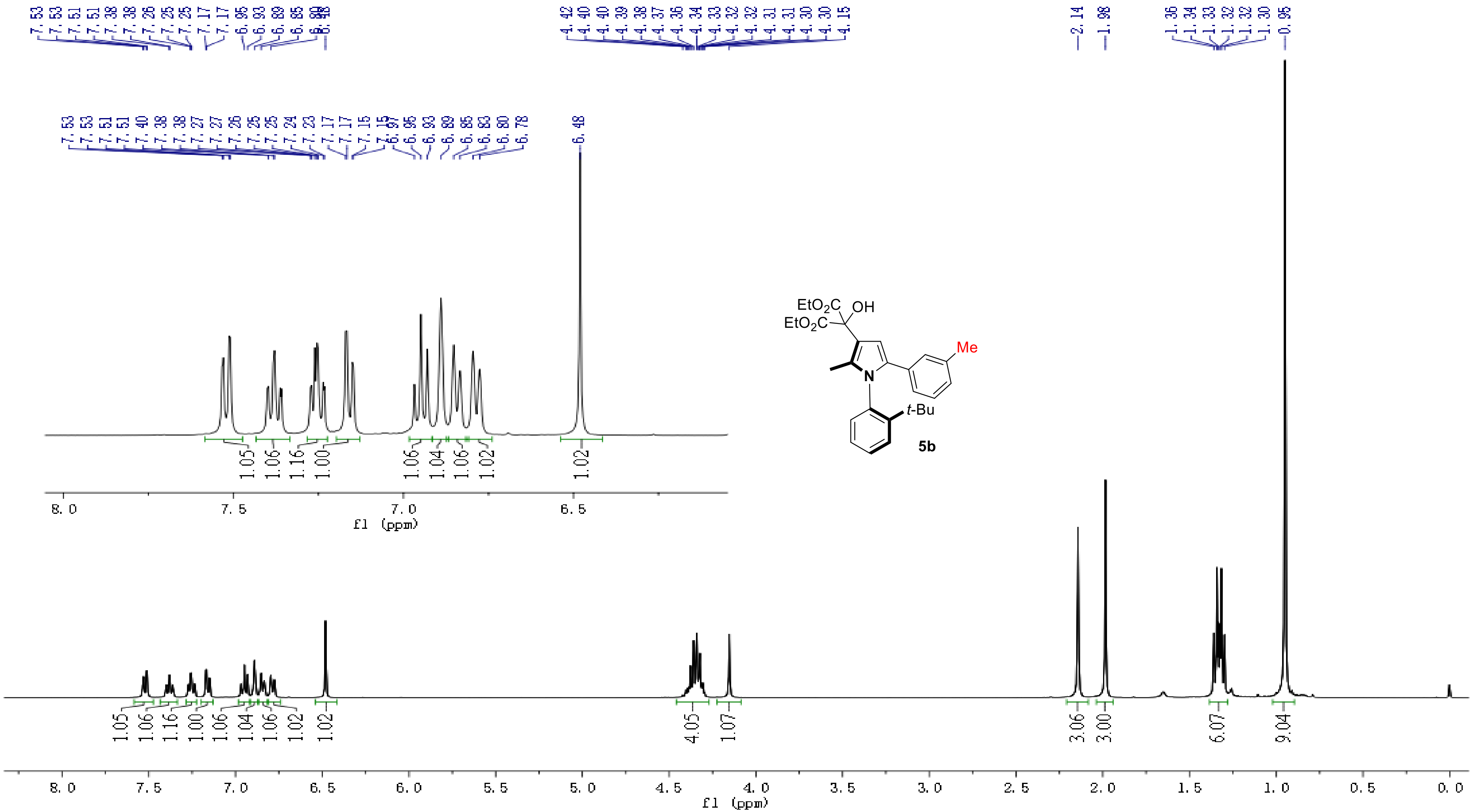


**Supplementary Figure 132.** $^{1}$H NMR of **5b**.

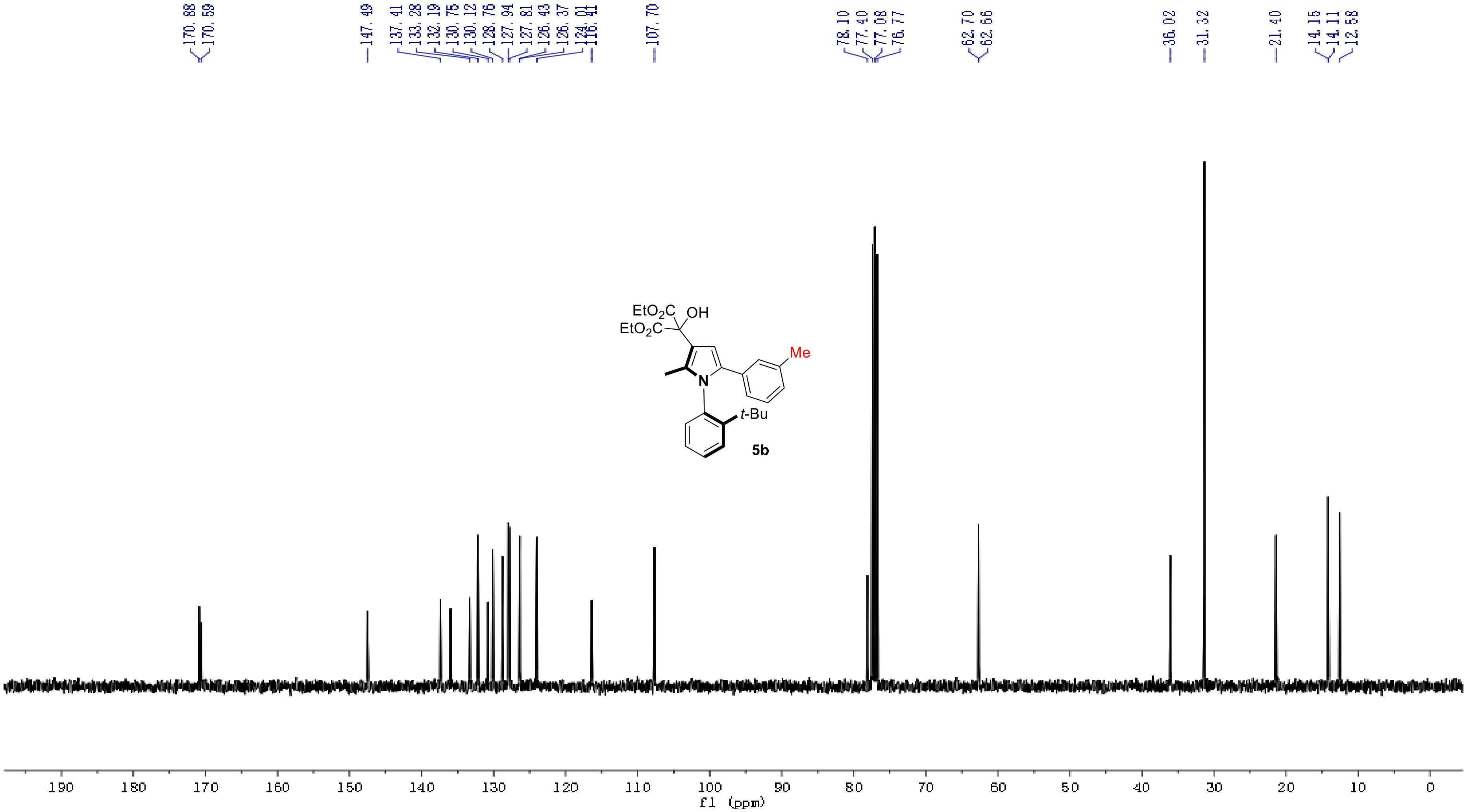


**Supplementary Figure 133.** $^{13}$C NMR of **5b**.

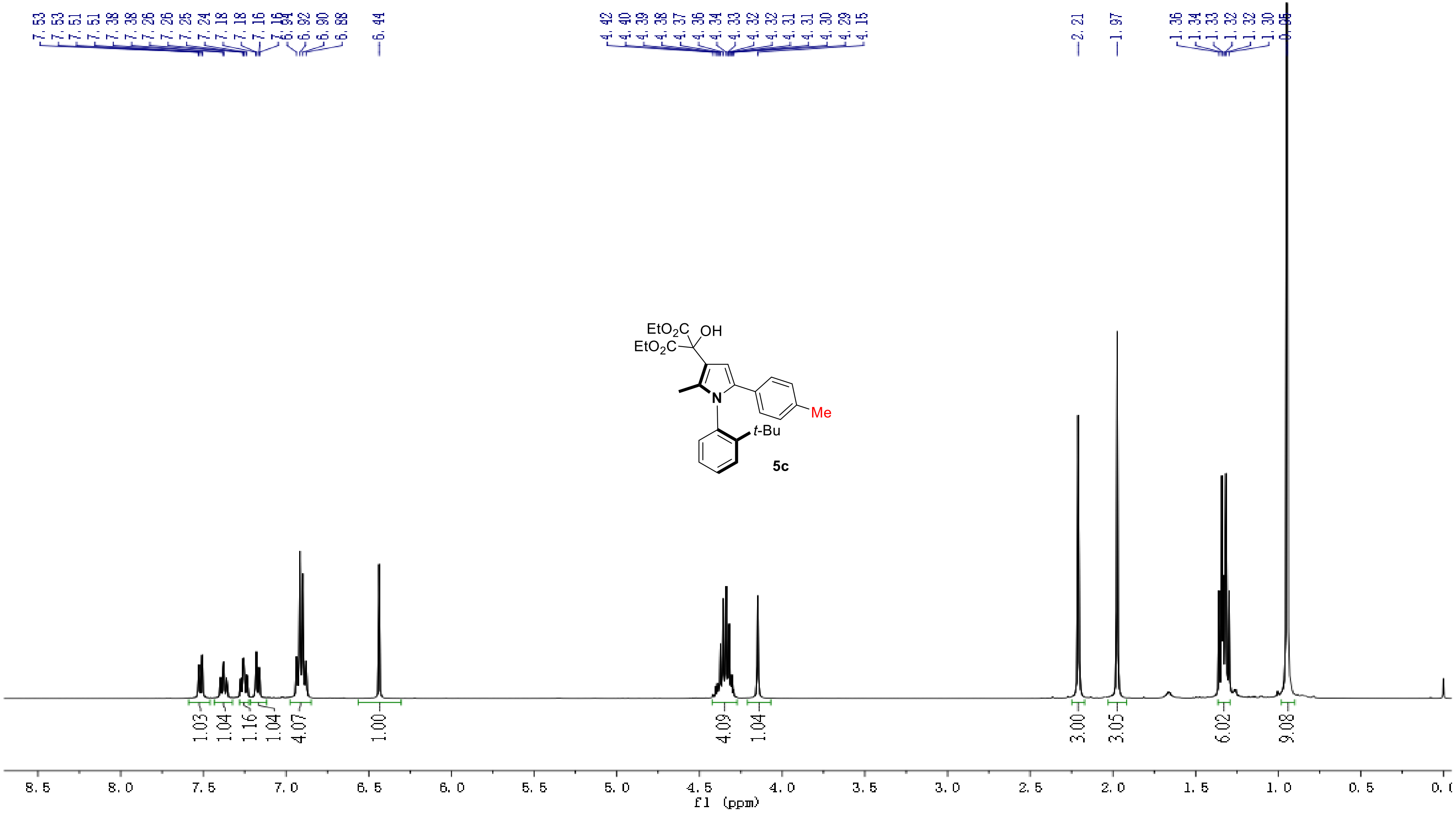


**Supplementary Figure 134.** $^{1}$H NMR of **5c**.

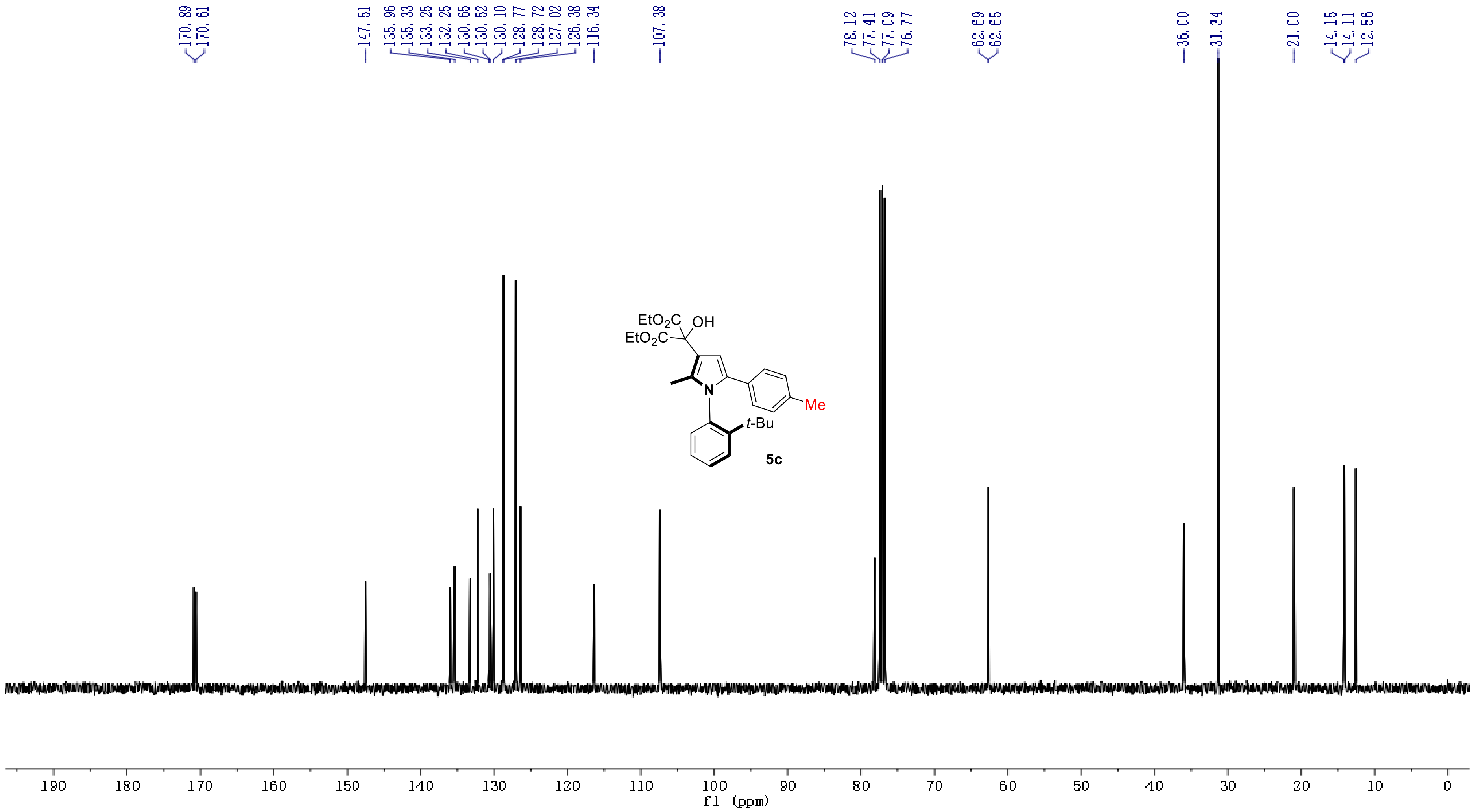


**Supplementary Figure 135.** $^{13}$C NMR of **5c**.

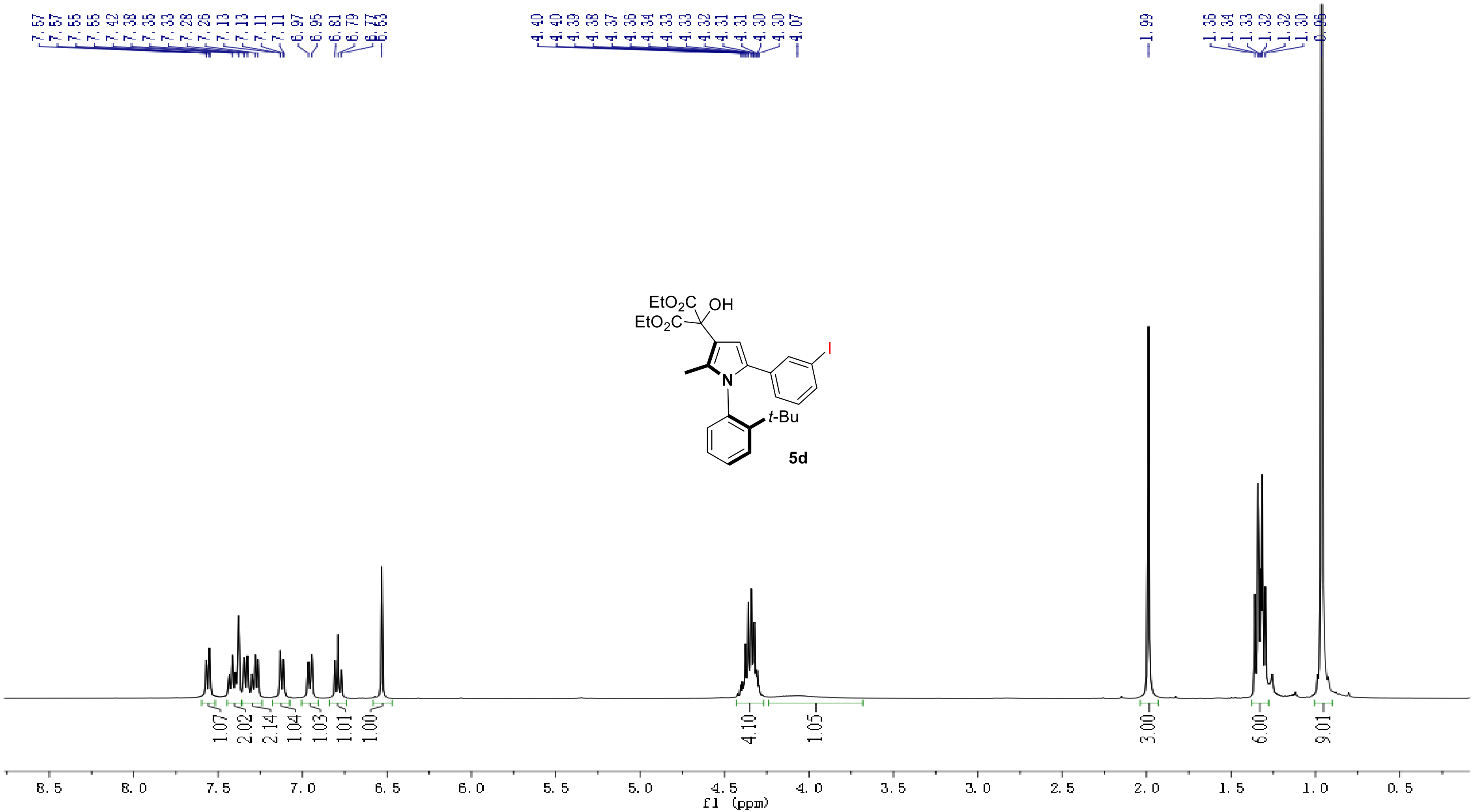

**Supplementary Figure 136.** $^{1}$H NMR of **5d**.

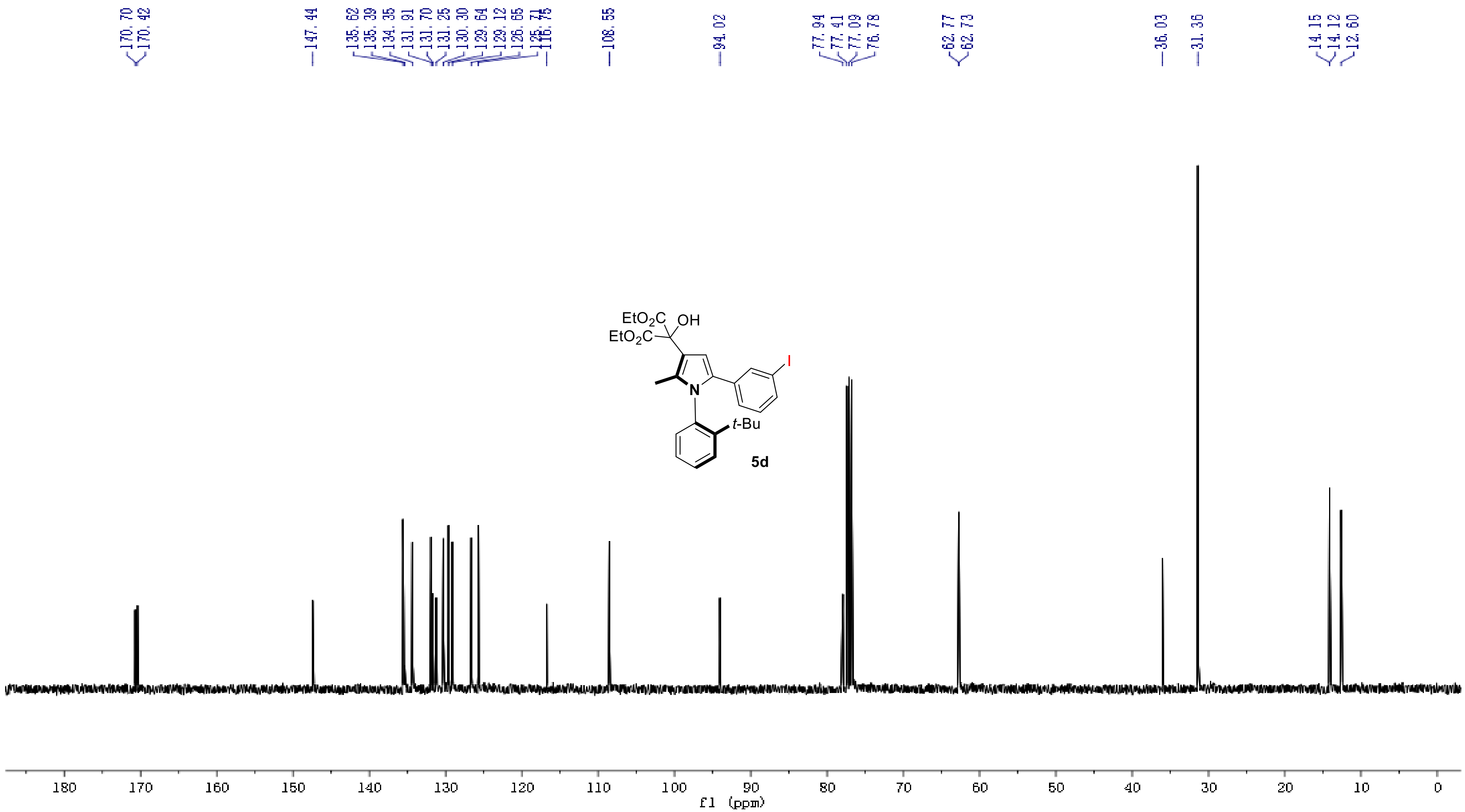


**Supplementary Figure 137.** $^{13}C$ NMR of **5d**.

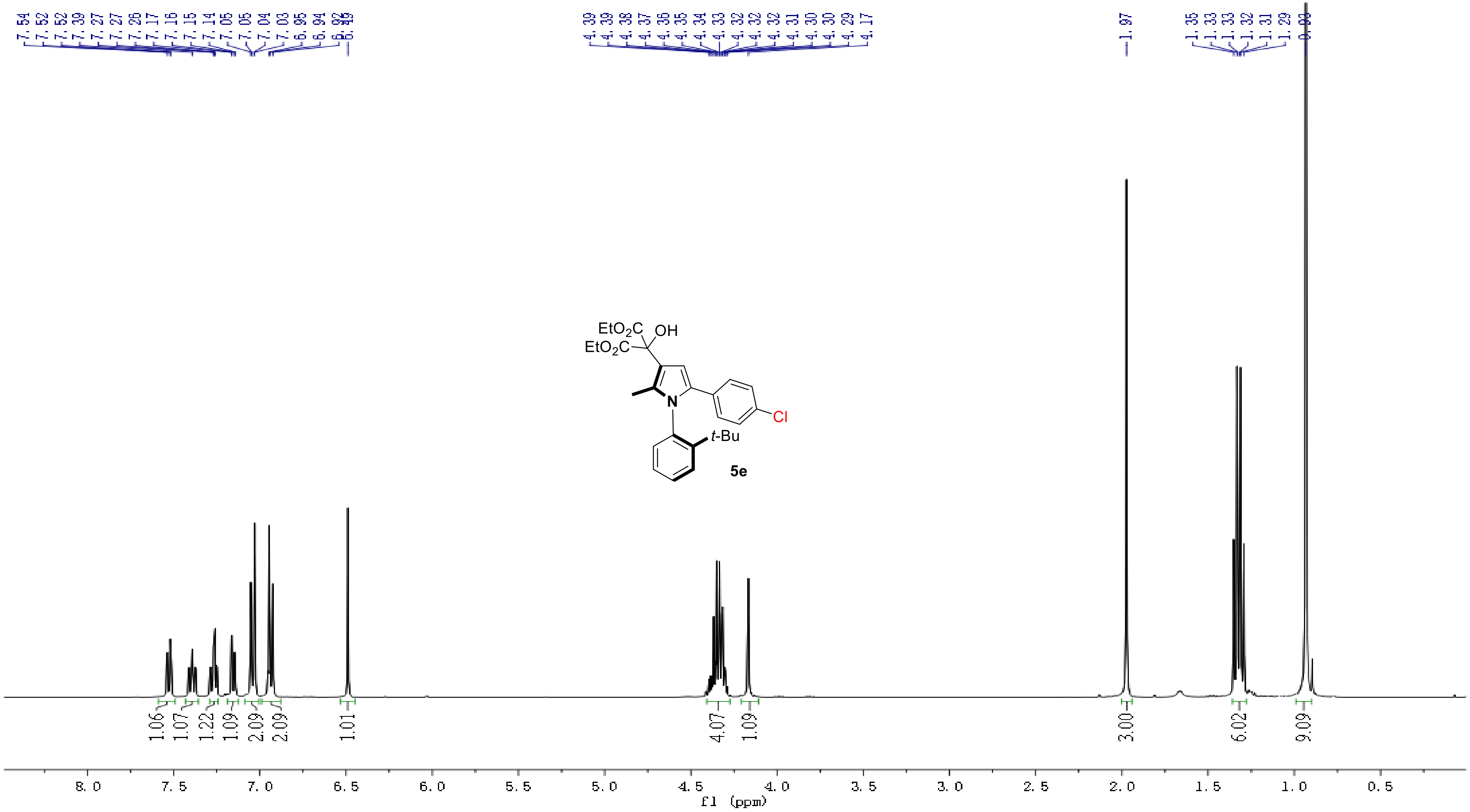


**Supplementary Figure 138.** $^{1}$H NMR of **5e**.

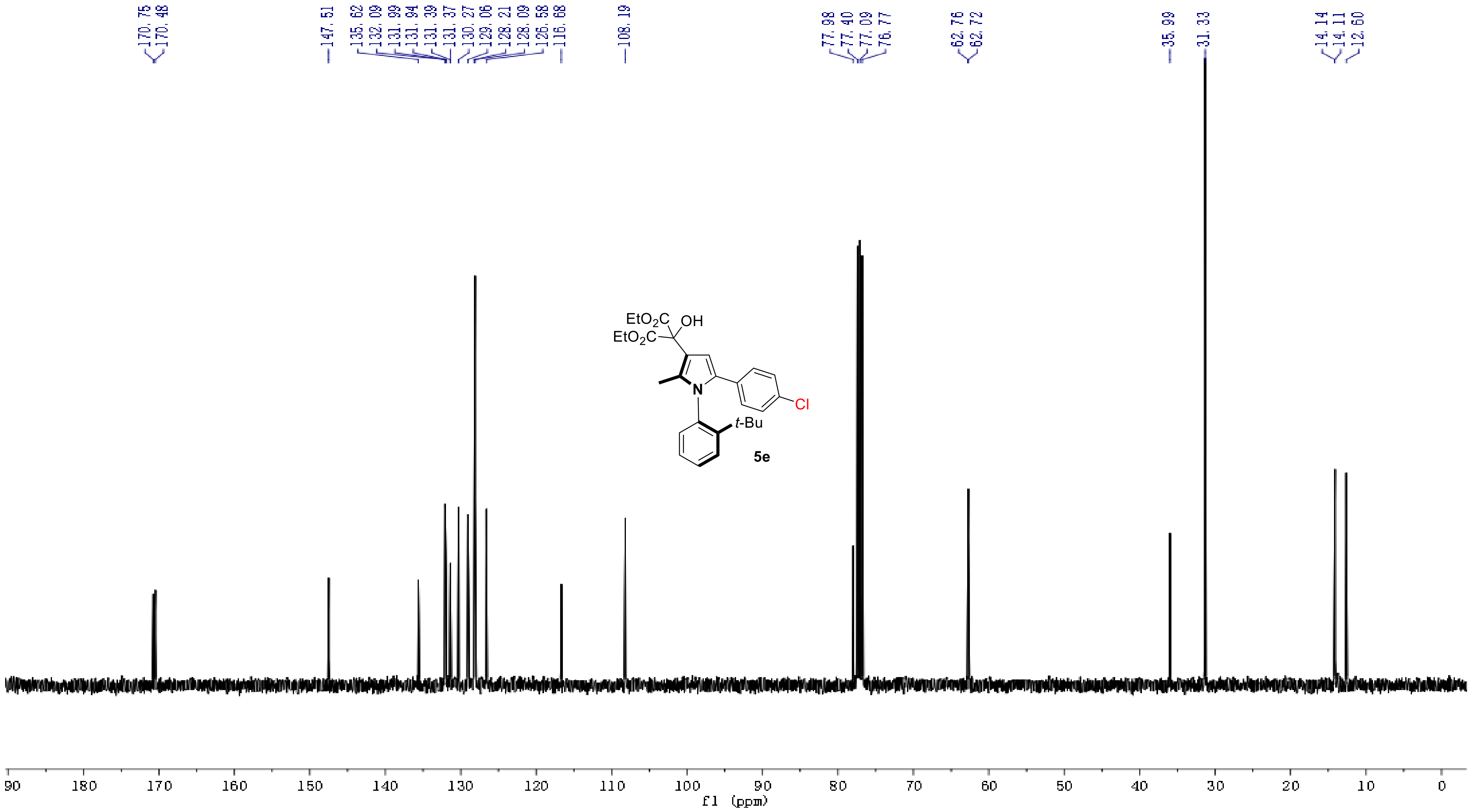


**Supplementary Figure 139.** $^{13}$C NMR of **5e**.

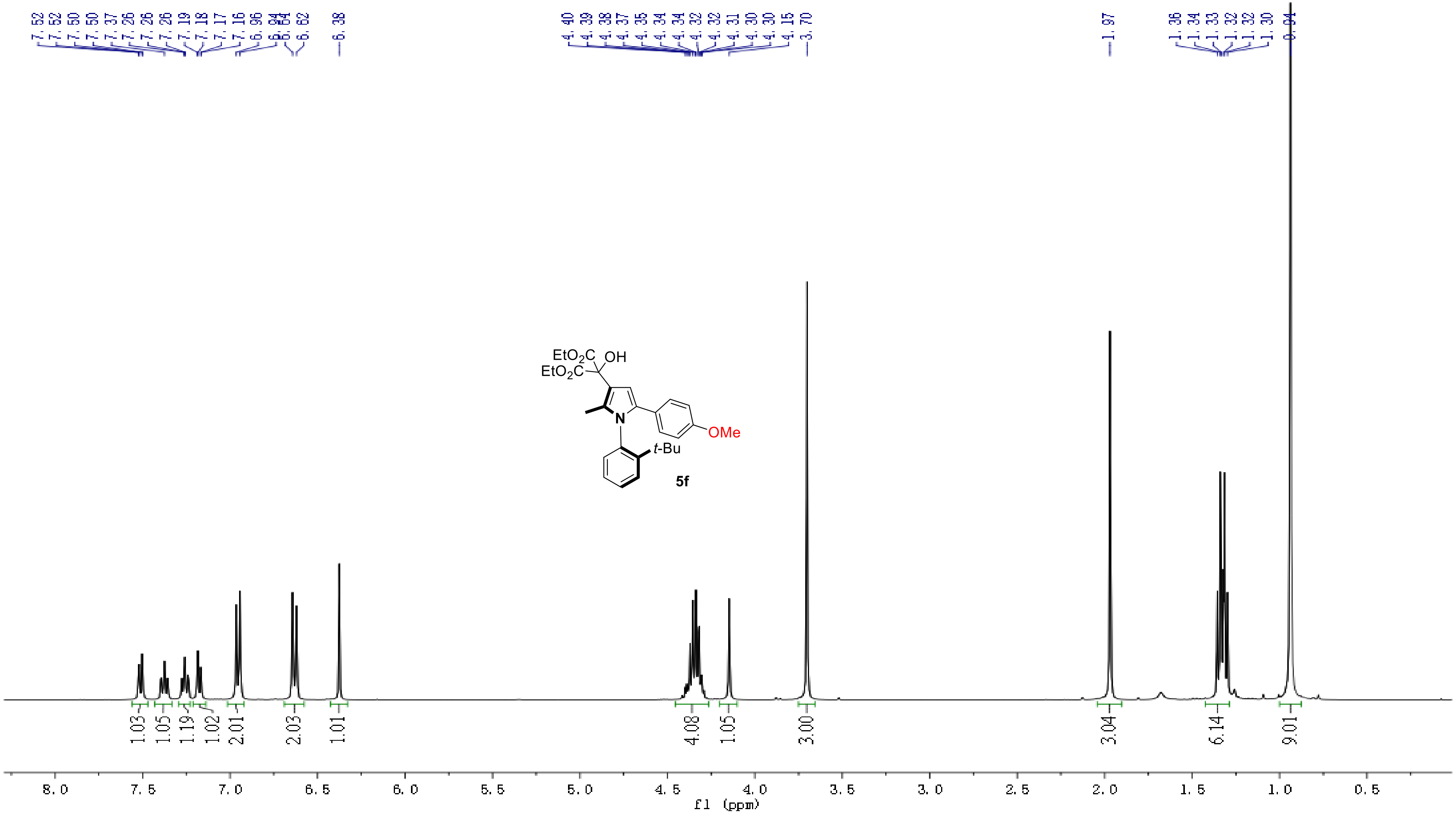

**Supplementary Figure 140.** $^{1}$H NMR of **5f**.

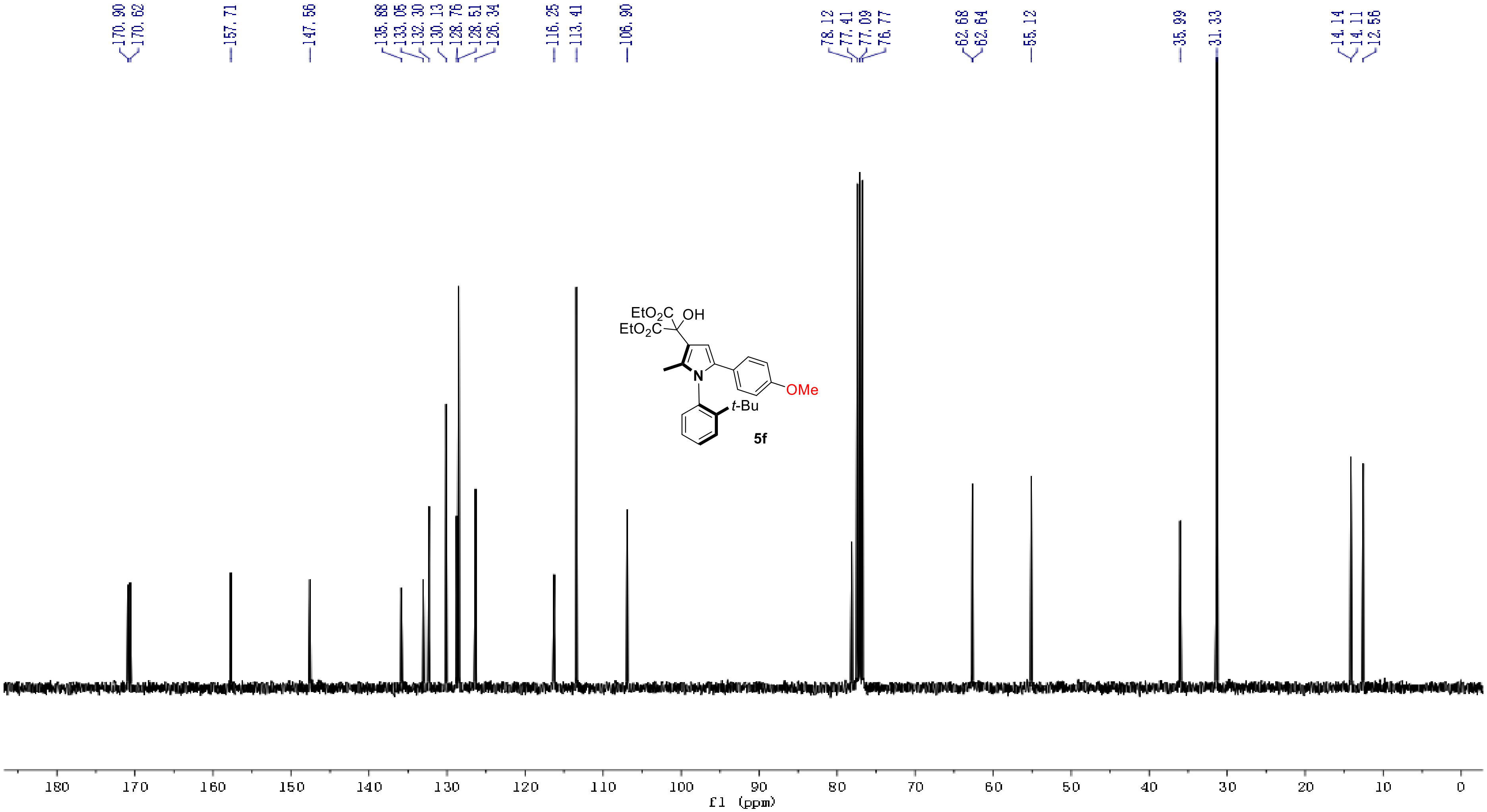


**Supplementary Figure 141.** $^{13}$C NMR of **5f**.

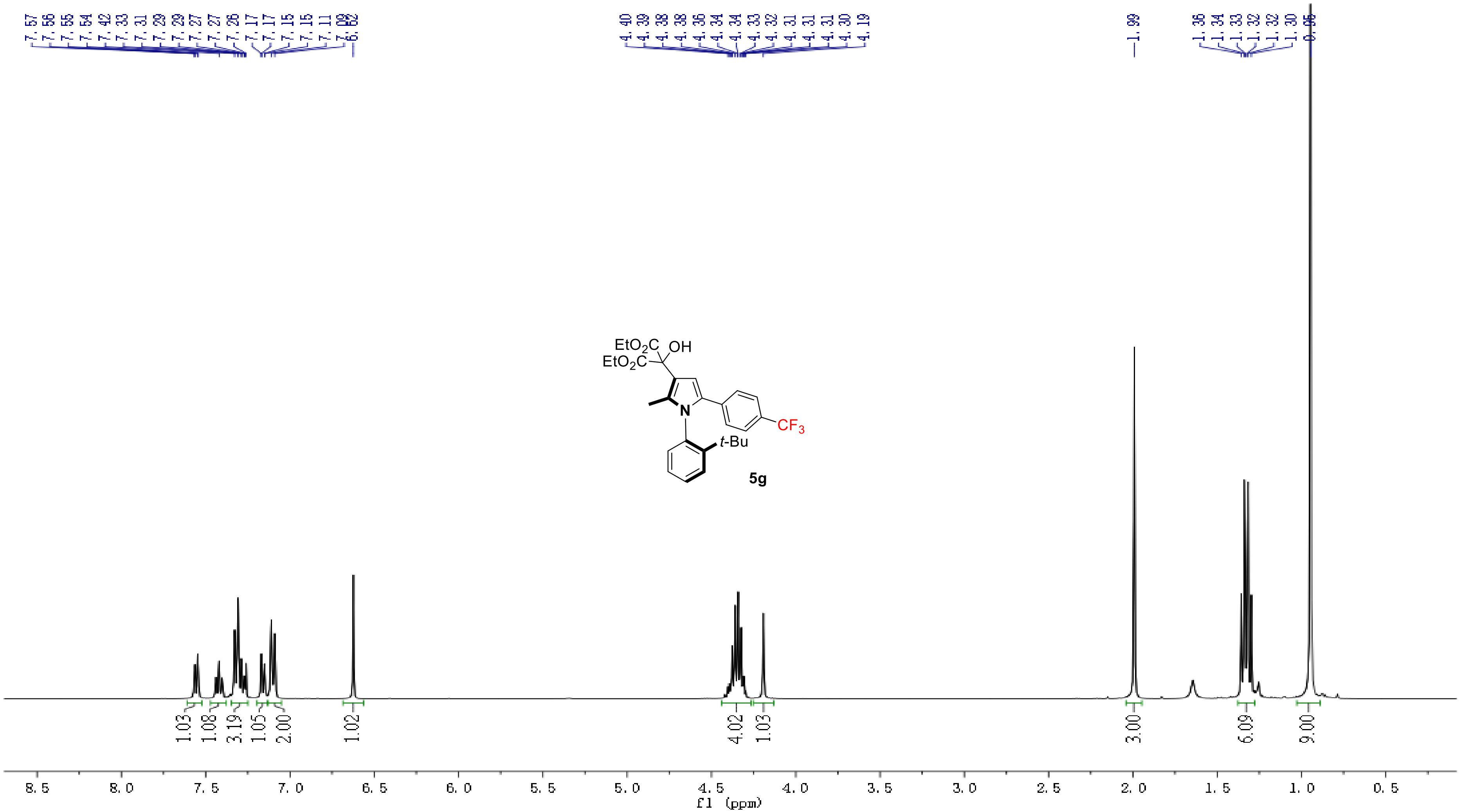

**Supplementary Figure 142.** $^1$H NMR of **5g**.

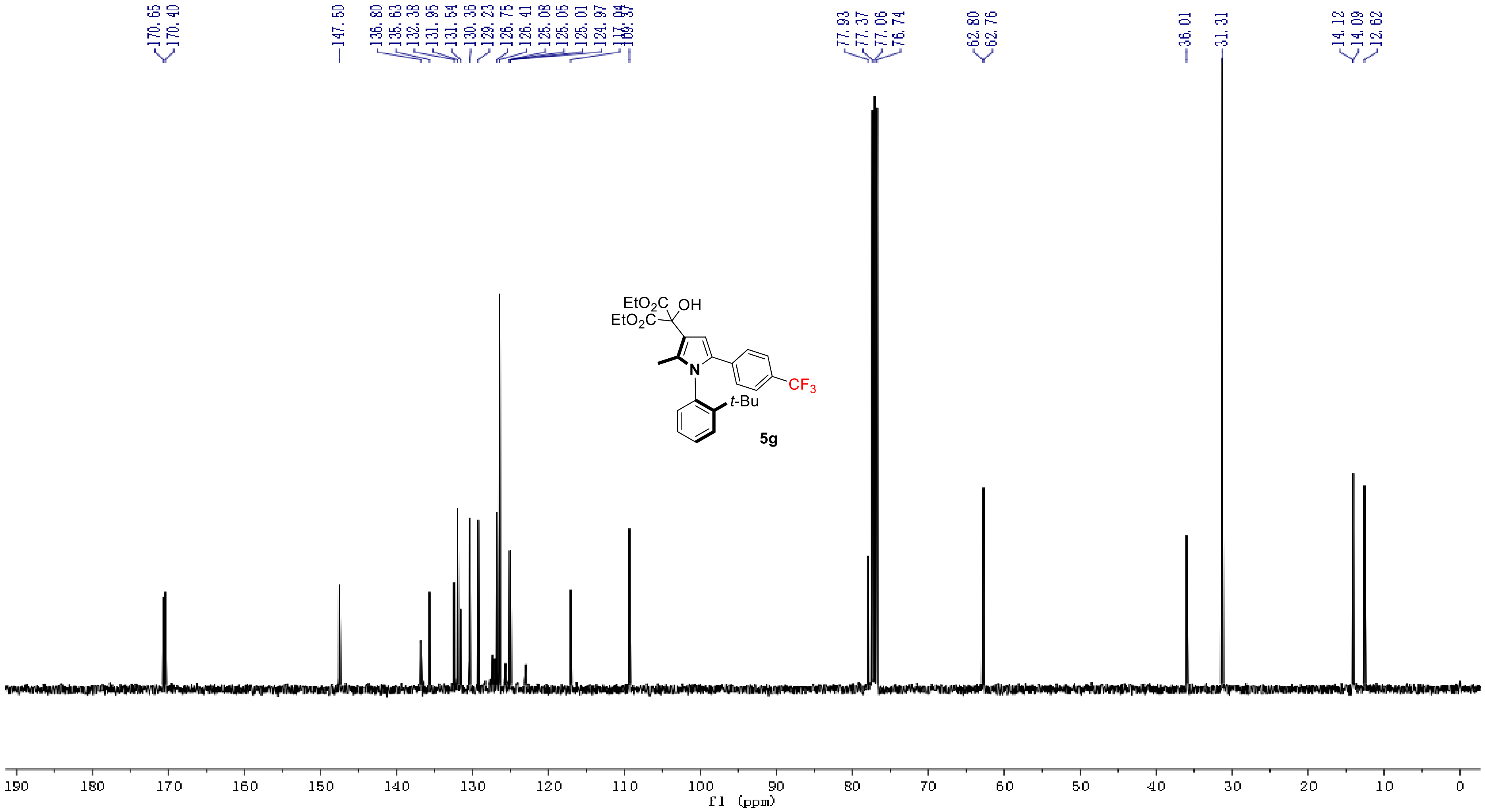


**Supplementary Figure 143.** $^{13}$C NMR of **5g**.

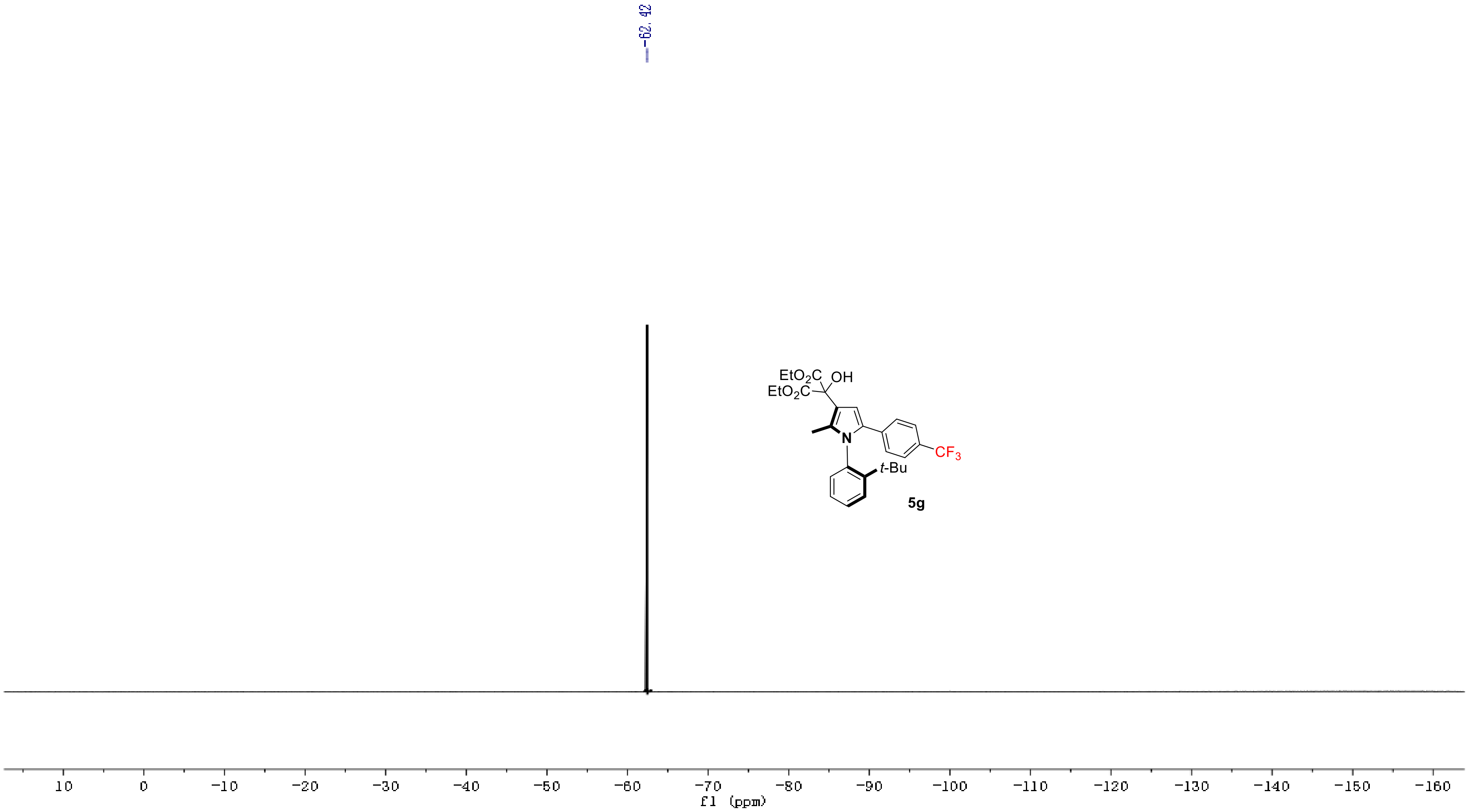


**Supplementary Figure 144.** $^{19}$F NMR of **5g**.

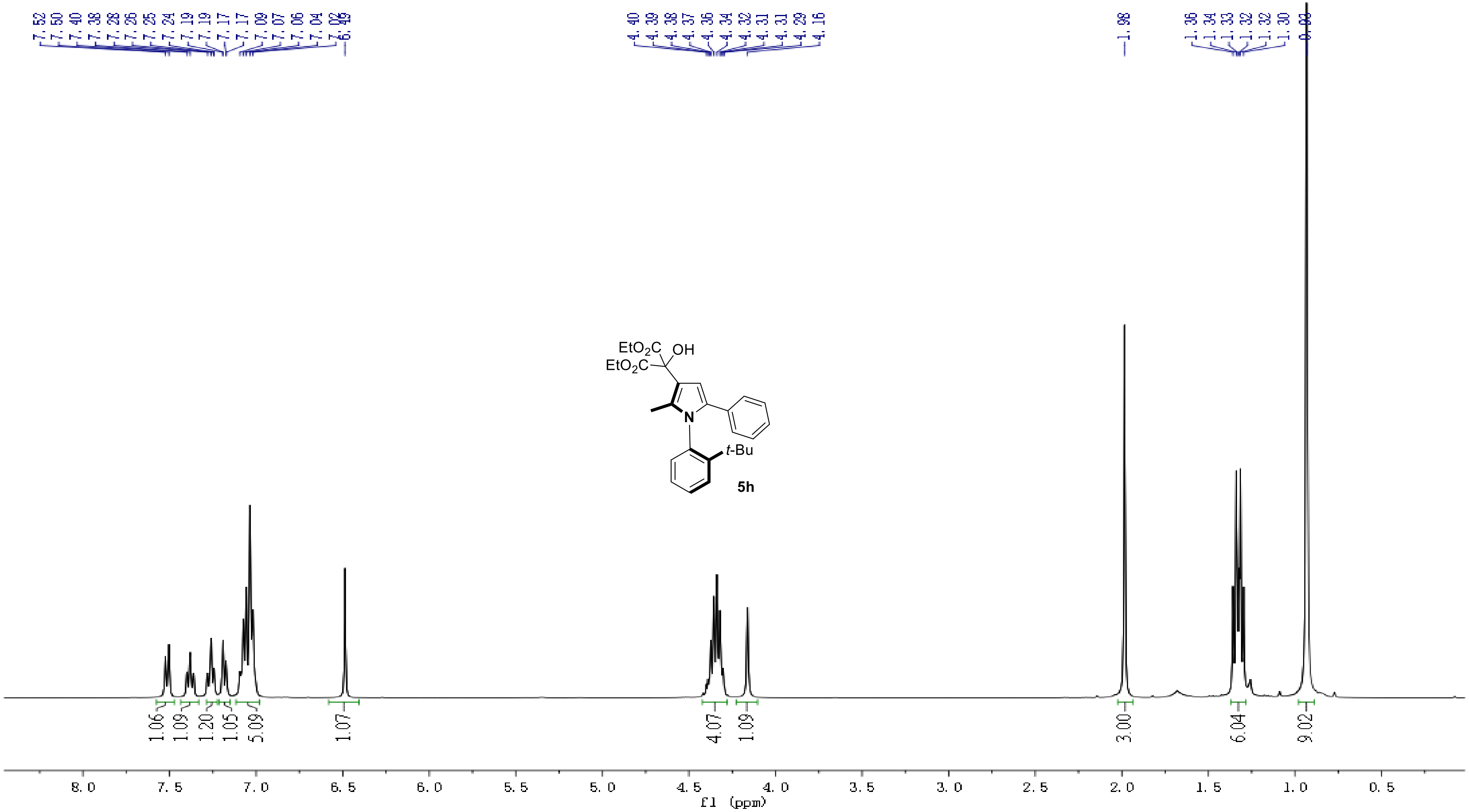


**Supplementary Figure 145.** $^{1}$H NMR of **5h**.

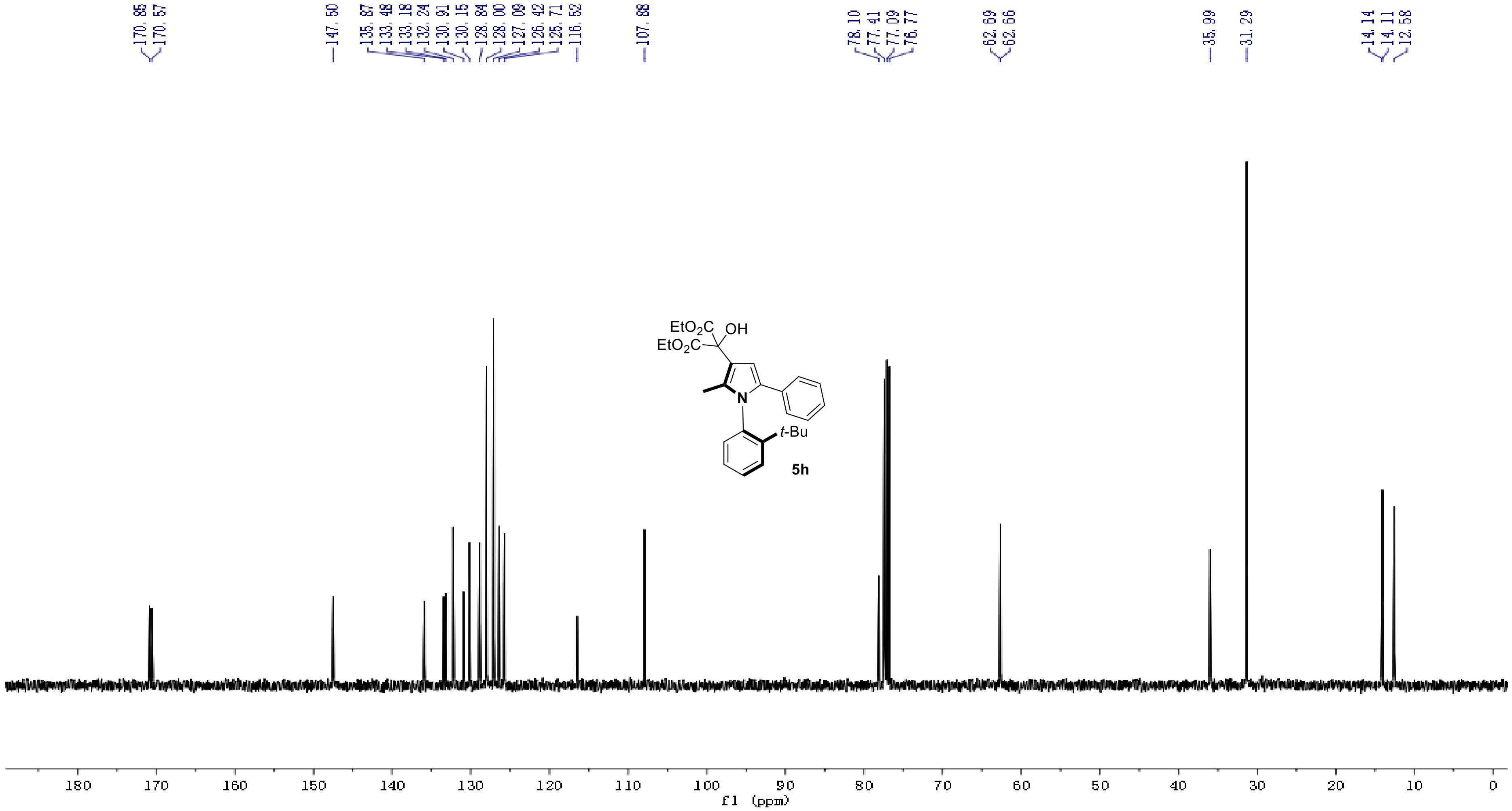


**Supplementary Figure 146.** $^{13}$C NMR of **5h**.

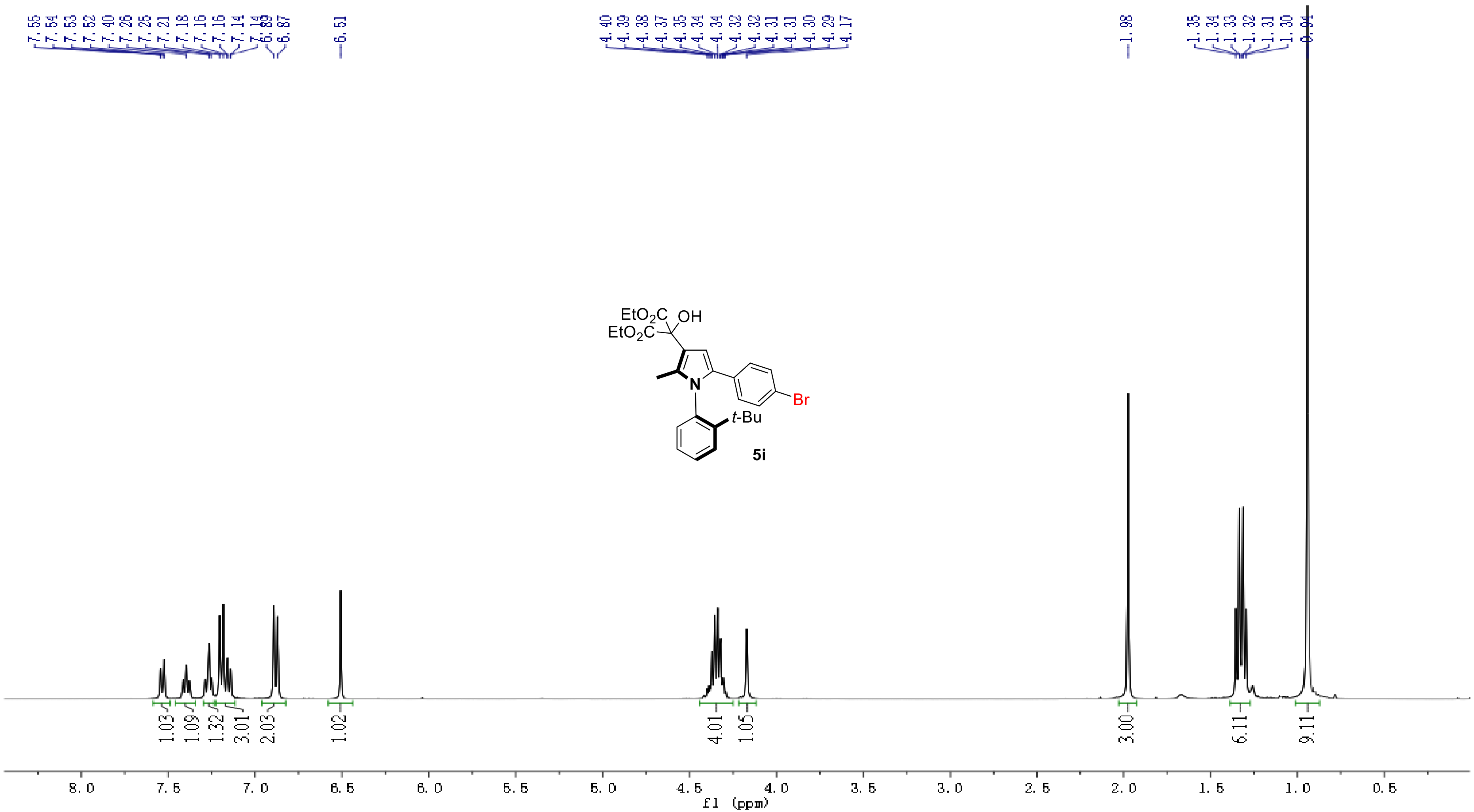


**Supplementary Figure 147.** $^{1}$H NMR of **5i**.

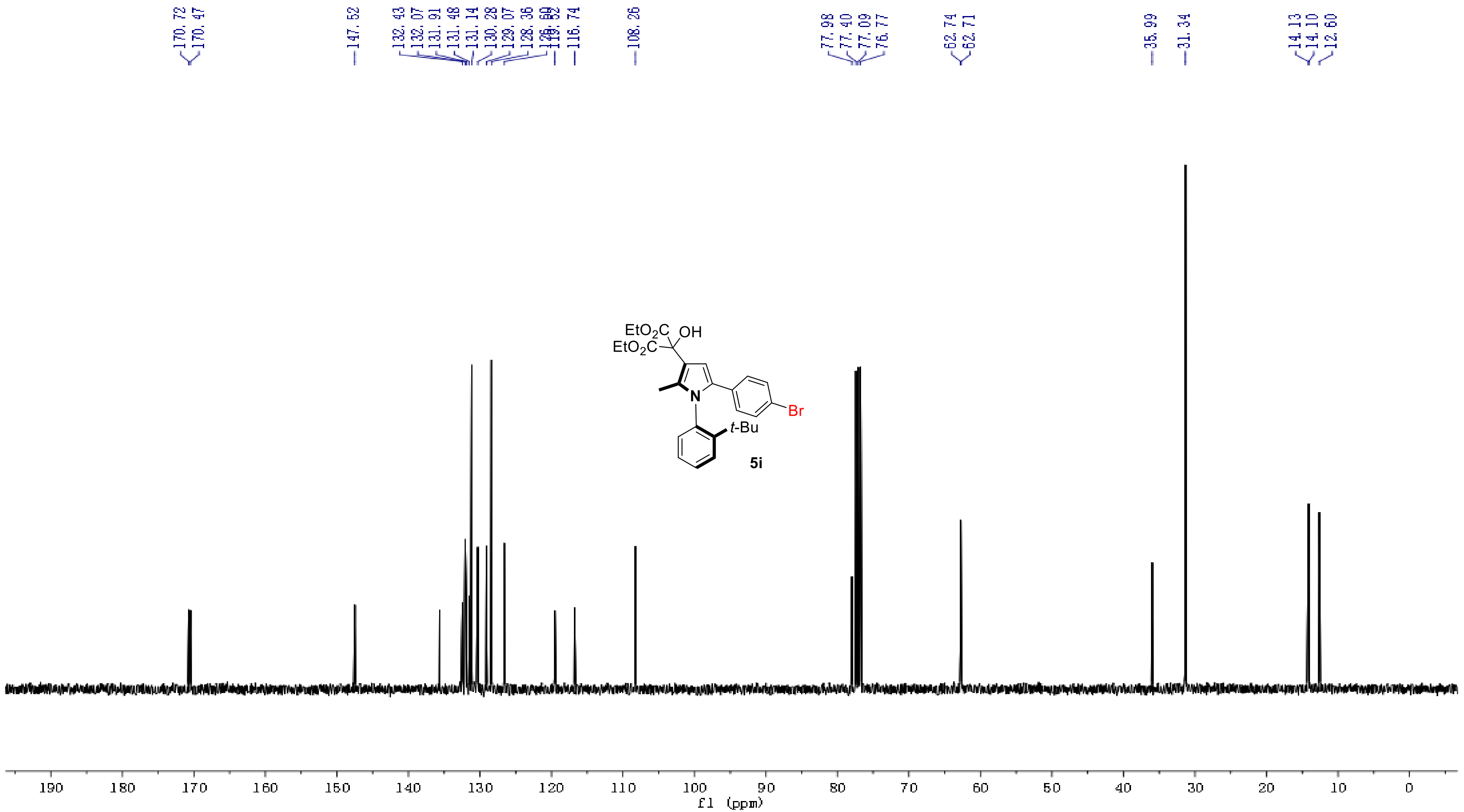


**Supplementary Figure 148.** $^{13}$C NMR of **5i**.

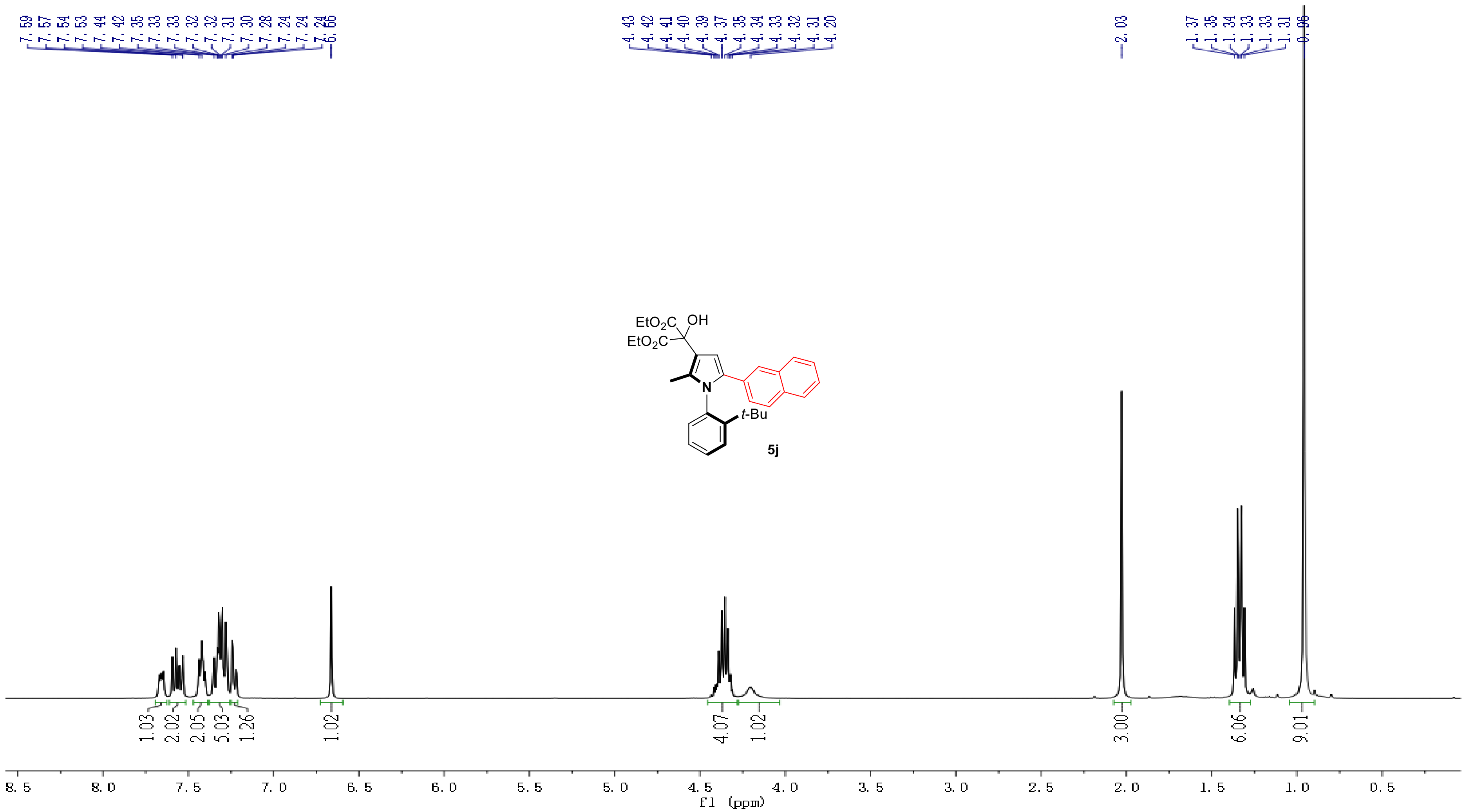

**Supplementary Figure 149.** $^{1}$H NMR of **5j**.

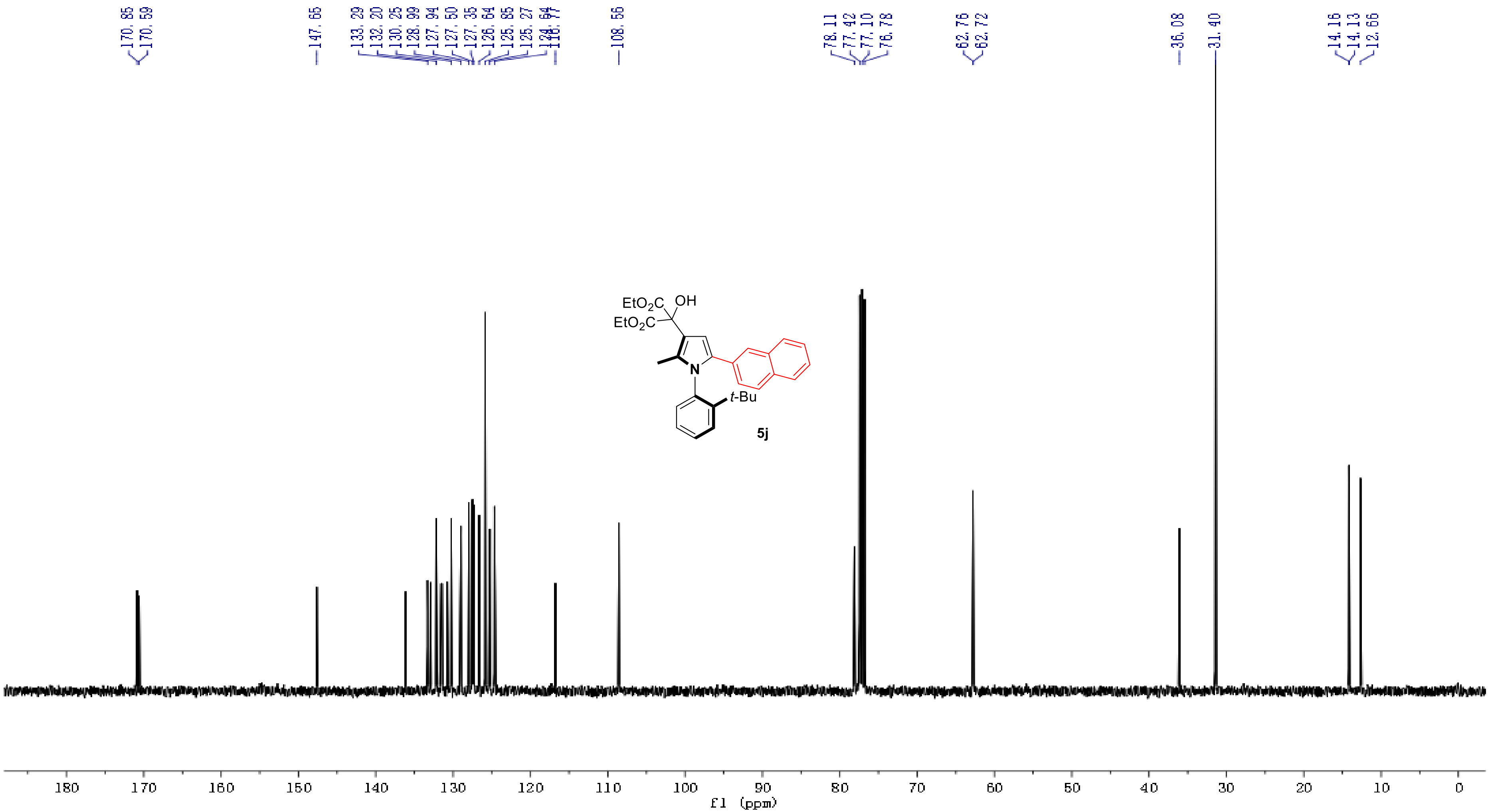


**Supplementary Figure 150.** $^{13}$C NMR of **5j**.

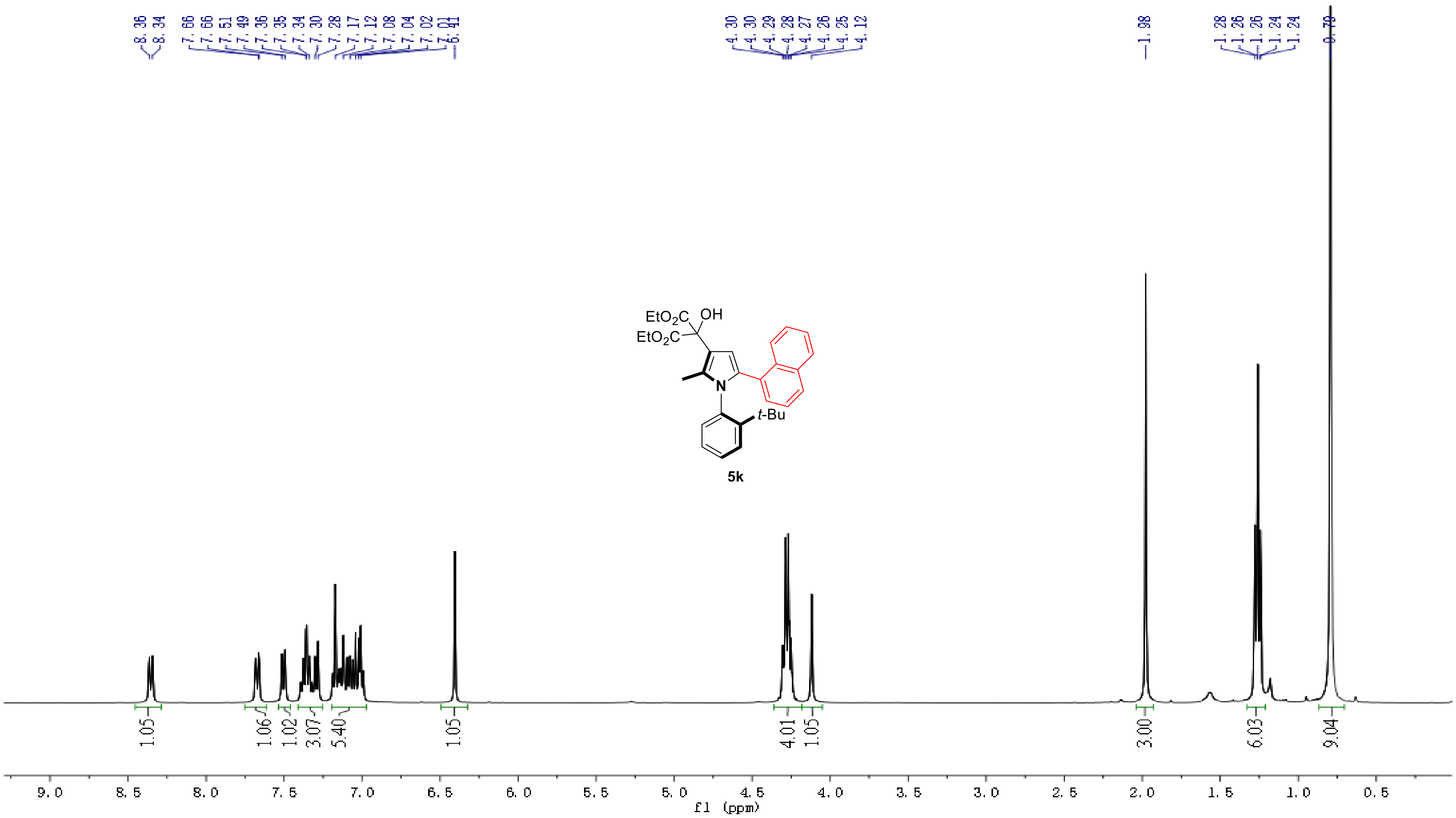


**Supplementary Figure 151.** $^{1}$H NMR of **5k**.

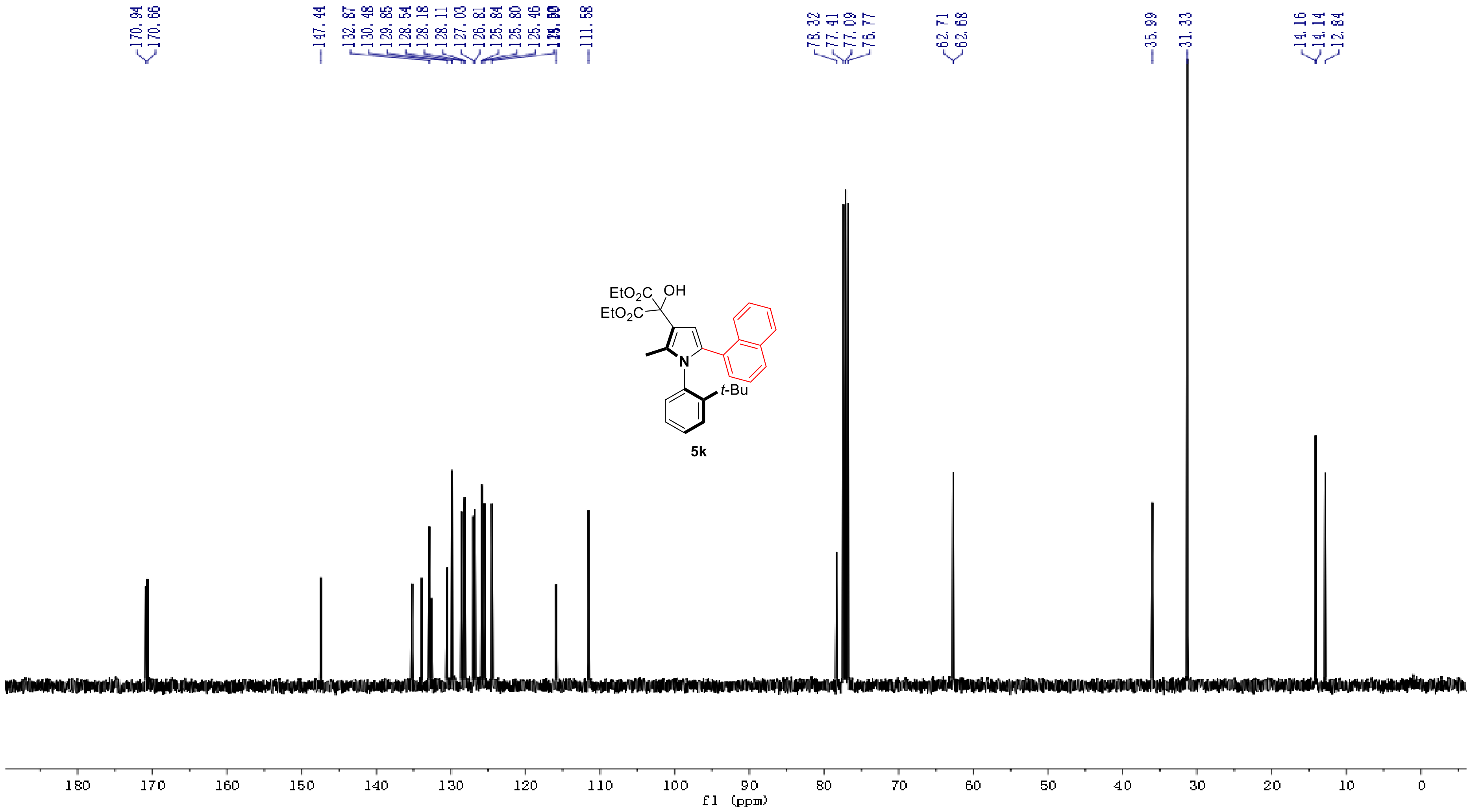


**Supplementary Figure 152.** $^{13}$C NMR of **5k**.

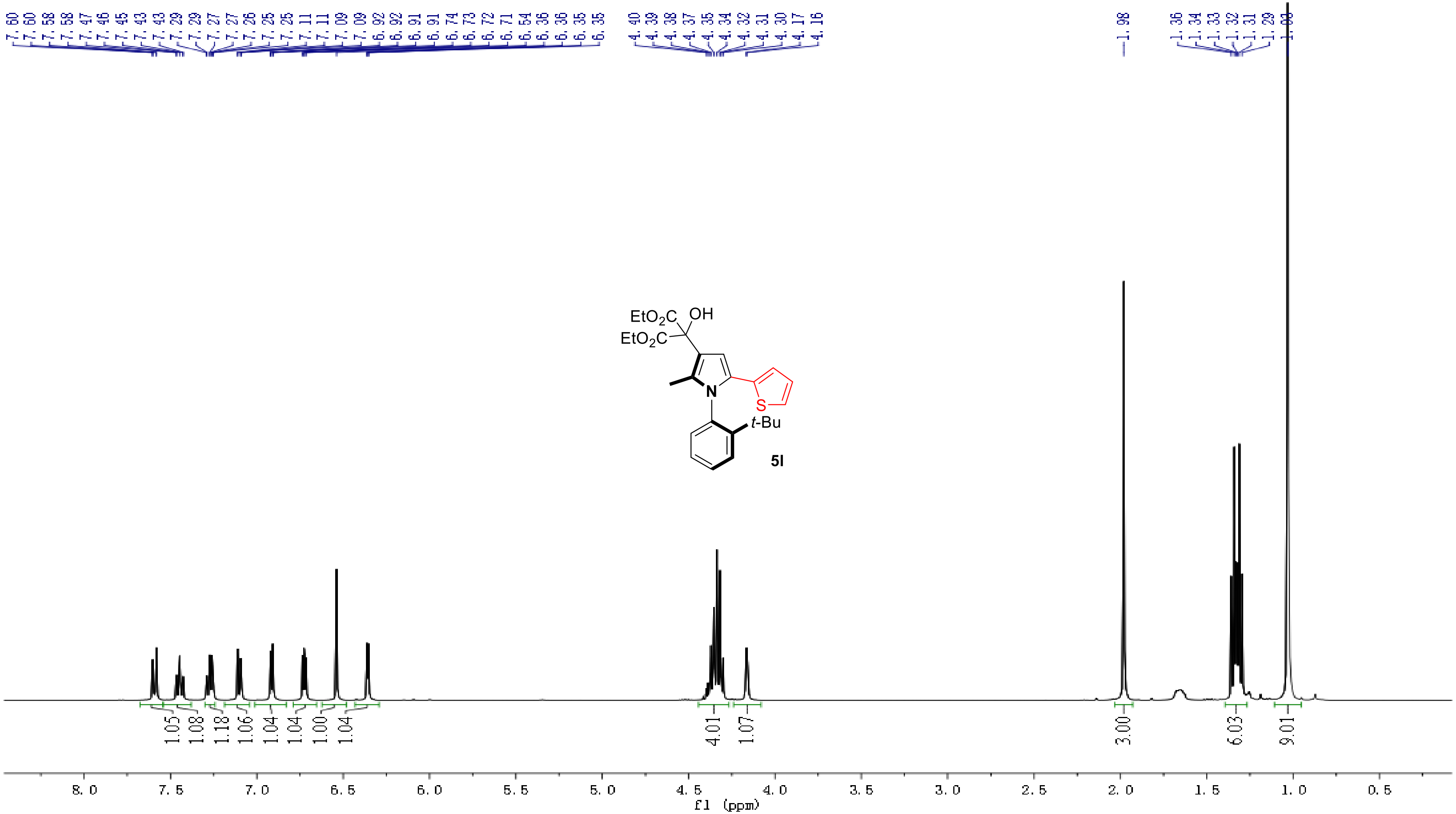


**Supplementary Figure 153.** $^{1}$H NMR of **5l**.

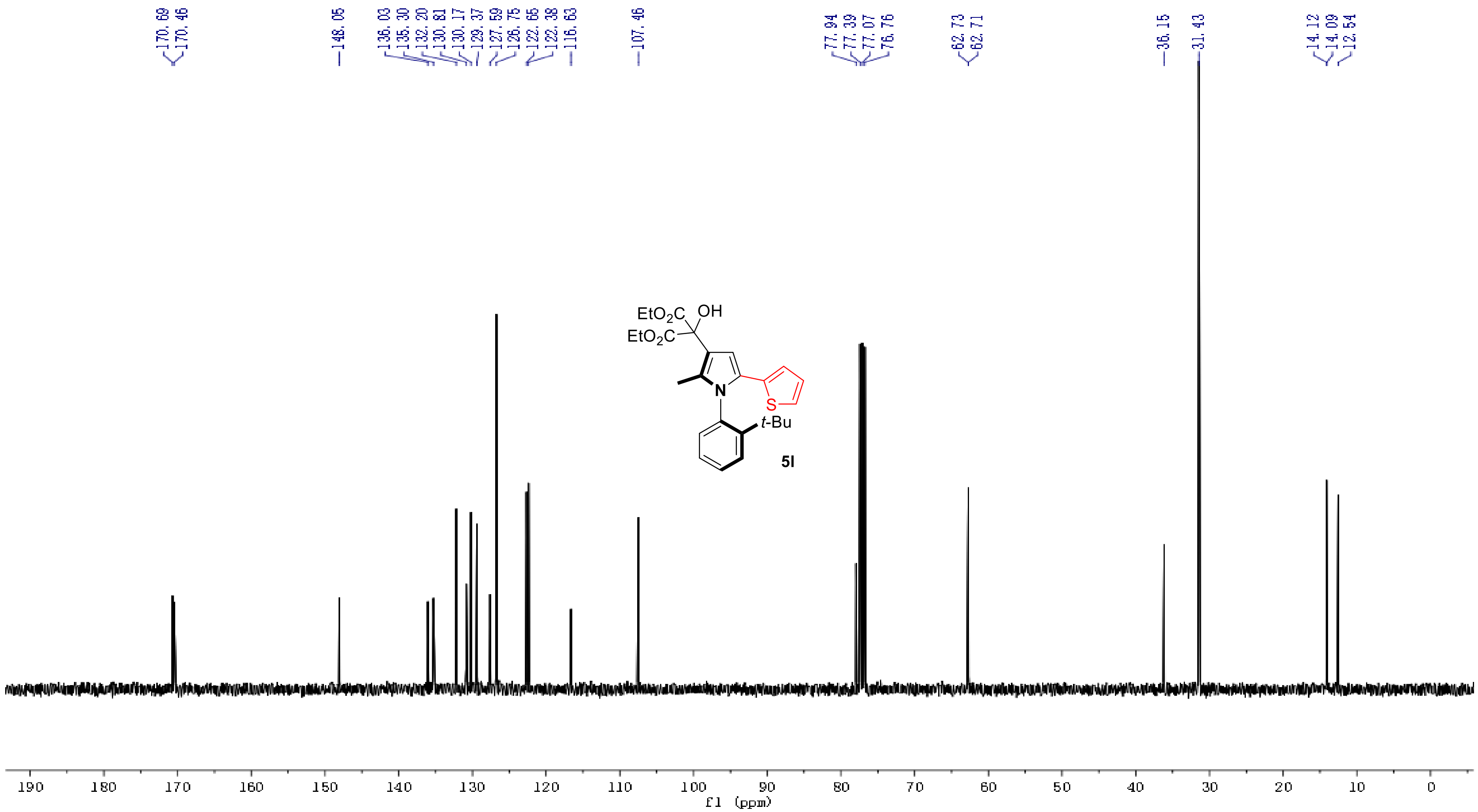


**Supplementary Figure 154.** $^{13}$C NMR of **5l**.

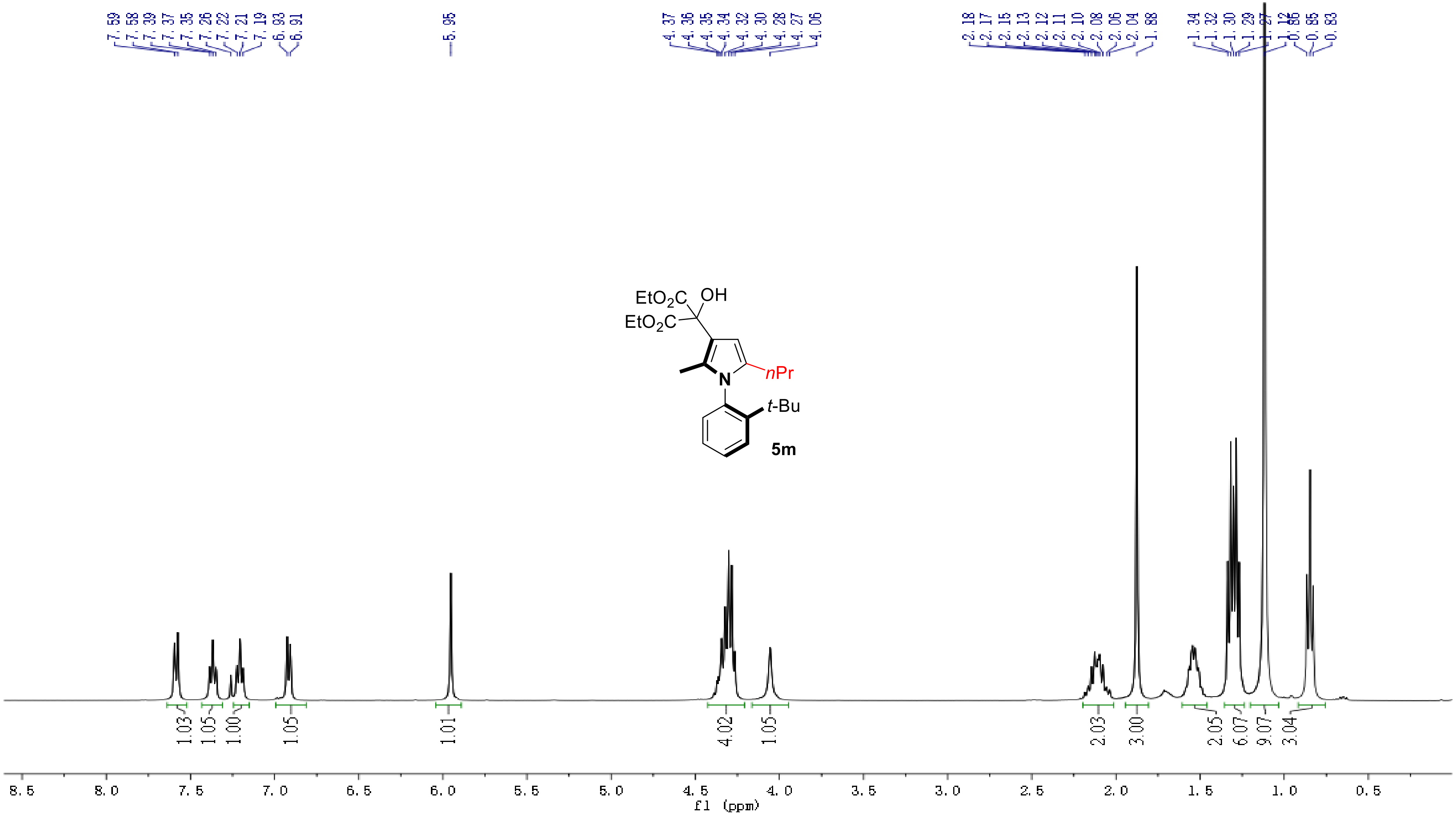


**Supplementary Figure 155.** $^1$H NMR of **5m**.

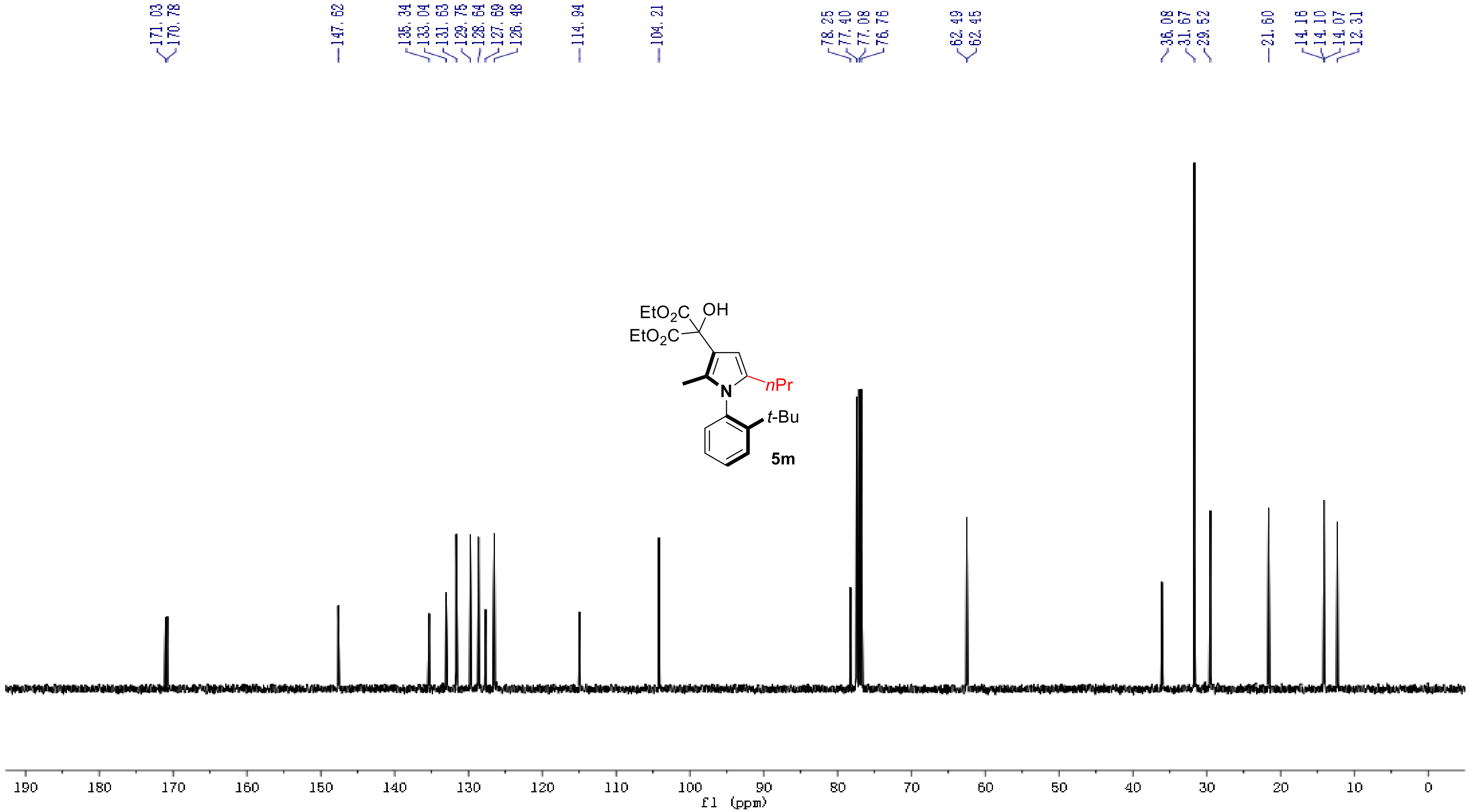


**Supplementary Figure 156.** $^{13}$C NMR of **5m**.

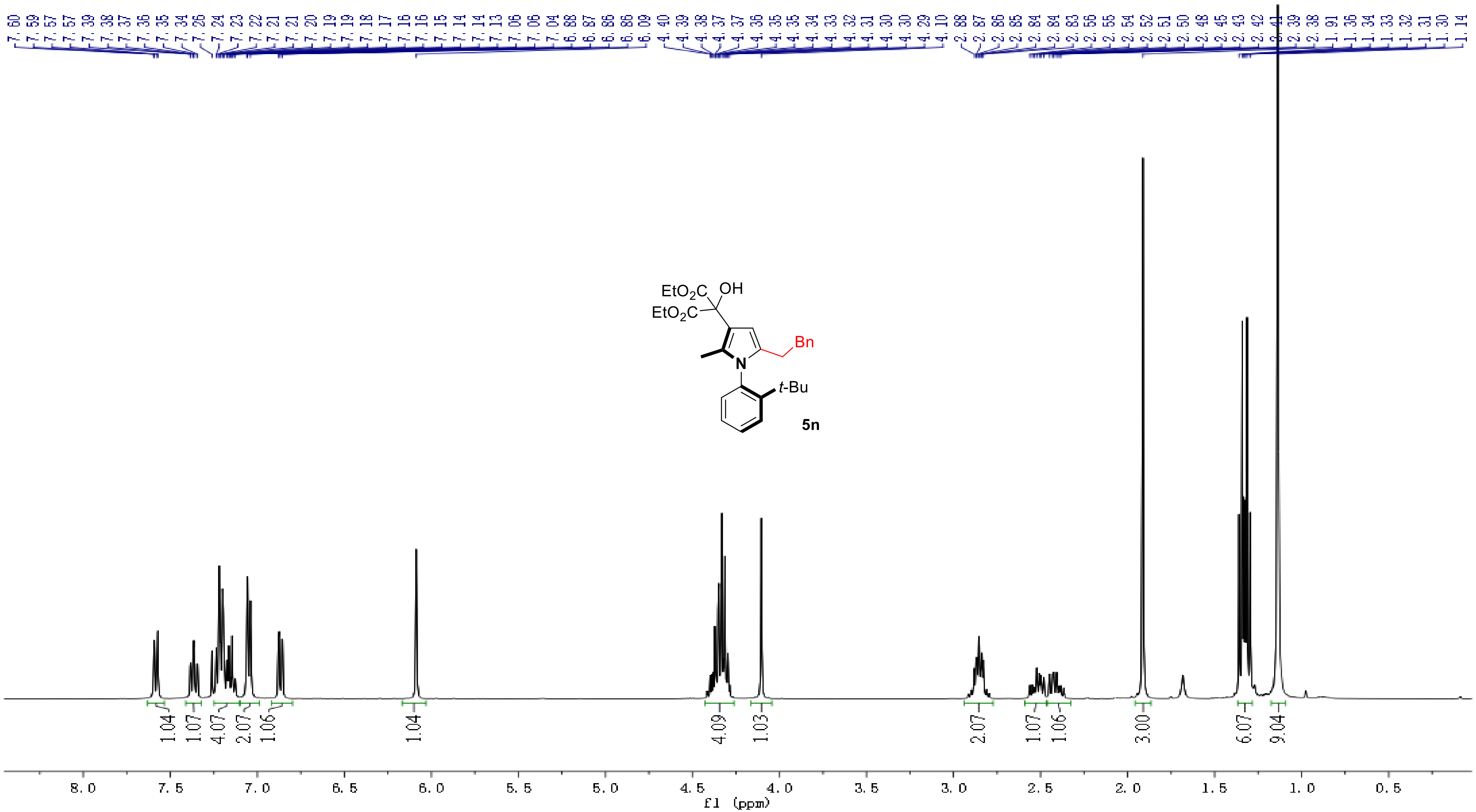


**Supplementary Figure 157.** $^{1}$H NMR of **5n**.

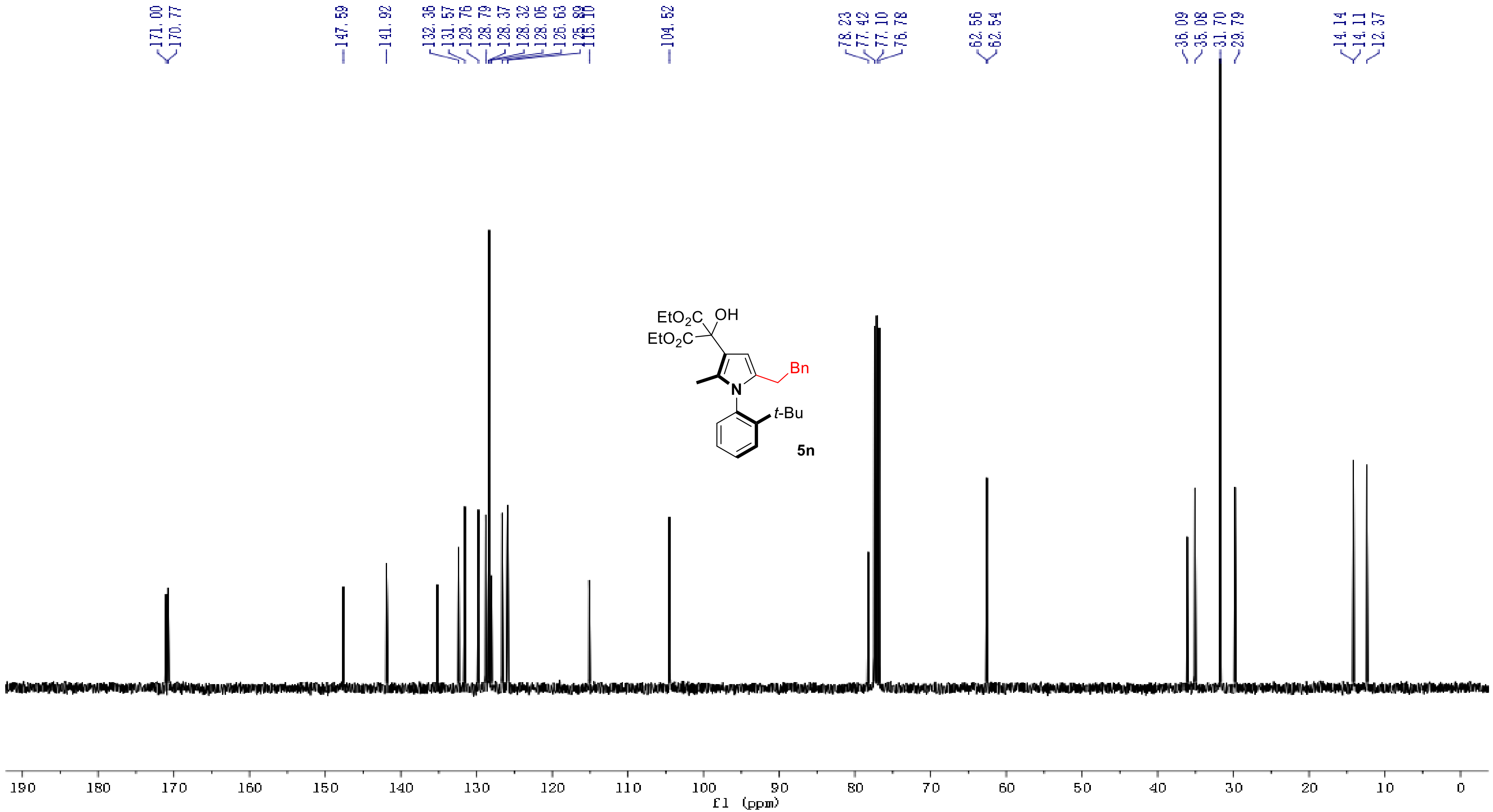


**Supplementary Figure 158.** $^{13}$C NMR of **5n**.

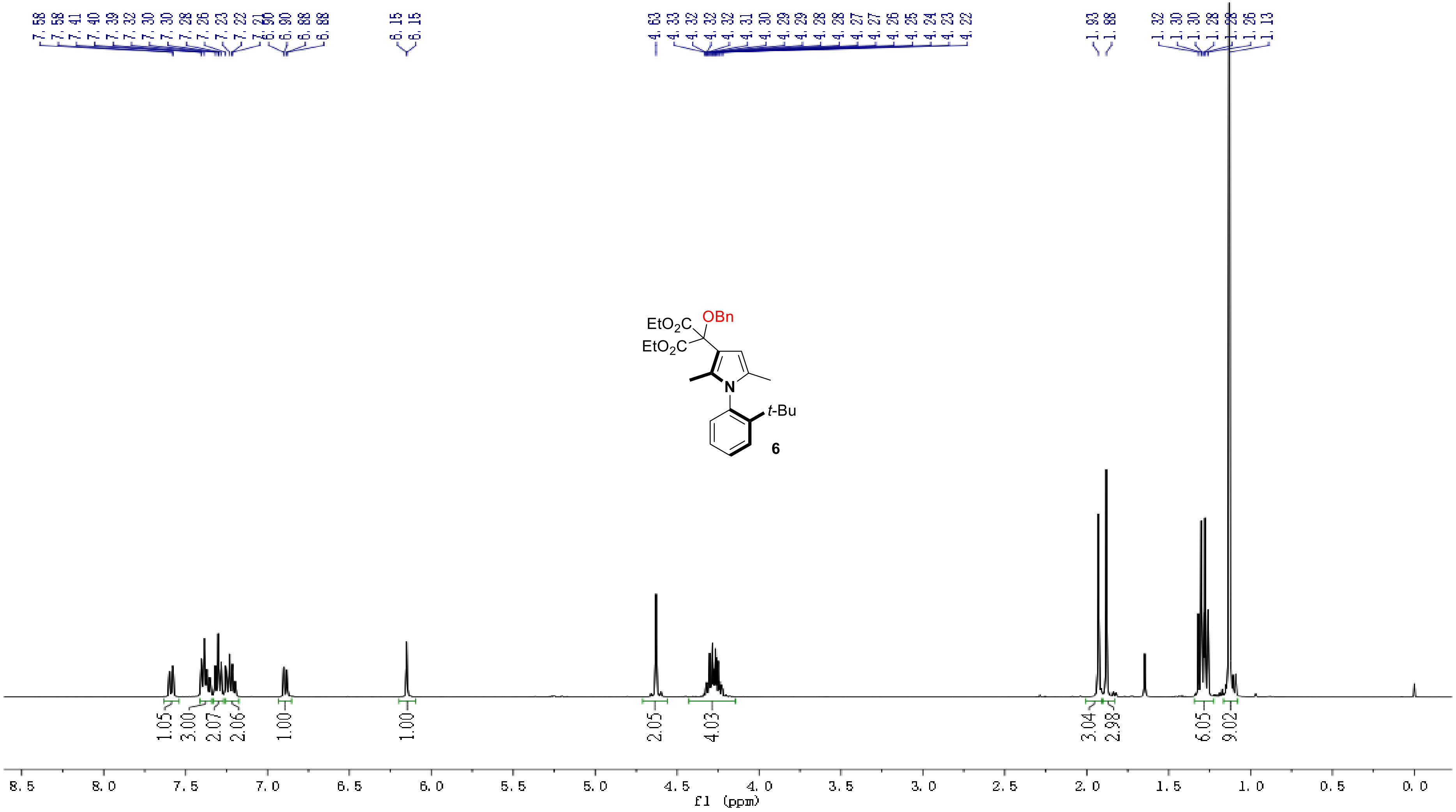


**Supplementary Figure 159.** $^{1}$H NMR of **6**.

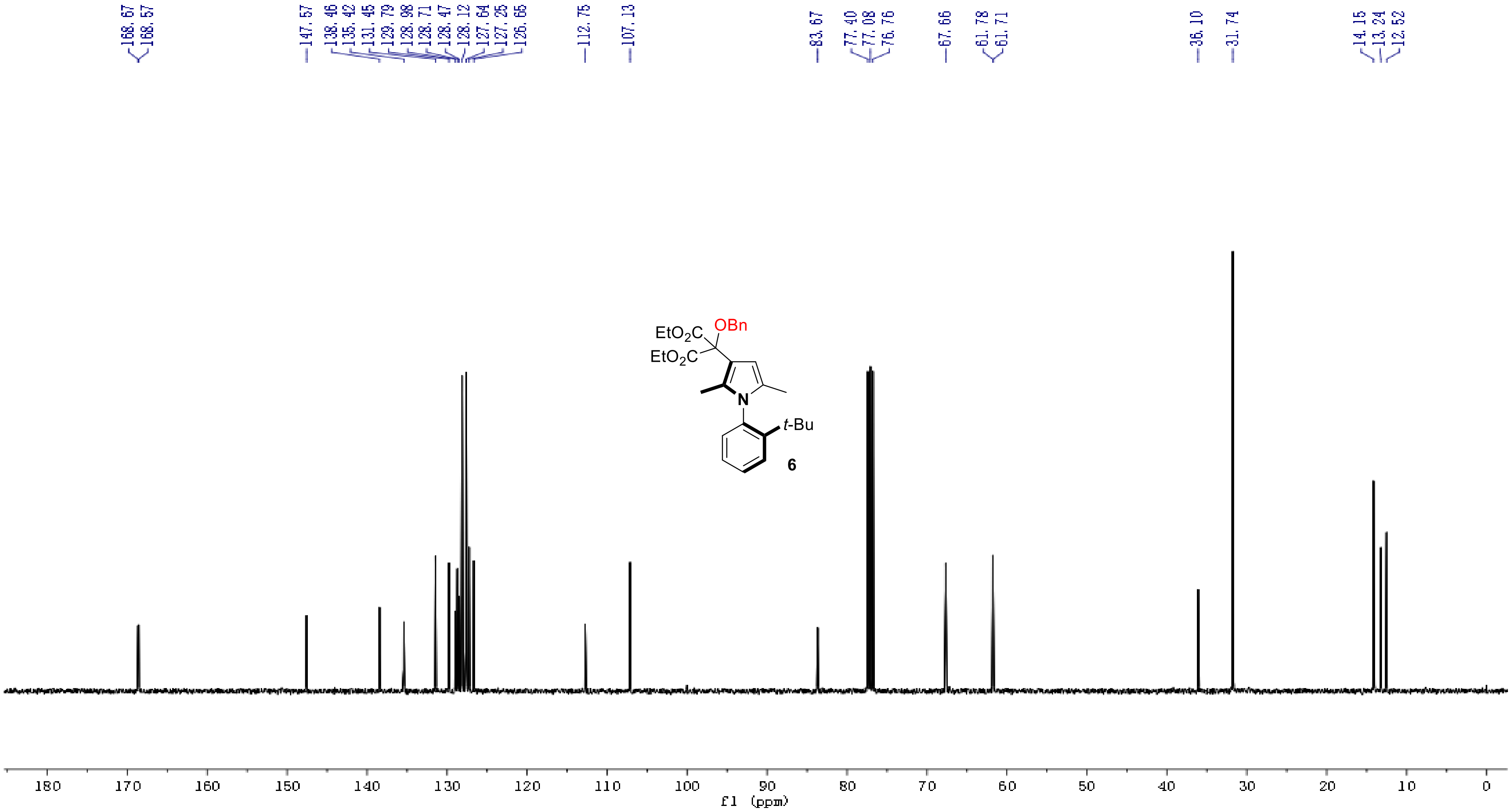


**Supplementary Figure 160.** $^{13}$C NMR of **6**.

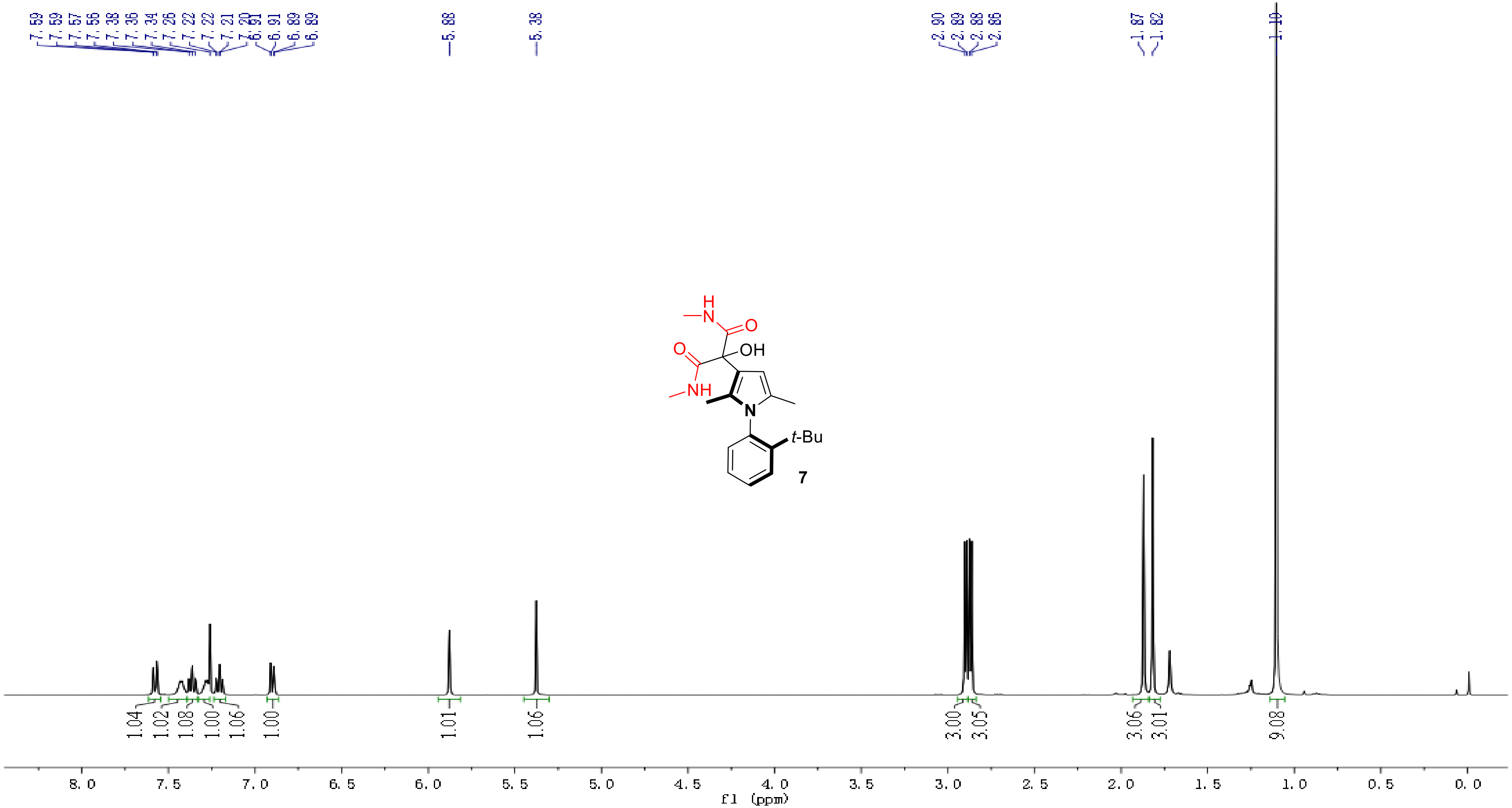

**Supplementary Figure 161.** $^{1}$H NMR of **7**.

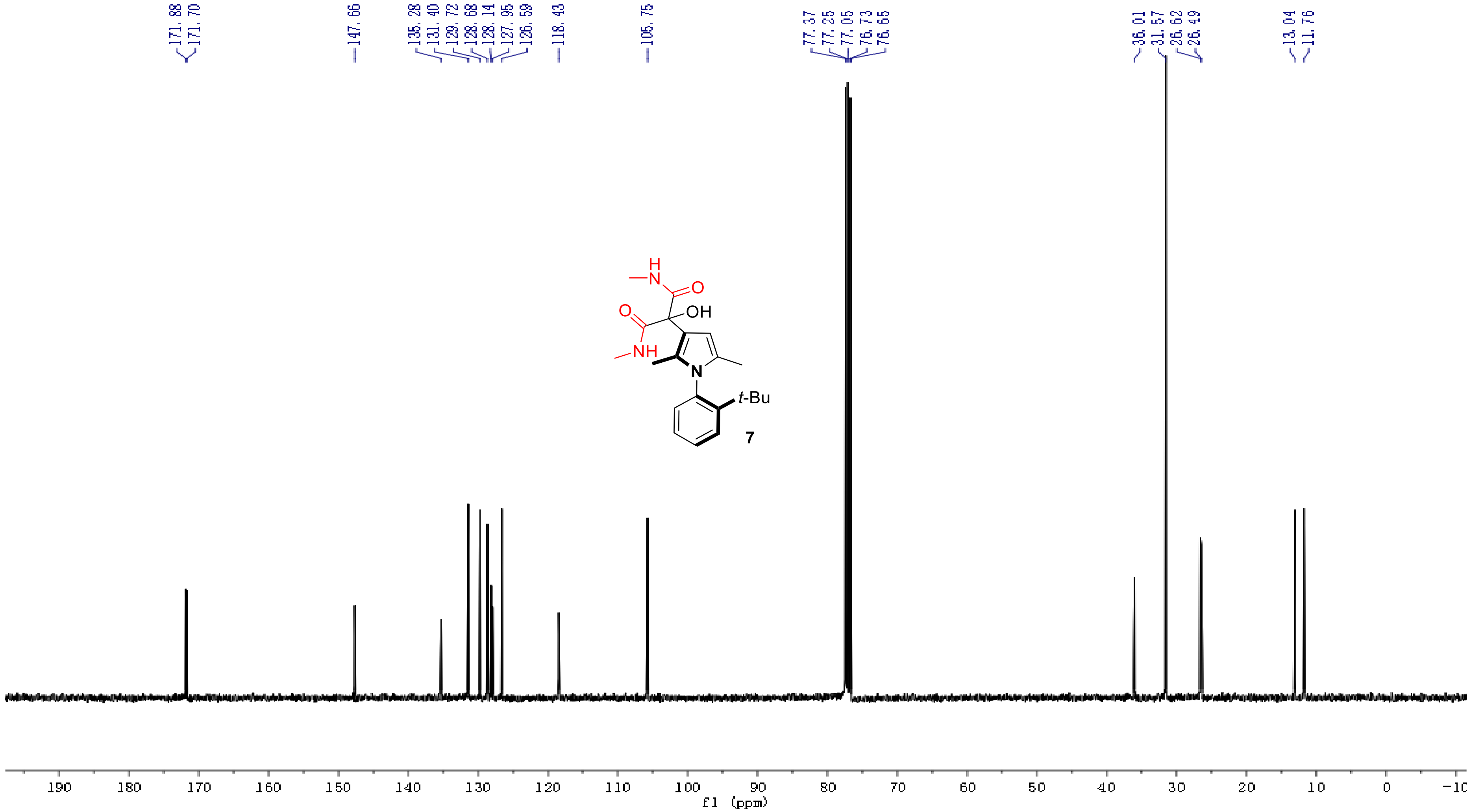


**Supplementary Figure 162.** $^{13}$C NMR of **7**.

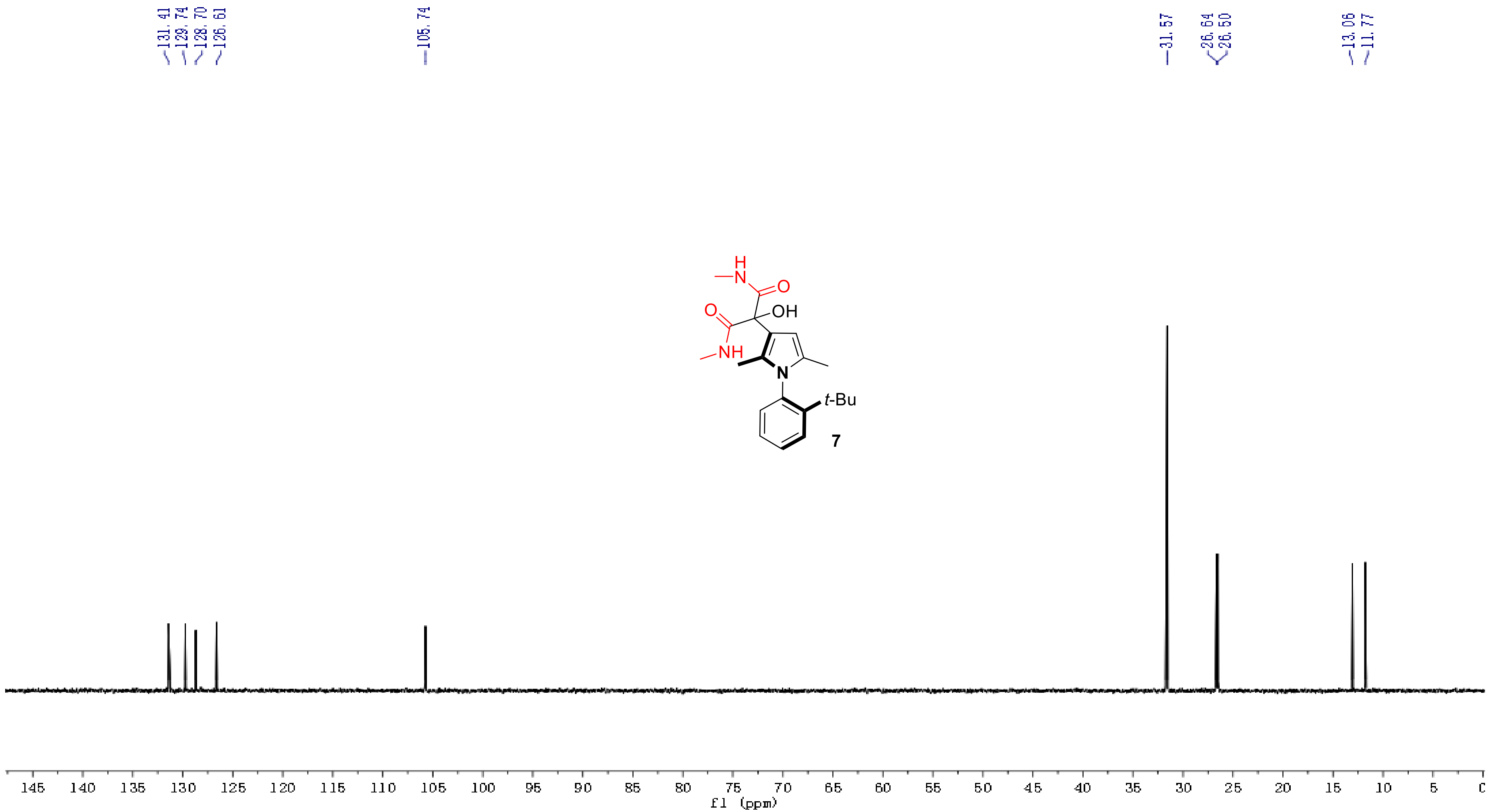


**Supplementary Figure 163.** $^{13}$C NMR-DEPT 135 of **7**.

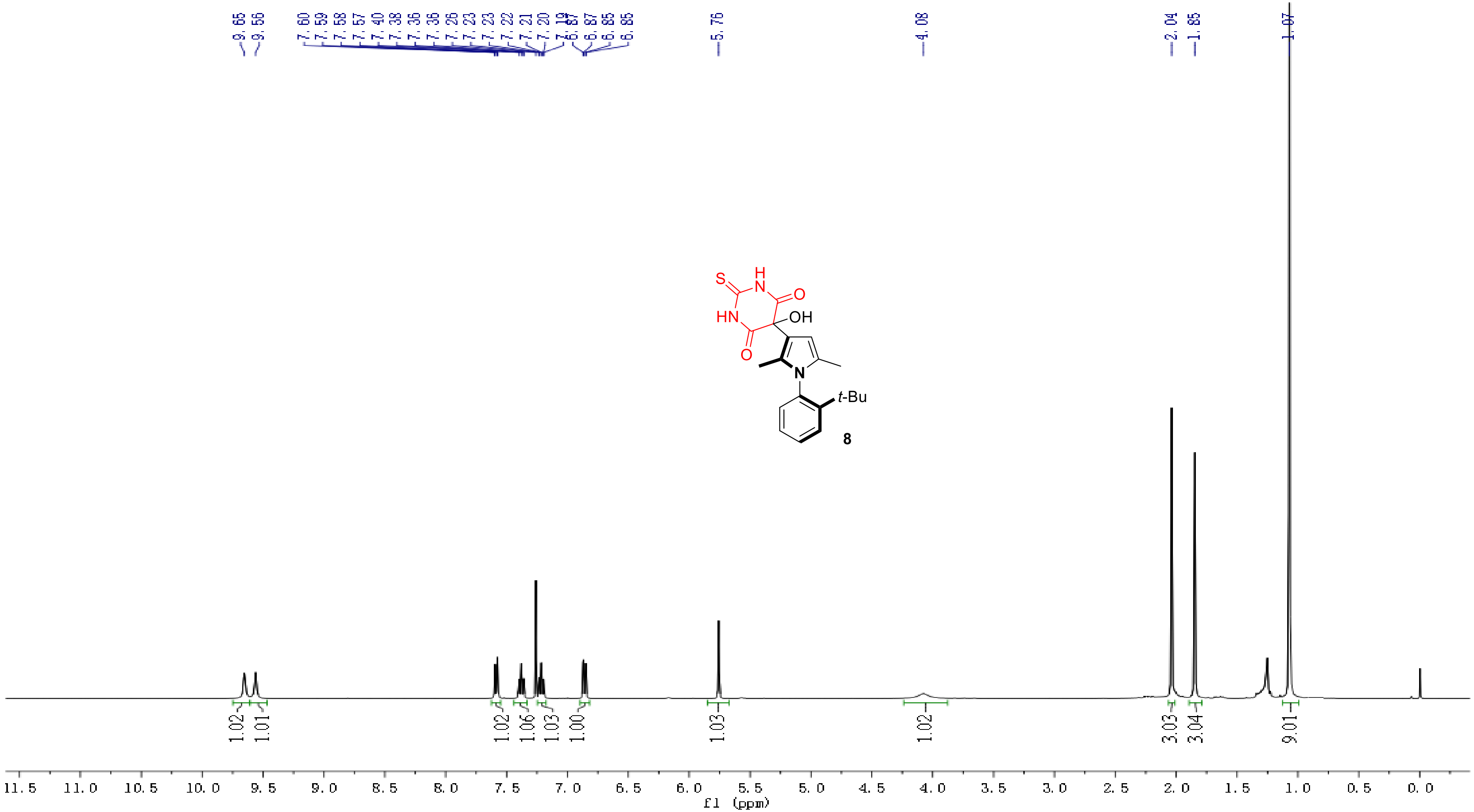


**Supplementary Figure 164.** $^{1}$H NMR of **8**.

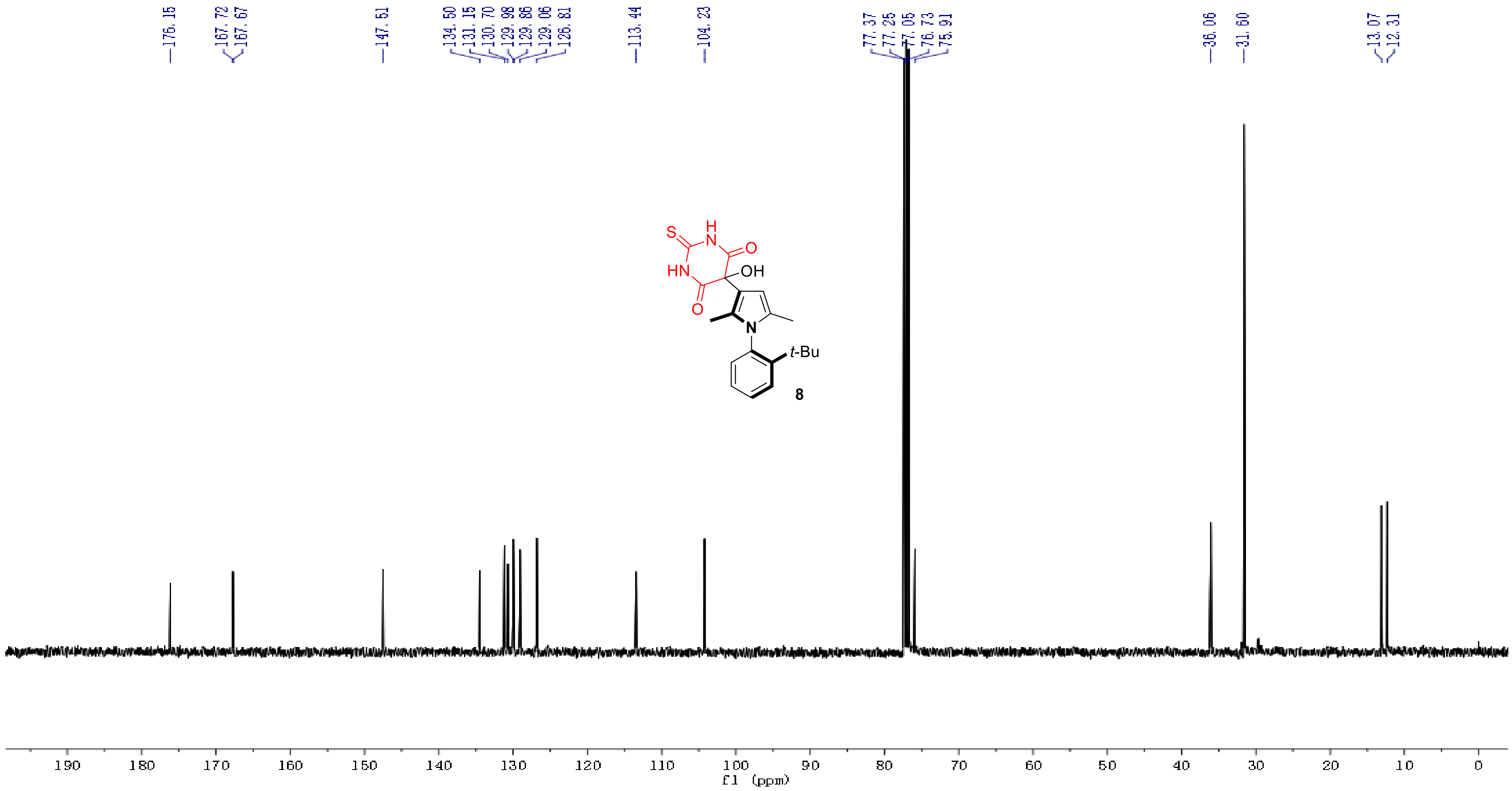


**Supplementary Figure 165.** $^{13}$C NMR of **8**.

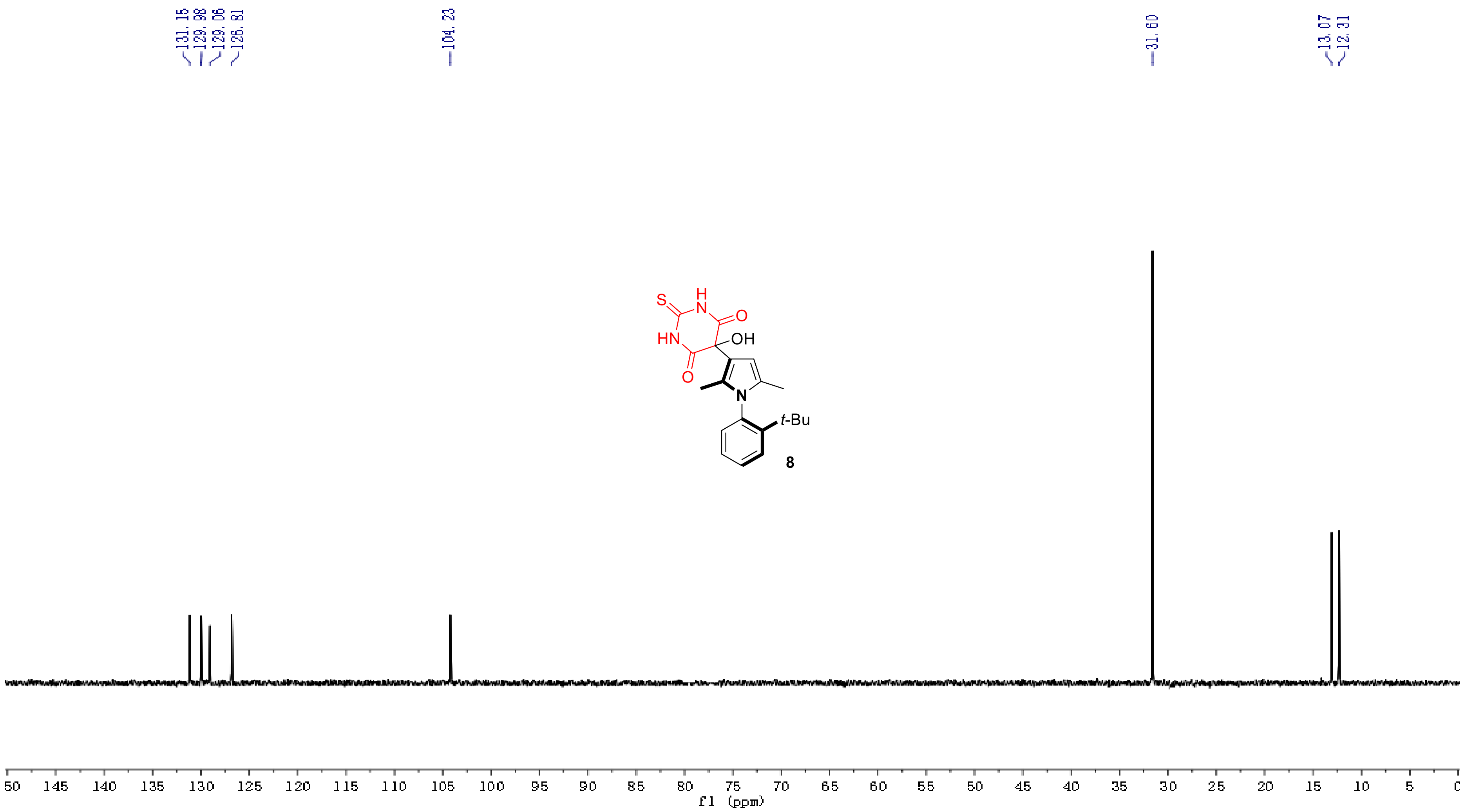


**Supplementary Figure 166.** $^{13}C$ NMR-DEPT 135 of **8**.

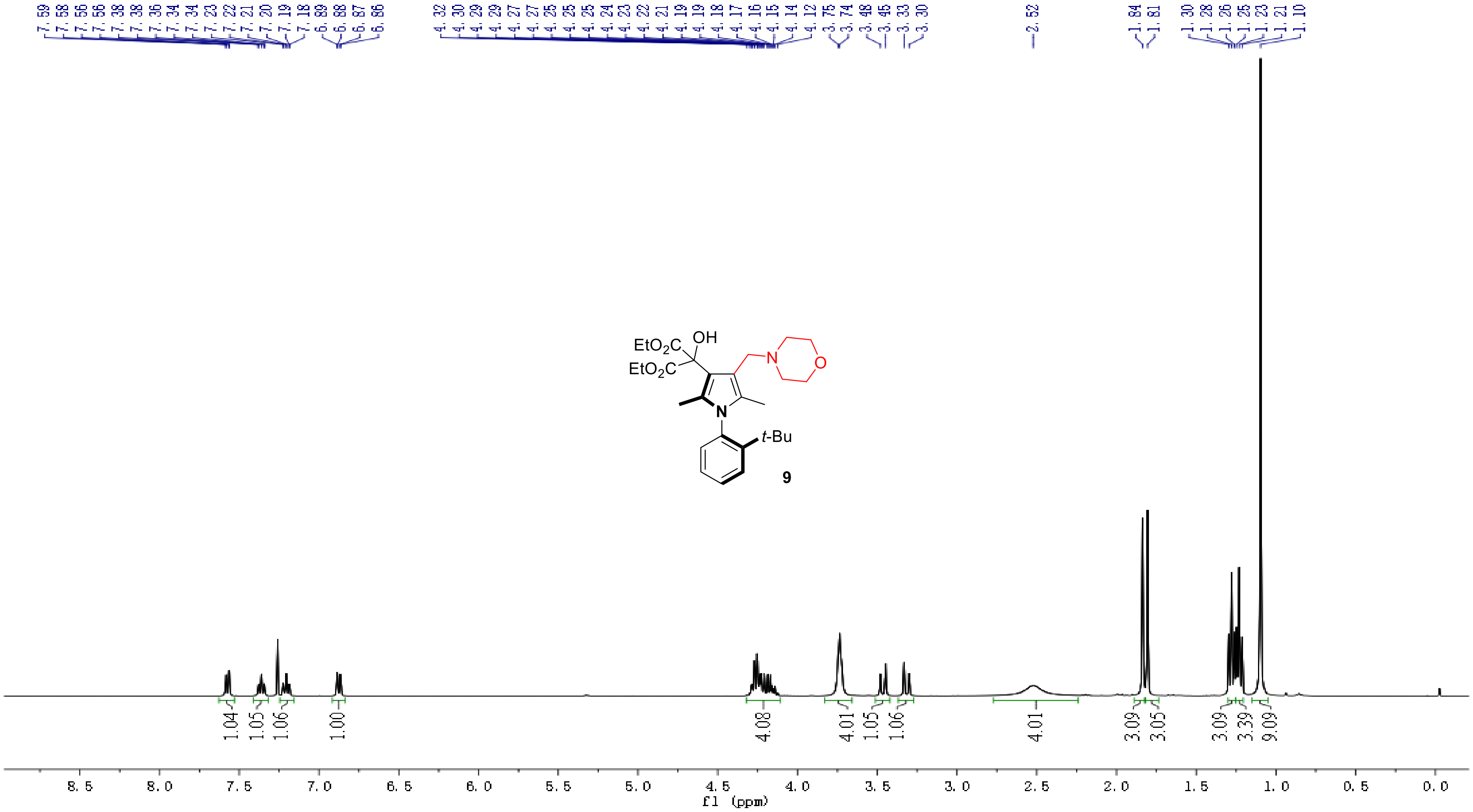


**Supplementary Figure 167.** $^{1}$H NMR of **9**.

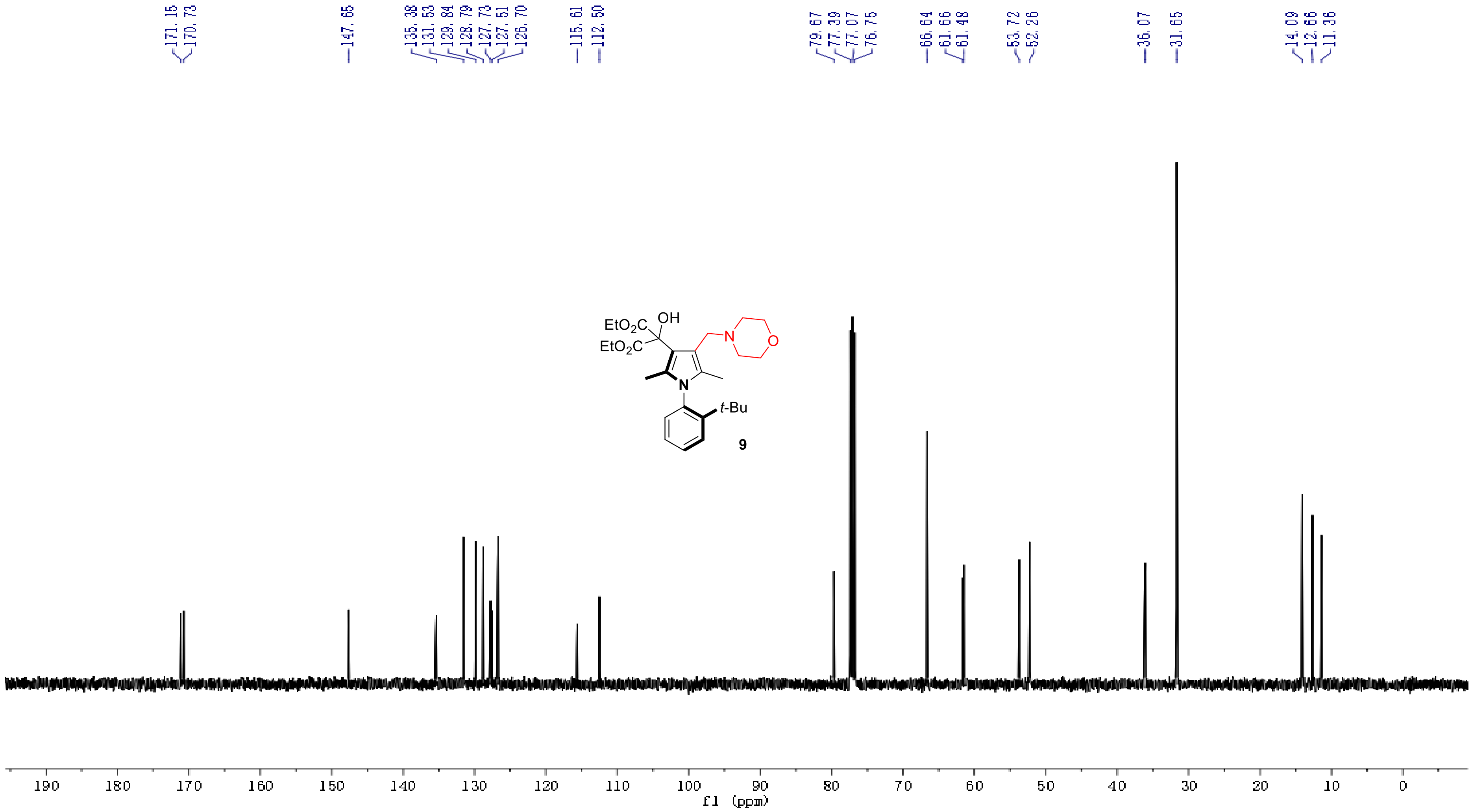


**Supplementary Figure 168.** $^{13}$C NMR of **9**.

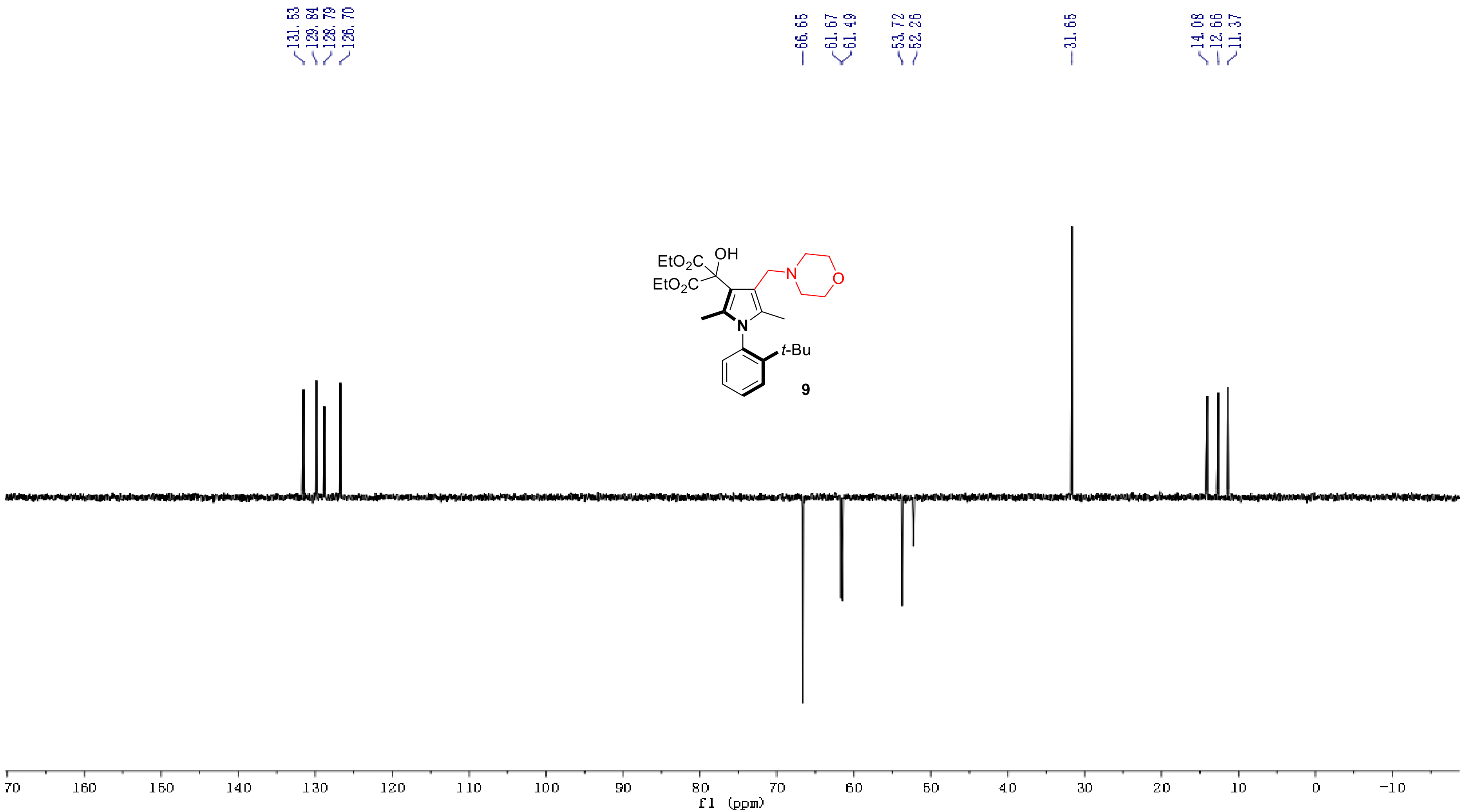


**Supplementary Figure 169.** $^{13}$C NMR-DEPT 135 of **9**.

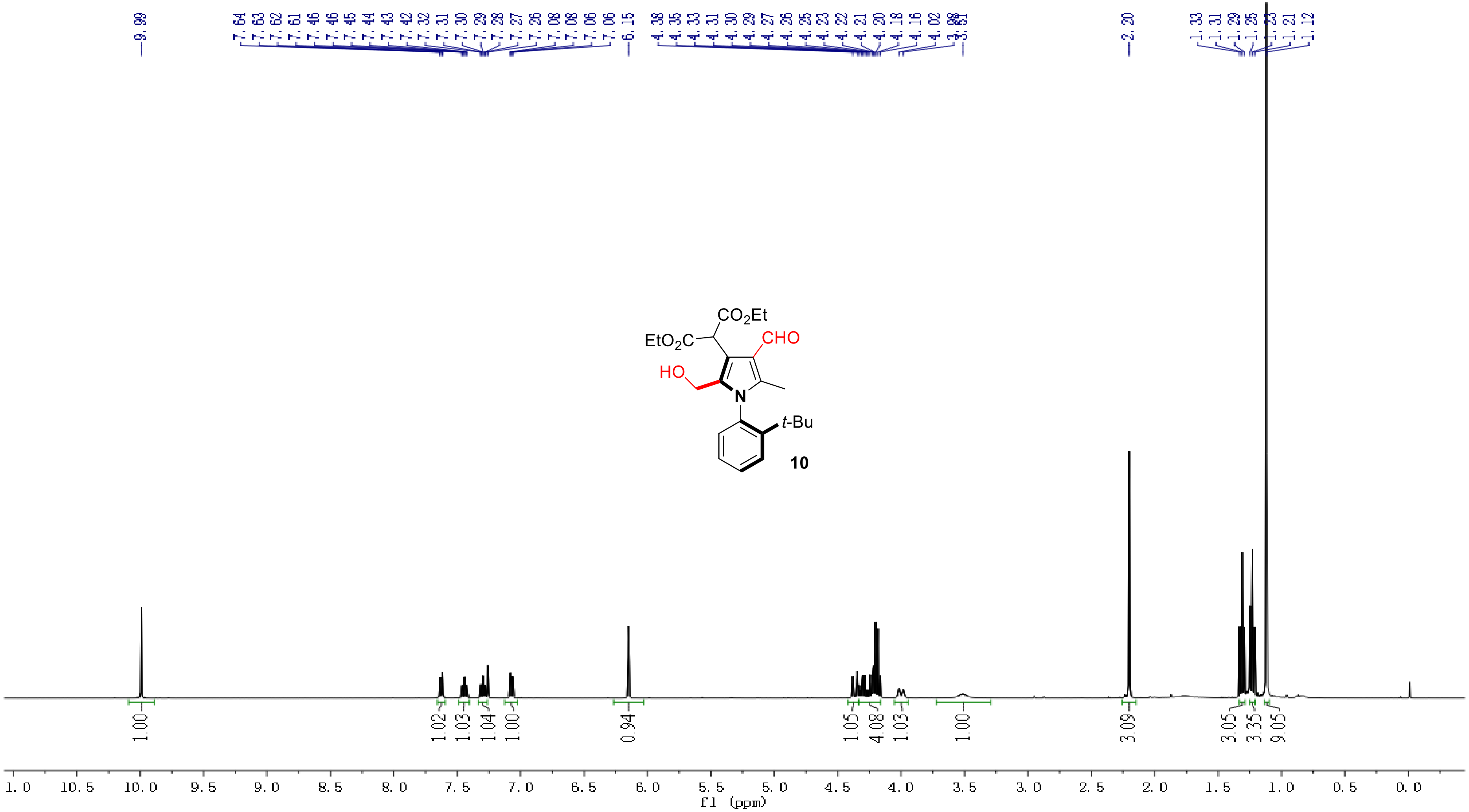


**Supplementary Figure 170.** $^{1}$H NMR of **10**.

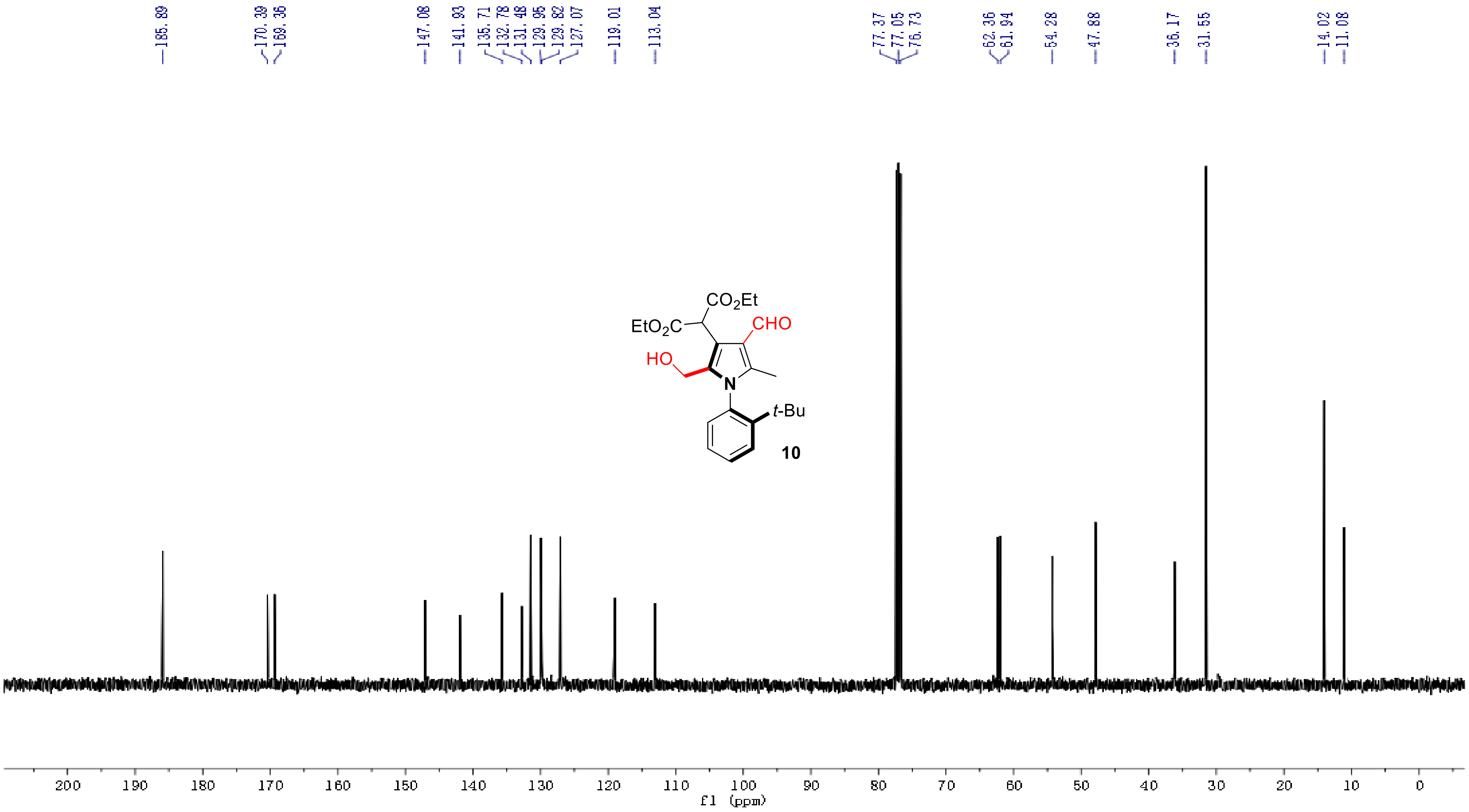


**Supplementary Figure 171.** $^{13}$C NMR of **10**.

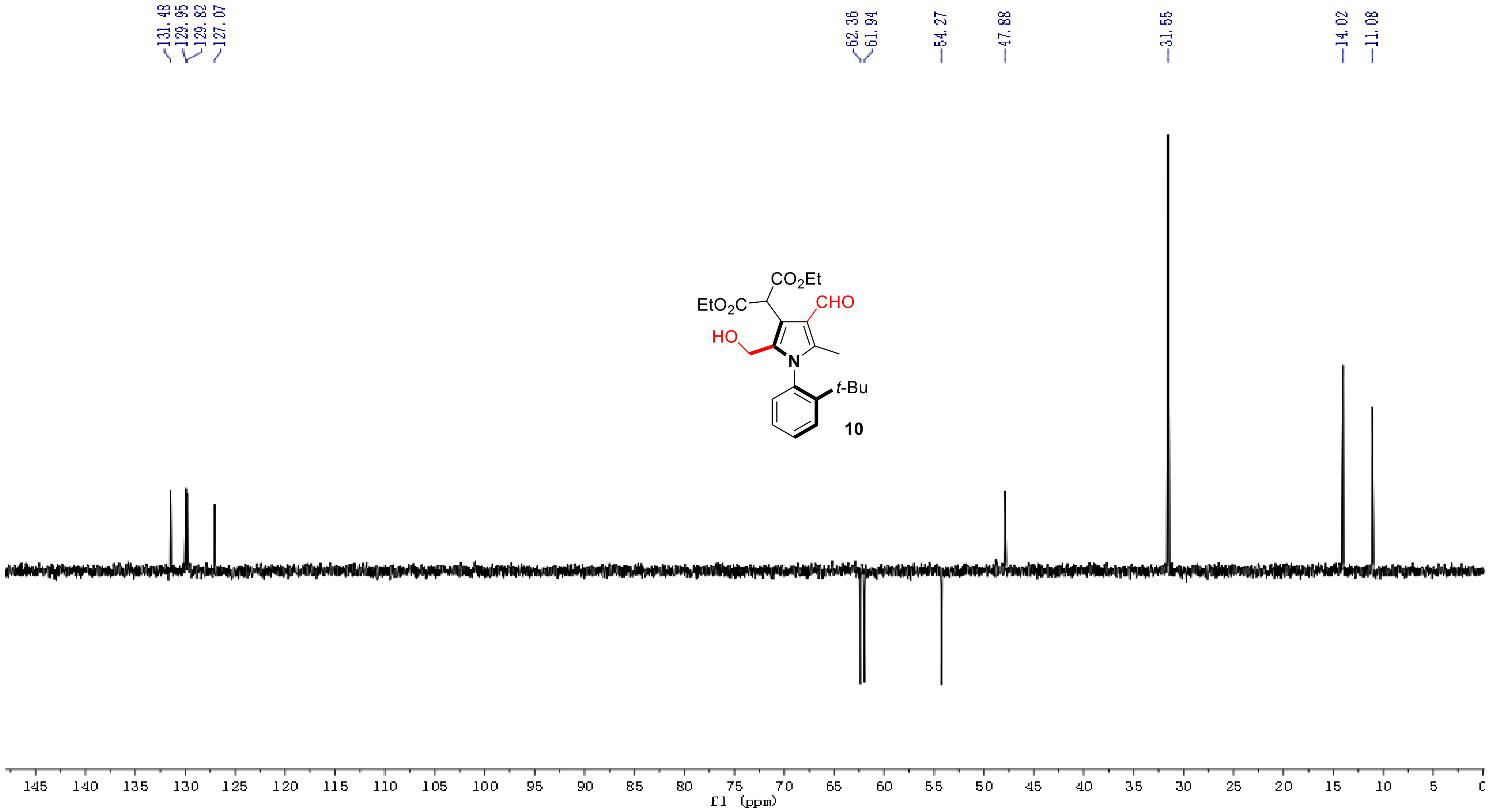


**Supplementary Figure 172.** $^{13}C$ NMR-DEPT 135 of **10**.

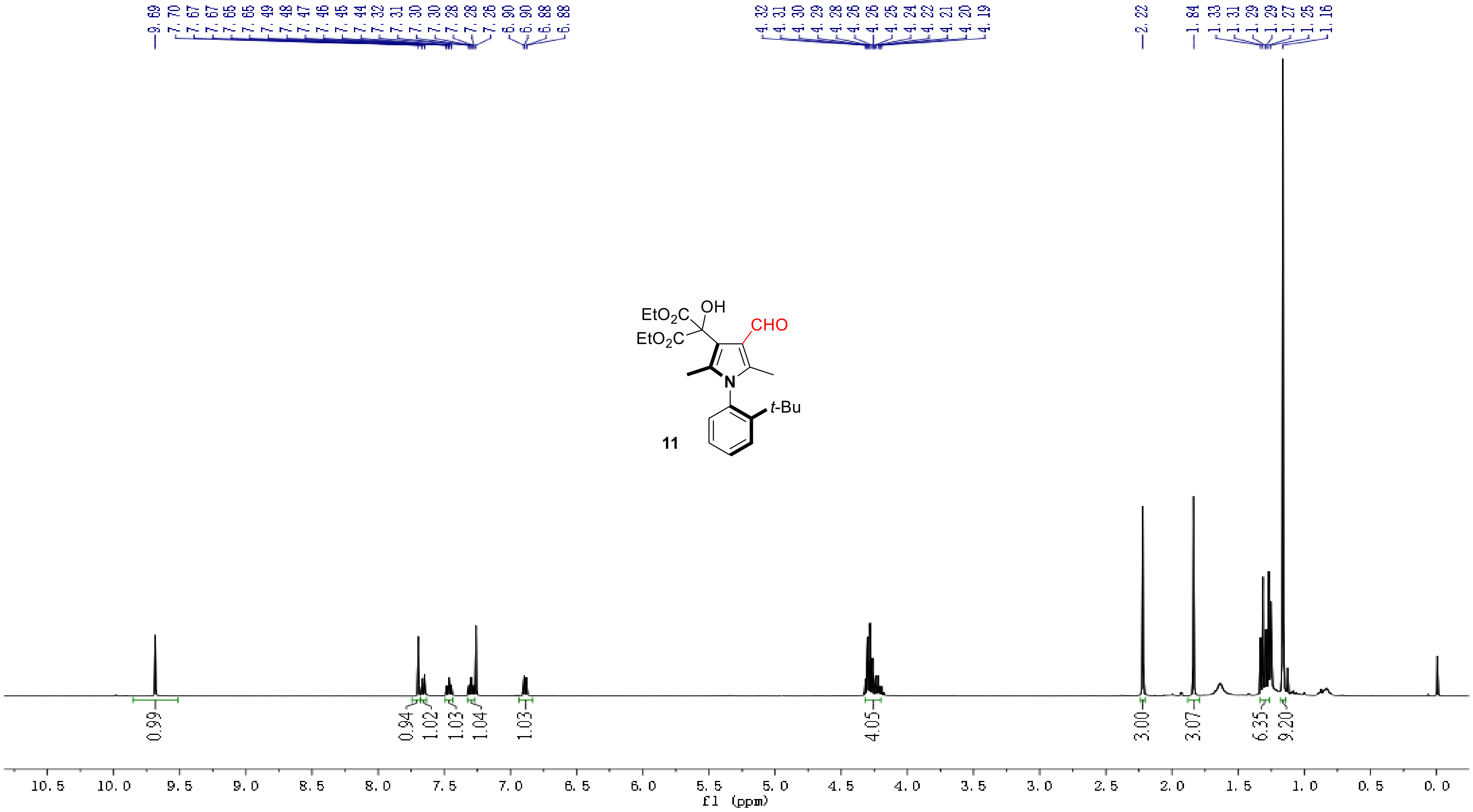

**Supplementary Figure 173.** $^1$H NMR of **11**.

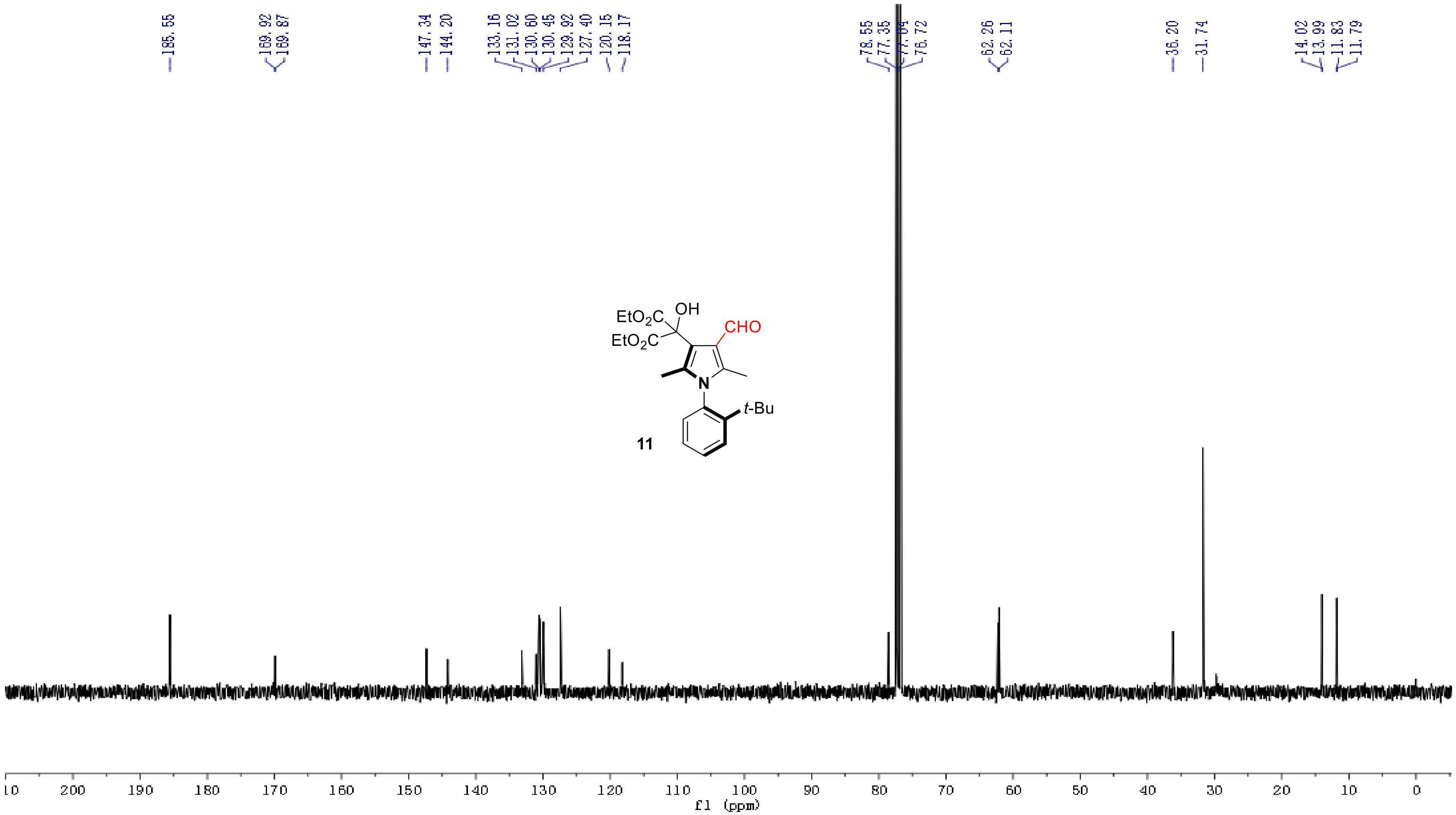


**Supplementary Figure 174.** $^{13}$C NMR of **11**.

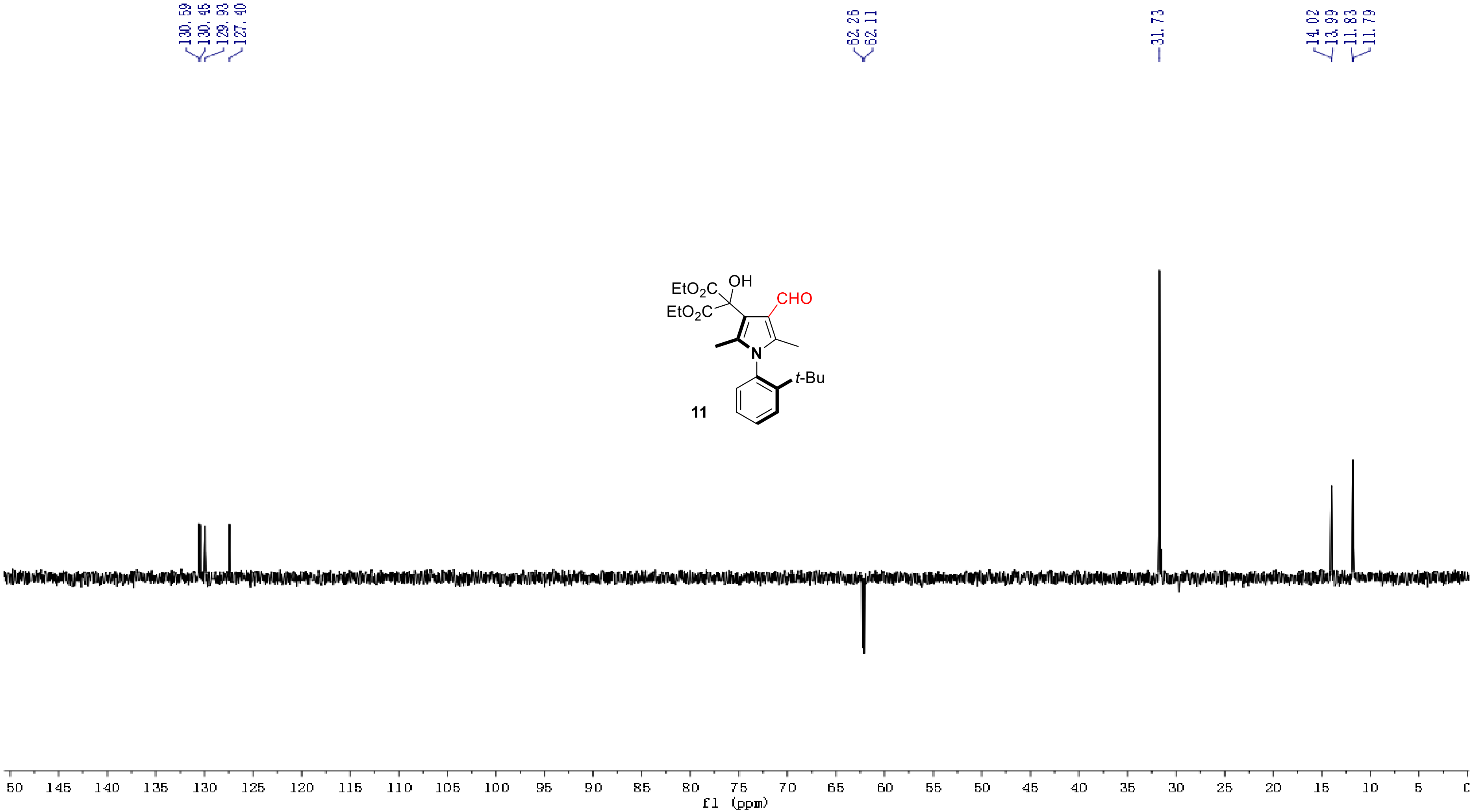


**Supplementary Figure 175.** $^{13}$C NMR-DEPT 135 of **11**.

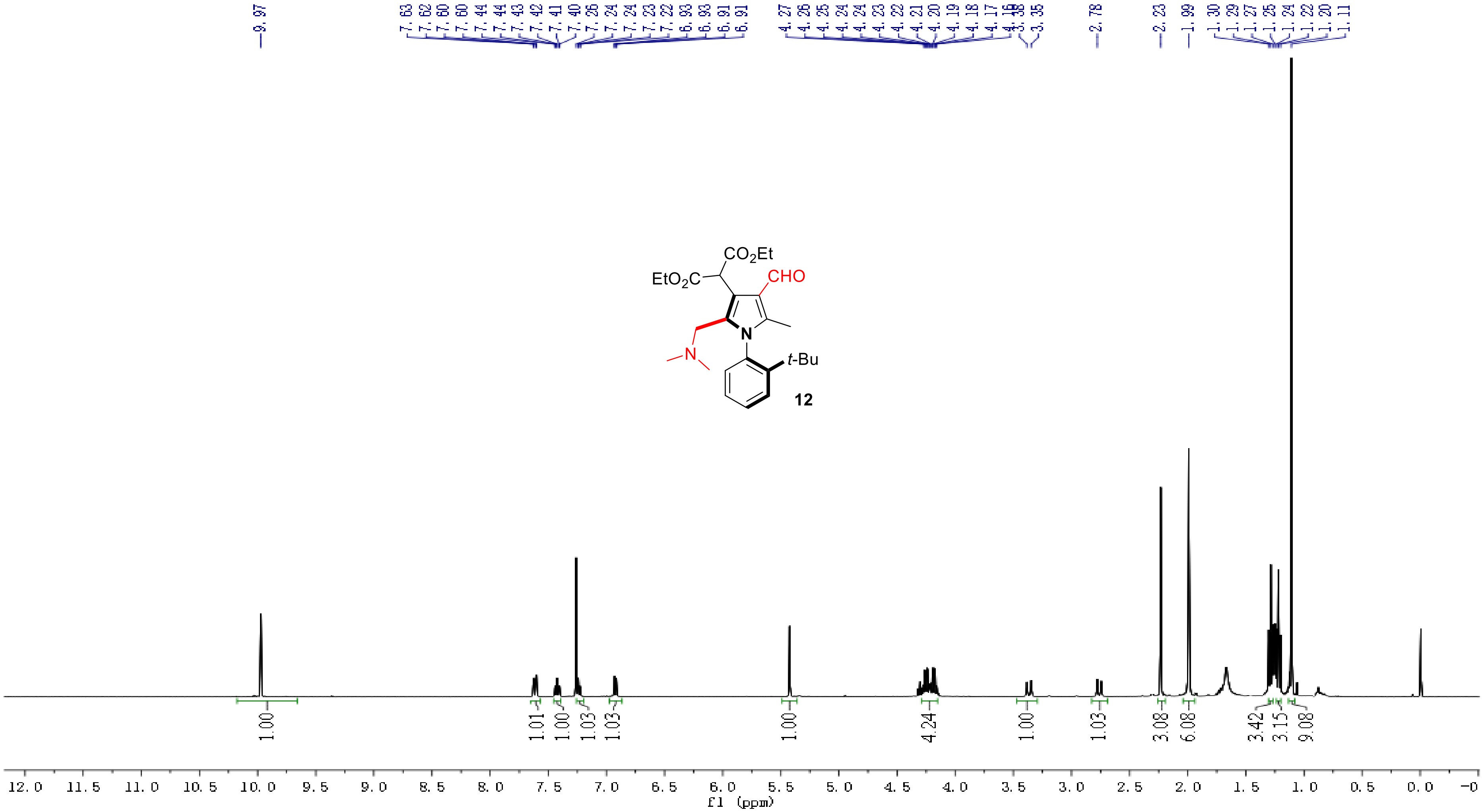


**Supplementary Figure 176.** $^{1}$H NMR of **12**.

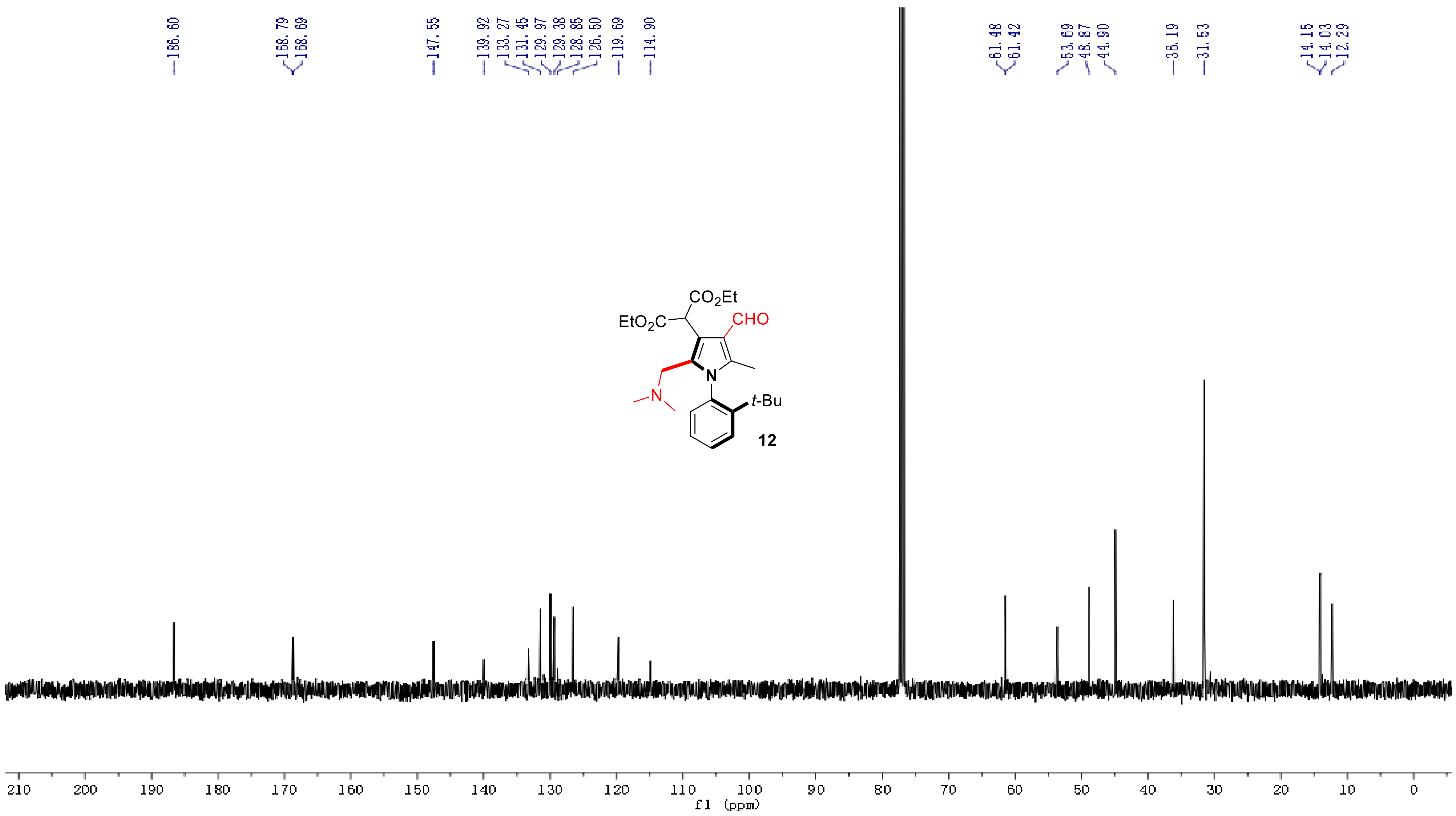


**Supplementary Figure 177.** $^{13}$C NMR of **12**.

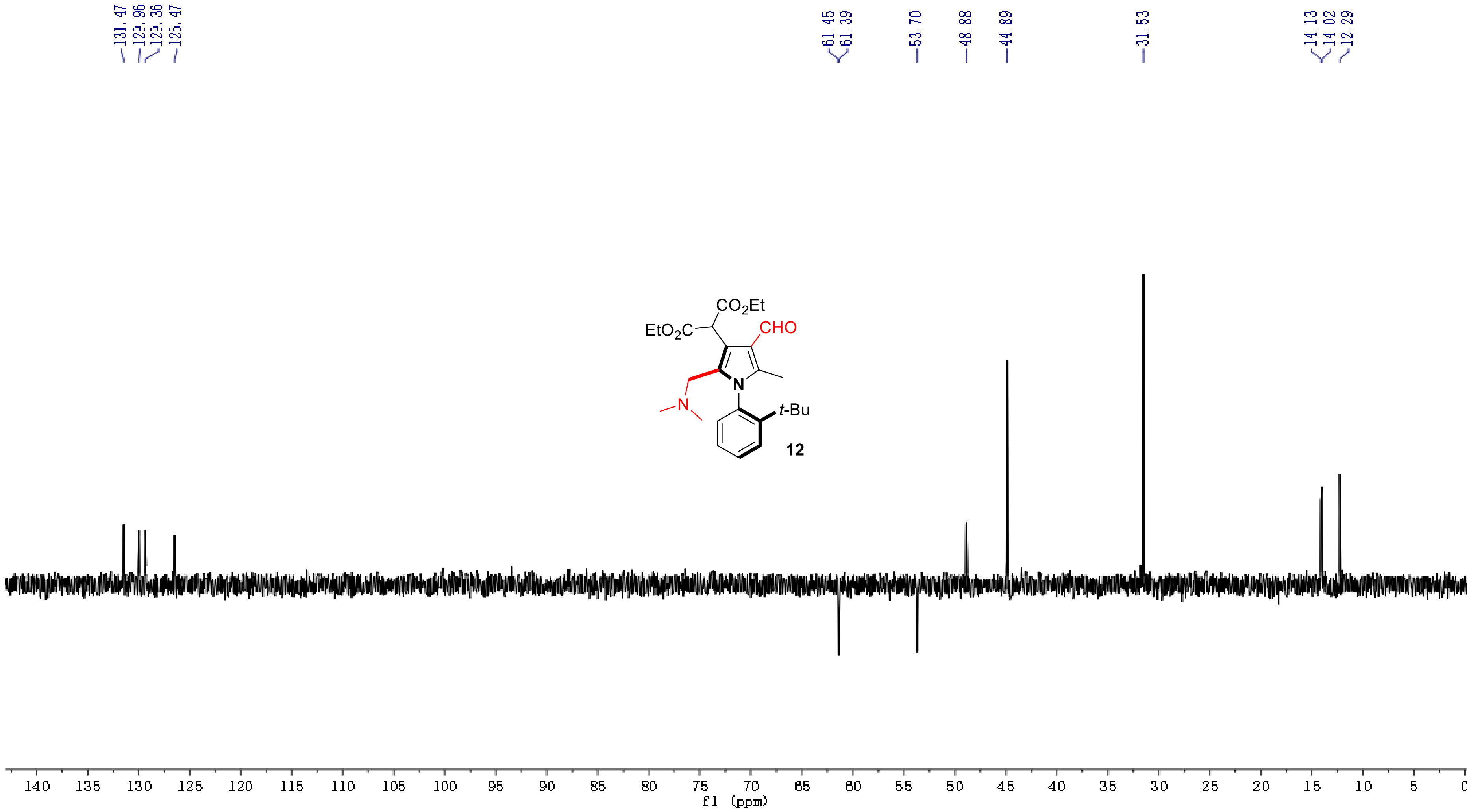


**Supplementary Figure 178.** $^{13}C$ NMR-DEPT 135 of **12**.

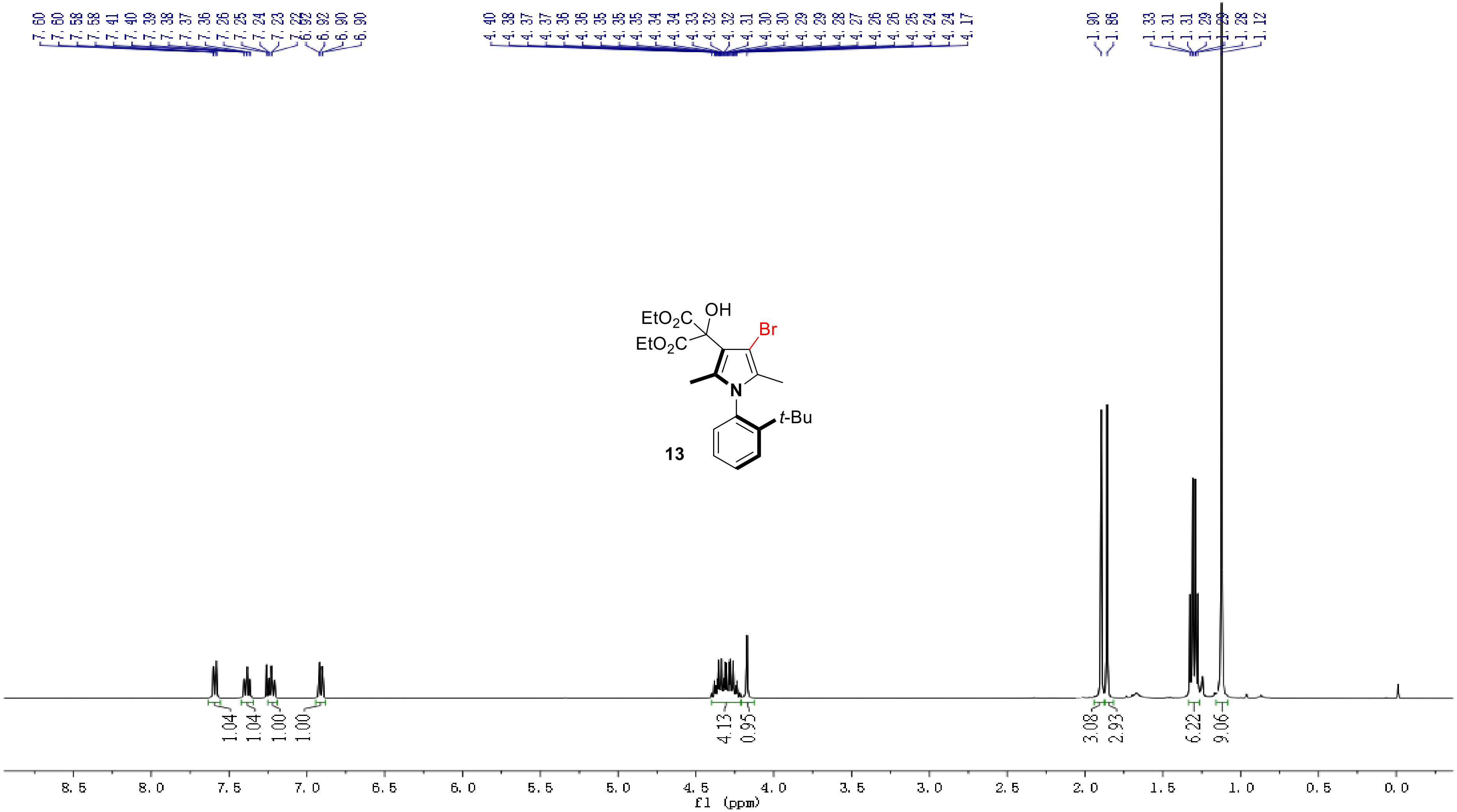

**Supplementary Figure 179.** $^{1}$H NMR of **13**.

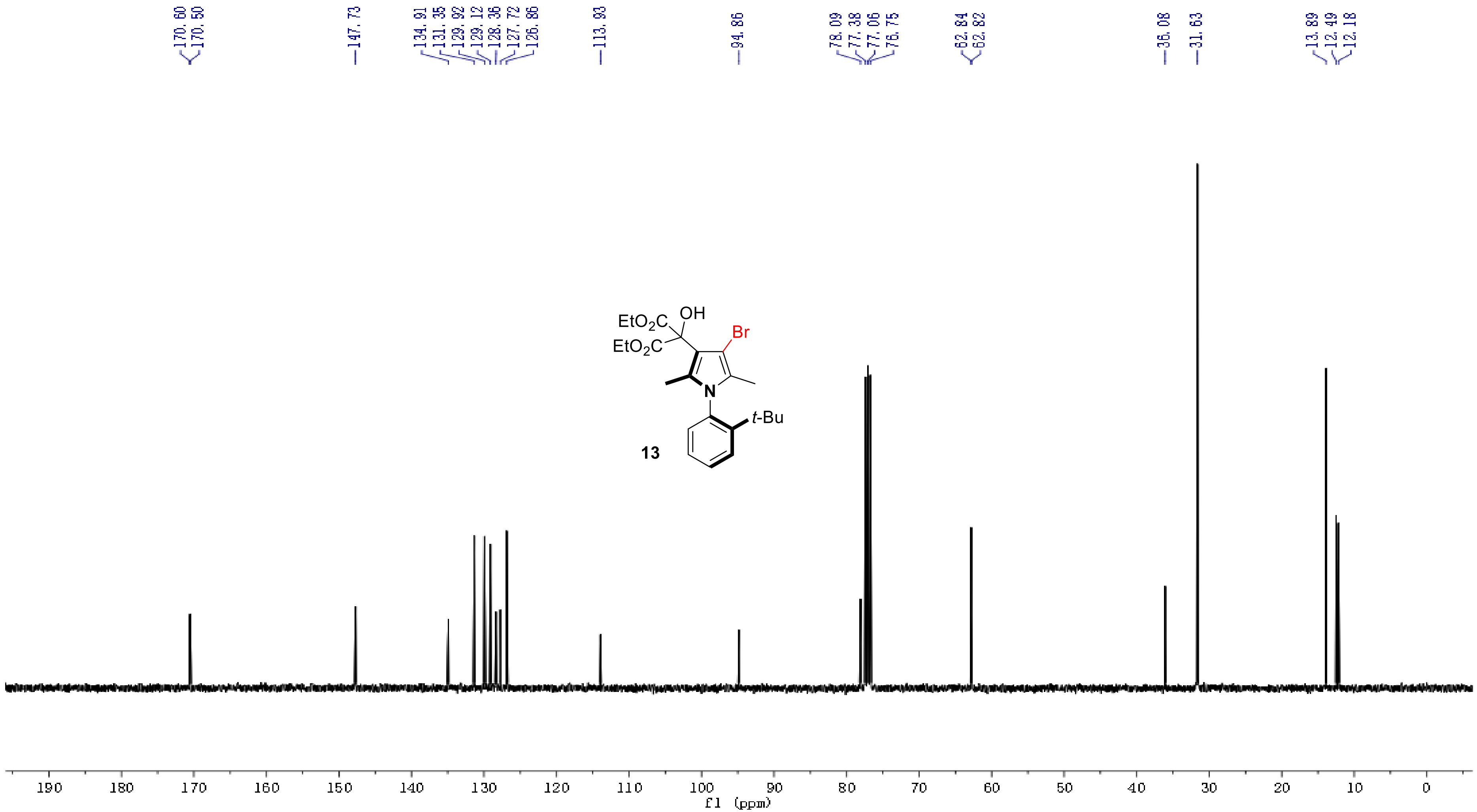


**Supplementary Figure 180.** $^{13}$C NMR of **13**.

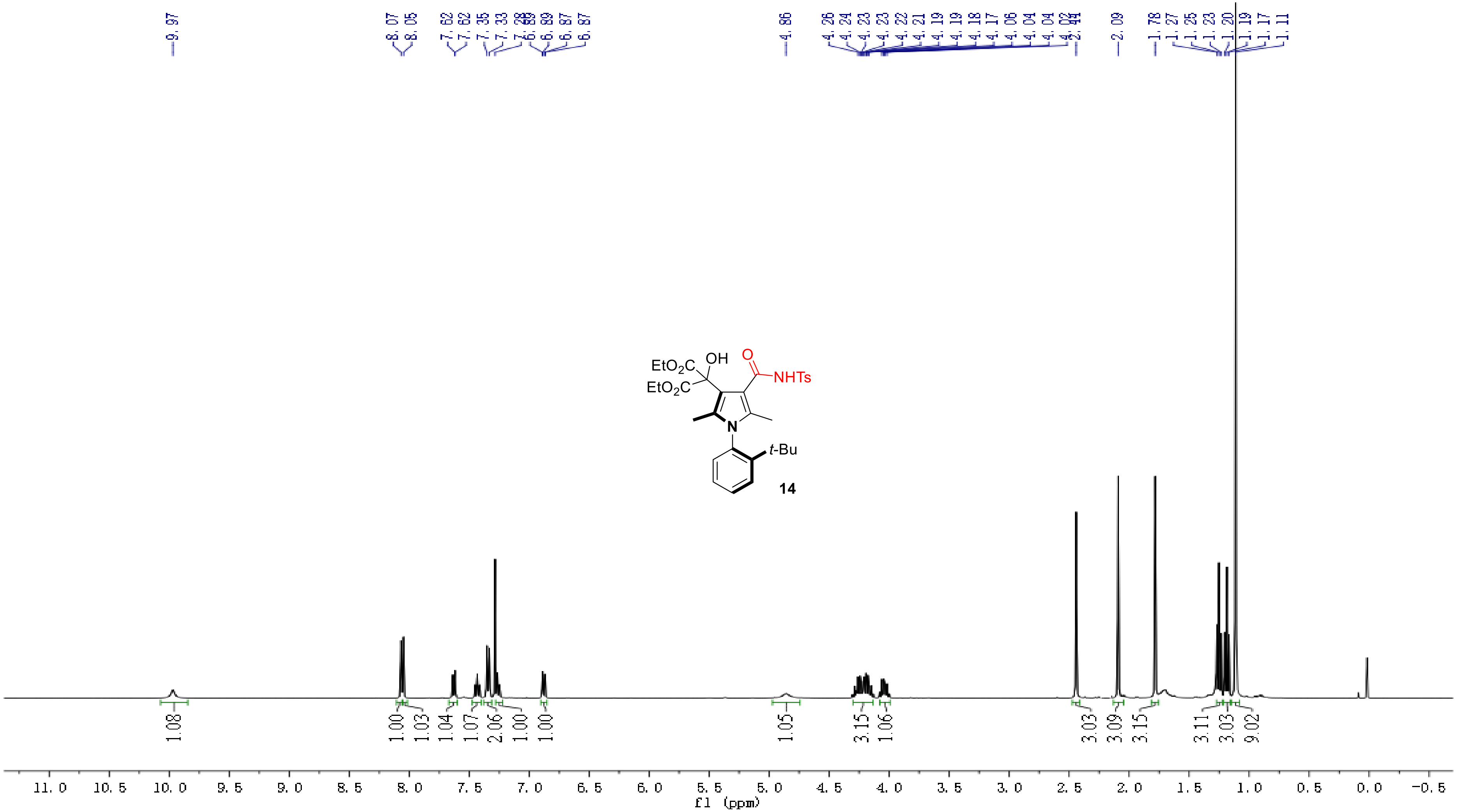

**Supplementary Figure 181.** $^{1}$H NMR of **14**.

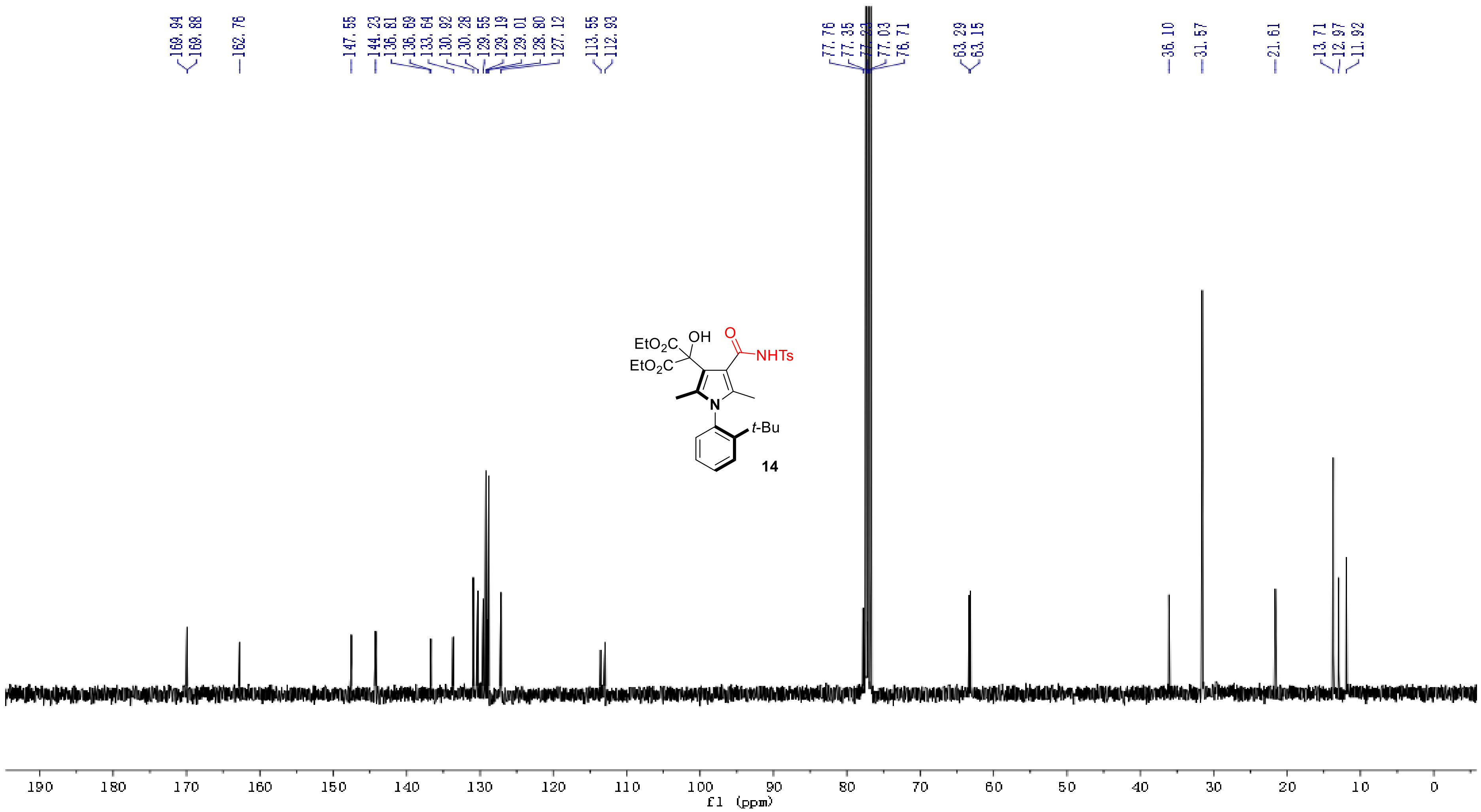

**Supplementary Figure 182.** $^{13}$C NMR of **14**.

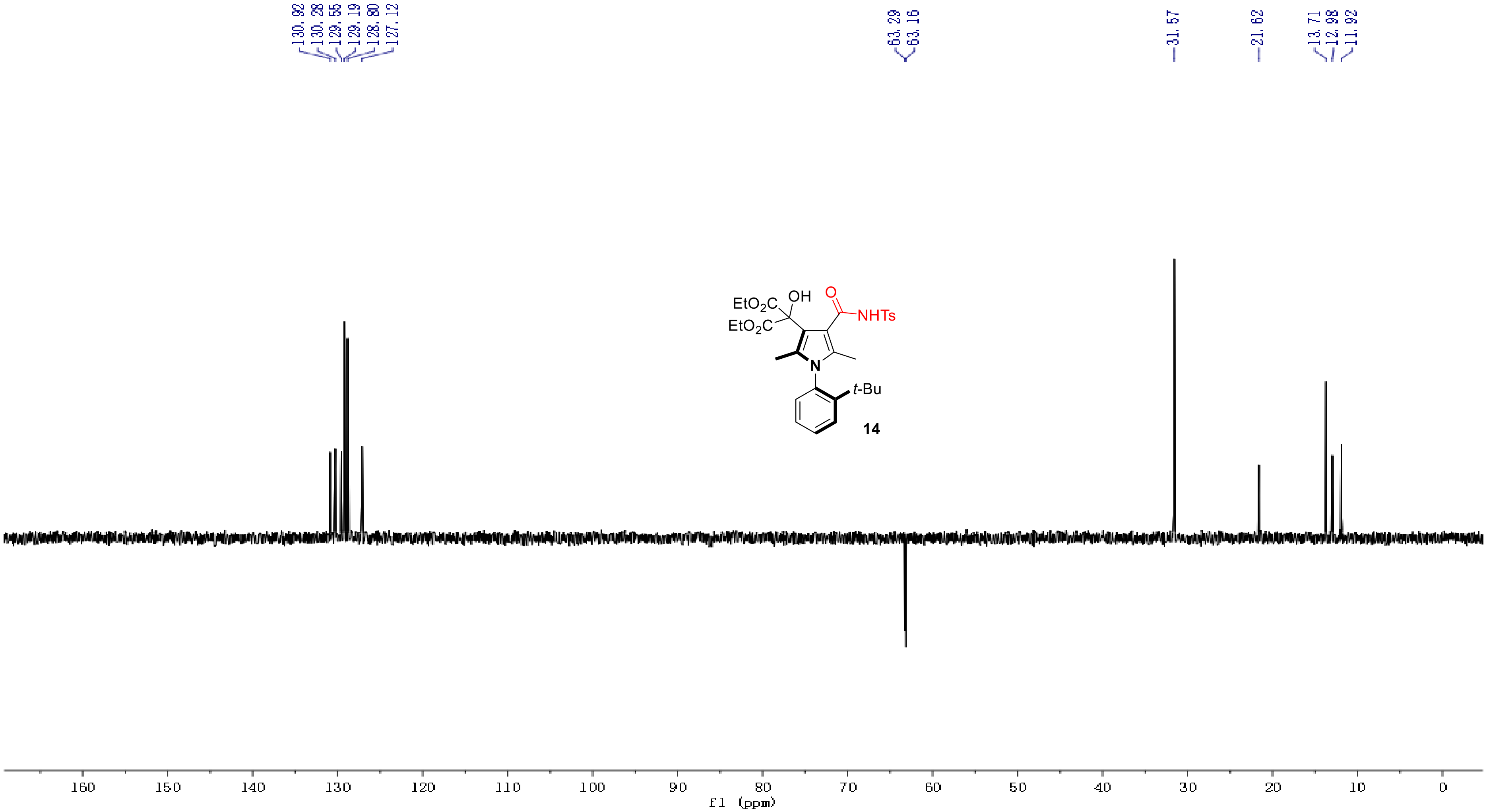


**Supplementary Figure 183.** $^{13}$C NMR-DEPT 135 of **14**.

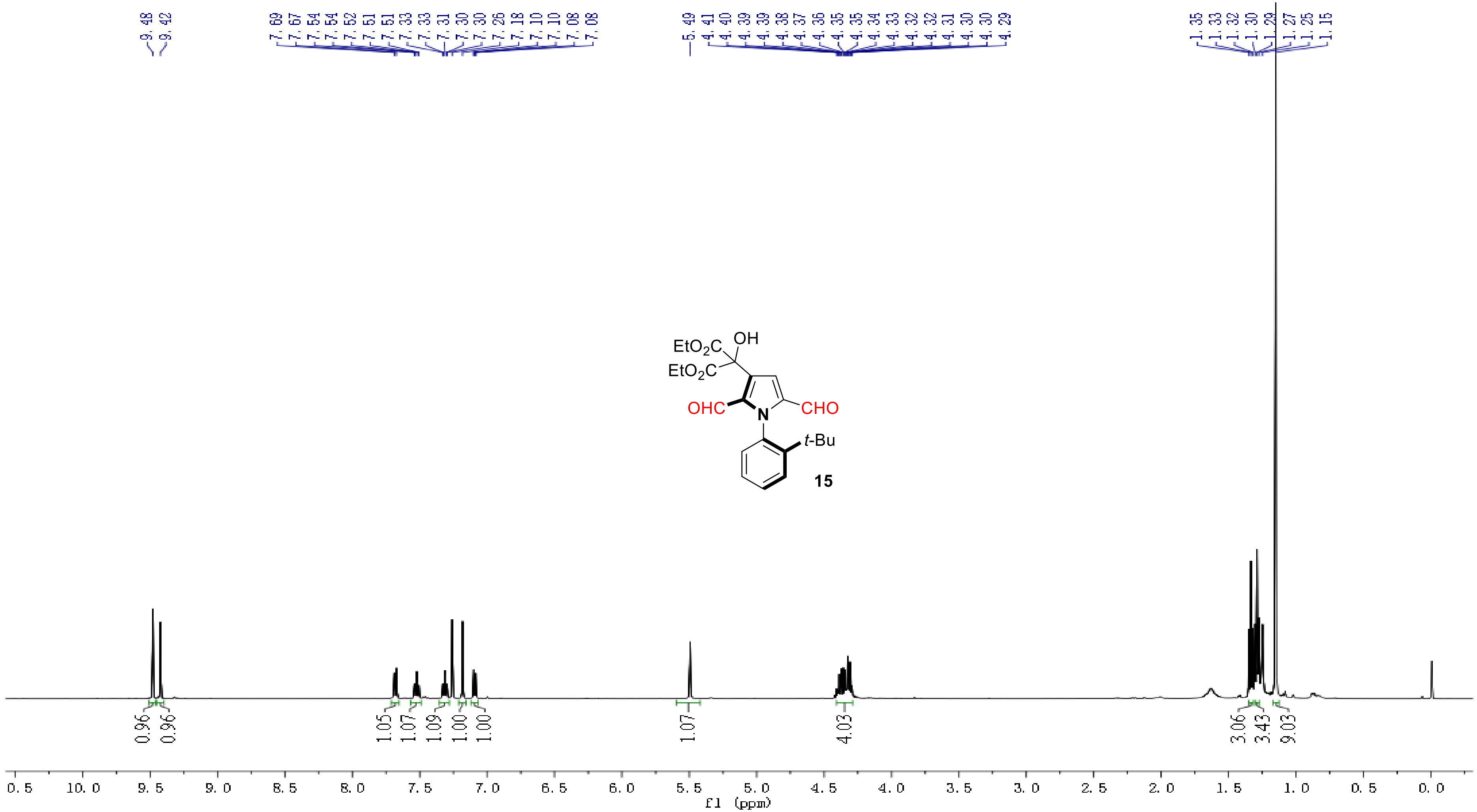


**Supplementary Figure 184.** $^{1}$H NMR of **15**.

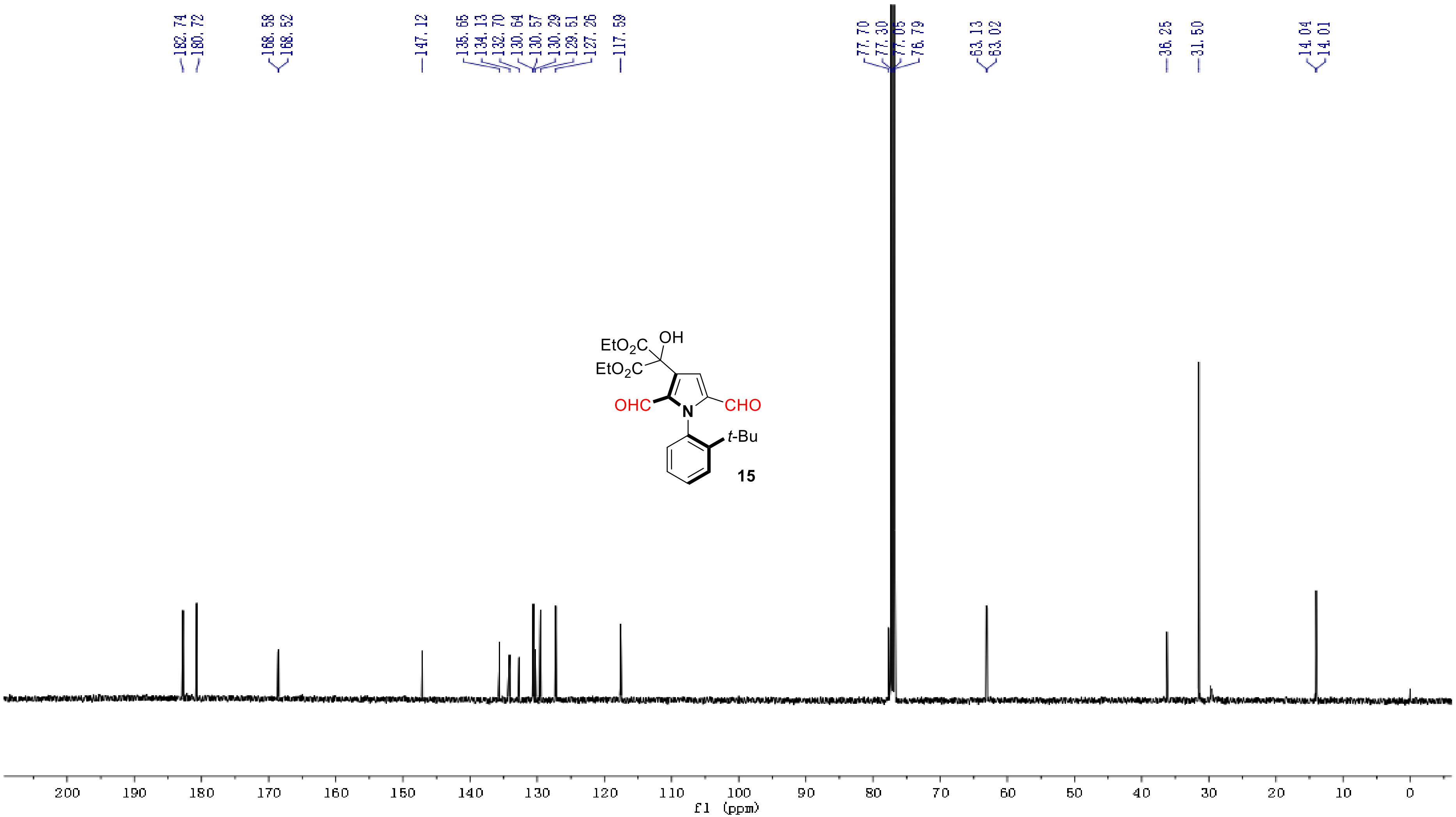


**Supplementary Figure 185.** $^{13}$C NMR of **15**.

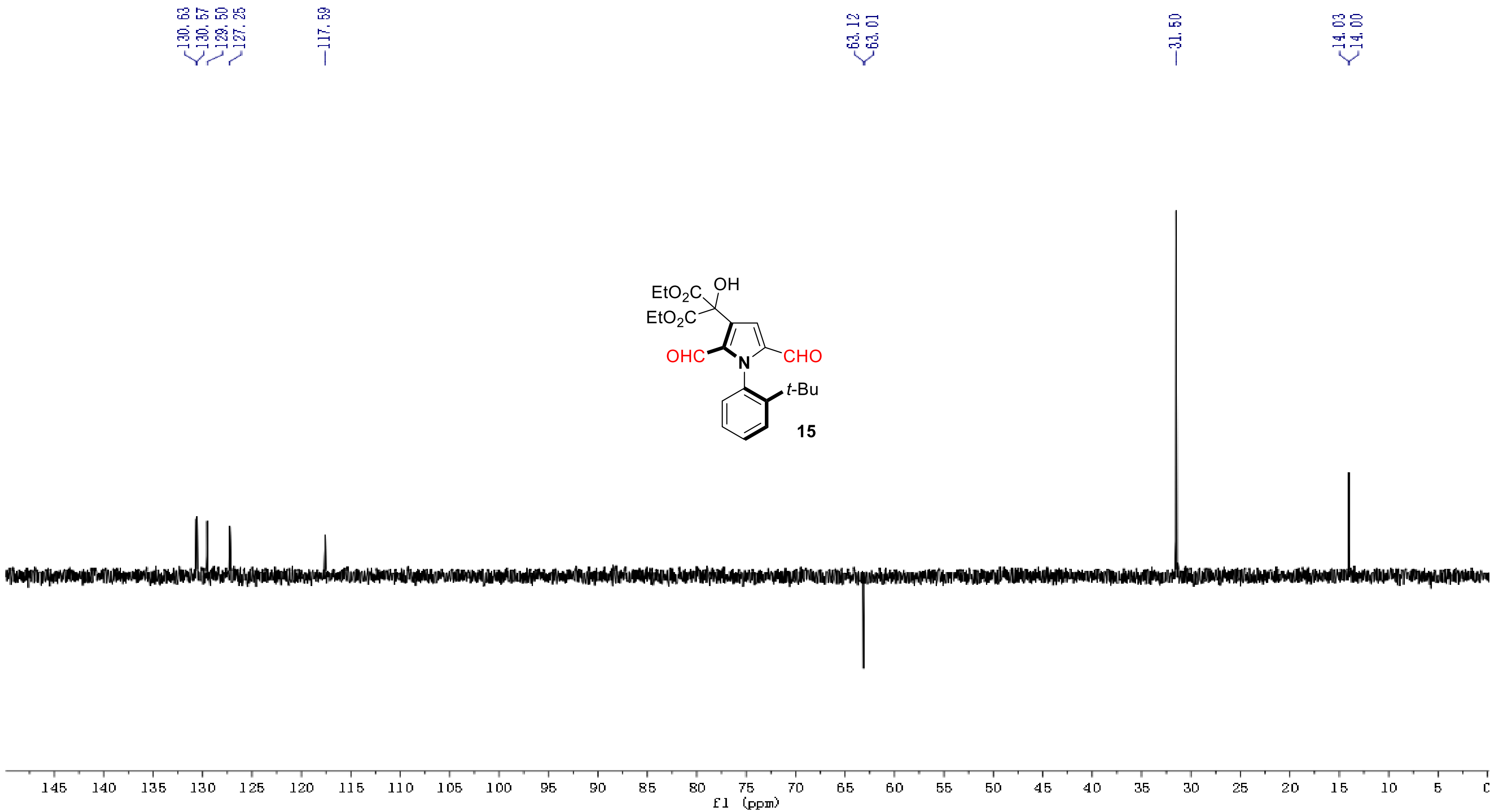


**Supplementary Figure 186.** $^{13}C$ NMR-DEPT 135 of **15**.

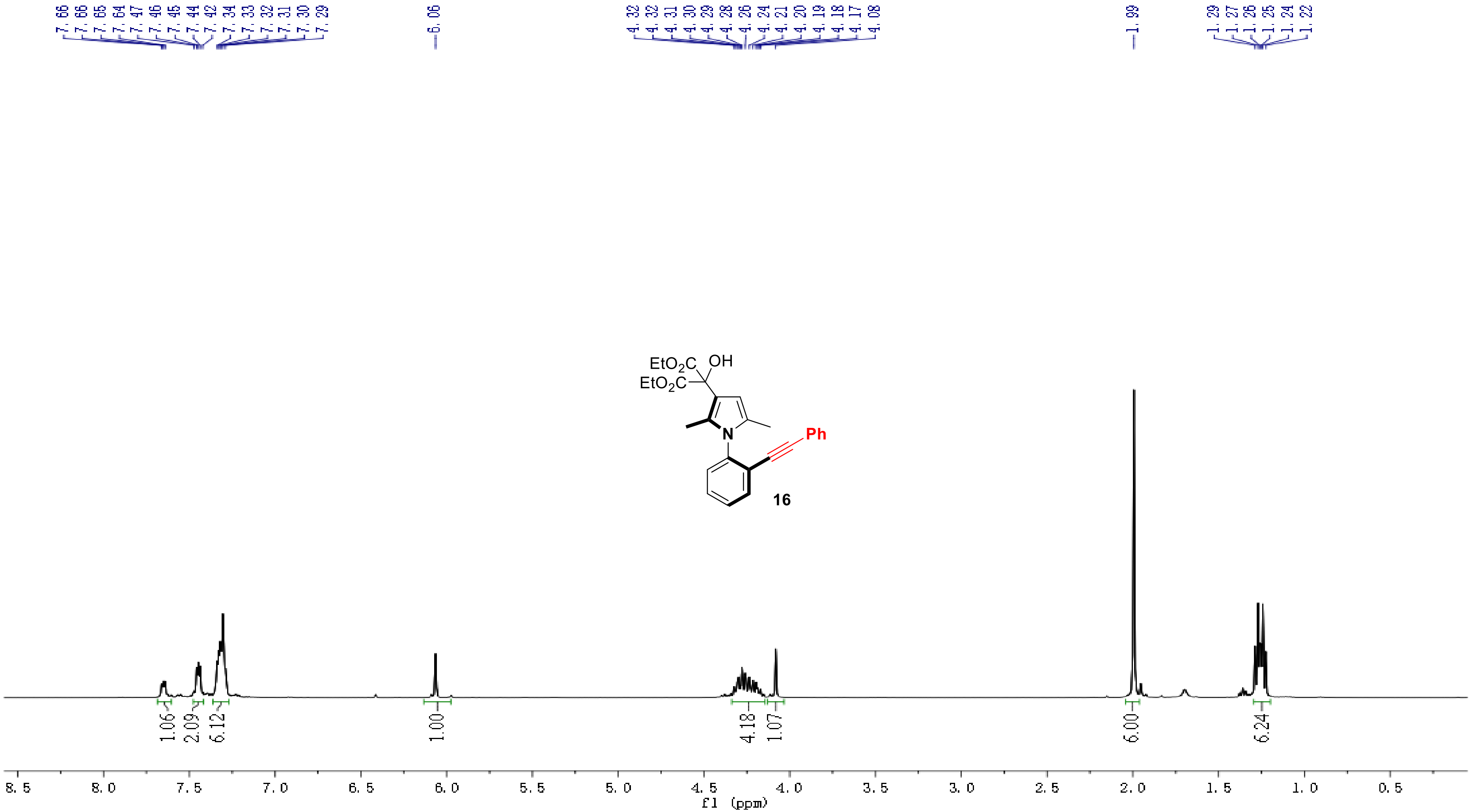


**Supplementary Figure 187.** $^{1}$H NMR of **16**.

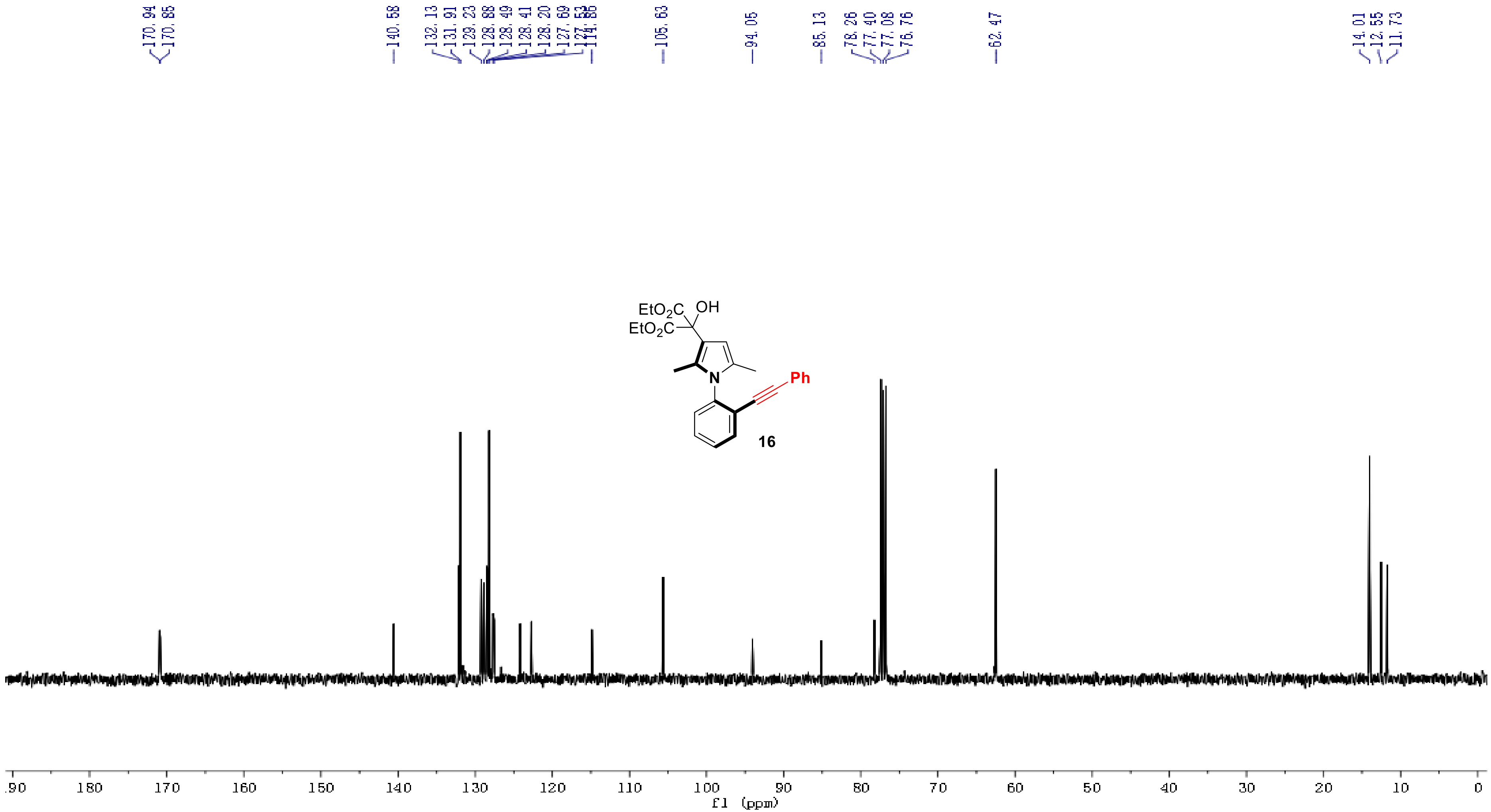


**Supplementary Figure 188.** $^{13}$C NMR of **16**.

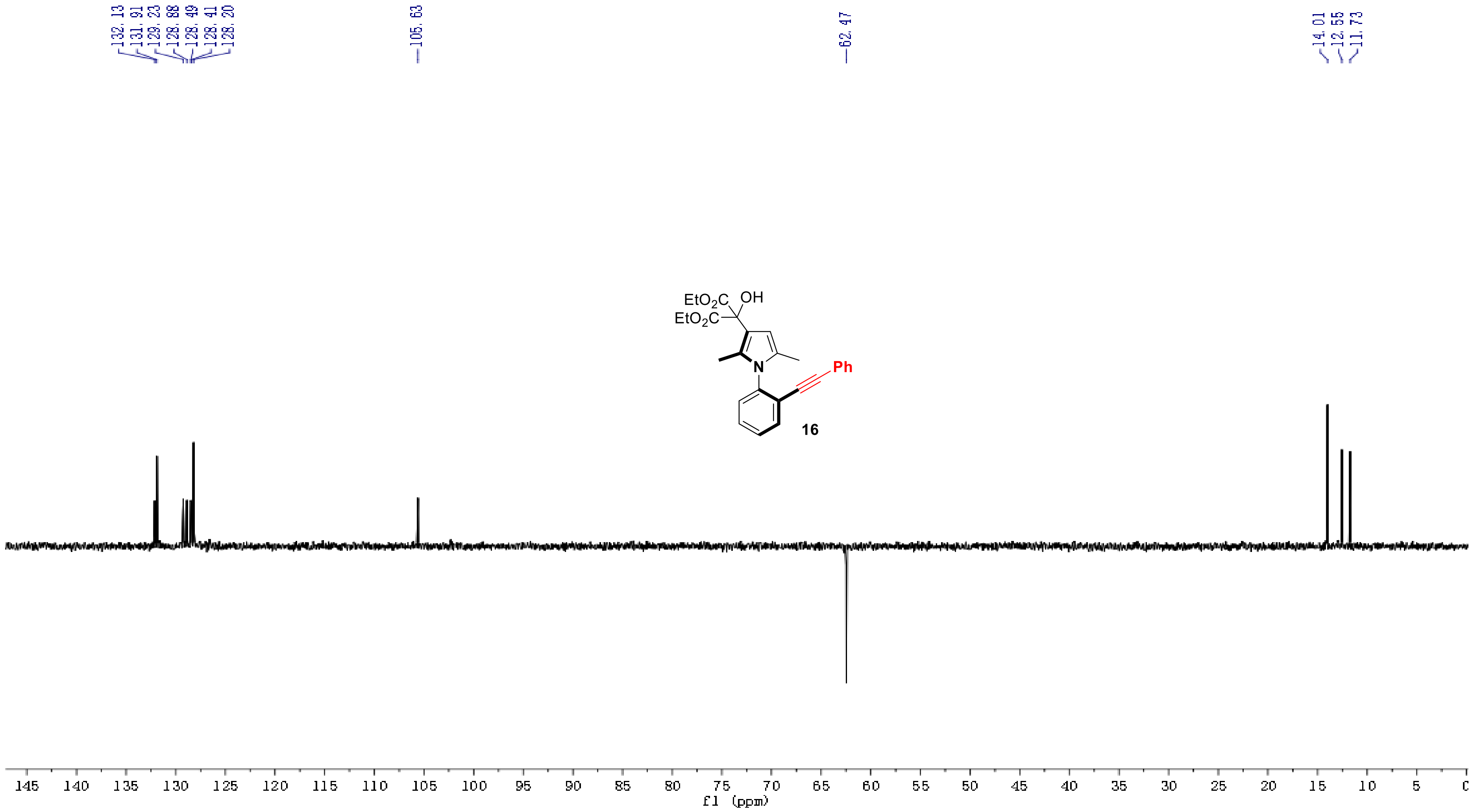


**Supplementary Figure 189.** $^{13}$C NMR-DEPT 135 of **16**.

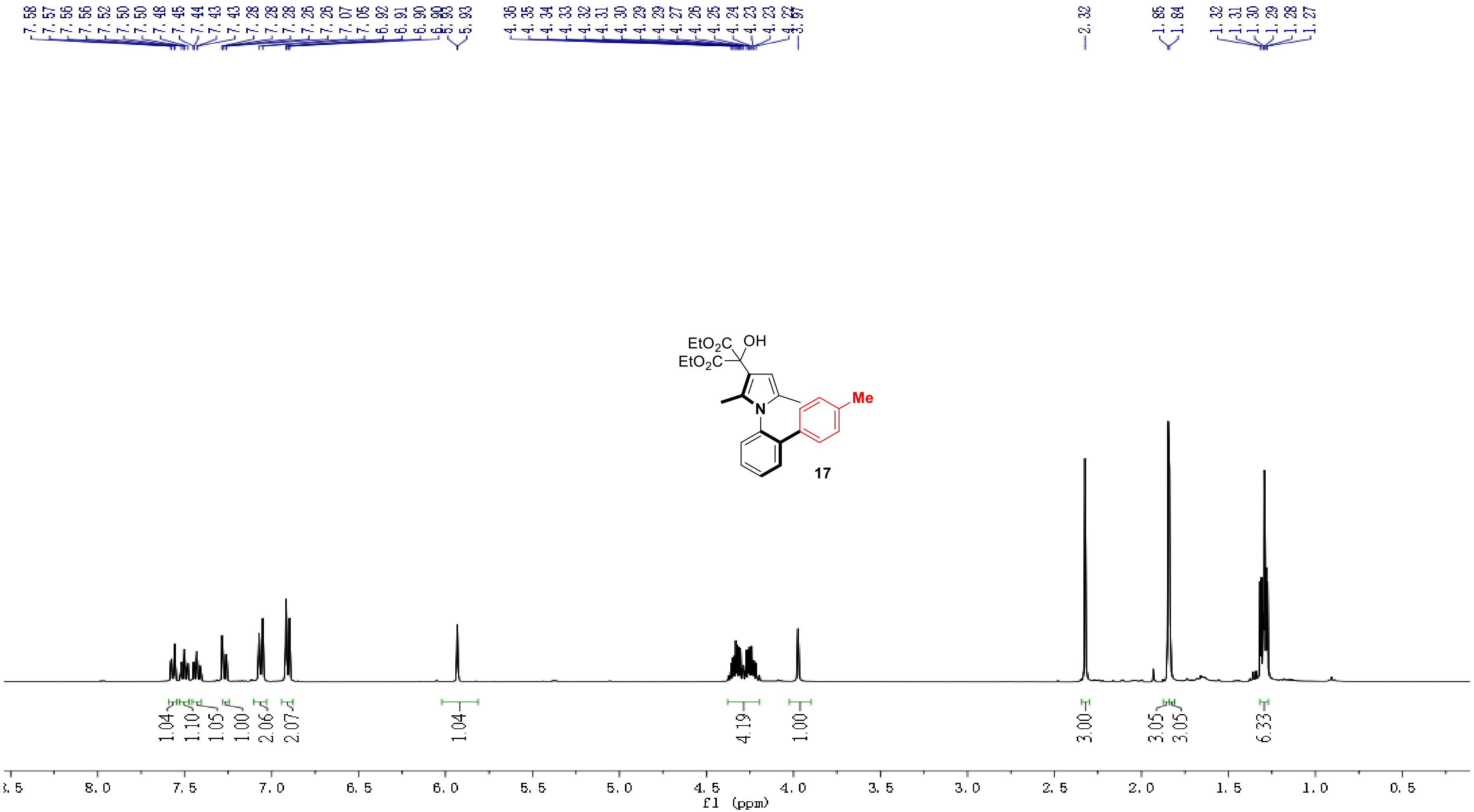

**Supplementary Figure 190.** $^{1}$H NMR of **17**.

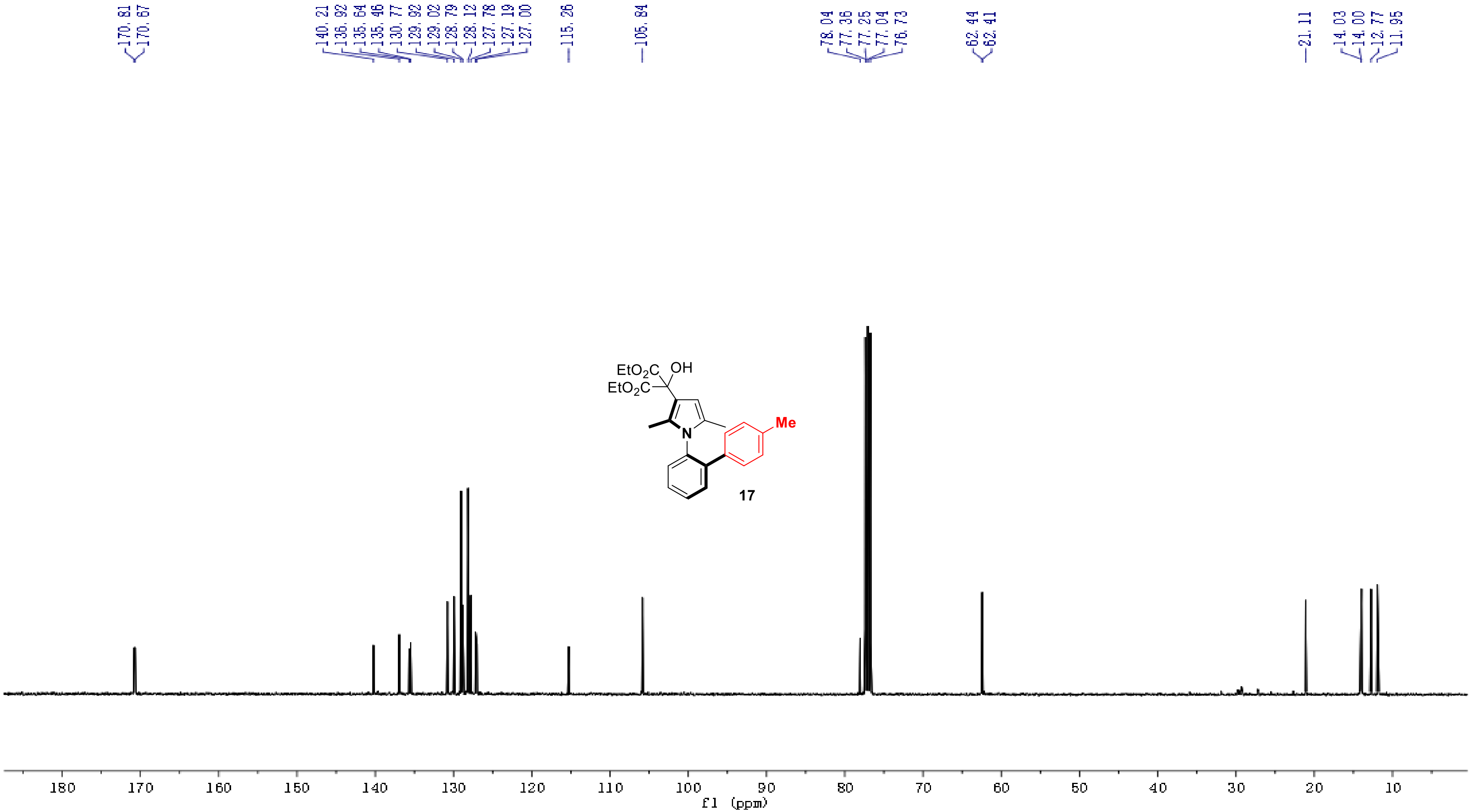


**Supplementary Figure 191.** $^{13}$C NMR of **17**.

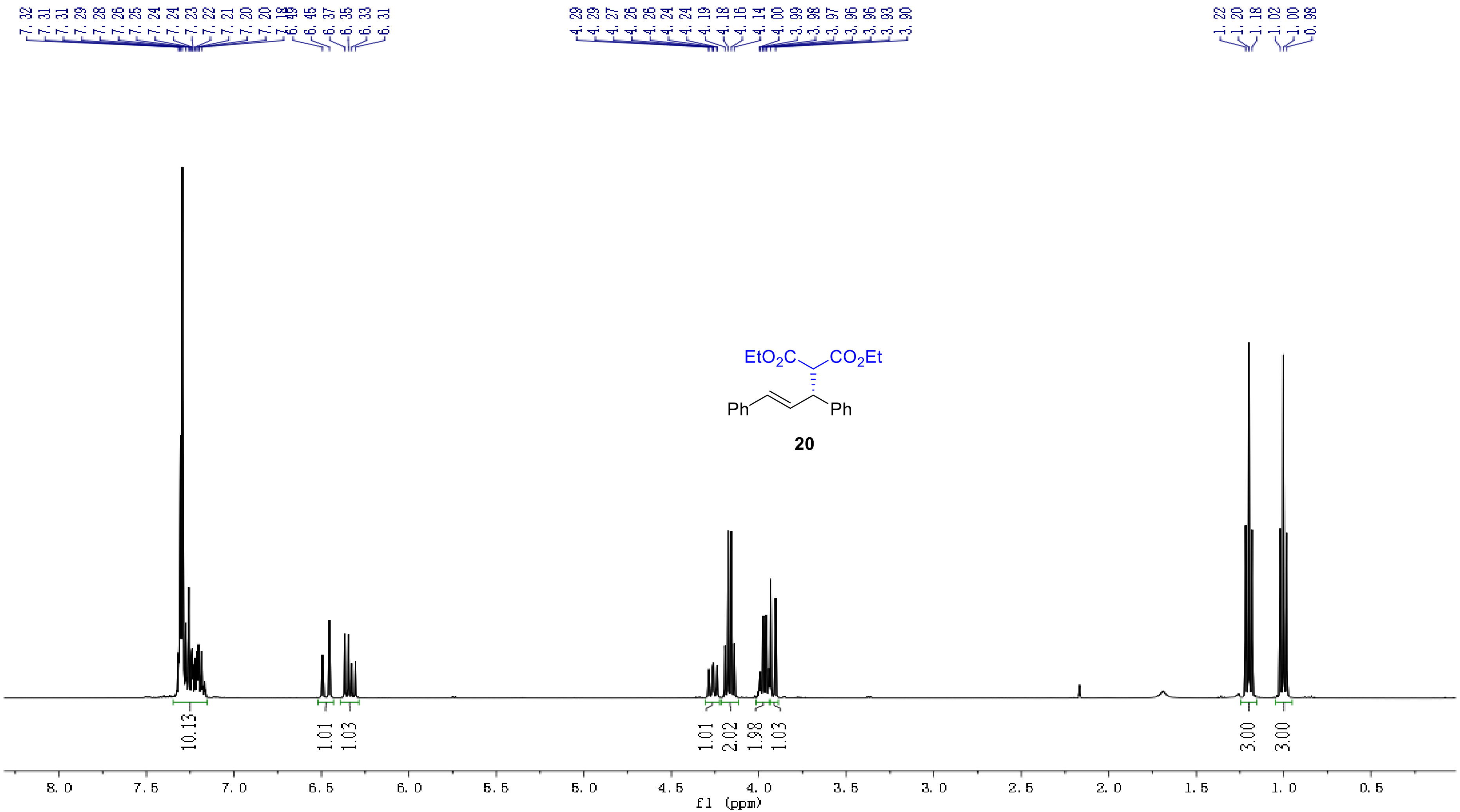

**Supplementary Figure 192.** $^{1}$H NMR of **20**.

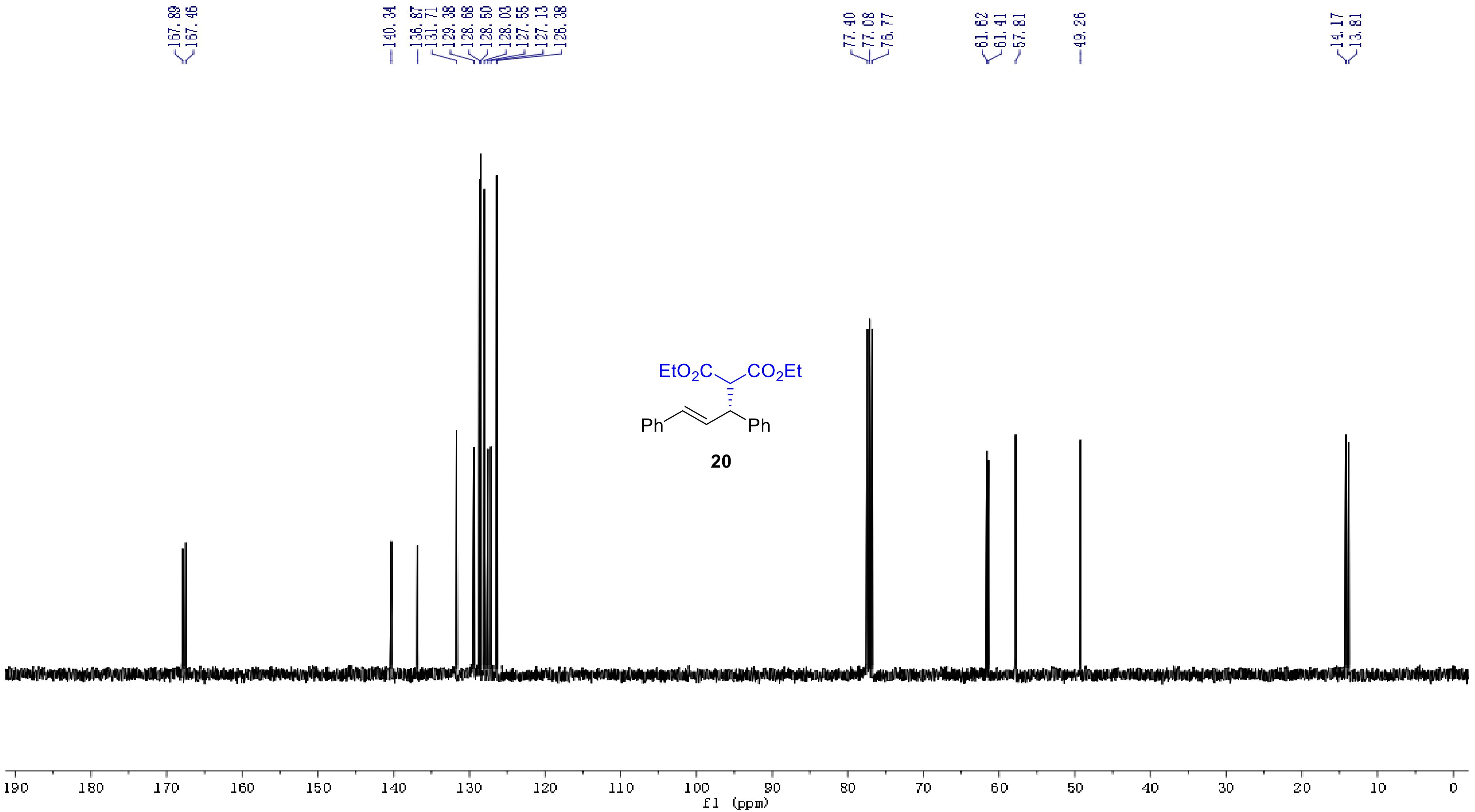


**Supplementary Figure 193.** $^{13}$C NMR of **20**.

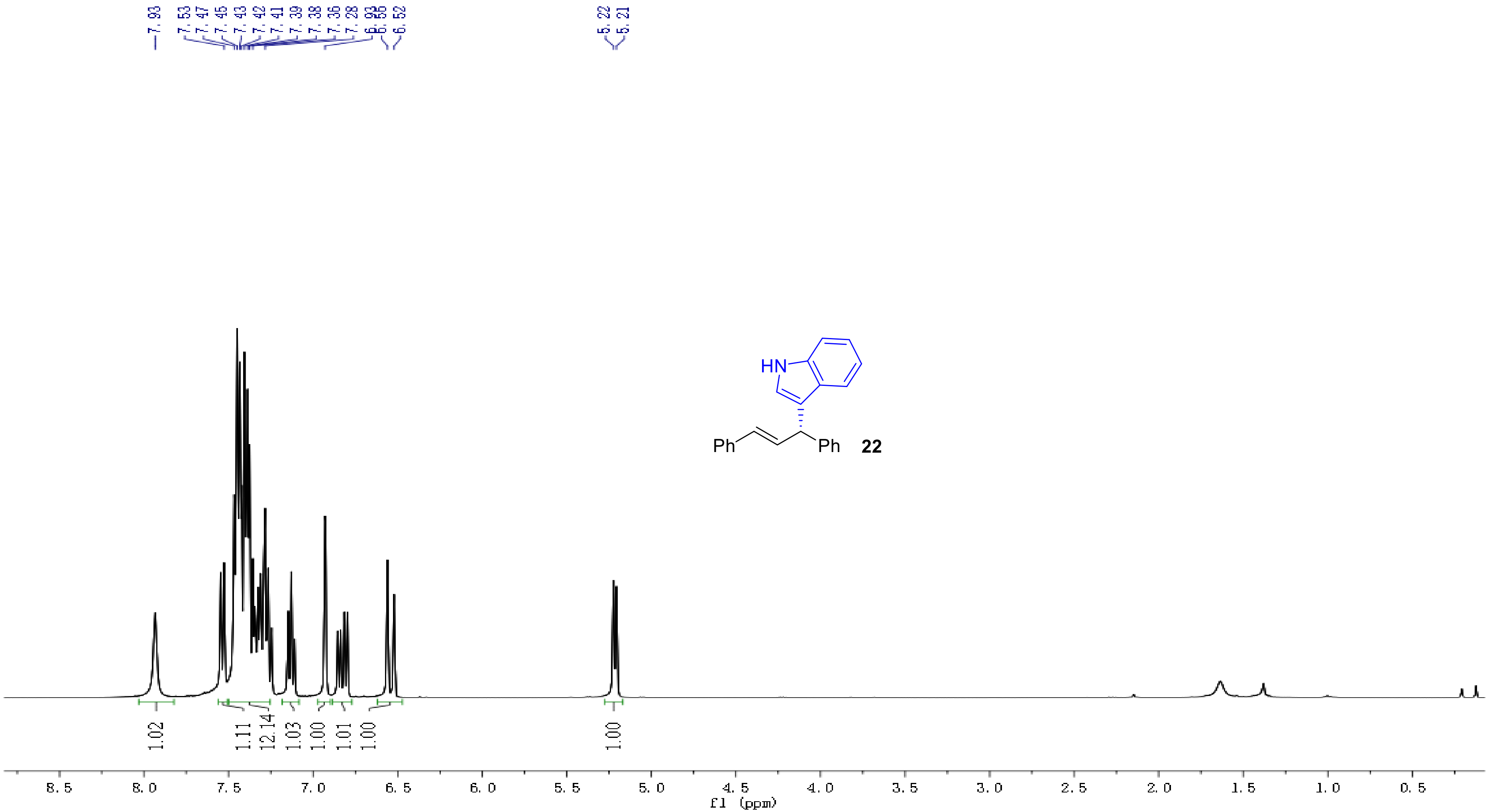


**Supplementary Figure 194.** $^{1}$H NMR of **22**.

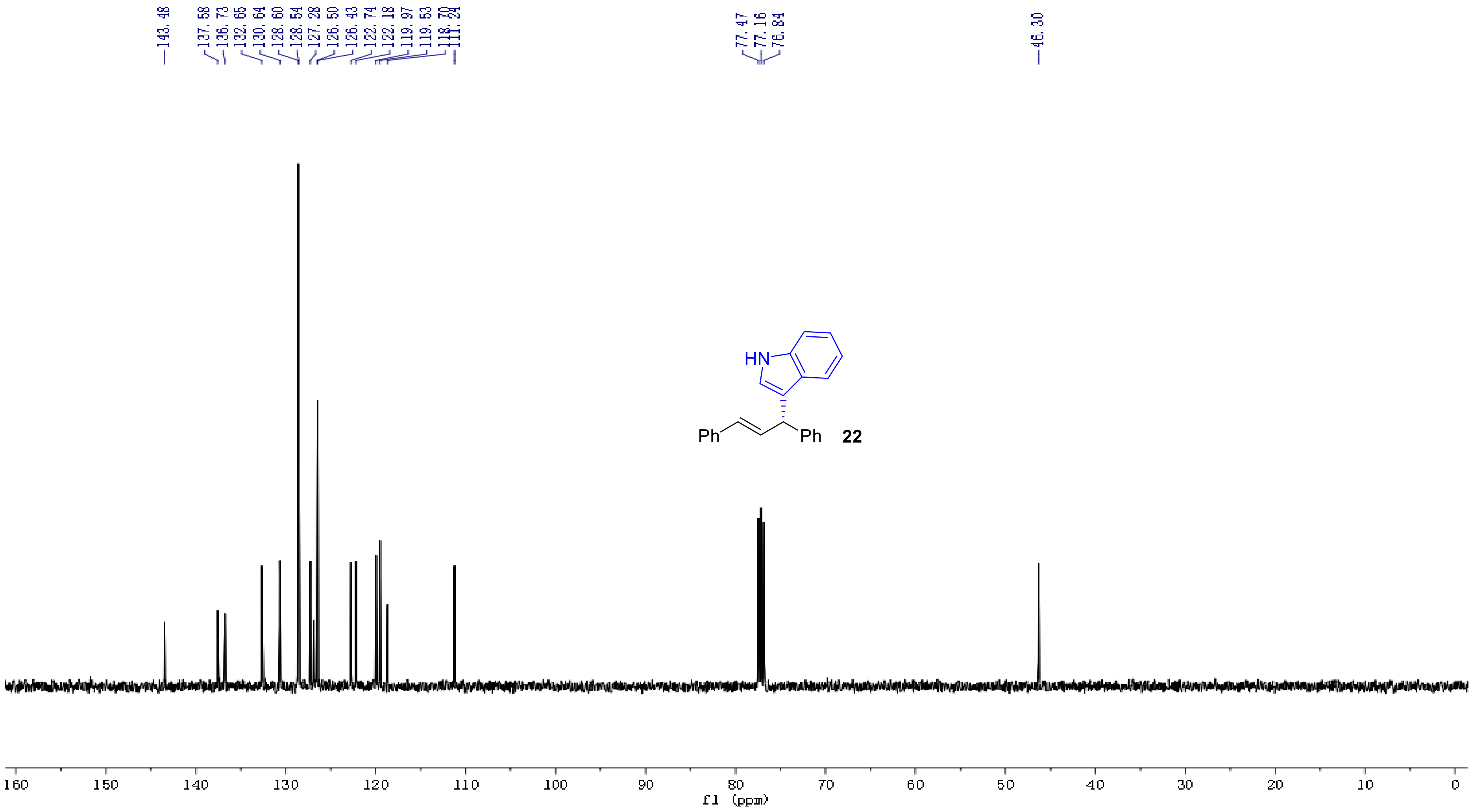


**Supplementary Figure 195.** $^{13}$C NMR of **22**.

# Supplementary Tables

## Supplementary Table 1. Chiral phosphoric acids screening[a]

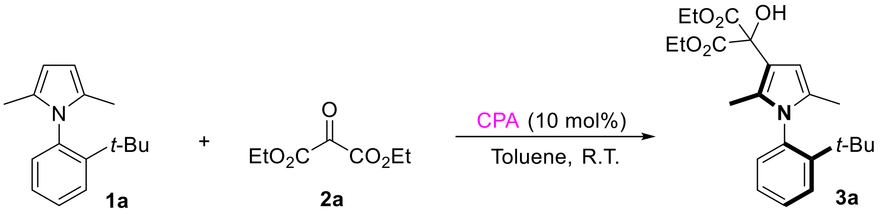


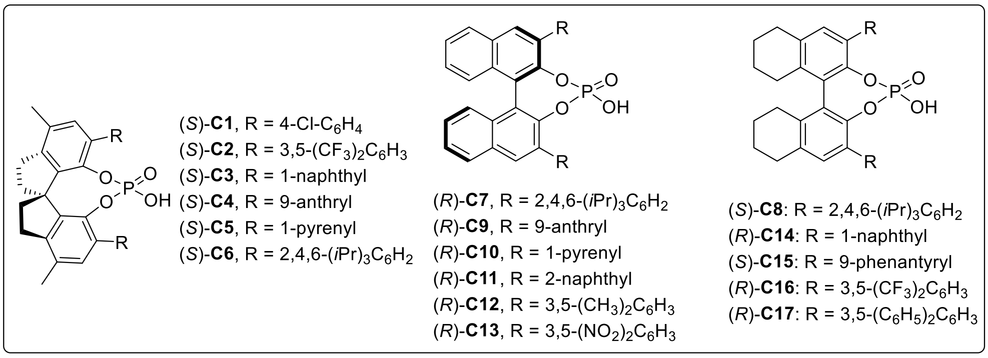


| entry | catalyst | T (h) | yield (%)[b] | ee (%)[c] |
|---|---|---|---|---|
| 1 | **(*S*)-C1** | 24 | 74 | -22 |
| 2 | **(*S*)-C2** | 24 | 85 | -42 |
| 3 | **(*S*)-C3** | 24 | 38 | -47 |
| 4 | **(*S*)-C4** | 24 | 43 | -71 |
| 5 | **(*S*)-C5** | 24 | 59 | -72 |
| 6 | **(*S*)-C6** | 24 | 75 | -86 |
| 7 | **(*R*)-C7** | 24 | 91 | -89 |
| 8 | **(*S*)-C8** | 24 | 93 | 90 |
| 9 | **(*R*)-C9** | 24 | 66 | -61 |
| 10 | **(*R*)-C10** | 24 | 88 | -58 |
| 11 | **(*R*)-C11** | 32 | 80 | -19 |
| 12 | **(*R*)-C12** | 32 | 69 | -32 |
| 13 | **(*R*)-C13** | 32 | 70 | -44 |
| 14 | **(*R*)-C14** | 36 | 24 | -29 |
| 15 | **(*S*)-C15** | 24 | 71 | 65 |
| 16 | **(*R*)-C16** | 22 | 70 | -34 |
| 17 | **(*R*)-C17** | 22 | 72 | -42 |

[a]Reaction was carried out with **1a** (0.15 mmol), **2a** (0.10 mmol), and **CPA** (10 mol%) in 1.5 mL toluene, unless noted otherwise; [b]Isolated yield; [c]Determined by HPLC analysis using a chiral stationary phase.

## Supplementary Table 2. Solvent effect screening screening[a]

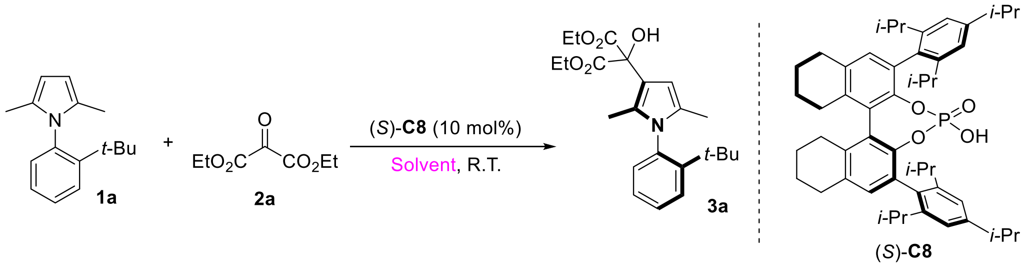


| entry | solvent | T (h) | yield (%)[b] | ee (%)[c] |
|---|---|---|---|---|
| 1 | Toluene | 32 | 96 | 90 |
| 2 | $CHCl_3$ | 38 | 89 | 78 |
| 3 | $CH_2Cl_2$ | 24 | 91 | 77 |
| 4 | THF | 42 | 22 | 90 |
| 5 | $CCl_4$ | 38 | 86 | 93 |
| 6 | DCE | 38 | 91 | 80 |
| 7 | $Et_2O$ | 40 | 45 | 92 |
| 8 | $CH_3CN$ | 24 | 92 | 62 |
| 9 | EtOAc | 24 | 60 | 86 |
| 10 | Benzotrifluoride | 38 | 88 | 82 |
| 11 | Cyclohexane | 24 | 92 | 96 |
| 12 | Methylcyclohexane | 30 | 83 | 93 |

[a]Reaction was carried out with **1a** (0.15 mmol), **2a** (0.10 mmol), and (*S*)-**C8** (10 mol%) in 1.5 mL of solvent; [b]Isolated yield; [c]Determined by HPLC analysis using a chiral stationary phase.

## Supplementary Table 3. The effects of the temperature and the molar ratio of reactants[a]

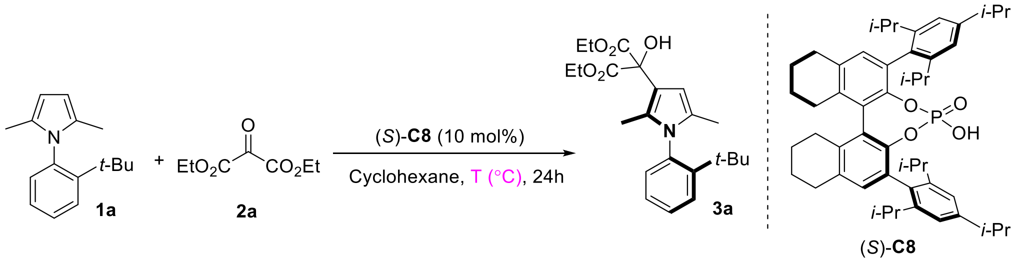


| entry | molar ratio of **1a** and **2a** | T (°C) | yield (%)[b] | ee (%)[c] |
|---|---|---|---|---|
| 1 | 1.0 : 1.0 | R.T. | 84 | 94 |
| 2 | 1.2 : 1.0 | R.T. | 88 | 96 |
| 3 | 1.5 : 1.0 | R.T. | 92 | 96 |
| 4 | 2.0 : 1.0 | R.T. | 97 | 93 |
| 5 | 1.0 : 1.2 | R.T. | 80 | 95 |
| 6 | 1.0 : 1.5 | R.T. | 83 | 94 |
| 7 | 1.0 : 2.0 | R.T. | 72 | 93 |
| 8 | 1.5 : 1.0 | 20 | 87 | 96 |
| 9 | 1.5 : 1.0 | 15 | 68 | 96 |
| 10 | 1.5 : 1.0 | 10 | 45 | 97 |

[a]Reaction was carried out with **1a** (0.10-0.20 mmol), **2a** (0.10-0.20 mmol), and (*S*)-**C8** (10 mol%) in 1.5 mL cyclohexane. [b]Isolated yield; [c]Determined by HPLC analysis using a chiral stationary phase.

## Supplementary Table 4. The influence of reactant concentration and catalyst loading[a]

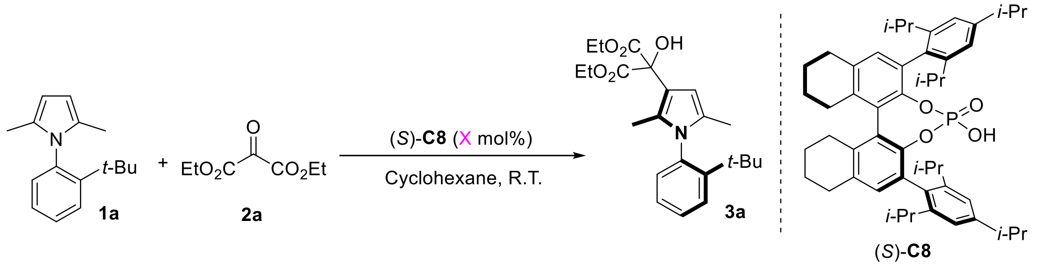


| entry | X (mol%) | solvent (mL) | yield (%)[b] | ee (%)[c] |
|---|---|---|---|---|
| 1 | 10 | 1.5 | 92 | 96 |
| 2 | 10 | 1.0 | 97 | 95 |
| 3 | 10 | 2.0 | 86 | 96 |
| 4 | 5 | 1.5 | 83 | 95 |
| 5[d] | 5 | 1.5 | 96 | 95 |

[a]Reaction was carried out with **1a** (0.15 mmol), **2a** (0.10 mmol), and (*S*)-**C8** (5-10 mol%) in 1.0-2.0 mL cyclohexane for 24 hours at R.T., unless noted otherwise;. [b]Isolated yield; [c]Determined by HPLC analysis using a chiral stationary phase. [d]Reaction was allowed to stir at room temperature for 36 hours.

## Supplementary Table 5. Optimization of kinetic resolution conditions[a]

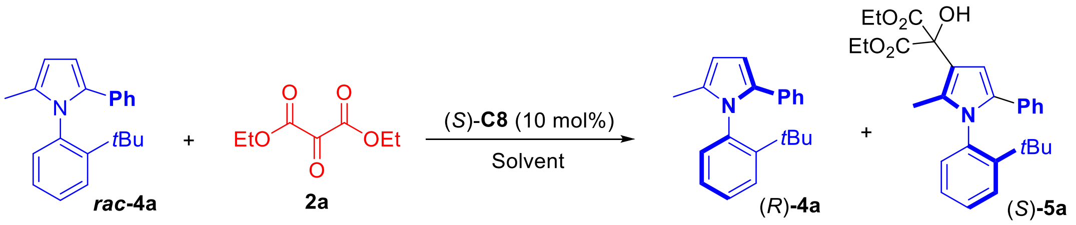


| entry | Solvent | T (h) | t (℃) | CPA | 4 | | 5 | | Conv. (%) | $S^d$ |
|---|---|---|---|---|---|---|---|---|---|---|
| | | | | | yield (%)[b] | **ee** (%)[c] | yield (%)[b] | **ee** (%)[c] | | |
| 1 | *c*-hexane | 26 | r.t. | (*S*)-**C6** | 74 | -27 | 21 | -91 | 22.9 | 28 |
| 2 | *c*-hexane | 26 | r.t. | (*R*)-**C7** | 62 | -52 | 35 | -89 | 36.9 | 29 |
| 3 | *c*-hexane | 26 | r.t. | (*S*)-**C8** | 65 | 45 | 31 | 92 | 32.8 | 37 |
| 4 | *c*-hexane | 26 | r.t. | (*S*)-**C15** | 62 | 34 | 33 | 61 | 35.8 | 6 |
| 5 | *c*-hexane | 76 | r.t. | (*S*)-**C8** | 61 | 53 | 35 | 92 | 36.6 | 41 |
| 6 | *c*-hexane | 120 | r.t. | (*S*)-**C8** | 56 | 62 | 40 | 89 | 41.1 | 32 |
| 7 | *c*-hexane | 67 | 40 | (*S*)-**C8** | 50 | 73 | 44 | 88 | 45.3 | 34 |
| 8 | $CCl_4$ | 68 | 40 | (*S*)-**C8** | 48 | 71 | 45 | 80 | 47.0 | 19 |
| 9 | THF | 68 | 40 | (*S*)-**C8** | *NR* | - | - | - | - | - |
| 10 | DCM | 68 | 40 | (*S*)-**C8** | 62 | 41 | 34 | 70 | 36.9 | 8 |
| 11 | EtOAc | 68 | 40 | (*S*)-**C8** | *NR* | - | - | - | - | - |
| 12 | *c*-hexane | 68 | 45 | (*S*)-**C8** | 53 | 71 | 44 | 86 | 45.2 | 28 |
| 13[e] | *c*-hexane | 84 | 40 | (*S*)-**C8** | 54 | 76 | 44 | 88 | 46.3 | 36 |
| 14[e] | *c*-hexane | 84 | 35 | (*S*)-**C8** | 53 | 72 | 43 | 90 | 44.4 | 41 |
| 15[e] | *c*-hexane | 84 | 30 | (*S*)-**C8** | 48 | 81 | 47 | 89 | 47.6 | 43 |
| 16[e,f] | *c*-hexane | 84 | 30 | (*S*)-**C8** | 50 | 78 | 45 | 88 | 47.0 | 37 |
| 17[e,g] | *c*-hexane | 84 | 30 | (*S*)-**C8** | 49 | 81 | 46 | 88 | 48.0 | 39 |

[a] All reactions were carried out with *rac*-**4** (0.20 mmol), **2a** (0.10 mmol), **CPA** (10 mol%) in cyclohexane (2.4 mL). [b] Isolated yield. [c] Determined by chiral stationary phase HPLC analysis. [d] The selectivity factor was calculated as $S = \ln[(1 - C)(1 - ee(\mathbf{4}))]/\ln[(1 - C)(1 + ee(\mathbf{4}))]$, $C = ee(\mathbf{4})/(ee(\mathbf{5}) + ee(\mathbf{4}))$. [e] reactions were carried out under argon. [f] *rac*-**4** (0.19 mmol) was used. [g] *rac*-**4** (0.18 mmol) was used.

## Supplementary Table 6. Studies of the configurational stability of (*R*)-3a

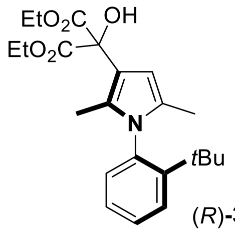

(*R*)-**3a**

| entry | T (℃) | Time (h) | ee (%)[a] | solvent |
|---|---|---|---|---|
| 1 | 25 | 24 | 95 | *i*PrOH |
| 2 | 50 | 24 | 95 | |
| 3 | 80 | 24 | 95 | |
| 4 | 100 | 24 | 95 | |
| 5 | 120 | 36 | 95 | |
| 6[b] | 150 | 12 | 95 | |
| 7[b] | 150 | 24 | 95 | |
| 8 | 80 | 36 | 95 | DCE |
| 9[b] | 100 | 24 | 95 | |
| 10[b] | 80 | 36 | 95 | toluene |
| 11[b] | 110 | 24 | 95 | |
| 12[b] | 130 | 24 | 95 | |
| 13[b] | 150 | 12 | 95 | |
| 14[b] | 150 | 24 | 95 | |

[a] The configurational stability of the product was studied by heating a solution of (*R*)-**3a** (0.02 mmol) in solvent (2.0 mL). [b] The ee values were determined by HPLC analysis using a chiral stationary phase. [c] Compound **3a** was partially decomposed.

## Supplementary Table 7. Studies of the configurational stability of (*S*)-5c

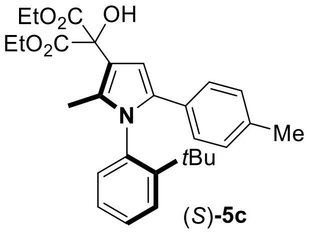


(*S*)-**5c**

| entry[a] | T (°C) | Time (h) | ee (%)[b] | solvent |
|---|---|---|---|---|
| 1 | 25 | 24 | 90 | *i*PrOH |
| 2 | 50 | 24 | 90 | |
| 3 | 80 | 24 | 90 | |
| 4 | 100 | 24 | 90 | |
| 5 | 120 | 36 | 90 | |
| 6[c] | 150 | 17 | 90 | |
| 7[e] | 150 | 30 | - | |
| 8 | 80 | 36 | 90 | DCE |
| 9 | 100 | 24 | 90 | |
| 10 | 110 | 24 | 90 | |
| 11 | 140 | 24 | 90 | |
| 12 | 150 | 30 | 90 | |
| 13[c] | 160 | 12 | 90 | |
| 14[c] | 160 | 16 | 90 | |
| 15[d] | 170 | 12 | - | |
| 16 | 80 | 36 | 90 | toluene |
| 17 | 110 | 24 | 90 | |
| 18 | 130 | 24 | 90 | |
| 19[c] | 150 | 17 | 90 | |
| 20[c] | 150 | 30 | 90 | |
| 21[d] | 160 | 12 | 88 | |
| 22[d] | 160 | 16 | 88 | |
| 23[e] | 170 | 12 | - | |

[a] The configurational stability of the product was studied by heating a solution of (*S*)-**5c** (0.02 mmol) in solvent (2.0 mL).[b] The ee values were determined by HPLC analysis using a chiral stationary phase. [c] Compound **5c** was partially decomposed. [d] Compound **5c** was mostly decomposed. [e] Compound **5c** was totally decomposed.

## Supplementary Table 8. Studies of the configurational stability of (*R*)-3o

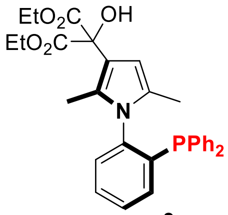


**3o**

| entry[a] | T (°C) | Time (h) | ee (%)[b] | solvent |
|---|---|---|---|---|
| 1 | 25 | 24 | 91 | |
| 2 | 50 | 24 | 91 | |
| 3 | 80 | 24 | 91 | |
| 4 | 100 | 24 | 91 | |
| 5 | 110 | 48 | 91 | |
| 6 | 120 | 16 | 91 | *i*PrOH |
| 7 | 120 | 32 | 91 | |
| 8[c] | 130 | 12 | 90 | |
| 9[c] | 130 | 36 | 89 | |
| 10[d] | 140 | 12 | 89 | |
| 11[e] | 140 | 36 | - | |
| 12 | 80 | 36 | 91 | |
| 13 | 100 | 24 | 91 | |
| 14 | 110 | 24 | 91 | DCE |
| 15 | 110 | 48 | 91 | |
| 16[c] | 120 | 16 | 90 | |
| 17[d] | 120 | 32 | - | |
| 18 | 80 | 36 | 91 | |
| 19 | 100 | 24 | 91 | |
| 20 | 100 | 48 | 91 | |
| 21 | 110 | 48 | 91 | toluene |
| 22[c] | 120 | 16 | 91 | |
| 23[c] | 120 | 32 | 90 | |
| 24[c] | 130 | 12 | 90 | |
| 25[d] | 130 | 36 | - | |

[a] The configurational stability of the product was studied by heating a solution of (*R*)-**3o** (0.02 mmol) in solvent (2.0 mL). [b] The ee values were determined by HPLC analysis using a chiral stationary phase. [c] Compound **3o** was partially decomposed. [d] Compound **3o** was mostly decomposed. [e] Compound **3o** was totally decomposed.

**Supplementary Table 9. Crystal data and structure refinement for 3s.**

| | |
|---|---|
| Identification code | **3s** |
| Empirical formula | $C_{25}H_{35}NO_5$ |
| Formula weight | 429.54 |
| Temperature/K | 100.0 |
| Crystal system | tetragonal |
| Space group | $P4_12_12$ |
| a/Å | 8.4043(9) |
| b/Å | 8.4043(9) |
| c/Å | 70.241(8) |
| α/° | 90 |
| β/° | 90 |
| γ/° | 90 |
| Volume/Å$^3$ | 4961.3(12) |
| Z | 8 |
| $\rho_{calc}$g/cm$^3$ | 1.150 |
| μ/mm$^{-1}$ | 0.639 |
| F(000) | 1856.0 |
| Crystal size/mm$^3$ | 0.4 × 0.36 × 0.34 |
| Radiation | CuKα (λ = 1.54178) |
| 2Θ range for data collection/° | 5.032 to 139.17 |
| Index ranges | $-10 \leq h \leq 10$, $-9 \leq k \leq 9$, $-84 \leq l \leq 84$ |
| Reflections collected | 32979 |
| Independent reflections | 4558 [$R_{int}$ = 0.1044, $R_{sigma}$ = 0.0687] |
| Data/restraints/parameters | 4558/0/293 |
| Goodness-of-fit on F$^2$ | 1.129 |
| Final R indexes [I>=2σ (I)] | $R_1$ = 0.0862, $wR_2$ = 0.1899 |
| Final R indexes [all data] | $R_1$ = 0.0998, $wR_2$ = 0.1997 |
| Largest diff. peak/hole / e Å$^{-3}$ | 0.53/-0.30 |
| Flack parameter | 0.0(2) |

**Supplementary Table 10. Crystal data and structure refinement for 5a.**

| | |
|---|---|
| Identification code | **5a** |
| Empirical formula | $C_{28}H_{32}FNO_5$ |
| Formula weight | 481.54 |
| Temperature/K | 100 |
| Crystal system | monoclinic |
| Space group | P2 |
| a/Å | 14.3321(13) |
| b/Å | 8.8694(8) |
| c/Å | 20.3683(19) |
| α/° | 90 |
| β/° | 102.448(3) |
| γ/° | 90 |
| Volume/$Å^3$ | 2528.3(4) |
| Z | 4 |
| $\rho_{calc}$g/$cm^3$ | 1.265 |
| μ/$mm^{-1}$ | 0.748 |
| F(000) | 1024.0 |
| Crystal size/$mm^3$ | 0.38 × 0.34 × 0.29 |
| Radiation | CuKα (λ = 1.54178) |
| 2Θ range for data collection/° | 6.316 to 136.886 |
| Index ranges | $-17 \leq h \leq 17$, $-10 \leq k \leq 10$, $-24 \leq l \leq 24$ |
| Reflections collected | 28554 |
| Independent reflections | 9197 [$R_{int}$ = 0.0398, $R_{sigma}$ = 0.0430] |
| Data/restraints/parameters | 9197/1/648 |
| Goodness-of-fit on $F^2$ | 1.035 |
| Final R indexes [I>=2σ (I)] | $R_1$ = 0.0374, $wR_2$ = 0.1049 |
| Final R indexes [all data] | $R_1$ = 0.0380, $wR_2$ = 0.1056 |
| Largest diff. peak/hole / e $Å^{-3}$ | 0.54/-0.22 |
| Flack parameter | -0.10(5) |

# Supplementary Note 1

## General Information

Chemicals were purchased from commercial suppliers and used without further purification unless otherwise stated. Chiral phosphoric acid (CPA) was purchased from Daicel Chiral Technologies (China). Analytical thin layer chromatography (TLC) was performed on precoated silica gel 60 GF254 plates. Flash column chromatography was performed using Tsingdao silica gel (60, particle size 0.040-0.063 mm). Visualization on TLC was achieved by use of UV light (254 nm) or iodine. NMR spectra were recorded on a Bruker DPX 400 spectrometer at 400/500 MHz for $^{1}$H NMR, 100/125 MHz for $^{13}$C NMR and 376 MHz for $^{19}$F NMR in $CDCl_3$, DMSO-$d_6$ with tetramethylsilane (TMS) as internal standard. The chemical shifts are expressed in ppm and coupling constants are given in Hz. Data for $^{1}$H NMR are recorded as follows: chemical shift (δ, ppm), multiplicity (s = singlet; d = doublet; t = triplet; q = quartet; p = pentet; m = multiplet; br = broad), coupling constant (Hz), integration. Data for $^{13}$C NMR are reported in terms of chemical shift (δ, ppm). Mass spectrometric data were obtained using Bruker Apex IV RTMS. The enantiomeric excess values were determined by chiral HPLC with an Agilent 1200 LC instrument and CHIRALPAK and CHIRALCEL columns. High resolution mass spectroscopy (HRMS) analyses were performed at a Bruker Daltonics. Inc mass instrument (ESI), Thermo Scientific. Q-Exactive (HESI) and Thermo Scientific. Orbitrap Fusion (HESI).

# Supplementary Note 2

## Synthesis of the symmetrical arylpyrroles (1a-1r)
## Procedure A

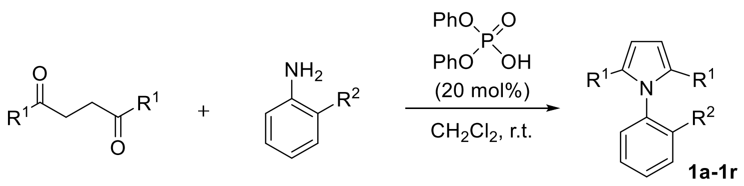


Diphenyl phosphonate (20 mol%) was added to a solution of 1,4-diketones (12.0 mmol) and aromatic amines (10.0 mmol) in methylene chloride. The mixture was stirred until aromatic amines completely consumed (monitored by TLC). The mixture was concentrated *in vacuo* and purified by flash chromatography on silica gel eluted with PE/EA to afford the corresponding *N*-arylpyrrole derivatives **1a-1r**.

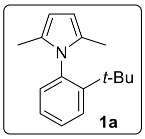


**1-(2-(*tert*-Butyl)phenyl)-2,5-dimethyl-1*H*-pyrrole (1a)**

**$^{1}$H NMR (500 MHz, $CDCl_3$)** δ 7.68 (d, *J* = 8.2 Hz, 1H), 7.45-7.42 (m, 1H), 7.30-7.27 (m, 1H), 6.99 (d, *J* = 7.7 Hz, 1H), 5.95 (s, 2H), 2.03 (s, 6H), 1.25 (s, 9H).

**$^{13}$C NMR (125 MHz, CDCl$_3$)** δ = 147.8, 136.2, 131.6, 129.7, 129.7, 128.5, 126.6, 105.4, 36.1, 31.8, 13.4.
**HRMS (ESI)** calcd for $[M+H]^+$ $C_{16}H_{22}N^+$, m/z: 228.1747, found: 228.1745.

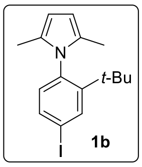


**1-(2-(*tert*-Butyl)-4-iodophenyl)-2,5-dimethyl-1*H*-pyrrole (1b)**
**$^1$H NMR (400 MHz, CDCl$_3$)** δ 7.91 (d, *J* = 2.0 Hz, 1H), 7.56 (dd, *J* = 8.2, 2.0 Hz, 1H), 6.64 (d, *J* = 8.2 Hz, 1H), 5.86 (s, 2H), 1.93 (s, 6H), 1.14 (s, 9H).
**$^{13}$C NMR (125 MHz, CDCl$_3$)** δ = 150.3, 139.0, 136.1, 135.8, 133.4, 129.6, 105.7, 94.6, 36.2, 31.5, 13.3.
**HRMS (ESI)** calcd for $[M+H]^+$ $C_{16}H_{21}IN^+$, m/z: 354.0713, found: 354.0707.

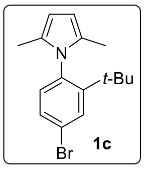


**1-(4-Bromo-2-(*tert*-butyl)phenyl)-2,5-dimethyl-1*H*-pyrrole (1c)**
**$^1$H NMR (400 MHz, CDCl$_3$)** δ 7.72 (d, *J* = 2.3 Hz, 1H), 7.36 (dd, *J* = 8.3, 2.3 Hz, 1H), 6.80 (d, *J* = 8.3 Hz, 1H), 5.87 (s, 2H), 1.94 (s, 6H), 1.15 (s, 9H).
**$^{13}$C NMR (100 MHz, CDCl$_3$)** δ = 150.2, 135.3, 133.2, 132.9, 129.8, 129.6, 122.5, 105.7, 36.3, 31.5, 13.3.
**HRMS (ESI)** calcd for $[M+H]^+$ $C_{16}H_{21}BrN^+$, m/z: 306.0852, found: 306.0849.

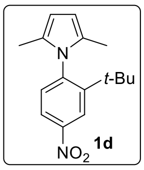


**1-(2-(*tert*-Butyl)-4-nitrophenyl)-2,5-dimethyl-1*H*-pyrrole (1d)**
**$^1$H NMR (400 MHz, CDCl$_3$)** δ 8.22 (dd, *J* = 2.6 Hz, 1H), 8.20 (dd, *J* = 2.6 Hz, 1H), 7.85 (d, *J* = 2.6 Hz, 1H), 7.80 (d, *J* = 9.0 Hz, 1H), 5.90 (s, 2H), 1.95 (s, 6H), 1.20 (s, 9H).
**$^{13}$C NMR (100 MHz, CDCl$_3$)** δ = 156.0, 146.0, 137.3, 131.1, 129.6, 126.7, 123.1, 106.4, 37.1, 31.4, 13.3.
**HRMS (ESI)** calcd for $[M+H]^+$ $C_{16}H_{21}N_2O_2^+$, m/z: 273.1598, found: 273.1595.

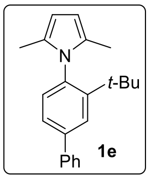

**1-(3-(*tert*-Butyl)-[1,1'-biphenyl]-4-yl)-2,5-dimethyl-1*H*-pyrrole (1e)**

**$^{1}$H NMR (400 MHz, CDCl$_3$)** δ 7.83 (d, *J* = 2.0 Hz, 1H), 7.73-7.61 (m, 2H), 7.51-7.45 (m, 3H), 7.42-7.38 (m, 1H), 7.01 (d, *J* = 8.0 Hz, 1H), 5.91 (s, 2H), 2.01 (s, 6H), 1.24 (s, 9H).

**$^{13}$C NMR (125 MHz, CDCl$_3$)** δ = 147.9, 141.1, 140.8, 135.4, 131.9, 129.8, 128.9, 128.6, 127.6, 127.2, 125.3, 105.4, 36.3, 31.8, 13.5.

**HRMS (ESI)** calcd for $[M+H]^+$ $C_{22}H_{26}N^+$, m/z: 304.2060, found: 304.2056.

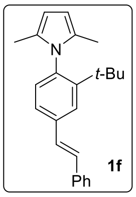


**(*E*)-1-(2-(*tert*-Butyl)-4-styrylphenyl)-2,5-dimethyl-1*H*-pyrrole (1f)**

**$^{1}$H NMR (500 MHz, CDCl$_3$)** δ 7.68 (d, *J* = 2.0 Hz, 1H), 7.59-7.53 (m, 2H), 7.44 (dd, *J* = 8.1, 2.0 Hz, 1H), 7.40-7.37 (m, 2H), 7.32-7.27 (m, 1H), 7.15 (s, 2H), 6.92 (d, *J* = 8.1 Hz, 1H), 5.88 (s, 2H), 1.97 (s, 6H), 1.20 (s, 9H).

**$^{13}$C NMR (125 MHz, CDCl$_3$)** δ = 147.9, 137.3, 137.2, 135.5, 131.9, 129.8, 129.5, 128.8, 128.6, 128.2, 127.9, 126.6, 123.9, 105.3, 36.1, 31.7, 13.4.

**HRMS (ESI)** calcd for $[M+H]^+$ $C_{24}H_{28}N^+$, m/z: 330.2216, found: 330.2213.

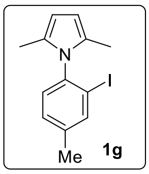


**1-(2-Iodo-4-methylphenyl)-2,5-dimethyl-1*H*-pyrrole (1g)**

**$^{1}$H NMR (400 MHz, CDCl$_3$)** δ 7.78 (d, *J* = 0.9 Hz, 1H), 7.29-7.21 (m, 1H), 7.15 (d, *J* = 7.9 Hz, 1H), 5.92 (s, 2H), 2.39 (s, 3H), 1.95 (s, 6H).

**$^{13}$C NMR (100 MHz, CDCl$_3$)** δ = 140.2, 139.9, 139.4, 130.0, 129.2, 128.2, 105.6, 100.4, 20.7, 12.9.

**HRMS (ESI)** calcd for $[M+H]^+$ $C_{13}H_{15}IN^+$, m/z: 312.0244, found: 312.0241.

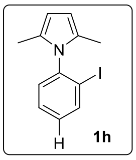


**1-(2-Iodophenyl)-2,5-dimethyl-1*H*-pyrrole (1h)**

**$^{1}$H NMR (400 MHz, CDCl$_3$)** δ 7.96 (d, *J* = 7.9 Hz, 1H), 7.48-7.44 (m, 1H), 7.30-7.28 (m, 1H), 7.17-7.13 (m, 1H), 5.94 (s, 2H), 1.96 (s, 6H).

**$^{13}$C NMR (100 MHz, CDCl$_3$)** δ = 142.2, 139.5, 129.9, 129.8, 129.1, 128.1, 105.7, 100.6, 12.9.

**HRMS (ESI)** calcd for $[M+H]^+$ $C_{12}H_{13}IN^+$, m/z: 298.0087, found: 298.0082.

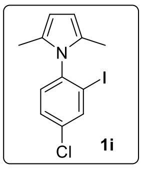

**1-(4-Chloro-2-iodophenyl)-2,5-dimethyl-1*H*-pyrrole (1i)**

**$^1$H NMR (400 MHz, $CDCl_3$)** δ 7.98 (d, *J* = 2.3 Hz, 1H), 7.47 (dd, *J* = 8.3, 2.3 Hz, 1H), 7.23 (d, *J* = 8.3 Hz, 1H), 5.96 (s, 2H), 1.99 (s, 6H).

**$^{13}$C NMR (100 MHz, $CDCl_3$)** δ = 141.0, 138.9, 134.9, 130.3, 129.4, 128.1, 106.2, 101.0, 12.9.

**HRMS (ESI)** calcd for $[M+H]^+$ $C_{12}H_{12}ClIN^+$, m/z: 331.9697, found: 331.9692.

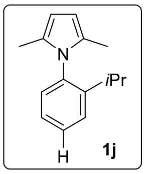

**1-(2-*iso*-Propylphenyl)-2,5-dimethyl-1*H*-pyrrole (1j)**

**$^1$H NMR (400 MHz, $CDCl_3$)** δ 7.54-7.37 (m, 2H), 7.34-7.21 (m, 1H), 7.18-7.07 (m, 1H), 5.93 (s, 2H), 2.54-2.44 (m, 1H), 1.95 (s, 6H), 1.15 (s, 3H), 1.13 (s, 3H).

**$^{13}$C NMR (100 MHz, $CDCl_3$)** δ = 147.6, 136.5, 129.0, 128.9, 128.9, 126.6, 126.4, 105.1, 27.4, 23.8, 12.8.

**HRMS (ESI)** calcd for $[M+H]^+$ $C_{15}H_{20}N^+$, m/z: 214.1590, found: 214.1588.

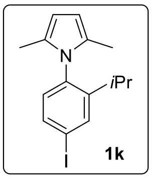

**1-(4-Iodo-2-*iso*-propylphenyl)-2,5-dimethyl-1*H*-pyrrole (1k)**

**$^1$H NMR (500 MHz, $CDCl_3$)** δ 7.74 (d, *J* = 2.0 Hz, 1H), 7.60 (dd, *J* = 8.2, 2.0 Hz, 1H), 6.85 (d, *J* = 8.2 Hz, 1H), 5.92 (s, 2H), 2.42 (hept, *J* = 6.9 Hz, 1H), 1.94 (s, 6H), 1.13 (s, 3H), 1.11 (s, 3H).

**$^{13}$C NMR (125 MHz, $CDCl_3$)** δ = 150.1, 136.4, 136.2, 135.7, 130.9, 128.7, 105.6, 94.8, 27.5, 23.6, 12.8.

**HRMS (ESI)** calcd for $[M+H]^+$ $C_{15}H_{19}IN^+$, m/z: 340.0557, found: 340.0551.

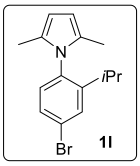

**1-(4-Bromo-2-*iso*-propylphenyl)-2,5-dimethyl-1*H*-pyrrole (1l)**

**$^1$H NMR (500 MHz, $CDCl_3$)** δ 7.56 (d, *J* = 2.2 Hz, 1H), 7.42 (dd, *J* = 8.3, 2.2 Hz, 1H), 7.01 (d, *J* = 8.3 Hz, 1H), 5.93 (s, 2H), 2.47 (hept, *J* = 6.9 Hz, 1H), 1.95 (s, 6H), 1.15 (s, 3H), 1.13 (s, 3H).

**$^{13}$C NMR (125 MHz, $CDCl_3$)** δ = 150.0, 135.7, 130.7, 130.1, 129.7, 128.8, 122.9, 105.7, 27.7, 23.6, 12.8.

**HRMS (ESI)** calcd for $[M+H]^+$ $C_{15}H_{19}BrN^+$, m/z: 292.0695, found: 292.0691.

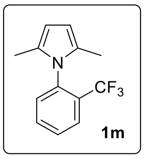

**2,5-Dimethyl-1-(2-(trifluoromethyl)phenyl)-1*H*-pyrrole (1m)**

**$^{1}$H NMR (400 MHz, $CDCl_3$)** δ 7.84 (d, *J* = 7.8 Hz, 1H), 7.70-7.67 (m, 1H), 7.61-7.57 (m, 1H), 7.28 (d, *J* = 7.8 Hz, 1H), 5.93 (s, 2H), 1.94 (s, 6H).

**$^{13}$C NMR (100 MHz, $CDCl_3$)** δ = 137.5, 132.9, 131.7, 130.0, 129.7 (q, J = 30.2 Hz), 128.9, 127.3 (q, J = 5.1 Hz), 123.0 (q, J = 272.0 Hz), 105.7, 12.5, 12.5.

**$^{19}$F NMR (376 MHz, $CDCl_3$)** δ = -61.44.

**HRMS (ESI)** calcd for $[M+H]^+$ $C_{13}H_{13}F_3N^+$, m/z: 240.0995, found: 240.0991.

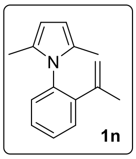

**2,5-Dimethyl-1-(2-(prop-1-en-2-yl)phenyl)-1*H*-pyrrole (1n)**

**$^{1}$H NMR (500 MHz, $CDCl_3$)** δ 7.48-7.42 (m, 1H), 7.43-7.35 (m, 2H), 7.21-7.13 (m, 1H), 5.91 (s, 2H), 5.16-5.00 (m, 1H), 4.91 (s, 1H), 1.99 (s, 6H), 1.58 (s, 3H).

**$^{13}$C NMR (125 MHz, $CDCl_3$)** δ = 144.3, 142.3, 136.1, 129.7, 129.7, 128.9, 128.3, 128.0, 116.3, 105.8, 21.5, 12.9.

**HRMS (ESI)** calcd for $[M+H]^+$ $C_{15}H_{18}N^+$, m/z: 212.1434, found: 212.1431.

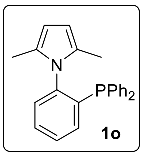

**1-(2-(Diphenylphosphanyl)phenyl)-2,5-dimethyl-1*H*-pyrrole (1o)**

**$^{1}$H NMR (400 MHz, $CDCl_3$)** δ 7.51-7.47 (m, 1H), 7.44-7.40 (m, 1H), 7.36-7.34 (m, 6H), 7.32-7.24 (m, 6H), 5.93 (s, 2H), 1.80 (s, 6H).

**$^{13}$C NMR (100 MHz, $CDCl_3$)** δ = 143.6 (d, *J* = 26.4 Hz), 139.3 (d, *J* = 14.8 Hz), 136.4 (d, *J* = 12.0 Hz), 134.9 (d, *J* = 2.3 Hz), 134.0 (d, *J* = 20.4 Hz), 130.06, 129.5 (d, *J* = 3.0 Hz), 128.97, 128.74, 128.59, 128.4 (d, *J* = 6.7 Hz), 105.5, 12.9, 12.9.

**$^{31}$P NMR (162 MHz, $CDCl_3$)** δ = -17.31.

**HRMS (ESI)** calcd for $[M+H]^+$ $C_{24}H_{23}NP^+$, m/z: 356.1563, found: 356.1566.

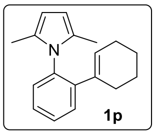

**2,5-Dimethyl-1-(2',3',4',5'-tetrahydro-[1,1'-biphenyl]-2-yl)-1*H*-pyrrole (1p)**

**$^{1}$H NMR (400 MHz, $CDCl_3$)** δ 7.40-7.27 (m, 3H), 7.18-7.08 (m, 1H), 5.86 (s, 2H), 5.70-5.49 (m, 1H), 2.12-2.08 (m, 2H), 1.96 (s, 6H), 1.63-1.62 (m, 2H), 1.54-1.51 (m, 4H).
**$^{13}$C NMR (100 MHz, $CDCl_3$)** δ = 143.5, 137.4, 135.9, 129.5, 129.5, 128.8, 128.1, 127.7, 127.1, 105.5, 27.0, 26.0, 23.0, 21.9, 12.9.
**HRMS (ESI)** calcd for $[M+H]^+$ $C_{18}H_{22}N^+$, m/z: 252.1747, found: 252.1741.

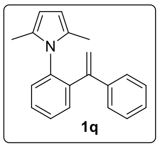


**2,5-Dimethyl-1-(2-(1-phenylvinyl)phenyl)-1*H*-pyrrole (1q)**
**$^{1}$H NMR (400 MHz, $CDCl_3$)** δ 7.50-7.33 (m, 3H), 7.23-7.12 (m, 4H), 7.08-7.05 (m, 2H), 5.66 (s, 2H), 5.39 (s, 1H), 5.04 (s, 1H), 1.83 (s, 6H).
**$^{13}$C NMR (100 MHz, $CDCl_3$)** δ = 147.8, 141.2, 140.9, 137.3, 131.3, 130.0, 128.4, 128.4, 128.0, 127.7, 127.4, 127.0, 117.2, 105.5, 12.9.
**$^{13}$C NMR-DEPT 135 (100 MHz, $CDCl_3$)** δ = 131.31, 129.96, 128.37, 128.01, 127.72, 127.44, 126.98, 117.18, 105.50, 12.87.
**HRMS (ESI)** calcd for $[M+H]^+$ $C_{20}H_{20}N^+$, m/z: 274.1590, found: 274.1593.

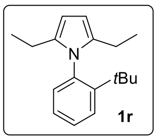


**1-(2-(*tert*-Butyl)phenyl)-2,5-diethyl-1*H*-pyrrole (1r)**
**$^{1}$H NMR (400 MHz, $CDCl_3$)** δ 7.62 (dd, *J* = 8.1, 1.1 Hz, 1H), 7.41-7.37 (m, 1H), 7.25-7.21 (m, 1H), 6.97 (dd, *J* = 7.7, 1.3 Hz, 1H), 5.95 (s, 2H), 2.32-2.15 (m, 4H), 1.23-1.10 (m, 15H).
**$^{13}$C NMR (100 MHz, $CDCl_3$)** δ = 147.6, 136.1, 135.9, 131.8, 129.7, 128.4, 126.4, 103.3, 36.1, 31.6, 20.7, 12.9.
**HRMS (ESI)** calcd for $[M+H]^+$ $C_{18}H_{26}N^+$, m/z: 256.2060, found: 256.2055.

## Synthesis of 2b and 2c

*Diisopropyl 2,2-dihydroxymalonate (**2b**) and di-tert-butyl 2,2-dihydroxymalonate(**2c**) were prepared according to the reference.*[1-2]

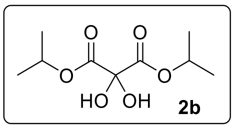


**Di-*iso*-propyl 2,2-dihydroxymalonate (2b)**
**$^{1}$H NMR (400 MHz, $CDCl_3$)** δ 5.15 (hept, *J* = 6.3 Hz, 2H), 4.80 (s, 2H), 1.30 (d, *J* = 6.3 Hz, 12H).
**$^{13}$C NMR (100 MHz, $CDCl_3$)** δ = 168.1, 89.9, 71.7, 21.4.
**HRMS (ESI)** calcd for $[M+Na]^+$ $C_9H_{16}NaO_6^+$, m/z: 243.0839, found: 243.0838.

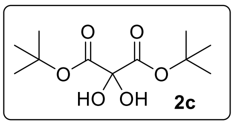

**Di-*tert*-butyl 2,2-dihydroxymalonate (2c)**

**$^1$H NMR (400 MHz, CDCl$_3$)** δ 4.76 (s, 2H), 1.51 (s, 18H).

**$^{13}$C NMR (100 MHz, CDCl$_3$)** δ = 167.8, 89.9, 84.4, 27.7.

**HRMS (ESI)** calcd for $[M+Na]^+$ $C_{11}H_{20}NaO_6^+$, m/z: 271.1152, found: 271.1151.

## Synthesis of the asymmetrical arylpyrroles (4a-4t)
## Procedure B

*asymmetrical arylpyrroles (**4a-4t**) were prepared according to the reference.*[3-4]

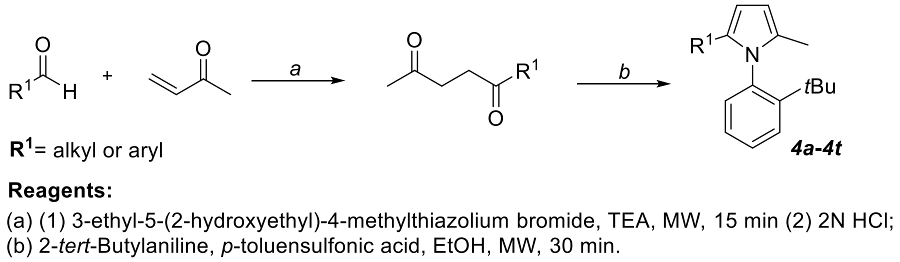


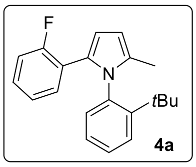

**1-(2-(*tert*-Butyl)phenyl)-2-(2-fluorophenyl)-5-methyl-1*H*-pyrrole (4a)**

**$^1$H NMR (400 MHz, CDCl$_3$)** δ 7.47 (dd, *J* = 8.1, 1.4 Hz, 1H), 7.39-7.29 (m, 1H), 7.29-7.16 (m, 2H), 7.08-6.91 (m, 2H), 6.85-6.66 (m, 2H), 6.48-6.46 (m, 1H), 6.10 (dd, *J* = 3.5, 0.7 Hz, 1H), 2.03 (s, 3H), 0.93 (s, 9H).

**$^{13}$C NMR (100 MHz, CDCl$_3$)** δ = 159.7 (d, *J* = 246.2 Hz), 147.5, 135.9, 132.6, 132.5, 130.0, 129.9, 128.6, 127.4, 127.3, 126.1, 123.2 (d, *J* = 3.7 Hz), 122.0 (d, *J* = 12.8 Hz), 115.8 (d, *J* = 23.2 Hz), 111.3 (d, *J* = 6.6 Hz), 107.0, 35.9, 31.2, 13.6.

**$^{19}$F NMR (376 MHz, CDCl$_3$)** δ = -112.92.

**HRMS (ESI)** calcd for $[M+H]^+$ $C_{21}H_{23}FN^+$, m/z: 308.1809, found: 308.1805.

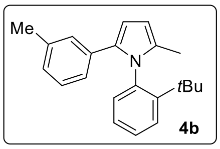

**1-(2-(*tert*-Butyl)phenyl)-2-methyl-5-(m-tolyl)-1*H*-pyrrole (4b)**

**$^1$H NMR (400 MHz, CDCl$_3$)** δ 7.53 (d, *J* = 8.1 Hz, 1H), 7.40-7.36 (m, 1H), 7.28-7.24 (m, 1H), 7.16 (d, *J* = 7.7 Hz, 1H), 6.98-6.95 (m, 1H), 6.89 (s, 1H), 6.85-6.80 (m, 2H), 6.41 (d, *J* = 3.4 Hz, 1H), 6.05 (d, *J* = 3.4 Hz, 1H), 2.15 (s, 3H), 2.02 (s, 3H), 0.96 (s, 9H).

**$^{13}$C NMR (100 MHz, CDCl$_3$)** δ = 147.5, 137.4, 136.6, 134.8, 133.9, 132.5, 132.2, 130.0, 128.5, 127.8, 127.7, 126.3, 126.2, 123.8, 107.8, 106.9, 36.0, 31.3, 21.4, 13.5.

**HRMS (ESI)** calcd for $[M+H]^+$ $C_{22}H_{26}N^+$, m/z: 304.2060, found: 304.2055.

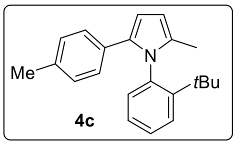


**1-(2-(*tert*-Butyl)phenyl)-2-methyl-5-(p-tolyl)-1*H*-pyrrole (4c)**

**$^{1}$H NMR (400 MHz, CDCl$_3$)** δ 7.52 (d, *J* = 8.1 Hz, 1H), 7.39-7.35 (m, 1H), 7.27-7.24 (m, 1H), 7.16 (d, *J* = 7.7 Hz, 1H), 6.95-6.88 (m, 4H), 6.36 (d, *J* = 3.4 Hz, 1H), 6.04 (d, *J* = 3.1 Hz, 1H), 2.21 (s, 3H), 2.01 (s, 3H), 0.95 (s, 9H).

**$^{13}$C NMR (100 MHz, CDCl$_3$)** δ = 147.5, 136.6, 135.0, 134.9, 132.3, 132.2, 131.2, 130.0, 128.8, 128.5, 126.8, 126.3, 107.5, 106.8, 36.0, 31.3, 21.0, 13.5.

**HRMS (ESI)** calcd for $[M+H]^+$ $C_{22}H_{26}N^+$, m/z: 304.2060, found: 304.2054.

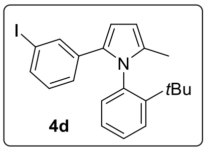


**1-(2-(*tert*-Butyl)phenyl)-2-(3-iodophenyl)-5-methyl-1*H*-pyrrole (4d)**

**$^{1}$H NMR (400 MHz, CDCl$_3$)** δ 7.62 (d, *J* = 8.2 Hz, 1H), 7.47-7.45 (m, 2H), 7.38-7.31 (m, 2H), 7.17 (d, *J* = 7.7 Hz, 1H), 7.04 (d, *J* = 7.9 Hz, 1H), 6.86-6.82 (m, 1H), 6.49 (d, *J* = 3.5 Hz, 1H), 6.11 (d, *J* = 2.9 Hz, 1H), 2.08 (s, 3H), 1.03 (s, 9H).

**$^{13}$C NMR (100 MHz, CDCl$_3$)** δ = 147.5, 136.2, 136.1, 135.4, 134.1, 133.4, 132.8, 132.0, 130.3, 129.8, 129.0, 126.7, 125.5, 108.9, 107.5, 94.3, 36.1, 31.4, 13.6.

**HRMS (ESI)** calcd for $[M+H]^+$ $C_{21}H_{23}IN^+$, m/z: 416.0870, found: 416.0864.

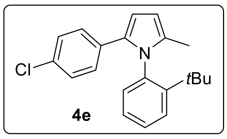


**1-(2-(*tert*-Butyl)phenyl)-2-(4-chlorophenyl)-5-methyl-1*H*-pyrrole (4e)**

**$^{1}$H NMR (400 MHz, CDCl$_3$)** δ 7.54 (d, *J* = 8.1 Hz, 1H), 7.42-7.38 (m, 1H), 7.30-7.26 (m, 1H), 7.16 (d, *J* = 7.7 Hz, 1H), 7.05 (d, *J* = 8.6 Hz, 2H), 6.95 (d, *J* = 8.6 Hz, 2H), 6.40 (d, *J* = 3.4 Hz, 1H), 6.05 (d, *J* = 3.4 Hz, 1H), 2.01 (s, 3H), 0.95 (s, 9H).

**$^{13}$C NMR (100 MHz, CDCl$_3$)** δ = 147.6, 136.3, 133.5, 133.1, 132.6, 132.1, 131.1, 130.2, 128.8, 128.2, 127.8, 126.5, 108.4, 107.2, 36.0, 31.3, 13.5.

**HRMS (ESI)** calcd for $[M+H]^+$ $C_{21}H_{23}ClN^+$, m/z: 324.1514, found: 324.1511.

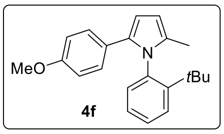


**1-(2-(*tert*-Butyl)phenyl)-2-(4-methoxyphenyl)-5-methyl-1*H*-pyrrole (4f)**

**$^1$H NMR (400 MHz, $CDCl_3$)** δ 7.52 (dd, *J* = 8.1, 1.2 Hz, 1H), 7.40-7.36 (m, 1H), 7.29-7.25 (m, 1H), 7.18 (dd, *J* = 7.7, 1.3 Hz, 1H), 6.97 (d, *J* = 8.9 Hz, 2H), 6.65 (d, *J* = 8.9 Hz, 2H), 6.30 (d, *J* = 3.4 Hz, 1H), 6.04 (d, *J* = 3.4 Hz, 1H), 3.72 (s, 3H), 2.02 (s, 3H), 0.95 (s, 9H).
**$^{13}$C NMR (100 MHz, $CDCl_3$)** δ = 157.6, 147.6, 136.5, 134.7, 132.3, 131.8, 130.0, 128.5, 128.3, 127.0, 126.3, 113.5, 106.9, 106.7, 55.1, 36.0, 31.3, 13.5.
**HRMS (ESI)** calcd for $[M+H]^+$ $C_{22}H_{26}NO^+$, m/z: 320.2009, found: 320.2005.

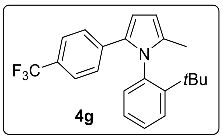


**1-(2-(*tert*-Butyl)phenyl)-2-methyl-5-(4-(trifluoromethyl)phenyl)-1*H*-pyrrole (4g)**
**$^1$H NMR (400 MHz, $CDCl_3$)** δ 7.58 (dd, *J* = 8.2, 1.3 Hz, 1H), 7.45-7.41 (m, 1H), 7.35-7.28 (m, 3H), 7.18-7.12 (m, 3H), 6.54 (d, *J* = 3.6 Hz, 1H), 6.10 (d, *J* = 3.6 Hz, 1H), 2.04 (s, 3H), 0.97 (s, 9H).
**$^{13}$C NMR (100 MHz, $CDCl_3$)** δ = 147.5, 137.3, 136.3, 134.1, 133.1, 132.0, 130.3, 129.0, 126.7, 126.9 (q, *J* = 32.3 Hz), 126.1, 125.1 (q, *J* = 3.8 Hz), 124.4 (q, *J* = 269.9 Hz), 109.6, 107.7, 36.0, 31.3, 13.5.
**$^{19}$F NMR (376 MHz, $CDCl_3$)** δ = -62.37.
**HRMS (ESI)** calcd for $[M+H]^+$ $C_{22}H_{23}F_3N^+$, m/z: 358.1777, found: 358.1773.

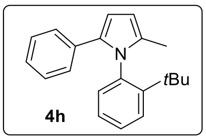


**1-(2-(*tert*-Butyl)phenyl)-2-methyl-5-phenyl-1*H*-pyrrole (4h)**
**$^1$H NMR (400 MHz, $CDCl_3$)** δ 7.52 (dd, *J* = 8.1, 1.3 Hz, 1H), 7.40-7.36 (m, 1H), 7.29-7.25 (m, 1H), 7.18 (dd, *J* = 7.7, 1.4 Hz, 1H), 7.10-7.00 (m, 5H), 6.40 (d, *J* = 3.5 Hz, 1H), 6.05 (d, *J* = 4.0 Hz, 1H), 2.02 (s, 3H), 0.94 (s, 9H).
**$^{13}$C NMR (125 MHz, $CDCl_3$)** δ = 147.5, 136.5, 134.8, 134.0, 132.6, 132.3, 130.1, 128.6, 128.0, 126.9, 126.4, 125.4, 108.0, 107.0, 36.0, 31.2, 13.5.
**HRMS (ESI)** calcd for $[M+H]^+$ $C_{21}H_{24}N^+$, m/z: 290.1903, found: 290.1900.

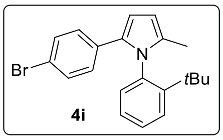


**2-(4-Bromophenyl)-1-(2-(*tert*-butyl)phenyl)-5-methyl-1*H*-pyrrole (4i)**
**$^1$H NMR (400 MHz, $CDCl_3$)** δ 7.54 (d, *J* = 8.1 Hz, 1H), 7.42-7.38 (m, 1H), 7.30-7.26 (m, 1H), 7.20 (d, *J* = 8.6 Hz, 2H), 7.15 (d, *J* = 7.7 Hz, 1H), 6.89 (d, *J* = 8.6 Hz, 2H), 6.41 (d, *J* = 3.6 Hz, 1H), 6.06 (d, *J* = 3.4 Hz, 1H), 2.01 (s, 3H), 0.95 (s, 9H).
**$^{13}$C NMR (100 MHz, $CDCl_3$)** δ = 147.5, 136.3, 133.4, 133.2, 133.0, 132.1, 131.1, 130.2, 128.8, 128.1, 126.5, 119.2, 108.4, 107.3, 36.0, 31.3, 13.5.
.**HRMS (ESI)** calcd for $[M+H]^+$ $C_{21}H_{23}BrN^+$, m/z: 368.1008, found: 368.1007.

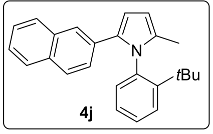

4j

**1-(2-(*tert*-Butyl)phenyl)-2-methyl-5-(naphthalen-2-yl)-1*H*-pyrrole (4j)**

**$^1$H NMR (500 MHz, CDCl$_3$)** δ 7.67-7.65 (m, 1H), 7.60 (d, *J* = 8.7 Hz, 1H), 7.55 (d, *J* = 8.1 Hz, 1H), 7.44-7.37 (m, 3H), 7.32-7.30 (m, 3H), 7.25-7.22 (m, 2H), 6.57 (d, *J* = 3.5 Hz, 1H), 6.11 (d, *J* = 3.3 Hz, 1H), 2.06 (s, 3H), 0.96 (s, 9H).

**$^{13}$C NMR (125 MHz, CDCl$_3$)** δ = 147.6, 136.8, 134.5, 133.4, 133.2, 132.2, 131.4, 131.3, 130.2, 128.7, 127.9, 127.5, 127.3, 126.6, 125.8, 125.8, 125.1, 124.1, 108.7, 107.3, 36.1, 31.3, 13.6.

**HRMS (ESI)** calcd for $[M+H]^+$ $C_{25}H_{26}N^+$, m/z: 340.2060, found: 340.2055.

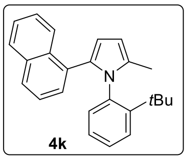

4k

**1-(2-(*tert*-Butyl)phenyl)-2-methyl-5-(naphthalen-1-yl)-1*H*-pyrrole (4k)**

**$^1$H NMR (400 MHz, CDCl$_3$)** δ 8.47 (d, *J* = 8.1 Hz, 1H), 7.77-7.75 (m, 1H), 7.58 (d, *J* = 8.0 Hz, 1H), 7.48-7.37 (m, 3H), 7.28-7.16 (m, 3H), 7.15-7.11 (m, 1H), 7.08-7.06 (m, 1H), 6.42 (d, *J* = 3.4 Hz, 1H), 6.17 (d, *J* = 2.9 Hz, 1H), 2.08 (s, 3H), 0.86 (s, 9H).

**$^{13}$C NMR (100 MHz, CDCl$_3$)** δ = 147.5, 135.9, 134.0, 133.0, 132.7, 132.0, 131.5, 131.2, 129.8, 128.3, 128.2, 127.8, 127.0, 126.8, 125.8, 125.7, 125.4, 124.6, 111.8, 106.5, 36.0, 31.3, 13.8.

**HRMS (ESI)** calcd for $[M+H]^+$ $C_{25}H_{26}N^+$, m/z: 340.2060, found: 340.2056.

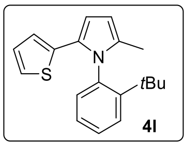

4l

**1-(2-(*tert*-Butyl)phenyl)-2-methyl-5-(thiophen-2-yl)-1*H*-pyrrole (4l)**

**$^1$H NMR (500 MHz, CDCl$_3$)** δ 7.64 (d, *J* = 8.1 Hz, 1H), 7.50-7.47 (m, 1H), 7.31 (t, *J* = 7.5 Hz, 1H), 7.15 (d, *J* = 7.7 Hz, 1H), 6.94 (d, *J* = 5.1 Hz, 1H), 6.78-6.76 (m, 1H), 6.49 (d, *J* = 3.6 Hz, 1H), 6.39 (d, *J* = 3.6 Hz, 1H), 6.07 (d, *J* = 3.5 Hz, 1H), 2.06 (s, 3H), 1.10 (s, 9H).

**$^{13}$C NMR (125 MHz, CDCl$_3$)** δ = 148.1, 136.8, 135.9, 132.5, 132.3, 130.1, 129.2, 129.1, 126.8, 126.7, 122.3, 121.9, 107.7, 107.3, 36.2, 31.5, 13.5.

**HRMS (ESI)** calcd for $[M+H]^+$ $C_{19}H_{22}NS^+$, m/z: 296.1467, found: 296.1463.

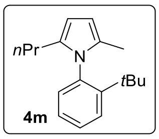

4m

**1-(2-(*tert*-Butyl)phenyl)-2-methyl-5-propyl-1*H*-pyrrole (4m)**

**$^1$H NMR (400 MHz, CDCl$_3$)** δ 7.61 (dd, *J* = 8.2, 1.4 Hz, 1H), 7.40-7.36 (m, 1H), 7.24-7.20 (m, 1H), 6.93 (dd, *J* = 7.7, 1.5 Hz, 1H), 5.90-5.88 (m, 2H), 2.26-2.08 (m, 2H), 1.94 (s, 3H), 1.62-1.52 (m, 2H), 1.15 (s, 9H), 0.88 (t, *J* = 7.4 Hz, 3H).

**$^{13}$C NMR (100 MHz, CDCl$_3$)** δ = 147.7, 136.0, 134.9, 131.7, 129.7, 129.3, 128.4, 126.4, 105.2, 104.0, 36.1, 31.7, 29.7, 22.0, 14.2, 13.2.

**HRMS (ESI)** calcd for $[M+H]^+$ $C_{18}H_{26}N^+$, m/z: 256.2060, found: 256.2056.

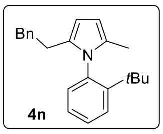


**1-(2-(*tert*-Butyl)phenyl)-2-methyl-5-phenethyl-1*H*-pyrrole (4n)**

**$^{1}$H NMR (400 MHz, CDCl$_3$)** δ 7.57 (dd, *J* = 8.2, 1.4 Hz, 1H), 7.37-7.32 (m, 1H), 7.23-7.11 (m, 4H), 7.06-7.04 (m, 2H), 6.85 (dd, *J* = 7.7, 1.5 Hz, 1H), 5.98 (d, *J* = 3.3 Hz, 1H), 5.92 (d, *J* = 3.3 Hz, 1H), 2.88-2.83 (m, 2H), 2.61-2.53 (m, 1H), 2.46-2.38 (m, 1H), 1.94 (s, 3H), 1.14 (s, 9H).

**$^{13}$C NMR (100 MHz, CDCl$_3$)** δ = 147.7, 142.1, 135.8, 134.1, 131.7, 129.7, 129.7, 128.6, 128.5, 128.4, 126.6, 125.9, 105.5, 104.4, 36.1, 35.5, 31.7, 30.0, 13.3.

**HRMS (ESI)** calcd for $[M+H]^+$ $C_{23}H_{28}N^+$, m/z: 318.2216, found: 318.2213.

# Supplementary Note 3

## General procedure for preparation of racemic compound 3

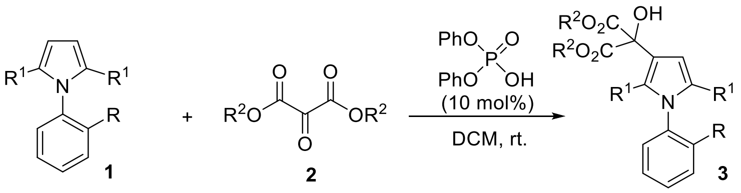

An oven-dried 10 mL of Schlenk tube was charged with arylpyrroles **1** (0.15 mmol), 1 mL of $CH_2Cl_2$ and diphenyl phosphate (0.01 mmol) at ambient temperature. Then, ketomalonate **2** (0.10 mmol) was added to the above solution and the mixture was stirred until the starting material was completely consumed. The mixture was concentrated under reduced pressure and purified by flash column chromatography (ethyl acetate/petroleum ether) to afford the corresponding racemic product **3**.

## General procedure for the asymmetric synthesis of compound (*R*)-3

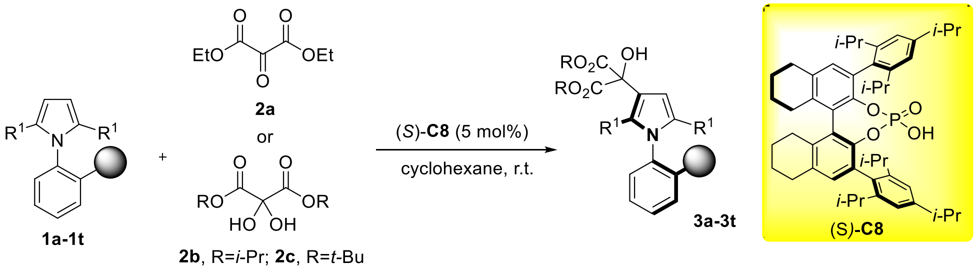

An oven-dried 10 mL of Schlenk tube was charged with arylpyrroles **1** (0.30 mmol), (*S*)-**C8** (0.01 mmol), 2.0 mL of dry cyclohexane, and the mixture was stirred at ambient temperature for 10 min. A solution of ketomalonate **2a** (0.20 mmol) in dry cyclohexane (1.0 mL) was added dropwise to the above solution and the mixture was stirred until the starting material was completely consumed. Then the mixture was concentrated under reduced pressure and purified by flash chromatography eluted with PE/EA (10/1 to 5/1) to afford the corresponding axially chiral arylpyrrole products (*R*)-**3**.

For **3g**-**3i**, **3m**, **3r**, the reaction conditions are as follows: an oven-dried 10 mL of Schlenk tube was charged with arylpyrroles **1** (0.30 mmol), (*S*)-**C8** (0.02 mmol) and 1.5 mL of mixed solvent (0.75 mL cyclohexane/0.75mL methylcyclohexane). After the mixture was stirred at -30 ℃ for 30 min, a solution of ketomalonate **2a** (0.20 mmol) in 1.5 mL of mixed solvent (0.75 mL cyclohexane/0.75mL methylcyclohexane) was added dropwise to the above solution and the mixture was stirred until the starting material was completely consumed. Then the mixture was concentrated under reduced pressure and purified by flash chromatography eluted with PE/EA (10/1 to 5/1) to afford the corresponding axially chiral arylpyrrole products.

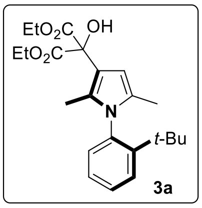

**(*R*)-Diethyl 2-(1-(2-(*tert*-butyl)phenyl)-2,5-dimethyl-1*H*-pyrrol-3-yl)-2-hydroxymalonate ((*R*)-3a)**

According to the general procedure, *(R)*-**3a** was obtained in **96% yield** with **95% ee**.

**$^1$H NMR (400 MHz, CDCl$_3$)** δ 7.58 (d, *J* = 8.1 Hz, 1H), 7.37-7.34 (m, 1H), 7.22-7.18 (m, 1H), 6.90 (d, *J* = 7.7 Hz, 1H), 5.95 (s, 1H), 4.43-4.21 (m, 4H), 4.06 (s, 1H), 1.89 (s, 6H), 1.33-1.26 (m, 6H), 1.13 (s, 9H).

**$^{13}$C NMR (100 MHz, CDCl$_3$)** δ = 171.0, 170.8, 147.7, 135.4, 131.3, 129.7, 128.7, 128.1, 128.0, 126.6, 115.0, 105.5, 78.2, 62.5, 62.5, 36.1, 31.7, 14.1, 14.1, 13.1, 12.5.

**HRMS (ESI)** calcd for $[M+H]^+$ $C_{23}H_{32}NO_5^+$, m/z: 402.2275, found: 402.2271.

**HPLC analysis**: DAICEL CHIRALPAK AD-3, hexane/isopropyl alcohol = 95/05, flow rate = 1.0 mL/min, λ = 254 nm), $t_R$ (minor) = 14.3 min, $t_R$ (major) = 16.0 min, ee = 95%.

*Chiral HPLC spectrum of racemic **3a***

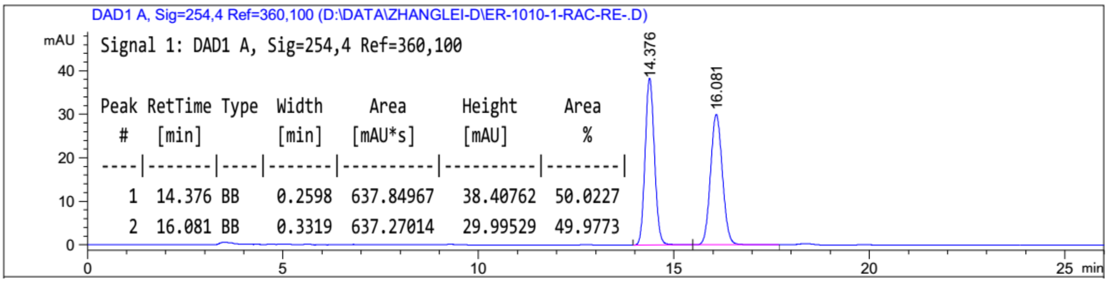


*Chiral HPLC spectrum of (R)-**3a***

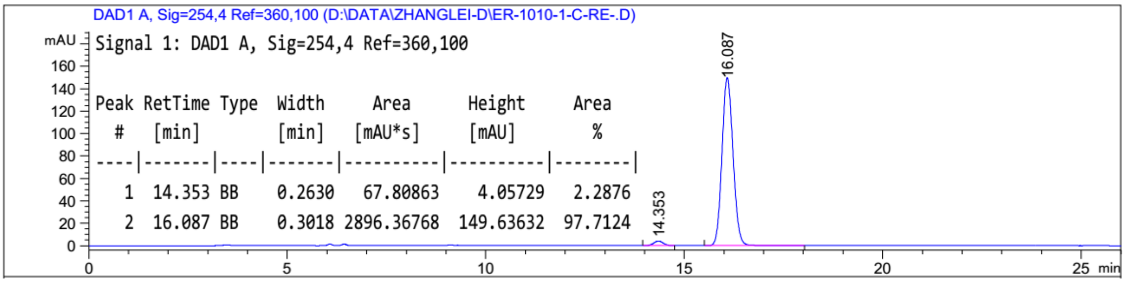


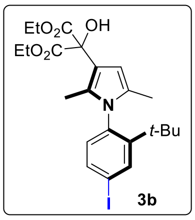


**(*R*)-Diethyl 2-(1-(2-(*tert*-butyl)-4-iodophenyl)-2,5-dimethyl-1*H*-pyrrol-3-yl)-2-hydroxymalonate ((*R*)-3b)**

According to the general procedure, *(R)*-**3b** was obtained in **99% yield** with **96% ee**.

**$^1$H NMR (500 MHz, CDCl$_3$)** δ 7.88 (d, *J* = 2.0 Hz, 1H), 7.53 (dd, *J* = 8.2, 2.0 Hz, 1H), 6.64 (d, *J* = 8.2 Hz, 1H), 5.94 (d, *J* = 0.5 Hz, 1H), 4.39-4.20 (m, 4H), 4.05 (s, 1H), 1.88 (s, 3H), 1.87 (s, 3H), 1.35-1.22 (m, 6H), 1.10 (s, 9H).

**$^{13}$C NMR (125 MHz, CDCl$_3$)** δ = 170.8, 170.6, 150.2, 139.1, 135.9, 135.4, 133.3, 128.0, 127.9, 115.4, 105.9, 95.0, 78.1, 62.5, 62.5, 36.2, 31.5, 14.1, 14.1, 13.2, 12.5.

**$^{13}$C NMR-DEPT 135 (125 MHz, CDCl$_3$)** δ = 139.1, 135.9, 133.3, 105.9, 62.5, 62.5, 31.5, 14.1, 14.1, 13.2, 12.5.

**HRMS (ESI)** calcd for $[M+H]^+$ $C_{23}H_{31}INO_5^+$, m/z: 528.1241, found: 528.1242.

**HPLC analysis**: DAICEL CHIRALPAK IC, hexane/isopropyl alcohol = 85/15, flow rate = 1.0 mL/min, λ = 230 nm), $t_R$ (major) = 25.8 min, $t_R$ (minor) = 32.5 min, ee = 96%.

*Chiral HPLC spectrum of racemic **3b***

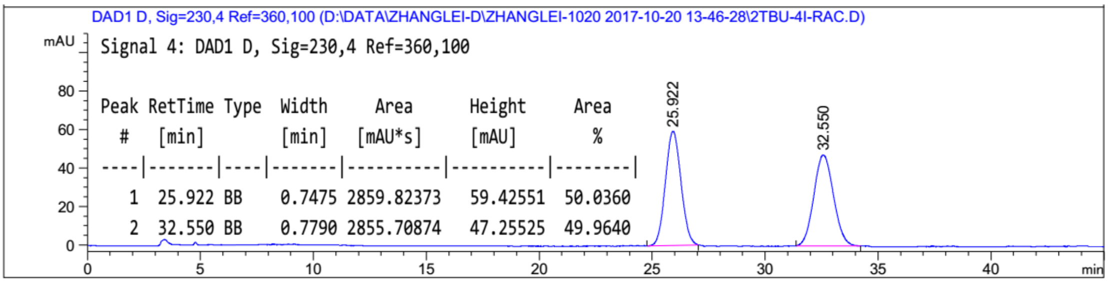


*Chiral HPLC spectrum of (R)-**3b***

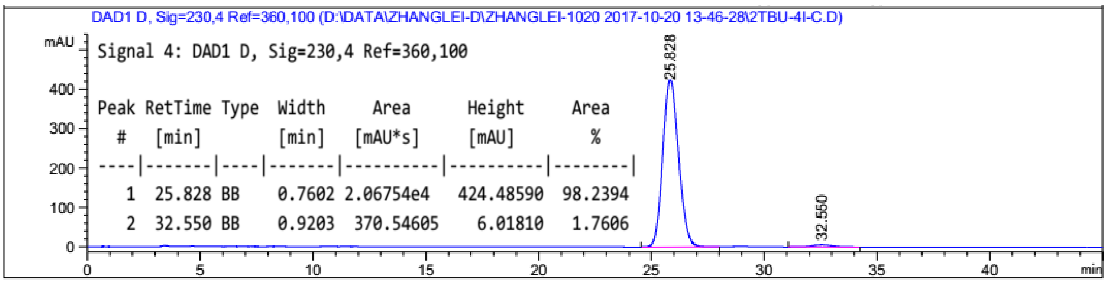


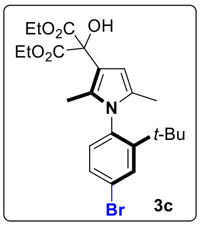


***(R)*-Diethyl 2-(1-(4-bromo-2-(*tert*-butyl)phenyl)-2,5-dimethyl-1*H*-pyrrol-3-yl)-2-hydroxymalonate ((*R*)-3c)**

According to the general procedure, *(R)*-**3c** was obtained in **95% yield** with **97% ee**.

**$^1$H NMR (500 MHz, CDCl$_3$)** δ 7.69 (d, *J* = 2.3 Hz, 1H), 7.34 (dd, *J* = 8.3, 2.3 Hz, 1H), 6.79 (d, *J* = 8.3 Hz, 1H), 5.95 (d, *J* = 0.7 Hz, 1H), 4.45-4.19 (m, 4H), 4.05 (s, 1H), 1.88 (s, 3H), 1.87 (s, 3H), 1.32-1.26 (m, 6H), 1.11 (s, 9H).

**$^{13}$C NMR (125 MHz, CDCl$_3$)** δ = 170.9, 170.6, 150.1, 134.7, 133.1, 133.0, 129.9, 128.1, 128.0, 122.8, 115.4, 105.9, 78.1, 62.6, 62.5, 36.3, 31.5, 14.1, 14.1, 13.1, 12.5.

**HRMS (ESI)** calcd for $[M+H]^+$ $C_{23}H_{31}BrNO_5^+$, m/z: 480.1380, found: 480.1373.

**HPLC analysis**: DAICEL CHIRALPAK IC, hexane/isopropyl alcohol = 85/15, flow rate = 1.0 mL/min, λ = 230 nm), $t_R$ (major) = 25.0 min, $t_R$ (minor) = 31.4 min, ee = 97%.

*Chiral HPLC spectrum of racemic **3c***

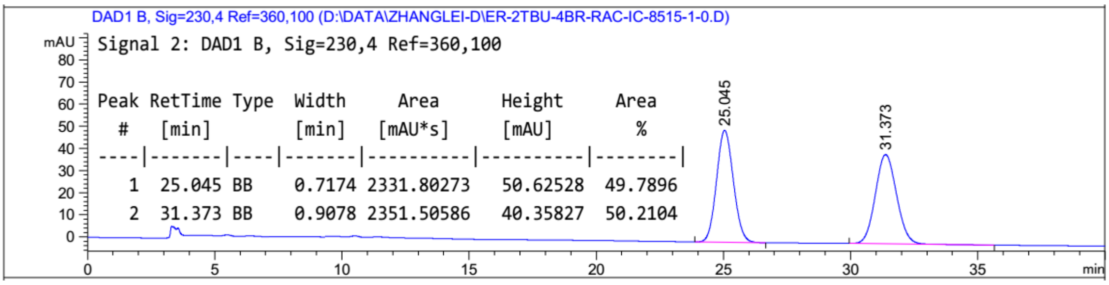


*Chiral HPLC spectrum of (R)-**3c***

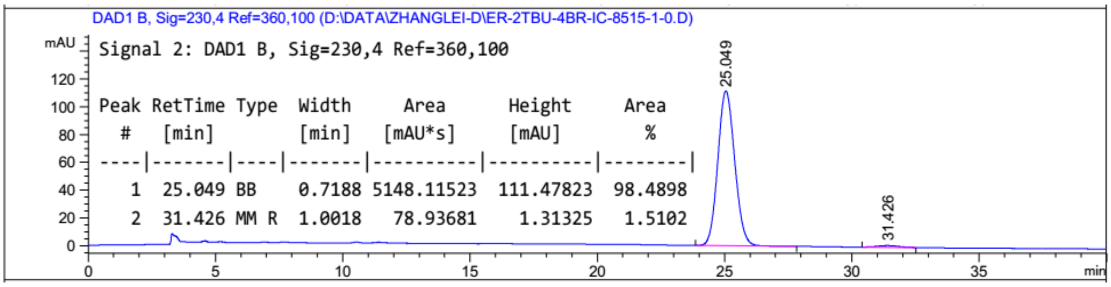


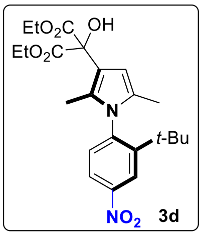


**(*R*)-Diethyl 2-(1-(2-(*tert*-butyl)-4-nitrophenyl)-2,5-dimethyl-1*H*-pyrrol-3-yl)-2-hydroxymalonate ((*R*)-3d)**

According to the general procedure, *(R)*-**3d** was obtained in **82% yield** with **97% ee**.

**$^1$H NMR (500 MHz, CDCl$_3$)** δ 8.20 (dd, *J* = 9.0, 2.5 Hz, 1H), 7.84 (d, *J* = 2.0 Hz, 1H), 7.79 (d, *J* = 9.0 Hz, 1H), 6.01 (s, 1H), 4.39-4.24 (m, 4H), 4.08 (s, 1H), 1.90 (s, 6H), 1.30 (dt, *J* = 11.5, 7.1 Hz, 6H), 1.17 (s, 9H).

**$^{13}$C NMR (125 MHz, CDCl$_3$)** δ = 170.7, 170.5, 156.0, 146.0, 136.6, 131.2, 128.1, 128.0, 126.6, 123.3, 115.9, 106.6, 78.0, 62.6, 37.0, 31.4, 14.1, 14.1, 13.2, 12.6.

**HRMS (ESI)** calcd for $[M+H]^+$ $C_{23}H_{31}N_2O_7^+$, m/z: 447.2126, found: 447.2127.

**HPLC analysis**: DAICEL CHIRALPAK AD-3, hexane/isopropyl alcohol = 85/15, flow rate = 1.0 mL/min, λ = 254 nm), $t_R$ (minor) = 12.4 min, $t_R$ (major) = 19.3 min, ee = 97%.

*Chiral HPLC spectrum of racemic **3d***

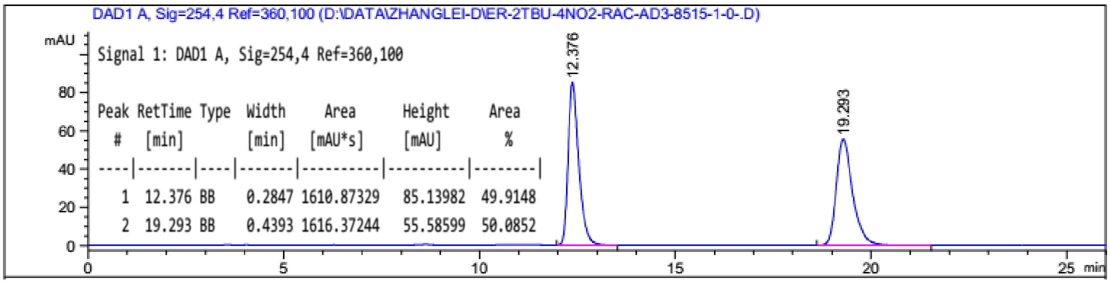


*Chiral HPLC spectrum of (R)-**3d***

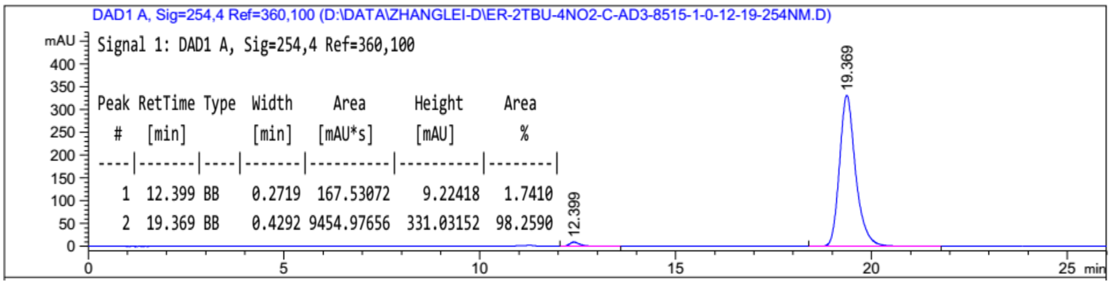


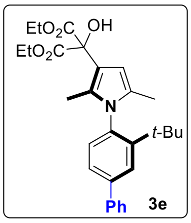


**(R)-Diethyl2-(1-(3-(*tert*-butyl)-[1,1'-biphenyl]-4-yl)-2,5-dimethyl-1*H*-pyrrol-3-yl)-2-hydroxymalonate((*R*)-3e)**

According to the general procedure, *(R)*-**3e** was obtained in **98% yield** with **96% ee**.

**$^{1}$H NMR (500 MHz, CDCl$_3$)** δ 7.81 (d, *J* = 1.9 Hz, 1H), 7.71-7.58 (m, 2H), 7.46 (dt, *J* = 8.1, 4.8 Hz, 3H), 7.38 (t, *J* = 7.4 Hz, 1H), 7.00 (d, *J* = 8.0 Hz, 1H), 6.00 (s, 1H), 4.52-4.24 (m, 4H), 4.10 (s, 1H), 1.96 (s, 6H), 1.33 (dt, *J* = 12.9, 7.1 Hz, 6H), 1.20 (s, 9H).

**$^{13}$C NMR (125 MHz, CDCl$_3$)** δ = 171.0, 170.8, 147.9, 141.4, 140.6, 134.7, 131.9, 128.9, 128.7, 128.2, 128.2, 127.6, 127.2, 125.4, 115.1, 105.6, 78.2, 62.6, 62.5, 36.3, 31.8, 14.1, 14.1, 13.3, 12.6.

**HRMS (ESI)** calcd for $[M+H]^+$ $C_{29}H_{36}NO_5^+$, m/z: 478.2588, found: 478.2587.

**HPLC analysis**: DAICEL CHIRALPAK IC, hexane/isopropyl alcohol = 85/15, flow rate = 1.0 mL/min, λ = 254 nm), $t_R$ (major) = 33.4 min, $t_R$ (minor) = 42.8 min, ee = 96%.

*Chiral HPLC spectrum of racemic **3e***

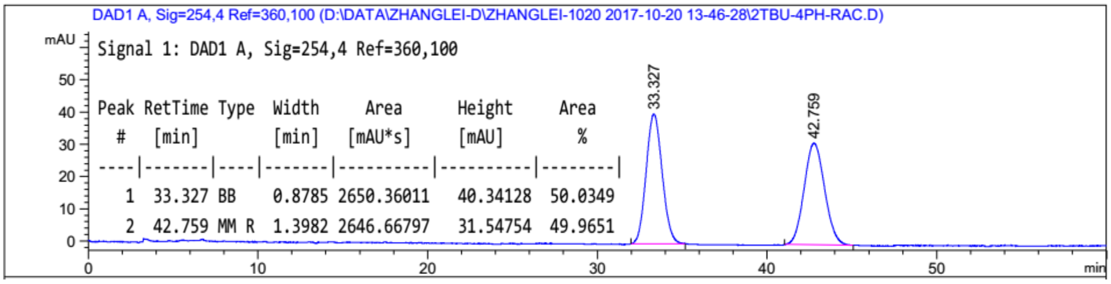


*Chiral HPLC spectrum of (R)-**3e***

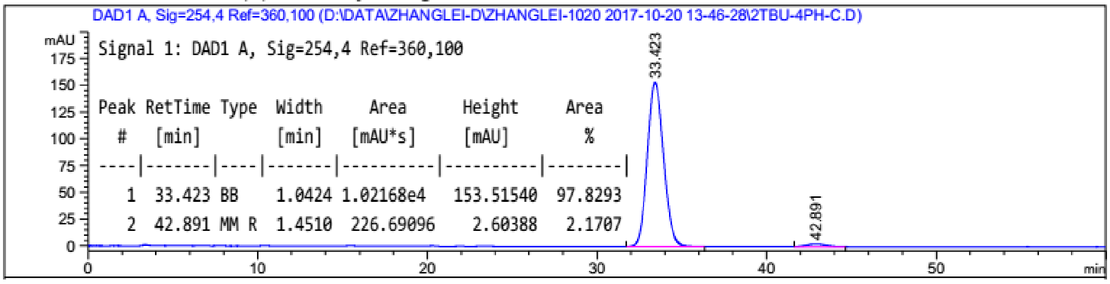

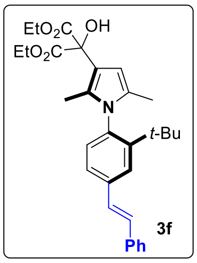


**(R)-Diethyl (E)-2-(1-(2-(*tert*-butyl)-4-styrylphenyl)-2,5-dimethyl-1*H*-pyrrol-3-yl)-2-hydroxymalonate(*(R)*-3f)**

According to the general procedure, ***(R)*-3f** was obtained in **97% yield** with **94% ee**.

**$^{1}$H NMR (500 MHz, $CDCl_3$)** δ 7.69 (d, $J$ = 1.8 Hz, 1H), 7.56-7.54 (m, 2H), 7.44 (dd, $J$ = 8.1, 1.8 Hz, 1H), 7.40-7.37 (m, 2H), 7.30-7.27 (m, 1H), 7.16 (s, 2H), 6.94 (d, $J$ = 8.1 Hz, 1H), 6.00 (s, 1H), 4.43-4.25 (m, 4H), 4.12 (s, 1H), 1.95 (s, 6H), 1.35-1.30 (m, 6H), 1.20 (s, 9H).

**$^{13}$C NMR (125 MHz, $CDCl_3$)** δ = 171.0, 170.8, 147.9, 137.6, 137.1, 134.8, 131.9, 129.7, 128.8, 128.7, 128.2, 128.1, 128.0, 127.9, 126.6, 124.0, 115.2, 105.6, 78.2, 62.5, 62.5, 36.1, 31.7, 14.1, 14.1, 13.2, 12.5.

**HRMS (ESI)** calcd for $[M+H]^+$ $C_{31}H_{38}NO_5^+$, m/z: 504.2744, found: 504.2745.

**HPLC analysis**: DAICEL CHIRALCEL OD-3, hexane/isopropyl alcohol = 98/02, flow rate = 1.0 mL/min, λ = 270 nm), $t_R$ (major) = 15.1 min, $t_R$ (minor) = 30.8 min, ee = 94%.

*Chiral HPLC spectrum of racemic **3f***

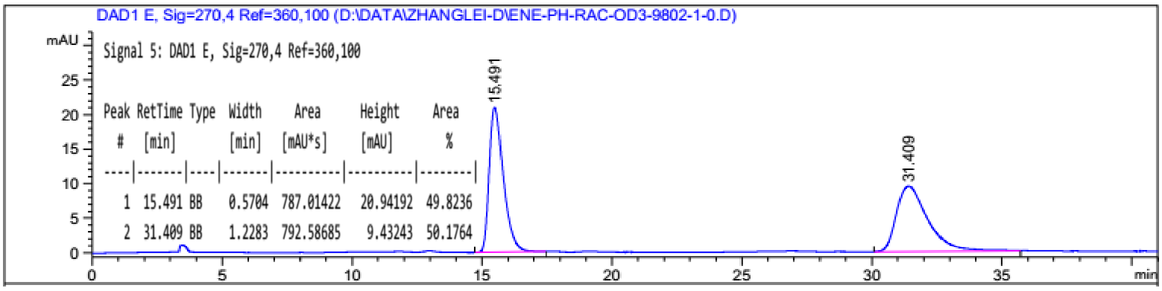


*Chiral HPLC spectrum of (R)-**3f***

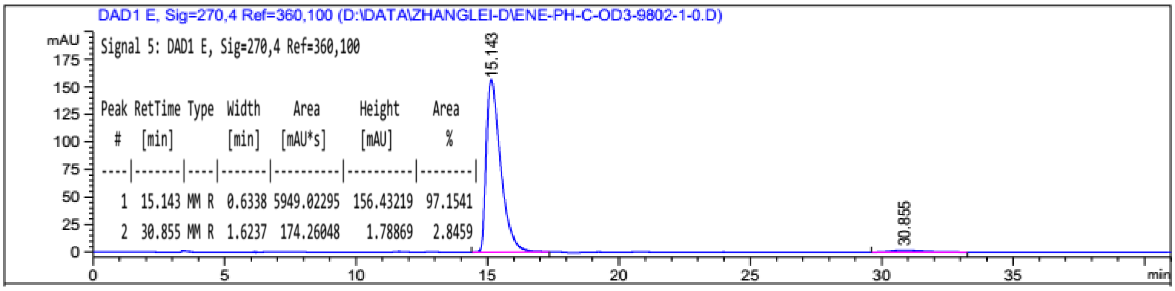


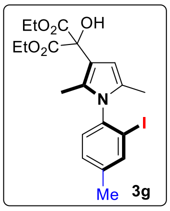


**(R)-Diethyl 2-hydroxy-2-(1-(2-iodo-4-methylphenyl)-2,5-dimethyl-1*H*-pyrrol-3-yl)malonate**

**((*R*)-3g)**

According to the general procedure, *(R)*-**3g** was obtained in **98% yield** with **88% ee**.

**$^{1}$H NMR (400 MHz, $CDCl_3$)** δ 7.76 (d, *J* = 1.0 Hz, 1H), 7.24 (dd, *J* = 7.9, 1.0 Hz, 1H), 7.14 (d, *J* = 7.9 Hz, 1H), 6.00 (s, 1H), 4.40-4.25 (m, 4H), 4.05 (s, 1H), 2.38 (s, 3H), 1.89 (s, 6H), 1.32 (q, *J* = 7.2 Hz, 6H).

**$^{13}$C NMR (100 MHz, $CDCl_3$)** δ = 170.9, 170.7, 140.4, 140.0, 138.9, 130.0, 129.2, 126.8, 126.7, 115.3, 105.8, 100.2, 78.1, 62.6, 62.6, 20.7, 14.2, 14.2, 12.7, 11.9.

**HRMS (ESI)** calcd for $[M+H]^+$ $C_{20}H_{25}INO_5^+$, m/z: 486.0772, found: 486.0762.

**HPLC analysis**: DAICEL CHIRALPAK AD-3, hexane/isopropyl alcohol = 95/05, flow rate = 1.0 mL/min, λ = 230 nm), $t_R$ (major) = 21.1 min, $t_R$ (minor) = 25.1 min, ee = 88%.

*Chiral HPLC spectrum of racemic **3g***

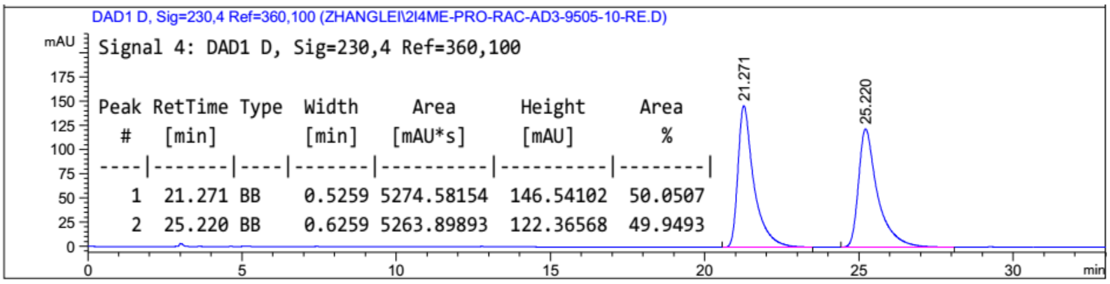


*Chiral HPLC spectrum of (R)-**3g***

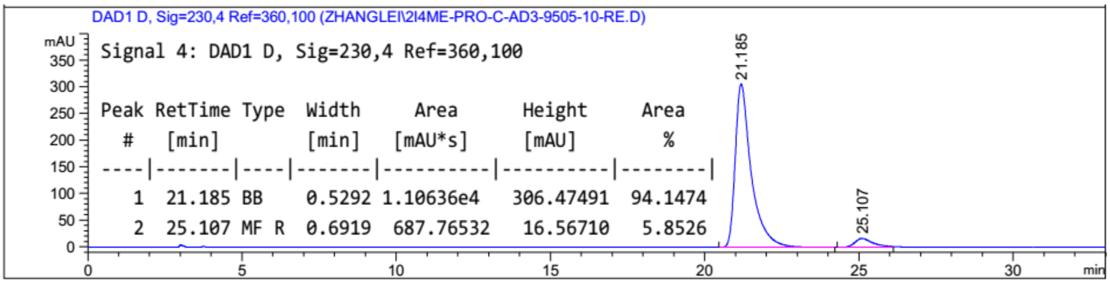


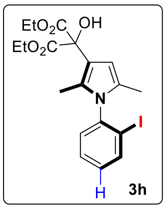


**(*R*)-Diethyl 2-hydroxy-2-(1-(2-iodophenyl)-2,5-dimethyl-1*H*-pyrrol-3-yl)malonate((*R*)-3h)**

According to the general procedure, *(R)*-**3h** was obtained in **96% yield** with **90% ee**.

The reaction gave *(R)*-**3h** was obtained in **95% yield** with **89% ee** at a 0.8 mmol scale. The coupling reactions were performed with this batch of *(R)*-**3h** as the starting material.

**$^{1}$H NMR (400 MHz, $CDCl_3$)** δ 7.95 (d, *J* = 7.9 Hz, 1H), 7.48-7.44 (m, 1H), 7.29-7.27 (m, 1H), 7.17-7.13 (m, 1H), 6.03 (s, 1H), 4.41-4.25 (m, 4H), 4.12 (s, 1H), 1.91 (s, 6H), 1.35-1.30 (m, 6H).

**$^{13}$C NMR (100 MHz, $CDCl_3$)** δ = 170.8, 170.6, 141.6, 139.6, 130.1, 129.8, 129.2, 126.7, 126.5, 115.4, 106.0, 100.5, 78.1, 62.6, 62.5, 14.2, 14.2, 12.7, 11.9.

**HRMS (ESI)** calcd for $[M+H]^+$ $C_{19}H_{23}INO_5^+$, m/z: 472.0615, found: 472.0614.

**HPLC analysis**: DAICEL CHIRALPAK AD-3, hexane/isopropyl alcohol = 94/06, flow rate = 1.0 mL/min, λ = 254 nm), $t_R$ (major) = 26.7 min, $t_R$ (minor) = 32.1 min, ee = 90%.

*Chiral HPLC spectrum of racemic **3h***

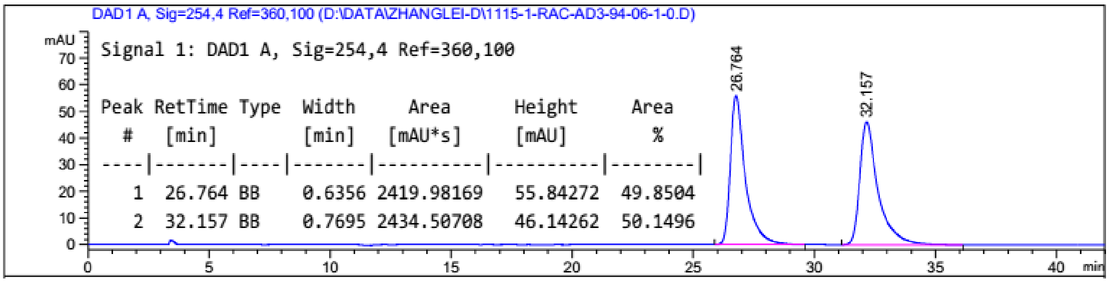


*Chiral HPLC spectrum of (R)-**3h***

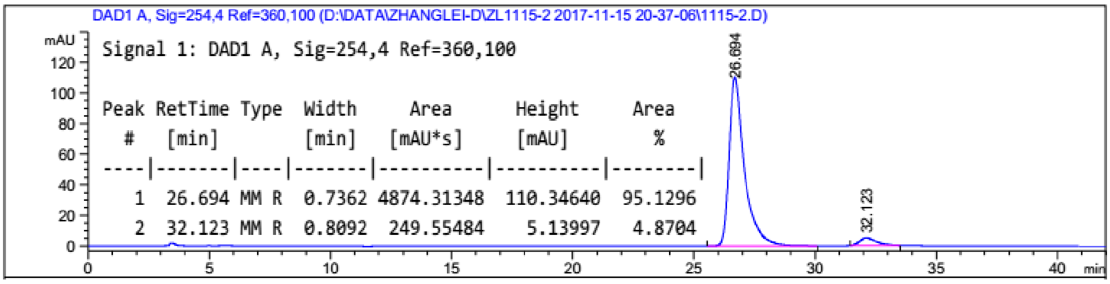


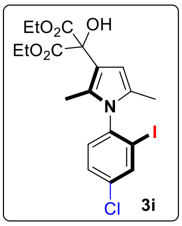


***(R)*-Diethyl 2-(1-(4-chloro-2-iodophenyl)-2,5-dimethyl-1*H*-pyrrol-3-yl)-2-hydroxymalonate ((*R*)-3i)**

According to the general procedure, *(R)*-**3i** was obtained in **97% yield** with **89% ee**.

**$^{1}$H NMR (400 MHz, CDCl$_3$)** δ 7.92 (d, *J* = 2.1 Hz, 1H), 7.43 (dd, *J* = 8.3, 2.1 Hz, 1H), 7.19 (d, *J* = 8.3 Hz, 1H), 6.01 (s, 1H), 4.43-4.21 (m, 4H), 4.07 (s, 1H), 1.89 (s, 3H), 1.89 (s, 3H), 1.31 (q, *J* = 7.1 Hz, 6H).

**$^{13}$C NMR (100 MHz, CDCl$_3$)** δ = 170.7, 170.6, 140.4, 139.0, 135.0, 130.3, 129.5, 126.7, 126.5, 115.7, 106.4, 100.9, 78.0, 62.6, 14.2, 14.2, 12.7, 11.9.

**HRMS (ESI)** calcd for $[M+H]^+$ $C_{19}H_{22}ClINO_5^+$, m/z: 506.0226, found: 506.0225.

**HPLC analysis**: DAICEL CHIRALPAK AD-3, hexane/isopropyl alcohol = 95/05, flow rate = 1.0 mL/min, λ = 240 nm), $t_R$ (major) = 23.9 min, $t_R$ (minor) = 30.9 min, ee = 89%.

*Chiral HPLC spectrum of racemic **3i***

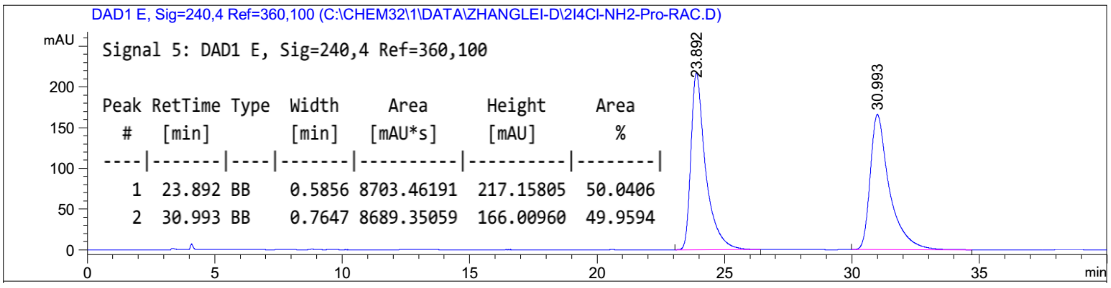

*Chiral HPLC spectrum of (R)-**3i***

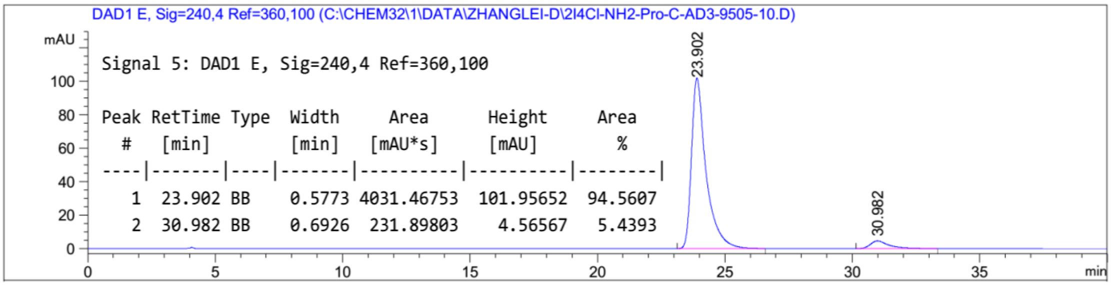


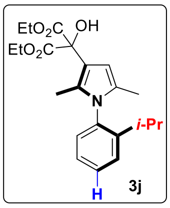


**(*R*)-Diethyl 2-hydroxy-2-(1-(2-*iso*propylphenyl)-2,5-dimethyl-1*H*-pyrrol-3-yl)malonate ((*R*)-3j)**

According to the general procedure, *(R)*-**3j** was obtained in **95% yield** with **91% ee**.

**$^1$H NMR (400 MHz, CDCl$_3$)** δ 7.42 (dd, *J* = 4.9, 1.0 Hz, 2H), 7.32-7.20 (m, 1H), 7.10 (d, *J* = 7.6 Hz, 1H), 5.99 (d, *J* = 0.8 Hz, 1H), 4.44-4.26 (m, 4H), 4.05 (s, 1H), 2.45 (dd, *J* = 13.8, 6.9 Hz, 1H), 1.89 (s, 3H), 1.88 (s, 3H), 1.35-1.30 (m, 6H), 1.12-1.09 (m, 6H).

**$^{13}$C NMR (100 MHz, CDCl$_3$)** δ = 170.9, 170.8, 147.5, 136.0, 129.1, 129.0, 127.4, 127.3, 126.7, 126.4, 114.8, 105.4, 78.2, 62.6, 27.3, 23.7, 23.7, 14.1, 12.6, 11.8.

**HRMS (ESI)** calcd for $[M+H]^+$ $C_{22}H_{30}NO_5^+$, m/z: 388.2118, found: 388.2116.

**HPLC analysis**: DAICEL CHIRALPAK AD-3, hexane/isopropyl alcohol = 98/02, flow rate = 1.0 mL/min, λ = 254 nm), $t_R$ (minor) = 18.4 min, $t_R$ (major) = 20.5 min, ee = 91%.

*Chiral HPLC spectrum of racemic **3j***

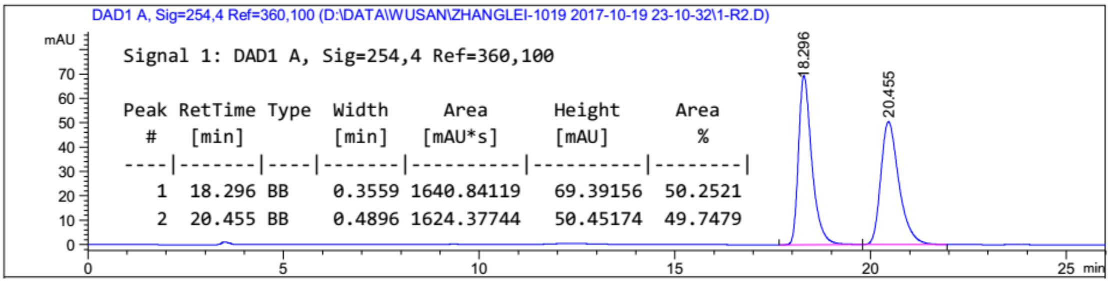


*Chiral HPLC spectrum of (R)-**3j***

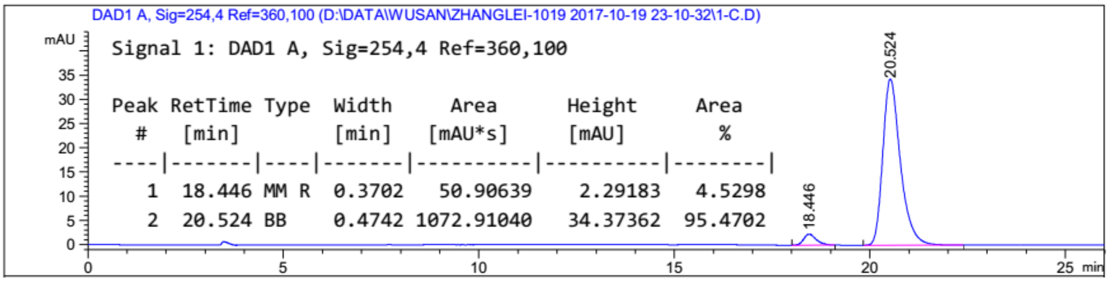

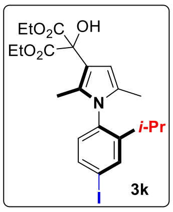


**(R)-Diethyl 2-hydroxy-2-(1-(4-iodo-2-*iso*propylphenyl)-2,5-dimethyl-1*H*-pyrrol-3-yl)malonate ((R)-3k)**

According to the general procedure, *(R)*-**3k** was obtained in **95% yield** with **92% ee**.

**$^1$H NMR (400 MHz, CDCl$_3$)** δ 7.71 (d, *J* = 1.2 Hz, 1H), 7.58 (dd, *J* = 8.1, 1.2 Hz, 1H), 6.82 (d, *J* = 8.2 Hz, 1H), 5.97 (s, 1H), 4.42-4.22 (m, 4H), 4.06 (s, 1H), 2.42-2.35 (m, 1H), 1.86 (s, 6H), 1.32-1.27 (m, 6H), 1.16-1.00 (m, 6H).

**$^{13}$C NMR (125 MHz, CDCl$_3$)** δ = 170.8, 170.6, 150.0, 136.2, 136.0, 135.8, 130.9, 127.2, 127.1, 115.2, 105.9, 95.1, 78.1, 62.6, 27.4, 23.6, 23.5, 14.1, 14.1, 12.6, 11.8.

**HRMS (ESI)** calcd for $[M+H]^+$ $C_{22}H_{29}INO_5^+$, m/z: 514.1085, found: 514.1088.

**HPLC analysis**: DAICEL CHIRALPAK ID, hexane/isopropyl alcohol = 95/05, flow rate = 0.8 mL/min, λ = 254 nm), $t_R$ (major) = 39.2 min, $t_R$ (minor) = 43.0 min, ee = 92%.

*Chiral HPLC spectrum of racemic **3k***

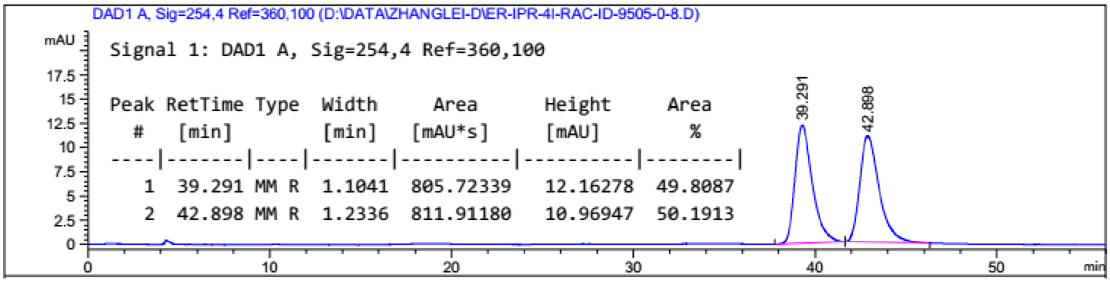


*Chiral HPLC spectrum of (R)-**3k***

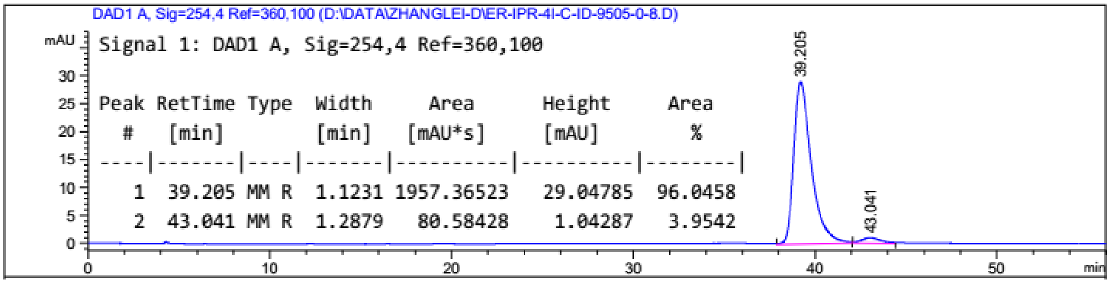


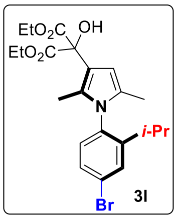


**(R)-Diethyl 2-(1-(4-bromo-2-*iso*propylphenyl)-2,5-dimethyl-1*H*-pyrrol-3-yl)-2-hydroxymalonate ((R)-3l)**

According to the general procedure, *(R)*-**3l** was obtained in **91% yield** with **93% ee**.

**$^{1}$H NMR (400 MHz, CDCl$_3$)** δ 7.52 (d, *J* = 2.2 Hz, 1H), 7.38 (dd, *J* = 8.3, 2.2 Hz, 1H), 6.97 (d, *J* = 8.3 Hz, 1H), 5.98 (s, 1H), 4.39-4.25 (m, 4H), 4.06 (s, 1H), 2.55-2.28 (m, 1H), 1.86 (s, 6H), 1.32-1.27 (m, 6H), 1.09-1.06 (m, 6H).

**$^{13}$C NMR (125 MHz, CDCl$_3$)** δ = 170.7, 170.6, 149.8, 135.1, 130.7, 130.1, 129.8, 127.2, 127.1, 123.1, 115.2, 105.9, 78.1, 62.6, 27.6, 23.6, 23.5, 14.1, 14.1, 12.6, 11.8.

**HRMS (ESI)** calcd for $[M+H]^+$ $C_{22}H_{29}BrNO_5^+$, m/z: 466.1224, found: 466.1218.

**HPLC analysis**: DAICEL CHIRALPAK ID, hexane/isopropyl alcohol = 95/05, flow rate = 0.8 mL/min, λ = 254 nm), $t_R$ (major) = 38.0 min, $t_R$ (minor) = 42.0 min, ee = 93%.

*Chiral HPLC spectrum of racemic **3l***

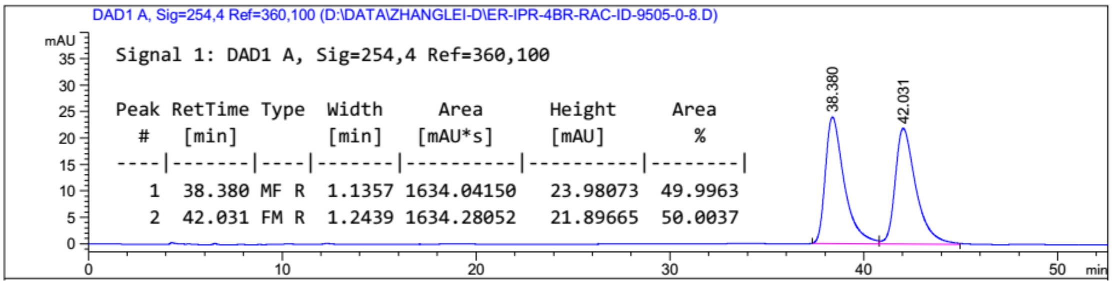


*Chiral HPLC spectrum of (R)-**3l***

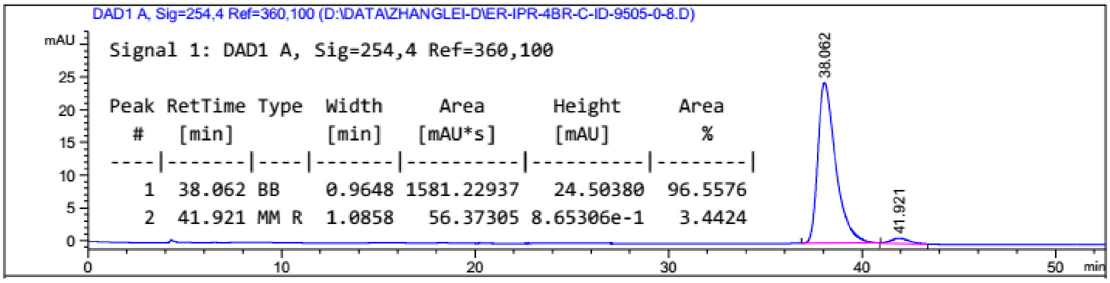


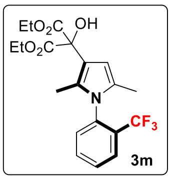


**(*R*)-Diethyl 2-(2,5-dimethyl-1-(2-(trifluoromethyl)phenyl)-1*H*-pyrrol-3-yl)-2-hydroxymalonate ((*R*)-3m)**

According to the general procedure, *(R)*-**3m** was obtained in **93% yield** with **89% ee**.

**$^{1}$H NMR (400 MHz, CDCl$_3$)** δ 7.82 (d, *J* = 7.6 Hz, 1H), 7.68 (t, *J* = 7.3 Hz, 1H), 7.59 (t, *J* = 7.6 Hz, 1H), 7.28 (d, *J* = 7.6 Hz, 1H), 6.01 (s, 1H), 4.45-4.18 (m, 4H), 3.92 (s, 1H), 1.88 (s, 3H), 1.87 (s, 3H), 1.34-1.28 (m, 6H).

**$^{13}$C NMR (100 MHz, CDCl$_3$)** δ = 170.9, 170.6, 136.8, 133.0, 131.7, 129.3 (q, *J* = 30.4 Hz), 129.2, 128.4, 128.2, 127.3 (q, *J* = 5.0 Hz), 122.8 (q, *J* = 272.1 Hz), 115.2, 105.9, 78.0, 62.6, 62.6, 14.0, 14.0, 12.3, 11.6.

**$^{19}$F NMR (376 MHz, CDCl$_3$)** δ = -61.41.

**HRMS (ESI)** calcd for $[M+H]^+$ $C_{20}H_{23}F_3NO_5^+$, m/z: 414.1523, found: 414.1521.

**HPLC analysis**: DAICEL CHIRALPAK AD-3, hexane/isopropyl alcohol = 96/04, flow rate = 1.0 mL/min, λ = 230 nm), $t_R$ (major) = 26.5 min, $t_R$ (minor) = 34.3 min, ee = 89%.

*Chiral HPLC spectrum of racemic **3m***

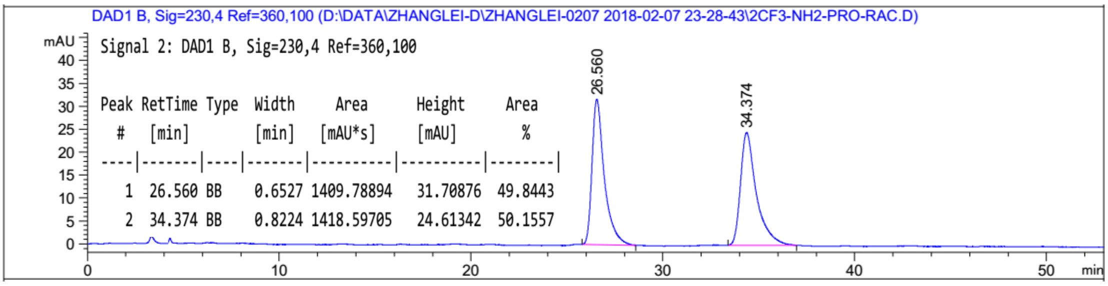


*Chiral HPLC spectrum of (R)-**3m***

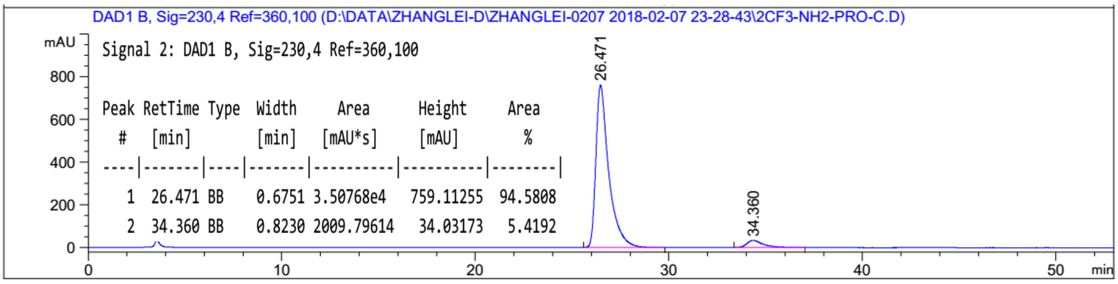


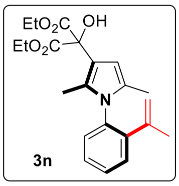


**(R)-Diethyl 2-(2,5-dimethyl-1-(2-(prop-1-en-2-yl)phenyl)-1*H*-pyrrol-3-yl)-2-hydroxymalonate ((*R*)-3n)**

According to the general procedure, *(R)*-**3n** was obtained in **96% yield** with **90% ee**.

**$^{1}$H NMR (400 MHz, CDCl$_3$)** δ 7.42-7.30 (m, 3H), 7.11 (dd, *J* = 7.3, 1.4 Hz, 1H), 5.96 (s, 1H), 4.99 (d, *J* = 1.3 Hz, 1H), 4.87 (s, 1H), 4.39-4.22 (m, 4H), 4.06 (s, 1H), 1.90 (s, 3H), 1.89 (s, 3H), 1.51 (s, 3H), 1.33-1.27 (m, 6H).

**$^{13}$C NMR (125 MHz, CDCl$_3$)** δ = 171.0, 171.0, 144.0, 142.3, 135.4, 129.7, 129.6, 128.6, 128.0, 127.4, 127.3, 116.5, 115.3, 105.8, 78.1, 62.5, 62.5, 21.3, 14.1, 14.0, 12.7, 11.8.

**HRMS (ESI)** calcd for $[M+H]^+$ $C_{22}H_{28}NO_5^+$, m/z: 386.1962, found: 386.1959.

**HPLC analysis**: DAICEL CHIRALPAK AD-3, hexane/isopropyl alcohol = 99/01, flow rate = 0.8 mL/min, λ = 230 nm), $t_R$ (major) = 37.6 min, $t_R$ (minor) = 40.6 min, ee = 90%.

*Chiral HPLC spectrum of racemic **3n***

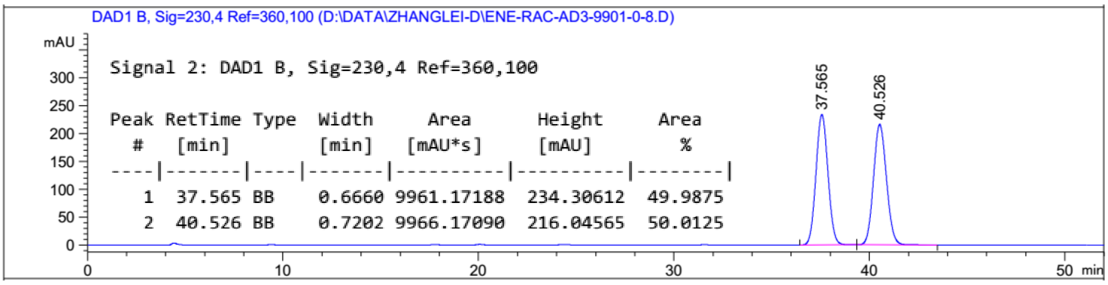


*Chiral HPLC spectrum of (R)-**3n***

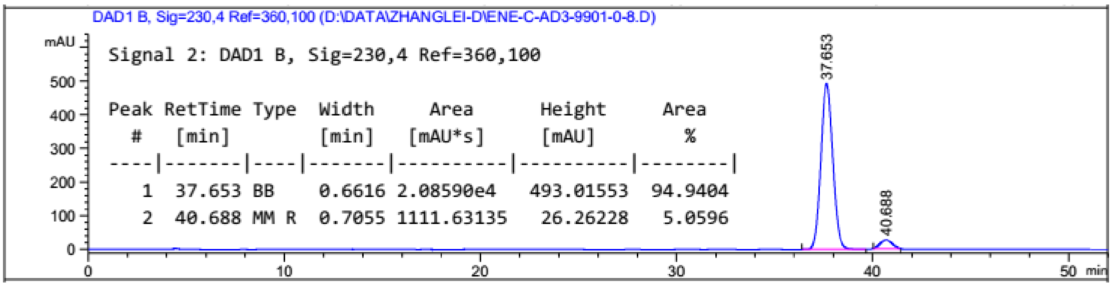


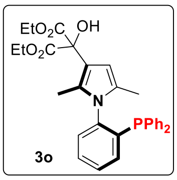


**(R)-Diethyl 2-(1-(2-(diphenylphosphanyl)phenyl)-2,5-dimethyl-1*H*-pyrrol-3-yl)-2-hydroxymalonate ((R)-3o)**

According to the general procedure, *(R)*-**3o** was obtained in **91% yield** with **89% ee**.

**$^1$H NMR (400 MHz, $CDCl_3$)** δ 7.45-7.41 (m, 1H), 7.39-7.35 (m, 1H), 7.31-7.28 (m, 6H), 7.26-7.15 (m, 6H), 5.95 (s, 1H), 4.40-4.23 (m, 4H), 3.89 (s, 1H), 1.74 (s, 3H), 1.64 (s, 3H), 1.34-1.27 (m, 6H).

**$^{13}$C NMR (100 MHz, $CDCl_3$)** δ = 170.87, 170.54, 142.8 (d, *J* = 25.9 Hz), 139.4 (d, *J* = 16.2 Hz), 136.2 (d, *J* = 12.1 Hz), 135.8 (d, *J* = 12.4 Hz), 134.6 (d, *J* = 1.7 Hz), 134.2, 134.0, 134.0, 133.8, 130.0, 129.6 (d, *J* = 2.9 Hz), 128.9, 128.8, 128.7, 128.5, 128.4 (d, *J* = 1.2 Hz), 128.4, 127.5, 127.3, 115.0, 105.8, 78.3, 62.5, 62.4, 14.2, 14.1, 12.8 (d, *J* = 5.0 Hz), 11.9 (d, *J* = 4.4 Hz).

**$^{31}$P NMR (162 MHz, $CDCl_3$)** δ = -16.64.

**HRMS (ESI)** calcd for $[M+H]^+$ $C_{31}H_{33}NO_5P^+$, m/z: 530.2091, found: 530.2089.

**HPLC analysis**: DAICEL CHIRALPAK IF, hexane/isopropyl alcohol = 90/10, flow rate = 0.8 mL/min, λ = 254 nm), $t_R$ (minor) = 28.0 min, $t_R$ (major) = 29.7 min, ee = 89%.

*Chiral HPLC spectrum of racemic **3o***

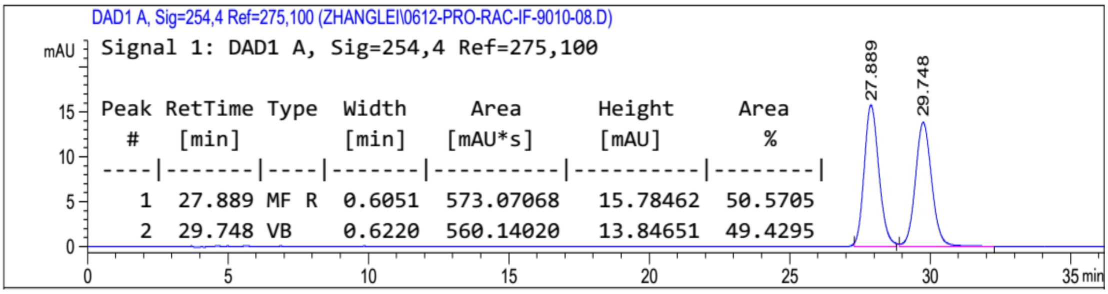


*Chiral HPLC spectrum of (R)-**3o***

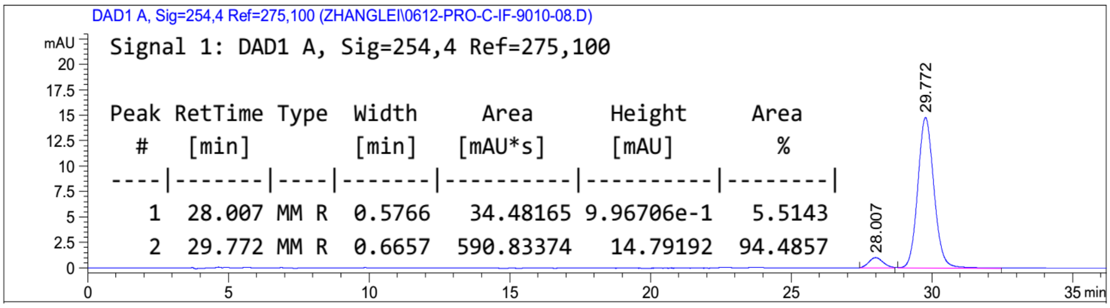

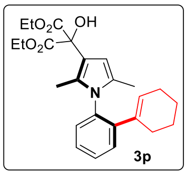


**(*R*)-Diethyl 2-(2,5-dimethyl-1-(2',3',4',5'-tetrahydro-[1,1'-biphenyl]-2-yl)-1*H*-pyrrol-3-yl)-2-hydroxymalonate ((*R*)-3p)**

According to the general procedure, *(R)*-**3p** was obtained in **95% yield** with **86% ee**.

**$^{1}$H NMR (400 MHz, $CDCl_3$)** δ 7.40-7.29 (m, 3H), 7.12 (d, *J* = 7.4 Hz, 1H), 5.96 (s, 1H), 5.59-5.57 (m, 1H), 4.43-4.23 (m, 4H), 4.03 (s, 1H), 2.07-2.06 (m, 2H), 1.92 (s, 6H), 1.65 (s, 2H), 1.54-1.52 (m, 4H), 1.36-1.31 (m, 6H).

**$^{13}$C NMR (100 MHz, $CDCl_3$)** δ = 171.0, 170.7, 143.5, 137.0, 135.3, 129.5, 129.5, 128.4, 127.9, 127.4, 127.2, 127.1, 115.0, 105.8, 78.2, 62.5, 62.4, 27.0, 25.9, 23.0, 21.8, 14.1, 12.7, 11.9.

**$^{13}$C NMR-DEPT 135 (100 MHz, $CDCl_3$)** δ = 129.52, 129.45, 128.42, 127.89, 127.13, 105.76, 62.50, 62.44, 27.03, 25.90, 23.00, 21.83, 14.07, 12.73, 11.92.

**HRMS (ESI)** calcd for $[M+H]^+$ $C_{25}H_{32}NO_5^+$, m/z: 426.2275, found: 426.2275.

**HPLC analysis**: DAICEL CHIRALCEL OD-3, hexane/isopropyl alcohol = 98/02, flow rate = 1.0 mL/min, λ = 254 nm), $t_R$ (minor) = 8.9 min, $t_R$ (major) = 12.3 min, ee = 86%.

*Chiral HPLC spectrum of racemic **3p***

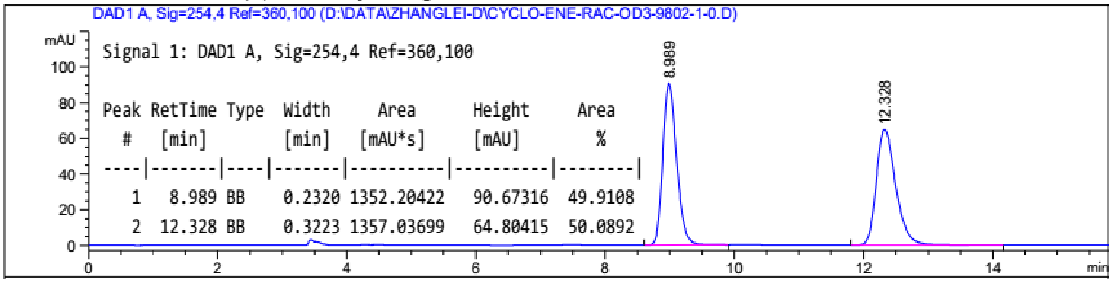


*Chiral HPLC spectrum of (R)-**3p***

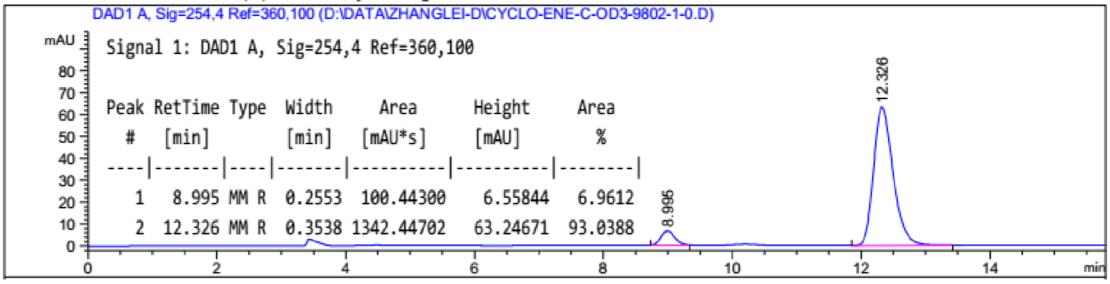


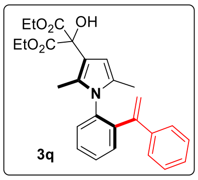


**(*R*)-Diethyl**

**2-(2,5-dimethyl-1-(2-(1-phenylvinyl)phenyl)-1*H*-pyrrol-3-yl)-2-hydroxymalonate(*(R)*-3q)**

According to the general procedure, *(R)*-**3q** was obtained in **86% yield** with **92% ee**.

**$^1$H NMR (400 MHz, CDCl$_3$)** δ 7.48-7.37 (m, 3H), 7.21-7.10 (m, 4H), 7.00 (dd, *J* = 7.8, 1.6 Hz, 2H), 5.66 (s, 1H), 5.34 (d, *J* = 1.0 Hz, 1H), 5.08 (d, *J* = 1.0 Hz, 1H), 4.51-4.14 (m, 4H), 3.48 (s, 1H), 1.77 (s, 3H), 1.74 (s, 3H), 1.33-1.26 (m, 6H).

**$^{13}$C NMR (100 MHz, CDCl$_3$)** δ = 170.7, 170.2, 147.9, 141.2, 141.1, 136.6, 131.2, 129.9, 128.5, 128.4, 127.7, 127.4, 127.1, 127.0, 117.6, 115.2, 105.8, 78.2, 62.4, 62.3, 14.1, 14.1, 12.6, 11.9.

**HRMS (ESI)** calcd for $[M+Na]^+$ $C_{27}H_{29}NNaO_5^+$, m/z: 470.1938, found: 470.1937.

**HPLC analysis**: DAICEL CHIRALCEL OD-H, hexane/isopropyl alcohol = 98/02, flow rate = 0.8 mL/min, λ = 240 nm), $t_R$ (major) = 22.2 min, $t_R$ (minor) = 25.3min, ee = 92%.

*Chiral HPLC spectrum of racemic* ***3q***

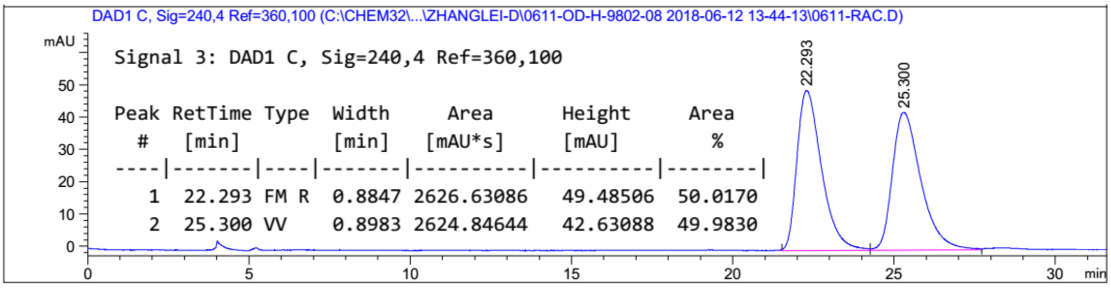


*Chiral HPLC spectrum of (R)-**3q***

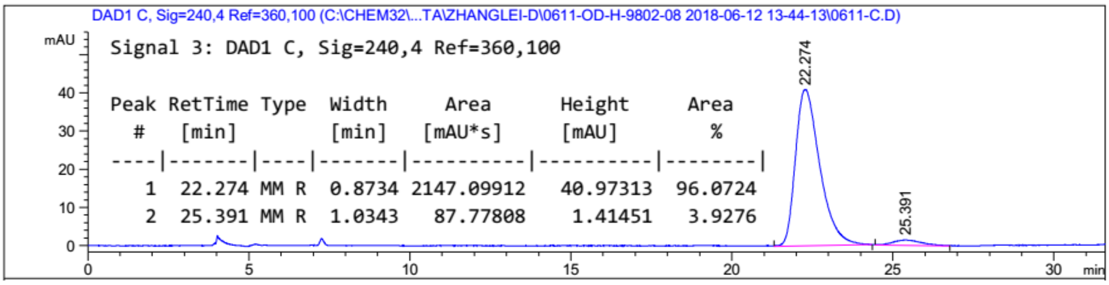


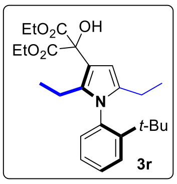


**(*R*)-Diethyl 2-(1-(2-(*tert*-butyl)phenyl)-2,5-diethyl-1*H*-pyrrol-3-yl)-2-hydroxymalonate(*(R)*-3r)**

According to the general procedure, *(R)*-**3r** was obtained in **95% yield** with **83% ee**.

**$^1$H NMR (400 MHz, CDCl$_3$)** δ 7.60-7.57 (m, 1H), 7.43-7.31 (m, 1H), 7.25-7.15 (m, 1H), 7.03-7.01 (m, 1H), 5.93 (s, 1H), 4.46-4.20 (m, 4H), 4.05 (s, 1H), 2.60-2.51 (m, 1H), 2.17-2.01 (m, 3H), 1.34 (t, *J* = 7.1 Hz, 3H), 1.28 (t, *J* = 7.1 Hz, 3H), 1.19-1.00 (m, 12H), 0.89 (t, *J* = 7.4 Hz, 3H).

**$^{13}$C NMR (100 MHz, CDCl$_3$)** δ = 171.3, 170.9, 147.5, 135.0, 134.4, 134.3, 132.1, 129.8, 128.7, 126.1, 114.4, 104.0, 78.5, 62.5, 36.2, 31.6, 20.5, 19.7, 15.0, 14.1, 14.0, 12.4.

**HRMS (ESI)** calcd for $[M+H]^+$ $C_{25}H_{36}NO_5^+$, m/z: 430.2588, found: 430.2586.

**HPLC analysis**: DAICEL CHIRALPAK AD-3, hexane/isopropyl alcohol = 95/05, flow rate = 1.0 mL/min, λ = 254 nm), $t_R$ (minor) = 8.5 min, $t_R$ (major) = 11.7 min, ee = 83%.

*Chiral HPLC spectrum of racemic* ***3r***

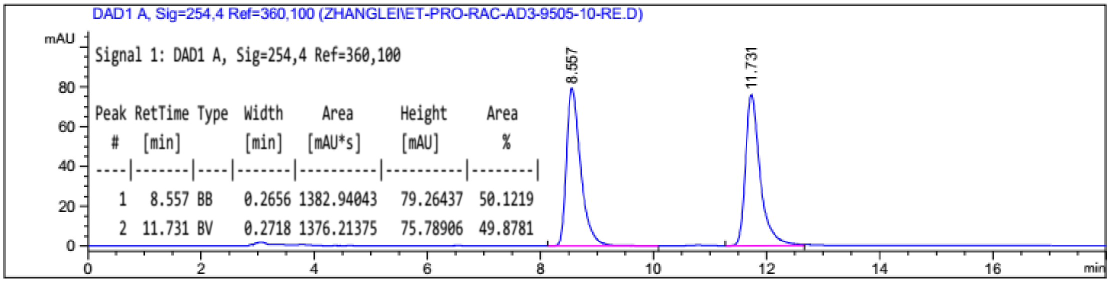


*Chiral HPLC spectrum of (R)-****3r***

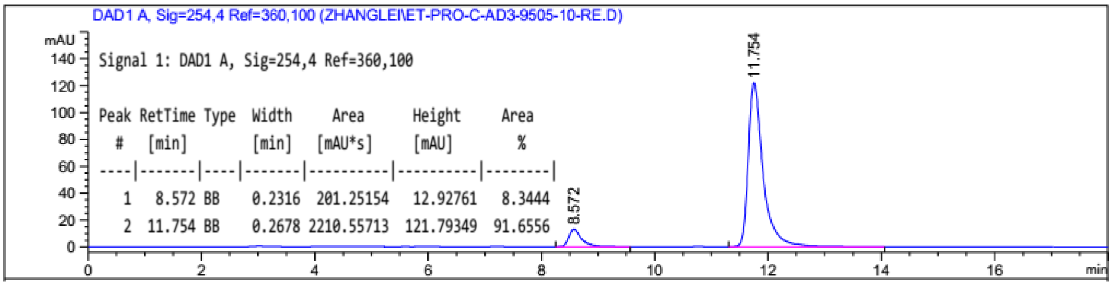


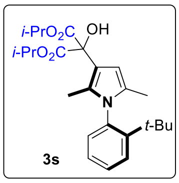


***(R)*****-diisopropyl**

**2-(1-(2-(tert-butyl)phenyl)-2,5-dimethyl-1H-pyrrol-3-yl)-2-hydroxymalonate(*(R)*-3s)**

According to the general procedure, *(R)***-3s** was obtained in **95% yield** with **96% ee**.

**$^{1}$H NMR (400 MHz, CDCl$_3$)** δ 7.59 (d, $J$ = 8.0 Hz, 1H), 7.39-7.35 (m, 1H), 7.23-7.19 (m, 1H), 6.91 (d, $J$ = 7.6 Hz, 1H), 5.97 (s, 1H), 5.19-5.10 (m, 2H), 4.05 (s, 1H), 1.90 (s, 6H), 1.45-1.20 (m, 12H), 1.14 (s, 9H).

**$^{13}$C NMR (100 MHz, CDCl$_3$)** δ = 170.6, 170.3, 147.7, 135.6, 131.5, 129.7, 128.6, 128.0, 127.9, 126.6, 115.0, 105.6, 78.1, 70.3, 36.1, 31.8, 21.7, 21.6, 21.6, 13.2, 12.6.

**HRMS (ESI)** calcd for $[M+H]^+$ $C_{25}H_{36}NO_5^+$, m/z: 430.2588, found: 430.2587.

**HPLC analysis**: DAICEL CHIRALCEL OD-3, hexane/isopropyl alcohol = 98/02, flow rate = 0.8 mL/min, λ = 214 nm), $t_R$ (minor) = 12.6 min, $t_R$ (major) = 13.9 min, ee = 96%.

*Chiral HPLC spectrum of racemic* ***3s***

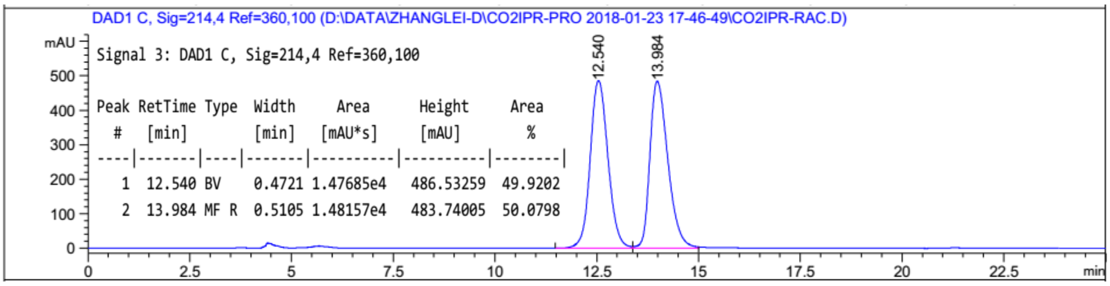


*Chiral HPLC spectrum of (R)-****3s***

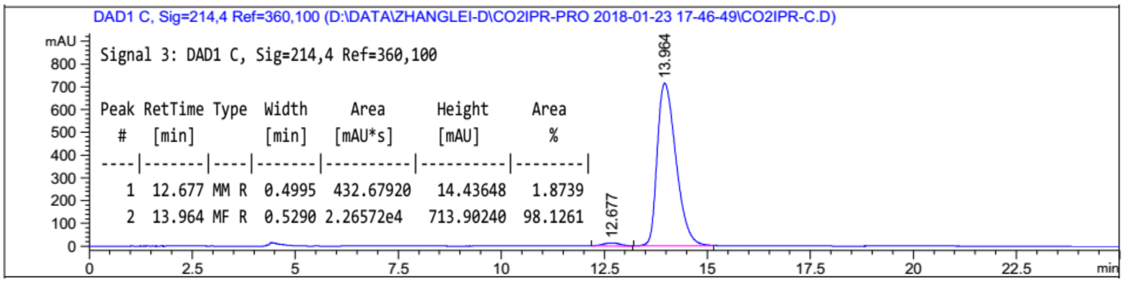


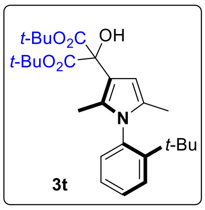


**(R)-Di-*tert*-butyl 2-(1-(2-(*tert*-butyl)phenyl)-2,5-dimethyl-1*H*-pyrrol-3-yl)-2-hydroxymalonate(*(R)*-3t)**

According to the general procedure, *(R)*-**3t** was obtained in **88% yield** with **94% ee**.

**$^{1}$H NMR (400 MHz, CDCl$_3$)** δ 7.58 (dd, *J* = 8.1, 1.3 Hz, 1H), 7.41-7.31 (m, 1H), 7.21 (td, *J* = 7.6, 1.4 Hz, 1H), 6.92 (dd, *J* = 7.7, 1.4 Hz, 1H), 6.00 (d, *J* = 0.5 Hz, 1H), 4.06 (s, 1H), 1.92 (s, 3H), 1.89 (s, 3H), 1.52 (s, 9H), 1.48 (s, 9H), 1.14 (s, 9H).

**$^{13}$C NMR (100 MHz, CDCl$_3$)** δ = 170.1, 169.9, 147.6, 135.7, 131.6, 129.7, 128.5, 127.8, 127.7, 126.6, 115.4, 105.8, 82.8, 82.6, 78.6, 36.1, 31.8, 27.9, 27.8, 13.2, 12.9.

**HRMS (ESI)** calcd for $[M+H]^+$ $C_{27}H_{40}NO_5^+$, m/z: 458.2901, found: 458.2900.

**HPLC analysis**: DAICEL CHIRALPAK AD-3, hexane/isopropyl alcohol = 98/02, flow rate = 1.0 mL/min, λ = 214 nm), $t_R$ (major) = 12.4 min, $t_R$ (minor) = 13.7 min, ee = 94%.

*Chiral HPLC spectrum of racemic **3t***

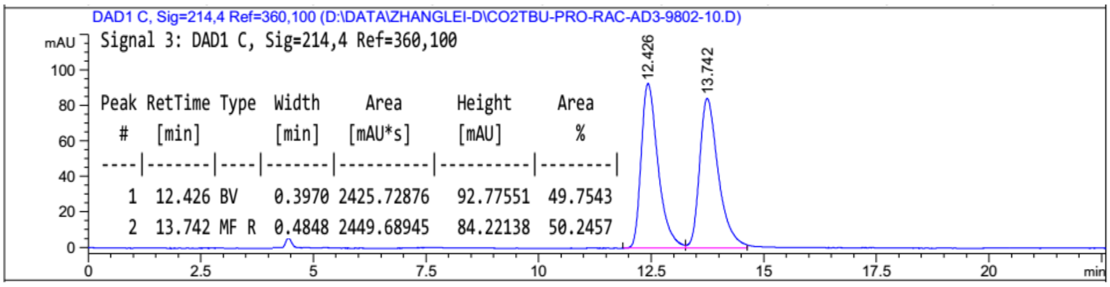


*Chiral HPLC spectrum of (R)-**3t***

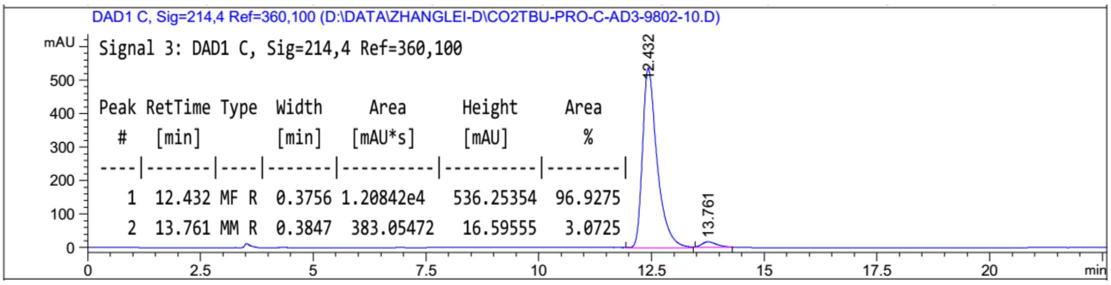

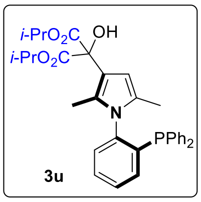


**(R)-Di-iso-propyl 2-(1-(2-(diphenylphosphanyl)phenyl)-2,5-dimethyl-1H-pyrrol-3-yl)-2-hydroxymalonate((R)-3u)**

According to the general procedure, *(R)*-**3u** was obtained in **80% yield** with **91% ee**.

**$^{1}$H NMR (400 MHz, CDCl$_3$)** δ 7.45 (t, *J* = 7.4 Hz, 1H), 7.38 (t, *J* = 7.4 Hz, 1H), 7.33-7.28 (m, 6H), 7.27-7.17 (m, 6H), 5.96 (s, 1H), 5.21-5.12 (m, 2H), 3.92 (s, 1H), 1.75 (s, 3H), 1.68 (s, 3H), 1.34-1.29 (m, 12H).

**$^{13}$C NMR (100 MHz, CDCl$_3$)** δ = 170.4, 170.1, 143.2, 142.9, 139.5, 139.3, 136.2, 136.1, 134.5, 134.2, 134.0, 134.0, 133.8, 130.0, 129.6, 128.8, 128.7, 128.7, 128.5, 128.4, 128.3, 127.4, 127.2, 115.1, 105.9, 78.2, 70.3, 70.2, 21.7, 21.6, 12.8, 12.7, 12.2, 12.2.

**$^{31}$P NMR (162 MHz, CDCl$_3$)** δ = -16.81.

**HRMS (ESI)** calcd for $[M+H]^+$ $C_{33}H_{37}NO_5P^+$, m/z: 558.2404, found: 558.2408.

**HPLC analysis**: DAICEL CHIRALPAK IC, hexane/isopropyl alcohol = 80/20, flow rate = 1.0 mL/min, λ = 254 nm), $t_R$ (major) = 16.3 min, $t_R$ (minor) = 32.2 min, ee = 91%.

*Chiral HPLC spectrum of racemic **3u***

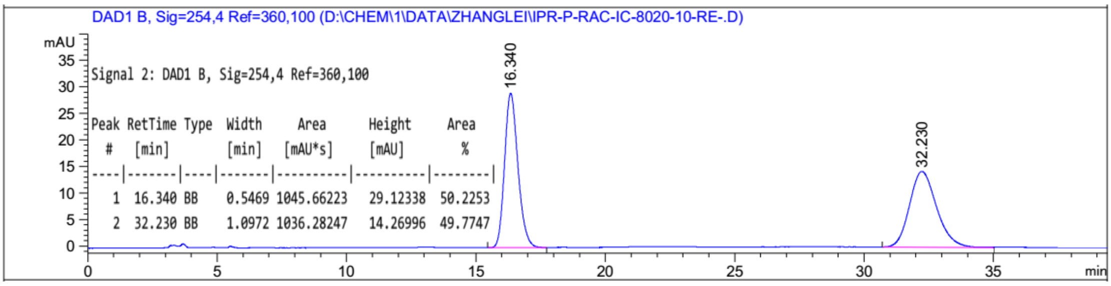


*Chiral HPLC spectrum of (R)-**3u***

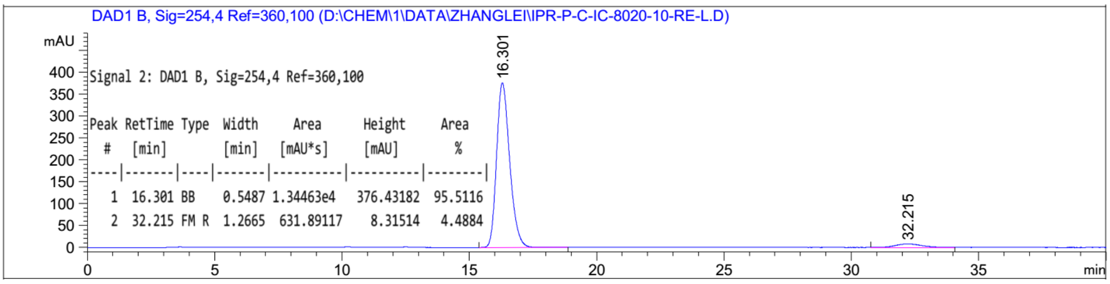

# Supplementary Note 4

## General procedure for preparation of racemic compound 5

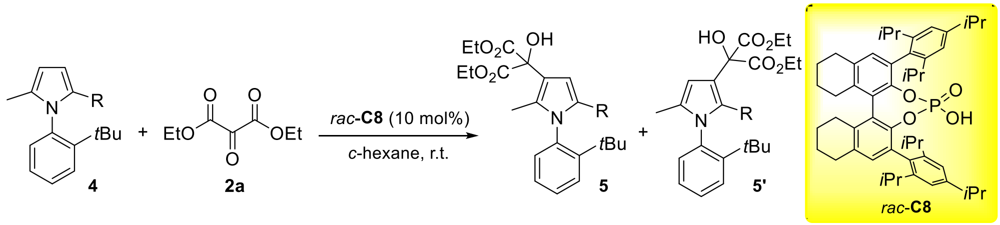


An oven-dried 10 mL of Schlenk tube was charged with arylpyrrole **4** (0.20 mmol), 1 mL cyclohexane and *rac*-**C8** (0.01 mmol) at ambient temperature. Then, ketomalonate **2a** (0.10 mmol) was added to the above solution and the mixture was stirred until the starting material was completely consumed. The mixture was concentrated under reduced pressure and purified by flash column chromatography (ethyl acetate/petroleum ether) to afford the corresponding racemic product **5**. Notably, there was also by-product **5'** was obtained, which is the isomer of **5**.

## General procedure for the kinetic resolution of racemic arylpyrroles (*rac*-4)

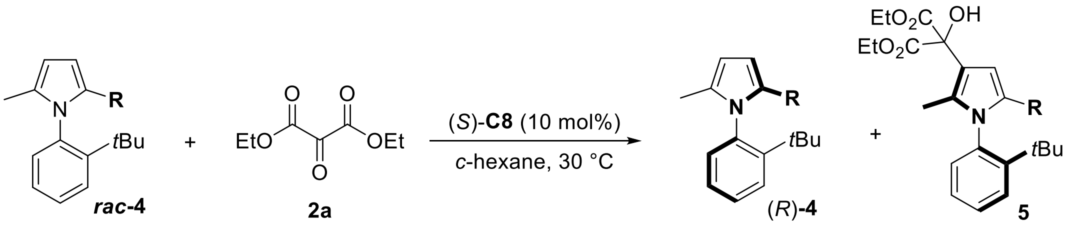


Under nitrogen atmosphere, an oven-dried 10 mL of Schlenk tube was charged with asymmetric arylpyrroles *rac*-**4** (0.40 mmol), (*S*)-**C8** (0.02 mmol), 2.4 mL of dry cyclohexane, and the mixture was stirred at 30 ℃ for 10 min. Then, a solution of ketomalonate **2a** (0.20 mmol) in dry cyclohexane (2.4 mL) was added dropwise to the above solution and the mixture was stirred until the starting material was completely consumed, then the mixture was concentrated under reduced pressure and purified by flash chromatography eluted with PE/EA (10/1 to 5/1) to afford the corresponding axially chiral arylpyrroles product **5** and recovered substrates (*R*)-**4**.

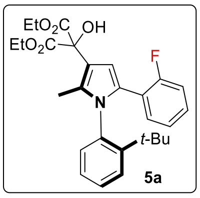


**(*S*)-Diethyl 2-(1-(2-(*tert*-butyl)phenyl)-5-(2-fluorophenyl)-2-methyl-1*H*-pyrrol-3-yl)-2-hydroxymalonate ((*S*)-5a)**

According to the general procedure, *(S)*-**5a** was obtained in **43% yield** with **91% ee**.

**$^1$H NMR (400 MHz, $CDCl_3$)** δ 7.47 (dd, *J* = 8.1, 1.3 Hz, 1H), 7.39-7.30 (m, 1H), 7.26-7.15 (m, 2H), 7.08-6.91 (m, 2H), 6.86-6.69 (m, 2H), 6.54 (d, *J* = 3.0 Hz, 1H), 4.43-4.26 (m, 4H), 4.14 (s, 1H), 2.00 (s, 3H), 1.36-1.30 (m, 6H), 0.93 (s, 9H).

**$^{13}$C NMR (100 MHz, CDCl$_3$)** δ = 170.8, 170.5, 159.6 (d, *J* = 246.7 Hz), 147.5, 135.4, 132.5, 131.0, 130.2 (d, *J* = 2.4 Hz), 130.0, 128.8, 127.6 (d, *J* = 8.3 Hz), 126.1, 125.9, 123.2 (d, *J* = 3.6 Hz), 121.4 (d, *J* = 12.8 Hz), 116.4, 115.7 (d, *J* = 23.1 Hz), 111.2 (d, *J* = 6.7 Hz), 78.1, 62.7, 62.7, 36.0, 31.2, 14.1, 14.1, 12.6.
**$^{19}$F NMR (376 MHz, CDCl$_3$)** δ = -112.34.
**HRMS (ESI)** calcd for $[M+H]^+$ $C_{28}H_{33}FNO_5^+$, m/z: 482.2337, found: 482.2337.
**HPLC analysis**: DAICEL CHIRALPAK ID, hexane/isopropyl alcohol = 90/10, flow rate = 1.0 mL/min, λ = 270 nm), $t_R$ (major) = 17.2 min, $t_R$ (minor) = 23.5 min, ee = 91%.

*Chiral HPLC spectrum of racemic **5a***

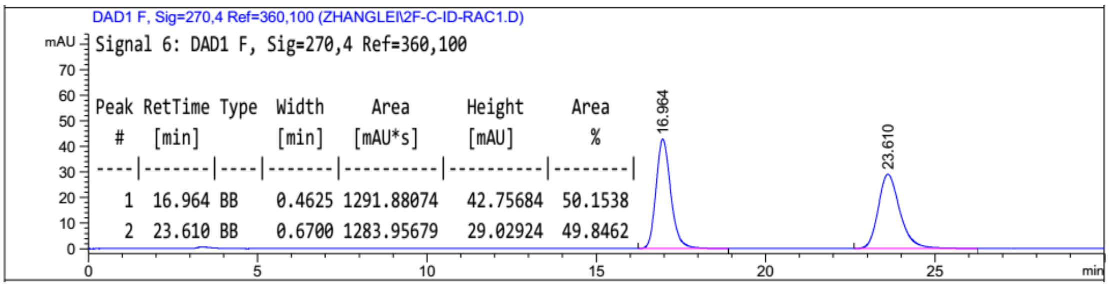


*Chiral HPLC spectrum of (S)-**5a***

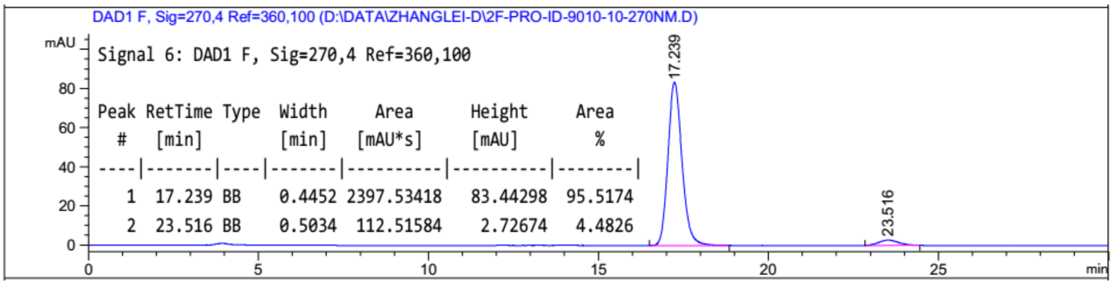


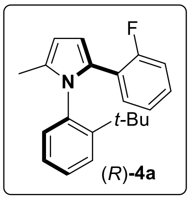


**(*R*)-1-(2-(*tert*-butyl)phenyl)-2-(2-fluorophenyl)-5-methyl-1*H*-pyrrole ((*R*)-4a)**
According to the general procedure, *(R)*-**4a** was obtained in **52% yield** with **72% ee**.
**HPLC analysis**: DAICEL CHIRALPAK AD-3, hexane/isopropyl alcohol = 98/02, flow rate = 0.5 mL/min, λ = 300 nm), $t_R$ (major) = 6.6 min, $t_R$ (minor) = 7.1 min, ee = 72%.

*Chiral HPLC spectrum of racemic **4a***

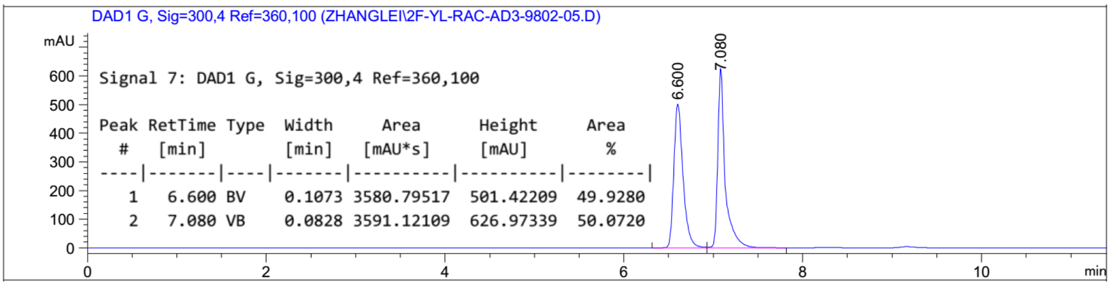

*Chiral HPLC spectrum of (R)-**4a***

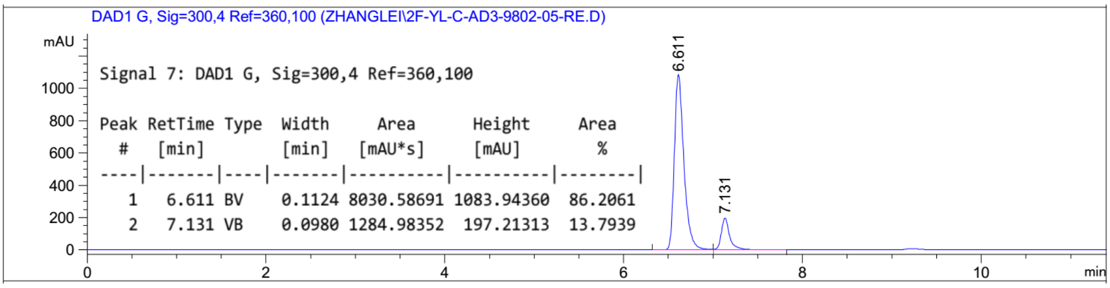


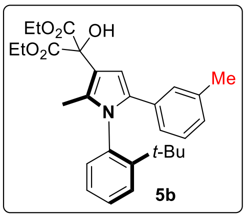


**(*S*)-Diethyl 2-(1-(2-(*tert*-butyl)phenyl)-2-methyl-5-(*m*-tolyl)-1*H*-pyrrol-3-yl)-2-hydroxymalonate ((*S*)-5b)**

According to the general procedure, *(S)*-**5b** was obtained in **45% yield** with **89% ee**.

**$^1$H NMR (400 MHz, $CDCl_3$)** δ 7.52 (dd, *J* = 8.1, 1.2 Hz, 1H), 7.40-7.36 (m, 1H), 7.27-7.23 (m, 1H), 7.16 (dd, *J* = 7.8, 1.4 Hz, 1H), 6.97-6.93 (m, 1H), 6.89 (s, 1H), 6.84 (d, *J* = 7.5 Hz, 1H), 6.79 (d, *J* = 7.8 Hz, 1H), 6.48 (s, 1H), 4.42-4.30 (m, 4H), 4.15 (s, 1H), 2.14 (s, 3H), 1.98 (s, 3H), 1.36-1.30 (m, 6H), 0.95 (s, 9H).

**$^{13}$C NMR (100 MHz, $CDCl_3$)** δ = 170.9, 170.6, 147.5, 137.4, 136.0, 133.3, 133.2, 132.2, 130.8, 130.1, 128.8, 127.9, 127.8, 126.4, 126.4, 124.0, 116.4, 107.7, 78.1, 62.7, 62.7, 36.0, 31.3, 21.4, 14.2, 14.1, 12.6.

**HRMS (ESI)** calcd for $[M+H]^+$ $C_{29}H_{36}NO_5^+$, m/z: 478.2588, found: 478.2590.

**HPLC analysis**: DAICEL CHIRALCEL OD-3, hexane/isopropyl alcohol = 95/05, flow rate = 1.0 mL/min, λ = 300 nm), $t_R$ (minor) = 11.0 min, $t_R$ (major) = 14.9min, ee = 89%.

*Chiral HPLC spectrum of racemic **5b***

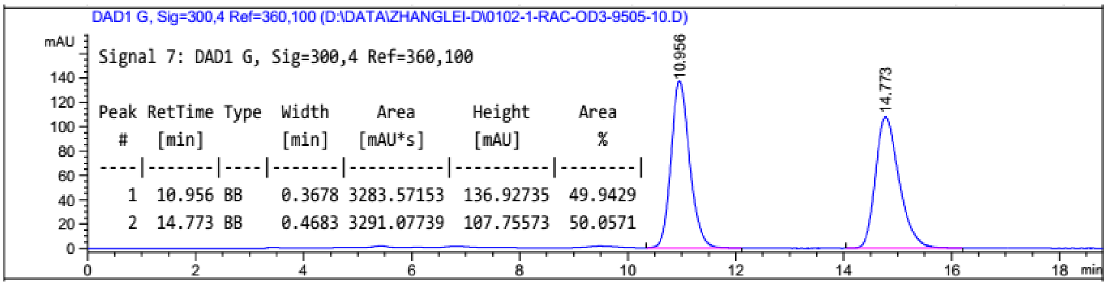


*Chiral HPLC spectrum of (S)-**5b***

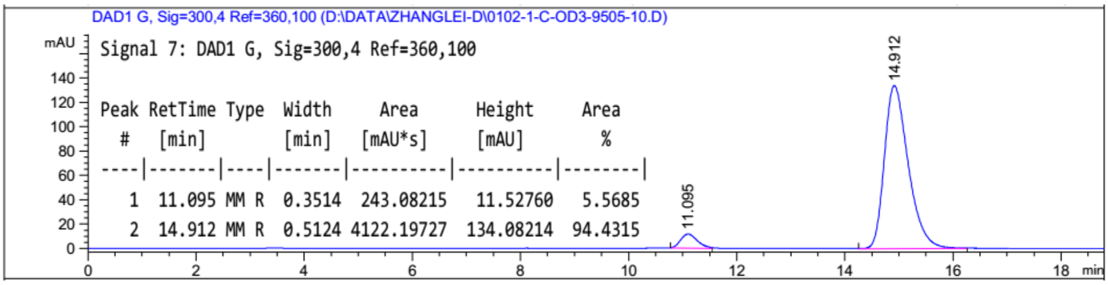


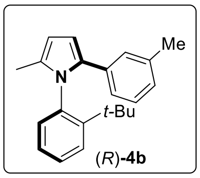


**(*R*)-1-(2-(*tert*-butyl)phenyl)-2-methyl-5-(m-tolyl)-1*H*-pyrrole ((*R*)-4b)**

According to the general procedure, *(R)*-**4b** was obtained in **51% yield** with **80% ee**.

**HPLC analysis**: DAICEL CHIRALPAK AD-3, hexane/isopropyl alcohol = 98/02, flow rate = 0.5 mL/min, $\lambda$ = 210 nm), $t_R$ (major) = 7.4 min, $t_R$ (minor) = 7.9 min, ee = 80%.

*Chiral HPLC spectrum of racemic* ***4b***

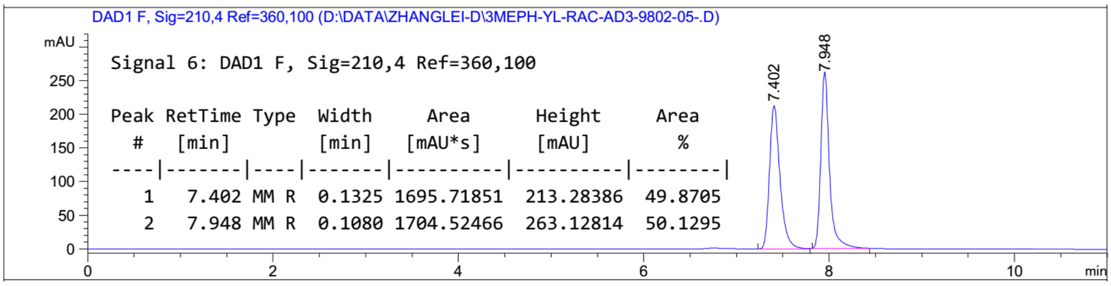


*Chiral HPLC spectrum of (R)-**4b***

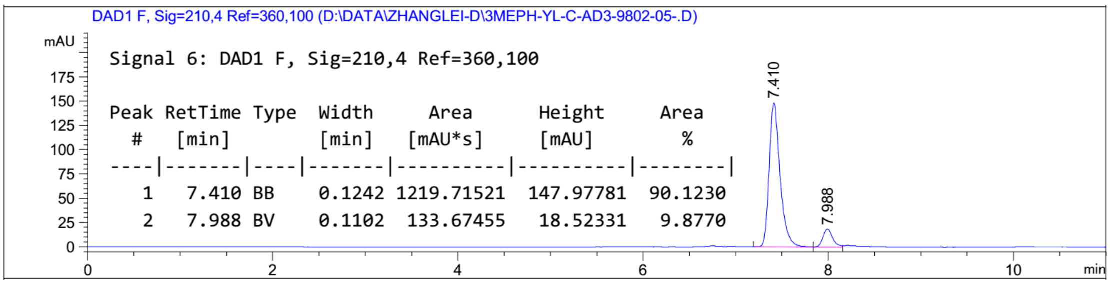


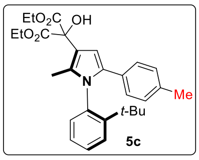


**(*S*)-Diethyl 2-(1-(2-(*tert*-butyl)phenyl)-2-methyl-5-(*p*-tolyl)-1*H*-pyrrol-3-yl)-2-hydroxymalonate ((*S*)-5c)**

According to the general procedure, *(S)*-**5c** was obtained in **44% yield** with **90% ee**.

**$^{1}$H NMR (400 MHz, $CDCl_3$)** δ 7.52 (dd, *J* = 8.1, 1.3 Hz, 1H), 7.40-7.36 (m, 1H), 7.28-7.23 (m, 1H), 7.17 (dd, *J* = 7.8, 1.5 Hz, 1H), 6.94-6.88 (m, 4H), 6.44 (s, 1H), 4.42-4.29 (m, 4H), 4.15 (s, 1H), 2.21 (s, 3H), 1.97 (s, 3H), 1.36-1.30 (m, 6H), 0.95 (s, 9H).

**$^{13}$C NMR (100 MHz, $CDCl_3$)** δ = 170.9, 170.6, 147.5, 136.0, 135.3, 133.3, 132.3, 130.7, 130.5, 130.1, 128.8, 128.7, 127.0, 126.4, 116.3, 107.4, 78.1, 62.7, 62.7, 36.0, 31.3, 21.0, 14.2, 14.1, 12.6.

**HRMS (ESI)** calcd for $[M+H]^+$ $C_{29}H_{36}NO_5^+$, m/z: 478.2588, found: 478.2593.

**HPLC analysis**: DAICEL CHIRALCEL OD-3, hexane/isopropyl alcohol = 98/02, flow rate = 0.8 mL/min, λ = 300 nm), $t_R$ (major) = 23.0 min, $t_R$ (minor) = 25.4min, ee = 90%.

*Chiral HPLC spectrum of racemic **5c***

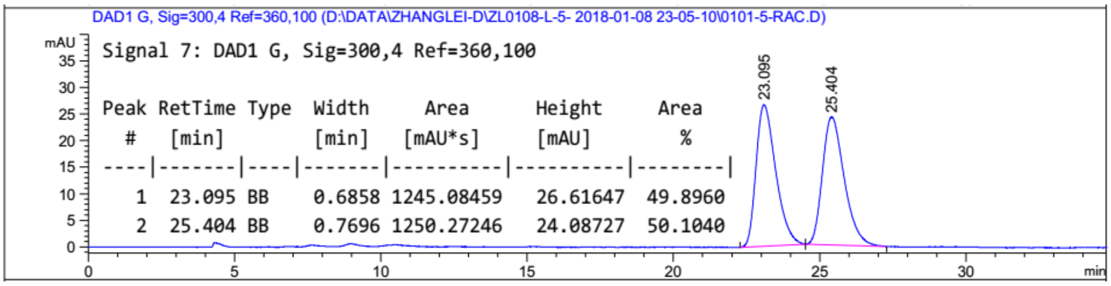


*Chiral HPLC spectrum of (S)-**5c***

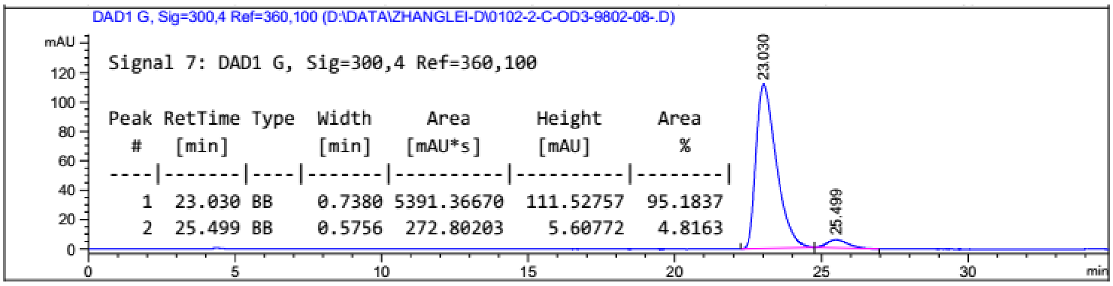


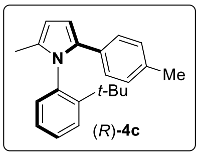


**(*R*)-1-(2-(*tert*-butyl)phenyl)-2-methyl-5-(p-tolyl)-1*H*-pyrrole ((*R*)-4c)**

According to the general procedure, *(R)*-**4c** was obtained in **52% yield** with **76% ee**.

**HPLC analysis**: DAICEL CHIRALPAK AD-3, hexane/isopropyl alcohol = 98/02, flow rate = 0.5 mL/min, λ = 300 nm), $t_R$ (major) = 7.9 min, $t_R$ (minor) = 8.2 min, ee = 76%.

*Chiral HPLC spectrum of racemic **4c***

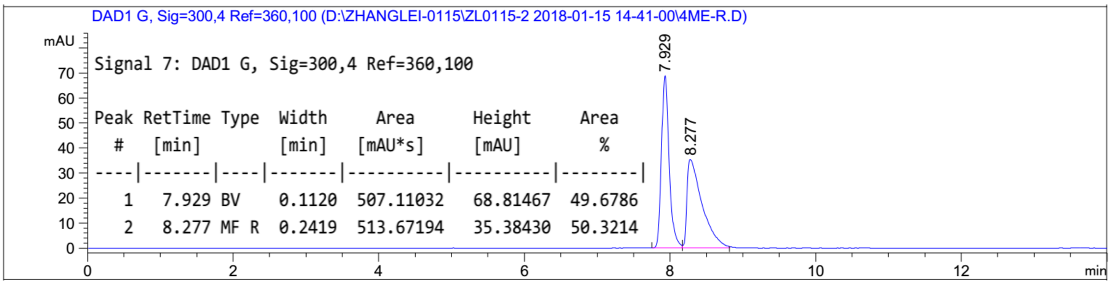


*Chiral HPLC spectrum of (R)-**4c***

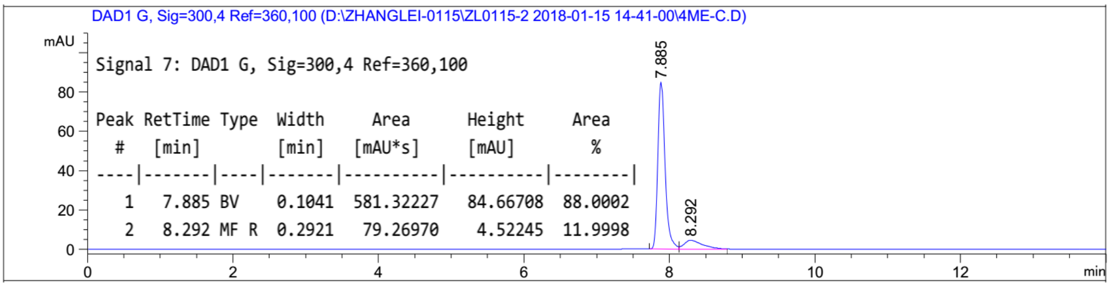


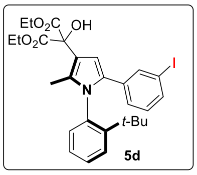


**(*S*)-Diethyl 2-(1-(2-(*tert*-butyl)phenyl)-5-(3-iodophenyl)-2-methyl-1*H*-pyrrol-3-yl)-2-hydroxymalonate ((*S*)-5d)**

According to the general procedure, *(S)*-**5d** was obtained in **46% yield** with **91% ee**.

**$^{1}$H NMR (400 MHz, CDCl$_3$)** δ 7.56 (dd, *J* = 8.1, 1.0 Hz, 1H), 7.44-7.38 (m, 2H), 7.35-7.26 (m, 2H), 7.12 (dd, *J* = 7.7, 1.2 Hz, 1H), 6.96 (d, *J* = 8.0 Hz, 1H), 6.81-6.77 (m, 1H), 6.53 (s, 1H), 4.40-4.30 (m, 4H), 4.07 (s, 1H), 1.99 (s, 3H), 1.36-1.30 (m, 6H), 0.96 (s, 9H).

**$^{13}$C NMR (100 MHz, CDCl$_3$)** δ = 170.7, 170.4, 147.4, 135.6, 135.5, 135.4, 134.4, 131.9, 131.7, 131.3, 130.3, 129.6, 129.1, 126.6, 125.7, 116.8, 108.6, 94.0, 77.9, 62.8, 62.7, 36.0, 31.4, 14.2, 14.1, 12.6.

**HRMS (ESI)** calcd for $[M+H]^+$ $C_{28}H_{33}INO_5^+$, m/z: 590.1398, found: 590.1399.

**HPLC analysis**: DAICEL CHIRALPAK AD-3, hexane/isopropyl alcohol = 98/02, flow rate = 1.0 mL/min, λ = 300 nm), $t_R$ (major) = 24.6 min, $t_R$ (minor) = 31.0 min, ee = 91%.

*Chiral HPLC spectrum of racemic **5d***

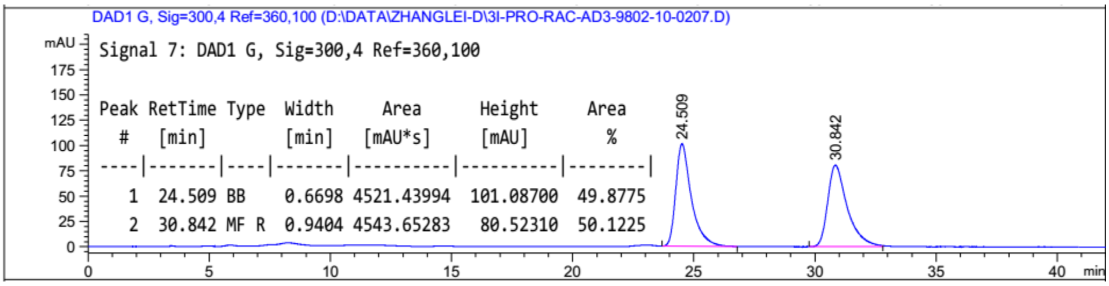


*Chiral HPLC spectrum of (S)-**5d***

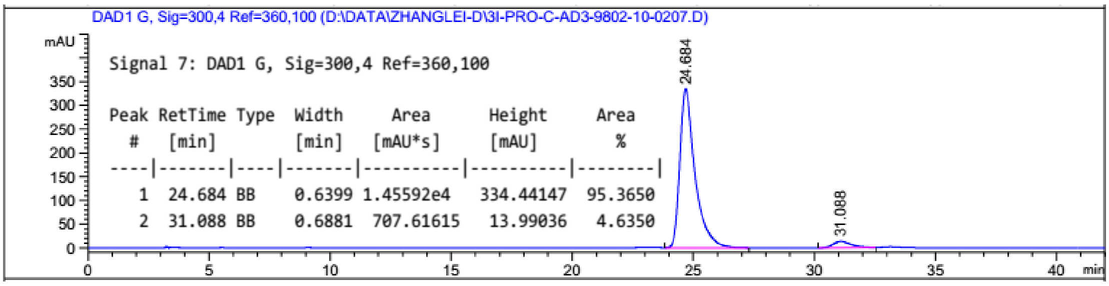

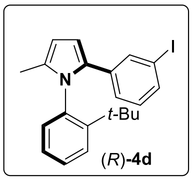


**(*R*)-1-(2-(*tert*-butyl)phenyl)-2-(3-iodophenyl)-5-methyl-1*H*-pyrrole ((*R*)-4d)**

According to the general procedure, *(R)*-**4d** was obtained in **49% yield** with **89% ee**.

**HPLC analysis**: DAICEL CHIRALPAK AD-3, hexane/isopropyl alcohol = 98/02, flow rate = 0.5 mL/min, $\lambda$ = 240 nm), $t_R$ (major) = 7.1 min, $t_R$ (minor) = 7.6 min, ee = 89%.

*Chiral HPLC spectrum of racemic* ***4d***

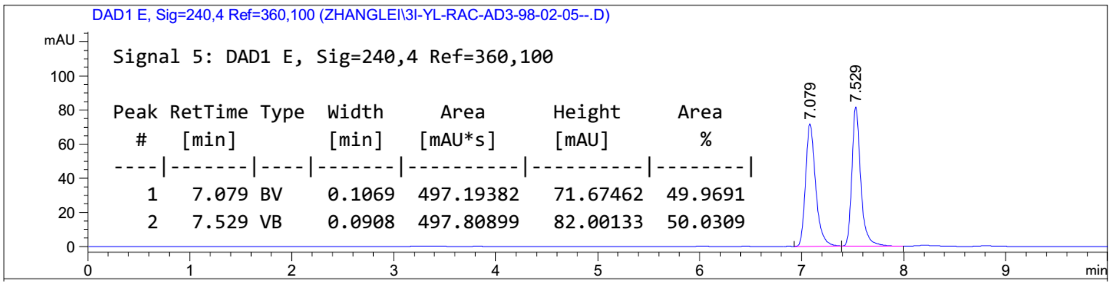


*Chiral HPLC spectrum of (R)-****4d***

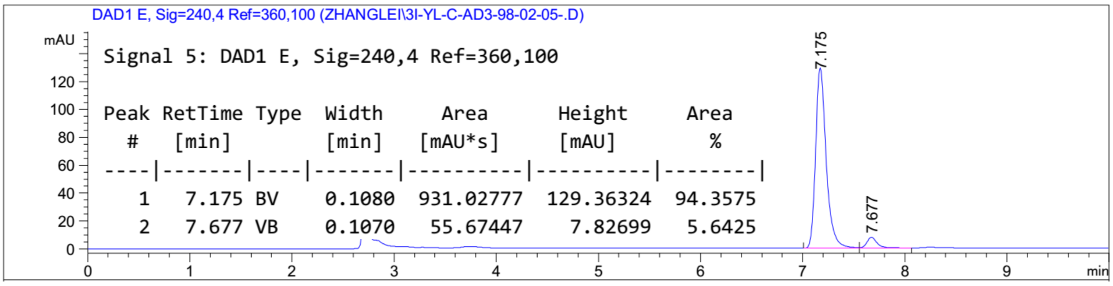


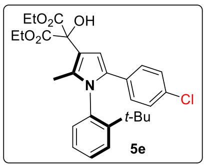


**(*S*)-Diethyl 2-(1-(2-(*tert*-butyl)phenyl)-5-(4-chlorophenyl)-2-methyl-1*H*-pyrrol-3-yl)-2-hydroxymalonate ((*S*)-5e)**

According to the general procedure, *(S)*-**5e** was obtained in **44% yield** with **91% ee**.

**$^1$H NMR (400 MHz, CDCl$_3$)** $\delta$ 7.53 (dd, *J* = 8.2, 1.4 Hz, 1H), 7.41-7.37 (m, 1H), 7.29-7.25 (m, 1H), 7.15 (dd, *J* = 7.8, 1.5 Hz, 1H), 7.06-7.02 (m, 2H), 6.95-6.92 (m, 2H), 6.49 (s, 1H), 4.39-4.29 (m, 4H), 4.17 (s, 1H), 1.97 (s, 3H), 1.35-1.29 (m, 6H), 0.93 (s, 9H).

**$^{13}$C NMR (100 MHz, CDCl$_3$)** $\delta$ = 170.8, 170.5, 147.5, 135.6, 132.1, 132.0, 131.9, 131.4, 131.4, 130.3, 129.1, 128.2, 128.1, 126.6, 116.7, 108.2, 78.0, 62.8, 62.7, 36.0, 31.3, 14.1, 14.1, 12.6.

**HRMS (ESI)** calcd for $[M+H]^+$ $C_{28}H_{33}ClNO_5^+$, m/z: 498.2042, found: 498.2042.

**HPLC analysis**: DAICEL CHIRALPAK ID, hexane/isopropyl alcohol = 90/10, flow rate = 1.0 mL/min, $\lambda$ = 320 nm), $t_R$ (major) = 15.5 min, $t_R$ (minor) = 18.5min, ee = 91%.

*Chiral HPLC spectrum of racemic* ***5e***

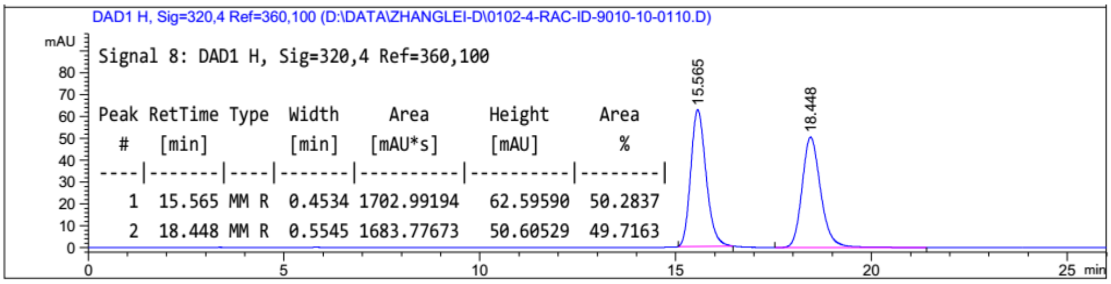


*Chiral HPLC spectrum of (S)-****5e***

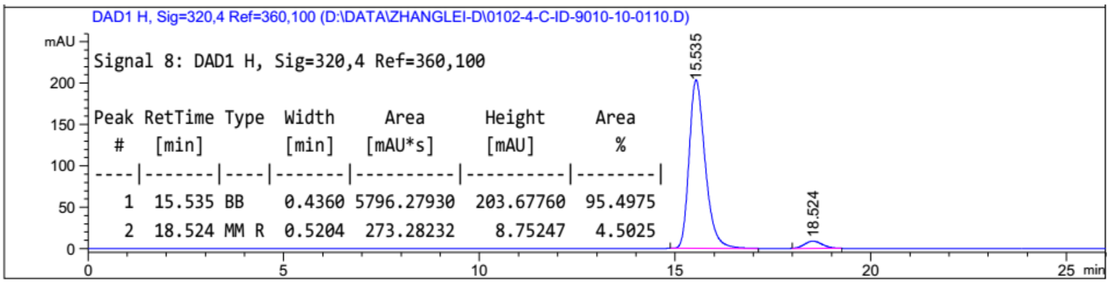


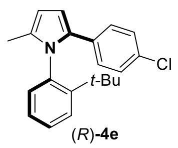


(*R*)-**4e**

**(*R*)-1-(2-(*tert*-butyl)phenyl)-2-(4-chlorophenyl)-5-methyl-1*H*-pyrrole ((*R*)-4e)**

According to the general procedure, *(R)*-**4e** was obtained in **51% yield** with **73% ee**.

**HPLC analysis**: DAICEL CHIRALPAK AD-3, hexane/isopropyl alcohol = 98/02, flow rate = 0.5 mL/min, λ = 300 nm), $t_R$ (major) = 7.9 min, $t_R$ (minor) = 8.3 min, ee = 73%.

*Chiral HPLC spectrum of racemic* ***4e***

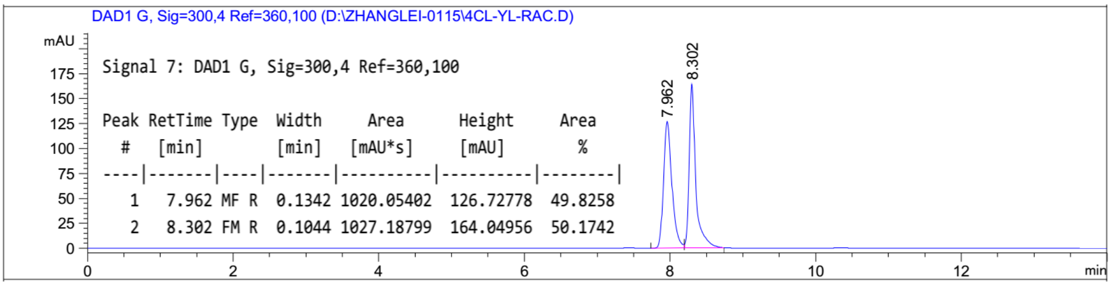


*Chiral HPLC spectrum of (R)-****4e***

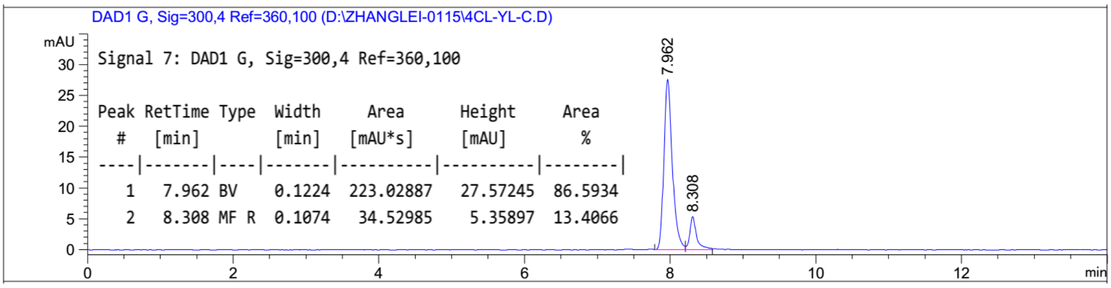

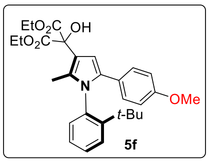


**(*S*)-Diethyl 2-(1-(2-(*tert*-butyl)phenyl)-5-(4-methoxyphenyl)-2-methyl-1*H*-pyrrol-3-yl)-2-hydroxymalonate ((*S*)-5f)**

According to the general procedure, *(S)*-**5f** was obtained in **45% yield** with **88% ee**.

**$^1$H NMR (400 MHz, $CDCl_3$)** δ 7.51 (dd, *J* = 8.1, 1.2 Hz, 1H), 7.40-7.35 (m, 1H), 7.28-7.24 (m, 1H), 7.18 (dd, *J* = 7.7, 1.4 Hz, 1H), 6.95 (d, *J* = 8.9 Hz, 2H), 6.63 (d, *J* = 8.9 Hz, 2H), 6.38 (s, 1H), 4.40-4.30 (m, 4H), 4.15 (s, 1H), 3.70 (s, 3H), 1.97 (s, 3H), 1.36-1.30 (m, 6H), 0.94 (s, 9H).

**$^{13}$C NMR (100 MHz, $CDCl_3$)** δ = 170.9, 170.6, 157.7, 147.6, 135.9, 133.1, 132.3, 130.1, 128.8, 128.5, 126.3, 116.3, 113.4, 106.9, 78.1, 62.7, 62.6, 55.1, 36.0, 31.3, 14.1, 14.1, 12.6.

**HRMS (ESI)** calcd for $[M+H]^+$ $C_{29}H_{36}NO_6^+$, m/z: 494.2537, found: 494.2542.

**HPLC analysis**: DAICEL CHIRALCEL OD-3, hexane/isopropyl alcohol = 95/05, flow rate = 1.0 mL/min, λ = 270 nm), $t_R$ (minor) = 17.9 min, $t_R$ (major) = 20.7 min, ee = 88%.

*Chiral HPLC spectrum of racemic **5f***

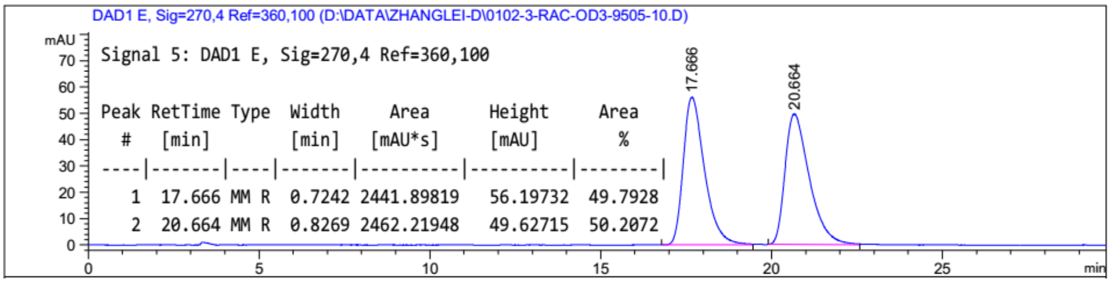


*Chiral HPLC spectrum of (S)-**5f***

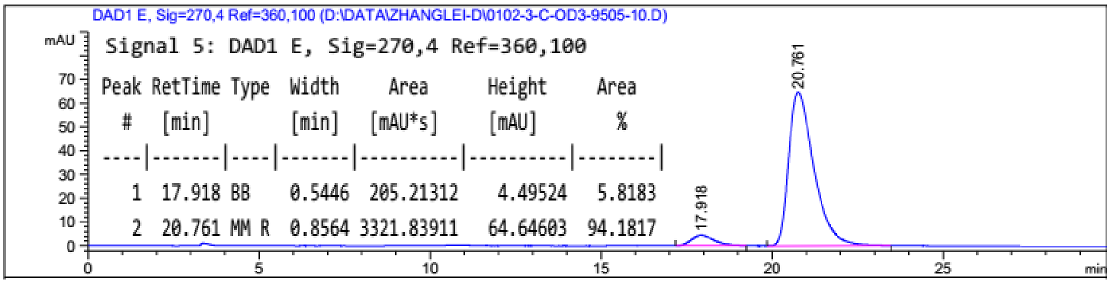


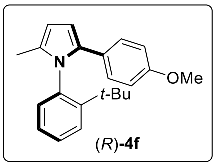


**(*R*)-1-(2-(*tert*-butyl)phenyl)-2-(4-methoxyphenyl)-5-methyl-1*H*-pyrrole ((*R*)-4f)**

According to the general procedure, *(R)*-**4f** was obtained in **52% yield** with **76% ee**.

**HPLC analysis**: DAICEL CHIRALPAK AD-3, hexane/isopropyl alcohol = 98/02, flow rate = 0.5 mL/min, λ = 230 nm), $t_R$ (major) = 9.2 min, $t_R$ (minor) = 11.2 min, ee = 76%.

*Chiral HPLC spectrum of racemic **4f***

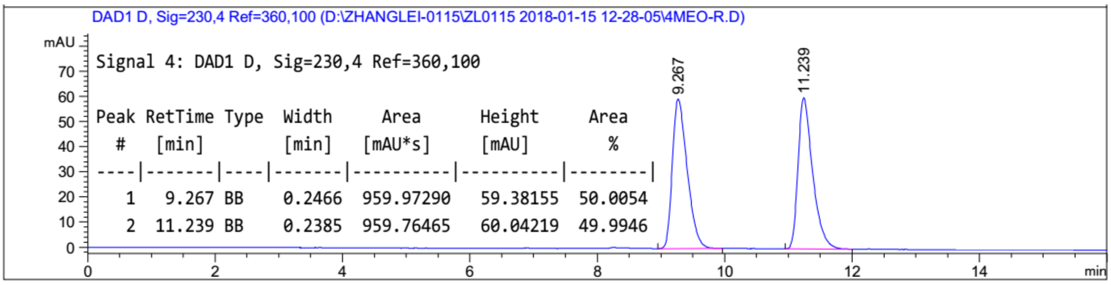


*Chiral HPLC spectrum of (R)-**4f***

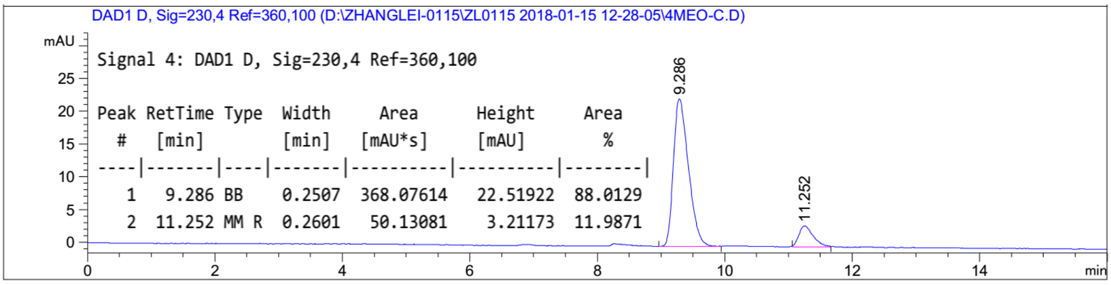


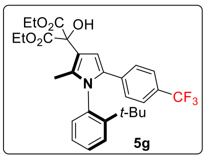


**(*S*)-Diethyl 2-(1-(2-(*tert*-butyl)phenyl)-2-methyl-5-(4-(trifluoromethyl)phenyl)-1*H*-pyrrol-3-yl)-2-hydroxymalonate ((*S*)-5g)**

According to the general procedure, *(S)*-**5g** was obtained in **45% yield** with **92% ee**.

**$^{1}$H NMR (400 MHz, CDCl$_3$)** δ 7.56 (dd, *J* = 8.2, 1.2 Hz, 1H), 7.44-7.40 (m, 1H), 7.33-7.26 (m, 3H), 7.16 (dd, *J* = 7.8, 1.3 Hz, 1H), 7.10 (d, *J* = 8.3 Hz, 2H), 6.62 (s, 1H), 4.40-4.30 (m, 4H), 4.19 (s, 1H), 1.99 (s, 3H), 1.36-1.30 (m, 6H), 0.95 (s, 9H).

**$^{13}$C NMR (100 MHz, CDCl$_3$)** δ = 170.6, 170.4, 147.5, 136.8, 135.6, 132.4, 131.9, 131.5, 130.4, 129.2, 127.2 (q, *J* = 32.4 Hz), 126.8, 126.4, 125.0 (q, *J* = 3.8 Hz), 124.3 (q, *J* = 269.9 Hz), 117.0, 109.4, 77.9, 62.8, 62.8, 36.0, 31.3, 14.1, 14.1, 12.6.

**$^{19}$F NMR (376 MHz, CDCl$_3$)** δ = -62.42.

**HRMS (ESI)** calcd for $[M+H]^+$ $C_{29}H_{33}F_3NO_5^+$, m/z: 532.2305, found: 532.2305.

**HPLC analysis**: DAICEL CHIRALPAK AD-3, hexane/isopropyl alcohol = 90/10, flow rate = 1.0 mL/min, λ = 320 nm), $t_R$ (major) = 7.99 min, $t_R$ (minor) = 13.2 min, ee = 92%.

*Chiral HPLC spectrum of racemic **5g***

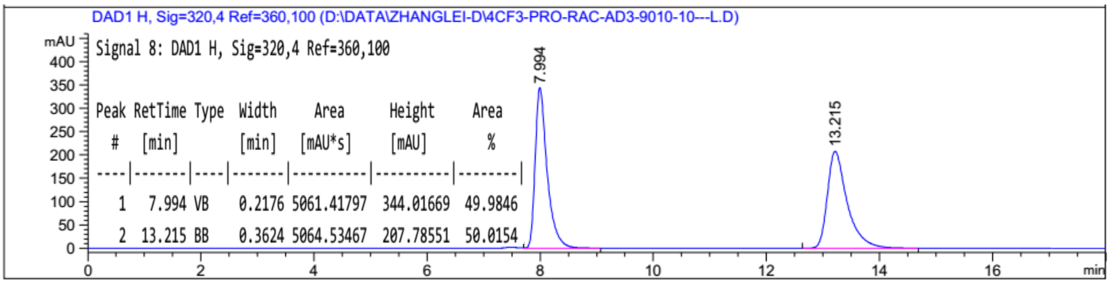


*Chiral HPLC spectrum of (S)-**5g***

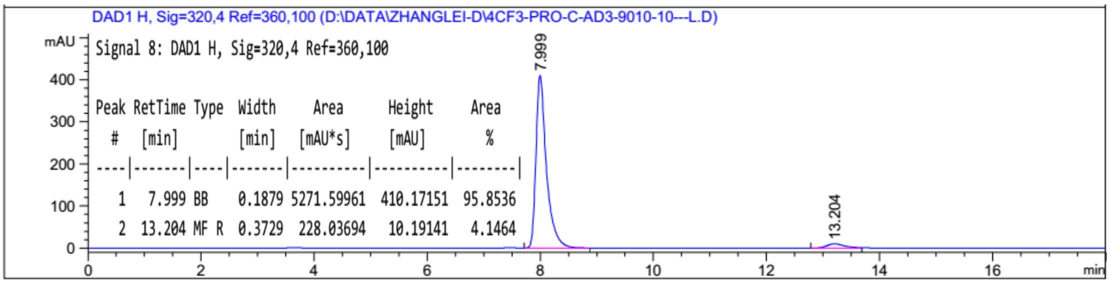


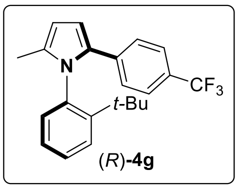


**(*R*)-1-(2-(*tert*-butyl)phenyl)-2-methyl-5-(4-(trifluoromethyl)phenyl)-1*H*-pyrrole ((*R*)-4g)**

According to the general procedure, *(R)*-**4g** was obtained in **50% yield** with **77% ee**.

**HPLC analysis**: DAICEL CHIRALCEL OD-3, hexane/isopropyl alcohol = 99/01, flow rate = 0.5 mL/min, $\lambda$ = 320 nm), $t_R$ (minor) = 6.8 min, $t_R$ (major) = 7.2 min, ee = 77%.

*Chiral HPLC spectrum of racemic* ***4g***

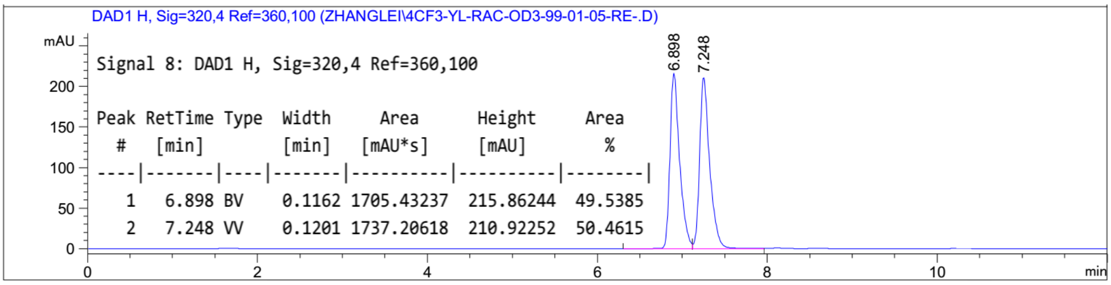


*Chiral HPLC spectrum of (R)-****4g***

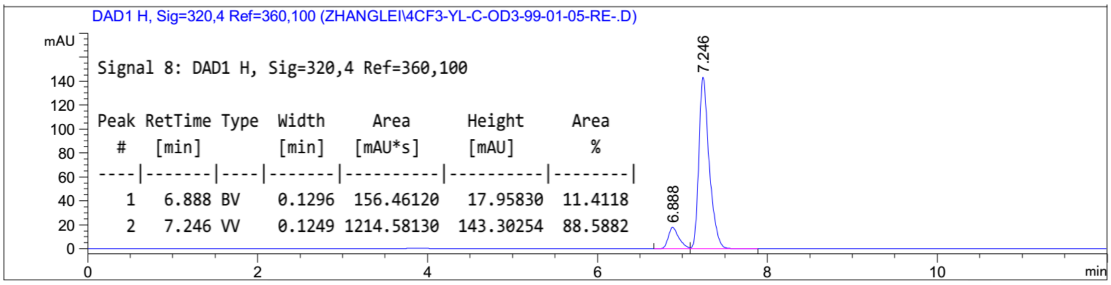


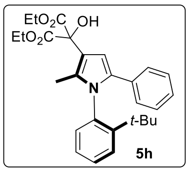


**(*S*)-Diethyl 2-(1-(2-(*tert*-butyl)phenyl)-2-methyl-5-phenyl-1*H*-pyrrol-3-yl)-2-hydroxymalonate ((*S*)-5h)**

According to the general procedure, *(S)*-**5h** was obtained in **47% yield** with **89% ee**.

**$^{1}$H NMR (400 MHz, $CDCl_3$)** δ 7.51 (d, *J* = 8.1 Hz, 1H), 7.40-7.36 (m, 1H), 7.28-7.24 (m, 1H), 7.18 (dd, *J* = 7.7, 1.1 Hz, 1H), 7.09-7.00 (m, 5H), 6.49 (s, 1H), 4.40-29 (m, 4H), 4.16 (s, 1H), 1.98 (s, 3H), 1.36-1.30 (m, 6H), 0.93 (s, 9H).
**$^{13}$C NMR (100 MHz, $CDCl_3$)** δ = 170.9, 170.6, 147.5, 135.9, 133.5, 133.2, 132.2, 130.9, 130.2, 128.8, 128.0, 127.1, 126.4, 125.7, 116.5, 107.9, 78.1, 62.7, 62.7, 36.0, 31.3, 14.1, 14.1, 12.6.
**HRMS (ESI)** calcd for $[M+H]^+$ $C_{28}H_{34}NO_5^+$, m/z: 464.2431, found: 464.2434.
**HPLC analysis**: DAICEL CHIRALCEL OD-H, hexane/isopropyl alcohol = 98/02, flow rate = 1.0 mL/min, λ = 270 nm), $t_R$ (minor) = 17.4 min, $t_R$ (major) = 20.0 min, ee = 89%.

*Chiral HPLC spectrum of racemic **5h***

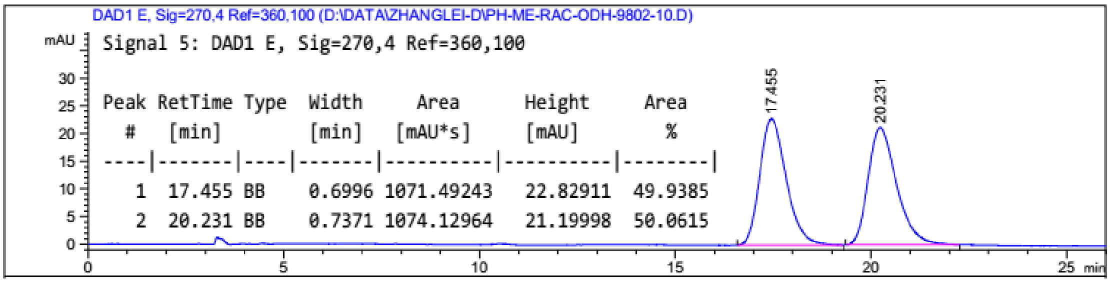


*Chiral HPLC spectrum of (S)-**5h***

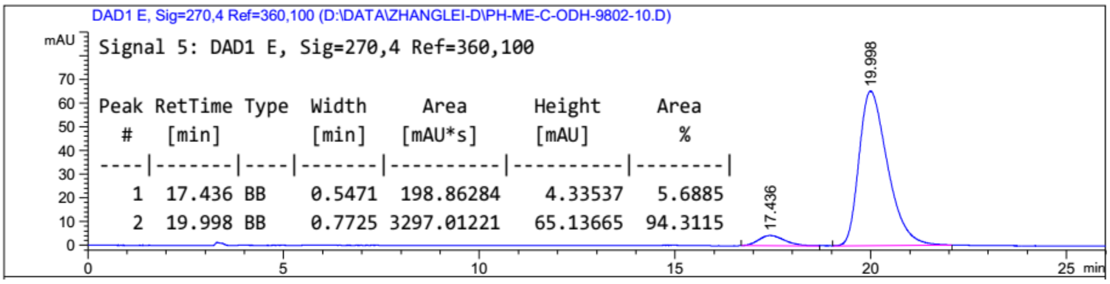


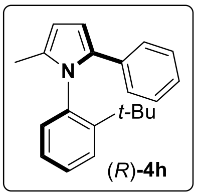


**(*R*)-1-(2-(*tert*-butyl)phenyl)-2-methyl-5-phenyl-1*H*-pyrrole ((*R*)-4h)**
According to the general procedure, *(R)*-**4h** was obtained in **48% yield** with **81% ee**.
**HPLC analysis**: DAICEL CHIRALPAK AD-3, hexane/isopropyl alcohol = 98.5/1.5, flow rate = 0.5 mL/min, λ = 300 nm), $t_R$ (major) = 6.8 min, $t_R$ (minor) = 7.4 min, ee = 81%.
*Chiral HPLC spectrum of racemic **4h***

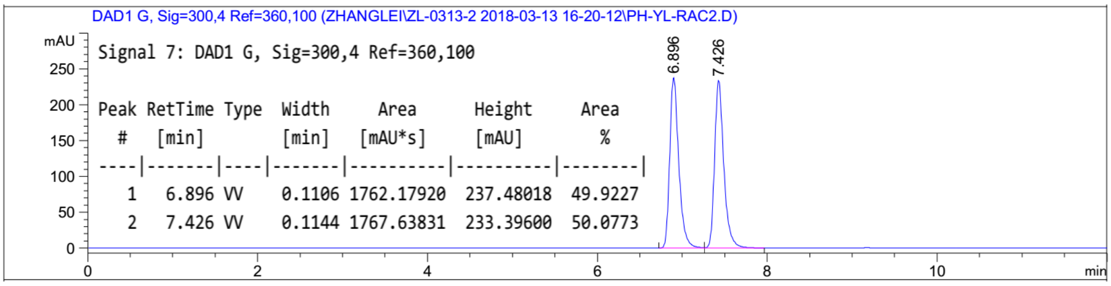


*Chiral HPLC spectrum of (R)-**4h***

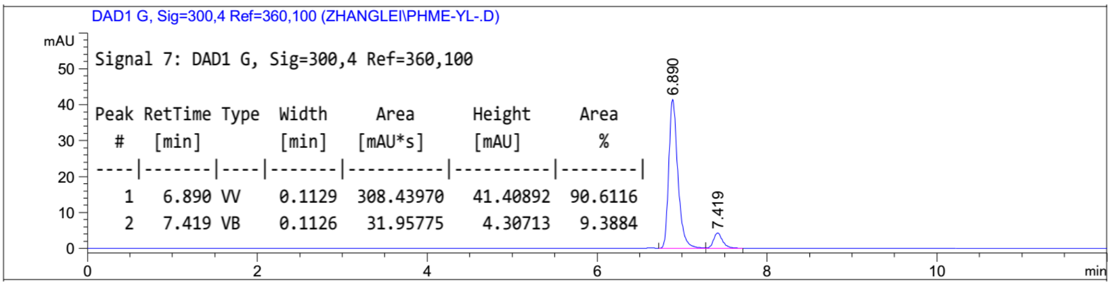


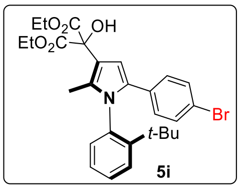


**(*S*)-Diethyl 2-(5-(4-bromophenyl)-1-(2-(*tert*-butyl)phenyl)-2-methyl-1*H*-pyrrol-3-yl)-2-hydroxymalonate ((*S*)-5i)**

According to the general procedure, *(S)*-**5i** was obtained in **43% yield** with **90% ee**.

**$^{1}$H NMR (400 MHz, CDCl$_3$)** δ 7.53 (dd, *J* = 8.1, 1.1 Hz, 1H), 7.42-7.38 (m, 1H), 7.29-7.25 (m, 1H), 7.21-7.14 (m, 3H), 6.88 (d, *J* = 8.6 Hz, 2H), 6.51 (s, 1H), 4.40-4.29 (m, 4H), 4.17 (s, 1H), 1.98 (s, 3H), 1.35-1.30 (m, 6H), 0.94 (s, 9H).

**$^{13}$C NMR (100 MHz, CDCl$_3$)** δ = 170.7, 170.5, 147.5, 135.6, 132.4, 132.1, 131.9, 131.5, 131.1, 130.3, 129.1, 128.4, 126.6, 119.5, 116.7, 108.3, 78.0, 62.7, 62.7, 36.0, 31.3, 14.1, 14.1, 12.6.

**HRMS (ESI)** calcd for $[M+H]^+$ $C_{28}H_{33}BrNO_5^+$, m/z: 542.1537, found: 542.1543.

**HPLC analysis**: DAICEL CHIRALPAK IC, hexane/isopropyl alcohol = 90/10, flow rate = 1.0 mL/min, λ = 300 nm), $t_R$ (major) = 25.0 min, $t_R$ (minor) = 29.3 min, ee = 90%.

*Chiral HPLC spectrum of racemic **5i***

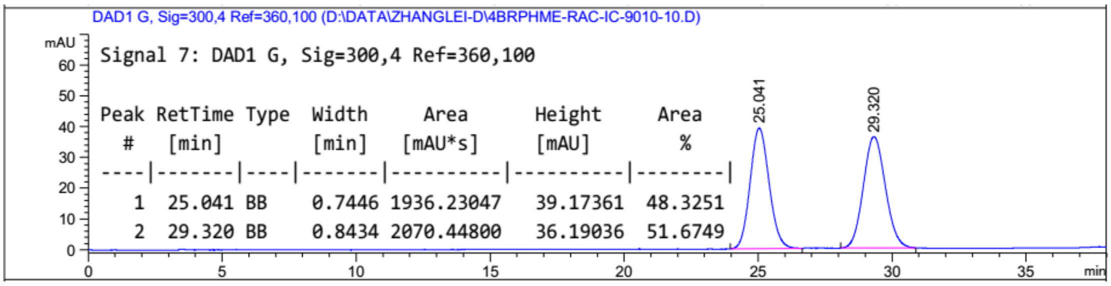


*Chiral HPLC spectrum of (S)-**5i***

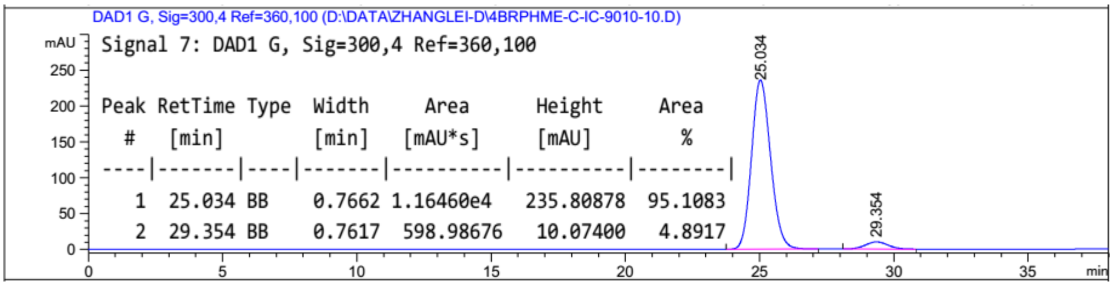

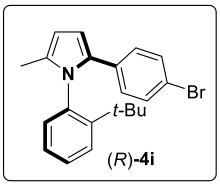


**(*R*)-2-(4-bromophenyl)-1-(2-(*tert*-butyl)phenyl)-5-methyl-1*H*-pyrrole ((*R*)-4i)**

According to the general procedure, *(R)*-**4i** was obtained in **52% yield** with **72% ee**.

**HPLC analysis**: DAICEL CHIRALPAK AD-3, hexane/isopropyl alcohol = 99.7/0.3, flow rate = 0.5 mL/min, $\lambda$ = 300 nm), $t_R$ (major) = 13.9 min, $t_R$ (minor) = 15.5 min, ee = 72%.

*Chiral HPLC spectrum of racemic **4i***

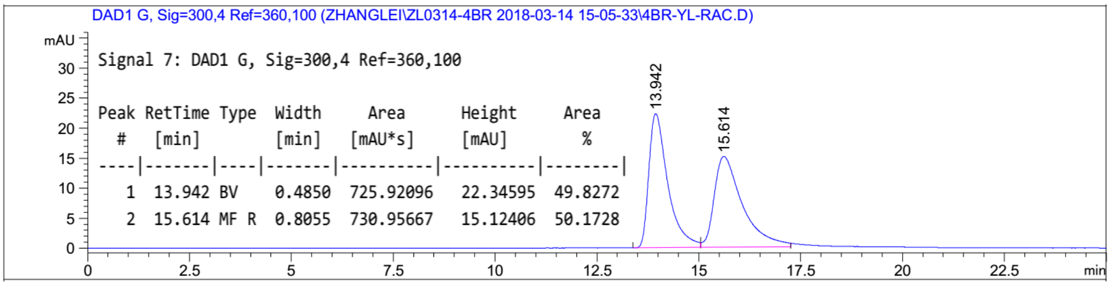


*Chiral HPLC spectrum of (R)-**4i***

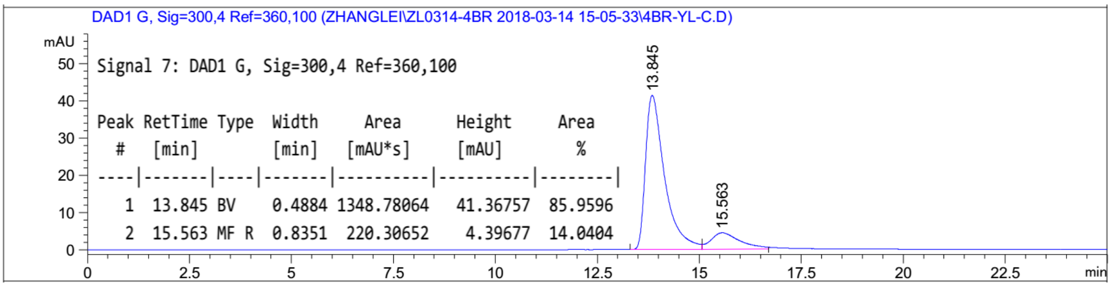


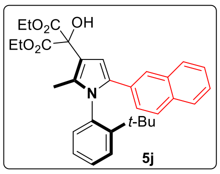


**(*S*)-Diethyl 2-(1-(2-(*tert*-butyl)phenyl)-2-methyl-5-(naphthalen-2-yl)-1*H*-pyrrol-3-yl)-2-hydroxymalonate ((*S*)-5j)**

According to the general procedure, *(S)*-**5j** was obtained in **46% yield** with **91% ee**.

**$^{1}$H NMR (400 MHz, $CDCl_3$)** δ 7.67-7.65 (m, 1H), 7.59-7.53 (m, 2H), 7.44-7.40 (m, 2H), 7.35-7.28 (m, 5H), 7.24-7.22 (m, 1H), 6.66 (s, 1H), 4.43-4.31 (m, 4H), 4.20 (s, 1H), 2.03 (s, 3H), 1.37-1.31 (m, 6H), 0.96 (s, 9H).

**$^{13}$C NMR (100 MHz, $CDCl_3$)** δ = 170.9, 170.6, 147.7, 136.2, 133.3, 132.9, 132.2, 131.5, 131.4, 130.8, 130.3, 129.0, 127.9, 127.5, 127.4, 126.6, 125.8, 125.3, 124.6, 116.8, 108.6, 78.1, 62.8, 62.7, 36.1, 31.4, 14.2, 14.1, 12.7.

**HRMS (ESI)** calcd for $[M+H]^+$ $C_{32}H_{36}NO_5^+$, m/z: 514.2588, found: 514.2592.

**HPLC analysis**: DAICEL CHIRALCEL OD-3, hexane/isopropyl alcohol = 97/03, flow rate = 1.0

mL/min, λ = 230 nm), $t_R$ (minor) = 21.4 min, $t_R$ (major) = 23.3 min, ee = 91%.

*Chiral HPLC spectrum of racemic **5j***

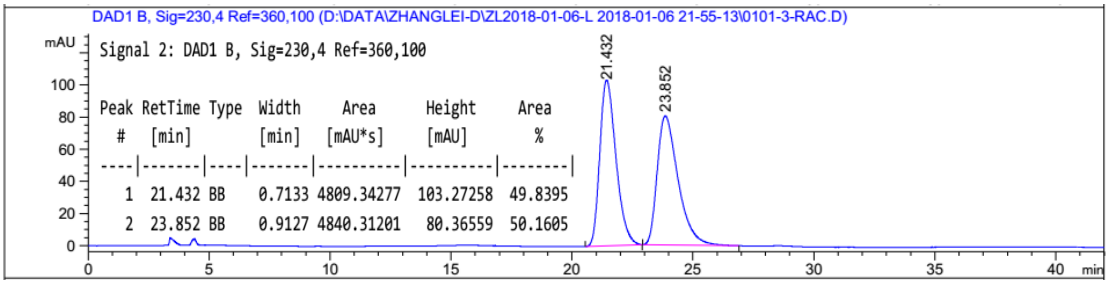


*Chiral HPLC spectrum of (S)-**5j***

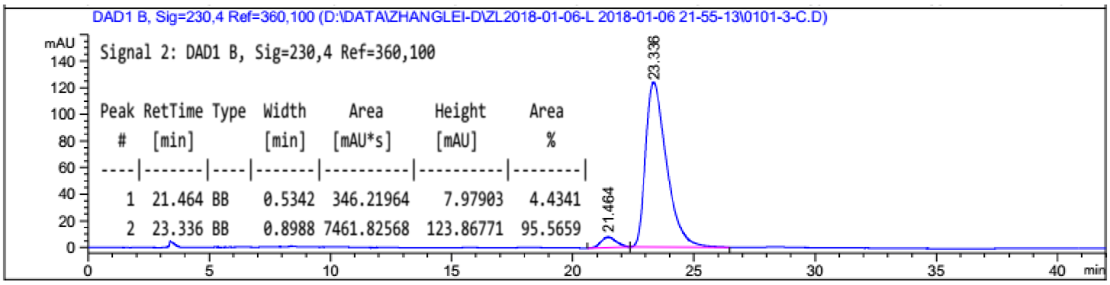


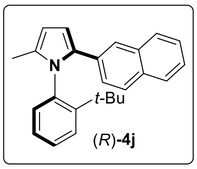


**(*R*)-1-(2-(*tert*-butyl)phenyl)-2-methyl-5-(naphthalen-2-yl)-1*H*-pyrrole ((*R*)-4j)**

According to the general procedure, *(R)*-**4j** was obtained in **51% yield** with **81% ee**.

**HPLC analysis**: DAICEL CHIRALPAK AD-3, hexane/isopropyl alcohol = 98/02, flow rate = 0.5 mL/min, λ = 270 nm), $t_R$ (major) = 8.9 min, $t_R$ (minor) = 10.9 min, ee = 81%.

*Chiral HPLC spectrum of racemic **4j***

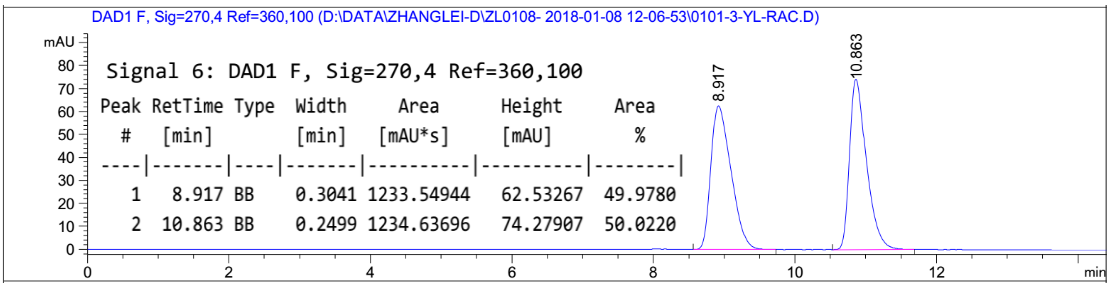


*Chiral HPLC spectrum of (R)-**4j***

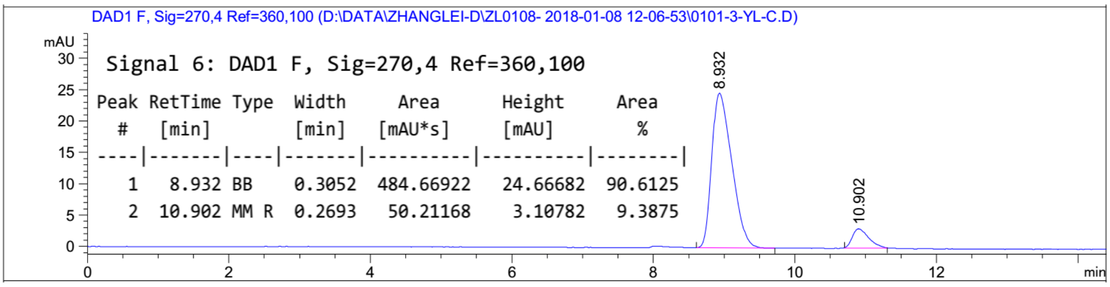


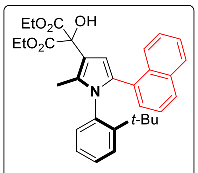


**5k**

**(*S*)-Diethyl 2-(1-(2-(*tert*-butyl)phenyl)-2-methyl-5-(naphthalen-1-yl)-1*H*-pyrrol-3-yl)-2-hydroxymalonate ((*S*)-5k)**

According to the general procedure, *(S)*-**5k** was obtained in **45% yield** with **87% ee**.

**$^1$H NMR (400 MHz, CDCl$_3$)** δ 8.35 (d, *J* = 8.2 Hz, 1H), 7.68-7.66 (m, 1H), 7.50 (d, *J* = 7.7 Hz, 1H), 7.39-7.28 (m, 3H), 7.19-6.99 (m, 5H), 6.41 (s, 1H), 4.30-4.25 (m, 4H), 4.12 (s, 1H), 1.98 (s, 3H), 1.28-1.24 (m, 6H), 0.79 (s, 9H).

**$^{13}$C NMR (100 MHz, CDCl$_3$)** δ = 170.9, 170.7, 147.4, 135.2, 133.9, 132.9, 132.6, 130.5, 130.5, 129.9, 128.5, 128.2, 128.1, 127.0, 126.8, 125.8, 125.8, 125.5, 124.5, 115.9, 111.6, 78.3, 62.7, 62.7, 36.0, 31.3, 14.2, 14.1, 12.8.

**HRMS (ESI)** calcd for $[M+H]^+$ $C_{32}H_{36}NO_5^+$, m/z: 514.2588, found: 514.2589.

**HPLC analysis**: DAICEL CHIRALPAK AD-3, hexane/isopropyl alcohol = 90/10, flow rate = 1.0 mL/min, λ = 230 nm), $t_R$ (major) = 14.4 min, $t_R$ (minor) = 21.0 min, ee = 87%.

*Chiral HPLC spectrum of racemic* ***5k***

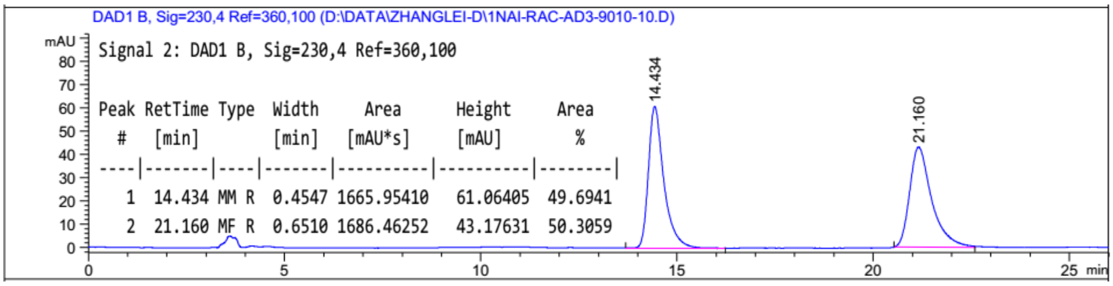


*Chiral HPLC spectrum of (S)-**5k***

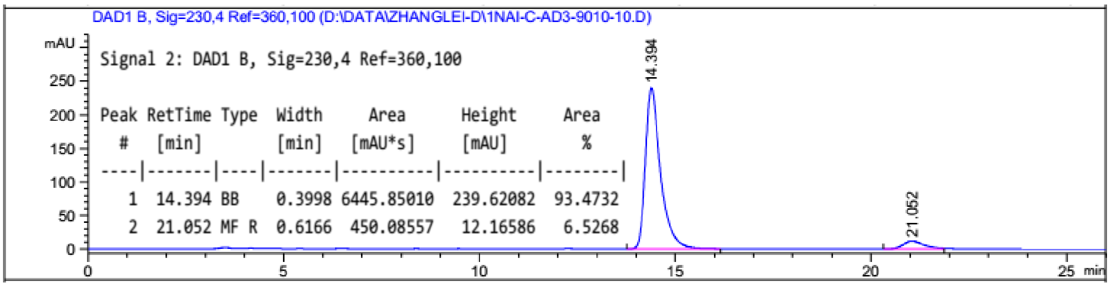

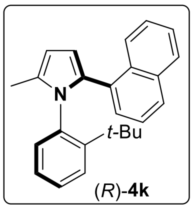


**(*R*)-1-(2-(*tert*-butyl)phenyl)-2-methyl-5-(naphthalen-1-yl)-1*H*-pyrrole ((*R*)-4k)**

According to the general procedure, *(R)*-**4k** was obtained in **51% yield** with **75% ee**.

**HPLC analysis**: DAICEL CHIRALPAK AD-3, hexane/isopropyl alcohol = 99.7/0.3, flow rate = 0.5 mL/min, $\lambda$ = 254 nm), $t_R$ (major) = 17.2 min, $t_R$ (minor) = 20.9 min, ee = 75%.

*Chiral HPLC spectrum of racemic* ***4k***

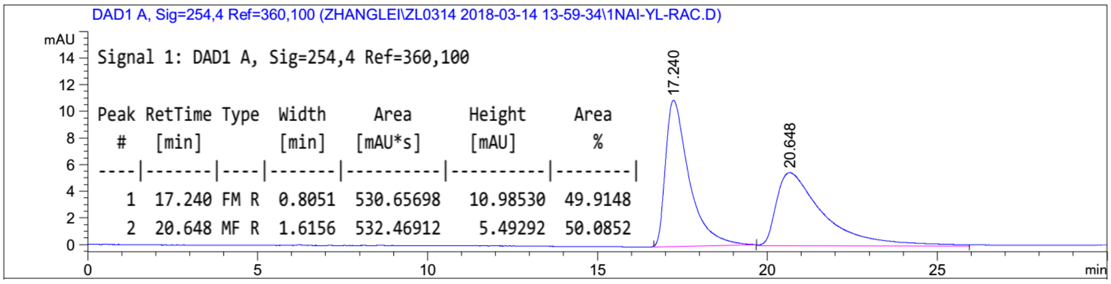


*Chiral HPLC spectrum of (R)-****4k***

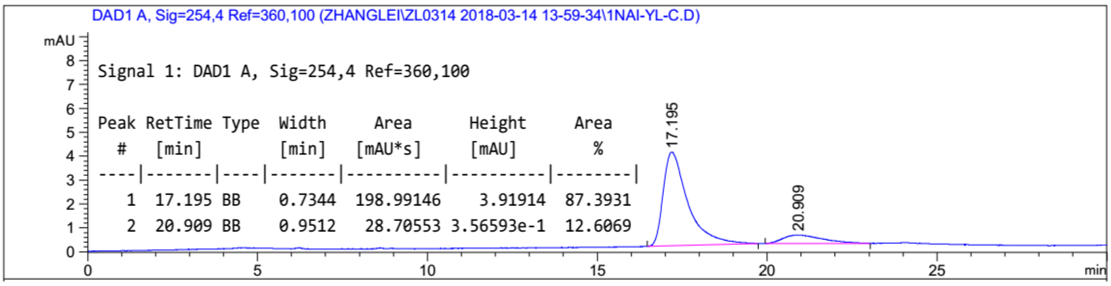


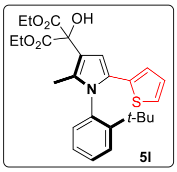


**(*S*)-Diethyl**
**2-(1-(2-(*tert*-butyl)phenyl)-2-methyl-5-(thiophen-2-yl)-1*H*-pyrrol-3-yl)-2-hydroxymalonate ((*S*)-5l)**

According to the general procedure, *(S)*-**5l** was obtained in **42% yield** with **94% ee**.

**$^1$H NMR (400 MHz, CDCl$_3$)** $\delta$ 7.59 (dd, *J* = 8.2, 1.2 Hz, 1H), 7.47-7.43(m, 1H), 7.29-7.25 (m, 1H), 7.10 (dd, *J* = 7.8, 1.4 Hz, 1H), 6.91 (dd, *J* = 5.1, 0.6 Hz, 1H), 6.74-6.71 (m, 1H), 6.54 (s, 1H), 6.36 (dd, *J* = 3.6, 0.6 Hz, 1H), 4.40-4.30 (m, 4H), 4.16 (d, *J* = 1.3 Hz, 1H), 1.98 (s, 3H), 1.36-1.29 (m, 6H), 1.03 (s, 9H).

**$^{13}$C NMR (100 MHz, CDCl$_3$)** $\delta$ = 170.7, 170.5, 148.1, 136.0, 135.3, 132.2, 130.8, 130.2, 129.4, 127.6, 126.8, 122.7, 122.4, 116.6, 107.5, 77.9, 62.7, 62.7, 36.2, 31.4, 14.1, 14.1, 12.5.

**HRMS (ESI)** calcd for $[M+H]^+$ $C_{26}H_{32}NO_5S^+$, m/z: 470.1996, found: 470.2001.

**HPLC analysis**: DAICEL CHIRALPAK AD-3, hexane/isopropyl alcohol = 95/05, flow rate = 1.0 mL/min, $\lambda$ = 300 nm), $t_R$ (major) = 22.4 min, $t_R$ (minor) = 31.6 min, ee = 94%.

*Chiral HPLC spectrum of racemic **5l***

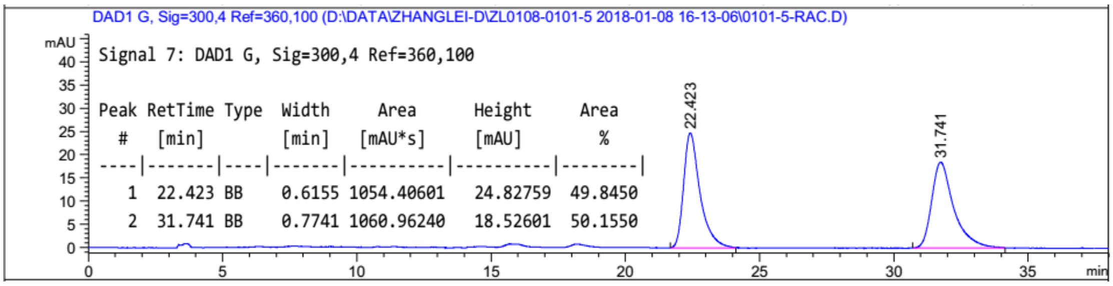


*Chiral HPLC spectrum of (S)-**5l***

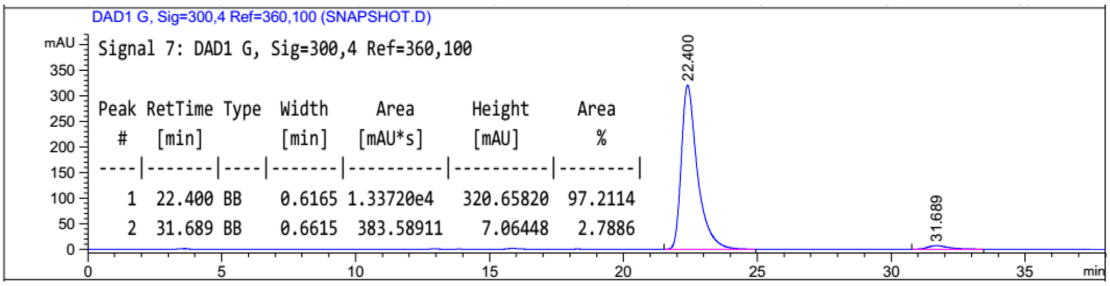


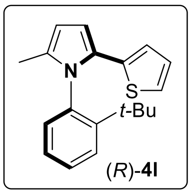


**(*R*)-1-(2-(*tert*-butyl)phenyl)-2-methyl-5-(thiophen-2-yl)-1*H*-pyrrole ((*R*)-4l)**

According to the general procedure, *(R)*-**4l** was obtained in **54% yield** with **71% ee**.

**HPLC analysis**: DAICEL CHIRALCEL OD-3, hexane/isopropyl alcohol = 98/02, flow rate = 0.5 mL/min, $\lambda$ = 240 nm), $t_R$ (major) = 8.9 min, $t_R$ (minor) = 10.2 min, ee = 71%.

*Chiral HPLC spectrum of racemic **4l***

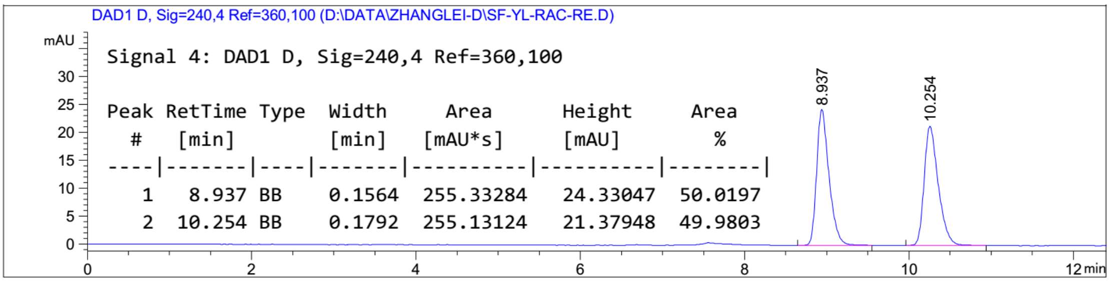


*Chiral HPLC spectrum of (R)-**4l***

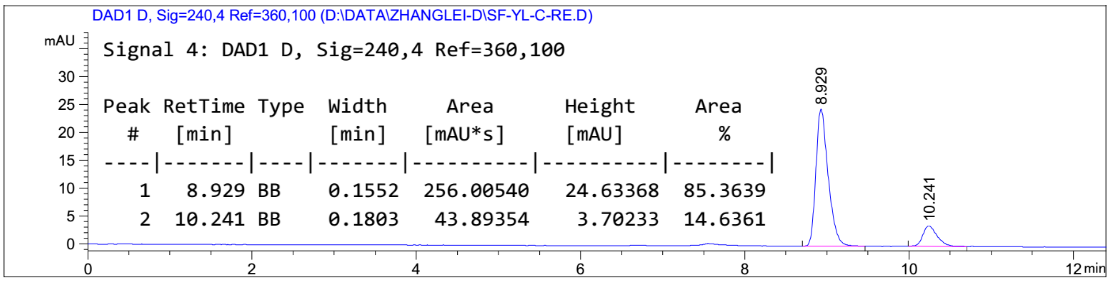

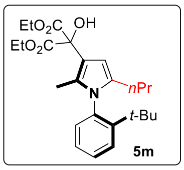


**(*R*)-Diethyl (*R*)-2-(1-(2-(tert-butyl)phenyl)-2-methyl-5-propyl-1H-pyrrol-3-yl)-2-hydroxymalonate ((*R*)-5m)**

According to the general procedure, *(R)*-**5m** was obtained in **45% yield** with **91% ee**.

**$^1$H NMR (400 MHz, CDCl$_3$)** δ 7.58 (d, *J* = 7.9 Hz, 1H), 7.37 (t, *J* = 7.2 Hz, 1H), 7.21 (t, *J* = 7.2 Hz, 1H), 6.92 (d, *J* = 7.2 Hz, 1H), 5.95 (s, 1H), 4.37-4.27 (m, 4H), 4.06 (s, 1H), 2.18-2.04 (m, 2H), 1.88 (s, 3H), 1.58-1.49 (m, 2H), 1.34-1.27 (m, 6H), 1.12 (s, 9H), 0.85 (t, *J* = 7.3 Hz, 3H).

**$^{13}$C NMR (100 MHz, CDCl$_3$)** δ = 171.0, 170.8, 147.6, 135.3, 133.0, 131.6, 129.8, 128.6, 127.7, 126.5, 114.9, 104.2, 78.3, 62.5, 62.5, 36.1, 31.7, 29.5, 21.6, 14.2, 14.1, 14.1, 12.3.

**HRMS (ESI)** calcd for $[M+H]^+$ $C_{25}H_{36}NO_5^+$, m/z: 430.2588, found: 430.2589.

**HPLC analysis**: DAICEL CHIRALPAK AD-3, hexane/isopropyl alcohol = 98/02, flow rate = 1.0 mL/min, λ = 240 nm), $t_R$ (minor) = 14.9 min, $t_R$ (major) = 18.5 min, ee = 91%.

*Chiral HPLC spectrum of racemic* ***5m***

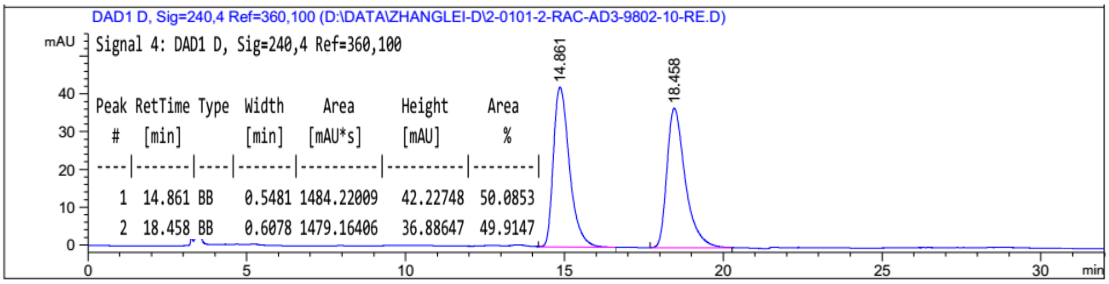


*Chiral HPLC spectrum of (R)-****5m***

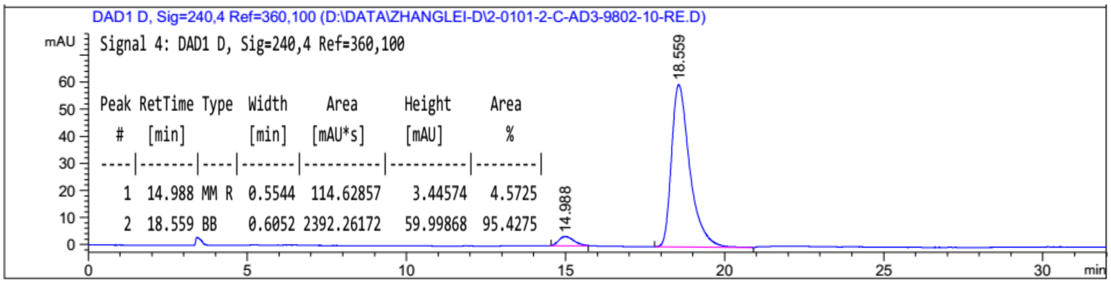


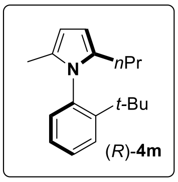


**(*R*)-1-(2-(*tert*-butyl)phenyl)-2-methyl-5-propyl-1*H*-pyrrole ((*R*)-4m)**

According to the general procedure, *(R)*-**4m** was obtained in **50% yield** with **82% ee**.

**HPLC analysis**: DAICEL CHIRALCEL OD-3, hexane/isopropyl alcohol = 98/02, flow rate = 0.5 mL/min, λ = 240 nm), $t_R$ (minor) = 7.1 min, $t_R$ (major) = 7.4 min, ee = 82%.

*Chiral HPLC spectrum of racemic* ***4m***

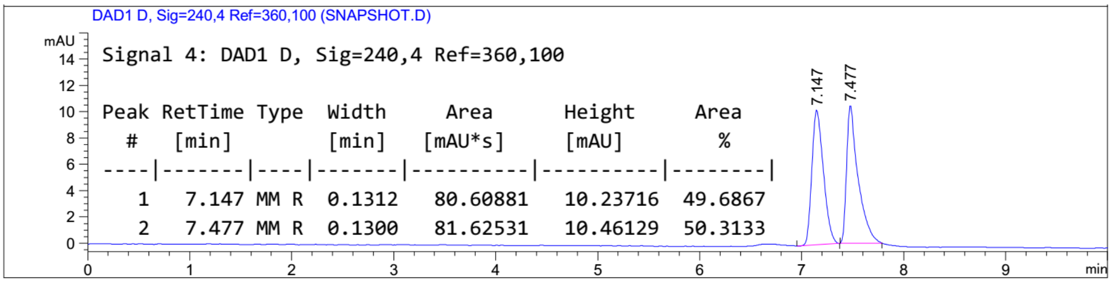


*Chiral HPLC spectrum of (R)-****4m***

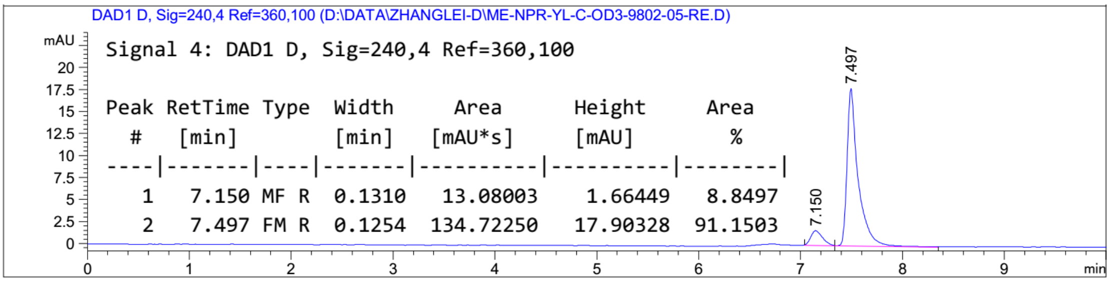


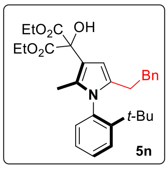


**(*R*)-Diethyl-2-(1-(2-(*tert*-butyl)phenyl)-2-methyl-5-phenethyl-1*H*-pyrrol-3-yl)-2-hydroxymalonate ((*R*)-5n)**

According to the general procedure, *(R)*-**5n** was obtained in **47% yield** with **90% ee**.

**$^1$H NMR (400 MHz, CDCl$_3$)** δ 7.58 (dd, *J* = 8.2, 1.3 Hz, 1H), 7.39-7.34 (m, 1H), 7.24-7.13 (m, 4H), 7.06-7.04 (m, 2H), 6.87 (dd, *J* = 7.7, 1.4 Hz, 1H), 6.09 (s, 1H), 4.42-4.29 (m, 4H), 4.10 (s, 1H), 2.91-2.79 (m, 2H), 2.56-2.48 (m, 1H), 2.45-2.37 (m, 1H), 1.91 (s, 3H), 1.36-1.30 (m, 6H), 1.14 (s, 9H).

**$^{13}$C NMR (100 MHz, CDCl$_3$)** δ = 171.0, 170.8, 147.6, 141.9, 135.2, 132.4, 131.6, 129.8, 128.8, 128.4, 128.3, 128.1, 126.6, 125.9, 115.1, 104.5, 78.2, 62.6, 62.5, 36.1, 35.1, 31.7, 29.8, 14.1, 14.1, 12.4.

**HRMS (ESI)** calcd for $[M+H]^+$ $C_{30}H_{38}NO_5^+$, m/z: 492.2744, found: 492.2747.

**HPLC analysis**: DAICEL CHIRALCEL OD-3, hexane/isopropyl alcohol = 98/02, flow rate = 0.8 mL/min, λ = 230 nm), $t_R$ (minor) = 22.6 min, $t_R$ (major) = 24.6 min, ee = 90%.

*Chiral HPLC spectrum of racemic* ***5n***

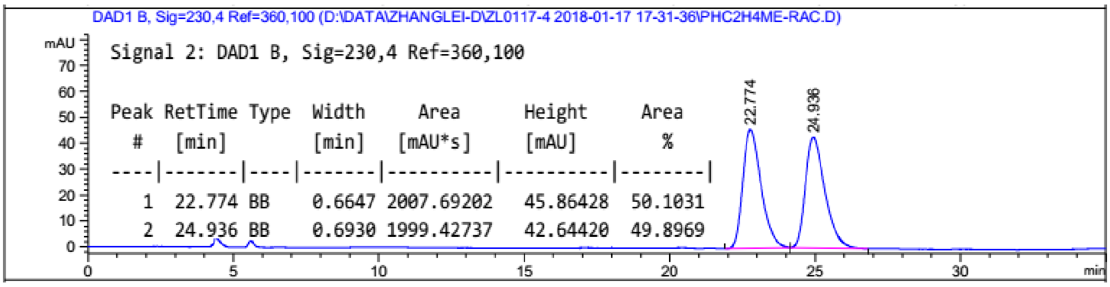


*Chiral HPLC spectrum of (R)-****5n***

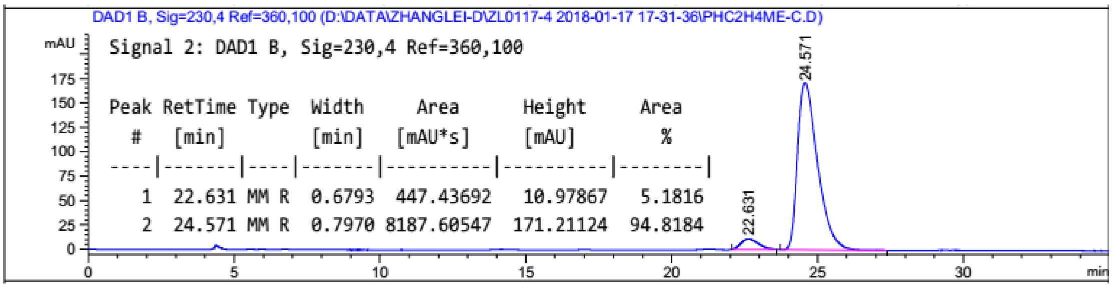


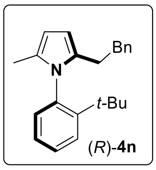


**(*R*)-1-(2-(*tert*-butyl)phenyl)-2-methyl-5-phenethyl-1*H*-pyrrole ((*R*)-4n)**

According to the general procedure, *(R)*-**4n** was obtained in **46% yield** with **85% ee**.

**HPLC analysis**: DAICEL CHIRALCEL OD-3, hexane/isopropyl alcohol = 98/02, flow rate = 0.5 mL/min, λ = 240 nm), $t_R$ (minor) = 8.7 min, $t_R$ (major) = 10.0 min, ee = 85%.

*Chiral HPLC spectrum of racemic **4n***

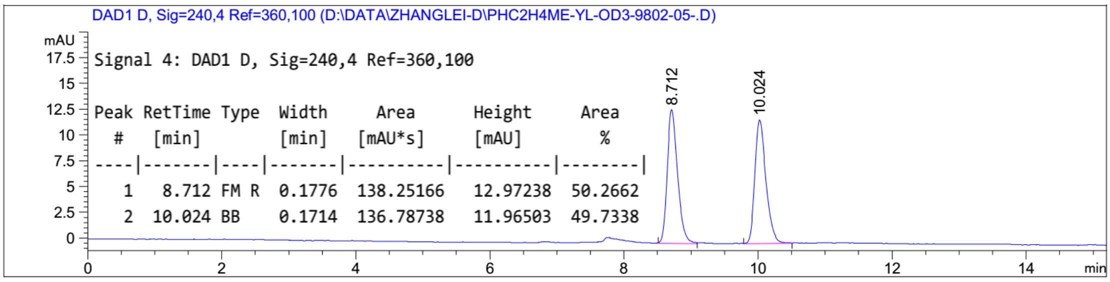


*Chiral HPLC spectrum of (R)-**4n***

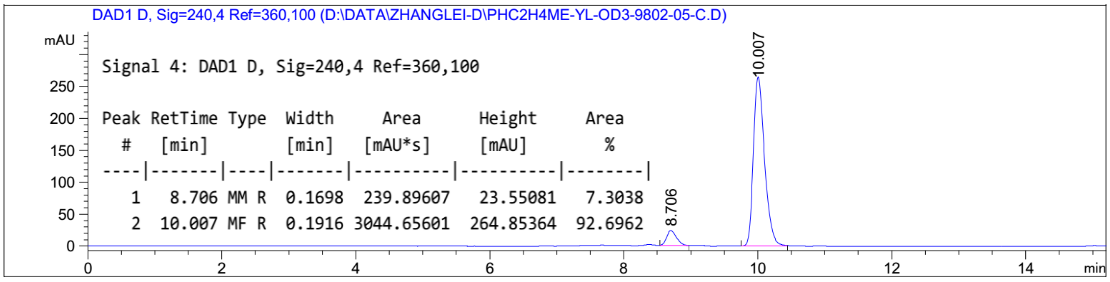

## Gram Scale Reaction

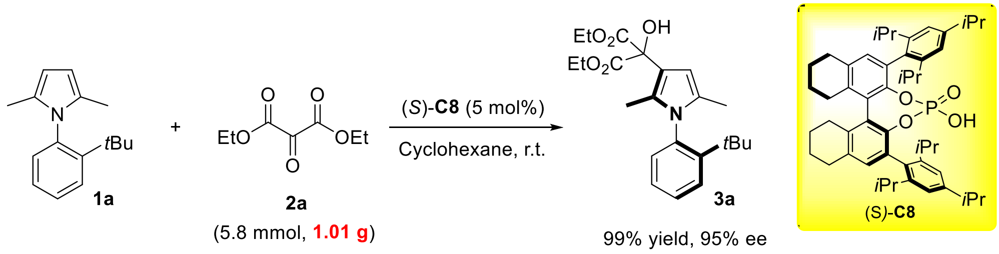


An oven-dried 100 mL of Schlenk tube was charged with arylpyrroles **1a** (8.70 mmol), (*S*)-**C8** (0.29 mmol), 50 mL of dry cyclohexane, and the mixture was stirred at ambient temperature for 10 min. After that a solution of ketomalonate **2a** (5.80 mmol) in dry cyclohexane (5.0 mL) was added dropwise to the above solution and the mixture was stirred at ambient temperature for 48 hours. Then the mixture was concentrated under reduced pressure and purified by flash chromatography eluted with PE/EA (10/1 to 5/1) to afford the corresponding axially chiral arylpyrrole product (*R*)-**3a** with 99% yield and 95% ee.

## Versatile transformations from the compound (*R*)-3a

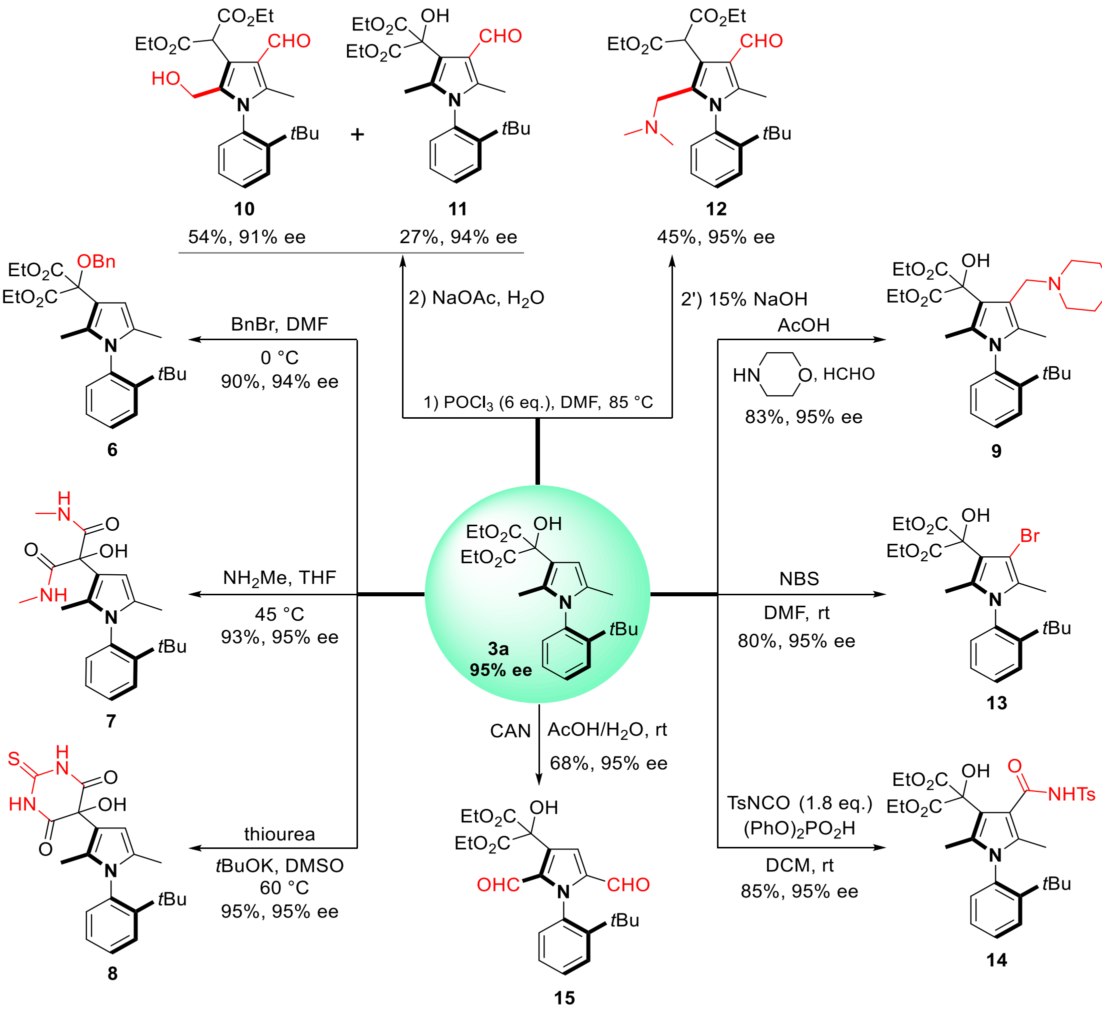

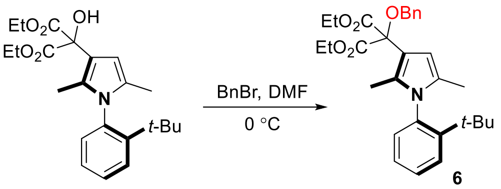


(*R*)-**3a**
95% ee

90% yield
94% ee

According to the literature,[5] a suspension of NaH (60% in mineral oil) (8.0 mg, 0.12 mmol) in dry DMF (1.0 mL) was cooled in an ice bath. Then a solution of (*R*)-**3a** (40.1 mg, 0.1 mmol) in dry DMF (1.0 mL) was added dropwise over 30 minutes. After stirring the mixture for 15 minutes, benzyl bromide (13.4 μL, 0.11 mmol) was added and the mixture was stirred at room temperature for 6 hours. Then the reaction was quenched with $H_2O$/saturated aqueous $NH_4Cl$ 1:1 (2.0 mL) and extracted with PE/$Et_2O$ 1:1 (3×2.0 mL). The organic layer was then washed with water (5.0 mL). After drying ($Na_2SO_4$) and evaporation, the crude product was finally purified by silica chromatography. The corresponding product **6** was obtained with 90% yield and 94% ee.

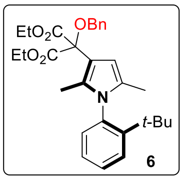


**Diethyl 2-(benzyloxy)-2-(1-(2-(*tert*-butyl)phenyl)-2,5-dimethyl-1*H*-pyrrol-3-yl)malonate (6)**

**$^{1}$H NMR (400 MHz, $CDCl_3$)** δ 7.59 (dd, *J* = 8.2, 1.4 Hz, 1H), 7.45-7.34 (m, 3H), 7.34-7.27 (m, 2H), 7.25-7.17 (m, 2H), 6.89 (dd, *J* = 7.8, 1.5 Hz, 1H), 6.15 (d, *J* = 0.7 Hz, 1H), 4.63 (s, 2H), 4.34-4.19 (m, 4H), 1.93 (s, 3H), 1.88 (s, 3H), 1.32-1.26 (m, 6H), 1.13 (s, 9H).

**$^{13}$C NMR (100 MHz, $CDCl_3$)** δ = 168.7, 168.6, 147.6, 138.5, 135.4, 131.5, 129.8, 129.0, 128.7, 128.5, 128.1, 127.6, 127.3, 126.7, 112.8, 107.1, 83.7, 67.7, 61.8, 61.7, 36.1, 31.7, 14.2, 13.2, 12.5.

**HRMS (ESI)** calcd for $[M+H]^+$ $C_{30}H_{38}NO_5^+$, m/z: 492.2744, found: 492.2742.

**HPLC analysis**: DAICEL CHIRALPAK ID, hexane/isopropyl alcohol = 98/02, flow rate = 0.8 mL/min, λ = 254 nm), $t_R$ (major) = 20.8 min, $t_R$ (minor) = 22.9 min, ee = 94%.

*Chiral HPLC spectrum of racemic **6***

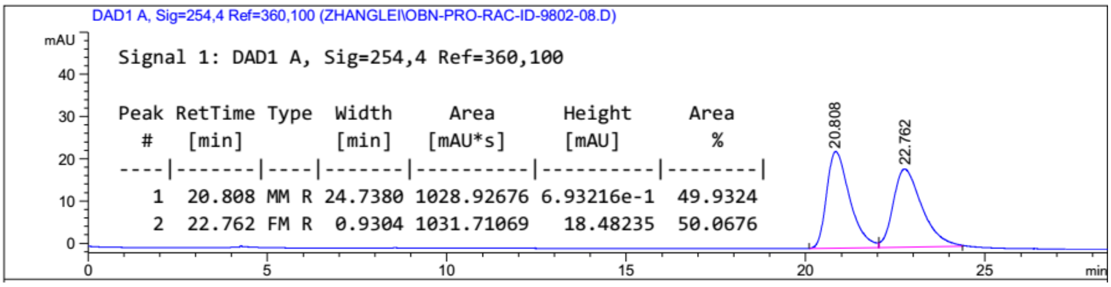


*Chiral HPLC spectrum of **6***

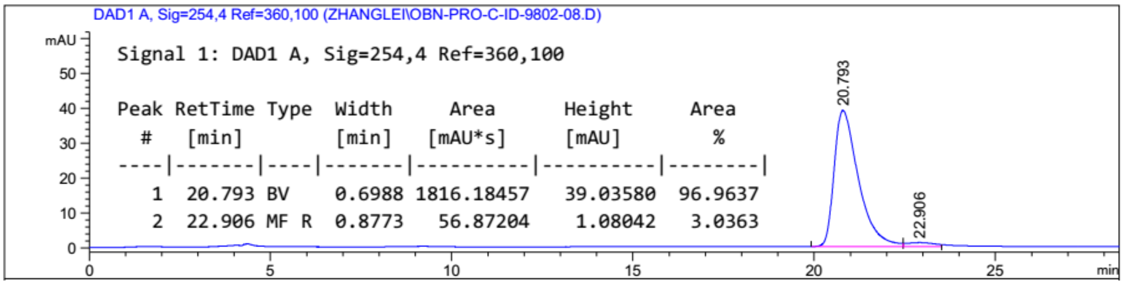


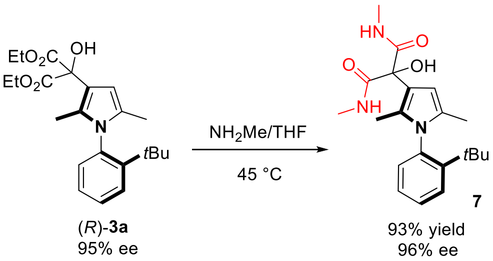


Compound **7** was prepared according to the literature.[6] To a solution of (*R*)-**3a** (40.1 mg, 0.10 mmol) in THF (1.0 mL), methylamine (2 M in THF, 5.0 mL) was added. After stirring at 45 °C for 72 hours, the mixture was concentrated *in vacuo* and chromatographed directly on silica gel to afford **7** in 93% yield and 96% ee.

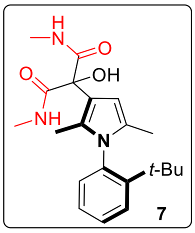


**2-(1-(2-(*tert*-Butyl)phenyl)-2,5-dimethyl-1*H*-pyrrol-3-yl)-2-hydroxy-N1,N3-dimethylmalonamide (7)**

**$^1$H NMR (400 MHz, CDCl$_3$)** δ 7.58 (dd, *J* = 8.1, 1.1 Hz, 1H), 7.43-7.42 (m, 1H), 7.40-7.32 (m, 1H), 7.29-7.27 (m, 1H), 7.22-7.18 (m, 1H), 6.90 (dd, *J* = 7.7, 1.3 Hz, 1H), 5.88 (s, 1H), 5.38 (s, 1H), 2.90 (d, *J* = 5.0 Hz, 3H), 2.87 (d, *J* = 5.0 Hz, 3H), 1.87 (s, 3H), 1.82 (s, 3H), 1.10 (s, 9H).

**$^{13}$C NMR (100 MHz, CDCl$_3$)** δ = 171.9, 171.7, 147.7, 135.3, 131.4, 129.7, 128.7, 128.1, 128.0, 126.6, 118.4, 105.8, 76.7, 36.0, 31.6, 26.6, 26.5, 13.0, 11.8.

**$^{13}$C NMR-DEPT 135 (100 MHz, CDCl$_3$)** δ = 131.4, 129.7, 128.7, 126.6, 105.8, 31.6, 26.6, 26.5, 13.0, 11.8.

**HRMS (ESI)** calcd for $[M+H]^+$ $C_{21}H_{30}N_3O_3^+$, m/z: 372.2282, found: 372.2285.

**HPLC analysis**: DAICEL CHIRALCEL OD-3, hexane/isopropyl alcohol = 90/10, flow rate = 1.0 mL/min, λ = 210 nm), $t_R$ (major) = 9.7 min, $t_R$ (minor) = 17.2 min, ee = 96%.

*Chiral HPLC spectrum of racemic **7***

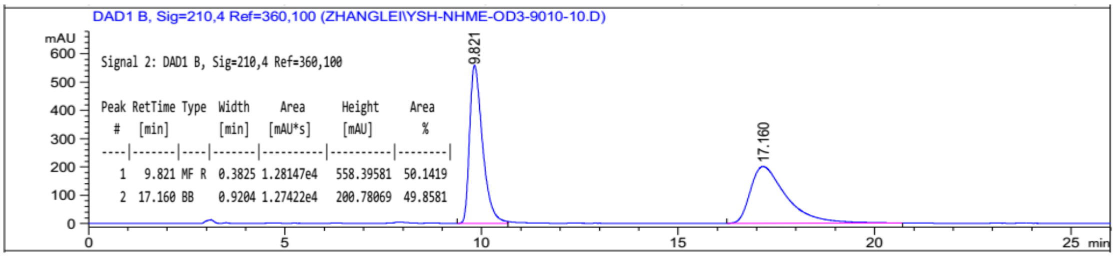


Chiral HPLC spectrum of **7**

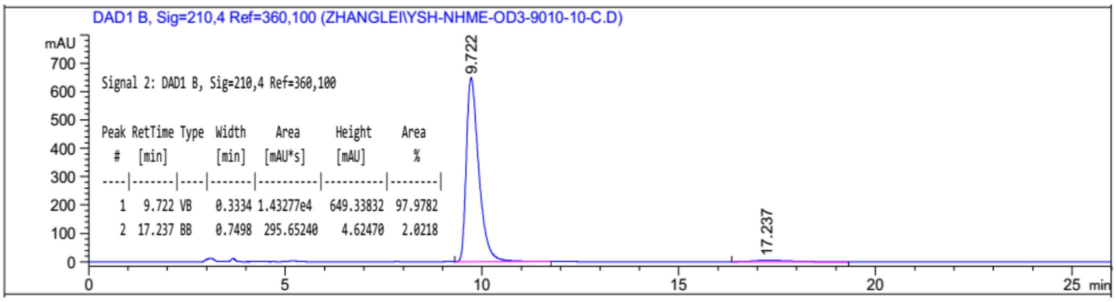


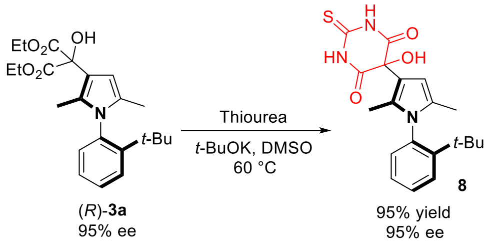


Compound **8** was prepared according to the literature.[7] Thiourea (45.7 mg, 0.6 mmol, 6.0 equiv) was added to a solution of (*R*)-**3a** (0.1 mmol, 1.0 equiv, 40.1 mg) in dry DMSO (2 mL). Then a solution 1M of potassium *tert*-butoxide (25.0 mg, 0.22 mmol, 2.2 equiv.) was added dropwise. After stirring at 60 ℃ for 12 hours, the reaction mixture was diluted with ethyl acetate (5 mL) and washed with a solution of 1N hydrochloric acid. The layers were separated and the aqueous phase was extracted with ethyl acetate. The collected organic phase was washed with brine, dried over anhydrous $Na_2SO_4$, filtered and concentrated**.** The residue was purified with column chromatography, affording the corresponding **8** with 95% yield and 95% ee.

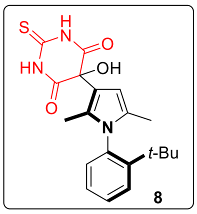


**5-(1-(2-(*tert*-Butyl)phenyl)-2,5-dimethyl-1*H*-pyrrol-3-yl)-5-hydroxy-2-thioxodihydropyrimidine-4,6(1H,5H)-dione (8)**

**$^{1}$H NMR (400 MHz, CDCl$_3$)** δ 9.65 (s, 1H), 9.56 (s, 1H), 7.58 (dd, *J* = 8.2, 1.2 Hz, 1H), 7.44-7.33 (m, 1H), 7.23-7.19 (m, 1H), 6.86 (dd, *J* = 7.8, 1.3 Hz, 1H), 5.76 (s, 1H), 4.08 (br, 1H), 2.04 (s, 3H), 1.85 (s, 3H), 1.07 (s, 9H).

**$^{13}$C NMR (100 MHz, CDCl$_3$)** δ = 176.2, 167.7, 167.7, 147.5, 134.5, 131.2, 130.7, 130.0, 129.9, 129.1, 126.8, 113.4, 104.2, 75.9, 36.1, 31.6, 13.1, 12.3.

**$^{13}$C NMR-DEPT 135 (100 MHz, CDCl$_3$)** δ = 131.2, 130.0, 129.1, 126.8, 104.2, 31.6, 13.1, 12.3.

**HRMS (ESI)** calcd for $[M+H]^+$ $C_{20}H_{24}N_3O_3S^+$, m/z: 386.1533, found: 386.1535.

**HPLC analysis**: DAICEL CHIRALPAK IB, hexane/isopropyl alcohol/TFA= 90/10/0.1, flow rate = 1.0 mL/min, $\lambda$ = 300 nm), $t_R$ (minor) = 33.9 min, $t_R$ (major) = 35.7 min, ee = 95%.

*Chiral HPLC spectrum of racemic **8***

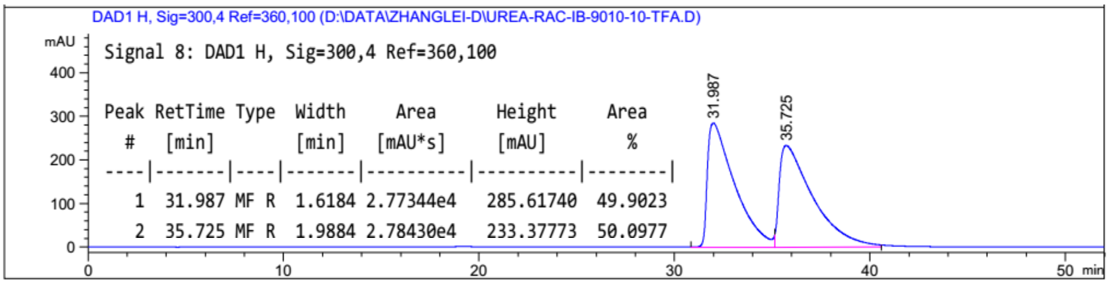


*Chiral HPLC spectrum of **8***

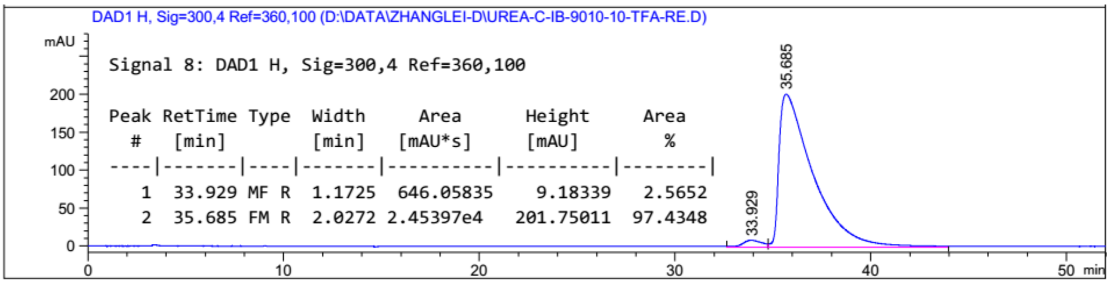


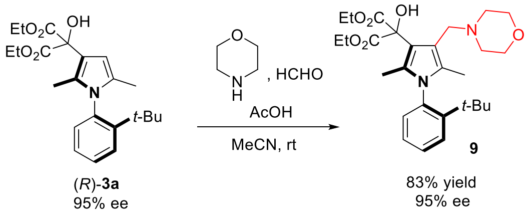


Compound **9** was prepared according to the literature.[8] To a solution of (*R*)-**3a** (0.1 mmol, 1.0 equiv, 40.1 mg) in acetonitrile (0.3 mL) was added a mixture of morpholine (0.12 mmol, 1.2 equiv, 10.5 μL), formaldehyde (40% in water, 1.2 equiv, 8.6 μL) and acetic acid (87.6 μL). After the mixture was stirred at r.t. for 3 hours (monitored by TLC), it was treated with a solution of aq. NaOH (20%, w/v) and extracted with EtOAc. The organic phase was separated, washed with brine, dried over anhydrous $Na_2SO_4$, filtered and concentrated. The residue was purified by a silica gel flash chromatography (Hexane/EtOAc) to give compound **9** with 83% yield and 95% ee.

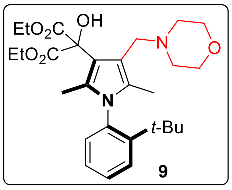


**Diethyl 2-(1-(2-(tert-butyl)phenyl)-2,5-dimethyl-4-(morpholinomethyl)-1H-pyrrol-3-yl)-2-hydroxymalonate (9)**

**$^{1}$H NMR (400 MHz, $CDCl_3$)** δ 7.57 (dd, *J* = 8.1, 1.1 Hz, 1H), 7.42-7.32 (m, 1H), 7.23-7.18 (m, 1H), 6.88 (dd, *J* = 7.7, 1.3 Hz, 1H), 4.32-4.12 (m, 4H), 3.75-3.72 (m, 4H), 3.46 (d, *J* = 13.0 Hz, 1H), 3.32 (d, *J* = 13.0 Hz, 1H), 2.52 (br, 4H), 1.84 (s, 3H), 1.81 (s, 3H), 1.28 (t, *J* = 7.1 Hz, 3H), 1.23 (t, *J* = 7.1 Hz, 3H), 1.10 (s, 9H).
**$^{13}$C NMR (100 MHz, $CDCl_3$)** δ = 171.2, 170.7, 147.7, 135.4, 131.5, 129.8, 128.8, 127.7, 127.5, 126.7, 115.6, 112.5, 79.7, 66.7, 61.7, 61.5, 53.7, 52.3, 36.1, 31.6, 14.1, 12.7, 11.4.
**$^{13}$C NMR-DEPT 135 (100 MHz, $CDCl_3$)** δ = 131.5, 129.8, 128.8, 126.7, 66.7, 61.7, 61.5, 53.7, 52.3, 31.6, 14.1, 12.7, 11.4.
**HRMS (ESI)** calcd for $[M+H]^+$ $C_{28}H_{41}N_2O_6^+$, m/z: 501.2959, found: 501.2951.
**HPLC analysis**: DAICEL CHIRALPAK IC, hexane/isopropyl alcohol = 70/30, flow rate = 1.0 mL/min, λ = 230 nm), $t_R$ (minor) = 30.4 min, $t_R$ (major) = 37.4 min, ee = 95%.

*Chiral HPLC spectrum of racemic* ***9***

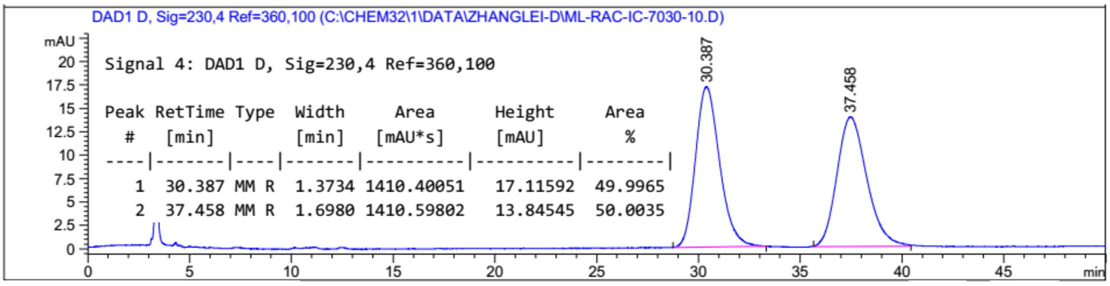


*Chiral HPLC spectrum of* ***9***

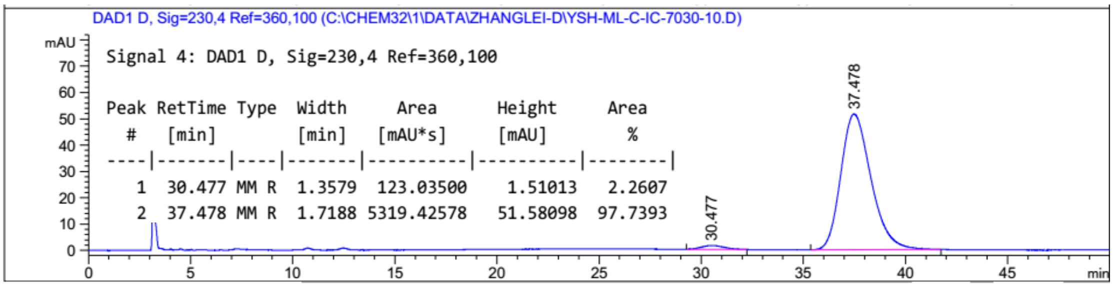


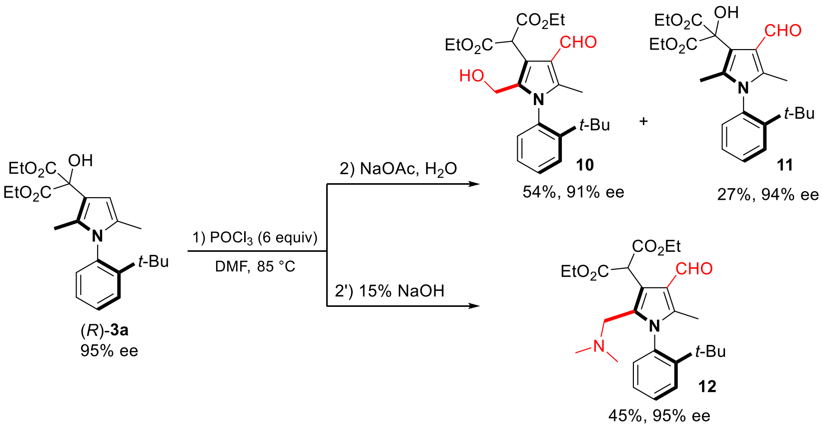


According to the literature,[9] under nitrogen atmosphere, $POCl_3$ (0.6 mmol, 6.0 equiv) was added dropwise to stirred ice-cooled DMF (1.0 mL) during 2 min and stirred at 0 ℃ for 30 min, then diluted by dry DCE (3.0 mL), followed by addition of a solution of (*R*)-**3a** (0.1 mmol, 1.0 equiv, 40.1 mg) in dry DCE (1.0 mL), then heated at 85 ℃ for 1 hours. After cooling a solution of NaOAc (0.4 mmol, 4.0

equiv) in $H_2O$ (1.0 mL) was added dropwise to the mixture, the mixture then heated at 85 ℃ for 1 hours. After cooling to room temperature, the reaction was quenched by addition of water and extracted with EtOAc. The organic phase was separated, washed with water, dried over anhydrous $Na_2SO_4$, filtered and concentrated. The residue was purified by a silica gel flash chromatography (Hexane/EtOAc) to give compound **10** and **11**.

Phosphorous oxychloride (0.6 mmol, 6.0 equiv) was added dropwise to stirred ice-cooled DMF (1.0 mL) under a $N_2$ atmosphere. The mixture was kept at 0 ℃ for 30 min and then a solution of the (*R*)-**3a** (0.1 mmol, 40.1 mg) in DMF (1.0 mL) was added and the mixture then heated at 85 ℃ for 3 hours. After cooling 30% NaOH was added dropwise to adjust to pH~10. Then, the mixture was extracted with EtOAc, the organic phase was separated, washed with water, dried over anhydrous $Na_2SO_4$, filtered and concentrated. The residue was purified by a silica gel flash chromatography (Hexane/EtOAc) to give **12** in 45% yield and 95% ee.

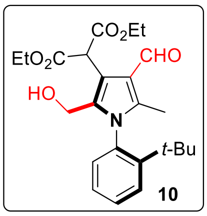


**Diethyl 2-(1-(2-(*tert*-butyl)phenyl)-4-formyl-2-(hydroxymethyl)-5-methyl-1*H*-pyrrol-3-yl)malonate (10)**

**$^{1}$H NMR (400 MHz, $CDCl_3$)** δ 9.99 (s, 1H), 7.63 (dd, *J* = 8.2, 1.4 Hz, 1H), 7.50-7.39 (m, 1H), 7.32-7.27 (m, 1H), 7.07 (dd, *J* = 7.8, 1.5 Hz, 1H), 6.15 (s, 1H), 4.36 (d, *J* = 13.5 Hz, 1H), 4.34-4.15 (m, 4H), 4.00 (d, *J* = 13.5 Hz, 1H), 3.51 (br, 1H), 2.20 (s, 3H), 1.31 (t, *J* = 7.1 Hz, 3H), 1.23 (t, *J* = 7.1 Hz, 3H), 1.12 (s, 9H).

**$^{13}$C NMR (100 MHz, $CDCl_3$)** δ = 185.9, 170.4, 169.4, 147.1, 141.9, 135.7, 132.8, 131.5, 130.0, 129.8, 127.1, 119.0, 113.0, 62.4, 61.9, 54.3, 47.9, 36.2, 31.6, 14.0, 11.1.

**$^{13}$C NMR-DEPT 135 (100 MHz, $CDCl_3$)** δ = 131.5, 130.0, 129.8, 127.1, 62.4, 61.9, 54.3, 47.9, 31.6, 14.0, 11.1.

**HRMS (ESI)** calcd for $[M+H]^+$ $C_{24}H_{32}NO_6^+$, m/z: 430.2224, found: 430.2214.

**HPLC analysis**: DAICEL CHIRALPAK ID, hexane/isopropyl alcohol = 70/30, flow rate = 1.0 mL/min, λ = 254 nm), $t_R$ (major) = 12.9 min, $t_R$ (minor) = 16.1 min, ee = 91%.

*Chiral HPLC spectrum of racemic **10***

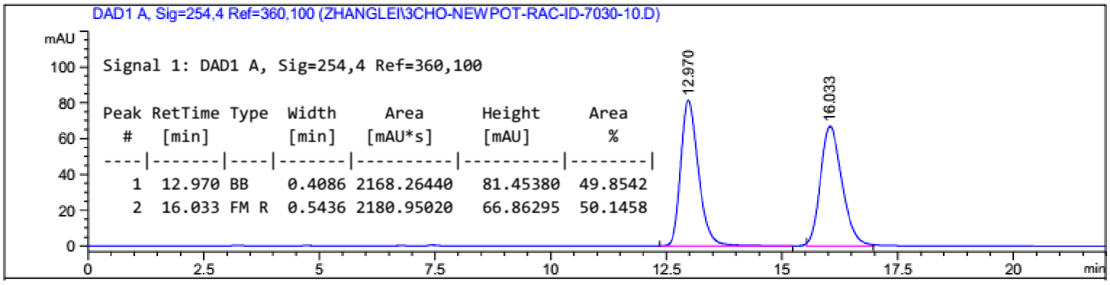


*Chiral HPLC spectrum of **10***

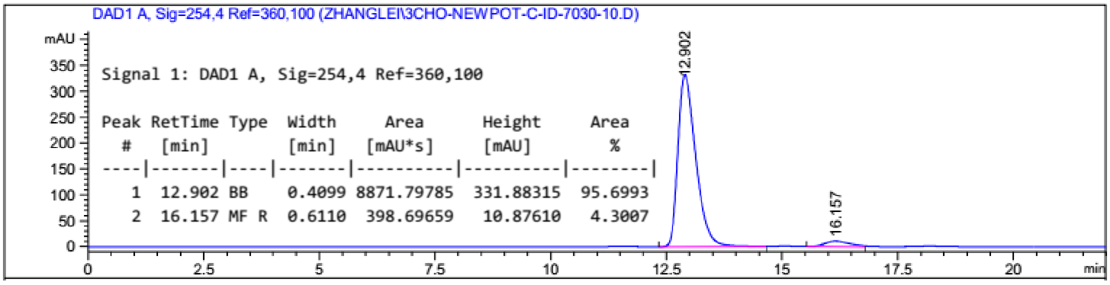


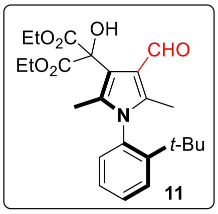


**Diethyl 2-(1-(2-(*tert*-butyl)phenyl)-4-formyl-2,5-dimethyl-1*H*-pyrrol-3-yl)-2-hydroxymalonate(11)**

**$^{1}$H NMR (400 MHz, CDCl$_3$)** δ 9.69 (s, 1H), 7.70 (s, 1H), 7.66 (dd, $J$ = 8.2, 1.3 Hz, 1H), 7.49-7.43 (m, 1H), 7.30 (td, $J$ = 7.6, 1.4 Hz, 1H), 6.89 (dd, $J$ = 7.8, 1.4 Hz, 1H), 4.33-4.18 (m, 4H), 2.22 (s, 3H), 1.84 (s, 3H), 1.34-1.26 (m, 6H), 1.16 (s, 9H).

**$^{13}$C NMR (100 MHz, CDCl$_3$)** δ = 185.6, 169.9, 169.9, 147.3, 144.2, 133.2, 131.0, 130.6, 130.5, 129.9, 127.4, 120.2, 118.2, 78.6, 62.3, 62.1, 36.2, 31.7, 14.0, 14.0, 11.8, 11.8.

**$^{13}$C NMR-DEPT 135 (100 MHz, CDCl$_3$)** δ = 130.6, 130.5, 129.9, 127.4, 62.3, 62.1, 31.7, 14.0, 14.0, 11.8, 11.8.

**HRMS (ESI)** calcd for $[M+H]^+$ $C_{24}H_{32}NO_6^+$, m/z: 430.2224, found: 430.2213.

**HPLC analysis**: DAICEL CHIRALPAK ID, hexane/isopropyl alcohol = 70/30, flow rate = 1.0 mL/min, λ = 300 nm), $t_R$ (minor) = 41.2 min, $t_R$ (major) = 54.3 min, ee = 94%.

*Chiral HPLC spectrum of racemic **11***

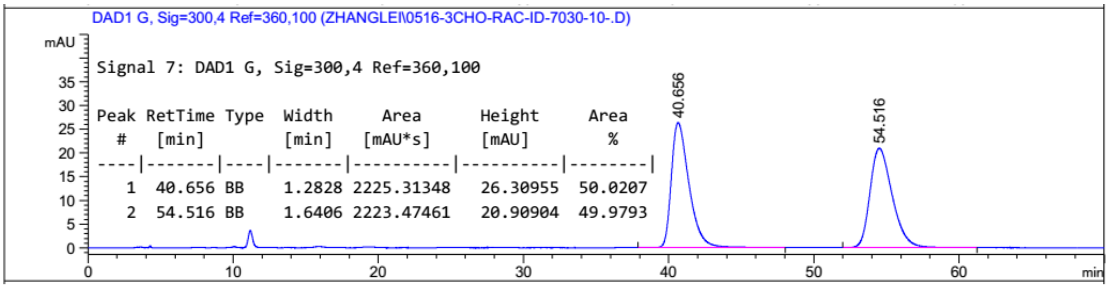


*Chiral HPLC spectrum of **11***

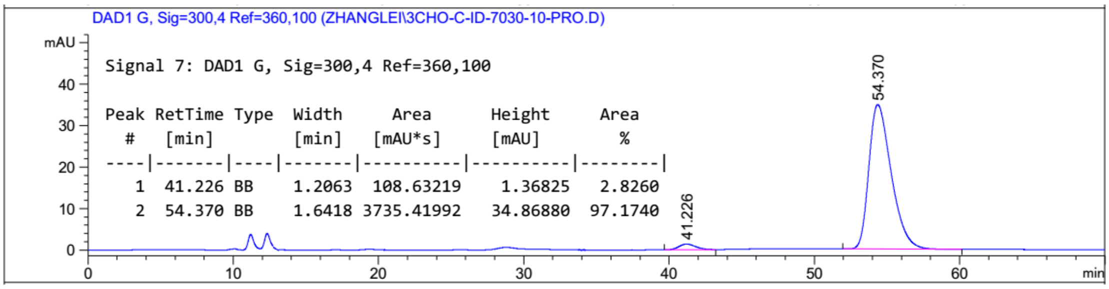

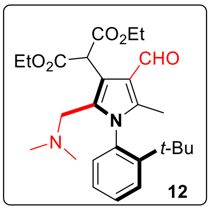


**Diethyl 2-(1-(2-(*tert*-butyl)phenyl)-2-((dimethylamino)methyl)-4-formyl-5-methyl-1H-pyrrol-3-yl)malonate (12)**

**$^1$H NMR (400 MHz, CDCl$_3$)** δ 9.97 (s, 1H), 7.61 (dd, $J$ = 8.2, 1.3 Hz, 1H), 7.47-7.38 (m, 1H), 7.26-7.22 (m, 1H), 6.92 (dd, $J$ = 7.8, 1.4 Hz, 1H), 5.42 (s, 1H), 4.30-4.13 (m, 4H), 3.36 (d, $J$ = 14.0 Hz, 1H), 2.76 (d, $J$ = 14.0 Hz, 1H), 2.23 (s, 3H), 1.99 (s, 6H), 1.29 (t, $J$ = 7.1 Hz, 3H), 1.22 (t, $J$ = 7.1 Hz, 3H), 1.11 (s, 9H).

**$^{13}$C NMR (100 MHz, CDCl$_3$)** δ = 186.6, 168.8, 168.7, 147.6, 139.9, 133.3, 131.5, 130.0, 129.4, 128.9, 126.5, 119.7, 114.9, 61.5, 61.4, 53.7, 48.9, 44.9, 36.2, 31.5, 14.2, 14.0, 12.3.

**$^{13}$C NMR-DEPT 135 (100 MHz, CDCl$_3$)** δ = 131.5, 130.0, 129.4, 126.5, 61.5, 61.4, 53.7, 48.9, 44.9, 31.5, 14.2, 14.0, 12.3.

**HRMS (ESI)** calcd for $[M+H]^+$ $C_{26}H_{37}N_2O_5^+$, m/z: 457.2697, found: 457.2686.

**HPLC analysis**: DAICEL CHIRALPAK ID, hexane/isopropyl alcohol = 90/10, flow rate = 1.0 mL/min, λ = 270 nm), $t_R$ (minor) = 27.7 min, $t_R$ (major) = 30.0 min, ee = 95%.

*Chiral HPLC spectrum of racemic **12***

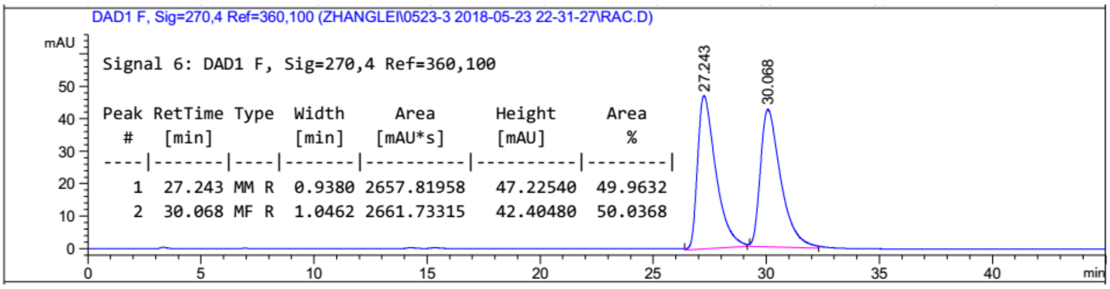


*Chiral HPLC spectrum of **12***

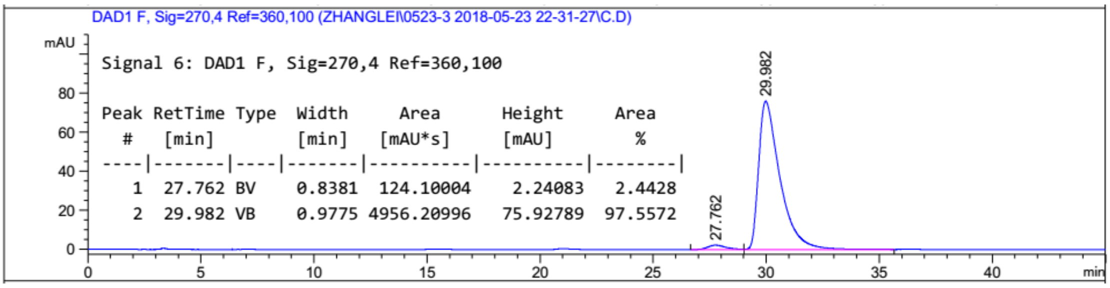


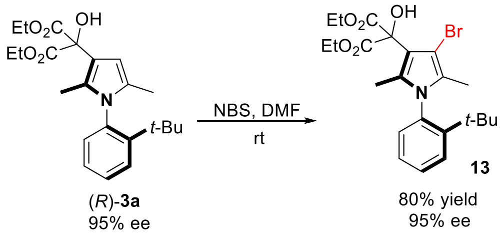

To a solution of (*R*)-**3a** (0.1 mmol, 1.0 equiv, 40.1 mg) in DMF (1.0 mL) was added NBS (0.12 mmol, 1.2 equiv, 21.6 mg) at room temperature and the resulting mixture was stirred at room temperature until the reaction was completed (monitored by TLC). The reaction mixture was added water (6.0 mL) and extracted with EtOAc. The organic phase was separated, washed with water and brine, dried over anhydrous $Na_2SO_4$, filtered and concentrated. The residue was purified by a silica gel flash chromatography (Hexane/EtOAc) to give compound **13** with 80% yield and 95% ee.

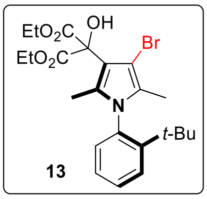


**Diethyl 2-(4-bromo-1-(2-(*tert*-butyl)phenyl)-2,5-dimethyl-1*H*-pyrrol-3-yl)-2-hydroxymalonate (13)**
**$^1$H NMR (400 MHz, $CDCl_3$)** δ 7.59 (dd, *J* = 8.2, 1.4 Hz, 1H), 7.43-7.34 (m, 1H), 7.23 (td, *J* = 7.6, 1.4 Hz, 1H), 6.91 (dd, *J* = 7.8, 1.4 Hz, 1H), 4.41-4.21 (m, 4H), 4.17 (s, 1H), 1.90 (s, 3H), 1.86 (s, 3H), 1.33-1.28 (m, 6H), 1.12 (s, 9H).
**$^{13}$C NMR (100 MHz, $CDCl_3$)** δ = 170.6, 170.5, 147.7, 134.9, 131.4, 129.9, 129.1, 128.4, 127.7, 126.9, 113.9, 94.9, 78.1, 62.9, 62.8, 36.1, 31.6, 13.9, 12.5, 12.2.
**$^{13}$C NMR-DEPT 135 (100 MHz, $CDCl_3$)** δ = 131.4, 129.9, 129.1, 126.9, 62.9, 62.8, 31.6, 13.9, 12.5, 12.2.
**HRMS (ESI)** calcd for $[M+H]^+$ $C_{23}H_{31}BrNO_5^+$, m/z: 480.1380, found: 480.1386.
**HPLC analysis**: DAICEL CHIRALCEL OD-H, hexane/isopropyl alcohol = 95/05, flow rate = 1.0 mL/min, λ = 270 nm), $t_R$ (minor) = 13.6 min, $t_R$ (major) = 17.2 min, ee = 95%.

*Chiral HPLC spectrum of racemic **13***

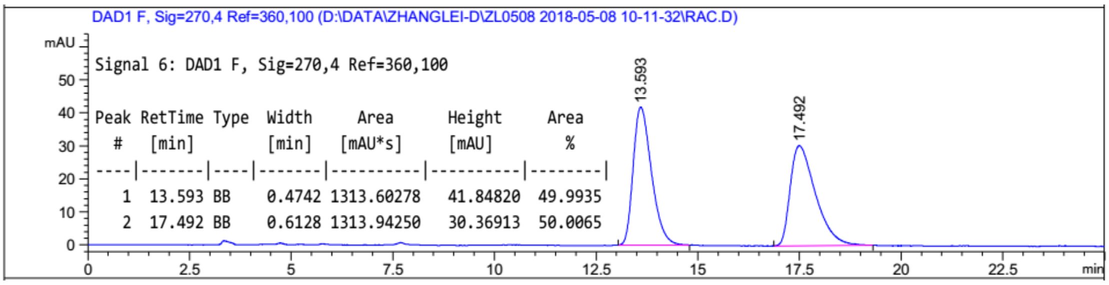


*Chiral HPLC spectrum of **13***

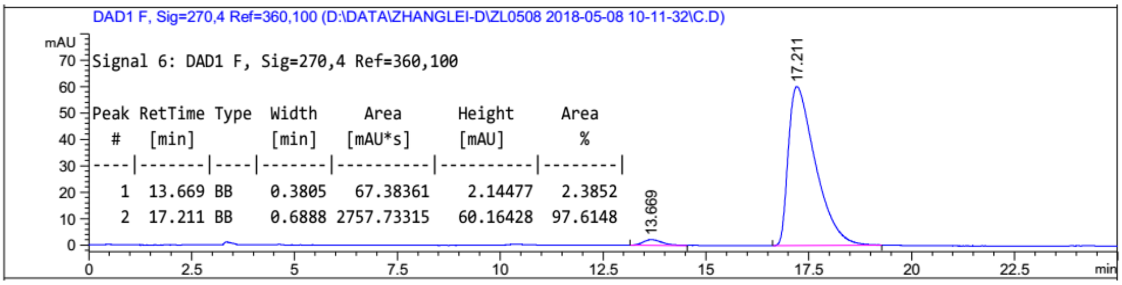

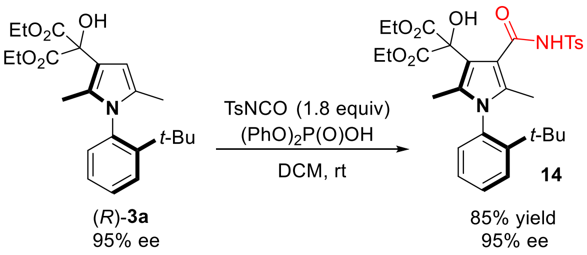


To a solution of (*R*)-**3a** (0.1 mmol, 1.0 equiv, 40.1 mg) in DCM (2.0 mL) was added tosyl isocyanate (0.18 mmol, 1.8 equiv, 36.0 mg) and diphenyl phosphate (0.015 mmol, 3.8 mg) at rt and the mixture was stirred at room temperature for 48 hours**,** then the mixture was concentrated *in vacuo* and chromatographed directly on silica gel to afford **14** with 85% yield and 95% ee.

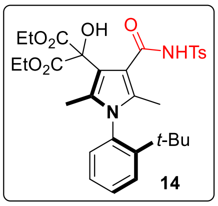


**Diethyl 2-(1-(2-(*tert*-butyl)phenyl)-2,5-dimethyl-4-(tosylcarbamoyl)-1*H*-pyrrol-3-yl)-2-hydroxymalonate (14)**

**$^{1}$H NMR (400 MHz, $CDCl_3$)** δ 9.97 (s, 1H), 8.07 (s, 1H), 8.05 (s, 1H), 7.63 (dd, *J* = 8.2, 1.1 Hz, 1H), 7.48-7.39 (m, 1H), 7.34 (d, *J* = 8.1 Hz, 2H), 7.26 (dd, *J* = 7.4, 1.1 Hz, 1H), 6.88 (dd, *J* = 7.8, 1.3 Hz, 1H), 4.86 (s, 1H), 4.33-4.11 (m, 3H), 4.05 (d, *J* = 7.2 Hz, 1H), 2.44 (s, 3H), 2.09 (s, 3H), 1.78 (s, 3H), 1.25 (t, *J* = 7.1 Hz, 3H), 1.19 (t, *J* = 7.1 Hz, 3H), 1.11 (s, 9H).

**$^{13}$C NMR (100 MHz, $CDCl_3$)** δ = 169.9, 169.9, 162.8, 147.6, 144.2, 136.8, 136.7, 133.6, 130.9, 130.3, 129.6, 129.2, 129.0, 128.8, 127.1, 113.6, 112.9, 77.8, 63.3, 63.2, 36.1, 31.6, 21.6, 13.7, 13.0, 11.9.

**$^{13}$C NMR-DEPT 135 (100 MHz, $CDCl_3$)** δ = 130.9, 130.3, 129.6, 129.2, 128.8, 127.1, 63.3, 63.2, 31.6, 21.6, 13.7, 13.0, 11.9.

**HRMS (ESI)** calcd for $[M-H]^-$ $C_{31}H_{37}N_2O_8S^-$, m/z: 597.2276, found: 597.2279.

**HPLC analysis**: DAICEL CHIRALPAK IA, hexane/isopropyl alcohol/TFA = 90/10/0.1, flow rate = 1.0 mL/min, λ = 270 nm), $t_R$ (minor) = 29.8 min, $t_R$ (major) = 41.0 min, ee = 95%.

*Chiral HPLC spectrum of racemic* ***14***

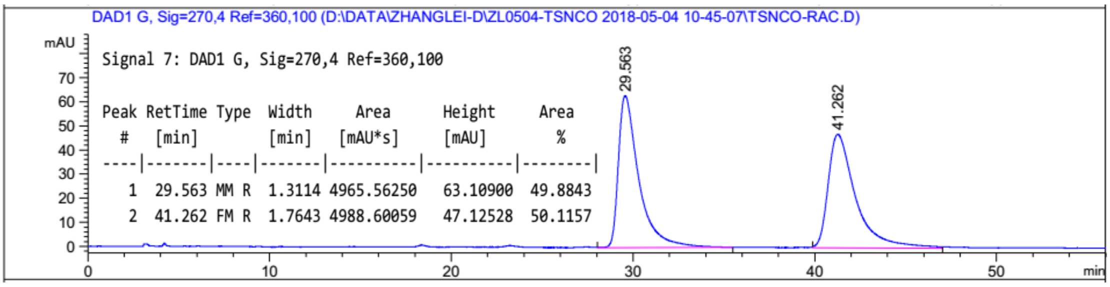


*Chiral HPLC spectrum of* ***14***

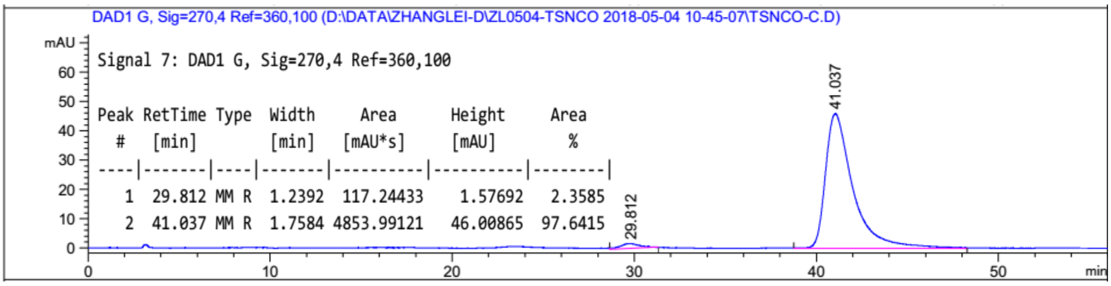


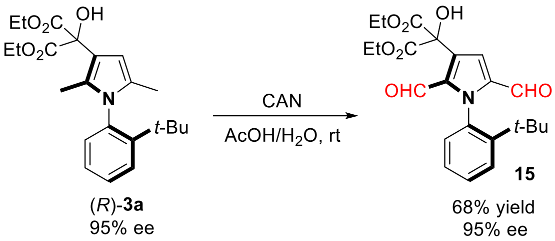


Compound **15** was prepared according to the literature[10]. (*R*)-**3a (**0.1 mmol, 1.0 equiv, 40.1 mg**)** was dissolved in THF (1.6 mL), HOAc (0.4 mL), and $H_2O$ (0.4 mL). Then 8.6 equiv of ceric ammonium nitrate (472 mg, 0.86 mmol) was added to the mixture all in once. The reaction mixture was stirred at room temperature for 17h until the reaction was complete (monitored by TLC). Then, the mixture was poured into 5.0 mL of water and extracted with dichloromethane (3×5.0 mL). The organic layer was washed with water (10 mL) three times followed by saturated aqueous $NaHCO_3$ (10 mL). Then the organic extracts were combined and dried over anhydrous $Na_2SO_4$. Finally the solution was concentrated under vacuum to remove the solvents. The residue was purified by a silica gel flash chromatography to give **15** with 68% yield and 95% ee.

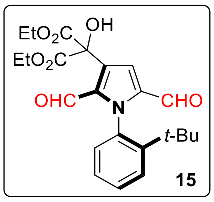


**Diethyl 2-(1-(2-(*tert*-butyl)phenyl)-2,5-diformyl-1*H*-pyrrol-3-yl)-2-hydroxymalonate (15)**

**$^1$H NMR (500 MHz, CDCl$_3$)** δ 9.48 (s, 1H), 9.42 (s, 1H), 7.68 (d, *J* = 8.1 Hz, 1H), 7.56-7.49 (m, 1H), 7.35-7.28 (m, 1H), 7.18 (s, 1H), 7.09 (dd, *J* = 7.7, 0.9 Hz, 1H), 5.49 (s, 1H), 4.41-4.29 (m, 4H), 1.33 (t, *J* = 7.1 Hz, 3H), 1.29 (t, *J* = 7.1 Hz, 3H), 1.15 (s, 9H).

**$^{13}$C NMR (125 MHz, CDCl$_3$)** δ = 182.7, 180.7, 168.6, 168.5, 147.1, 135.7, 134.1, 132.7, 130.6, 130.6, 130.3, 129.5, 127.3, 117.6, 77.7, 63.1, 63.0, 36.3, 31.5, 14.0, 14.0.

**$^{13}$C NMR-DEPT 135 (100 MHz, CDCl$_3$)** δ = 130.6, 130.6, 129.5, 127.3, 117.6, 63.1, 63.0, 31.5, 14.0, 14.0.

**HRMS (ESI)** calcd for $[M+H]^+$ $C_{23}H_{28}NO_7^+$, m/z: 430.1860, found: 430.1855.

**HPLC analysis**: DAICEL CHIRALPAK IA, hexane/isopropyl alcohol = 92/08, flow rate = 1.0 mL/min, λ = 300 nm), $t_R$ (major) = 29.0 min, $t_R$ (minor) = 32.5 min, ee = 95%.

*Chiral HPLC spectrum of racemic **15***

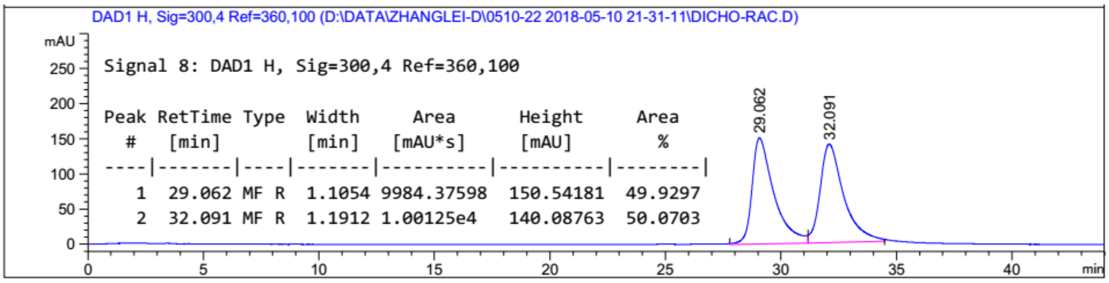


*Chiral HPLC spectrum of* ***15***

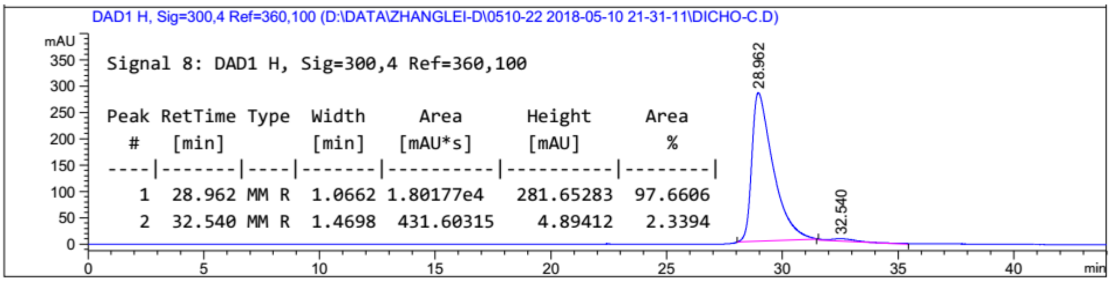


## Versatile transformations from the compound (*R*)-3h

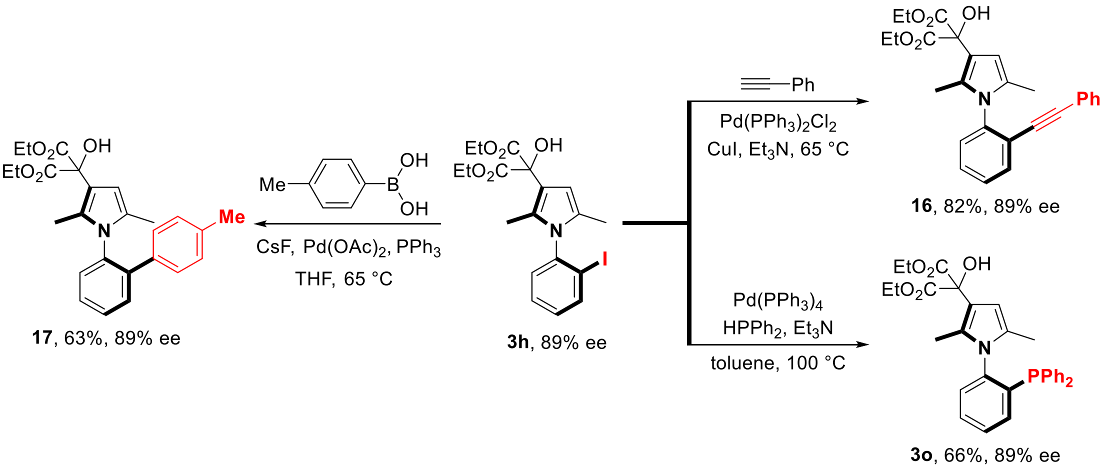


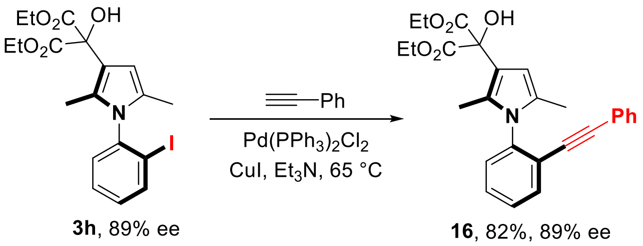


Under nitrogen atmosphere, $Pd(PPh_3)_2Cl_2$ (7.0 mg, 0.01 mmol) and CuI (1.9 mg, 0.01 mmol) were dissolved in $Et_3N$ (1.0 mL), then **3h** (47.1 mg, 0.1 mmol) and phenylacetylene (15.3 mg, 0.15 mmol) were added to the solution. The resulting mixture was stirred at 65 ℃ for 12 h and quenched with saturated aqueous $NH_4Cl$, then extracted with $CH_2Cl_2$ (2 × 3 mL). The combined organic layers were dried with anhydrous $Na_2SO_4$, filtered and concentrated. The residue was purified by a silica gel flash chromatography (Hexane/EtOAc) to give compound **16**.

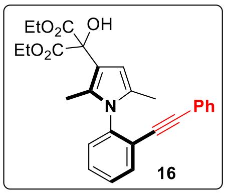


**Diethyl 2-(2,5-dimethyl-1-(2-(phenylethynyl)phenyl)-1H-pyrrol-3-yl)-2-hydroxymalonate (16)**

**$^1$H NMR (400 MHz, CDCl$_3$)** δ 7.66-7.64 (m, 1H), 7.47-7.42 (m, 2H), 7.34-7.29 (m, 6H), 6.06 (s, 1H), 4.32-4.17 (m, 4H), 4.08 (s, 1H), 1.99 (s, 6H), 1.29-1.22 (m, 6H).

**$^{13}$C NMR (100 MHz, CDCl$_3$)** δ = 170.9, 170.8, 140.6, 132.1, 131.9, 129.2, 128.9, 128.5, 128.4, 128.2, 127.7, 127.5, 124.2, 122.7, 114.9, 105.6, 94.1, 85.1, 78.3, 62.5, 14.0, 12.6, 11.7.

**$^{13}$C NMR-DEPT 135 (100 MHz, CDCl$_3$)** δ = 132.1, 131.9, 129.2, 128.9, 128.5, 128.4, 128.2, 105.6, 62.5, 14.0, 12.6, 11.7.

**HRMS (ESI)** calcd for $[M+H]^+$ $C_{27}H_{27}NNaO_5^+$, m/z: 468.1781, found: 468.1786.

**HPLC analysis**: DAICEL CHIRALPAK AD-H, hexane/isopropyl alcohol = 95/05, flow rate = 1.0 mL/min, λ = 300 nm), $t_R$ (major) = 24.1 min, $t_R$ (minor) = 29.9 min, ee = 89%.

*Chiral HPLC spectrum of racemic **16***

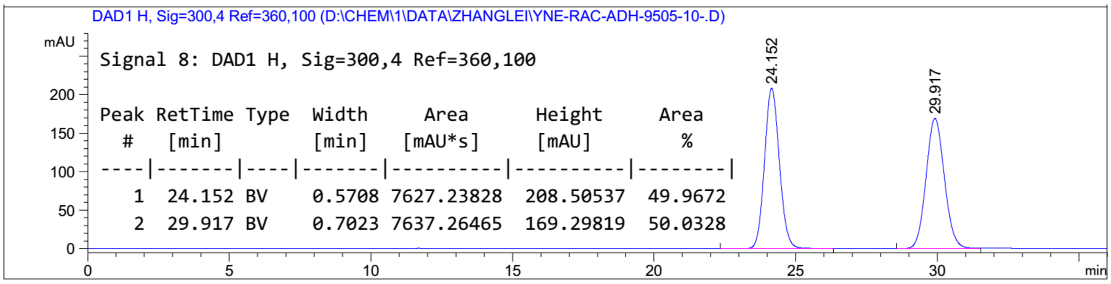


*Chiral HPLC spectrum of **16***

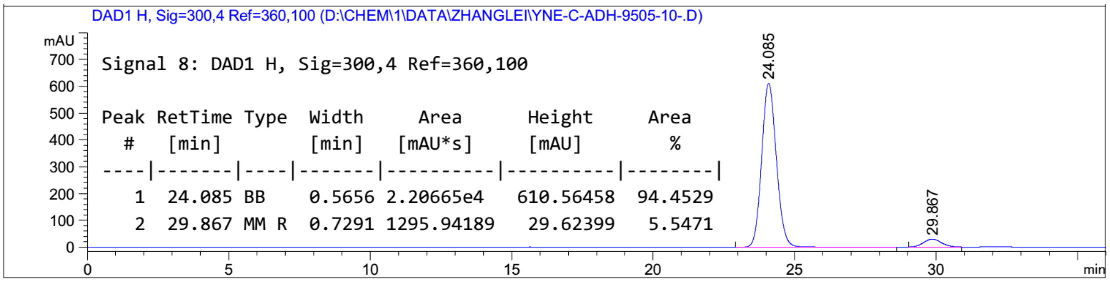


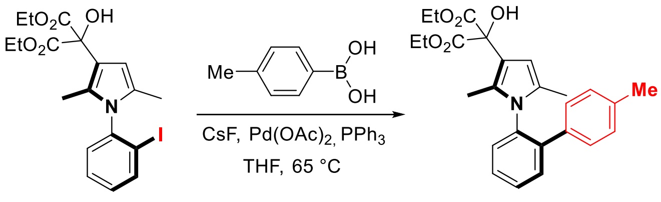


**3h**, 89% ee **17**, 63%, 89% ee

To an oven-dried 10 mL of Schlenk tube was added **3h** (47.1 mg, 0.10 mmol), 4-tolylboronic acid (27.2 mg, 0.20 mmol), Pd(OAc)$_2$ (3.38 mg, 15 mol %), PPh$_3$ (7.9 mg, 30 mol %), CsF (60.0 mg, 4.0 equiv), and THF (1.0 mL). The mixture was stirred at 65 ℃ in an oil bath under a nitrogen atmosphere for 72 h. The completion of the reaction was monitored by **TLC)**. The reaction mixture was cooled and purified

directly by column chromatography on a silica gel column. to afford the corresponding **17** with 63% yield and 89% ee.

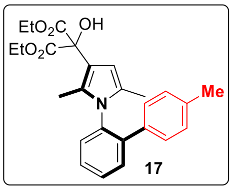


**Diethyl 2-(2,5-dimethyl-1-(4'-methyl-[1,1'-biphenyl]-2-yl)-1H-pyrrol-3-yl)-2-hydroxymalonate (17)**

**$^1$H NMR (400 MHz, $CDCl_3$)** δ 7.57 (dd, *J* = 7.7, 1.6 Hz, 1H), 7.50 (td, *J* = 7.5, 1.4 Hz, 1H), 7.43 (td, *J* = 7.5, 1.6 Hz, 1H), 7.27 (dd, *J* = 7.7, 1.4 Hz, 1H), 7.06 (d, *J* = 7.9 Hz, 2H), 6.92-6.90 (m, 2H), 5.93 (d, *J* = 0.7 Hz, 1H), 4.38-4.20 (m, 4H), 3.97 (s, 1H), 2.32 (s, 3H), 1.85 (s, 3H), 1.84 (s, 3H), 1.30 (td, *J* = 7.1, 2.5 Hz, 6H).

**$^{13}$C NMR (100 MHz, $CDCl_3$)** δ = 170.8, 170.7, 140.2, 136.9, 135.6, 135.5, 130.8, 129.9, 129.0, 128.8, 128.1, 127.8, 127.2, 127.0, 115.3, 105.8, 78.0, 62.4, 62.4, 21.1, 14.0, 14.0, 12.8, 11.9.

**HPLC analysis**: DAICEL CHIRALPAK ID, hexane/isopropyl alcohol = 90/10, flow rate = 1.0 mL/min, λ = 254 nm), $t_R$ (major) = 24.8 min, $t_R$ (minor) = 27.8 min, ee = 89%.

*Chiral HPLC spectrum of racemic* ***17***

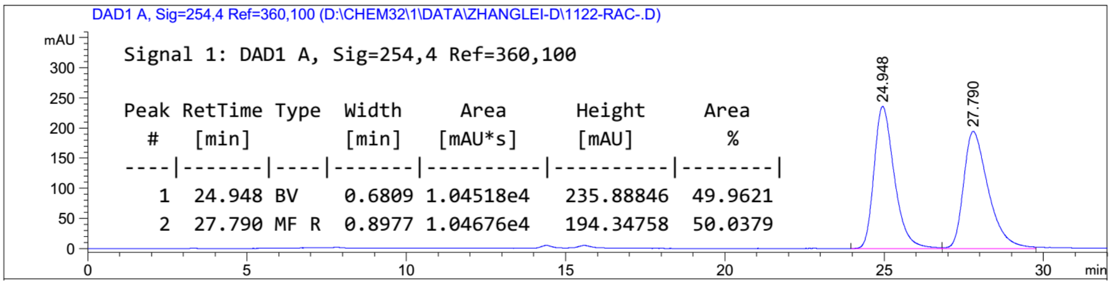


*Chiral HPLC spectrum of* ***17***

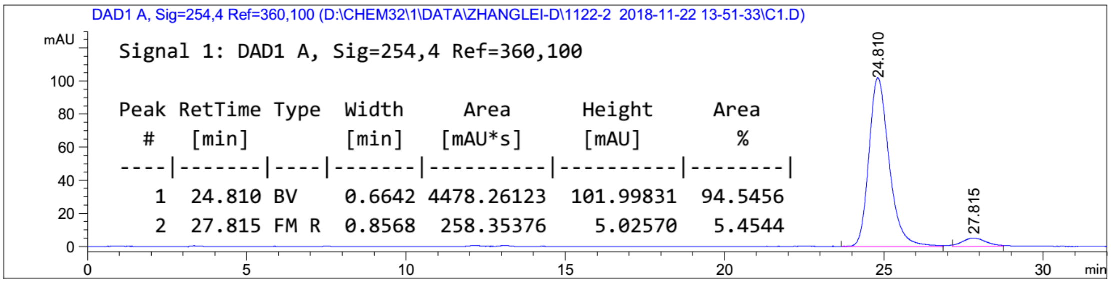


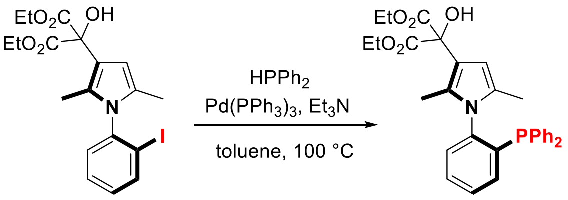


**3h**, 89% ee　　**3o**, 66%, 89% ee

According to the literature,[11] an oven-dried 10 mL of Schlenk tube was charged with (*R*)-**3h** (47.1 mg,

0.10 mmol), 23.0 mg (20 mol%) of $Pd(Ph_3P)_4$, 30.3 mg (**0.30 mmol,3.0** equiv) of $NEt_3$, 41.0 mg (0.22 mmol, 2.2 equiv) of $Ph_2PH$, 1.0 mL of dry toluene, and the mixture was stirred under a nitrogen atmosphere at 100 ℃ for 60 h. The reaction mixture was cooled and concentrated under reduced pressure, then purified directly by flash chromatography eluted with PE/EA to afford the corresponding **3o** with 66% yield and 89% ee.

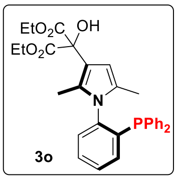


***(R)*-Diethyl 2-(1-(2-(diphenylphosphanyl)phenyl)-2,5-dimethyl-1*H*-pyrrol-3-yl)-2-hydroxymalonate ((*R*)-3o)**

**$^{1}$H NMR (400 MHz, $CDCl_3$)** δ 7.45-7.41 (m, 1H), 7.39-7.35 (m, 1H), 7.31-7.28 (m, 6H), 7.26-7.15 (m, 6H), 5.95 (s, 1H), 4.40-4.23 (m, 4H), 3.89 (s, 1H), 1.74 (s, 3H), 1.64 (s, 3H), 1.34-1.27 (m, 6H).

**$^{13}$C NMR (100 MHz, $CDCl_3$)** δ = 170.87, 170.54, 142.8 (d, *J* = 25.9 Hz), 139.4 (d, *J* = 16.2 Hz), 136.2 (d, *J* = 12.1 Hz), 135.8 (d, *J* = 12.4 Hz), 134.6 (d, *J* = 1.7 Hz), 134.2, 134.0, 134.0, 133.8, 130.0, 129.6 (d, *J* = 2.9 Hz), 128.9, 128.8, 128.7, 128.5, 128.4 (d, *J* = 1.2 Hz), 128.4, 127.5, 127.3, 115.0, 105.8, 78.3, 62.5, 62.4, 14.2, 14.1, 12.8 (d, *J* = 5.0 Hz), 11.9 (d, *J* = 4.4 Hz).

**$^{31}$P NMR (162 MHz, $CDCl_3$)** δ = -16.64.

**HRMS (ESI)** calcd for $[M+H]^+$ $C_{31}H_{33}NO_5P^+$, m/z: 530.2091, found: 530.2089.

**HPLC analysis**: DAICEL CHIRALPAK IG, hexane/isopropyl alcohol = 95/05, flow rate = 1.0 mL/min, λ = 254 nm), $t_R$ (minor) = 49.0 min, $t_R$ (major) = 56.7 min, ee = 89%.

*Chiral HPLC spectrum of racemic **3o***

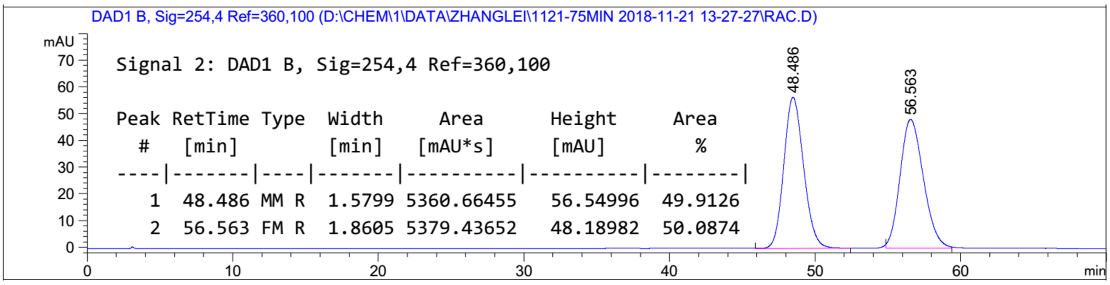


*Chiral HPLC spectrum of (R)-**3o***

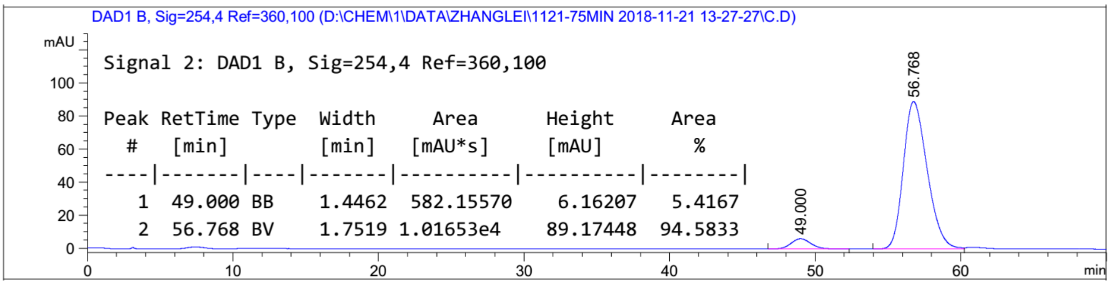

# Supplementary Note 5

## Application in asymmetric catalysis.

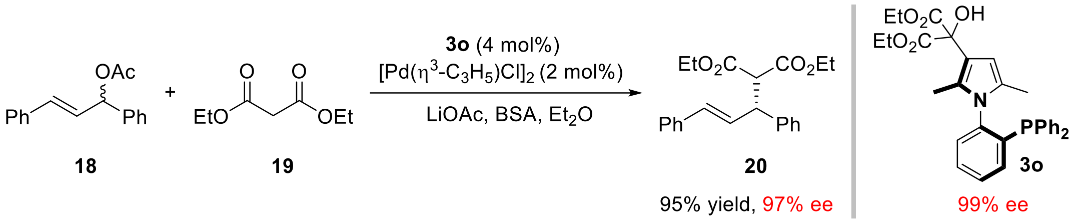


To a mixture of chiral ligand **3o** (0.02 mmol, 10.6 mg), [Pd(η3-$C_3H_5$)Cl]$_2$ (0.01 mmol, 3.7 mg) and LiOAc (0.02 mmol, 1.3 mg) in diethyl ether (1.5 mL) were added BSA (1.5 mmol, 322.0 mg) and allylic ester **18** (0.5 mmol, 126.2 mg) at room temperature under an Ar atmosphere. After 30 min, malonate **19** (1.5 mmol, 240.3 mg) was added. After 12 h, the reaction mixture was diluted with diethyl ether and water. The organic layer was washed with brine and dried over $Na_2SO_4$. The filtrate was concentrated *in vacuo* and purified by column chromatography to afford **20** with 95% yield and 97% ee (The absolute configuration was determined by contrasting with the previous study[12]).

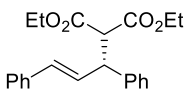


**20**

**Diethyl (*R*,*E*)-2-(1,3-diphenylallyl)malonate (20)**

**$^1$H NMR (400 MHz, CDCl$_3$)** δ 7.35-7.14 (m, 10H), 6.47 (d, *J* = 15.8 Hz, 1H), 6.34 (dd, *J* = 15.8, 8.4 Hz, 1H), 4.26 (dd, *J* = 11.0, 8.4 Hz, 1H), 4.17 (q, *J* = 7.1 Hz, 2H), 4.00-3.96 (m, 2H), 3.92 (d, *J* = 11.0 Hz, 1H), 1.20 (t, *J* = 7.1 Hz, 3H), 1.00 (t, *J* = 7.1 Hz, 3H).

**$^{13}$C NMR (100 MHz, CDCl$_3$)** δ = 167.9, 167.5, 140.3, 136.9, 131.7, 129.4, 128.7, 128.5, 128.0, 127.6, 127.1, 126.4, 61.6, 61.4, 57.8, 49.3, 14.2, 13.8.

**HRMS (ESI)** calcd for $[M+Na]^+$ $C_{22}H_{24}NaO_4^+$, m/z: 375.1567, found: 375.1565.

**HPLC analysis**: DAICEL CHIRALPAK AD-H, hexane/isopropyl alcohol = 93/07, flow rate = 1.0 mL/min, λ = 254 nm), $t_R$ (major) = 12.2 min, $t_R$ (minor) = 16.6 min, ee = 97%.

*Chiral HPLC spectrum of racemic **20***

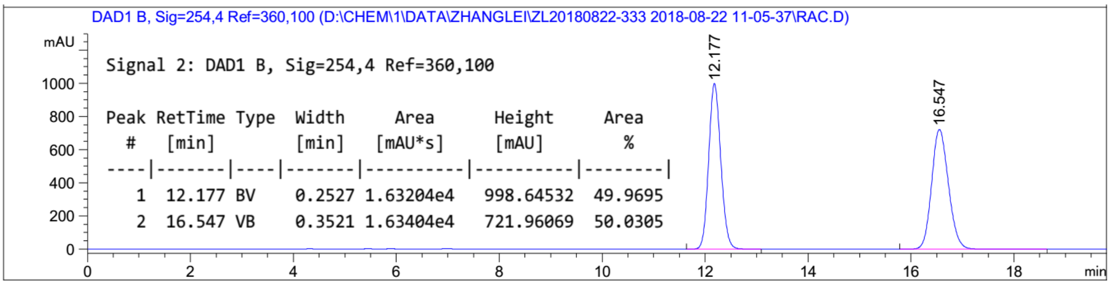


*Chiral HPLC spectrum of (R)-**20***

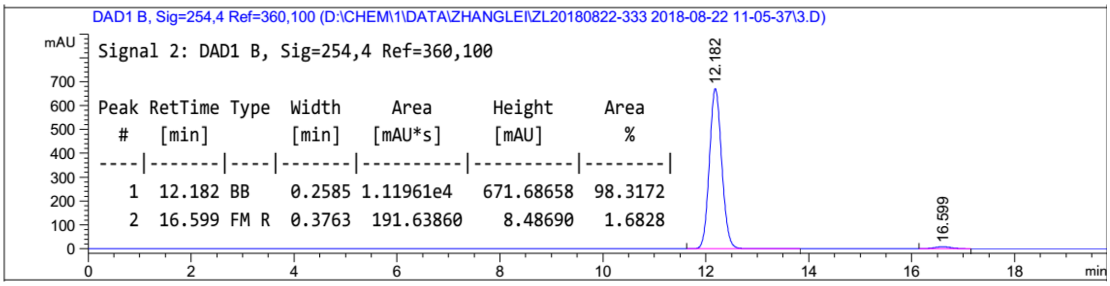


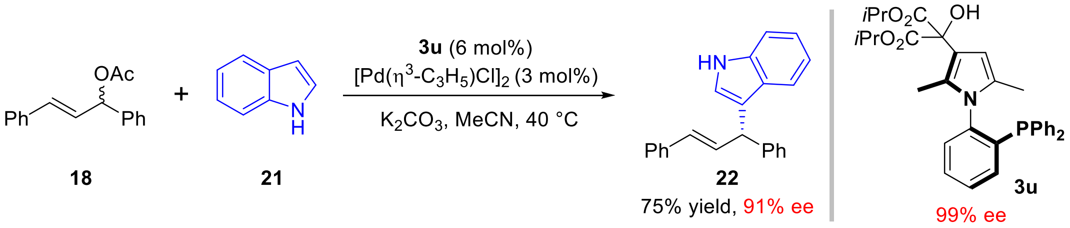


To a mixture of indole (0.2 mmol, 23.44 mg), allylic ester **18** (0.24 mmol, 62.5 mg), chiral ligand **3u** (6.68 mg, 12 μmol), $[Pd(\eta^3\text{-}C_3H_5)Cl]_2$ (2.2 mg, 6 μmol), and $K_2CO_3$ (55.7 mg, 0.4 mol) was added MeCN (0.2 mL) at room temperature under an Ar atmosphere. After stirring for 24 h at 40 ℃, the mixture was quenched with water and diluted with diethyl ether. The organic layer was washed with brine and dried over $Na_2SO_4$. The filtrate was concentrated *in vacuo* and purified by column chromatography (elution with n-hexane/EtOAc/Et3N = 40/2/1) to afford **22** with 75% yield and 91% ee (The absolute configuration was determined by contrasting with the previous study[13]).

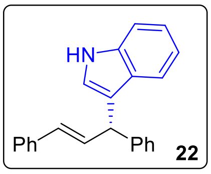


**(*S*,*E*)-3-(1,3-Diphenylallyl)-1*H*-indole**

**$^1$H NMR (400 MHz, CDCl$_3$)** δ 7.93 (s, 1H), 7.54 (d, *J* = 7.9 Hz, 1H), 7.49-7.25 (m, 12H), 7.13 (m, 1H), 6.93 (s, 1H), 6.83 (dd, *J* = 15.8, 7.4 Hz, 1H), 6.54 (d, *J* = 15.8 Hz, 1H), 5.22 (d, *J* = 7.4 Hz, 1H).

**$^{13}$C NMR (100 MHz, CDCl$_3$)** δ = 143.5, 137.6, 136.7, 132.6, 130.6, 128.6, 128.5, 127.3, 126.9, 126.5, 126.4, 122.7, 122.2, 119.9, 119.5, 118.7, 111.2, 46.3.

**HPLC analysis**: DAICEL CHIRALPAK AD-H, hexane/isopropyl alcohol = 90/10, flow rate = 1.0 mL/min, λ = 254 nm), $t_R$ (minor) = 17.0 min, $t_R$ (major) = 19.0 min, ee = 91%.

*Chiral HPLC spectrum of racemic **22***

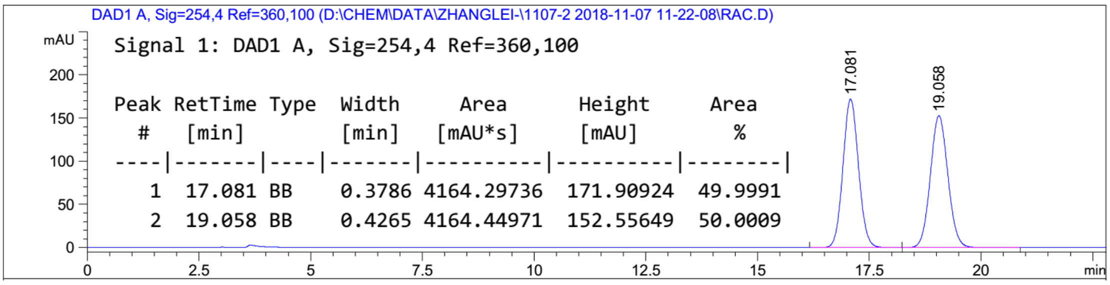


*Chiral HPLC spectrum of (S)-**22***

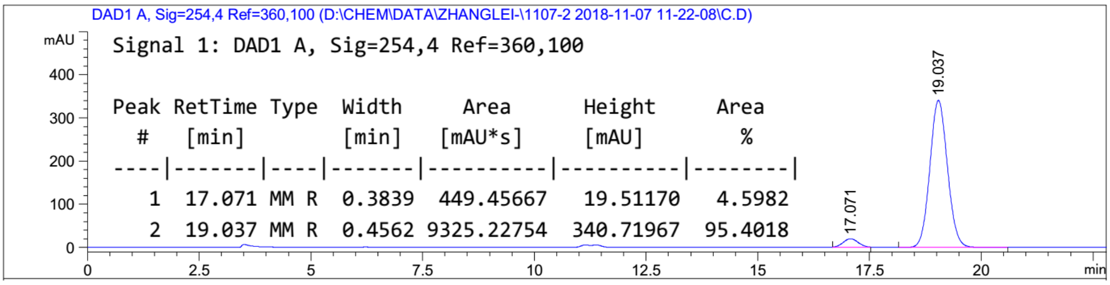


Signal 1: DAD1 A, Sig=254,4 Ref=360,100

| Peak # | RetTime [min] | Type | Width [min] | Area [mAU*s] | Height [mAU] | Area % |
|---|---|---|---|---|---|---|
| 1 | 17.071 | MM R | 0.3839 | 449.45667 | 19.51170 | 4.5982 |
| 2 | 19.037 | MM R | 0.4562 | 9325.22754 | 340.71967 | 95.4018 |

# Supplementary Note 6

## Investigation of the Reaction Mechanism

To give more mechanistic insights for this transformation, a series of control experiments were carried out. The reactions between **1a** and **2a** in *c*-hexane at room temperature for 24 hours gave the desired **3a** in more than 50% yields, indicating the strong background reaction for this transformation. Meanwhile, the reactions between **4a** and **2a** could also proceed in the absence of acidic catalyst, albeit lower yields. These results clearly illustrated C3 of the pyrrole is an applicable nucleophile to attack the ketomalonate. Based on the reported literatures and the above results, we anticipated that the **CPA** interacts with ketomalonate via double H-bond and enhance the electrophilicity of the ketone of ketomalonate. However, it is hard to predict the possibility of the interaction between **CPA** and H3 of the pyrrole with these initial results at this stage.

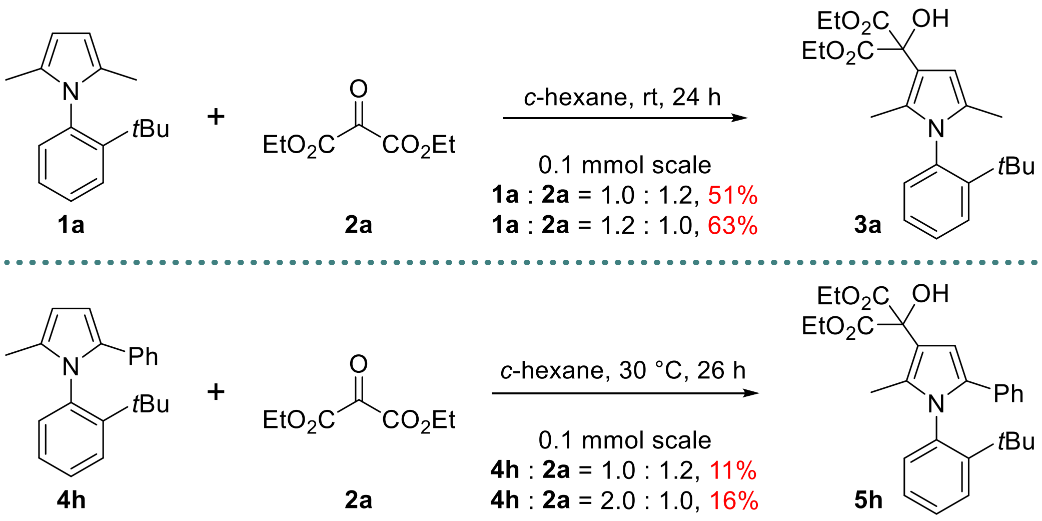


To test the probability of the interaction between **CPA** and H3 of the pyrrole, a series of NMR monitoring experiments were performed. First, a solution of (*S*)-**C8** (18.24 mg, 1.20 equiv) and **1a** (4.54 mg, 0.02 mmol) was stirred in $CDCl_3$ (1.0 mL) at rt for 12 hour. As depicted in **Supplementary Figure 3**, identical chemical shifts were observed (5.87 ppm) for H3 and H4 of pyrrole even in the presence of excess **CPA** (1.20 equiv). Meanwhile, a variation of the chemical shift (-0.14 ppm) was recorded as compared to the $^{31}$P NMR of **CPA** as shown in **Supplementary Figure 4**. Subsequently, the NMR monitoring experiments were performed in *c*-hexane-$d_{12}$ (the developed reaction solvent is *c*-hexane) and identical chemical shift was detected too. Similarly, a variation of the chemical shift (+0.20 ppm) was recorded as compared to the $^{31}$P NMR of CPA as shown in **Supplementary Figure 6** with *c*-hexane-$d_{12}$ as the solvent.

As shown in **Supplementary Figures 3-6**, no variation of the chemical shift was detected for H3 and H4 of the pyrrole by the $^{1}$H NMR spectra analysis in both $CDCl_3$ and *c*-hexane-$d_{12}$ even in the presence of excess **CPA**. On the other hand, inconspicuous variations of the chemical shift (-0.14 and +0.20 ppm) were observed for the **CPA** by the $^{31}$P NMR spectra analysis with both $CDCl_3$ and *c*-hexane-$d_{12}$ as the solvent. The above results demonstrated the possible weak interaction between **CPA** and *N* atom of the pyrrole and then ruled out the possibility of the interaction between **CPA** and H3.

Finally, to verify the interaction between **CPA** and the other substrate ketomalonate, a solution (*S*)-**C8**

(7.6 mg, 10 mol%) and **2a** (0.10 mmol) was stirred in $CDCl_3$ (1.0 mL) at rt for 12 hours. A variation of the chemical shift (-0.36 ppm) was recorded by $^{31}P$ NMR spectra analysis in **Supplementary Figure 7**. Meanwhile, -0.61 ppm of the chemical shift was observed in *c*-hexane-$d_{12}$ as shown in **Supplementary Figure 8**.

Overall, the more obvious variations of the chemical shifts clearly demonstrated that the interaction between **CPA** and ketomalonate should be much stronger than **CPA** and pyrrole (-0.14 vs -0.36 ppm in $CDCl_3$ and 0.2 vs -0.61 ppm in *c*-hexane-$d_{12}$). The observed identical chemical shift for H3 of pyrrole in both $CDCl_3$ and *c*-hexane-$d_{12}$ ruled out the interaction between **CPA** and H3 at the beginning of this reaction.

# Supplementary References


1. Kitanosono, T., Tani, S. & Kobayashi, S. Oxyfunctionalization of active methylene compounds using sodium chlorite in water. *Asian J. Org. Chem.* **7**, 350-354 (2018).
2. Kattamuri, P. V., Yin, J., Siriwongsup, S. & Sutton, S. C. Practical singly and doubly electrophilic aminating agents: a new, more sustainable platform for carbon–nitrogen bond formation. *J. Am. Chem. Soc.* **139**, 11184-11196 (2017).
3. Biava, M., Porretta, G. C., Poce, G. & Botta, M. 1,5-Diphenylpyrrole derivatives as antimycobacterial agents. Probing the influence on antimycobacterial activity of lipophilic substituents at the phenyl rings. *J. Med. Chem.* **51**, 3644-3648 (2008).
4. Biava, M., Porretta, G. C., Poce, G. & Botta, M. Identification of a novel pyrrole derivative endowed with antimycobacterial activity and protection index comparable to that of the current antitubercular drugs streptomycin and rifampin. *Bioorg. Med. Chem.* **18**, 8076-8084 (2010).
5. Guanti, G., Banfi, L., Powles, K. & Fossati, N. Asymmetric synthesis of (*R*)-(−)-chlozolinate through a chemoenzymatic procedure. *Tetrahedron: Asymmetry* **12**, 271-277 (2001).
6. Loosley, B. C., Andersen, R. J. & Dake, G. R. Total synthesis of cladoniamide G. *Org. Lett.* **15**, 1152-1154 (2013).
7. Bouhlel, A., Curti, C. & Vanelle, P. New methodology for the synthesis of thiobarbiturates mediated by manganese (III) acetate. *Molecules* **17**, 4313-4325 (2012).
8. Tan, X.-M., Lai, Q.-M., Yang, Z.-W. & Cui, H.-L. $La(OTf)_3$ catalyzed synthesis of α-aryl tetrasubstituted pyrroles through [4+1] annulation under microwave irradiation. *Tetrahedron Lett.* **58**, 163-167 (2017).
9. Mital, A., Murugesan, D., Kaiser, M. & Gilbert, I. H. Discovery and optimisation studies of antimalarial phenotypic hits. *Eur. J. Med. Chem.* **103**, 530-538 (2015).
10. Jiao, L., Hao, E. & Smith, K. M. Improved synthesis of functionalized 2,2'-bipyrroles. *J. Org. Chem.* **72**, 8119-8122 (2007).
11. Brauer, D. J., Hingst, M., Kottsieper, K. W. & Sheldrick, W. S. Water soluble phosphines: Part XV. Syntheses of multiply functionalized and chiral phosphine ligands by Pd-catalyzed P-C and C-C coupling reactions. *J. Organomet. Chem.* **645**, 14-26 (2002).
12. Yao, L., Nie, H., Zhang, D. & Zhang, S. Chiral ferrocenyl *N*,*N* ligands with intramolecular hydrogen bonds for highly enantioselective allylic alkylations. *ChemCatChem.* **10**, 804-809 (2018).
13. Feng, B., Pu, X.-Y., Liu, Z.-C. & Chen, J.-R. Highly enantioselective Pd-catalyzed indole allylic alkylation using binaphthyl-based phosphoramidite-thioether ligands. *Org. Chem. Front.* **3**, 1246-1249 (2016).
